# Low Molecular Weight Multistate Photoswitches Based on Simple Norbornadiene‐Triazine Scaffolds

**DOI:** 10.1002/anie.202507999

**Published:** 2025-07-16

**Authors:** Daniel Krappmann, Adrian J. Müller, Erik J. Schulze, Harald Maid, Andreas Dreuw, Andreas Hirsch

**Affiliations:** ^1^ Department of Chemistry and Pharmacy Friedrich‐Alexander‐Universität ErlangenNürnberg NikolausFiebiger Straße 10 91058 Erlangen Germany; ^2^ Interdisciplinary Center for Scientific Computing Universität Heidelberg Im Neuenheimer Feld 205 A 69120 Heidelberg Germany

**Keywords:** Energy conversion, Information storage systems, Multistate photoswitches, Norbornadiene, Photochemistry

## Abstract

We have synthesized and characterized a series of simple norbornadiene(NBD)‐triazine architectures, including multistate photoswitches with unprecedentedly high information storage densities. The simple mono‐NBDs served as suitable model systems to investigate the underlying absorption and switching characteristics. To increase the complexity stepwise, a *bis*‐NBD derivative with a symmetric substitution pattern was investigated next. By combining different NBD substituents with varying electron demands, two asymmetric compounds, one *bis*‐NBD and one *tris*‐NBD, were prepared and investigated. In the case of the *tris*‐NBD, the selective switching of the individual NBD chromophores is hampered by the too closely related optical properties of all three NBD units. On the other hand, the asymmetric photoswitch system containing two NBD‐substituents fulfilled the requirements of a selectively addressable multistate system with an extremely high information storage density. Nearly all possible NBD/quadricyclane (QC) combinations could be realized here, including their reversible interconversion and the respective protonated forms. Quantum chemical calculations corroborated our experimental findings.

## Graphical abstract

Our new polyswitchable norbornadiene‐triazine hybrids are promising candidates for molecular based data storage. These small, low‐molecular‐weight molecules can be both symmetric or contain different NBD chromophors and exhibit an unprecedently high variety of distinct switching states. By incorporating acid/base‐responsive moieties, the number of achievable states can be doubled.

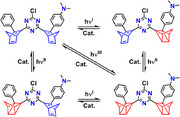

## Introduction

**Scheme 1:**
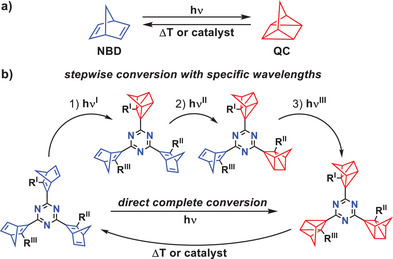
a) Basic principle of the NBD/QC conversion using light for the forward and heat or a catalyst for the back‐conversion. b) Schematic representation of the NBD/QC‐triazine multistate switches. The conversion can be induced stepwise using different wavelengths or at once using a single wavelength. Back‐conversion can be initiated via thermal treatment or with a catalyst. The number of potentially available switching states can be doubled by protonation of, for example, an amine‐containing ligand R.

The norbornadiene/quadricyclane (NBD/QC) interconversion (Scheme 1a) represents a well‐investigated photoswitch system.^[^ ^1^, ^2^, ^3^, ^4^ ^]^ The energy‐lean NBD can be converted to the much more strained and energy‐rich QC form by light. The corresponding back‐conversion, accompanied by a release of energy, can be accomplished, *inter alia*, by metal‐containing catalysts on demand (Scheme 1a).^[^ ^5^, ^6^ ^]^ We have recently demonstrated that the covalent attachment of electron acceptors, such as rylenes or fullerenes, enables the photochemically induced back‐conversion of the QC to the NBD scaffold. Interestingly, the latter reconversion process can be induced at longer wavelengths (lower energies) than the initial QC formation.^[^ ^7^, ^8^, ^9^, ^10^ ^]^ This concept of bi‐directional photo‐switching also has excellent potential for the development of information storage applications in closed *write*, *read*, and *erase* cycles.^[^ ^11^, ^12^, ^13^ ^]^ The complexity and information density can, in principle, be increased if two or more NBD/QC units are bound to one central electron acceptor platform as sketched in Scheme 1b. A few examples of oligo‐NBD derivatives that are unsuitable, however, for such a well‐defined information management goal have been reported.^[^ ^4^, ^14^, ^15^ ^]^ In this regard, a series of non‐NBD/QC‐based multiswitch systems have been synthesized and investigated previously, potentially suitable as information storage candidates.^[^ ^16^, ^17^, ^18^, ^19^, ^20^, ^21^, ^22^, ^23^, ^24^ ^]^ These are based on various combinations of azobenzene, spirane, and dihydroazulene/vinylheptafulvene (DHA/VHF) switches. In some cases, additional introduction of acid/base‐responsive moieties was built to increase the number of available interconversion states. However, these precedents are characterized by relatively large and complex architectures, making their synthesis rather demanding and limiting their potential for practical applications.^[^ ^25^, ^26^ ^]^

We present a straightforward approach to easily accessible *bis*‐ and *tris*‐NBDs coupled to a triazine platform, which can be reversibly converted to a rich series of switching states (Scheme 1b). Given their very high functional and switching density, they exhibit a remarkably low molecular weight. Larger triazine/oligo‐NBD hybrids with no direct covalent connection of the NBD and triazine units and much less favorable switching and storage properties have been reported before.^[^ ^27^ ^]^ In our new systems, the number of potentially and practically available states based on a single photoswitch type is unprecedented to the best of our knowledge. Furthermore, our molecules exhibit the highest information storage density per molecular weight reported so far.^[^ ^28^ ^]^

## Results and Discussion

### Synthesis

**Scheme 2:**
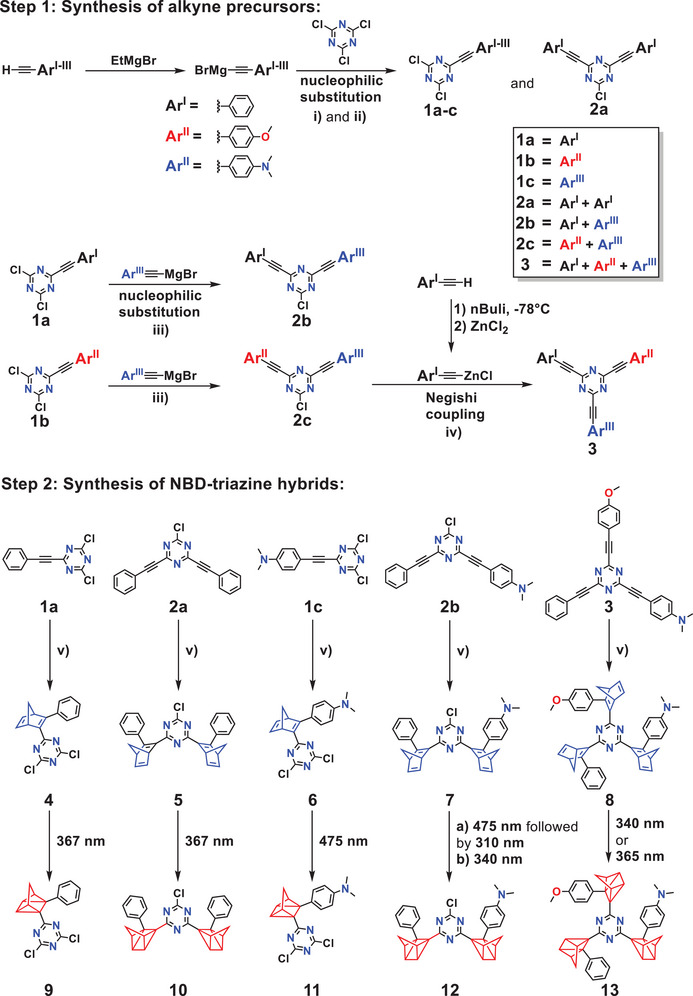
Synthesis of NBD‐triazine hybrids **4**–**9** and their photo‐conversion to the QC‐analogues **9**–**13**. Step 1: Synthesis of the acetylene precursors. Step 2: Diels–Alder reaction of the previously prepared acetylenes **1**–**3** to give the triazine‐NBD hybrids **4**–**8**. i) for 1a–c: alkynyl magnesium bromide (1.5 eq.), THF/Et_2_O 1:2 v/v, 0 °C → rt, 40–120 h; ii) for 2a: acetyl magnesium bromide (4.5 eq.), THF/Et_2_O 1:2 v/v, 0 °C → rt, 40–120 h; iii) alkynyl magnesium bromide (1.5 eq.) THF/Et_2_O 1:2 v/v, 0 °C → rt, 42–120 h; iv) alkynyl zinc chloride (4.0 eq.), Pd(PPh_3_)_4_ (5 mol%), THF/NMP 1:1 v/v, 50 °C, 20 h → rt; v) Diels–Alder reaction: cyclopentadiene (15–25 eq.), toluene, 120–180 °C, 2–38 h, 12–68%. The conversion of the NBD species into the corresponding QC derivatives **9**–**13** was achieved using distinct wavelengths. 367 nm for the conversion of **4** and **5**, 475 nm for **6**, first 475 nm followed by 310 nm (or vice versa) for the stepwise conversion, or 340 nm for direct conversion of **7** to **12**, and 340 or 365 nm for the isomerization of **8** to **13**.

The synthetic pathways toward the targeted multistage switches are depicted in Scheme 2. First, in a nucleophilic aromatic substitution, the mono‐substituted cyanuric acetylenes **1a–c** were generated. Therefore, cyanuric chloride was reacted with the corresponding alkynylmagnesium bromide prepared in situ by the treatment of phenyl acetylene, 4‐methoxyphenyl acetylene, and 4‐(*N*,*N*‐dimethylamino)phenyl acetylene with EtMgBr, using a literature procedure.^[^ ^29^ ^]^ To achieve control over the substitution degree at the triazine core, the stoichiometry of the reaction partners was carefully adjusted.^[^ ^30^ ^]^ For mono‐substitution, 1.5 equivalents of the alkynyl magnesium bromide were used, while for *bis*‐substitution product **2a**, 4.5 equivalents of phenylethynyl magnesium bromide were required. Starting from the mono‐substituted triazine‐acetylenes **1a–c**, a second substitution reaction was performed to generate the asymmetric derivatives **2b** and **2c**. Since complete *tris*‐substitution leading to **3** was not successful with this approach, even with catalytic support mimicking a Kumada‐like cross‐coupling reaction, an alternative pathway was chosen. A procedure introduced by Zhang and co‐workers was successful.^[^ ^31^ ^]^ In this way, the *tris*‐substituted compound **3** was obtained by the reaction of **2c** with phenylacetylene zinc chloride. The next step (step 2, Scheme 2) was the generation of the triazine hybrids (**4**–**8**). Based on a general procedure,^[^ ^1^, ^32^ ^]^ these target molecules were synthesized via Diels–Alder reaction with cyclopentadiene. All reactions were carried out in the dark to prevent photo‐isomerization to the respective QCs. In this way, the mono‐ and diphenyl substituted derivatives **4** and **5**, as well as the mono‐dimethylamine substituted **6**, were obtained in good yields. In the case of derivative **6** containing the electron‐donating dimethyl amine substituent, higher reaction temperatures of up to 170 °C were required for appreciable conversion. However, higher reaction temperatures led to the addition of a second cyclopentadiene, forming side products that cannot be separated.^[^ ^33^ ^]^ Therefore, careful temperature control and reaction monitoring using gas chromatography coupled to mass spectrometry (GC‐MS) analysis was employed. The asymmetric *bis*‐ and *tris*‐substituted compounds **7** and **8** could be prepared in moderate yields. Characterization of the target compounds was done via NMR and UV/vis spectroscopy and high‐resolution mass spectrometry. An overview of the absorption characteristics of all prepared molecules is provided in Table 1. Separating diastereoisomers stemming from the attachment of two or three chiral NBD units has not been carried out. It can be expected that different diastereoisomers give rise to almost the same optical and switching properties. By application of specific wavelengths, conversion into the respective QC derivatives **9**–**13** can be achieved, which will be discussed below.

**Table 1:** Overview of the spectroscopic data of all photoswitches measured in MeCN. Values in parentheses are the extinction coefficients (given in M^−1^ cm^−1^) for the respective wavelength.

Molecule	λ_max_ ^a)^ [nm]	λ_onset_ ^b)^ [nm]
**4**	363 (10 900) / 367 (11 400)^c)^	446
**9**	309 (8000)^c)^	/
**5**	229 (16 700), 356 (8900)	443
**10**	292 (7200)	/
**6**	482 (17 600)	594
**11**	264, 266, 267	/
**7**	229 (17 300), 270 (17 000), 324 (10 500), 351 (10 200), 455 (12 400)	577
**12b**	235 (17 200), 264 (21 000), 343 (10 600)	/
**12**	263 (23 000)	/
**8**	242 (22 300), 254 (21 400), 300 (13 900), 359 (14 600), 431 (9700)	531
**13**	235 (21 500), 261 (22 800)	/

^a)^ Additional given values for the asymmetric multisubstituted derivatives correspond to the absorptions originating from the additional substituents.

^b)^ Onsets were determined as *log*(ε)  =  2. Therefore, the determination of the onset values for the QC was not possible since leftover NBD species distorted the calculation.

^c)^ Measured in CHCl_3_.

### Investigation of the Interconversion Properties

#### Mono‐and *Bis*‐phenyl‐substituted NBDs 4 and 5 as Model Compounds

**Figure 1:**
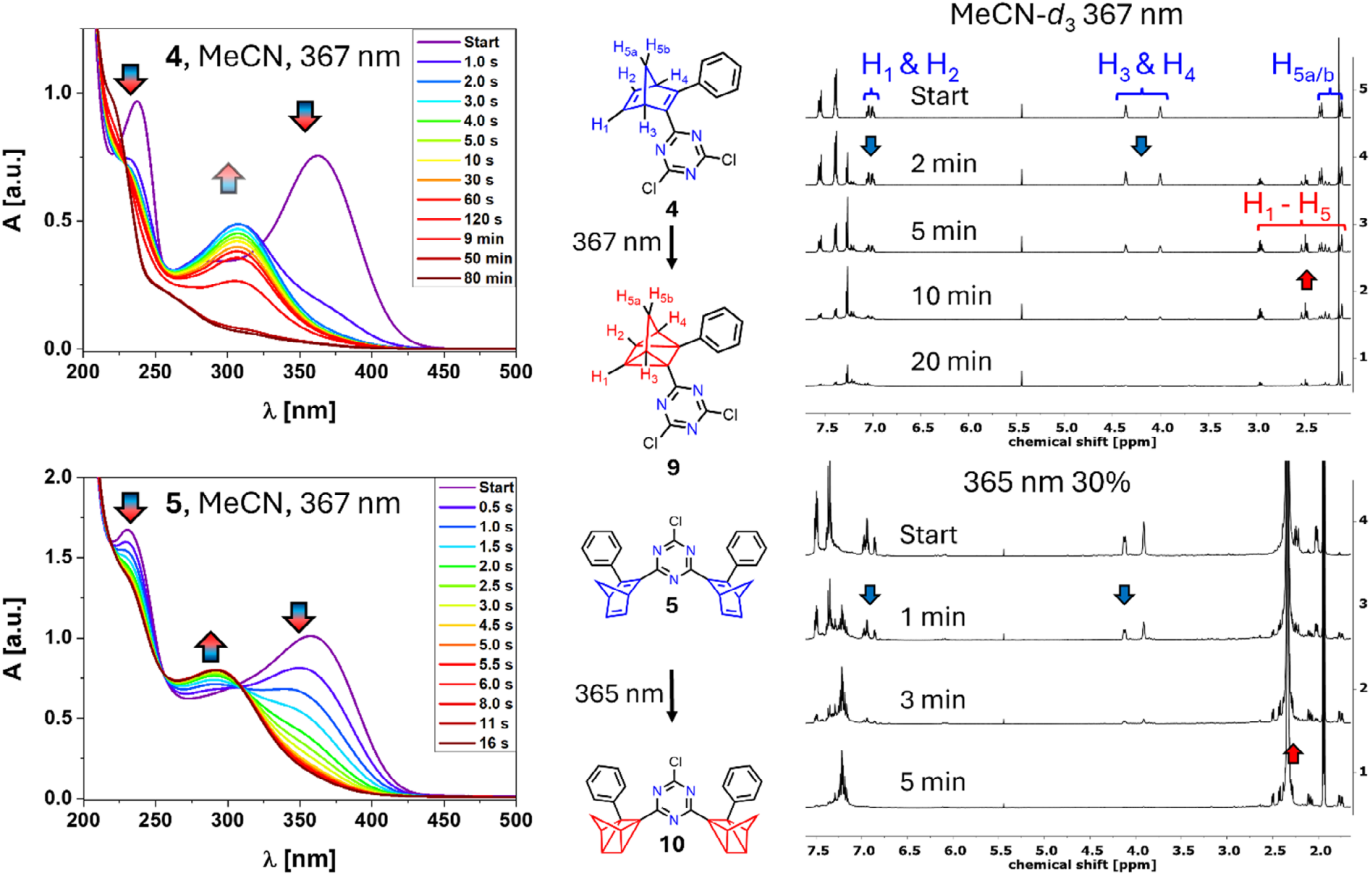
Interconversion of **4** (top) and **5** (bottom) into the QC isomers **9** and **10** monitored by UV/vis spectroscopy (left) measured in MeCN. Right: Corresponding ^1^H NMR monitored interconversion experiments of **4** (top) and **5** (bottom) into their respective QC analogs.

Using previously established methods, we first investigated the photoswitching of the simple mono‐NBD **4** and the symmetrical *bis*‐NBD **5**.^[^ ^3^ ^]^ For this purpose, UV/vis measurements were conducted to determine the absorption properties first. Since we observed pronounced solvent dependencies of the interconversion behavior, we investigated the optical properties in various solvents.^[^ ^34^, ^35^ ^]^ As an example for the general analysis, we here show and describe the conversion behavior in MeCN. The UV/vis monitoring of the switching process of **4** and **5** is displayed in Figure 1. In general, the purple “start” spectra reflect NBD behavior, while the dark red curves resemble the features of the converted QC analogues. The corresponding rearrangement was initiated by irradiation into the absorption maximum of the NBD derivative. For derivative **4**, the two NBD related absorptions at λ_max_ = 363 and 238 nm decrease upon irradiation. Simultaneously, a new, probably QC‐related absorption arises at λ_max_ = 308 nm after a few seconds (light blue line). However, no isosbestic points were found. Prolonged irradiation led to a decrease in the complete absorption, indicating photodecomposition of **9**. In the case of the *bis*‐NBD **5**, opposite results were found. Clean conversion of **5** into **10** was observed after 11 s. Longer irradiation times resulted in just minor decomposition (see Supporting Information). Again, a complete decrease of the NBD absorption at λ_max_ = 358 and 231 nm, accompanied by an increase of the QC absorption at λmax = 292 nm, was found. The lack of isosbestic points can be explained by the formation of an intermediate bearing one NBD and one QC substituent.

To provide comparability to the conducted NMR experiments commonly performed in CDCl_3_, similar UV/vis monitored measurements were conducted in CHCl_3_. Here, **4** was converted entirely to **9** after 3 s using a 367 nm LED. The presence of isosbestic points indicates clean conversion, and even after prolonged irradiation up to 8 min, no significant photodecomposition could be observed (for details, see Supporting Information). For **5**, initial QC formation can be observed after 10 s, accompanied by photodecomposition after prolonged irradiation (13 min).

The NBD/QC conversions were also monitored by ^1^H NMR spectroscopy. The applied concentrations are about 100 times higher compared to those used for the UV/vis measurements. This results in extended irradiation times necessary to achieve complete isomerization. For the monitoring process, the decrease of the signals of bridgehead protons *H*_3_ and *H*_4_ of the NBD moieties in the region between 4.40–3.99 ppm and the appearance of the QC mutiplet signals of the bridge at 2.35–2.29 and 2.12–2.09 ppm (*H*_5a_ and *H*_5b_, slightly overshadowed by the residual solvent signal) was followed. In the case of **4** complete conversion to **9,** in MeCN‐*d*_3_ was observed, contrary to the findings seen in the UV/vis experiment. Nearly quantitative isomerization to **9** was obtained after 10 min. After 20 min, however, decomposition started to set in (Figure 1). In CDCl_3_, complete isomerization was accomplished after 20 min, and no photodecomposition was observed (for details, see Supporting Information). Leaving the sample at room temperature (rt) for 12 h led to partial back‐conversion to **4**. By the addition of the Co^II^ porphyrin catalyst (**Por**, [5‐(p‐Carboxyphenyl)‐10,15,20‐(p‐*tert*‐butyltriphenylphenyl)porphyrinato] cobalt (II)), which is known to catalyze the back‐conversion,^[^ ^1^, ^36^ ^]^ quantitative reconversion to **4** occurs (see Supporting Information). For **5**, fast and decomposition‐less conversion towards **10** was observed after 5 min in MeCN‐*d*_3_ (Figure 1, bottom). For the conversion, a 365 nm LED at 30% power (15 W), which is significantly stronger than the 367 nm LED (1.0 W), was used to speed up the conversion (for more details, see Supporting Information). In CDCl_3_, complete conversion was found after 5 min without any decomposition. After leaving the solution for 8 h at rt, no thermal back‐reaction took place, whereas the addition of **Por** led to recovery of **5**. Methylene chloride (CD_2_Cl_2_) and toluene‐*d*_8_ were tested as additional solvents. In both cases, fast and clean conversion was observed (for the corresponding spectra see Supporting Information).

**Figure 2:**
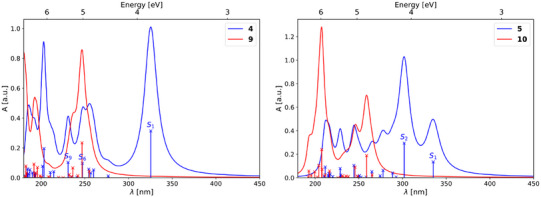
(TD‐)DFT predicted UV/vis spectra of **4** and **5** (blue) and their fully switched counterparts **9** and **10** (red), respectively.

(TD‐)DFT computations reveal that, for compound **4** (Figure 2, left), the NBD absorption at 367 nm corresponds to the first singlet excited state S_1,_ which is a π–π* transition. The attachment and detachment densities show the impact of the push–pull substitution pattern, i.e., electron density transitions from the phenyl to the triazine moiety. Further, this transition induces the photoisomerization to QC.^[^ ^37^ ^]^ The absorption band at 238 nm most likely corresponds to *S*_9,_ which is another π–π* transition where the excitation occurs from a different molecular π‐orbital than before for *S*_1_. The shoulder in the experimental spectrum around 290 nm probably arises from multiple n–π* states with major contributions from the nonbonding lone pairs of the nitrogen atoms within the triazine subsystem. Further, the computed spectrum of **9** predicts the main absorption band between the two maxima of **4** and in the range of the visible shoulder. The simulated spectrum of **5** (Figure 2, right) shows two absorption bands above 300 nm. Since the two NBD‐Ph moieties are in spatial proximity and exhibit nonorthogonal transition dipole moments, they show exciton coupling in the calculations, yielding two states with different energies, one below and one above compared to the *S*_1_ of **4**, and different oscillator strengths. At rt and in solution, however, these peaks are further broadened and other conformers are present as well with slightly distinct photophysics leading to coalescence. Hence, the experimental spectrum exhibits only one broad peak and is very similar to the singly substituted **4**. Nonetheless, the first absorption band is broader for **5**, which is a consequence of the above‐described effects.

#### Dimethylamino‐Substituted NBD 6

Next, the dimethylamino‐NBD **6** was prepared, exhibiting a pronounced redshift of the absorption features. However, the corresponding clean QC conversion to **11** did not occur, considering UV/vis investigations. During the ^1^H NMR spectroscopic monitoring of the irradiation process, photodecomposition and formation of unidentified side products took place irrespective of the solvent (MeCN, chloroform, and benzene; see Supporting Information). Within the simulated spectrum (SI), the significant bathochromic shift in comparison to **4** is well reproduced and arises from the electron‐donating effect of the dimethylamine group.

#### Asymmetric *Bis*‐NBD 7: Discussion of the Neutral state Conversions

**Figure 3:**
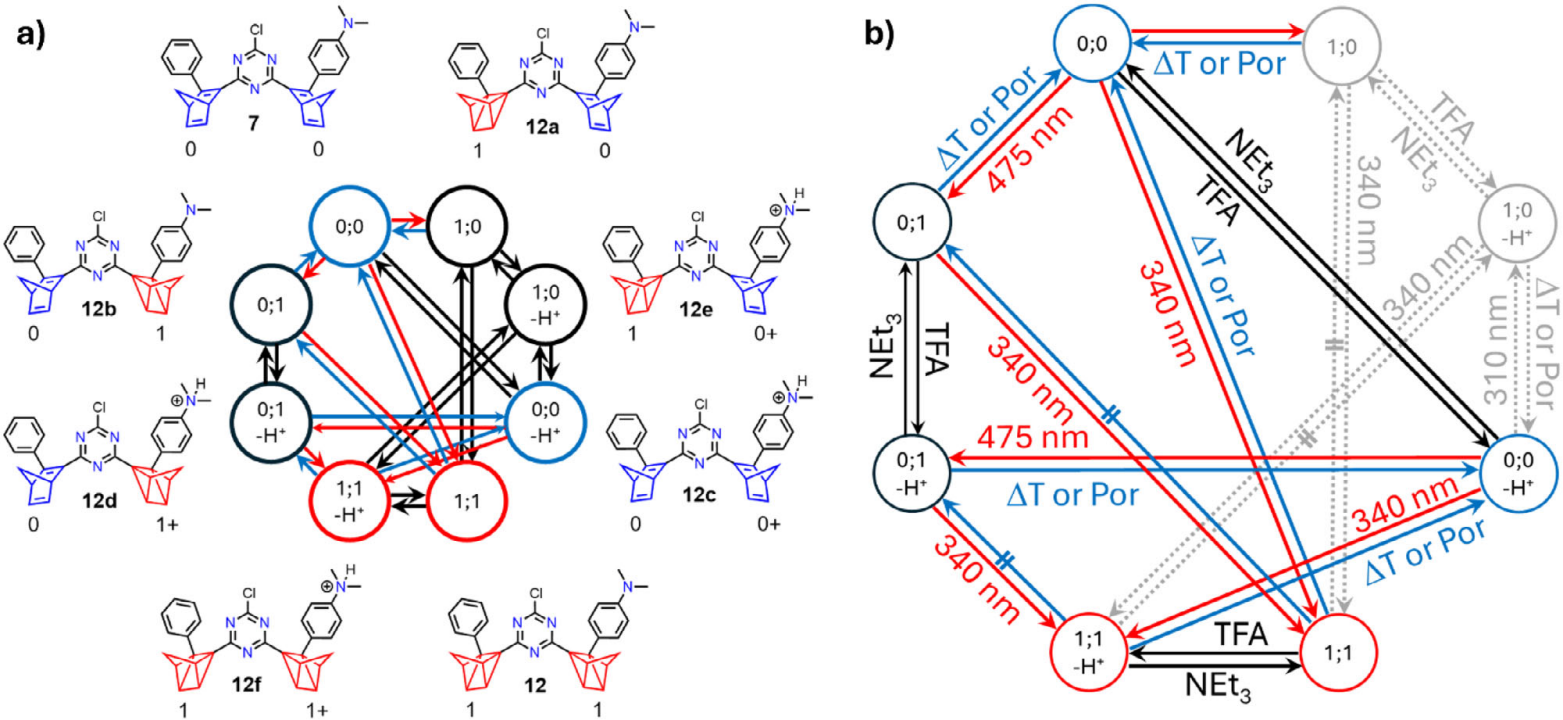
a) Schematic representation of the eight different states accessible for the photoswitch system **7**/**12**. b) Reaction conditions for the interconversion necessary. All indicated conversions can be achieved by using a single input. Conversions requiring more than one input are not shown for clarity. Red arrows indicate the formation of a NBD to a QC species, while blue arrows indicate the back‐conversion. Black arrows are used to show reversible protonation. Inaccessible species and conversions are colored in grey.

The asymmetrical *bis‐*NBD **7** with intermediate complexity turned out to be the best candidate in this series to precisely *write*, *read*, and *erase* a whole variety of switching states, including those of protonated forms. The complete conversion to *bis*‐QC **12** was targeted either by the application of one or two subsequent optical stimuli at suitable wavelengths. Consequently, a total of 2*^n^* = 4 states (with *n* = number of photoswitchable moieties) were specifically addressed (Figure 3). These are the parent *bis*‐NBD **7** (*0;0*), the completely converted *bis*‐QC state **12** (*1;1*), and the two partially converted states **12a** (*1;0*) and **12b** (*0;1*). Moreover, via reversible protonation of the dimethylamino functionality in **7**, the possible number of addressable and interconvertible isomers increases to eight total states (Figure 3).

**Figure 4:**
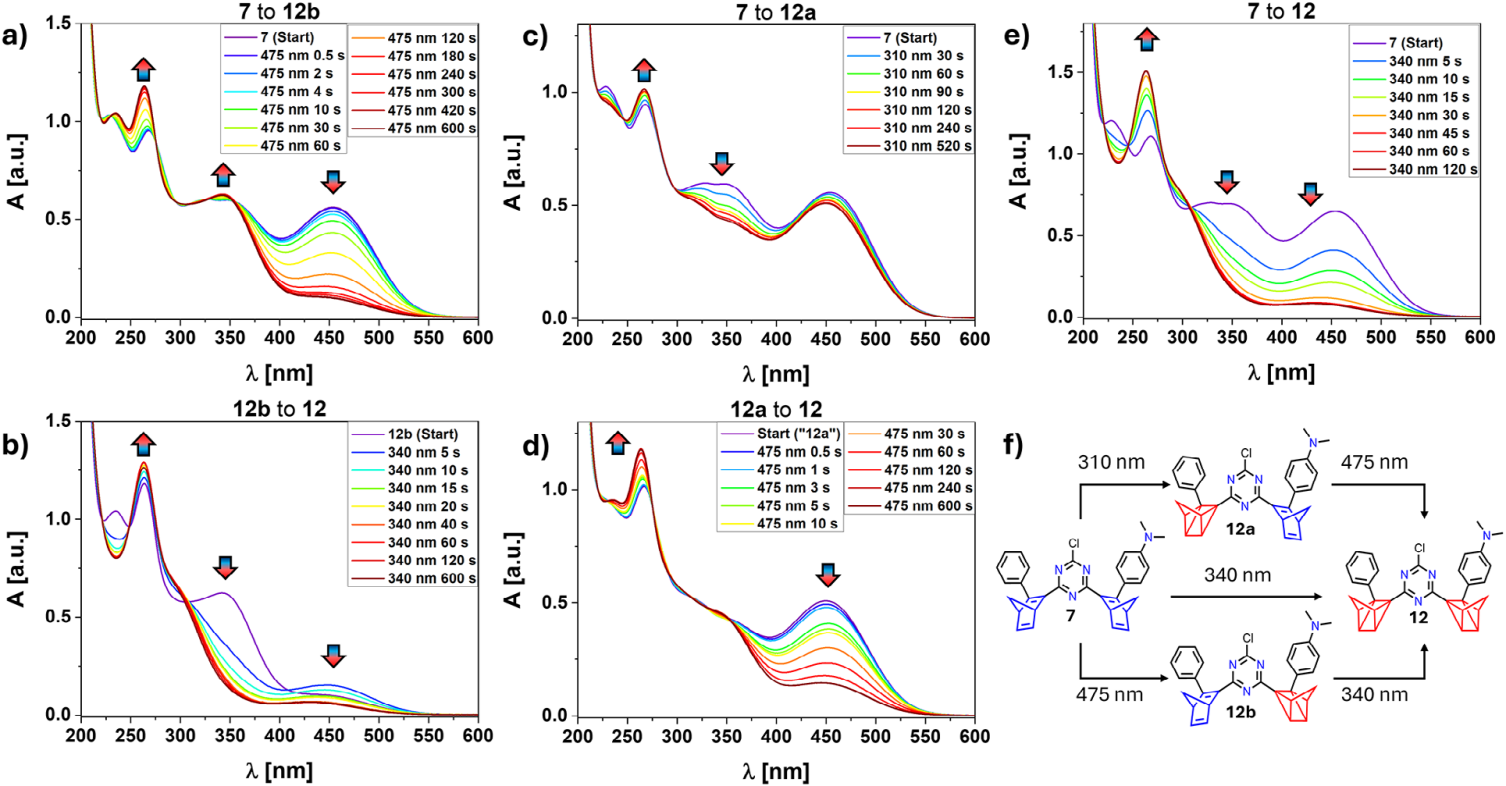
UV/vis monitoring of the photo‐switching of **7** in MeCN using different combinations of LEDs. a) Irradiation using 475 nm for the conversion to **12b**; b) Subsequent irradiation at 340 nm led to 12; c) Irradiation at 310 nm to approach **12a**; d) Subsequent irradiation using 475 nm to obtain a mixture of **12** and **12b**; e) Direct irradiation at 340 nm to get **12**; f) Schematic representation of the irradiation pathways.

We will first discuss the interconversion of neutral species **7** (*0;0*) leading to **12a** (*1;0*), **12b** (*0;1*), and **12** (*1;1*) using similar methods as described above. As expected, the optical absorption spectrum of **7** (Figure 4c, purple spectrum) exhibits two major absorption features in the visible region at λ_max _= 453 nm (dimethyamino‐phenyl‐NBD‐originated) and at λ_max _= 353 nm (phenyl‐NBD‐originated). First, the stepwise conversion of **7** (*0;0*) over **12b** (*0;1*) to **12** (*1;1*) was attempted (Figure 4a,b). Indeed, irradiation of **7** (*0;0*) at λ = 475 nm led to the formation of **12b** (*0;1*) after 600 s. This is reflected by the decrease of the amino‐centered absorption at λ_max_ = 453 nm and the simultaneous increase of the amino‐QC absorption at λ_max _= 343 nm. In a subsequent switching experiment, irradiation into the newly formed band at λ_max_ = 343 nm with a 340 nm LED resulted in the complete conversion into **12** (*1;1*) after another 600 s (Figure 4b). Then, stepwise conversion over the phenyl sided NBD **12a** as an intermediate was attempted (Figure 4c,d). Irradiation of **7** (*0;0*) with the shorter wavelength LED at 310 nm partially formed **12a** (*1;0*). Upon this treatment, the absorptions at λ_max_ = 323 and 353 nm decrease significantly, while the main amine absorption at 453 nm remains unchanged. This first isomerization process was finished after 520 s since no further change in absorption was observed. However, complete conversion into **12a** cannot be proven since no complete decrease of the previous absorption properties was found. Therefore, a photostationary state (PSS) composed of a mixture of **7** and **12a** can probably be observed in Figure 4c. Subsequent irradiation at λ = 475 nm resulted in the complete conversion of the amine NBD, leading mainly to **12** (*1;1*) (Figure 4d). By comparison of the red spectra of Figure 4d to Figure 4b, the remaining absorption around 350 nm is predominant. Therefore, in this experiment, for the final spectrum, a mixture of **12** (*1;1*) and **12b** (*0* *;1*) must be considered. Lastly, direct conversion of **7** to **12** was investigated. Since both the starting molecule **7** (*0;0*) and the first step intermediates **12a** (*1;0*) and **12b** (*0;1*) exhibit absorption features in the 340 nm region also direct switching of both sides of *bis*‐NBD **7** by irradiation with 340 nm was carried out (Figure 4e). Indeed, complete conversion into **12** (*1;1*) was observed after 120 s.

**Figure 5:**
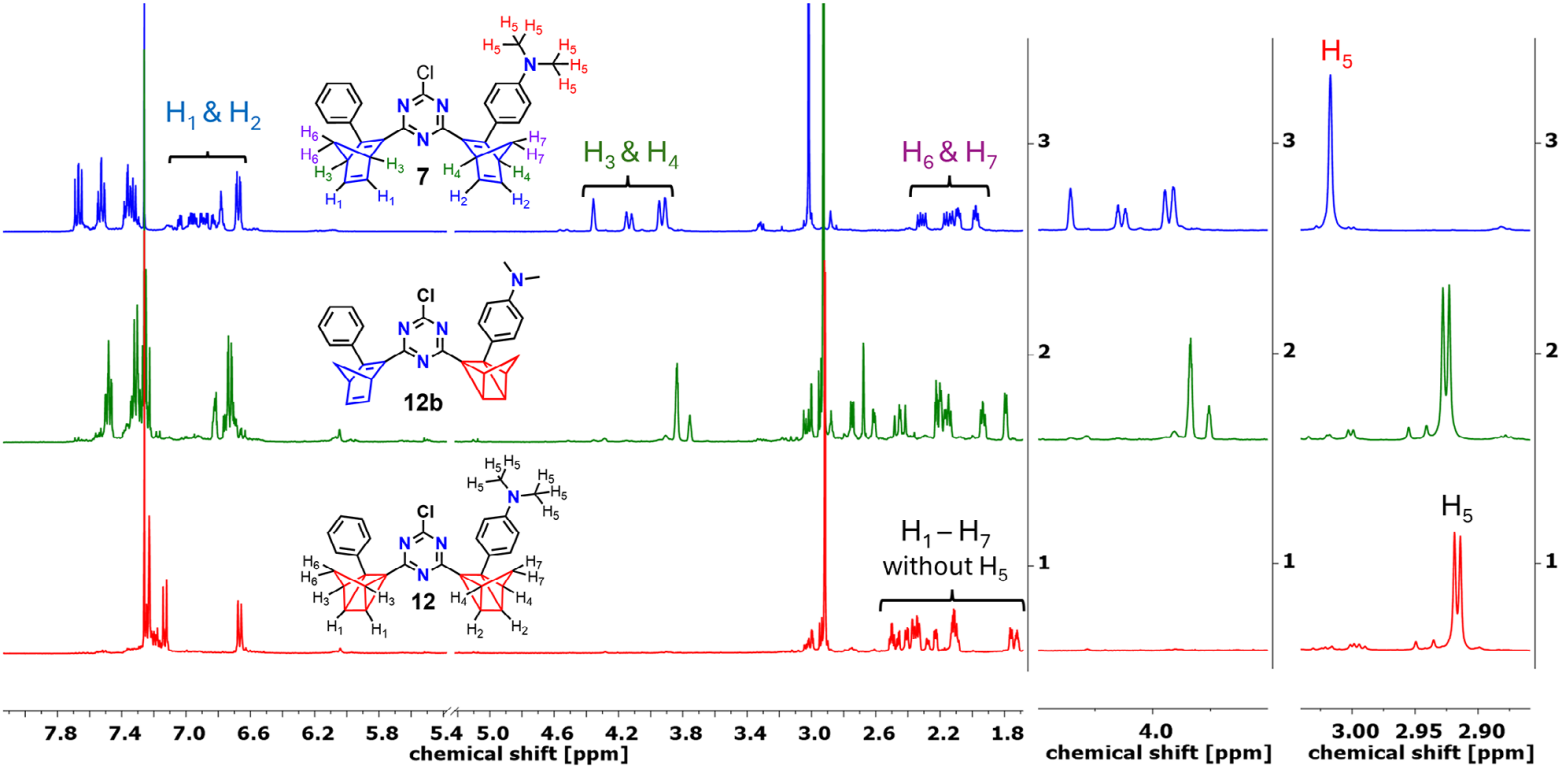
^1^H NMR spectra of **7** (blue), **12a** (green), and **12** (red) measured in CDCl_3_. Middle and right: Partial spectrum of the bridgehead region and the NMe_2_ signals, respectively. The splitting of the *H*_5_ signal can be explained by the presence of the two diastereoisomers, which provide coincidental isochrony in case of **7**.

We also monitored the identical photochemical rearrangements using ^1^H NMR spectroscopy. For this purpose, we used CDCl_3_ as solvent since in MeCN‐*d*_3,_ *bis*‐NBD **7** was not soluble enough to achieve satisfactory spectra. The entire monitoring of each switching experiment is provided in the Supporting Information. In Figure 5, the spectra of **7** (*0;0*) and those of the rearrangement **12b** (*0;1*) and **12** (*1;1*) obtained directly after photoconversion are depicted. **12a** (*1;0*) could not be obtained in NMR scale. The conversion of **7** (*0;0*) to **12b** (*0;1*) was accomplished using the 475 nm LED. Vanishing of the signal sets for the bridgehead signals of both NBD moieties (*H*_3_ and *H*_4_) between 4.37–3.90 ppm, together with the formation of a new single signal set corresponding to H_3_ of the NBD scaffold in **12b** at 3.86–3.74 ppm, was found. As in the optical monitoring studies, the subsequent irradiation of **12b** (*0;1*) at 340 nm afforded **12** (*1;1*). The ^1^H NMR spectrum of **12** clearly reveals the absence of any olefinic signals between 4.37 and 3.74 ppm, which would indicate the NBD units (Figure 5). The splitting of the *H*_5_ signal can be explained by the presence of the two diastereoisomers, which provide coincidental isochrony in the case of **7**. Similar conclusions can be drawn from ^13^C NMR spectroscopy (see Supporting Information). Reversibility by adding catalytic amounts of **Por** was successful in recovering clean **7** after removal of **Por** (see Supporting Information).

#### Asymmetric *Bis*‐NBD **7**: Increasing the Number of Possible States by Reversible Protonation

**Figure 6:**
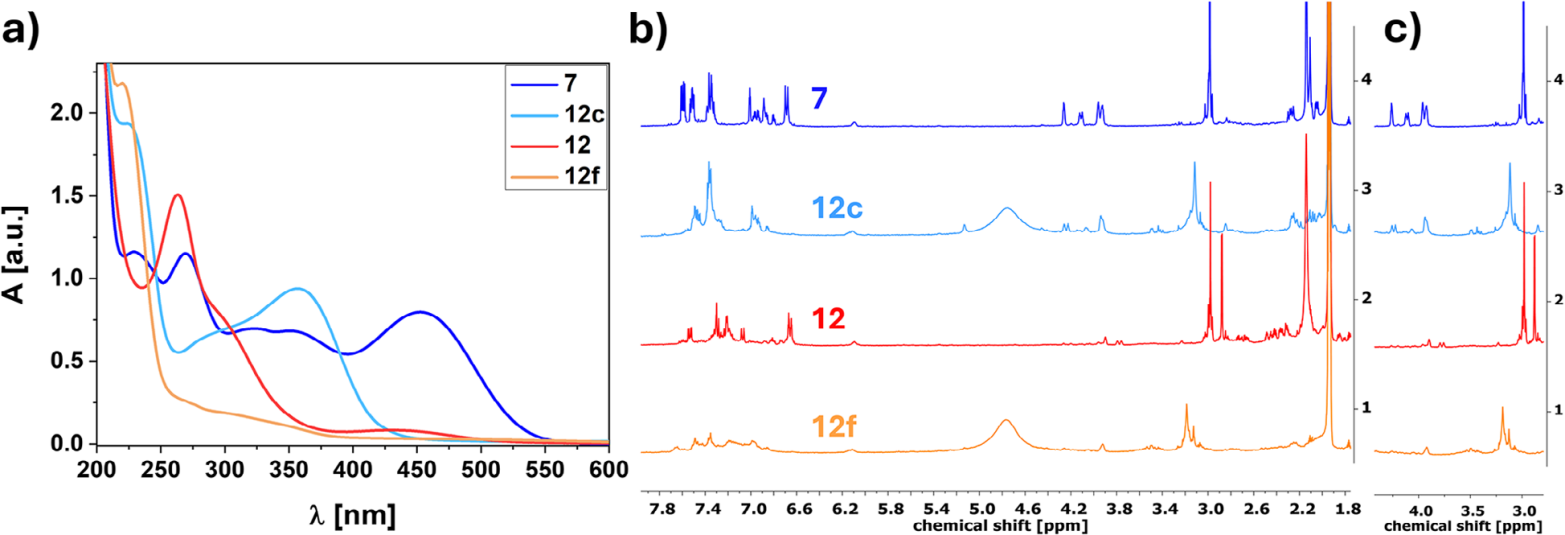
Investigation of the switching capability of **7**: a) UV/vis spectra of **7** and **12** and their protonated analogs **12c** and **12f** measured in MeCN. b) Corresponding ^1^H NMR spectra measured in MeCN‐d_3_. c) Significant section of the bridgehead region and the NMe_2_ signals.

By applying another stimulus, namely, protonation/deprotonation of the NMe_2_ group, the number of possible switching states can be doubled to eight (Figure 3). In addition to the states discussed above, the protonated analogs **12c** (*0;0‐H* ^+^), **12d** (*0;1‐H* ^+^), **12e** (*1;0‐H* ^+^), and **12f** (*1;1‐H* ^+^) are in principle available. Since **12a** (*1;0*) was not accessible in macroscopic quantities, attempts to obtain **12e** (*1;0‐H* ^+^) were discarded. These inaccessible states are also schematically depicted in light grey in Figure 3b. First, all endpoint spectra were recorded, which are shown in Figure 6a. Considering the UV/vis experiments (Figure 6a) by protonation of the base state **7** (*0;0*) (blue line) with trifluoroacetic acid (TFA), a strong blueshift of the absorption maximum to 357 nm for **12c** (*0;0‐H* ^+^) (light blue line) was found which correlates to the weakening of the electron donating character. The same was found for the respective QC analogs **12** (*1;1*) (red line) and **12f** (*1;1‐H* ^+^) (orange line), namely a hypsochromic shift from 264 to 221 nm. For **12f** (*1;1‐H* ^+^), no other significant absorption can be found at all. Therefore, investigation of solvents with self‐absorption in the short wavelength region was neglected. Since all of the four species basically provide absorption at different wavelengths, potential application as a keypad‐lock could be considered as described before for another amine‐substituted NBD derivative.^[^ ^38^ ^]^ The lacking single side‐switched species **12b** (*0;1*) and **12d** (*0;1‐H* ^+^) were omitted from Figure 6 for clarity and are just shown in the Supporting Information. The absorption maxima found for **12b** (*0;1*) at 343 and 264 nm disappear, and a new maximum appears at 354 nm for **12d** (*0;1‐H* ^+^). Further, by comparison of the spectra measured for **12d** (*0;1‐H* ^+^) and **12c** (*0;0‐H* ^+^), a slightly different maximum, combined with a different line shape, can be observed, assigning both spectra to different species (see Supporting Information.).

For further validation, NMR experiments were performed (Figure 6b,c). 10 µL TFA‐*d* were added to a sample of **7** in MeCN‐*d*_3_ resulting in the protonation of the amine group. As the main indication whether the protonation and subsequent deprotonation is possible, the singlet signal at about 3.0 ppm corresponding to the CH_3_ protons of the NMe_2_ group was used. Besides a downfield shift of the latter mentioned signals (Figure 6c), the shape and splitting of the olefinic signals for the bridgehead atoms between 4.2 and 3.8 ppm changes significantly for the protonated species (**12c** (*0;0‐H* ^+^), light blue line). **12** (*1;1*) was obtained in a similar fashion as described before solely by irradiation. Subsequent addition of TFA‐*d* resulted in a significant change of the spectrum (orange line). Pronounced aliphatic QC signals previously found around 2.6–2.2 ppm for **12** (*1;1*) (red line) cannot be observed, which could be overlapped by be the poor baseline. To improve the quality of the spectra, protonation of the respective species and subsequent isolation before re‐dissolving for the NMR experiments was not successful. Since the addition of **Por** did not result in any back‐conversion, decomposition in the concentration necessary for NMR investigation cannot be excluded. However, at the lower UV/vis concentrations, after deprotonation using diisopropylethylamine (DIPEA), through addition of minimal amounts of **Por**, the absorption spectrum of **7** (*0;0*) could be regained. Reversibility of the protonation by alternate addition of TFA followed by DIPEA was checked leading to the same relative absorptions remaining stable over three cycles (Table 2, sequence 1 and see Supporting Information). However, a slight decrease in the maximum absorption was found during each addition step. This is probably a consequence of the sample dilution combined with decomposition.

**Table 2:** Tested input sequences starting from the basic form **7** (0;0). All associated UV/vis spectra were measured in MeCN at rt and provided in the Supporting Information.

Input Sequence	Input^a)^	Resulting Species
**1**	TFA (10 µL) NEt(*i*Pr)_2_ (20 µL) TFA (20 µL) NEt(*i*Pr)_2_ (20 µL) TFA (50 µL) NEt(*i*Pr)_2_ (100 µL) 475 nm **Por**	**12c** (*0;0‐H^+^*) **7** (*0;0*) **12c** (*0;0‐H^+^*) **7** (*0;0*) **12c** (*0;0‐H^+^*) **7** (*0;0*) **12b** (*0;1*) /
**2**	340 nm TFA (10 µL) NEt_3_ (20 µL)	**12** (*1;1*) **12c** (*0;0‐H^+^*) **7** (*0;0*)
**3**	340 nm NEt_3_ (10 µL) TFA (20 µL) NEt_3_ (10 µL)	**12** (*1;1*) **12** (*1;1*) **12c** (*0;0‐H^+^*) **7** (*0;0*)
**4** ^b)^	340 nm TFA (10 µL) NEt_3_ (20 µL)	**12** (*1;1*) **12c** (*0;0‐H^+^*) **7** (*0;0*)
**5**	475 nm TFA (10 µL) 340 nm NEt_3_ (20 µL)	**12b** (*0;1*) **12d** (*0;1‐H^+^*) **12f** (*1;1‐H^+^*) /

^a)^ All sequences considering the use of **Por** were conducted using NEt(iPr)_2_ as base to decrease the possibility of coordination to the active metal site preventing the catalytic activity

^b)^ during addition of the specific inputs, the cuvette containing the solution was cooled to 0 °C.

We also investigated the accessibility of specific states via various combinations of alternative routes. For instance, **12f** (*1;1‐H* ^+^) should be available via first switching **7** (*0;0*) to **12** (*1;1*) and subsequent protonation or vice versa. Selective and representative examples of the various inputs that we tested are listed in Table 2. All measurements were conducted in MeCN using **7** as initial form. The concentration of the resulting solution after the addition of acid or base was not adjusted leading to some dilution and thus a slight decrease of absorption. For additional input sequences and the corresponding spectra, we to refer to the Supporting Information.

As initial point for the testing series, reversibility of the protonation was verified, by alternate addition of acid and base (Table 2, sequence 1) as mentioned before. Then, in sequence 2, first photo‐switching from **7** (*0;0*) to **12** (*1;1*) and then protonation to **12f** (*1;1‐H* ^+^) was targeted. As expected, irradiation at 340 nm yielded **12** (*1;1*), but the subsequent addition of TFA did not result in the formation of **12f** (*1;1‐H* ^+^) but instead caused the rearrangement to **12c** (*0;0‐H* ^+^). The addition of base ultimately resulted in recovery of **7** (*0;0*). The invers process of first adding TFA and subsequent irradiation at 340 nm indeed led to **12f** (*1;1‐H* ^+^) (compare Supporting Information). Sequence 3 and 4 were considered to investigate the influence of the acid/base reaction. Irradiation at 340 nm led to **12** (*1;1*) and subsequent addition of NEt_3_ did not result in any change. However, upon subsequent addition of acid, protonated NBD **12c** (*0;0‐H* ^+^) was generated rather than the expected QC **12f**. Addition of base again resulted in recovery of **7** (*0;0*) (Table 2, sequence 3). To exclude potential thermally induced back reactions initiated by the heat generated by the acid–base reaction in sequence 4, the cuvette containing the solution was cooled to 0 °C using an ice bath. Again, the same conversions as described in sequences 2 and 3 were observed, namely upon addition of acid, reconversion of QC to NBD is indicated. Thus, besides protonation, acid‐catalyzed back‐reaction might take place, which was reported before by Bren and co‐workers.^[^ ^14^ ^]^ Therefore, to reach **12f** (*1;1‐H* ^+^), the input sequence seems to be essential. However, by application of first protonation the absorption spectrum of **12c** (*0;0‐H* ^+^), change which hampers selective targeting of the amine side at long wavelength. To this end, in sequence 5, stepwise conversion into **12f** (*1;1‐H* ^+^) was accomplished by first irradiation of **7** (*0;0*) with 475 nm to get **12b** (*0;1*). Subsequent addition of TFA yielded **12d** (*0;1‐H* ^+^), which was converted with 340 nm light to **12f** (*1;1‐H* ^+^). The subsequent addition of base, however, resulted in the formation of an unidentified species rather than recovery of either neutral species **7** or **12**. For the single‐sided conversions, including **12b** and **12d,** we want to refer to the Supporting Information.

**Figure 7:**
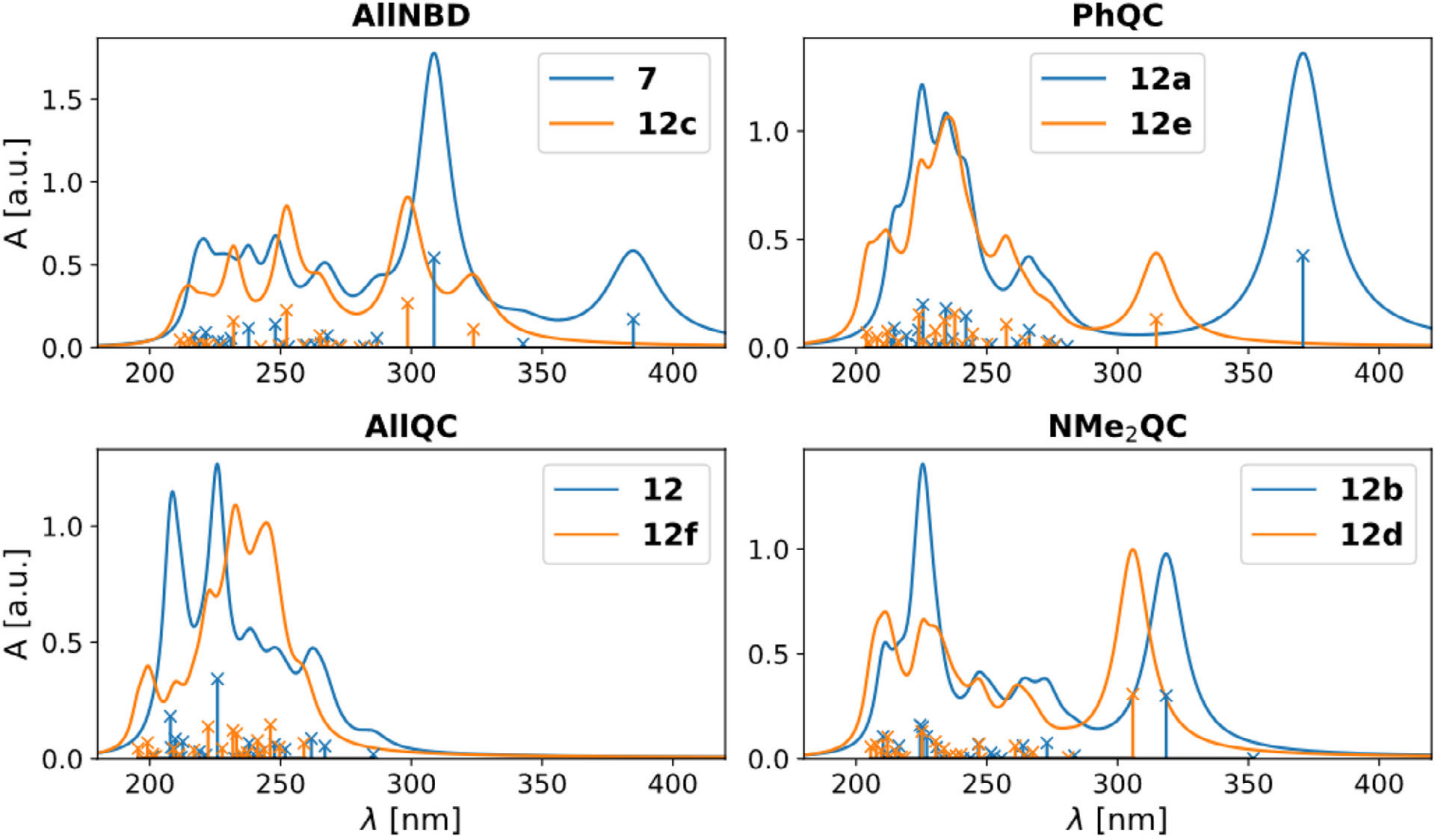
(TD‐)DFT predicted UV/vis spectra of all conversion possibilities of **7**, **12a**, **12b**, and **12** and their protonated analogs **12c**, **12d**, **12e**, and **12f**. Top left: (0;0), top right: (1,0), bottom left (1,1), bottom right: (0,1).

The computations for **7** show that the first two experimental absorption bands (453 and 353 nm) correspond to π–π* transitions, *S*_1_ and *S*_3_, respectively. The *S*_1_ excitation is localized on the NMe_2_‐NBD and triazine units, whereas *S*_3_ originates from the Ph‐NBD unit. The band around 320 nm arises from n–π* transitions of the triazine backbone (Figure 7). On the other hand, *S*_2_ is a dark charge transfer (CT) state between the two chromophores potentially prohibiting selective generation of **12a** (*1;0*). Further, switching either side results in a small blueshift of the absorption band of the nonswitched moiety since the size of the central π‐system is decreased upon QC formation. This can also be seen in the experimental spectra (Figure 4). Protonation of the dimethylamine group dramatically reduces its electron donating effect resulting in a significant blueshift. Hence the protonated analogs are more similar to the symmetric derivative **5**.

#### Asymmetric *Tris*‐NBD 8

Lastly, we investigated the photoswitching of the asymmetric *tris*‐NBD **8,** providing the most significant complexity. In this case, three distinct substituted NBD units cause a range of absorption bands, which in principle could be addressed individually. On the one hand, the methoxy functionality in **8** causes an electron donor strength between those of dimethylamine and the parent phenyl substituents.^[^ ^1^, ^2^, ^3^ ^]^ On the other hand, the electron withdrawing strength of the central triazine core is lowered with the removal of every chlorine atom. In principle, a total of 2*^n^* = 8 switching states are possible for **8,** which could be doubled to a total of 16 states by reversible protonation of the amino group.

**Figure 8:**
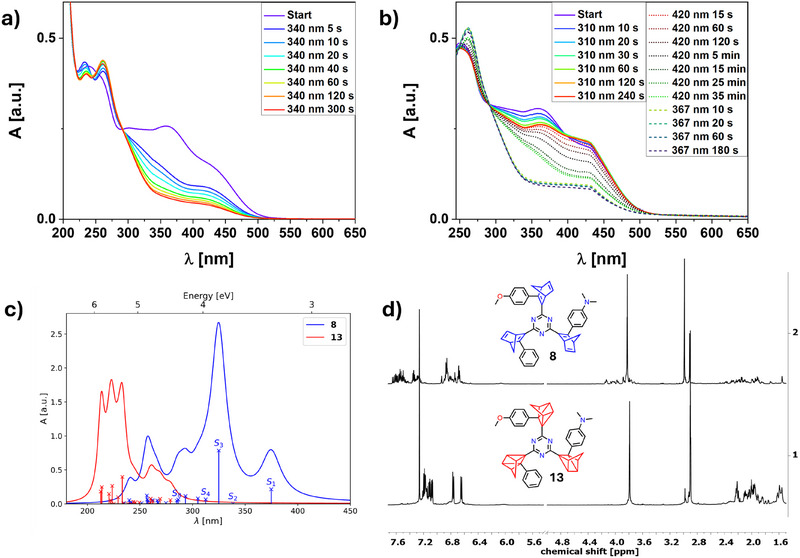
a) UV/vis switching spectra of 8 measured in MeCN using 340 nm to obtain 13. b) Initial irradiation at 310 nm to get single phenyl side switched QC then 420 nm and afterwards 367 nm get complete conversion to 13. c) (TD‐)DFT predicted UV/vis spectra of 8 and 13. d)^ 1^H NMR spectra of 8 and 13 measured in CDCl_3_. QC derivative 13 was obtained after irradiation at 365 nm (80%, 5 min, 15 °C).

UV/vis experiments were conducted to investigate the switching behavior as established. First, complete conversion of **8** to **13** was attempted. Therefore, the wavelength was chosen to irradiate into the λ_max_ absorption at 359 using the 367 nm LED. With this, a decrease in the overall absorption was found, indicating direct conversion to *tris*‐QC species **13**, however, stagnating after a specific time and ultimately resulting in photodecomposition (compare SI). By changing to 340 nm complete conversion of all NBD units of **8** to all QC derivative **13** (QC absorptions at λ_max _= 261 nm) was achieved after 300 s as shown in Figure 8a. Since the most bathochromic shifted absorption was addressed most easily in previous experiments, the same was attempted by irradiation in the onset region using a 475 nm LED to initiate selective single side switching. However, no significant change in the absorption could be found (see Supporting Information). Since the exclusive conversion of the amino‐NBD side seems impossible, the individual conversion of the other substituents was attempted. By irradiation at 310 nm (which partially converted the phenyl substituent in **7**, see above), conversion of either the phenyl or methoxyphenyl substituted NBD was addressed and is assumed to be achieved at least partially. After 240 s, however, stagnation of the absorption change was found. By switching to longer wavelengths (first 420 nm followed by 367 nm), stepwise isomerization resulting in nearly complete conversion to **13** could be accomplished (Figure 8b).

The ^1^H NMR spectra of **8** and **13** are displayed in Figure 8d. Due to the presence of four diastereoisomers, the signals are barely assignable for the individual “arms” of the molecules. In continuous monitoring experiments, the stepwise conversion of the signals belonging to the corresponding NBD‐ or switched QC species cannot be clearly identified since many signals overlap. Nevertheless, the conversion to the only QC bearing species **13** was achieved by irradiation at 365 nm and becomes apparent by the disappearance of the NBD‐bridgehead signals in the region between 4.2 and 3.7 ppm and the downfield shift of the O(CH_3_) and N(CH_3_)_2_ signals from 3.82 to 3.79 ppm and 2.99 to 2.90 ppm, respectively. Furthermore, no significant thermally induced back‐conversion to **8** was found after 2:15 hours at 50 °C. Addition of small amounts of **Por** led to complete recovery of **8**. Full conversion was also proven by ^13^C NMR spectroscopy (see Supporting Information). Partially switched species could not be generated considering NMR experiments.

Quantum chemical calculations help to understand the complex photochemical behavior of **8** upon irradiation. The simulated spectrum of **8** is depicted in Figure 8c. It reveals that the lowest excitation *S*_1_ corresponds to a π–π* transition originating from the dimethylamine substituted NBD. At the same time, *S*_3_ can be understood as a π–π* transition located at the methoxy NBD moiety. However, the methoxy and phenyl moieties are coupled since both contribute to *S*_3_. S_2_ corresponds to a dark CT state transferring electron density from a dimethylamine π orbital to a triazine/methoxy‐NBD π* orbital adding an additional relaxation pathway apart from just energy transfer. Hence, targeting the methoxy band with a 367  or 340 nm LED leads to simultaneous dimethylamine switching mediated by this charge transfer state. Thus, both subsystems switch upon irradiation, resulting in an intensity decrease of their absorption bands in the experiment. Another π–π* charge transfer state is *S*_4_ facilitating energy transfer upon excitation from the methoxy moiety to the other two. Interestingly, *S*_5_–*S*_8_, n–π* transitions originating from the nitrogen lone pairs at the central triazine lie below the Ph‐NBD main absorption band, with S_8_ possessing π contributions from the phenyl substituent. A comparison with the experimental absorption spectrum allows the assignment of the first two bands to *S*_1_ and *S*_3,_ corresponding to the dimethylamine and methoxy moieties, respectively. The plateau around 325 nm originates from the n–π* transitions, similar to the singly substituted system (see Figure 1). Irradiating with the 310 nm LED excites these transitions, which could explain the residual signal (at λ = 430 nm), which does not manifest when only lower energy LEDs are used. The observed blue shift compared to the single switches **4** and **5** might be due to the lack of the electron‐withdrawing chlorine atoms at the triazine core. Thus, selective switching is assumed to be prohibited by competing excited state relaxation pathways, such as energy transfer to lower‐lying excited states, which might be even further facilitated through the low‐lying CT states. Further, substituting every chlorine of the triazine subunit with electron‐donating substituents reduces the electron‐withdrawing effect of the triazine as well as the overall dipole moment of the complete system. This reduces the spectral separation of the employed NBD moieties, favoring charge transfer states and prohibiting selective switching.

### Determination of the Photophysical Key‐Properties

To enable comparison to other NBD‐based multiswitch derivatives, further determination of the photophysical properties was conducted. Therefore, key‐properties essential for actual application development, as photoisomerization quantum yields *Φ*_iso_ and half‐lives *t*_1/2_, were investigated, as well as preliminary cyclization experiments testing thermally induced back‐conversion. However, due to the complexity of the investigated system relying on multistep isomerization processes, evaluation of the obtained data was only partially successful. A summary of the results is provided in Table 3.

**Table 3:** Overview of the obtained photophysical key‐properties. The two observable single‐conversion processes (**4** to **9** and **7** to **12b** and vice versa) could be thoroughly analyzed.

Entry	*t*_1/2_ (25 °C) [min]	Φ_iso_ ^b)^ [%]
**4** to **9**	158	76.4
**5** to **10**	3912 days^a)^	/
**7** to **12b**	301	*1.83*
**12b** to **12**	/	/
**7** to **12**	/	/
**8** to **13**	/	/

^a)^ This value must be seen as an estimation since fitting with a first‐order kinetic model does not fully represent the ongoing multi‐step process

^b)^ values measured in MeCN using an automated setup (compare Supporting Information).

We refer to sections 7 and 8 of the Supporting Information for a detailed analysis and listing of the data. First, simple cyclization studies were conducted to check the thermal reversibility of the NBD to QC isomerization. For each derivative, a sample was prepared in C_2_D_2_Cl_4_ and irradiated at the respective wavelength to trigger the conversion. Subsequently, the same sample was heated to 130 °C, and after each step, a ^1^H NMR spectrum was recorded. This process was repeated five times or until complete decomposition occurred. In all cases, the observed degree of decomposition was larger during the heating process than during the illumination. Since no additional NMR standard was utilized in the experiments, quantifying the decomposition was impossible. However, the decomposition process is facilitated with the increasing complexity of the system. While for the phenyl substituted derivatives **9** and **10**, only negligible and minor decomposition was found, respectively, all derivatives containing an amine substituent (**12b**, **12**, and **13**) significantly degraded during the cycles. For **13**, complete decomposition was found after only two cycles.

Second, to determine the stability of the systems, thermal half‐lives were measured. The temperatures investigated for each derivative were based on the findings obtained during the cyclization experiments. For each derivative, the thermally induced back‐conversion from QC to NBD was triggered at different temperatures monitored via ^1^H NMR spectroscopy. The respective *t*_1/2_ values at 25 °C were calculated by fitting and extrapolation of the Eyring plot. In addition, the Δ*H* ^╪^_thermal_ and Δ*S* ^╪^_thermal_ values were determined (compare Supporting Information). For the single conversions **9** to **4** and **12b** to **7**, half‐lives of 158 and 301 min were obtained, respectively. For the back‐isomerization of **10** to **5**, including the conversion of two QC to two NBD moieties, the utilization of a unimolecular first‐order kinetic model is not entirely applicable. Only the overall isomerization from *bis*‐QC 10 to *bis*‐NBD 5 species was considered, abandoning intermediate NBD‐QC mixed species. An impressively high *t*_1/2_ value of 3912 days was calculated, which must be viewed as an approximation, considering the simplified kinetic fitting model. However, compared with the literature, a similar methodology was applied, neglecting the intermediate formed species.^[^ ^15^ ^]^ For the multiprocess conversions **12** to **7** and **13** to **8**, no half‐lives could be measured. For 1**2** to **7**, not further determined intermediate species formed at temperatures of 60 °C and 100 °C, ultimately resulting in degradation at 130 °C. Therefore, no quantitative investigation of the present NBD/QC ratio was performed. Thus, the corresponding calculation of the kinetic features was impossible. Considering **13** to **8**, the same findings were made.

Lastly, the determination of the photoisomerization quantum yield (*Φ*_iso_) was attempted. Here, a technique based on an automized reaction setup was utilized^[^ ^39^ ^]^ since our setup has been shown to be unsuitable for conventional chemical actinometry in recent studies.^[^ ^3^ ^]^ Again, only for the single conversions, satisfactory results could be obtained. For **4** to **9**, a quantum yield of 76.4% was obtained, and for the conversion of **7** to **12b**, a value of 1.83%. For the more complex systems incorporating multiple conversion processes, either no or values larger than 100% were found as for **5** to **10** (compare Supporting Information). The overall process of **7** to **12** and **8** to **13** was investigated, however, unsuccessful. Relying on deconvolution of the recorded UV/vis spectra, conversions with multiple processes can only be calculated with clean extinction spectra of all intermediate derivatives. Since generation and comprehensive characterization of the required intermediates **12a**, and the singly switched mixed NBD/QC species of **8** was not possible, evaluation of the generated data for the overall conversion processes failed. Lastly, the single process of **12b** to **12** was investigated, in which an unknown conversion was found, resulting in a failure of the determination of any *Φ*_iso_ value. For a detailed description and analysis of the experiments, we refer to the Supporting Information.

## Conclusion

We have synthesized and characterized simple norbornadiene‐triazine architectures including multistate photoswitches with unprecedentedly high information storage densities. Our general reaction scheme allows straightforward access to both symmetric and asymmetric triazene‐acetylene precursors, which were subsequently transformed into the respective target compounds. Spectroscopic investigations of the lesser complex phenyl and diphenyl derivatives **4** and **5** provided valuable insight into the absorption characteristics of this new compound class. With the loss of each chlorine atom attached in the central triazine unit, the electron‐withdrawing ability decreased, resulting in a hypochromic shift of the λ_max_ value from 363 to 357 nm, moving from **4** to **5**. A strong solvent dependency of the interconversion behavior was found. By adding the catalyst **Por**, complete regain of the NBD species was possible, while in the case of **4**/**9**, thermally induced back‐conversion was found at ambient conditions. The amino derivative **6** was prepared to investigate a stronger electron‐donating group. Here, no conversion to the respective QC **11** could be accomplished, while more likely polymerization or other noninvestigated photodegradation occurred. By a combination of different substituents, the two asymmetric derivatives **7** and **8** were prepared and investigated. In the case of the *tris*‐NBD **8**, selective switching of the individual NBD chromophores is hampered by the closely related optical properties of all three NBD units. On the other hand, the asymmetric photoswitch system **7/12**, containing two distinct NBD‐substituents, fulfilled the requirements of a selectively addressable multistate system with an extremely high information storage density. Here, all possible NBD/QC combinations **7**, **12a**, **12b**, and **12** could be realized. Reversible protonation of the NMe_2_ group was targeted to double the number of available states. Thereby, a total of at least six different states were accessible by using different input sequences. Furthermore, an acid‐catalyzed back‐reaction of the QC to NBD species was found. Photophysical key‐properties, such as half‐lives and photoisomerization quantum yields, were determined. For all QC derivatives, clean catalytic‐induced back‐isomerization was possible using **Por**, while thermal reconversion was accompanied by increasing decomposition with increasing complexity of the molecules. Our experimental findings were corroborated by quantum chemical calculations. Although the actual development of an application at this point has not yet been investigated and still provides significant challenges, such as the readout process, this work lays the foundation for a new promising class of multi‐NBD‐based systems. For future experiments unraveling the fundamental mechanistics enabling the accessibility of even more states will pave the way towards actual applications such as data storage systems or molecular‐based logical devices.

## Supporting Information

The authors have cited additional references within the Supporting Information.^[^ ^40^, ^41^, ^42^, ^43^, ^44^, ^45^, ^46^, ^47^, ^48^, ^49^, ^50^, ^51^ ^]^

## Conflict of Interests

The authors declare no conflict of interest.

## Supporting information



Supporting Information

## Data Availability

The data that support the findings of this study are available from the corresponding author upon reasonable request.
