# Supplementary material for: Low Molecular Weight Multistate Photoswitches Based on Simple Norbornadiene‐Triazine Scaffolds
Source: Angew Chem Int Ed Engl. 2025 Jul 16;64(35):e202507999. doi: 10.1002/anie.202507999 (PMC12377450; doi:10.1002/anie.202507999)
Supplement: Supplementary file 1 — Supporting Information [file ANIE-64-e202507999-s001.pdf]

Table of Contents

1 Materials and Methods 4

2 Synthesis and Characterization 8

2.1 Phenyl-mono-NBD hybrid 10

2.1.1 1a: *2,4-dichloro-6-(phenylethynyl)-1,3,5-triazine* 10

2.1.2 4: *2,4-dichloro-6-(3-phenylbicyclo[2.2.1]hepta-2,5-dien-2-yl)-1,3,5-triazine* 11

2.1.3 9: *1PhQC: 2,4-dichloro-6-(5-phenyltetracyclo[3.2.0.0^{2,7}.0^{4,6}]heptan-1-yl)-1,3,5-triazine* 11

2.2 Diphenyl-bis-NBD hybrid 12

2.2.1 2a: *2-chloro-4,6-bis(phenylethynyl)-1,3,5-triazine* 12

2.2.2 5: *2-chloro-4,6-bis(3-phenylbicyclo[2.2.1]hepta-2,5-dien-2-yl)-1,3,5-triazine* 13

2.2.3 10: *2-chloro-4,6-bis(5-phenyltetracyclo[3.2.0.0^{2,7}.0^{4,6}]heptan-1-yl)-1,3,5-triazine* 14

2.3 Dimethylaniline-mono-NBD hybrid 15

2.3.1 1c: *4-((4,6-dichloro-1,3,5-triazin-2-yl)ethynyl)-N,N-dimethylaniline* 15

2.3.2 6: *4-(3-(4,6-dichloro-1,3,5-triazin-2-yl)bicyclo[2.2.1]hepta-2,5-dien-2-yl)-N,N-dimethylaniline* 16

2.3.3 11: *4-(5-(4,6-dichloro-1,3,5-triazin-2-yl)tetracyclo[3.2.0.0^{2,7}.0^{4,6}]heptan-1-yl)-N,N-dimethylaniline* 17

2.4 Dimethylaniline-phenyl-bis-NBD hybrid 17

2.4.1 2b: *1Ph1NMeAc: 4-((4-chloro-6-(phenylethynyl)-1,3,5-triazin-2-yl)ethynyl)-N,N-dimethylaniline* 17

2.4.2 7: *4-(3-(4-chloro-6-(3-phenylbicyclo[2.2.1]hepta-2,5-dien-2-yl)-1,3,5-triazin-2-yl)bicyclo[2.2.1]hepta-2,5-dien-2-yl)-N,N-dimethylaniline* 18

2.4.3 12: *4-(5-(4-chloro-6-(5-phenyltetracyclo[3.2.0.0^{2,7}.0^{4,6}]heptan-1-yl)-1,3,5-triazin-2-yl)tetracyclo[3.2.0.0^{2,7}.0^{4,6}]heptan-1-yl)-N,N-dimethylaniline* 20

2.4.4 12b: *4-(3-(4-chloro-6-(5-phenyltetracyclo[3.2.0.0^{2,7}.0^{4,6}]heptan-1-yl)-1,3,5-triazin-2-yl)bicyclo[2.2.1]hepta-2,5-dien-2-yl)-N,N-dimethylaniline* 20

2.4.5 12a: *4-(3-(4-chloro-6-(5-phenyltetracyclo[3.2.0.0^{2,7}.0^{4,6}]heptan-1-yl)-1,3,5-triazin-2-yl)bicyclo[2.2.1]hepta-2,5-dien-2-yl)-N,N-dimethylaniline* 22

2.5 Asymmetric dimethylaniline-anisyl-phenyl-tris-NBD hybrid 22

2.5.1 3: *4-((4-((4-methoxyphenyl)ethynyl)-6-(phenylethynyl)-1,3,5-triazin-2-yl)ethynyl)-N,N-dimethylaniline* 22

2.5.2 8: *4-(3-(4-(3-(4-methoxyphenyl)bicyclo[2.2.1]hepta-2,5-dien-2-yl)-6-(3-phenylbicyclo[2.2.1]hepta-2,5-dien-2-yl)-1,3,5-triazin-2-yl)bicyclo[2.2.1]hepta-2,5-dien-2-yl)-N,N-dimethylaniline* 23

2.5.3 13: *4-(5-(4-(5-(4-methoxyphenyl)tetracyclo[3.2.0.0^{2,7}.0^{4,6}]heptan-1-yl)-6-(5-phenyltetracyclo[3.2.0.0^{2,7}.0^{4,6}]heptan-1-yl)-1,3,5-triazin-2-yl)tetracyclo[3.2.0.0^{2,7}.0^{4,6}]heptan-1-yl)-N,N-dimethylaniline* 25
2.6 Additional acetylene derivatives 26
2.6.1 1b: *2,4-dichloro-6-((4-methoxyphenyl)ethynyl)-1,3,5-triazine* 26
2.6.2 2c: *4-((4-chloro-6-((4-methoxyphenyl)ethynyl)-1,3,5-triazin-2-yl)ethynyl)-N,N-dimethylaniline* 27
2.6.3 Por [5-(p-Carboxyphenyl)-10,15,20-(p-tert-butyltriphenylphenyl)porphyrinato] cobalt (II) (carboxylic acid A_3B Cobalt (II) -Porphyrin) 28
3 Characterization of the Synthesized Molecules 29
3.1 Phenyl-mono-NBD hybrid 29
3.1.1 1a 29
3.1.2 4 32
3.1.3 9 36
3.2 Diphenyl-bis-NBD hybrid 38
3.2.1 2a 38
3.2.2 5 41
3.2.3 10 44
3.3 Dimethylaniline-mono-NBD hybrid 46
3.3.1 1c 46
3.3.2 6 49
3.3.3 11 52
3.4 Dimethylaniline-phenyl-bis-NBD hybrid 53
3.4.1 2b 53
3.4.2 7 56
3.4.3 12b 67
3.4.4 12 69
3.5 Asymmetric dimethylaniline-anisyl-phenyl-tris-NBD hybrid 73
3.5.1 3 73
3.5.2 8 75
3.5.3 13 84
3.6 Additional acetylene derivatives 91
3.6.1 1b 91
3.6.2 2c 96
4 Switching studies monitored via NMR spectroscopy 98

4.1 Phenyl-mono NBD hybdrid: NBD 4 to QC 9 99
4.2 Diphenyl-bis NBD hybrid: NBD 5 to QC 10 102
4.3 *N,N*-dimethylaniline-mono NBD hybrid: NBD 6 to QC 11 106
4.4 *N,N*-dimethylaniline-phenyl-bis-NBD hybrid: NBD 7 to QC 12 (and intermediate species) 109
4.4.1 NBD to QC interconversion experiments monitored via NMR 109
4.4.2 Reversible protonation experiments monitored via NMR 117
4.5 *N,N*-dimethylaniline-anisyl-phenyl-tris-NBD hybrid: NBD 8 to QC 13 124
5 Switching studies monitored via UV/Vis spectroscopy 127
5.1 NBD 4 to QC 9 127
5.2 NBD 5 to QC 10 128
5.3 NBD 6 to QC 11 130
5.4 NBD 7 to QC 12 (and single side switched intermediate species 12a and 12b) 132
5.4.1 Switching experiments of the normal states 132
5.4.2 Reversible protonation experiments towards 12c-12f 136
5.5 NBD 8 to QC 13 146
6 Photoisomerization Quantum Yields (ϕ_{iso}) 150
6.1 4 → 9 151
6.2 5 → 10 152
6.3 7 → 12b 153
6.4 12b → 12 154
6.5 7 → 12 155
6.6 8 → 13 156
7 Thermal half-lives ($t_{1/2}$) and cyclability of the QC derivatives 158
7.1 9 → 4 160
7.2 10 → 5 162
7.3 12 → 7 165
7.4 12b → 7 168
7.5 13 → 8 170
8 Computational Methods 171
8.1 (TD-)DFT predicted spectra 171
8.2 Geometries 172
8.3 Excited states 195
8.4 Attachment and detachment densities 204
9 References 207

1 Materials and Methods

All reactions involving moisture or oxygen sensitive compounds were carried out under an inert gas atmosphere of nitrogen or argon, using anhydrous solvents and standard Schlenk techniques. All used solvents were distilled on a rotary evaporator before usage. All required chemicals were purchased from commercial suppliers Sigma-Aldrich (St. Louis, MO, USA), ABCR (Karlsruhe, Germany), VWR (Darmstadt, Germany), Acros Organics (Geel, Belgium), or Roth (Karlsruhe, Germany) and used directly without further purification. TLC analysis was performed on aluminum plates coated with 0.20 mm Merck® silica gel 60 containing a fluorescent indicator (Machery-Nagel, ALUGRAM®, SILG/UV_{254}). Spots on TLC plates were visualized by exposure to ultraviolet light (λ = 254 nm and 366 nm). Purification via column chromatography was performed on silica gel 60M deactivated (0.04-0.063 mm / 230-400 mesh ASTM) from Macherey-Nagel®, Düren, Germany.

Flash column chromatography: Flash column chromatography was performed with a Biotage® Selekt apperatus, with the software SELEKT 1.1.1.-13044 using pre-packed columns purchased from Büchi (St. Gallen, Switzerland).

NMR: All NMR spectra were recorded on a BRUKER Avance gX (^{1}H NMR: 300 MHz, 400 MHz or 600 MHz; ^{13}C NMR: 75 MHz, 100 MHz or 151 MHz). All measurements were performed at room temperature if not otherwise stated. Chemical shifts δ are reported in ppm and were referenced to the residual solvent signal as an internal reference (*Table S1*). Solvent impurities were determined according to the work of *Fulmer et. al*.

Table S 1: List of deuterated solvents used for ^{1}H and ^{13}C NMR spectroscopy. The respective residual solvent signal was calibrated on the following values.[1]

**Solvent**	**δ_H [ppm]**	**δ_C [ppm]**
$CDCl_3$	7.26	77.16
Benzene-*d_6*	7.16	128.06
$C_2D_2Cl_4$	5.91	/
CD_2Cl_2	5.32	53.84
Toluene-*d_8*	2.08	20.43
Acetonitrile-*d_3*	1.94	118.26

Progressing of the raw data was done with MestReNova. During the characterization of the signal multiplicities the following abbreviations were used: s = singlet, d = doublet, ddd = doublet of a doublet of a doublet, t = triplet, q = quartet, m = multiplet. ^{13}C NMR spectra were recorded by broadband decoupling.

Mass Spectrometry: MS was performed on a BRUKER DALTONICS MaXis4g (ESI & APPI). As ionization method either ESI or atmospheric pressure photoionization (APPI) was used. For detection of the formed ions Time of Flight (TOF) was performed. The mass-to-charge ratios *m/z* are given in u.

GC-MS: Gas chromatographic coupled mass spectrometric measurements were performed with a GC-2010 Plus gas chromatography system (Shimadzu) with helium as carrier gas and detection by a QP2010 SE single quadrupole mass spectrometer (Shimadzu). As an ionization method electron ionization (EI) was used. A phenomenex™ Zebron-ZB5*Plus* GC column with the dimensions 30 m x 0.25 mm and a film thickness of 0.25 µm was used. Zebron 3.4 mm straight Z liners were used in split injection mode.

HPLC: High-performance liquid chromatography was performed with an LC20-AT Prominence (Shimadzu) on a normal-phase EC MN-Nucleosil 100-5 column with dimensions of 250 mm x 4 mm (Macherey-Nagel).

UV/Vis spectroscopy: UV-Vis spectroscopy was carried out on a Varian Carry 5000 UV/Vis-NIR spectrometer. The baseline and zero transmittance were calibrated before measurements were taken. A 10 x 10 mm high precision cell from Hellma®Analytics, Müllheim, Germany was used. All measurements were conducted using HPLC grade as solvents. λ_{onset} values are determined as $\log(\varepsilon) = 2$. All provided values for ε are given in M^{-1} cm^{-1}.

Infrared spectroscopy: IR measurements were performed on a PerkinElmer Frontier FT-IR spectrometer equipped with a PerkinElmer "Universal ATR Sampling Accessory" unit.

Microwave reactor:

Reactions including heating with microwave irradiation were performed in an Anton Paar Monowave 450 using Anton Paar G4, G10, or G30 microwave vials (30 bar max. pressure). The reaction mixtures were heated as fast as possible to the desired temperature with a maximum power of 850 W and subsequently cooled to 55 °C using pressurized air before the described workup procedure.

Irradiation setup:

For all switching experiments, an in-house-built irradiation setup was used (Figure *S1a*).[2] UV/Vis monitoring experiments were exclusively performed using this apparatus. All utilized LEDs were purchased from Neumüller Elektronik GmbH® or Conrad Electronics SE. The power of the used LEDs with respect to their emission wavelength is given for the maximum current in Table *S1*. The samples were irradiated directly in the NMR tube or a Quartz glass cuvette for NMR and UV/Vis switching studies, respectively. The distance between the sample and the LED was kept at 1.5 cm. For irradiation experiments, the maximum power of every LED was tied to use (specifications are provided in *table S2*).

All measurements, including a specific % value, were measured in a Lucent360™ Advanced Photoreactor by HepatoChem (*figure S1b*) with irradiation directly in the NMR tube. Wavelengths of the available LED blocks are 310 nm, 365 nm, and 425 nm, each with a power of approximately 50 W at 100% performance. The detailed technical data can be obtained on the supplier's website. All measured samples were cooled to 15 °C if not stated otherwise. External cooling was done by a Huber KISS K6 thermostat unit connected to the irradiation apparatus.

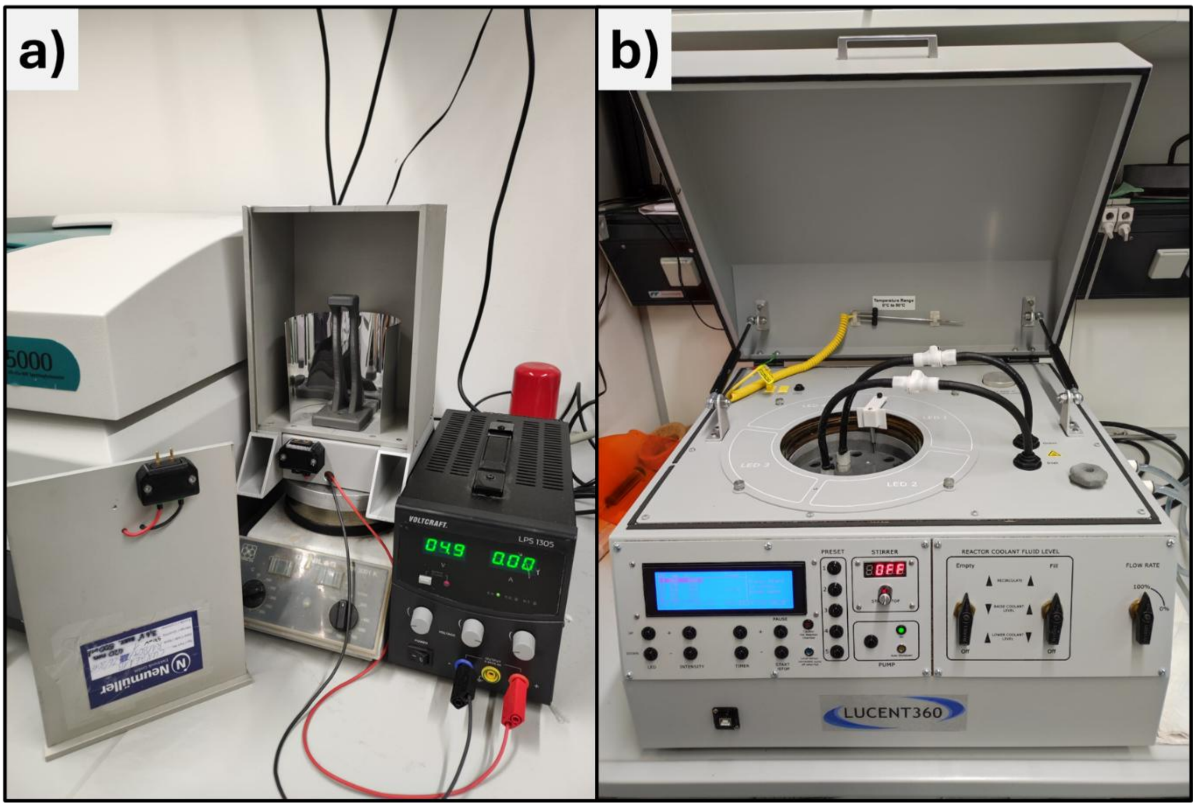

Figure S 1: a) Home-made irradiation apparatus. b) Lucent360TM Advance photoreactor purchased from HepatoChem.

Table S 2: LEDs used in the home-made irradiation setup.

Wavelength [nm]	Article Number (Supplier)	Max. Power and characteristics	Measured current during the experiment
275	CUD8AF4D (Neumüller Elektronik GmbH)	60 mW at 600 mA / 6.4V	6.9-7.2 V with 0.31-0.43 A
310	CUD1AF4D (Neumüller Elektronik GmbH)	30 mW at 600 mA / 5.5V	5.2-5.5 V with 0.34-0.50 A
340	CUD4GF1B (Neumüller Elektronik GmbH)	120 mW at 1200 mA (4.4V)	3.1-3.6 V with 0.26 – 0.45 A
367	CUN66A1B (Neumüller Elektronik GmbH)	1000 mW at 500 mA / 3.8V	3.5-3.7 V with 0.26-0.48 A
400	CUN0CF1 (Neumüller Elektronik GmbH)	61 mW at 50 mA / 3.2V	3.0 V with 0.04 A
420	CUN26A1B (Neumüller Elektronik GmbH)	970 mW at 500 mA 3.6V	3.5V with 0.35 A
475	Roschwege Star-BL475-03-00-00 (Conrad Electronic SE)	3000 mW at 350 mA / 3.2 V	3.0-3.2 V with 0.16-0.32 A
525	NMOP-10002B SMD LED (Mid-Power) (Neumüller Elektronik GmbH)	Power not specified<br>60 mA / 3.1 V	3.1 V with 50 mA

2 Synthesis and Characterization

Preparation of the GRIGNARD reagents:

The alkynylmagnesium bromides were prepared according to a procedure adapted from literature.[3] In a flame dried, argon containing apparatus with dropping funnel and dimroth condenser the respective phenyl acetylene (1.0 Eq) was dissolved in anhydrous THF. The reaction was cooled to 0 °C and a EtMgBr solution (3M in Et_2O, 1.0 Eq) added dropwise over 45 minutes. The resulting solvent ratio should be anhydrous THF/Et_2O 1:2 (v/v). After complete addition, the mixture was further stirred for 15 min at 0 °C. The reaction was slowly warmed to rt and then heated to 50 °C for 2 h. Afterwards, the reaction was cooled to rt and the obtained Grignard reagent used for the next step without additional purification.

General procedure for NUCLEOPHILIC SUBSTITUTION reactions:

The nucleophilic substitution was performed based on a procedure adapted from literature.[4] Cyanuric chloride (1.0 Eq) was put in a flame dried argon containing flask and dissolved in a 1:2 mixture of anhydrous THF and Et_2O to get a 0.1 M solution which was cooled to 0 °C. The previously prepared alkynylmagnesium bromide (1.5 Eq for single substitution, 4.5 Eq for two-fold substitution) diluted to get a 0.1 M solution (THF/Et_2O, 1:2, v/v) was added dropwise at 0 °C. After complete addition, the reaction was warmed to rt and stirred for 40 to 120 h at this temperature. Thereby the reaction was monitored using TLC and GC-MS analysis. After complete reaction, the reaction was quenched through addition of a sat. NH_4Cl solution and the mixture stirred for 15 minutes. The aqueous phase was extracted with EtOAc (3 x 50 mL) and the combined organic phases washed with brine (2 x 100 mL). The organic phases were dried over $MgSO_4$, filtered and the solvent removed under reduced pressure. Purification was done *via* automized flash column chromatography to obtain the cyanuric acetylene as colorless to red solid.

General procedure for the preparation of zinc acetylenes:

The zinc-acetylene species necessary for the subsequent Negishi couplings were prepared according to a procedure adapted from literature.[5] In a flame dried, argon containing apparatus phenyl acetylene (1.0 Eq) was dissolved in anhydrous THF (5 mL) and cooled to -78 °C. *n*BuLi (2.5 M in hexane, 1.2 Eq) was added dropwise and the resulting mixture stirred at -78 °C for 30 minutes. Afterwards, $ZnCl_2$ (1.2 Eq) was added in one portion and the reaction further stirred at -78 °C for 1 h. The colling bath was removed, the mixture warmed to rt and stirred at this temperature for 1.5 h before the obtained zinc acetylene was used for the next step without additional purification.

General procedure for NEGISHI cross-coupling reactions:

The NEGISHI cross-coupling reaction was carried out according to a procedure adapted from literature procedures.[5],[6] Cyanic chloride or a respective cyanuric acetylene (1.0 Eq) and $Pd(PPh_3)_4$ (5.0 mol%) were put in a flame dried argon containing pressure flask and dissolved in anhydrous NMP. The previously prepared zinc acetylene (4.0 Eq) was transferred to the reaction vessel, and the mixture heated to 50 °C for 20 h while the reaction was monitored using TLC and GC-MS analysis. After complete conversion, the reaction was quenched through addition of H_2O. The aqueous phase was extracted with EtOAc (3 x 30 mL) and the combined organic phases washed with brine (2 x 75 mL).

The organic phases were dried over $MgSO_4$, filtered and the solvent removed under reduced pressure. Purification was done *via* automized flash column chromatography to obtain the cyanuric acetylene.

General procedure for DIELS-ALDER reactions:

The respective cyanuric acetylene (1.0 Eq) and freshly cracked cyclopentadiene (CP) (15 Eq) were combined in a microwave pressure tube and diluted with toluene to reach the maximum fill level of the tube. The reaction vessel was sealed and heated to 120-180 °C (depending on the substituent; Ph < OMe < NMe_2) for 2-38 h using microwave irradiation. The reaction process was monitored using GCMS analysis. In case of slow or uncomplete conversion, additional CP was added and the reaction temperature slightly raised (10 °C steps). After complete conversion, the solvent and excessive cyclopentadiene were removed under reduced pressure. Purification was achieved *via* automized flash column chromatography (SiO_2, EtOAc in hexane) yielding the respective NBD.

General switching procedure for NMR experiments:

Photoisomerization towards molecule QC was achieved using a home-made irradiation apparatus or the Lucent360 photoreactor and verified *via* ^{1}H NMR spectroscopy.[7] The respective NBD (7.5 mg) was dissolved in 650 µl (750 µl in case of MeCN) of the respective deuterated solvent and transferred into a NMR test tube. One initial NMR was measured of each freshly prepared sample prior to the irradiation studies. The NMR test tube was irradiated using the given LED lamp for a certain period of time. For irradiation done with the photoreactor, besides wavelength, the used power and temperature is given as well. After each irradiation step, an additional ^{1}H NMR spectrum was measured to verify the conversion. This procedure was carried on until full conversion, no further conversion or photodecomposition could be observed.

General switching procedure for UV/Vis experiments:

Photoisomerization towards molecule QC was achieved using a home-made irradiation apparatus exclusively.[7] The respective NBD was dissolved in MeCN or methylene chloride to obtain an average concentration of 4.35×10^{-5} in the range of 1.83×10^{-5} to 9.49×10^{-5}. The prepared sample was transferred into a quartz glass cuvette and irradiated using the given LED lamp for the stated period of time. UV/Vis spectra were recorded prior irradiation and after each irradiation step. This procedure was carried on until full conversion, no further conversion or photodecomposition could be observed.

2.1 Phenyl-mono-NBD hybrid

2.1.1 **1a:** *2,4-dichloro-6-(phenylethynyl)-1,3,5-triazine*

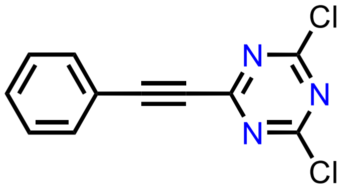

Chemical Formula: $C_{11}H_5Cl_2N_3$
Molecular Weight: 250.08

The alkynylmagnesium bromide was prepared according to a procedure adapted from literature.[3] In a flame dried, argon containing apparatus with dropping funnel and dimroth condenser phenyl acetylene (3.57 mL, 32.5 mmol, 1.0 Eq) was dissolved in anhydrous THF (5.4 mL). The reaction was cooled to 0 °C and a EtMgBr solution (3M in Et_2O, 10.8 mL, 32.5 mmol, 1.0 Eq) added dropwise over 45 minutes. After complete addition, the mixture was further stirred for 15 min at 0 °C. The reaction was slowly warmed to rt and then heated to 50 °C for 2 h. Afterwards, the reaction was cooled to rt and the obtained Grignard reagent used for the next step without additional purification. The nucleophilic substitution was performed based on a procedure adapted from literature.[4] Cyanuric chloride (1.00 g, 5.42 mmol, 1.0 Eq) was put in a flame dried argon containing flask and dissolved in a 1:2 mixture of anhydrous THF and Et_2O (18.1 mL and 36.2 ml, respectively) to get a 0.1 M solution which was cooled to 0 °C. The previously prepared phenylmagnesium bromide (8.13 mmol, 1.5 Eq) was thinned to get 0.1 M solution (THF/Et_2O, 1:2, v/v) and added dropwise at 0 °C over 1h. After complete addition, the reaction was warmed to rt and stirred for 44 h at this temperature. Thereby the reaction was monitored using TLC and GC-MS analysis. After complete reaction, the reaction was quenched through addition of a sat. NH_4Cl solution (100 mL) and the mixture stirred for 15 minutes. The aqueous phase was extracted with EtOAc (3 x 50 mL) and the combined organic phases washed with brine (2 x 100 mL). The organic phases were dried over $MgSO_4$, filtered and the solvent removed under reduced pressure. Purification was done *via* column chromatography eluted with a mixture of EtOAc in hexane (1:10, v/v) to obtain the cyanuric acetylene **1a** as pale-yellow solid.

Yield: 476 mg, 1.90 mmol, 35 %.

R_f = 0.49 (hexanes/EtOAc 10:1)

^{1}H NMR (400 MHz, $CDCl_3$, 25 °C) δ_H [ppm]: 7.73 – 7.68 (m, 2H), 7.55 – 7.50 (m, 1H), 7.47 – 7.41 (m, 2H).

^{13}C NMR (101 MHz, $CDCl_3$, 25 °C): (δ) [ppm] = 172.0, 162.3, 133.7, 131.8, 129.0, 119.5, 97.7, 85.9.

UV/Vis: λ_{max} (ε) [nm] = 229 (12100), 315 (23700)

HRMS (APPI): calc. for $(C_{11}H_6Cl_2N_3)^+$: 249.9933, 251.9907 ; found *m/z* = 249.9936, 251.9907 $[M+H]^+$.

2.1.2 **4:** *2,4-dichloro-6-(3-phenylbicyclo[2.2.1]hepta-2,5-dien-2-yl)-1,3,5-triazine*

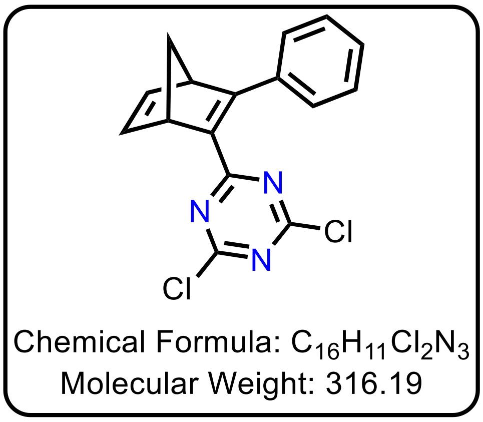

The respective cyanuric acetylene **1a** (100 mg, 0.400 mmol, 1.0 Eq) and freshly cracked cyclopentadiene (CP) (496 µL, 6.00 mmol, 15 Eq) were combined in a microwave pressure tube and diluted with 1.5 ml toluene. The reaction vessel was sealed and heated to 110 °C for 4 h using microwave irradiation. Afterwards, another 5.0 equivalents of CP (165 µL, 2.00 mmol, 5.0 Eq.) were added and the reaction temperature increased to 120 °C for 2h. Thereby the reaction was monitored using GCMS analysis. After complete conversion, the solvent and excessive cyclopentadiene were removed under reduced pressure. Purification was achieved *via* automized flash column chromatography (SiO_2, 5 % EtOAc in hexanes) and subsequent recrystallization from DCM/heptane yielding the product as slightly yellowish solid.

Yield: 57.5 mg, 0.182 mmol, 45 %.

$\mathbf{R_f}$ = 0.64 (hexanes/EtOAc 10:1)

^{1}H NMR (400 MHz, $CDCl_3$, 25 °C) δ_H [ppm]: 7.60–7.56 (m, 2H), 7.43–7.37 (m, 3H), 7.10–7.07 (m, 1H), 6.96–6.93 (m, 1H), 4.48–4.45 (m, 1H), 4.01–3.97 (m, 1H), 2.36–2.32 (m, 1H), 2.20–2.16 (m, 1H).

^{13}C NMR (101 MHz, $CDCl_3$, 25 °C): (δ) [ppm] = 173.7, 172.5, 171.0, 143.8, 143.3, 140.9, 135.7, 129.6, 128.4, 127.7, 69.6, 60.6, 53.1.

IR (ATR, cm^{-1}): $\tilde{v}$ = 3067 (w), 3011 (m), 2988 (m), 2948 (m), 2876 (w), 1505 (s), 1467 (s), 1399 (m), 1282 (m), 1263 (m), 1237 (s), 846 (s), 811 (s), 800 (m), 767 (vs), 751 (m), 717 (s) cm^{-1}

UV/Vis: λ_{max} (ε) [nm] = 235 (15300), 284 (5500), 363 (10900); λ_{onset} [nm] = 446 nm

HRMS (APPI): calc. for $(C_{16}H_{12}Cl_2N_3)^+$: 316.0403; found *m/z* = 316.0391[M+H]$^+$.

2.1.3 **9:** *1PhQC: 2,4-dichloro-6-(5-phenyltetracyclo[3.2.0.0^{2,7}.0^{4,6}]heptan-1-yl)-1,3,5-triazine*

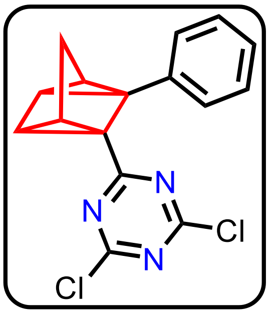

Switching of **4** was conducted according to general procedure D. The NBD was dissolved in $CDCl_3$ and irradiated at 367 nm (3.6 V, 0.37 A). Quantitative conversion could be achieved after 20 min in NMR scale. The same results were found in MeCN-d_3 accompanied by massive precipitation and additional photodegradation. In UV/Vis scale (general procedure E), full

isomerization to **9** was obtained after 3 seconds in $CHCl_3$ while photodecomposition could be observed in MeCN.

Yield: Quantitative (367 nm (3.6 V, 0.37 A) MeCN-d_3, 20 minutes)

Quantitative (367 nm (3.6 V, 0.37 A) $CDCl_3$, 20 minutes)

^{1}H NMR (400 MHz, $CDCl_3$, 25 °C) δ_H [ppm]: 7.31 – 7.26 (m, 4H), 7.26 – 7.21 (m, 1H), 3.03 – 2.97 (m, 2H), 2.55 – 2.51 (m, 1H), 2.41 (dd, J_1 = 4.9, J_2 = 2.4 Hz, 1H), 2.34 – 2.29 (m, 1H), 1.92 – 1.89 (m, 1H).

^{13}C NMR (MHz, $CDCl_3$, 25 °C): (δ) [ppm] = 182.7, 170.1, 136.1, 129.6, 127.7, 126.6, 44.5, 39.5, 38.7, 33.7, 32.7, 32.5, 21.7.

UV/Vis: λ_{max} (ε) [nm] = 309 (8000) ($CDCl_3$)

2.2 Diphenyl-bis-NBD hybrid

2.2.1 **2a:** *2-chloro-4,6-bis(phenylethynyl)-1,3,5-triazine*

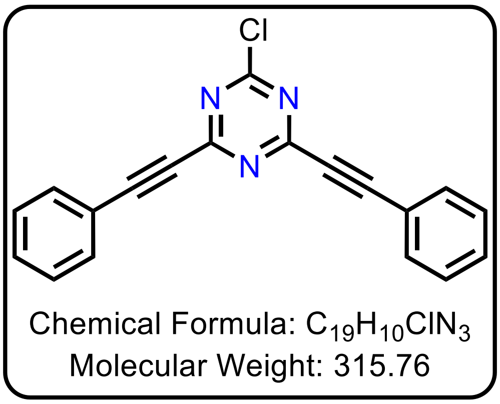

The alkynylmagnesium bromide was prepared according to a procedure adapted from literature.[3] In a flame dried, argon containing apparatus with dropping funnel and dimroth condenser phenyl acetylene (3.57 mL, 32.5 mmol, 1.0 Eq) was dissolved in anhydrous THF (5.4 mL). The reaction was cooled to 0 °C and a EtMgBr solution (3M in Et_2O, 10.8 mL, 32.5 mmol, 1.0 Eq) added dropwise over 45 minutes. After complete addition, the mixture was further stirred for 15 min at 0 °C. The reaction was slowly warmed to rt and then heated to 50 °C for 2 h. Afterwards, the reaction was cooled to rt and the obtained Grignard reagent used for the next step without additional purification. The nucleophilic substitution was performed based on a procedure adapted from literature.[4] Cyanuric chloride (1.00 g, 5.42 mmol, 1.0 Eq) was put in a flame dried argon containing flask and dissolved in a 1:2 mixture of anhydrous THF and Et_2O (18.1 mL and 36.2 ml, respectively) to get a 0.1 M solution and cooled to 0 °C. The previously prepared alkynylmagnesium bromide (24.4 mmol, 4.5 Eq) was thinned to get 0.1 M solution (dry THF/Et_2O, 1:2, v/v) and added dropwise at 0 °C. After complete addition, the reaction was warmed to rt and stirred for 114 h at this temperature. Afterwards, the reaction was quenched through addition of a sat. NH_4Cl solution (100 mL) and the mixture stirred for 15 minutes. The aqueous phase was extracted with EtOAc (3 x 50 mL) and the combined organic phases washed with brine (2 x 100 mL). The organic

phases were dried over $MgSO_4$, filtered and the solvent removed under reduced pressure. Purification was done *via* automized flash column chromatography (SiO_2, 9 % → 20 % EtOAc in hexane) to obtain the **2a** as yellow solid.

Yield: 320 mg, 1.01 mmol, 19 %.

R_f = 0.63 (hexanes/EtOAc 10:1)

^{1}H NMR (400 MHz, $CDCl_3$, 25 °C) δ_H [ppm]: 7.72 – 7.67 (m, 2H), 7.52 – 7.46 (m, 1H), 7.44 – 7.39 (m, 2H).

^{13}C NMR (101 MHz, $CDCl_3$, 25 °C): (δ) [ppm] = 171.4, 161.5, 133.5, 131.3, 128.8, 128.8, 119.9, 95.8, 86.2.

UV/Vis: λ_{max} (ε) [nm] = 230 (17000), 241 (15700), 318 (39600)

HRMS (APPI): calc. for $(C_{19}H_{11}ClN_3)^+$: 316.0636; found *m/z* = 316.0634 [M+H]$^+$.

2.2.2 **5:** *2-chloro-4,6-bis(3-phenylbicyclo[2.2.1]hepta-2,5-dien-2-yl)-1,3,5-triazine*

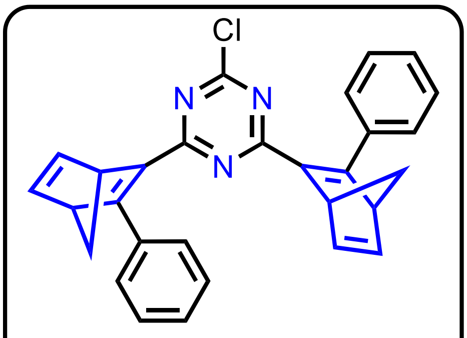

Chemical Formula: $C_{29}H_{22}ClN_3$
Molecular Weight: 447.97

The respective cyanuric acetylene 2**a** (100 mg, 0.316 mmol, 1.0 Eq) and freshly cracked cyclopentadiene (CP) (391 µL, 1.58 mmol, 15 Eq) were combined in a microwave pressure tube and diluted with 1.5 ml toluene. The reaction vessel was sealed and heated to 140 °C for 18 h using microwave irradiation. At this point a ratio of 6/71/23 for starting material/mono Diels-Alder adduct/product was found using GCMS analysis. Therefore, another 5.0 equivalents of CP (130 µL, 2.00 mmol, 5.0 Eq.) were added and the reaction temperature increased to 150 °C for 20 h. After this time, reaction was monitored using GCMS analysis yielding a ratio of 0/4/96. The solvent and excessive cyclopentadiene were removed under reduced pressure. Purification was achieved *via* automized flash column chromatography (SiO_2, 8 % EtOAc in hexane) yielding the product as yellow solid.

Yield: 95.8 mg, 0.214 mmol, 68 %.

R_f = 0.51 (hexanes/EtOAc 10:1)

^{1}H NMR (400 MHz, $CDCl_3$, 25 °C) δ_H [ppm]: 7.54 – 7.48 (m, 4H), 7.39 – 7.30 (m, 6H), 6.95 – 6.90 (m, 2H), 6.89 – 6.83 (m, 2H), 4.20 – 4.15 (m, 2H), 3.91 – 3.88 (m, 2H), 2.30 – 2.23 (m, 2H), 2.08 – 2.04 (m, 2H).

^{13}C NMR (101 MHz, $CDCl_3$, 25 °C): (δ) [ppm] = 171.3, 171.3, 170.3, 168.1, 168.1, 145.0, 145.0, 144.0, 141.1, 141.0, 136.8, 136.7, 128.6, 128.6, 128.2, 128.2, 127.8, 127.7, 69.8, 69.7, 60.0, 59.9, 53.3, 53.3.

IR (ATR, cm^{-1}): $\tilde{v}$= 3060 (w), 2967 (w), 2937 (w), 2868 (w), 1592 (w), 1505 (s), 1489 (s), 1464 (m) 1380 (m), 1266 (m), 1254 (m), 825 (m), 762 (s), 708 (s) cm^{-1}

UV/Vis: λ_{max} (ε) [nm] = 228 (20100), 357 (9600); λ_{onset} [nm] = 443 nm

HRMS (APPI): calc. for $(C_{29}H_{23}ClN_3)^+$: 448.1575; found m/z = 448.1582 $[M+H]^+$.

2.2.3 **10:** *2-chloro-4,6-bis(5-phenyltetracyclo[3.2.0.0^{2,7}.0^{4,6}]heptan-1-yl)-1,3,5-triazine*

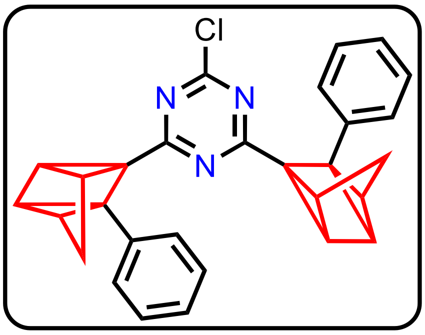

Switching of **5** was conducted according to general procedure D. The NBD was dissolved in DCM-d_2 and irradiated at 367 nm (3.6 V, 0.37 A). Quantitative conversion could be achieved after 7min in NMR scale. The same results were found in MeCN-d_3 accompanied slight precipitation and additional photodegradation. In UV/Vis scale (general procedure E), full isomerization to **10** was obtained after 10 seconds in $CHCl_3$ with subsequent decomposition after prolonged irradiation. In MeCN complete conversion was found after 8 seconds.

Yield: Quantitative (367 nm (3.6 V, 0.37 A) DCM-d_2, 7 minutes)

Quantitative (365 nm (30 %) $CDCl_3$, 5 minutes)

Quantitative (365 nm (30 %) toluene-d_8, 5 minutes)

Insoluble in MeCN.

^{1}H NMR (400 MHz, $CDCl_3$, 25 °C) δ_H [ppm]: 7.34 – 7.16 (m, 10H), 2.51– 2.48 (d, 1H), 2.44 – 2.41 (m, 1H), 2.40 – 2.36 (m, 2H), 2.36 – 2.33 (m, 1H), 2.32 – 2.29 (m, 1H), 2.23 (ddd, J = 9.5, 4.9, 2.4 Hz, 2H), 2.16 – 2.10 (m, 2H), 1.79 – 1.74 (m, 2H).

^{13}C NMR (MHz, $CDCl_3$, 25 °C): (δ) [ppm] = 178.7, 168.7, 137.4, 129.7, 129.6, 127.5, 126.2, 126.1, 41.1, 41.0, 37.2, 37.2, 34.5, 34.4, 33.3, 33.2, 32.9, 32.7, 32.2, 32.2, 21.3, 21.3.

UV/Vis: λ_{max} (ε) [nm] = 292 (7200)

2.3 Dimethylaniline-mono-NBD hybrid

2.3.1 **1c:** *4-((4,6-dichloro-1,3,5-triazin-2-yl)ethynyl)-N,N-dimethylaniline*

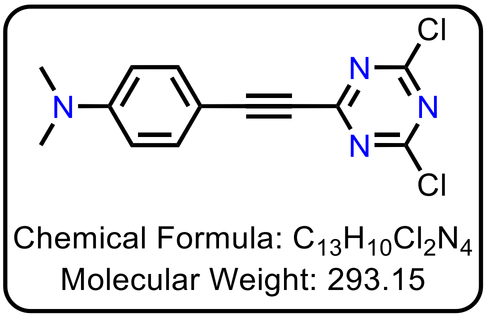

The alkynylmagnesium bromide was prepared according to a procedure adapted from literature.[3] In a flame dried, argon containing apparatus with dropping funnel and dimroth condenser 4-ethynyl-*N*,*N*-dimethylaniline (1.18 g, 8.13 mmol, 1.0 Eq) was dissolved in a 1:2 mixture of anhydrous THF and Et_2O (27.1 mL and 51.5 mL, respectively). The reaction was cooled to 0 °C and a EtMgBr solution (3M in Et_2O, 2.71 mL, 8.13 mmol, 1.0 Eq) was diluted to get 0.1 M solution (THF/Et_2O, 1:2, v/v) and added dropwise at 0 °C over 45 minutes. After complete addition, the mixture was further stirred for 15 min at 0 °C. The reaction was slowly warmed to rt and then heated to 50 °C for 1 h. Afterwards, the reaction was cooled to rt and the obtained Grignard reagent used for the next step without additional purification.

The nucleophilic substitution was performed based on a procedure adapted from literature.[4] Cyanuric chloride (1.00 g, 5.42 mmol, 1.0 Eq) was put in a flame dried argon containing flask and dissolved in a 1:2 mixture of anhydrous THF and Et_2O (18.1 mL and 36.2 ml, respectively) to get a 0.1 M solution and cooled to 0 °C. The previously prepared alkynylmagnesium bromide (8.13 mmol, 1.5 Eq) was thinned to get 0.1 M solution (dry THF/Et_2O, 1:2, v/v) and added dropwise at 0 °C. After complete addition, the reaction was further stirred for 2 h at 0 °C and subsequently warmed to rt and stirred for 42 h at this temperature. Afterwards, the reaction was quenched through addition of a sat. NH_4Cl solution (100 mL) and the mixture stirred for 15 minutes. The aqueous phase was extracted with EtOAc (3 x 50 mL) and the combined organic phases washed with brine (2 x 100 mL). The organic phases were dried over $MgSO_4$, filtered and the solvent removed under reduced pressure. Purification was done *via* automized flash column chromatography (SiO_2, 15 % EtOAc in hexane) to obtain the **1c** as orange solid.

Yield: 254 mg, 0.866 mmol, 16 %.

R_f = 0.44 (hexanes/EtOAc 4:1)

^{1}H NMR (400 MHz, $CDCl_3$, 25 °C) δ_H [ppm]: 7.59 – 7.55 (m, 2H), 6.67 – 6.62 (m, 2H), 3.06 (s, 6H).

^{13}C NMR (100 MHz, $CDCl_3$, 25 °C): (δ) [ppm] = 171.4, 162.2, 152.4, 135.9, 111.7, 104.9, 103.6, 87.5, 40.1.

UV/Vis: λ_{max} (ε) [nm] = 270 (11800), 432 (30700)

HRMS (APPI): calc. for $(C_{13}H_{11}Cl_2N_4)^+$: 293.0355; found *m/z* = 293.0361[M+H]$^+$.

2.3.2 **6:** *4-(3-(4,6-dichloro-1,3,5-triazin-2-yl)bicyclo[2.2.1]hepta-2,5-dien-2-yl)-N,N-dimethylaniline*

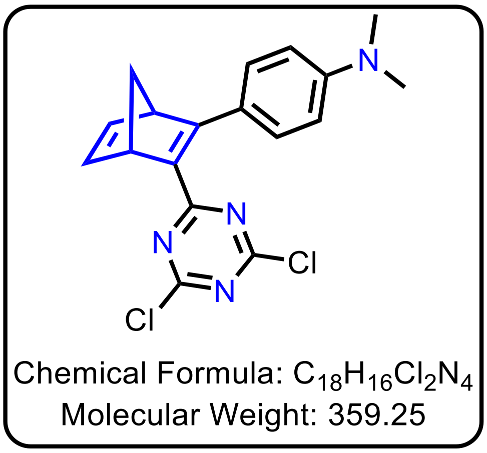

The respective cyanuric acetylene **1c** (91.9 mg, 0.313 mmol, 1.0 Eq) and freshly cracked cyclopentadiene (CP) (388 µL, 4.70 mmol, 15 Eq) were combined in a microwave pressure tube and diluted with 5.5 ml toluene. The reaction vessel was sealed and heated to 180 °C for 4 h using microwave irradiation. At this point GCMS analysis showed full conversion. The solvent and excessive cyclopentadiene were removed under reduced pressure. Purification was achieved *via* automized flash column chromatography (SiO_2, 10 % EtOAc in hexanes) yielding the product as red solid.

Yield: 25.3 mg, 70.4 µmol, 22 %.

R_f = 0.61 (hexanes/EtOAc 7:1)

^{1}H NMR (400 MHz, $CDCl_3$, 25 °C) δ_H [ppm]: 7.79 – 7.74 (m, 2H), 7.02 – 6.99 (m, 1H), 6.84 – 6.81 (m, 1H), 6.71 – 6.67 (m, 2H), 4.45 – 4.42 (m, 1H), 4.06 – 4.02 (m, 1H), 3.06 (s, 6H), 2.21 – 2.18 (m, 1H), 2.10 – 2.06 (m, 1H).

^{13}C NMR (101 MHz, $CDCl_3$, 25 °C): (δ) [ppm] = 175.0, 172.1, 170.4, 151.8, 143.7, 139.9, 137.7, 131.6, 110.6, 67.5, 59.9, 52.8, 40.3, 22.8.

UV/Vis: λ_{max} (ε) [nm] = 273 (19600), 318 (5500), 482 (17600); λ_{onset} [nm] = 594 nm

HRMS (APPI): calc. for $(C_{18}H_{17}Cl_2N_4)^+$: 359.0825; found *m/z* = 359.0826 [M+H]$^+$.

2.3.3 **11:** *4-(5-(4,6-dichloro-1,3,5-triazin-2-yl)tetracyclo[3.2.0.0^{2,7}.0^{4,6}]heptan-1-yl)-N,N-dimethylaniline*

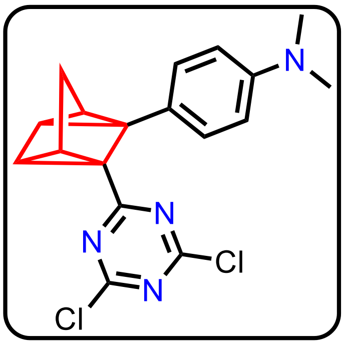

Switching of **6** was conducted according to general procedure D for NMR scale and general procedure E for UV/Vis experiments. In both cases, photodecomposition with massive precipitation was found in every tested solvent (MeCN-d_3, $CDCl_3$ and benzene-d_6). Indicated via mass spectrometry, potential poly- or dimerimerization is suggested (compare SI-Section 4).

Yield: Decomposition

HRMS (APPI): Calced. for $C_{36}H_{32}Cl_4N_8Na^+$: 741.1367, found 741.1146 $[2M+Na]^+$

2.4 Dimethylaniline-phenyl-bis-NBD hybrid

2.4.1 **2b:** *1Ph1NMeAc: 4-((4-chloro-6-(phenylethynyl)-1,3,5-triazin-2-yl)ethynyl)-N,N-dimethylaniline*

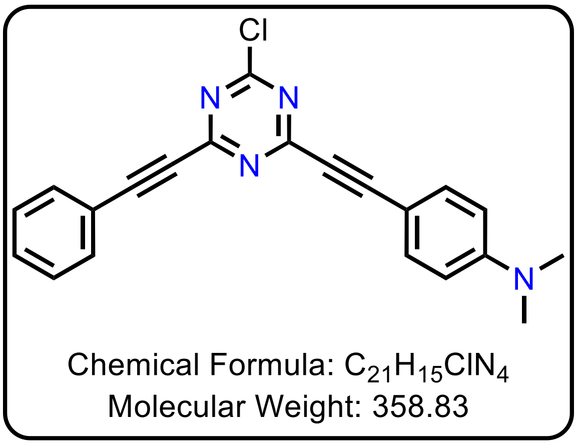

The alkynylmagnesium bromide was prepared according to a procedure adapted from literature.[3] In a flame dried, argon containing apparatus with dropping funnel and dimroth condenser 4-ethynyl-*N*,*N*-dimethylaniline (2.84 g, 19.6 mmol, 1.0 Eq) was dissolved in a 1:2 mixture of anhydrous THF and Et_2O (9.0 mL and 11.5 mL, respectively). The reaction was cooled to 0 °C and a EtMgBr solution (3M in Et_2O, 6.53 mL, 19.6 mmol, 1.0 Eq) was diluted with anhydrous THF (9.0 mL) and anhydrous Et_2O (11.5 mL) and added dropwise at 0 °C over 1 h. After complete addition, the mixture was warmed to rt fand stirred for 30 min. Subsquently, the reaction was diluted f anhydrous THF and Et_2O (10.0 mL and 20.0 mL, respectively) heated to 50 °C for 1 h. Afterwards, the reaction was cooled to rt and the obtained Grignard reagent used for the next step without additional purification.

The nucleophilic substitution was performed based on a procedure adapted from literature.[4] **1a** (1.36 g, 5.44 mmol, 1.0 Eq) was put in a flame dried argon containing flask and dissolved in a 1:2 mixture of anhydrous THF and Et_2O (18.1 mL and 36.2 ml, respectively) to get a 0.1 M solution and

cooled to 0 °C. The previously prepared alkynylmagnesium bromide (8.16 mmol, 1.5 Eq) was thinned to get 0.1 M solution (dry THF/Et_2O, 1:2, v/v) and added dropwise at 0 °C. After complete addition, the reaction was further stirred for 2 h at 0 °C and subsequently warmed to rt and stirred for 44 h at this temperature. Afterwards, the reaction was quenched through addition of a sat. NH_4Cl solution (100 mL) and the mixture stirred for 15 minutes. The aqueous phase was extracted with EtOAc (3 x 50 mL) and the combined organic phases washed with brine (2 x 100 mL). The organic phases were dried over $MgSO_4$, filtered and the solvent removed under reduced pressure. Purification was done *via* column chromatography (SiO_2, 5:1 hexane:EtOAc, v/v) to obtain **2b** as orange solid.

Yield: 1.113 g, 3.10 mmol, 57 %.

R_f = 0.42 (hexanes/EtOAc 5:1)

^{1}H NMR (400 MHz, $CDCl_3$, 25 °C) δ_H [ppm]: 7.71 – 7.67 (m, 2H), 7.60 – 7.56 (m, 2H), 7.50 – 7.45 (m, 1H), 7.43 – 7.38 (m, 2H), 6.67 – 6.63 (m, 2H), 3.05 (s, 6H).

^{13}C NMR (100 MHz, $CDCl_3$, 25 °C): (δ) [ppm] = 171.0, 161.7, 161.2, 152.2, 135.6, 133.4, 131.1, 128.8, 120.2, 111.7, 105.5, 101.2, 94.8, 87.3, 86.4, 40.2.
UV/Vis: λ_{max} (ε) [nm] = 278 (20600), 309 (24100), 423 (31000)

HRMS (APPI): calc. for $(C_{21}H_{16}ClN_4)^+$: 359.1058; found m/z = 359.1059[M+H]$^+$.

2.4.2 **7:** *4-(3-(4-chloro-6-(3-phenylbicyclo[2.2.1]hepta-2,5-dien-2-yl)-1,3,5-triazin-2-yl)bicyclo[2.2.1]hepta-2,5-dien-2-yl)-N,N-dimethylaniline*

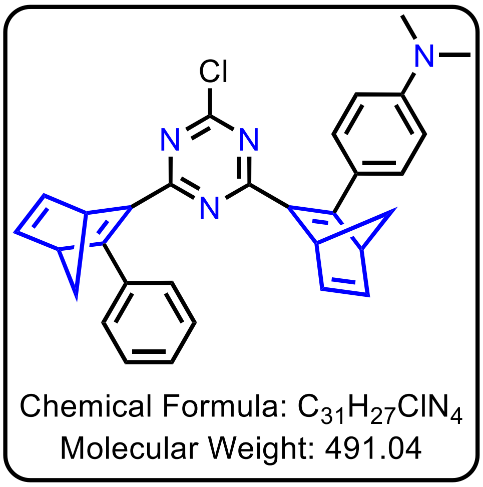

The respective cyanuric acetylene **2b** (100 mg, 0.279 mmol, 1.0 Eq) and freshly cracked cyclopentadiene (CP) (346 µL, 4.19 mmol, 15 Eq) were combined in a microwave pressure tube and diluted with 6.0 ml toluene. The reaction vessel was sealed and heated to 150 °C for 22 h using microwave irradiation. At this point a ratio of 20/4/72/4 for starting material/mono Amine-Diels-Alder adduct/ mono Phenyl-Diels-Alder adduct/product was found using GCMS analysis. Therefore, another 5.0 equivalents of CP (115 µL, 1.40 mmol, 5.0 Eq.) were added and the reaction temperature increased to 170 °C for 4 h which changed the ratio to 0/5/45/50. After further 12 h at 170 °C, reaction was monitored again yielding a ratio of 0/0/11/89. To prevent dimer formation, the reaction was stopped and the solvent and excessive cyclopentadiene were removed

under reduced pressure. Purification was achieved *via* automized flash column chromatography (SiO_2, 15 % EtOAc in hexanes) yielding the product as red solid.

Alternatively: The respective cyanuric acetylene **2b** (82.9 mg, 0.231 mmol, 1.0 Eq) and freshly cracked cyclopentadiene (207 µL, 2.51 mmol, 15 Eq) were combined in a microwave pressure tube and diluted with 6.0 ml toluene. The reaction vessel was sealed and heated to 170 °C for 16 h using microwave irradiation. At this point 90% product formation was found using GCMS analysis. Purification was achieved *via* automized flash column chromatography (SiO_2, 10 % EtOAc in hexanes) yielding the product as red solid. However, using this procedure with direct prolonged heating to 170 °C, dimer formation already occurred which could not be separated afterwards.

Yield: 36.7 mg, 74.7 µmol, 32 %.

$\mathbf{R_f}$ = 0.53 (hexanes/EtOAc 5:1)

^{1}H NMR (400 MHz, $CDCl_3$, 25 °C) δ_H [ppm]: 7.70 – 7.64 (m, 2H), 7.56 – 7.50 (m, 2H), 7.39 – 7.29 (m, 3H), 7.06 – 7.02 (m, 0.5H), 6.99 – 6.93 (m, 1H), 6.92 – 6.86 (m, 1H), 6.85 – 6.81 (m, 0.5H), 6.80 – 6.76 (m, 1H), 6.70 – 6.65 (m, 2H), 4.37 – 4.34 (m, 1H), 4.17 – 4.10 (m, 1H), 3.96 – 3.89 (m, 2H), 3.02 (s, 6H), 2.35 – 2.28 (m, 1H), 2.18 – 2.11 (m, 1H), 2.11 – 2.07 (m, 1H), 2.00 – 1.96 (m, 1H).

^{1}H NMR (600 MHz, $CDCl_3$, 25 °C) δ_H [ppm]: 7.69 – 7.65 (m, 2H), 7.55 – 7.50 (m, 2H), 7.38 –7.34 (m, 2H), 7.33 – 7.30 (m, 1H), 7.05 – 7.03 (m, 0.5H), 6.98 – 6.93 (m, 1H), 6.91 – 6.86 (m, 1H), 6.84 – 6.82 (m, 0.5H), 6.80 – 6.76 (m, 1H), 6.69 – 6.66 (m, 2H), 4.37 – 4.35 (m, 1H), 4.16 – 4.14 (m, 0.5H), 4.13 – 4.11 (m, 0.5H), 3.96 – 3.93 (m, 1H), 3.92 – 3.90 (m, 1H), 3.02 (s, 6H), 2.34 – 2.29 (m, 1H), 2.18 – 2.12 (m, 1H), 2.10 – 2.07 (m, 1H), 2.00 – 1.96 (m, 1H).

Both measurements performed at 400 and 600 MHz are provided due to the different splitting pattern according to the field strength. This results also in slightly different integration values.

^{13}C NMR (151 MHz, $CDCl_3$, 25 °C): (δ) [ppm] = 171.5, 171.2, 171.2, 170.0, 170.0, 168.9, 167.1, 167.1, 151.1, 145.3, 145.3, 144.1, 144.0, 143.9, 143.8, 141.2, 141.1, 140.3, 140.2, 140.2, 136.9, 136.9, 130.7, 130.6, 128.5, 128.5, 128.2, 128.2, 127.8, 127.7, 123.9, 123.9, 110.9, 110.9, 70.0, 69.8, 68.1, 68.0, 59.9, 59.8, 59.5, 59.4, 53.5, 53.2, 40.4.

IR (ATR, cm^{-1}): $\tilde{v}$ = 3049 (w), 2962 (w), 2934 (w), 2865 (w), 2802 (w), 1605 (m), 1485 (s), 1456 (m), 1443 (m), 1358 (m), 1252 (m), 1194 (m), 815 (m), 731 (s), 706 (s) cm^{-1}

UV/Vis: λ_{max} (ε) [nm] =229 (17300), 270 (17000), 324 (10500), 351 (10200), 455 (12400);
λ_{onset} [nm] = 557 nm

HRMS (APPI): calc. for $(C_{31}H_{28}ClN_4)^+$: 491.1997 ; found *m/z* = 491.2006 $[M+H]^+$.

2.4.3 **12:** *4-(5-(4-chloro-6-(5-phenyltetracyclo[3.2.0.0^{2,7}.0^{4,6}]heptan-1-yl)-1,3,5-triazin-2-yl)tetracyclo[3.2.0.0^{2,7}.0^{4,6}]heptan-1-yl)-N,N-dimethylaniline*

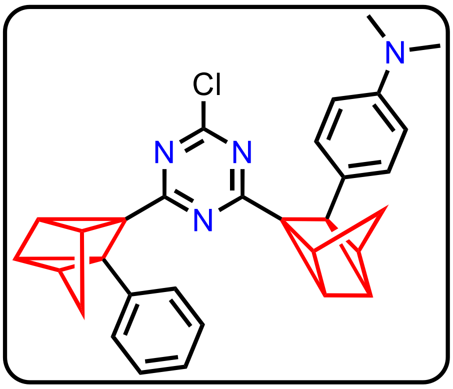

Switching of **7** was conducted according to general procedure D. The NBD was dissolved in $CDCl_3$ and irradiated at 340 nm (3.3 V, 0.38 A). Quantitative conversion could be achieved after 38 min in NMR scale. In UV/Vis scale (general procedure E), full isomerization to **12** was obtained after 60 seconds MeCN.

Yield: quantitative (38 min, 340 nm, $CDCl_3$)

^{1}H NMR (600 MHz, $CDCl_3$, 25 °C) δ_H [ppm]: ^{1}H NMR (601 MHz, Chloroform-*d*) δ 7.23 – 7.14 (m, 5H), 7.13 – 7.10 (m, 2H), 6.66 – 6.63 (m, 2H), 2.90 (d, *J* = 2.8 Hz, 6H), 2.49 – 2.47 (m, 1H), 2.44 (dd, *J* = 4.9, 2.4 Hz, 0.5H), 2.40 – 2.38 (m, 1H), 2.36 – 2.31 (m, 3H), 2.27 – 2.25 (m, 0.5H), 2.22 – 2.20 (m, 1H), 2.12 – 2.07 (m, 3H), 1.75 – 1.73 (m, 1H), 1.72 – 1.69 (m, 1H).

^{13}C NMR (151 MHz, $CDCl_3$, 25 °C): (δ) [ppm] = 178.9, 178.9, 178.4, 168.5, 168.5, 149.5, 149.5, 137.3, 130.6, 130.6, 129.5, 129.4, 127.4, 127.4, 126.0, 126.0, 125.2, 125.2, 112.2, 112.2, 41.4, 41.4, 41.0, 41.0, 40.9, 40.7, 40.3, 40.2, 37.2, 37.0, 34.4, 34.2, 34.1, 33.1, 33.1, 32.9, 32.8, 32.7, 32.0, 32.0, 31.7, 31.6, 21.2, 21.2, 20.8, 20.7.

UV/Vis: λ_{max} (ε) [nm] =263 (23000)

2.4.4 **12b:** *4-(3-(4-chloro-6-(5-phenyltetracyclo[3.2.0.0^{2,7}.0^{4,6}]heptan-1-yl)-1,3,5-triazin-2-yl)bicyclo[2.2.1]hepta-2,5-dien-2-yl)-N,N-dimethylaniline*

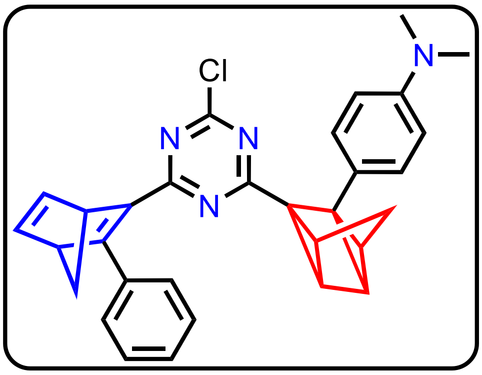

Switching of **7** was conducted according to general procedure D. The NBD was dissolved in $CDCl_3$ and irradiated at 475 nm. Quantitative conversion could be achieved after 20 min in NMR scale. In UV/Vis scale (general procedure E), full isomerization to **12b** was obtained after 420 seconds MeCN. Subsequent irradiation at 340 nm yielded to **7** also in quantitative fashion.

Yield: quantitative (20 min, 475 nm, $CDCl_3$)

^{1}H NMR (400 MHz, $CDCl_3$, 25 °C) δ_H [ppm]: 7.51 – 7.45 (m, 2H), 7.35 – 7.26 (m, 4H), 7.26 – 7.22 (m, 1H), 6.85 – 6.80 (m, 1H), 6.78 – 6.68 (m, 3H), 3.86 – 3.82 (m, 1.5H), 3.77 – 3.74 (m, 0.5H), 3.02 – 2.99 (m, 1H), 2.93 (d, *J* = 2.0 Hz, 6H), 2.75 (dd, *J* = 4.9, 2.4 Hz, 0.5H), 2.69 – 2.66 (m, 1H), 2.63 – 2.60 (m, 0.5H), 2.49 – 2.41 (m, 1.5H), 2.24 – 2.19 (m, 2H), 2.18 – 2.13 (m, 1.5H), 1.93 (m, 1H), 1.81 – 1.78 (m, 1H).

^{13}C NMR (101 MHz, $CDCl_3$, 25 °C): (δ) [ppm] = 144.2, 140.7, 140.6, 131.0, 130.9, 128.6, 128.3, 128.3, 127.6, 127.5, 112.6, 112.6, 69.5, 69.5, 59.8, 59.8, 53.3, 53.2, 42.1, 42.0, 41.1, 35.2, 35.2, 33.2, 32.3, 32.3, 31.8, 31.7, 21.0, 21.0.

UV/Vis: λ_{max} (ε) [nm] = 235 (17200), 264 (21000), 343 (10600).

2.4.5 **12a:** *4-(3-(4-chloro-6-(5-phenyltetracyclo[3.2.0.0^{2,7}.0^{4,6}]heptan-1-yl)-1,3,5-triazin-2-yl)bicyclo[2.2.1]hepta-2,5-dien-2-yl)-N,N-dimethylaniline*

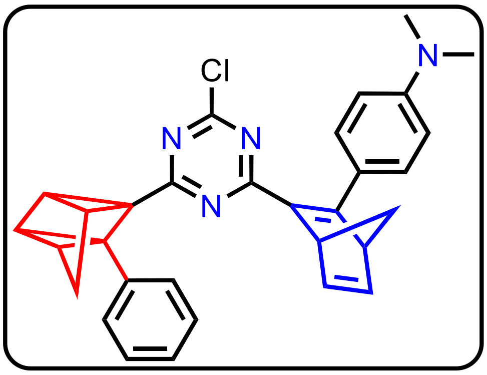

Only observed partially in the UV/Vis experiments. Observation during the NMR monitoring studies was not possible. The following UV/Vis absorption data are approximations which in accordance with the insights gained from the calculated data.

UV/Vis: λ_{max} = 267 nm, 449 nm; leftover absorption shoulders at 322 nm and 357 nm.

2.5 Asymmetric dimethylaniline-anisyl-phenyl-tris-NBD hybrid

2.5.1 **3:** *4-((4-((4-methoxyphenyl)ethynyl)-6-(phenylethynyl)-1,3,5-triazin-2-yl)ethynyl)-N,N-dimethylaniline*

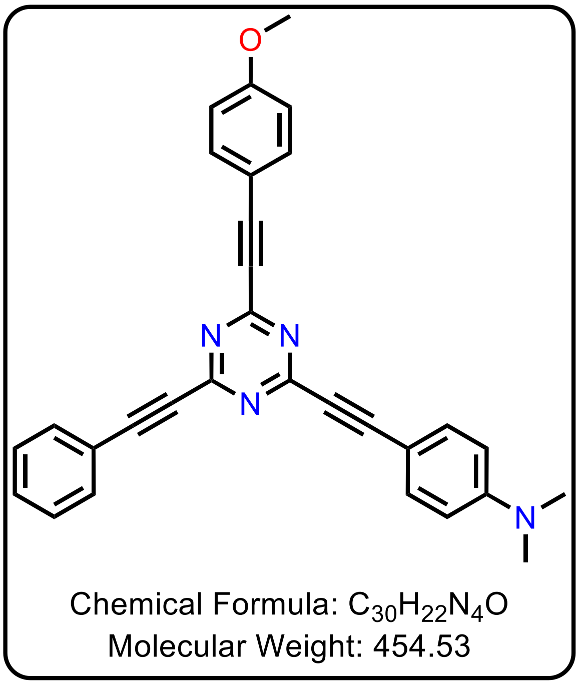

The zinc-acetylene species necessary for the subsequent Negishi couplings were prepared according to a procedure adapted from literature.[5] In a flame dried, argon containing apparatus phenyl acetylene (316 µL, 2.88 mmol, 1.0 Eq) was dissolved in anhydrous THF (5 mL) and cooled to -78 °C. *n*BuLi (2.5 M in hexane, 1.38 mL, 3.48 mmol, 1.2 Eq) was added dropwise over 30 minutes and the resulting mixture stirred at -78 °C for 30 minutes. Afterwards, $ZnCl_2$ (472 mg, 3.48 mmol, 1.2 Eq) was added in one portion and the reaction further stirred at -78 °C for 1 h. The colling bath was removed, the mixture warmed to rt and stirred at this temperature for 1.5h before the obtained zinc acetylene was used for the next step without additional purification.

The Negishi cross-coupling reaction was carried out according to a procedure adapted from literature procedures.[5],[6] **2c** (280 mg, 0.720 mmol, 1.0 Eq) and $Pd(PPh_3)_4$ (41.6 mg, 36.0 µmol, 0.05 Eq) were put in a flame dried argon containing pressure flask and dissolved in 5 mL anhydrous NMP. The previously prepared zinc acetylene (2.88 mmol, 4.0 Eq) was transferred to the reaction vessel, and

the mixture heated to 50 °C for 20 h. Afterwards, the reaction was quenched through addition of H_2O (10 mL). The aqueous phase was extracted with EtOAc (3 x 30 mL) and the combined organic phases washed with brine (2 x 75 mL). The organic phases were dried over $MgSO_4$, filtered and the solvent removed under reduced pressure. Purification was done *via* automized flash column chromatography (SiO_2, 25% EtOAc in hexane) to obtain **3** as orange solid.

Yield: 73.3 mg, 0.161 mmol, 22 %.

R_f = 0.55 (hexanes/EtOAc 2:1)

^{1}H NMR (600 MHz, $CDCl_3$, 25 °C) δ_H [ppm]: 7.71 – 7.68 (m, 2H), 7.67 – 7.64 (m, 2H), 7.61 – 7.58 (m, 2H), 7.48 – 7.44 (m, 1H), 7.42 – 7.38 (m, 2H), 6.94 – 6.91 (m, 2H), 6.67 – 6.64 (m, 2H), 3.86 (s, 3H), 3.05 (s, 6H).

^{13}C NMR (151 MHz, $CDCl_3$, 25 °C): (δ) [ppm] = 161.7, 160.9, 160.6, 160.4, 151.9, 135.3, 135.3, 133.3, 130.7, 128.7, 120.6, 114.5, 112.5, 111.7, 106.1, 98.6, 94.5, 93.0, 87.2, 86.9, 86.6, 55.6, 40.2.

IR (ATR, cm^{-1}): $\tilde{v}$ = 3066 (w), 2963 (w), 2930 (w), 2852 (w), 1607 (w), 1488 (s), 1374 (w), 1295 (m), 1249 (m), 1035 (w), 828 (w), 708 (m) cm^{-1}

UV/Vis: λ_{max} (ε) [nm] = 242 (25200), 251 (24000), 285 (25700), 296 (27500), 327 (32400), 412 (31700)

HRMS (APPI): calc. for $(C_{30}H_{23}N_4O)^+$: 455.1866; found *m/z* = 455.1876[M+H] $^+$.

2.5.2 **8:** *4-(3-(4-(3-(4-methoxyphenyl)bicyclo[2.2.1]hepta-2,5-dien-2-yl)-6-(3-phenylbicyclo[2.2.1]hepta-2,5-dien-2-yl)-1,3,5-triazin-2-yl)bicyclo[2.2.1]hepta-2,5-dien-2-yl)-N,N-dimethylaniline*

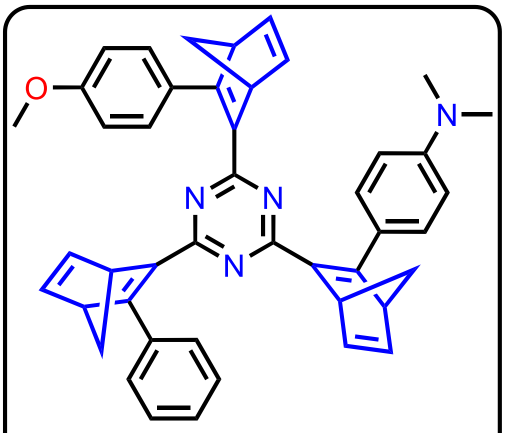

Chemical Formula: $C_{45}H_{40}N_4O$
Molecular Weight: 652.84

The respective cyanuric acetylene **3** (68.7 mg, 0.151 mmol, 1.0 Eq) and freshly cracked cyclopentadiene (188 µL, 2.27 mmol, 15 Eq) were combined in a microwave pressure tube and diluted with 6.0 ml toluene. The reaction vessel was sealed and heated to 170 °C for 18 h using microwave irradiation. The reaction was cooled to rt and the solvent and excessive cyclopentadiene were removed under reduced pressure. Purification was achieved *via*

automized flash column chromatography (SiO_2, 25 % EtOAc in hexanes) yielding the product as red solid.

Yield: 12 mg, 18.4 μmol, 12 %.

R_f = 0.74 (hexanes/EtOAc 3:1)

^{1}H NMR (400 MHz, $CDCl_3$, 25 °C) (δ) [ppm] = 7.66 – 7.46 (m, 6H), 7.38 – 7.28 (m, 3H), 6.95 – 6.61 (m, 10H), 4.14 – 4.09 (m, 1), 4.07 – 3.94 (m, 1.5H), 3.89 – 3.80 (m, 6.5H)*, 2.97 (s, 6H), 2.31 – 2.13 (m, 3H), 2.02 – 1.88 (m, 3H).

*Due to the complexity of the spectrum and minor impurities left in the sample (compare HPLC), proper integration is barely possible. *including the singlet originated by the OMe group which corresponds to 3H.*

^{1}H NMR (600 MHz, $CDCl_3$, 25 °C) (δ) [ppm] = 7.67 – 7.41 (m, 6H), 7.38 – 7.27 (m, 3H), 6.95 – 6.57 (m, 10H), 4.16 – 4.10 (m, 1H), 4.07 – 3.94 (m, 1.5H), 3.89 – 3.70 (m, 6.5H)*, 2.99 (s, 4.5H), 2.91 (s, 1.5H), 2.24 – 2.04 (m, 3H), 2.02 – 1.87 (m, 3H).

**including the singlet originated by the OMe group which corresponds to 3H.*

Two addition signals at 2.31 – 2.25 (1H) and 2.40 – 2.35 (0.5H) ppm were found corresponding to unknown impurity and already switched QC species

^{13}C NMR (151 MHz, $CDCl_3$, 25 °C): (δ) [ppm] = 170.0, 170.0, 169.9, 169.6, 169.6, 169.6, 169.4, 169.4, 168.5, 168.5, 168.4, 168.4, 168.3, 168.2, 168.2, 168.2, 164.4, 164.3, 164.3, 164.3, 163.3, 163.2, 163.2, 163.2, 163.1, 163.0, 163.0, 163.0, 162.9, 162.9, 162.8, 162.8, 162.7, 159.5, 159.4, 159.4, 159.4, 150.5, 150.5, 147.1, 147.1, 147.1, 146.9, 146.9, 145.2, 145.2, 145.2, 145.2, 145.0, 145.0, 144.5, 144.4, 144.4, 144.4, 144.3, 144.3, 144.3, 144.1, 144.1, 144.1, 142.8, 142.7, 141.3, 141.3, 141.3, 141.1, 141.0, 141.0, 140.9, 140.8, 140.8, 140.7, 140.6, 140.6, 140.5, 140.4, 140.3, 138.0, 138.0, 138.0, 137.8, 137.8, 137.8, 131.1, 131.0, 131.0, 130.9, 130.2, 130.1, 130.1, 130.1, 130.1, 130.0, 130.0, 130.0, 130.0, 130.0, 129.9, 129.9, 129.9, 129.9, 128.1, 128.1, 128.1, 128.0, 127.7, 127.7, 127.7, 127.7, 127.5, 127.5, 127.5, 127.5, 125.3, 125.2, 125.2, 113.1, 113.0, 113.0, 113.0, 112.8, 112.8, 112.8, 112.8, 112.8, 112.7, 111.2, 111.2, 111.2, 111.2, 70.1, 70.0, 70.0, 69.8, 69.3, 69.3, 69.3, 69.1, 68.6, 68.6, 68.5, 68.4, 59.7, 59.6, 59.6, 59.5, 59.4, 59.4, 59.4, 59.2, 59.1, 59.1, 55.5, 53.6, 53.6, 53.6, 53.6, 53.5, 53.5, 53.5, 53.5, 53.4, 53.4, 53.3, 53.3, 53.3, 53.3, 53.3, 53.2, 41.2, 40.6, 39.6, 39.5, 39.5, 39.4, 33.0, 32.9, 32.9, 32.8, 32.1, 32.0, 32.0, 31.9, 31.9, 31.9, 31.9.

IR (ATR, cm^{-1}): $\tilde{\nu}$=3066 (w), 2963 (w), 2930 (w), 2853 (w), 1607 (m), 1488 (s), 1374 (m), 1295 (m), 1249 (m), 1178 (w), 1035 (w), 947 (w), 828 (w), 708 (m) cm^{-1}

UV/Vis: λ_{max} (ε) [nm] = 242 (22300), 254 (21400), 300 (13900), 359 (14600), 431 (9700); λ_{onset} [nm] = 531 nm.

HRMS (APPI): calc. for $(C_{45}H_{41}N_4O)^+$: 653.3275; found *m/z* = 653.3269 $[M+H]^+$.

2.5.3 **13:** *4-(5-(4-(5-(4-methoxyphenyl)tetracyclo[3.2.0.0^{2,7}.0^{4,6}]heptan-1-yl)-6-(5-phenyltetracyclo[3.2.0.0^{2,7}.0^{4,6}]heptan-1-yl)-1,3,5-triazin-2-yl)tetracyclo[3.2.0.0^{2,7}.0^{4,6}]heptan-1-yl)-N,N-dimethylaniline*

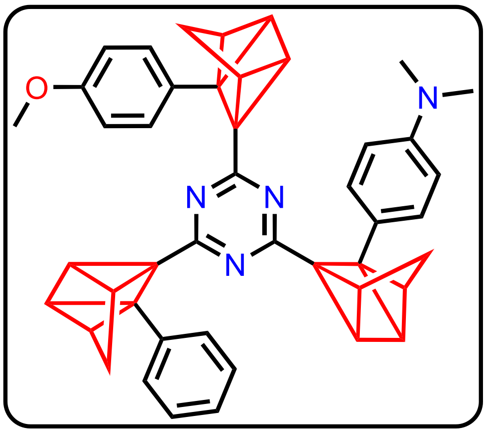

Switching of **8** was conducted according to general procedure D. The NBD was dissolved in $CDCl_3$ and irradiated at 365 nm (80%). Quantitative conversion could be achieved after 5 min in NMR scale. In UV/Vis scale (general procedure E), full isomerization to **13** was obtained after 60 seconds MeCN.

Yield: quantitative (5 min, 365 nm at 80%, $CDCl_3$)

Yield: quantitative ($CDCl_3$) 365 nm 80% 15°C 10 minutes.

^{1}H NMR (600 MHz, $CDCl_3$, 25 °C) δ_H [ppm]: 7.22 – 7.06 (m, 9H), 6.78 – 6.75 (m, 2H), 6.67 – 6.63 (m, 2H), 3.79 (m, 3H), 2.90 (m, 6H), 2.26 – 2.18 (m, 3H), 2.12 - 1.81 (m, 11H), 1.64 – 1.56 (m, 4H).

^{13}C NMR (151 MHz, $CDCl_3$, 10 °C): (δ) [ppm] = 175.0, 175.0, 174.7, 174.7, 174.7, 174.5, 174.5, 174.5, 174.5, 157.7, 157.7, 149.3, 149.3, 149.3, 149.3, 138.3, 138.3, 138.3, 130.9, 130.9, 130.8, 130.7, 130.7, 130.6, 130.6, 130.5, 129.5, 129.5, 129.4, 129.4, 127.1, 126.5, 126.5, 126.5, 125.6, 125.5, 125.5, 125.5, 112.7, 112.7, 112.5, 112.4, 55.4, 41.3, 41.3, 38.5, 38.5, 38.4, 38.3, 38.3, 38.3, 38.2, 38.0, 38.0, 37.8, 37.8, 36.4, 36.4, 36.4, 36.4, 36.3, 36.3, 36.3, 36.3, 36.3, 36.3, 36.2, 33.1, 32.9, 32.8, 32.7, 32.5, 32.5, 32.5, 32.5, 32.4, 32.3, 32.2, 32.2, 32.1, 32.1, 32.1, 32.0, 32.0, 32.0, 31.9, 31.8, 31.7, 31.7, 31.7, 31.7, 31.6, 31.6, 31.6, 31.6, 31.6, 31.6, 31.6, 31.2, 31.2, 31.1, 31.1, 31.1, 31.0, 31.0, 31.0, 31.0, 31.0, 30.9, 20.6, 20.6, 20.6, 20.6, 20.4, 20.3, 20.3, 20.2, 20.2, 20.1, 20.1.

Here, all signals are picked including the 4-fold signal split of each carbon induced by the presence of the diastereoisomers (compare spectra in section 3.5.3).

UV/Vis: λ_{max} = 236 nm, 261 nm.

2.6 Additional acetylene derivatives

2.6.1 **1b:** *2,4-dichloro-6-((4-methoxyphenyl)ethynyl)-1,3,5-triazine*

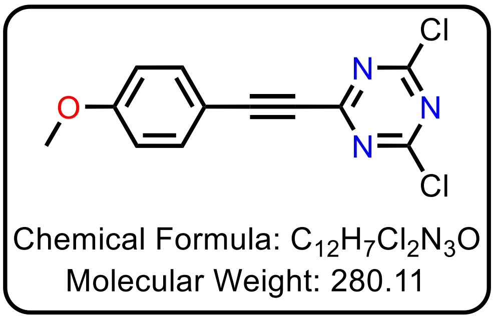

The alkynylmagnesium bromide was prepared according to a procedure adapted from literature.[3] In a flame dried, argon containing apparatus with dropping funnel and dimroth condenser 4-ethynylanisole (2.68 mL, 20.7 mmol, 1.0 Eq) was dissolved in a mixture of anhydrous THF (13.5 mL) and anhydrous Et_2O (10 mL). The reaction was cooled to 0 °C and a EtMgBr solution (3M in Et_2O, 6.90 mL, 32.5 mmol, 1.0 Eq) was diluted with 10 mL dry Et_2O was added dropwise over 30 minutes. After complete addition, the mixture was further stirred for 15 min at 0 °C. The obtained solvent ratio after resulted as 1/2 anhydrous THF/Et_2O. The reaction was slowly warmed to rt and then heated to 50 °C for 2 h. Afterwards, the reaction was cooled to rt and the obtained Grignard reagent used for the next step without additional purification.

The nucleophilic substitution was performed based on a procedure adapted from literature.[4] Cyanuric chloride (1.00 g, 5.42 mmol, 1.0 Eq) was put in a flame dried argon containing flask and dissolved in a 1:2 mixture of anhydrous THF and Et_2O (18.1 mL and 36.2 ml, respectively) and cooled to 0 °C. The previously prepared alkynylmagnesium bromide (8.13 mmol, 1.5 Eq) was thinned to get 0.1 M solution (dry THF/Et_2O, 1:2, v/v) was added dropwise at 0 °C. After complete addition, the reaction was warmed to rt and stirred for 120 h at this temperature. Afterwards, the reaction was quenched through addition of a sat. NH_4Cl solution (100 mL) and the mixture stirred for 15 minutes. The aqueous phase was extracted with EtOAc (3 x 50 mL) and the combined organic phases washed with brine (2 x 100 mL). The organic phases were dried over $MgSO_4$, filtered and the solvent removed under reduced pressure. Purification was done *via* automized flash column chromatography (SiO_2, 10 % → 30 % EtOAc in hexane) to obtain **1b** as yellow solid.

Yield: 732 mg, 2.61 mmol, 48%.

R_f = 0.47 (hexanes/EtOAc 10:1)

^{1}H NMR (400 MHz, $CDCl_3$, 25 °C) δ_H [ppm]: 7.68 – 7.64 (m, 2H), 6.95 – 6.91 (m, 2H), 3.86 (s, 3H).

^{13}C NMR (101 MHz, $CDCl_3$, 25 °C): (δ) [ppm] = 171.8, 162.6, 162.3, 135.9, 114.8, 99.4, 86.0, 55.7.

UV/Vis: λ_{max} (ε) [nm] = 240 (13200), 299 (8700), 320 (18700), 341 (28500).

HRMS (APPI): calc. for $(C_{12}H_8Cl_2N_3O)^+$: 280.0039; found *m/z* = 280.0043 $[M+H]^+$.

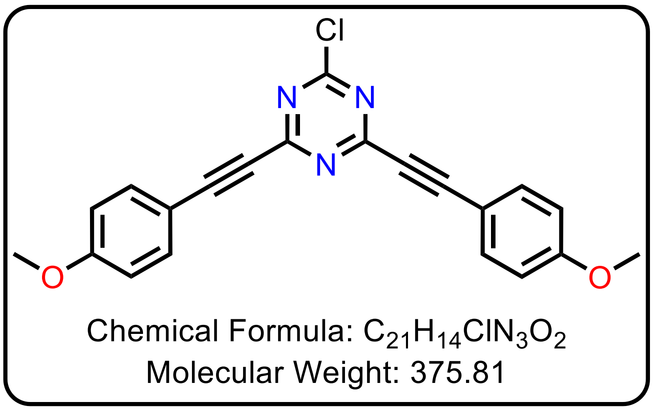

In addition, the two-fold substituted cyanuric acetylene was isolated as red solid.

Yield: 195 mg, 0.697 mmol, 13%.

R_f = 0.38 (hexanes/EtOAc 3:1)

^{1}H NMR (400 MHz, $CDCl_3$, 25 °C) δ_H [ppm]: 7.70 – 7.62 (m, 4H), 6.97 – 6.90 (m, 4H), 3.86 (s, 6H).

^{13}C NMR (101 MHz, $CDCl_3$, 25 °C): (δ) [ppm] = 171.2, 162.2, 161.5, 135.6, 114.6, 111.9, 96.9, 86.2, 55.6.

HRMS (APPI): calc. for $(C_{21}H_{15}ClN_3O_2)^+$: 376.0847; found *m/z* = 376.0850 $[M+H]^+$.

2.6.2 **2c:** *4-((4-chloro-6-((4-methoxyphenyl)ethynyl)-1,3,5-triazin-2-yl)ethynyl)-N,N-dimethylaniline*

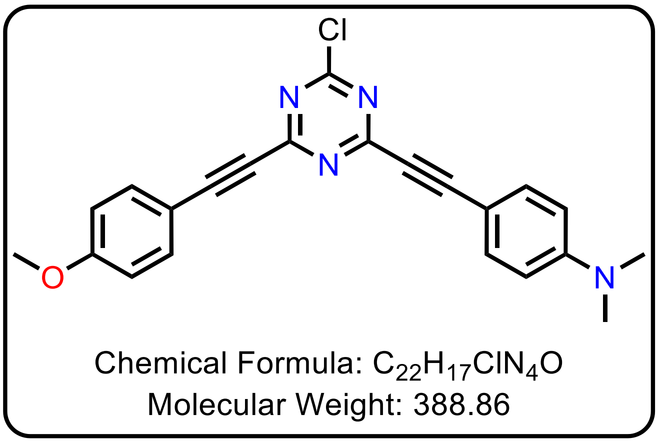

The alkynylmagnesium bromide was prepared according to a procedure adapted from literature.[3] In a flame dried, argon containing apparatus with dropping funnel and dimroth condenser 4-ethynyl-*N*,*N*-dimethylaniline (2.84 g, 19.6 mmol, 1.0 Eq) was dissolved in a 1:2 mixture of anhydrous THF and Et_2O (9.0 mL and 11.5 mL, respectively). The reaction was cooled to 0 °C and a EtMgBr solution (3M in Et_2O, 6.53 mL, 19.6 mmol, 1.0 Eq) was diluted with anhydrous THF (9.0 mL) and anhydrous Et_2O (11.5 mL) and added dropwise at 0 °C over 1 h. After complete addition, the mixture was warmed to rt fand stirred for 30 min. Subsequently, the reaction was diluted f anhydrous THF and Et_2O (10.0 mL and 20.0 mL, respectively) heated to 50 °C for 1 h. Afterwards, the reaction was cooled to rt and the obtained Grignard reagent used for the next step without additional purification.

The nucleophilic substitution was performed based on a procedure adapted from literature.[4] **1b** (600 mg, 2.14 mmol, 1.0 Eq) was put in a flame dried argon containing flask and dissolved in a 1:2 mixture of anhydrous THF and Et_2O (7.0 mL and 14 ml, respectively) to get a 0.1 M solution and

cooled to 0 °C. The previously prepared alkynylmagnesium bromide (3.21 mmol, 1.5 Eq) was thinned to get 0.1 M solution (dry THF/Et_2O, 1:2, v/v) and added dropwise at 0 °C. After complete addition, the reaction was further stirred for 2 h at 0 °C and subsequently warmed to rt and stirred for 61 h at this temperature. Afterwards, the reaction was quenched through addition of a sat. NH_4Cl solution (50 mL) and the mixture stirred for 15 minutes. The aqueous phase was extracted with EtOAc (3 x 50 mL) and the combined organic phases washed with brine (2 x 100 mL). The organic phases were dried over $MgSO_4$, filtered and the solvent removed under reduced pressure. Purification was done *via* column chromatography (SiO_2, 2:1 hexane:EtOAc, v/v) to obtain **2c** as orange solid.

Yield: 571 mg, 1.47 mmol, 69%.

R_f = 0.39 (hexanes/EtOAc 2:1)

^{1}H NMR (300 MHz, $CDCl_3$, 25 °C) δ_H [ppm]: 7.67 – 7.62 (m, 2H), 7.61 – 7.55 (m, 2H), 6.95 – 6.89 (m, 2H), 6.68 – 6.62 (m, 2H), 3.86 (s, 3H), 3.05 (s, 6H).

^{13}C NMR (151 MHz, $CDCl_3$, 25 °C): (δ) [ppm] = 170.9, 162.0, 161.5, 161.3, 152.1, 135.6, 135.4, 114.6, 112.0, 111.7, 105.6, 100.7, 96.2, 87.2, 86.2, 55.6, 40.1.

UV/Vis: λ_{max} (ε) [nm] = 248 (20500), 259 (20700), 273 (19200), 342 (28300), 421 (36500)

HRMS (APPI): calc. for $(C_{22}H_{17}ClN_4O)^+$: 388.1058; found *m/z* = 388.1086 $[M]^{+\cdot}$ and calc. for $(C_{22}H_{18}ClN_4O)^+$: 389.1164; found *m/z* = 389.1179 $[M+H]^+$.

2.6.3 **Por** [5-(p-Carboxyphenyl)-10,15,20-(p-tert-butyltriphenylphenyl)porphyrinato] cobalt (II) (carboxylic acid A_3B Cobalt (II) -Porphyrin)

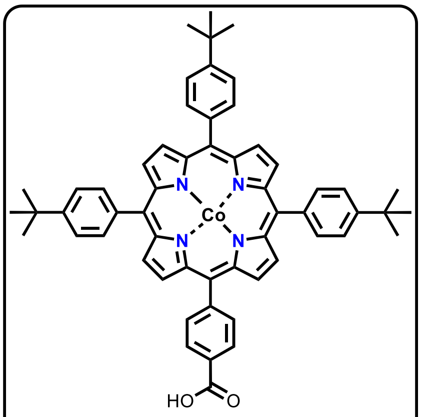

Chemical Formula: $C_{58}H_{55}CoN_4O_2$
Molecular Weight: 899.04

The carboxylic acid A_3B Cobalt (II)-Porphyrin (**Por**) which was used to investigate the catalytic back-conversion of QCs to NBDs was already available in our group and prepared according to literature. [8]

3 Characterization of the Synthesized Molecules

3.1 Phenyl-mono-NBD hybrid

3.1.1 **1a**

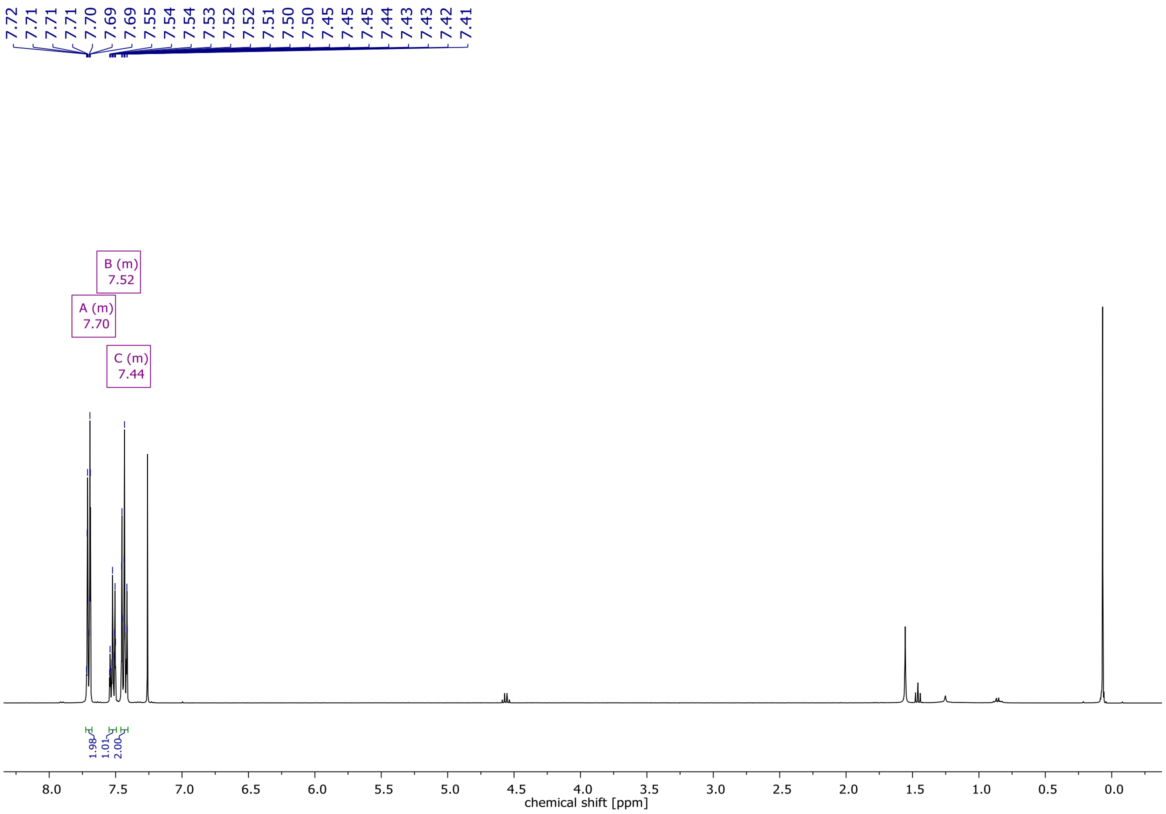

*Figure S 2: ^{1}H NMR Spectrum of **1a** measured in $CDCl_3$ (400 MHz).*

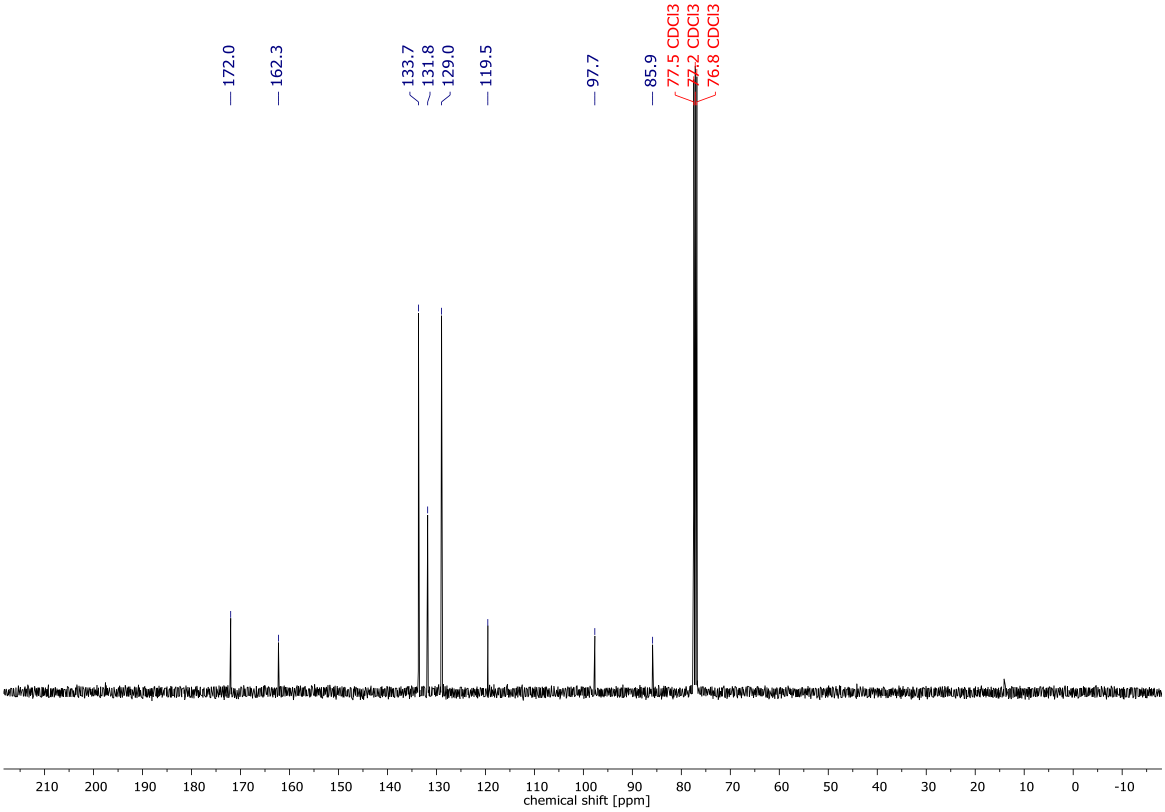

*Figure S 3: ^{13}C NMR Spectrum of **1a** measured in $CDCl_3$ (101 MHz).*

Acquisition Parameter

Source Type	APPI	Ion Polarity	Positive	Set Nebulizer	2.0 Bar
Focus	Active			Set Dry Heater	200 °C
Scan Begin	50 m/z	Set Capillary	700 V	Set Dry Gas	3.0 l/min
Scan End	1300 m/z	Set End Plate Offset	-500 V	Set Divert Valve	Waste

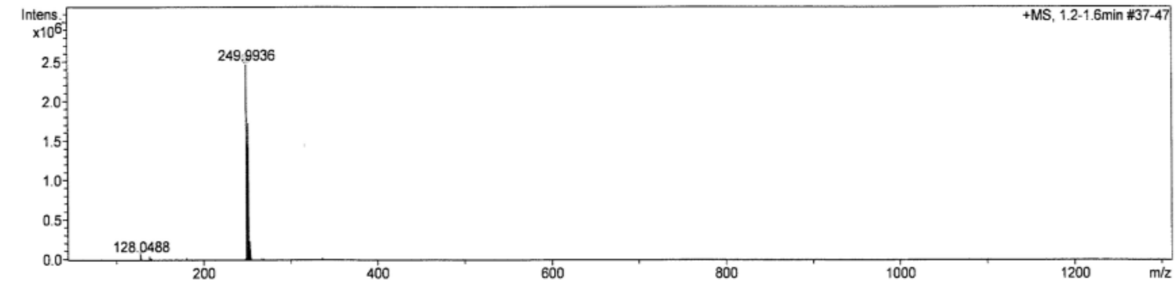

Meas. m/z	#	Ion Formula	m/z	err [ppm]	mSigma	# mSigma	Score	rdb	e⁻ Conf	N-Rule
249.9936	1	C11H6Cl2N3	249.9933	-1.0	20.6	1	100.00	9.5	even	ok

*Figure S 4: HRMS (APPI) of **1a***

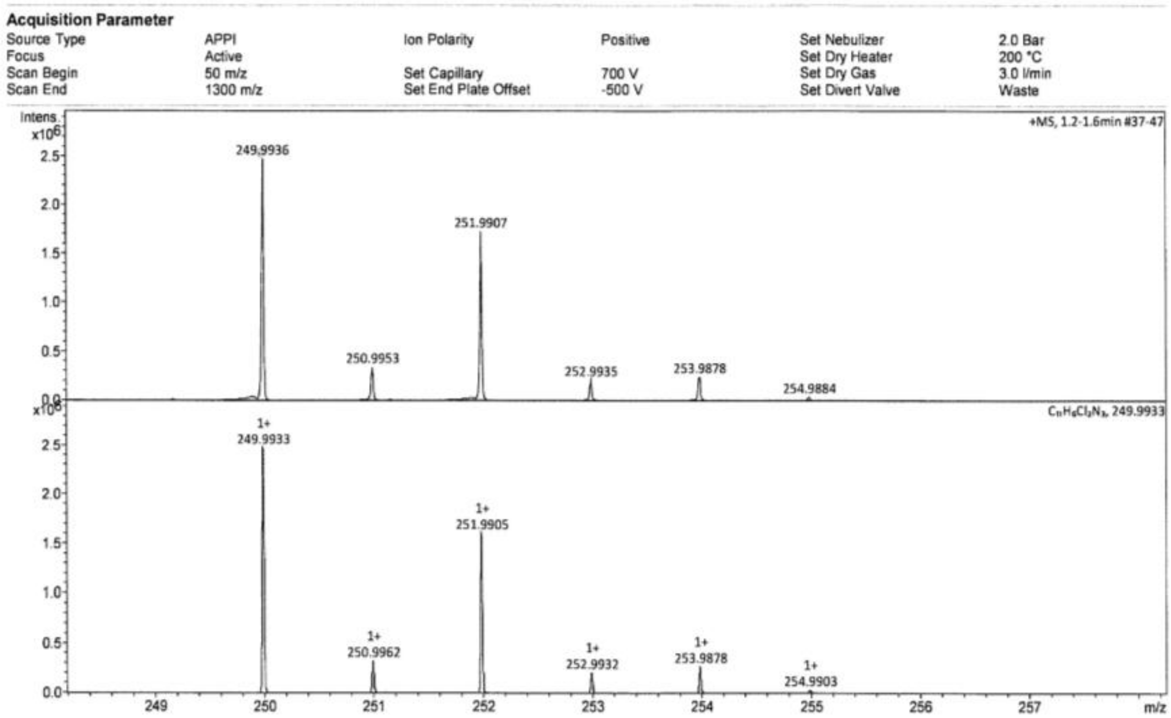

*Figure S 5: Zoom of HRMS (APPI) of **1a**.*

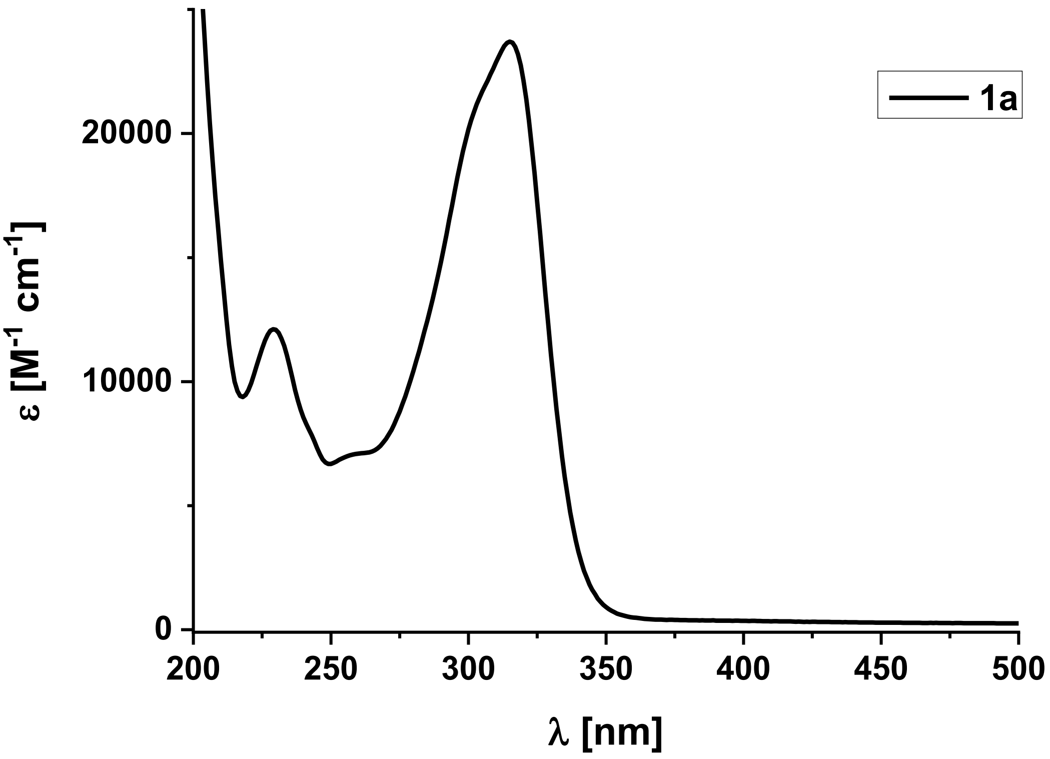

*Figure S 6: UV/Vis extinction spectrum of **1a** measured in MeCN.*

3.1.2 **4**

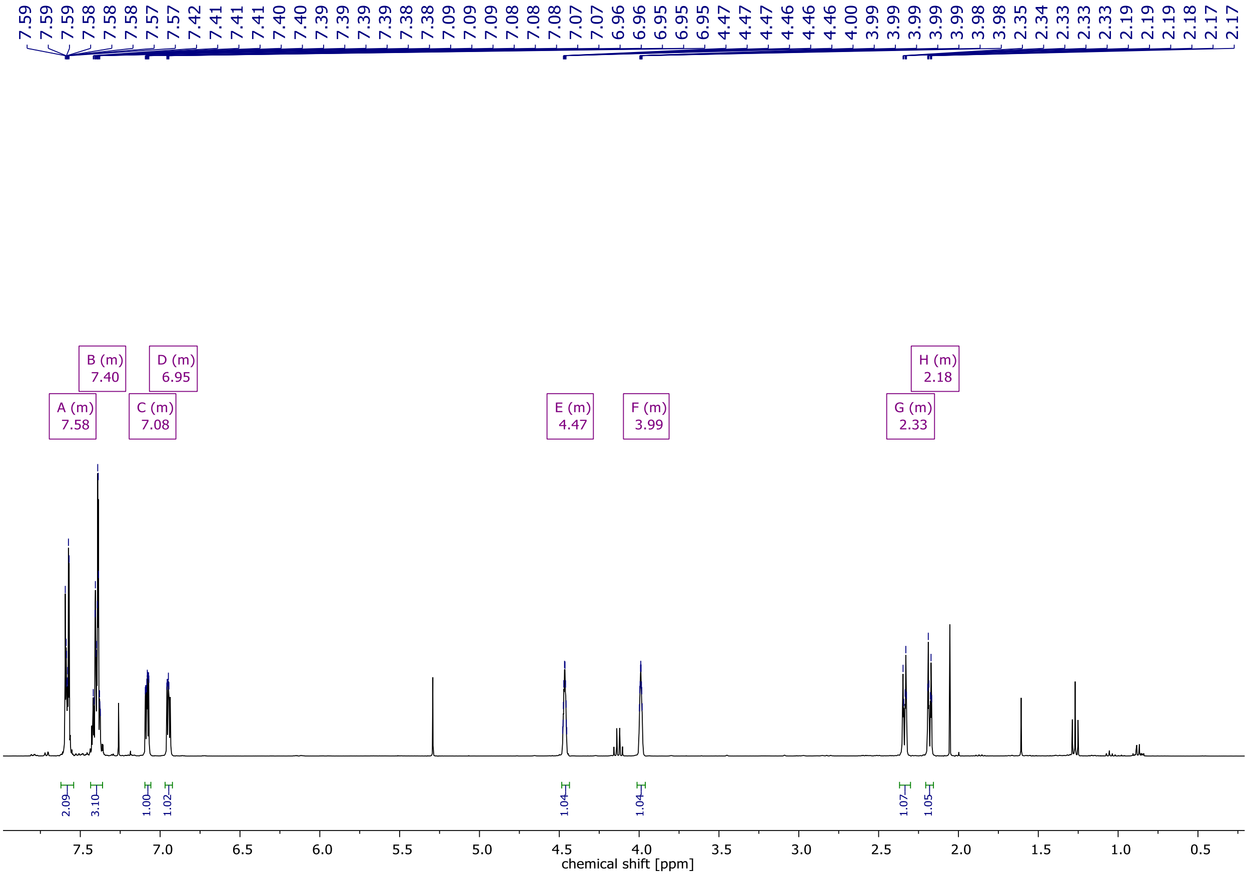

*Figure S 7: ^{1}H NMR Spectrum of **4** measured in $CDCl_3$ (400 MHz).*

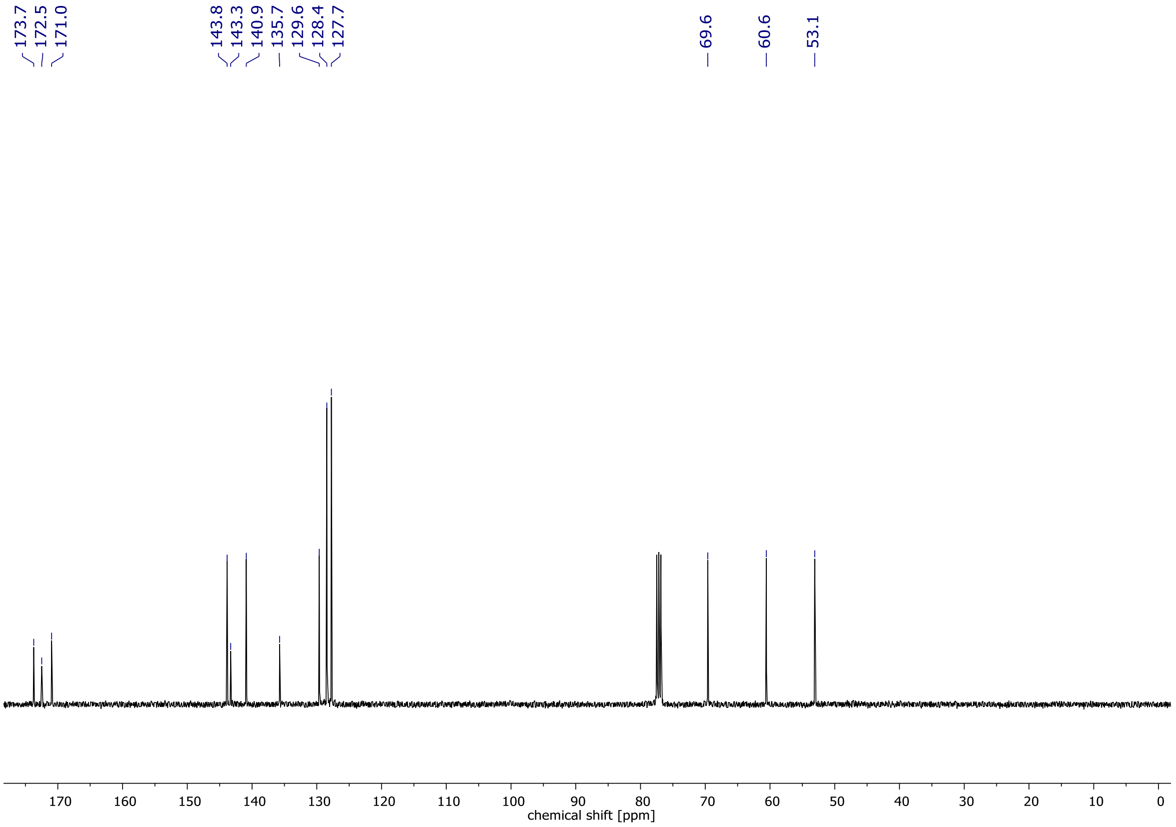

*Figure S 8: ^{13}C NMR Spectrum of **4** measured in $CDCl_3$ (101 MHz).*

Acquisition Parameter

Source Type	APPI	Ion Polarity	Positive	Set Nebulizer	2.0 Bar
Focus	Active			Set Dry Heater	200 °C
Scan Begin	50 m/z	Set Capillary	700 V	Set Dry Gas	2.0 l/min
Scan End	1500 m/z	Set End Plate Offset	-500 V	Set Divert Valve	Waste

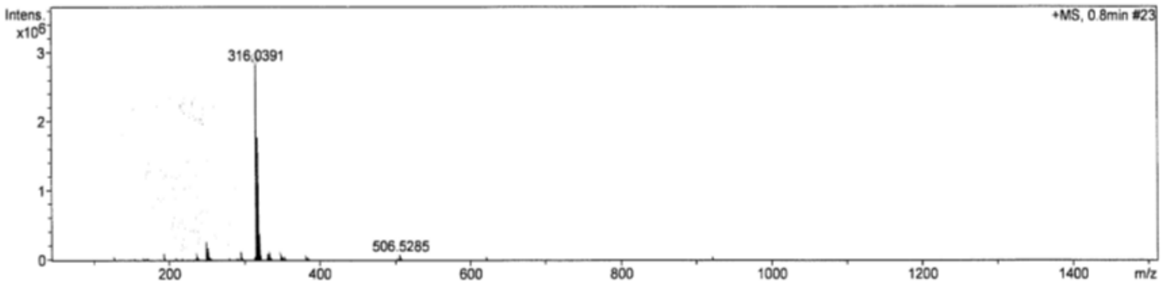

Meas. m/z	#	Ion Formula	m/z	err [ppm]	mSigma	# mSigma	Score	rdb	e¯ Conf	N-Rule
316.0391	1	C16H12Cl2N3	316.0403	3.9	28.8	1	100.00	11.5	even	ok

*Figure S 9: HRMS (APPI) of **4**.*

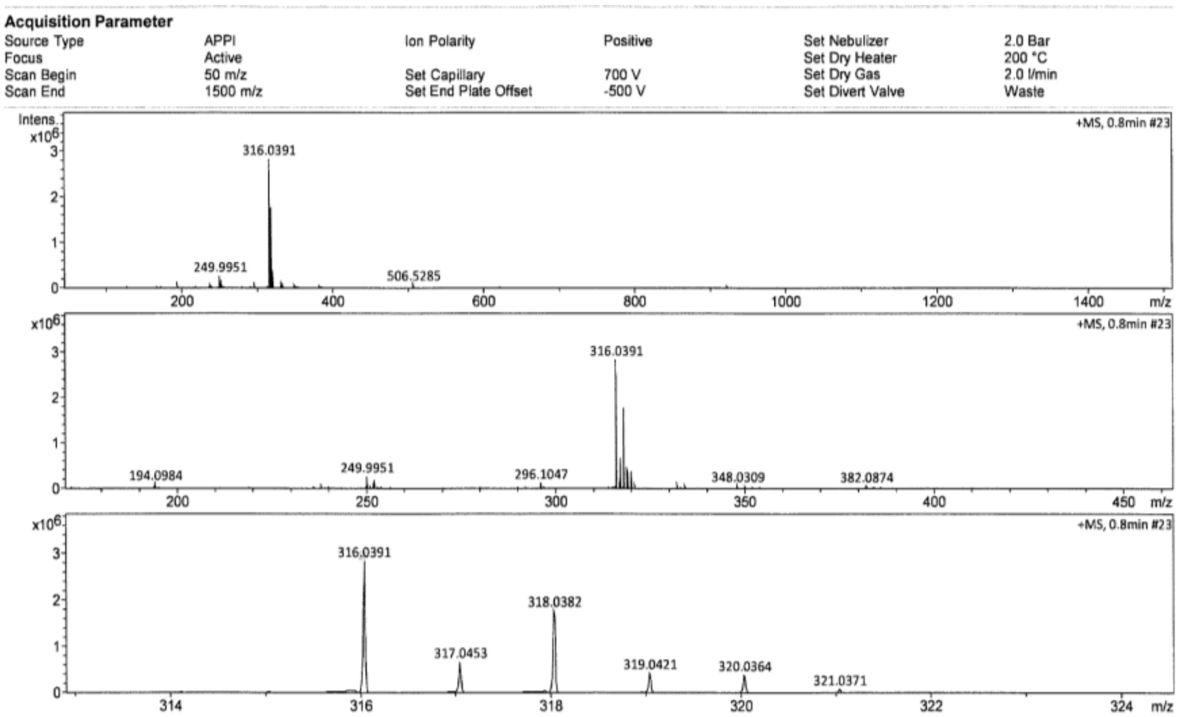

*Figure S 10: Zoom of HRMS (APPI) of **4**.*

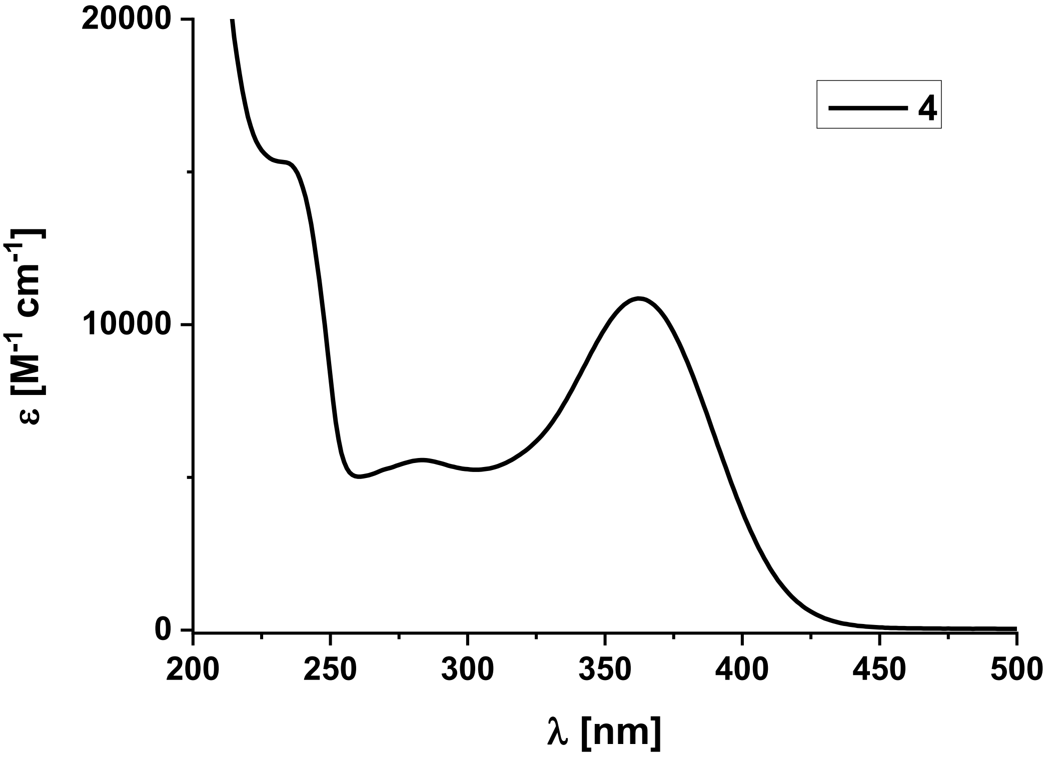

*Figure S 11: UV/Vis extinction spectrum of **4** measured in MeCN.*

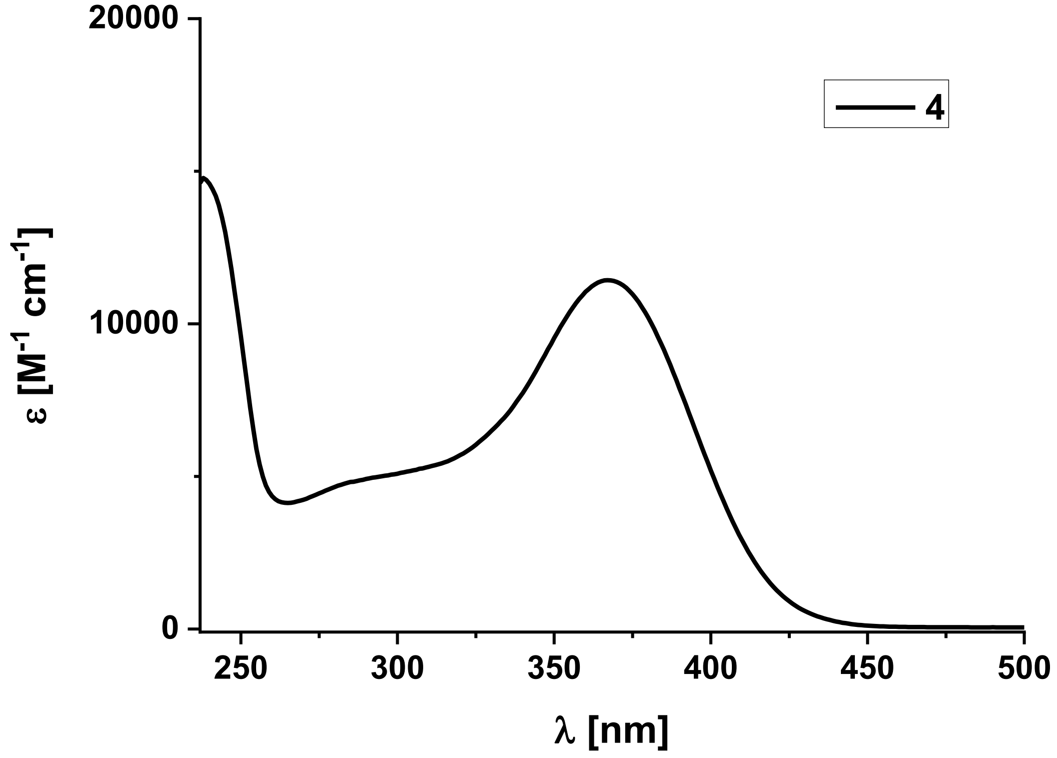

*Figure S 12: UV/Vis extinction spectrum of **4** measured in $CHCl_3$ which is required for the evaluation of the quantum yields.*

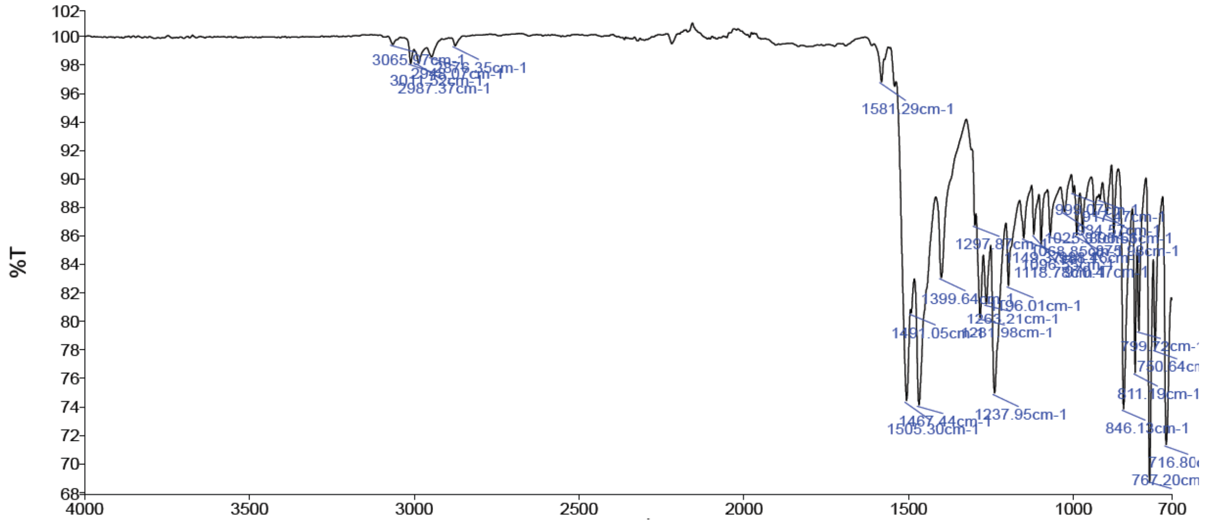

*Figure S 13: ATIR spectrum of **4**.*

3.1.3 **9**

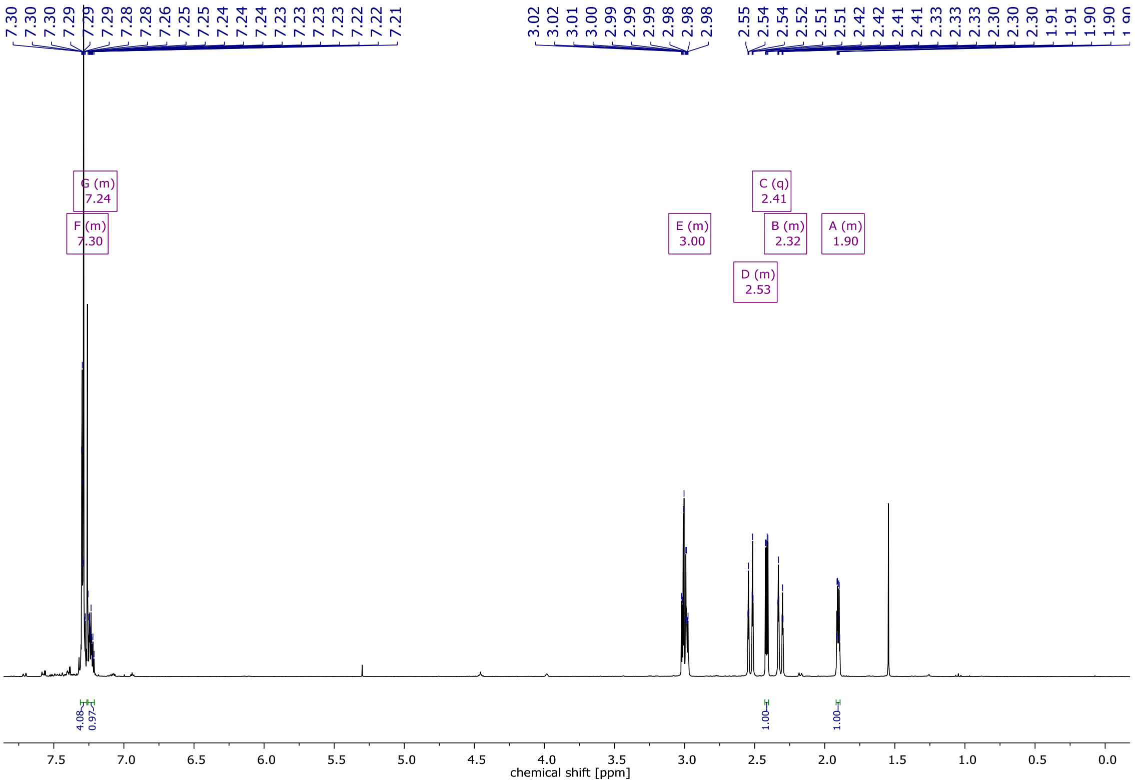

*Figure S 14: ^{1}H NMR spectrum of **9** measured in $CDCl_3$ (400 MHz).*

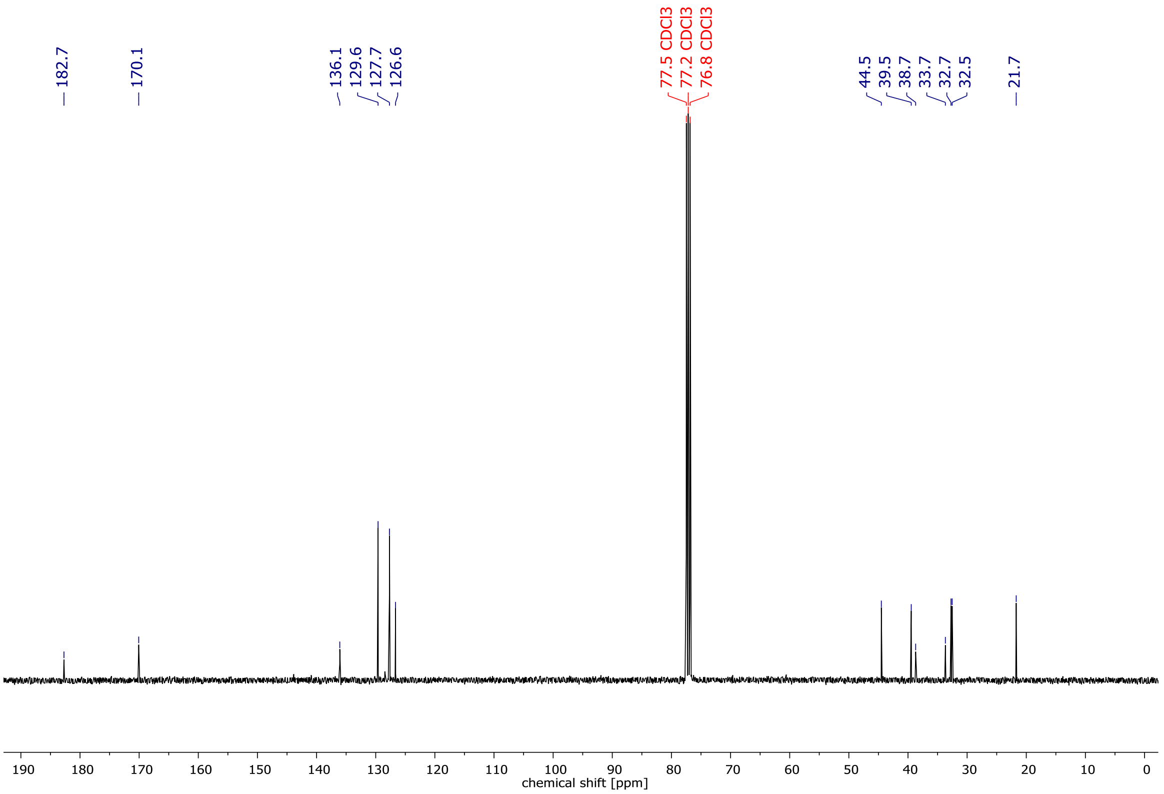

*Figure S 15: ^{13}C NMR Spectrum of **9** measured in $CDCl_3$ (101 MHz).*

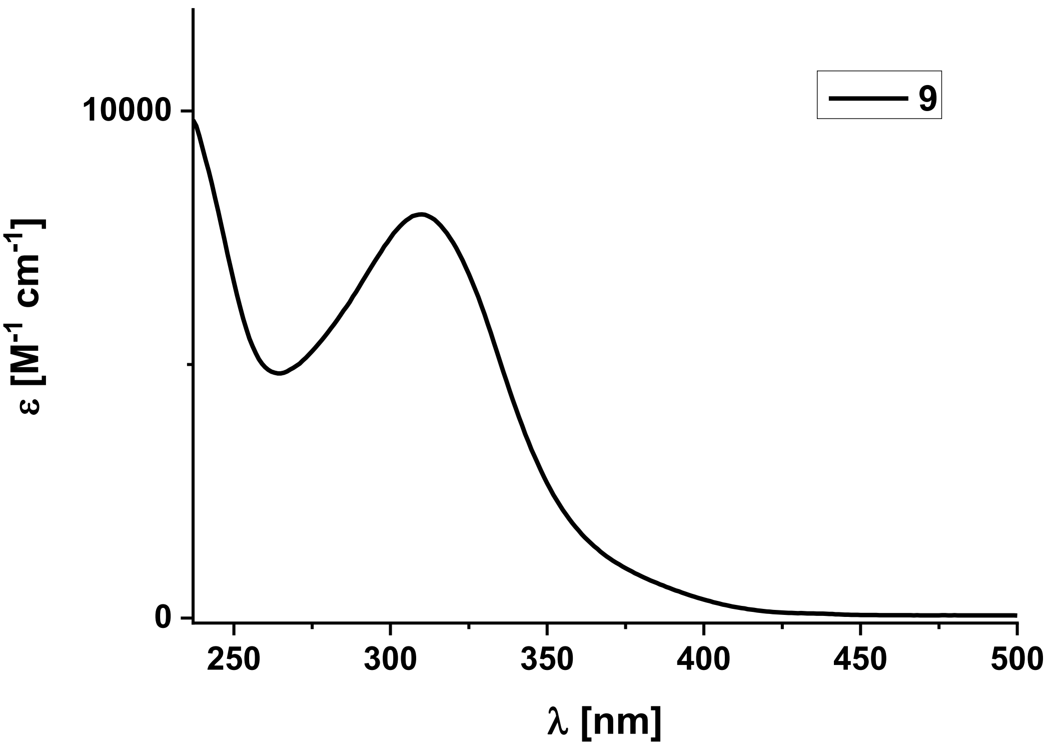

*Figure S 16: UV/Vis extinction spectrum of **4** measured in $CHCl_3$ which is required for the evaluation of the quantum yields.*

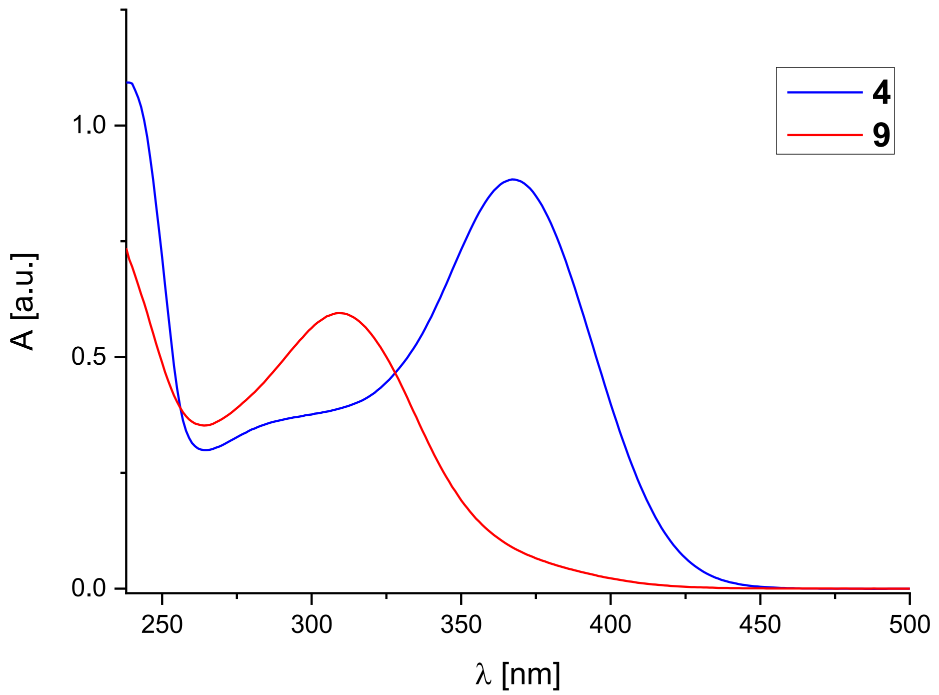

*Figure S 17: UV/Vis spectra of QC **9** and NBD **4** measured in $CHCl_3$.*

3.2 Diphenyl-bis-NBD hybrid

3.2.1 **2a**

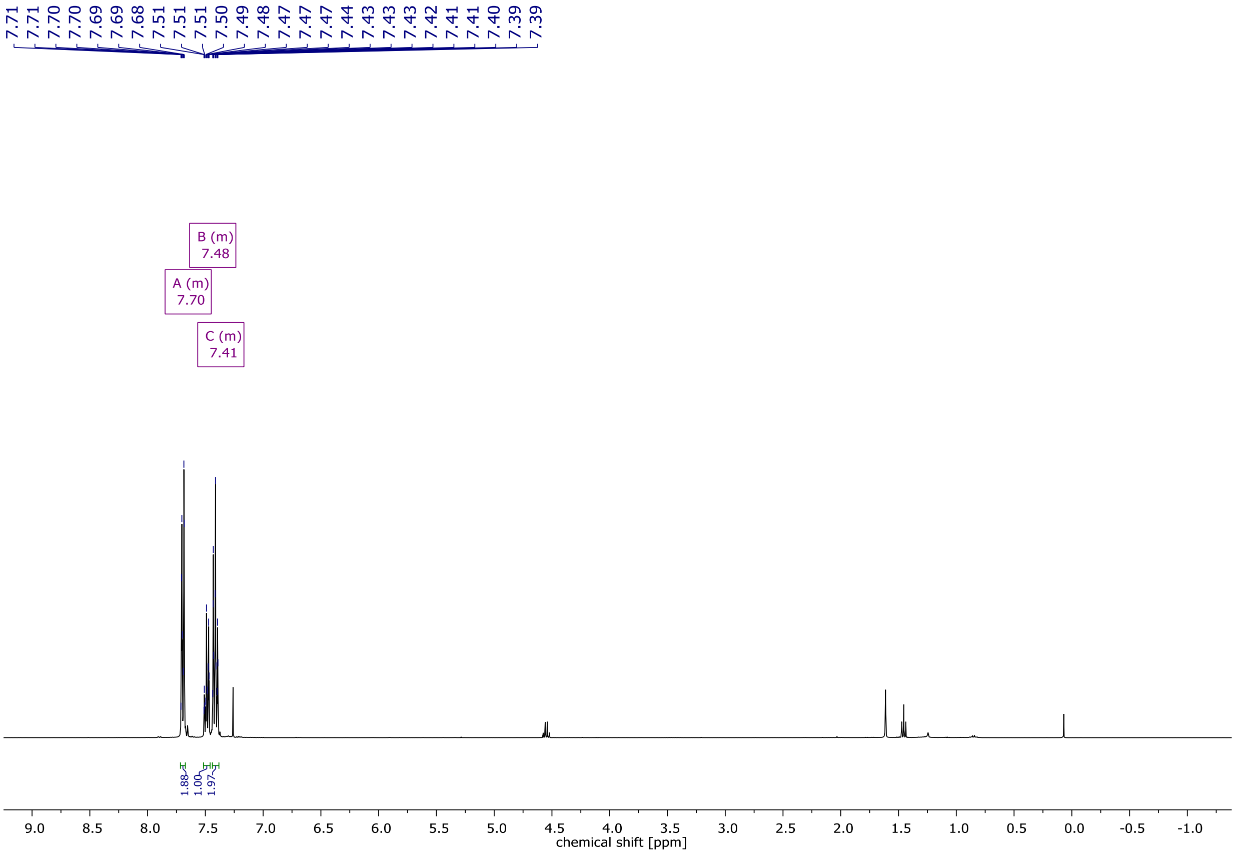

*Figure S 18: ^{1}H NMR Spectrum of **2a** measured in $CDCl_3$ (400 MHz). The leftover impurities belong to ethyl acetate, water and silicon grease.*

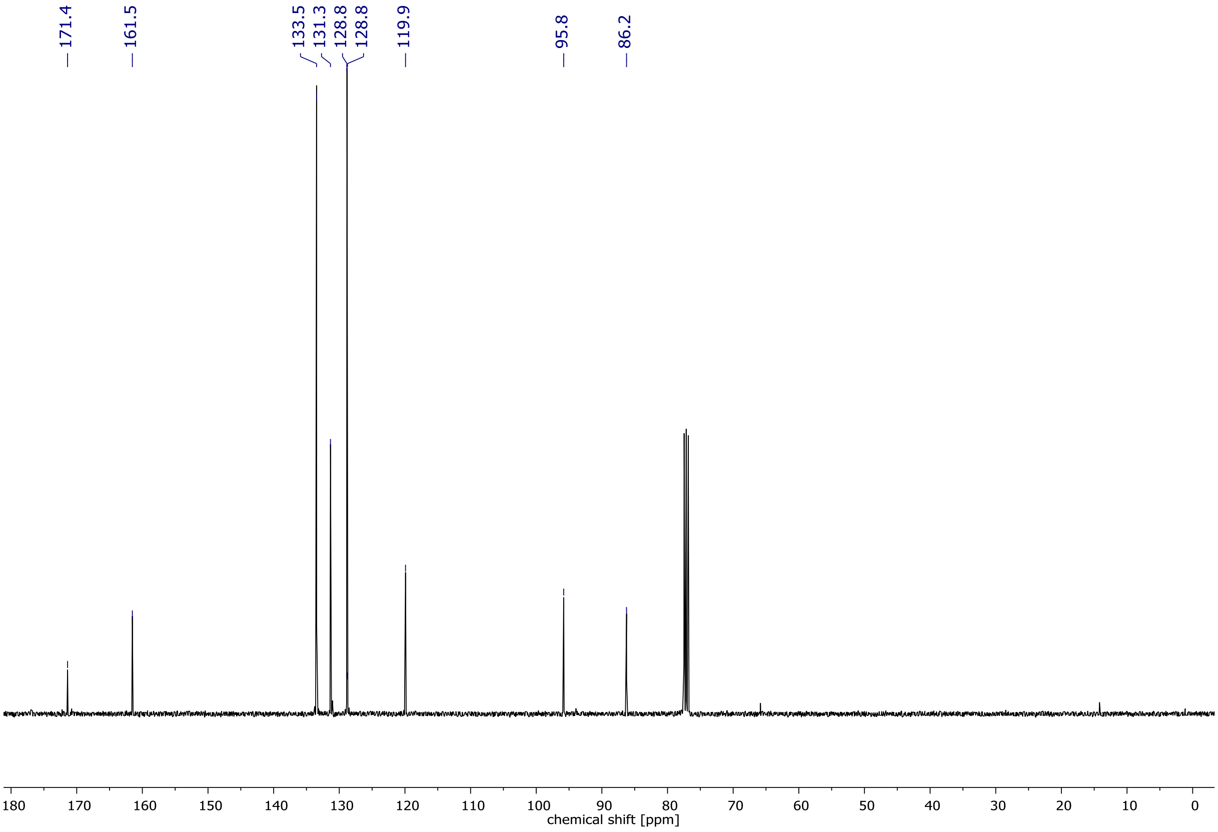

*Figure S 19: ^{13}C NMR Spectrum of **2a** measured in $CDCl_3$ (101 MHz).*

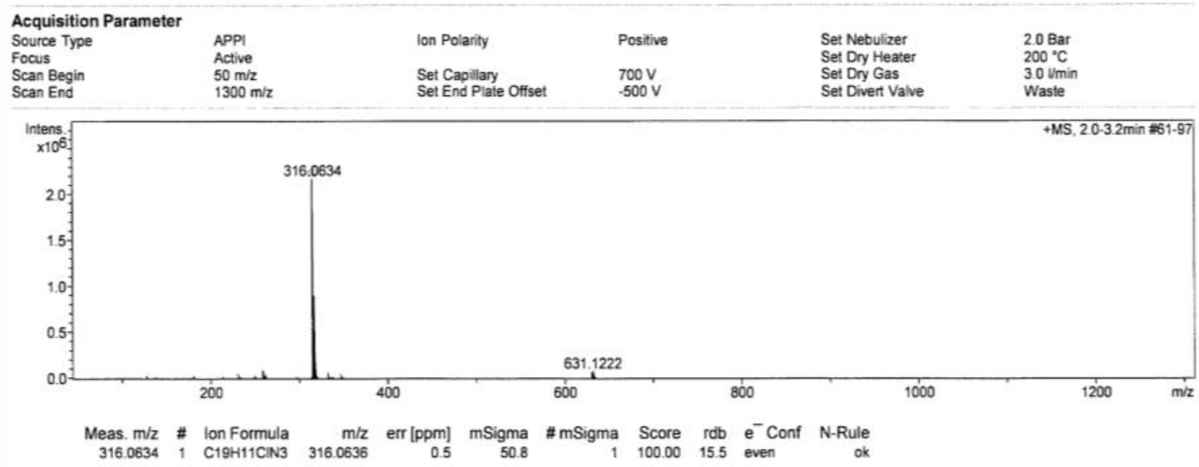

*Figure S 20: HRMS (APPI) of **2a**.*

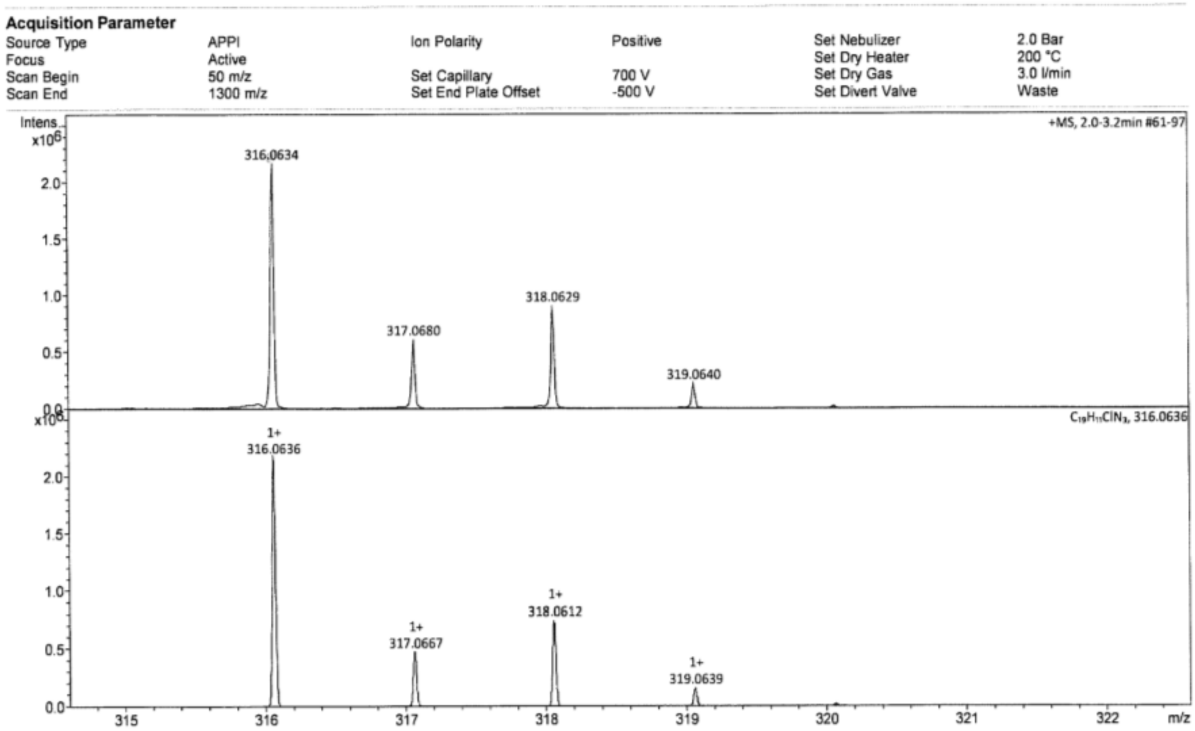

Figure S 21: Zoom of HRMS (APPI) of **2a**.

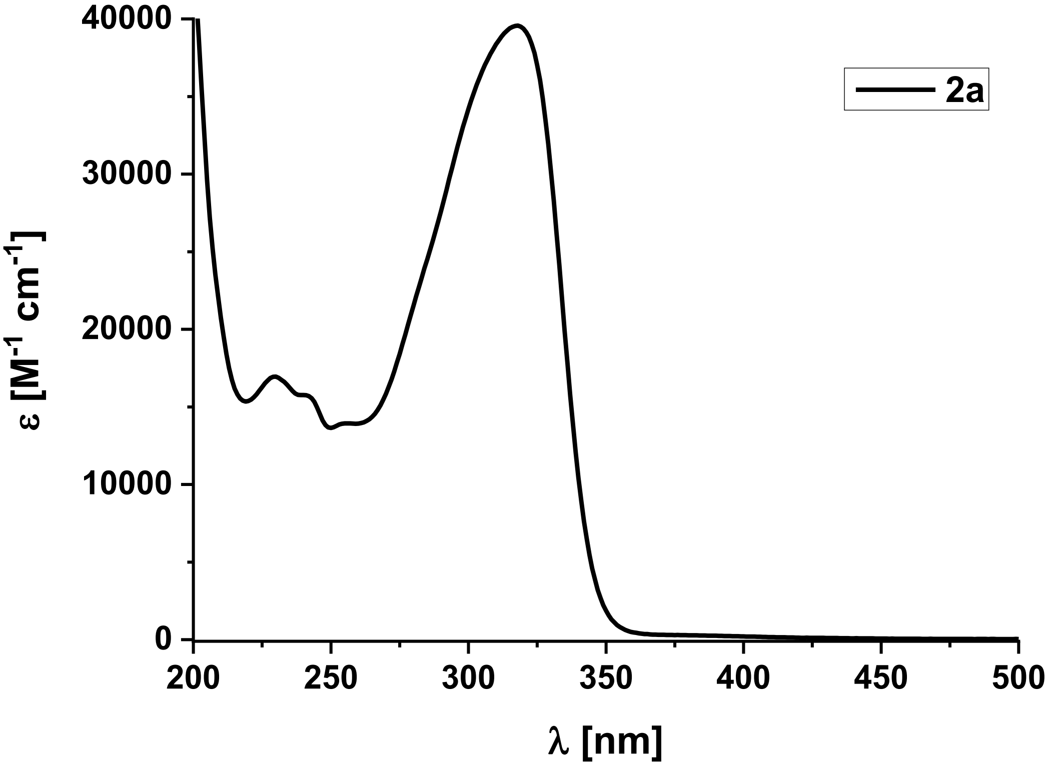

Figure S 22: UV/Vis extinction spectrum of **2a** measured in MeCN.

3.2.2 **5**

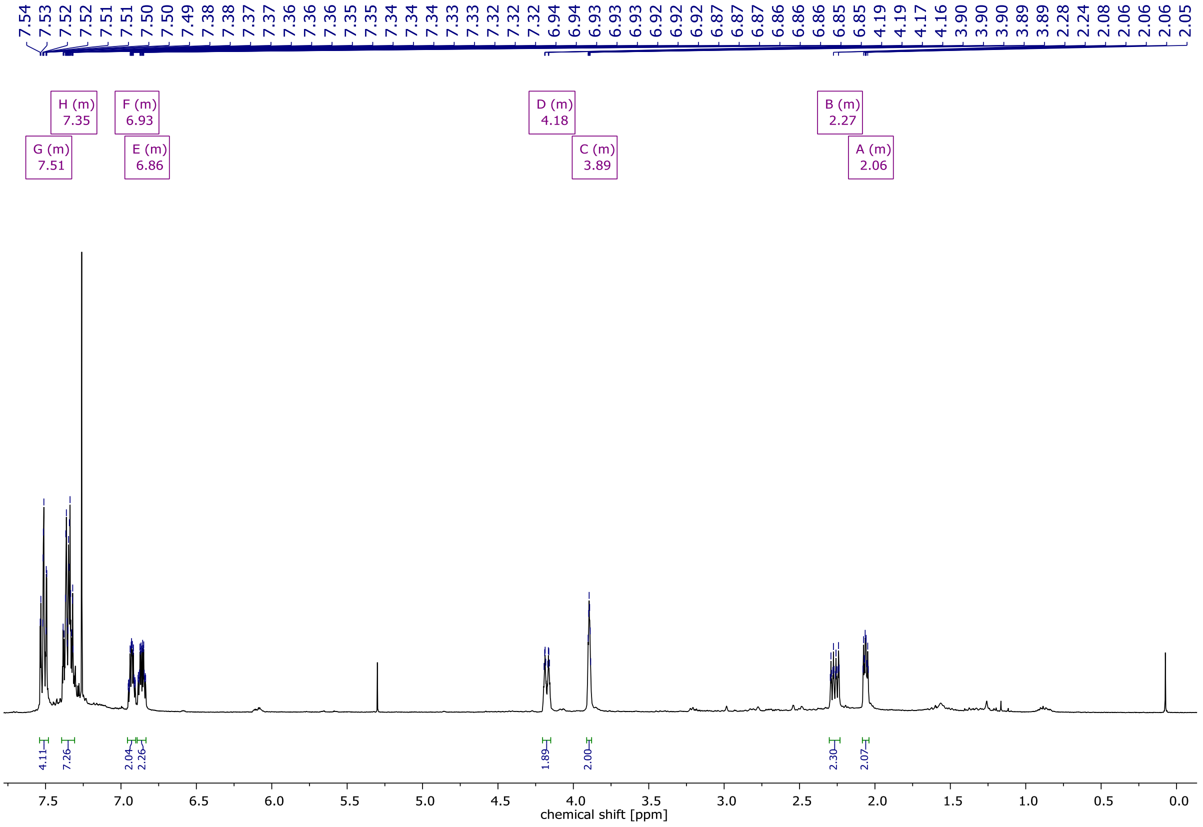

Figure S 23: ^{1}H NMR spectrum of **5** measured in $CDCl_3$ (400 MHz).

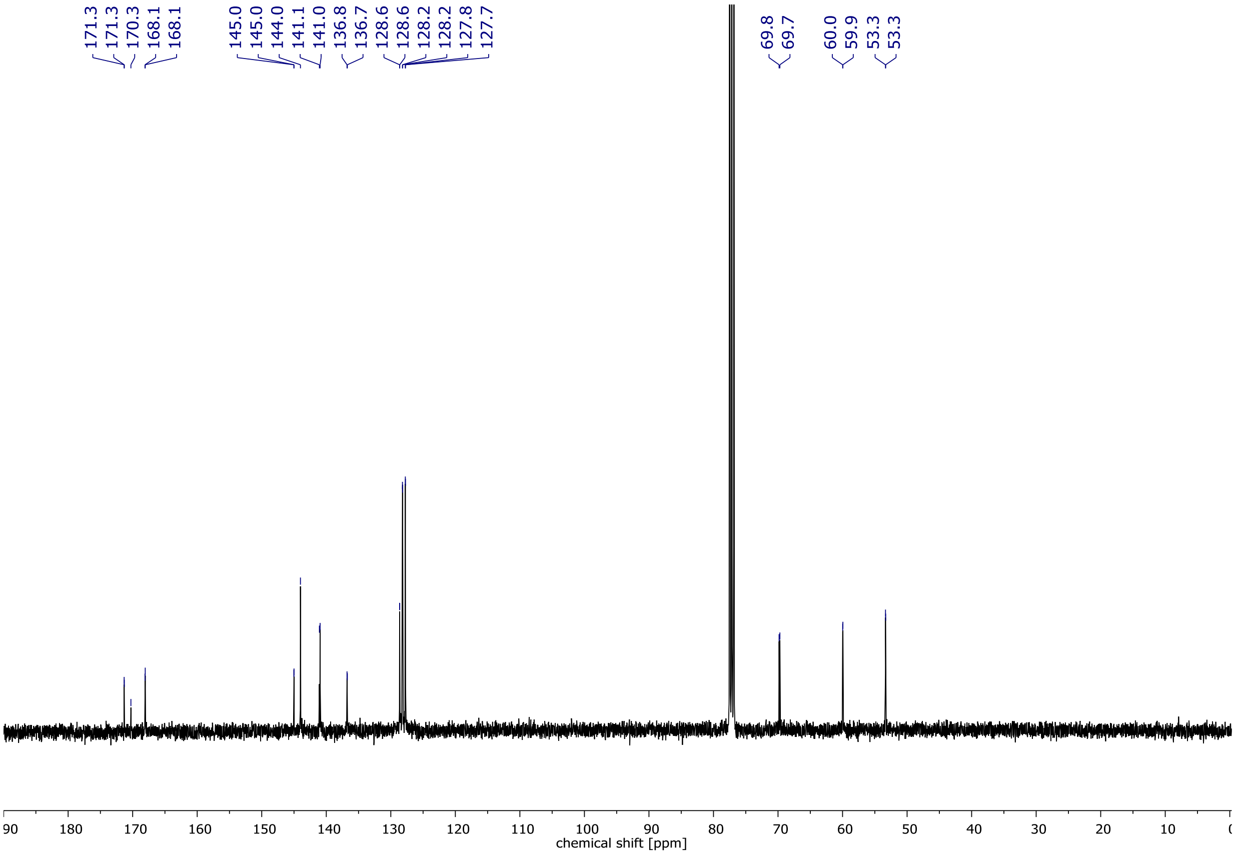

Figure S 24: ^{13}C NMR spectrum of 5 measured in $CDCl_3$ (101 MHz).

Acquisition Parameter

Source Type	APPI	Ion Polarity	Positive	Set Nebulizer	2.0 Bar
Focus	Active			Set Dry Heater	200 °C
Scan Begin	50 m/z	Set Capillary	700 V	Set Dry Gas	2.0 l/min
Scan End	1500 m/z	Set End Plate Offset	-500 V	Set Divert Valve	Waste

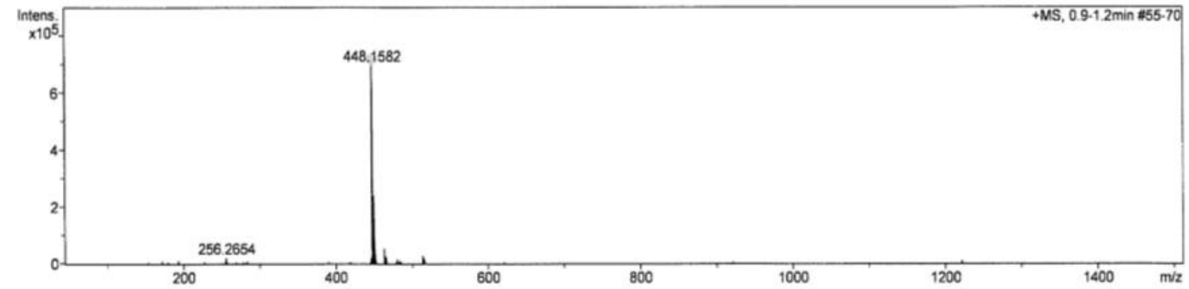

Meas. m/z	#	Ion Formula	m/z	err [ppm]	mSigma	# mSigma	Score	rdb	e⁻ Conf	N-Rule
448.1582	1	C29H23ClN3	448.1575	-1.5	12.3	1	100.00	19.5	even	ok

*Figure S 25: HRMS (APPI) of **5**.*

Acquisition Parameter

Source Type	APPI	Ion Polarity	Positive	Set Nebulizer	2.0 Bar
Focus	Active			Set Dry Heater	200 °C
Scan Begin	50 m/z	Set Capillary	700 V	Set Dry Gas	2.0 l/min
Scan End	1500 m/z	Set End Plate Offset	-500 V	Set Divert Valve	Waste

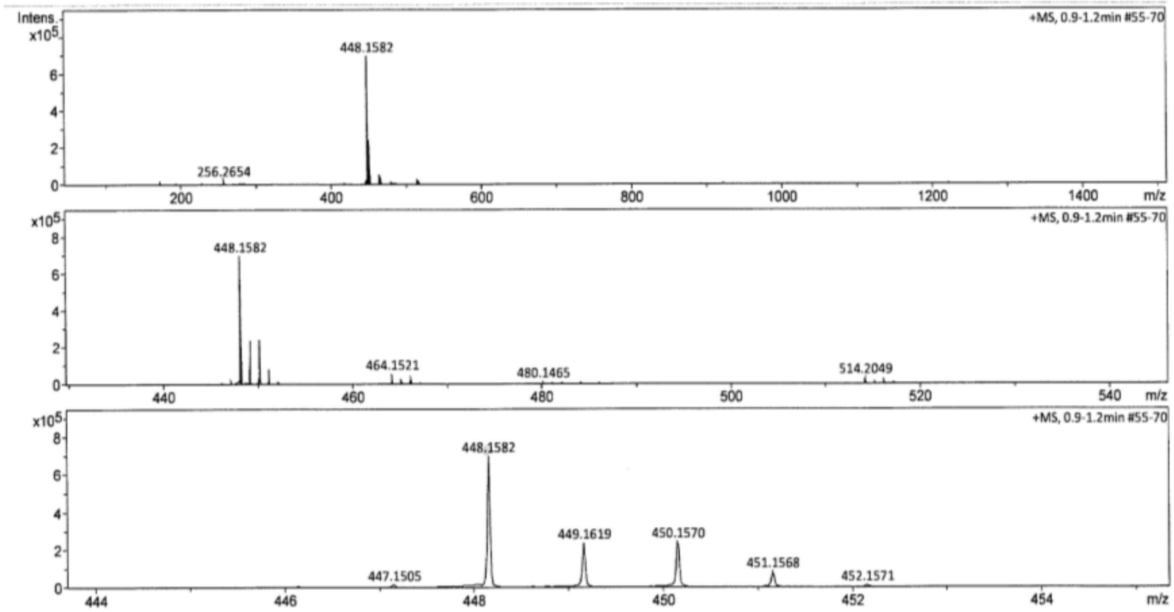

*Figure S 26: Zoom of HRMS (APPI) of **5**.*

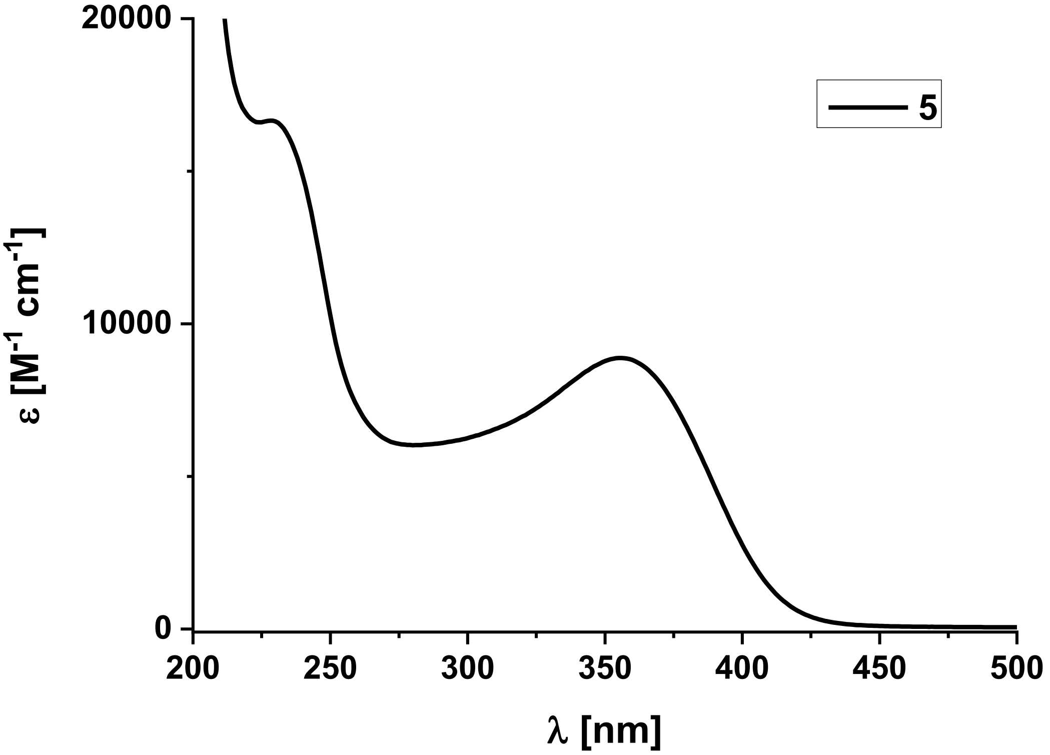

*Figure S 27: UV/Vis extinction spectrum of **5** measured in MeCN.*

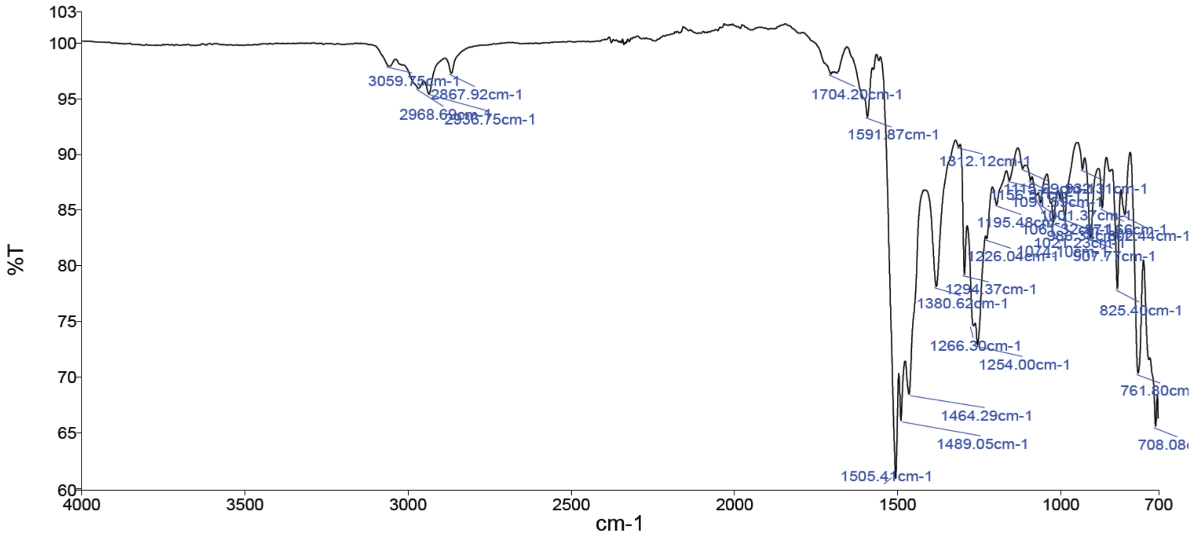

*Figure S 28: ATIR spectrum of **5**.*

3.2.3 **10**

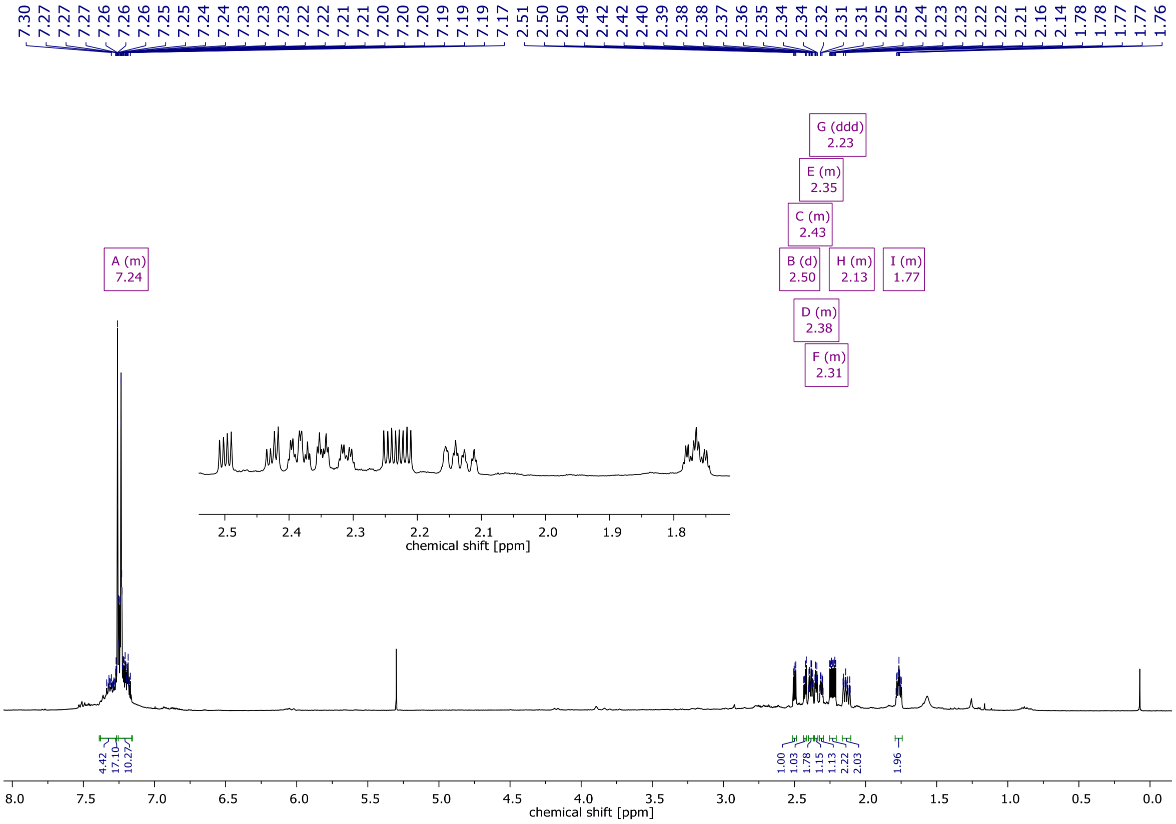

*Figure S 29: ^{1}H NMR spectrum of **10** measured in $CDCl_3$ (400 MHz) including a zoom-in of the significant aliphatic section.*

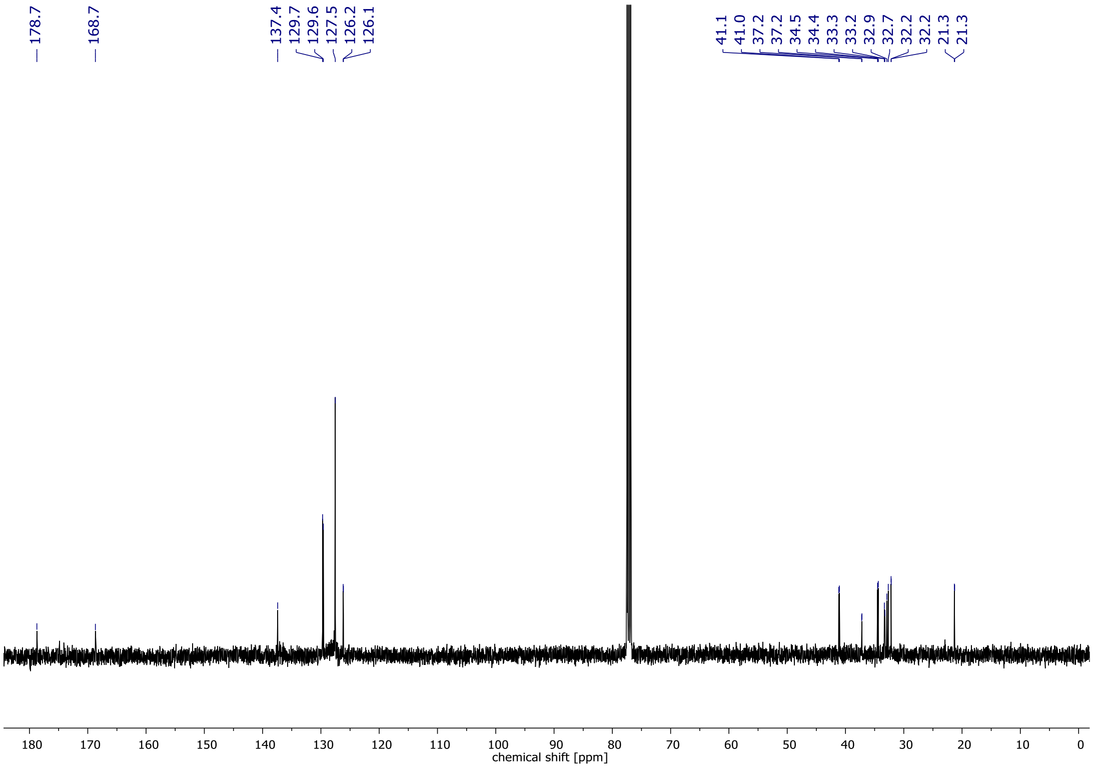

*Figure S 30: ^{13}C NMR Spectrum of **10** measured in $CDCl_3$ (101 MHz).*

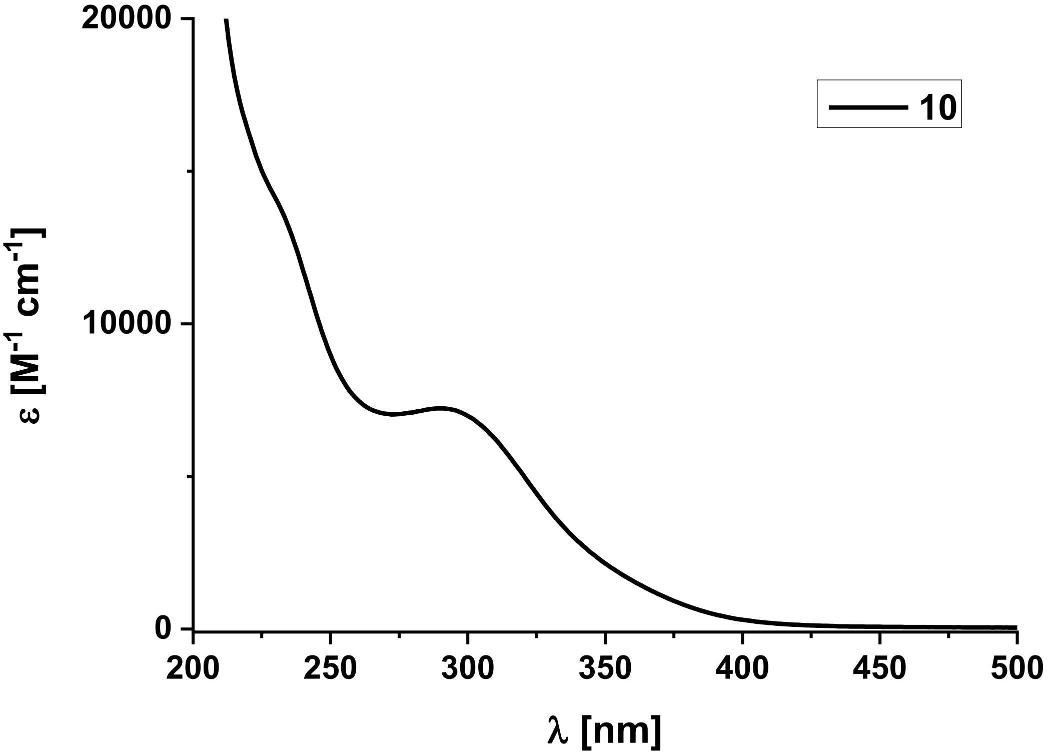

*Figure S 31: UV/Vis extinction spectrum of **10** measured in MeCN.*

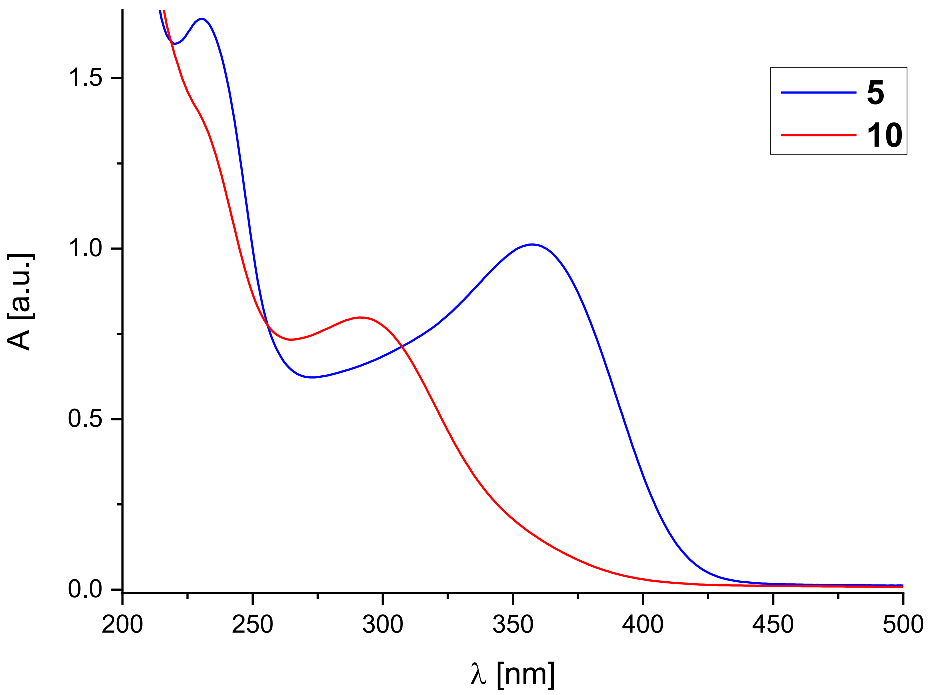

*Figure S 32: UV/Vis spectra of QC **10** and NBD **5** measured in MeCN.*

3.3 Dimethylaniline-mono-NBD hybrid

3.3.1 **1c**

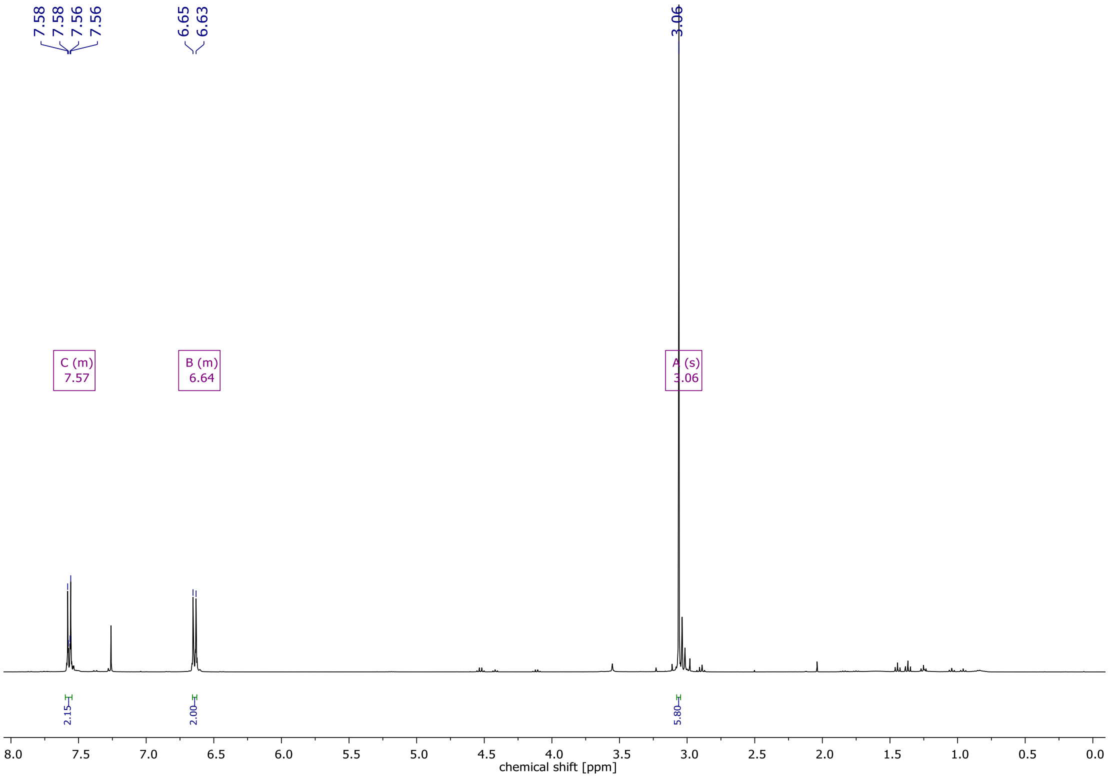

*Figure S 33: ^{1}H NMR spectrum of **1c** measured in $CDCl_3$ (400 MHz).*

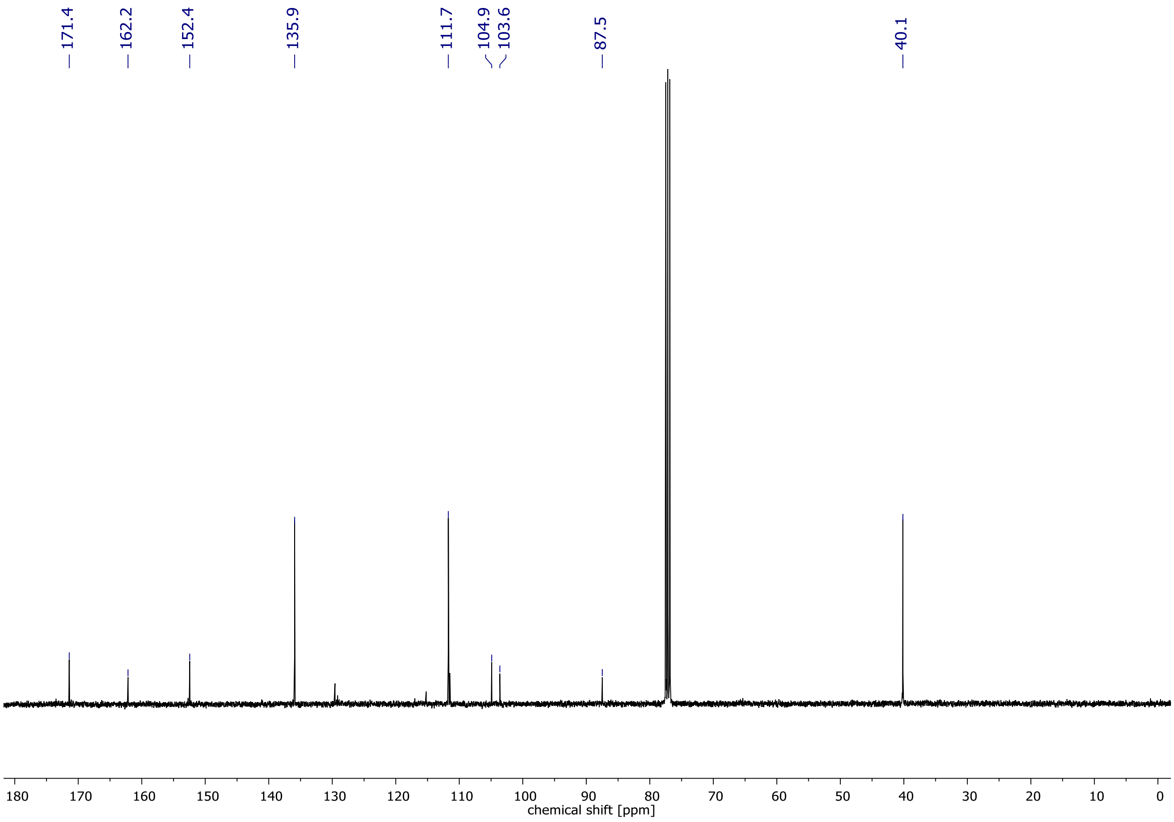

*Figure S 34: ^{13}C NMR Spectrum of **6** measured in $CDCl_3$ (101 MHz).*

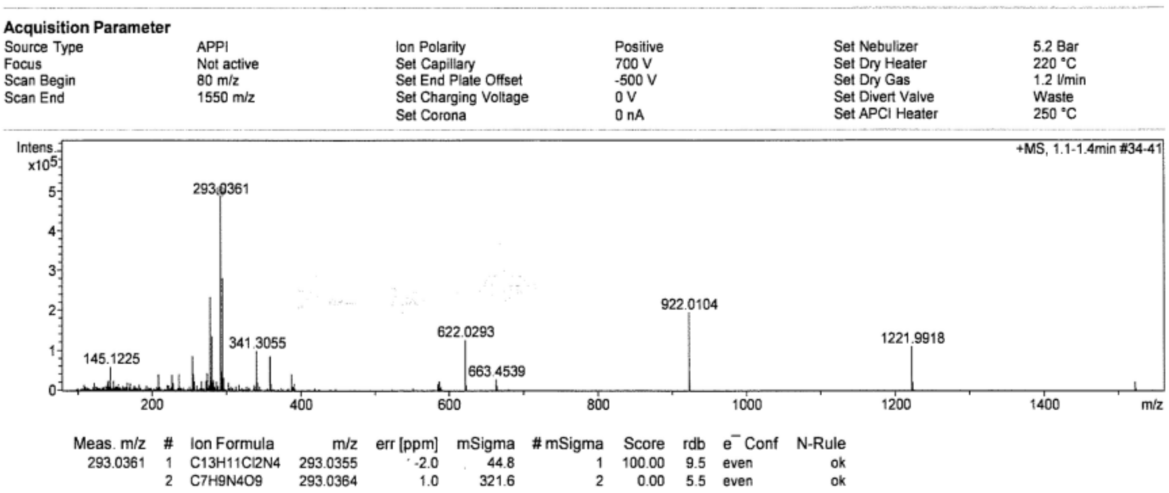

*Figure S 35: HRMS (APPI) of **6**. The additional signals at 622, 922 and 1221 m/z are impurities which were found during all mass experiments measured during the time of the synthesis of the molecules.*

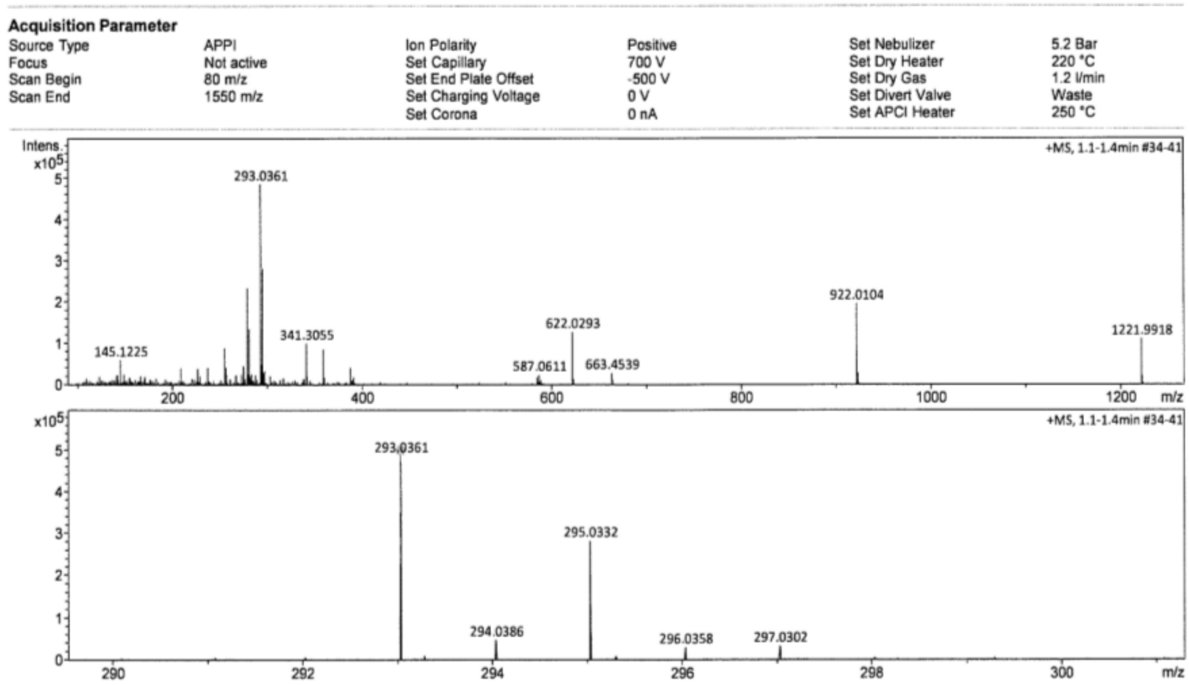

*Figure S 36: Zoom of HRMS (APPI) of **6**. The additional signals at 622, 922 and 1221 m/z are impurities which were found during all mass experiments measured during the time of the synthesis of the molecules.*

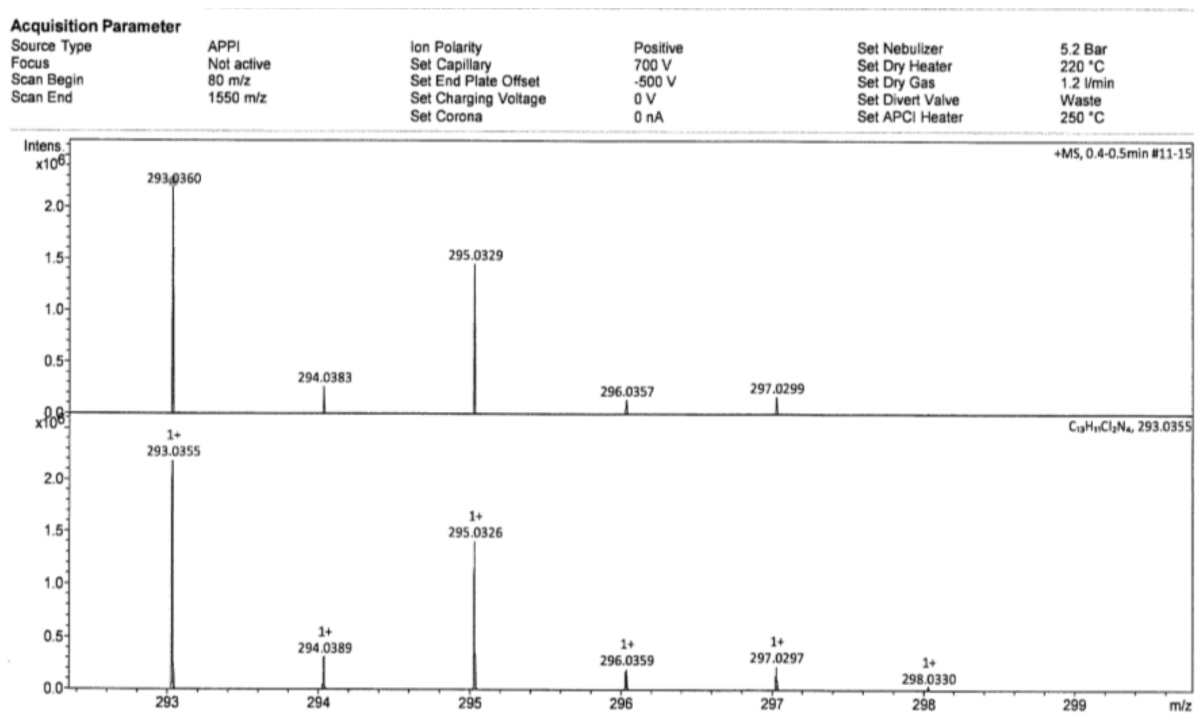

*Figure S 37: Zoom of the HRMS (APPI) of an additional sample of **6** for better comparison of the measured and calculated spectra.*

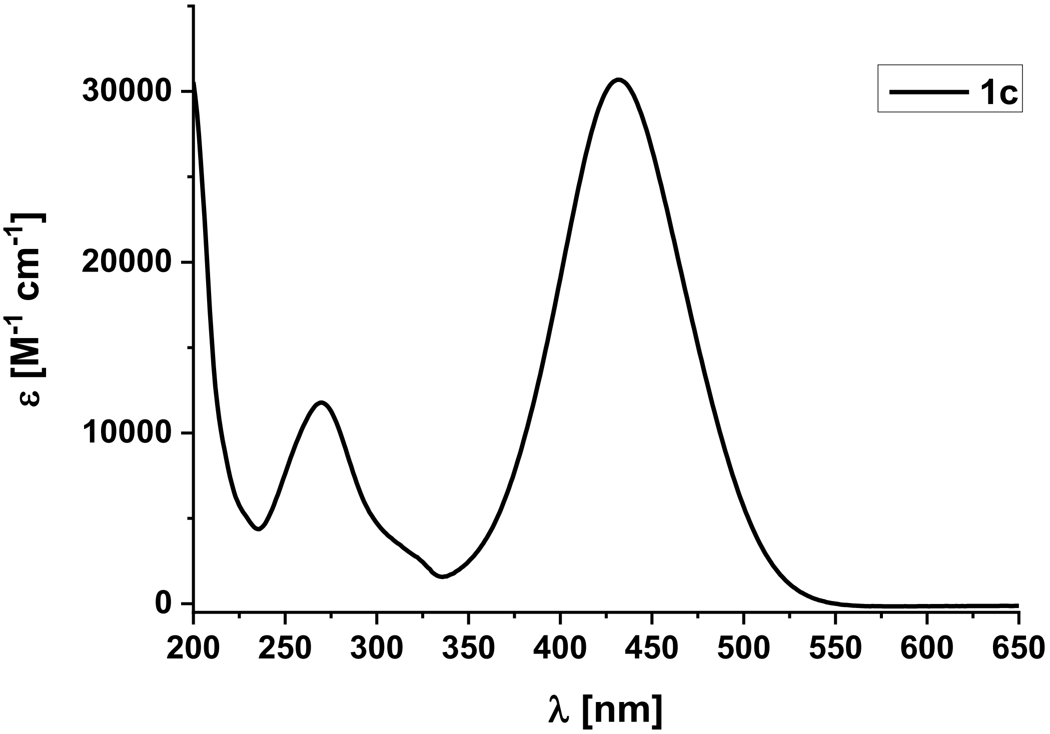

*Figure S 38: UV/Vis extinction spectrum of **1c** measured in MeCN.*

3.3.2 **6**

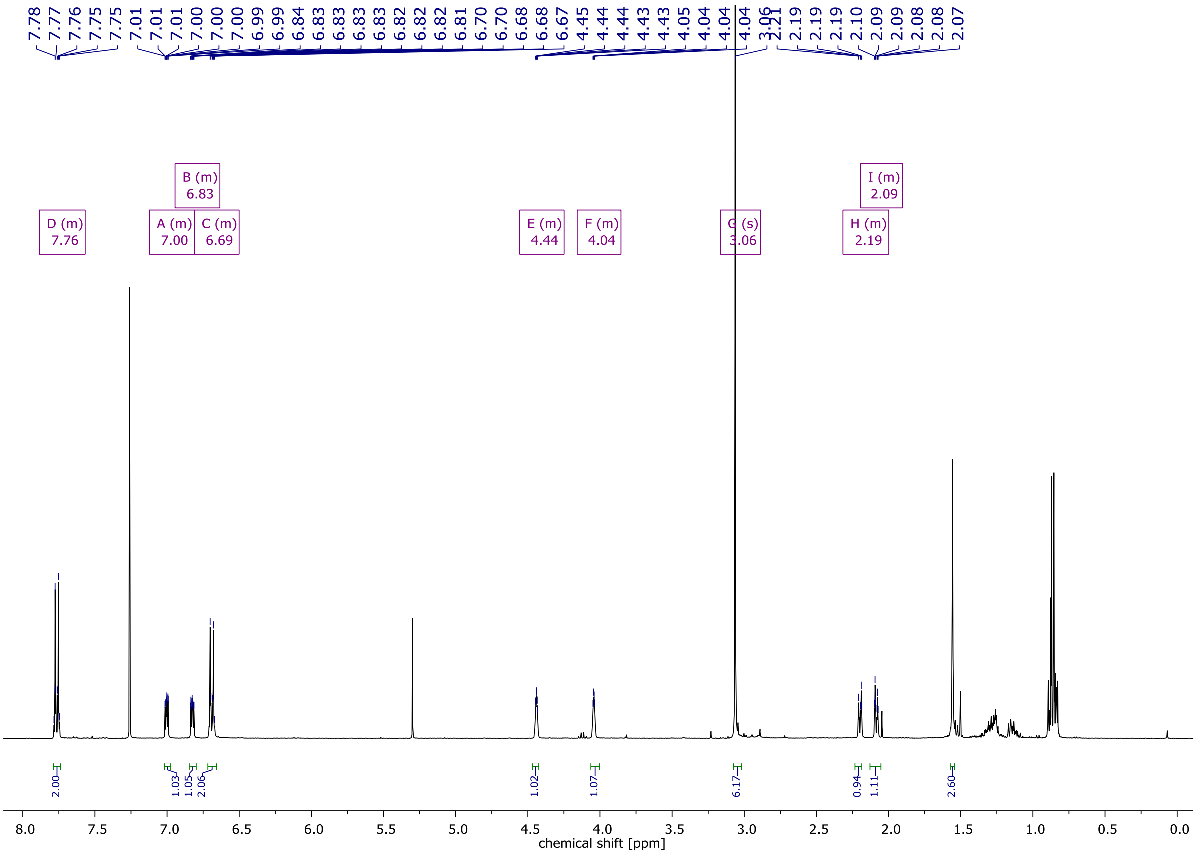

*Figure S 39: ^{1}H NMR spectrum of **6** measured in $CDCl_3$ (400 MHz). The impurity signals in the aliphatic region below 1.5 ppm belong to hexanes and H grease.*

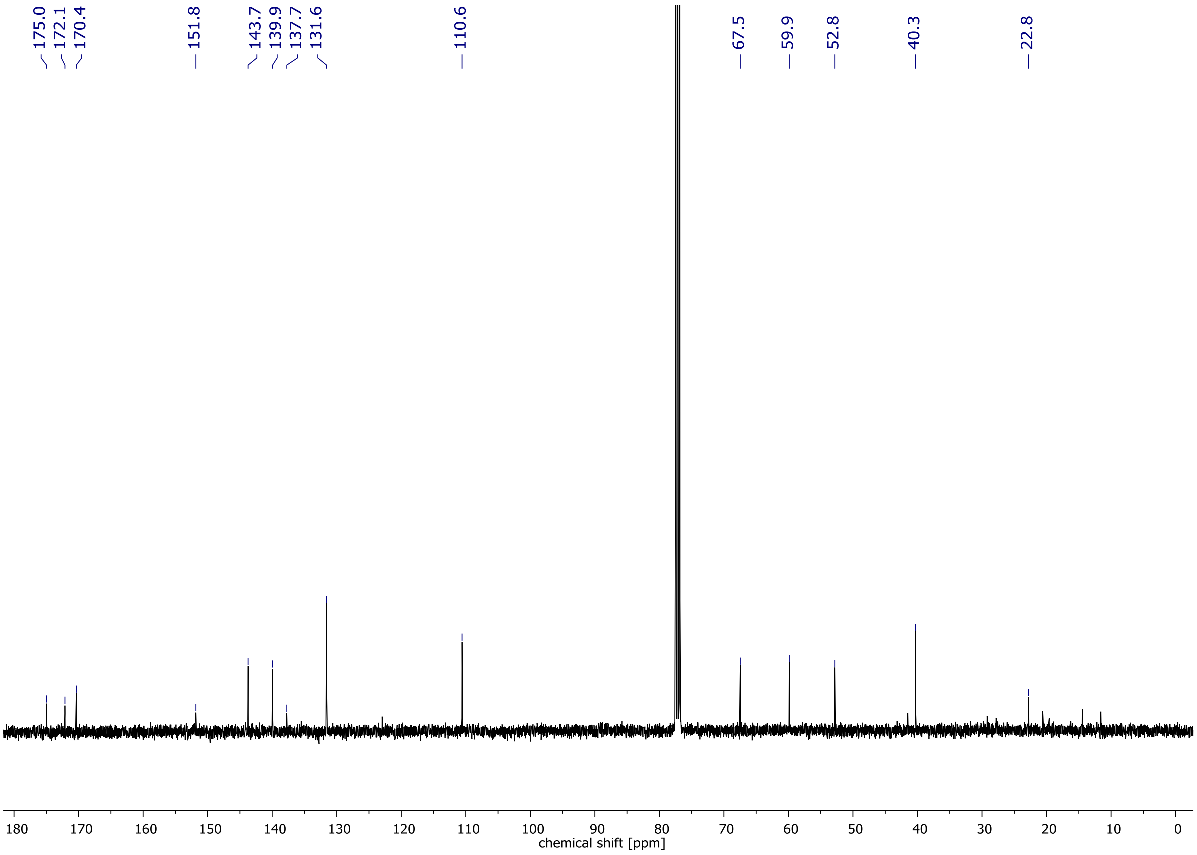

*Figure S 40: ^{13}C NMR Spectrum of **6** measured in $CDCl_3$ (101 MHz). Leftover signals in the aliphatic region belonging to unidentified impurities can be found.*

Acquisition Parameter

Source Type	APPI	Ion Polarity	Positive	Set Nebulizer	5.2 Bar
Focus	Not active	Set Capillary	700 V	Set Dry Heater	220 °C
Scan Begin	80 m/z	Set End Plate Offset	-500 V	Set Dry Gas	1.2 l/min
Scan End	1550 m/z	Set Charging Voltage	0 V	Set Divert Valve	Waste
		Set Corona	0 nA	Set APCI Heater	250 °C

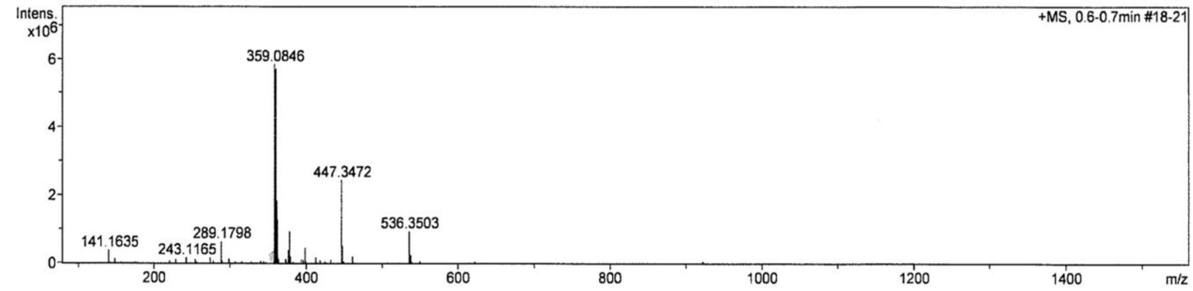

Meas. m/z	#	Ion Formula	m/z	err [ppm]	mSigma	# mSigma	Score	rdb	e⁻ Conf	N-Rule
358.0742	1	C18H16Cl2N4	358.0747	1.2	683.3	1	100.00	12.0	odd	ok

*Figure S 41: HRMS (APPI) of **6**.*

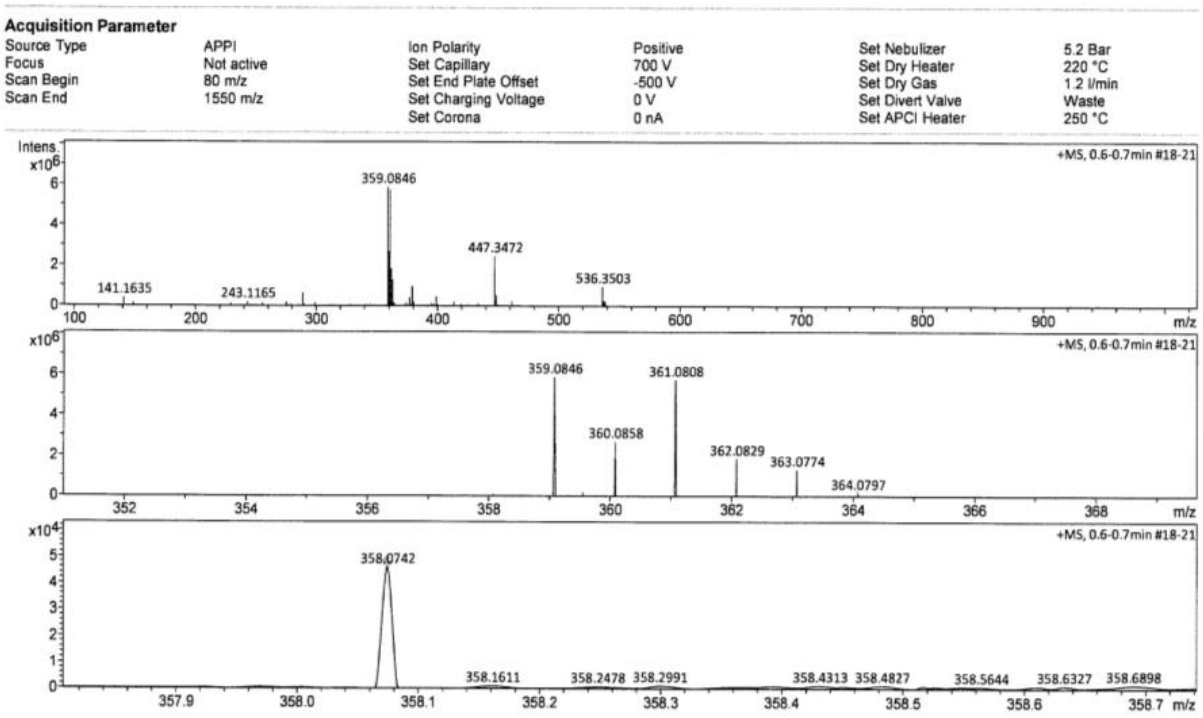

*Figure S 42: Zoom of HRMS (APPI) of **6**.*

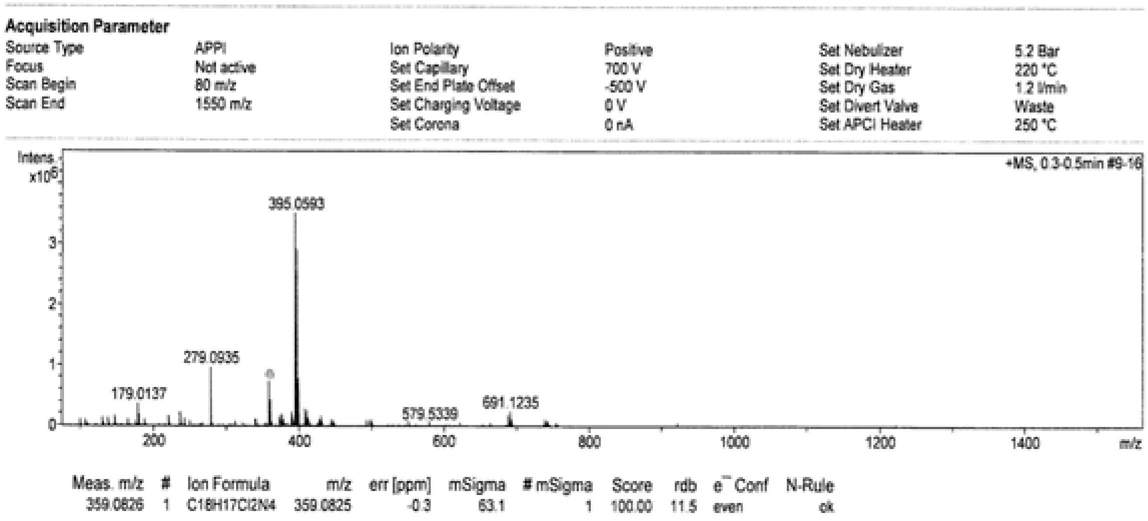

*Figure S 43: Second HRMS (APPI) measurement with the same sample of **6** including calculation for[M+H]⁺.*

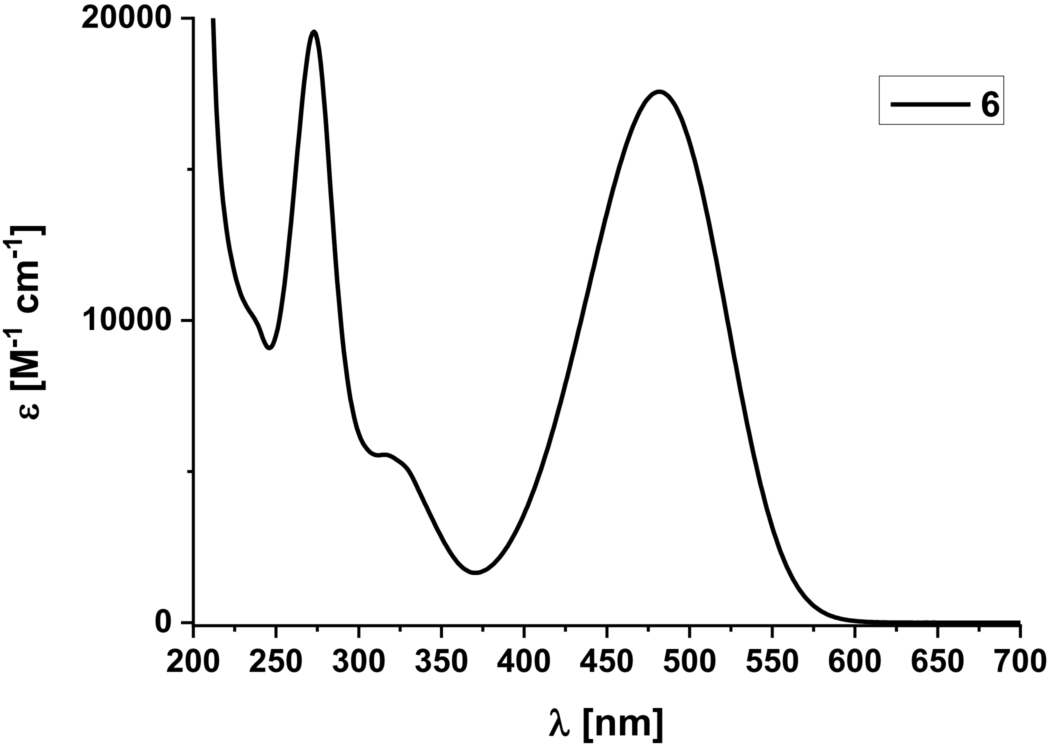

*Figure S 44: UV/Vis extinction spectrum of **6** measured in MeCN.*

3.3.3 **11**

Since clean switching was unsuccessful, proper characterization could not be conducted. However, as mentioned in the synthesis part, minor potential dimerization was found in mass spectrometry, as described below (section 4 - NMR Switching). The switching sections will also provide further discussion and analysis of the ongoing photo-induced conversions.

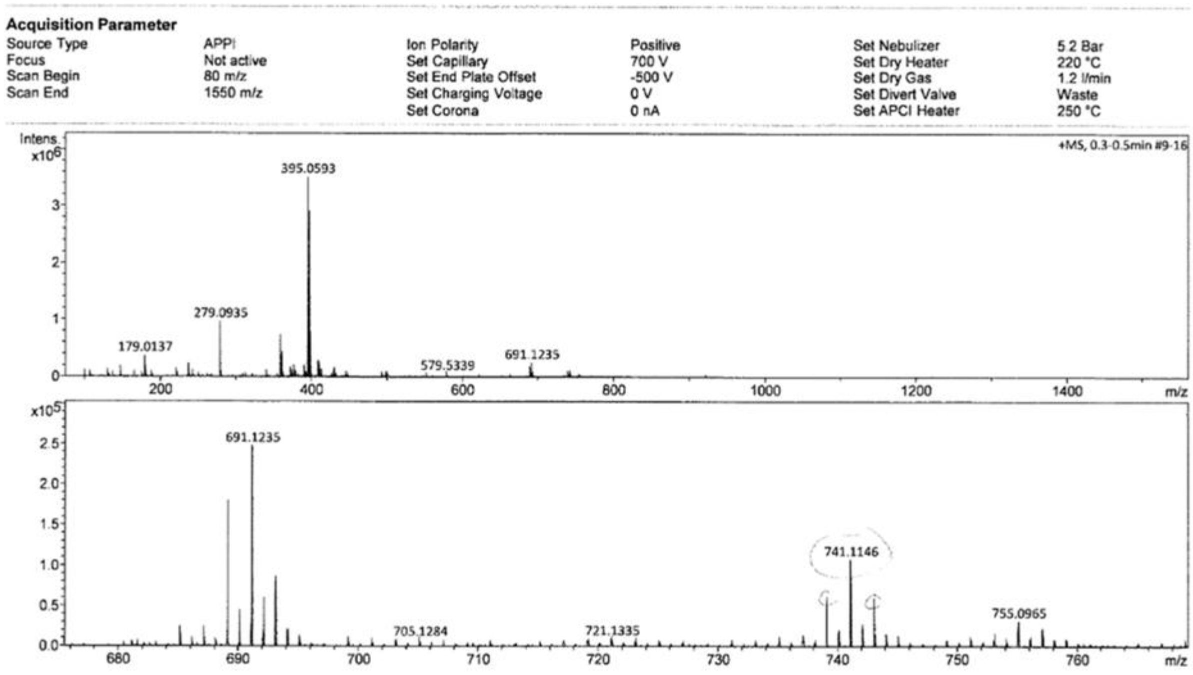

*Figure S 45: HRMS (APPI) of **6/11** after irradiation. Calced. for $C_{36}H_{32}Cl_4N_8Na^+$: 741.1367, found 741.1146 $[2M+Na]^+$.*

3.4 Dimethylaniline-phenyl-bis-NBD hybrid

3.4.1 **2b**

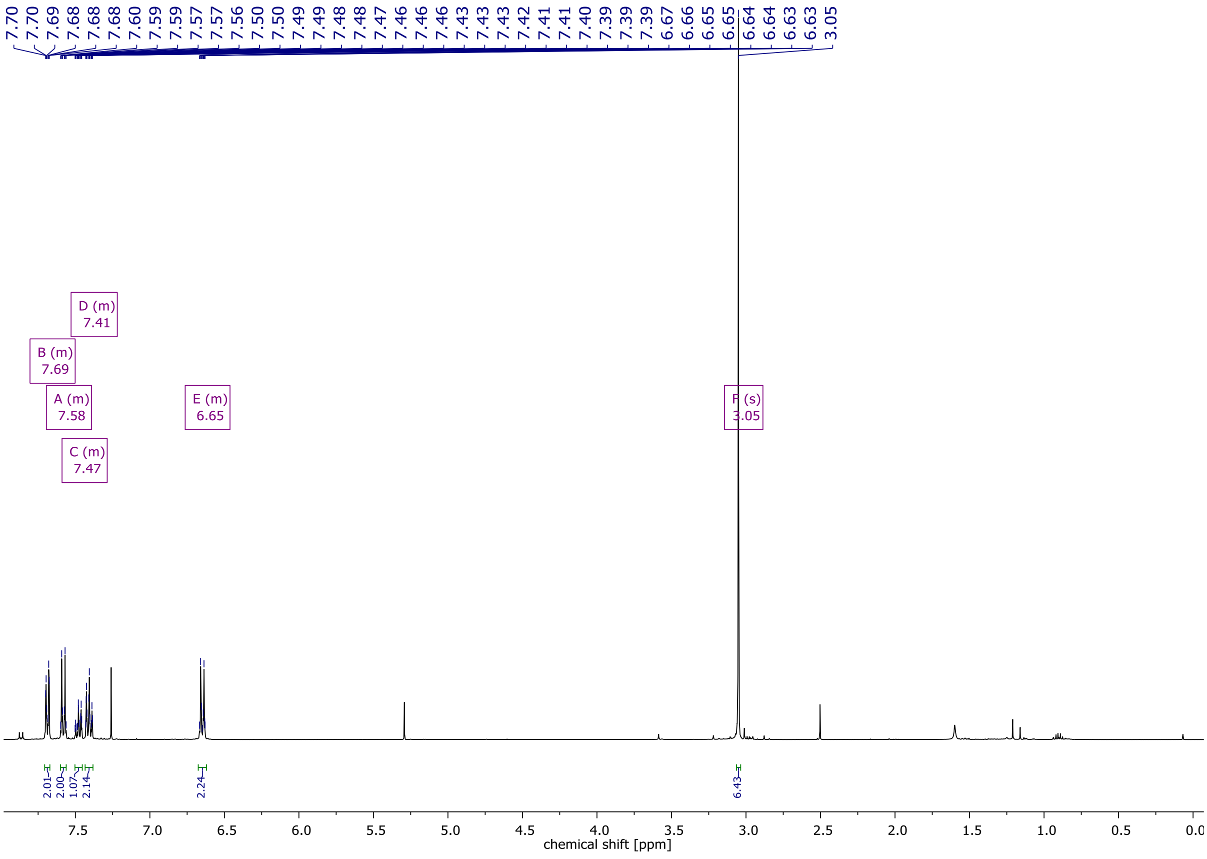

*Figure S 46: ^1H NMR spectrum of **2b** measured in $CDCl_3$ (400 MHz).*

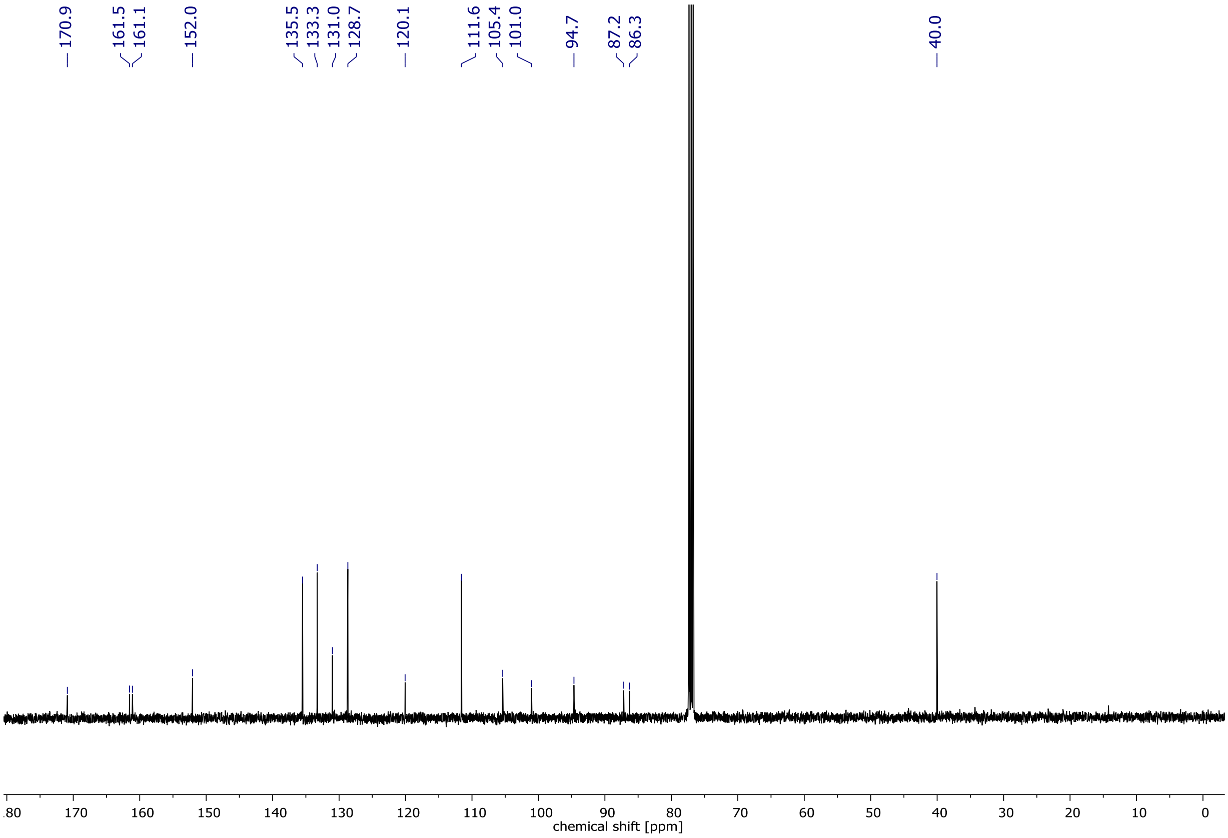

*Figure S 47: ^{13}C NMR Spectrum of **2b** measured in $CDCl_3$ (101 MHz).*

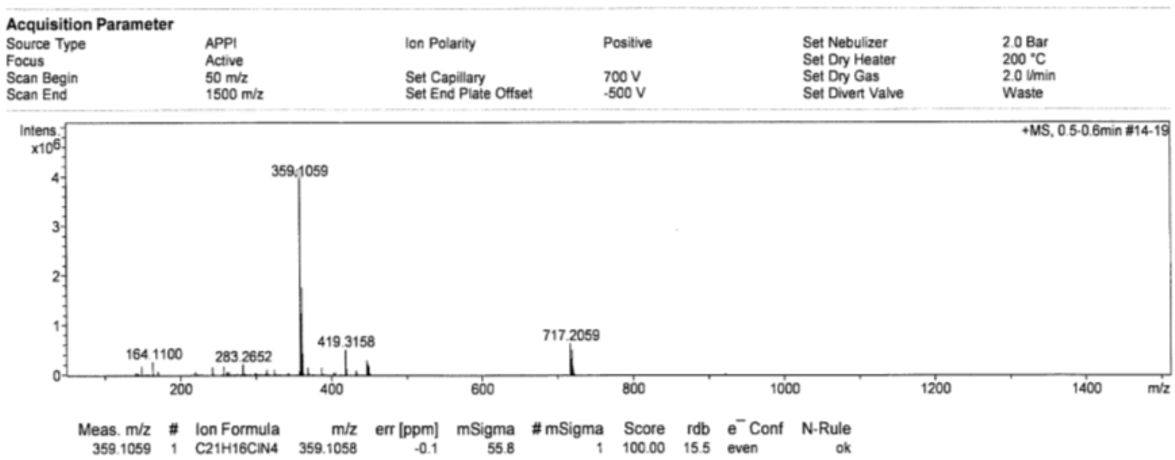

*Figure S 48: HRMS (APPI) of **2b.***

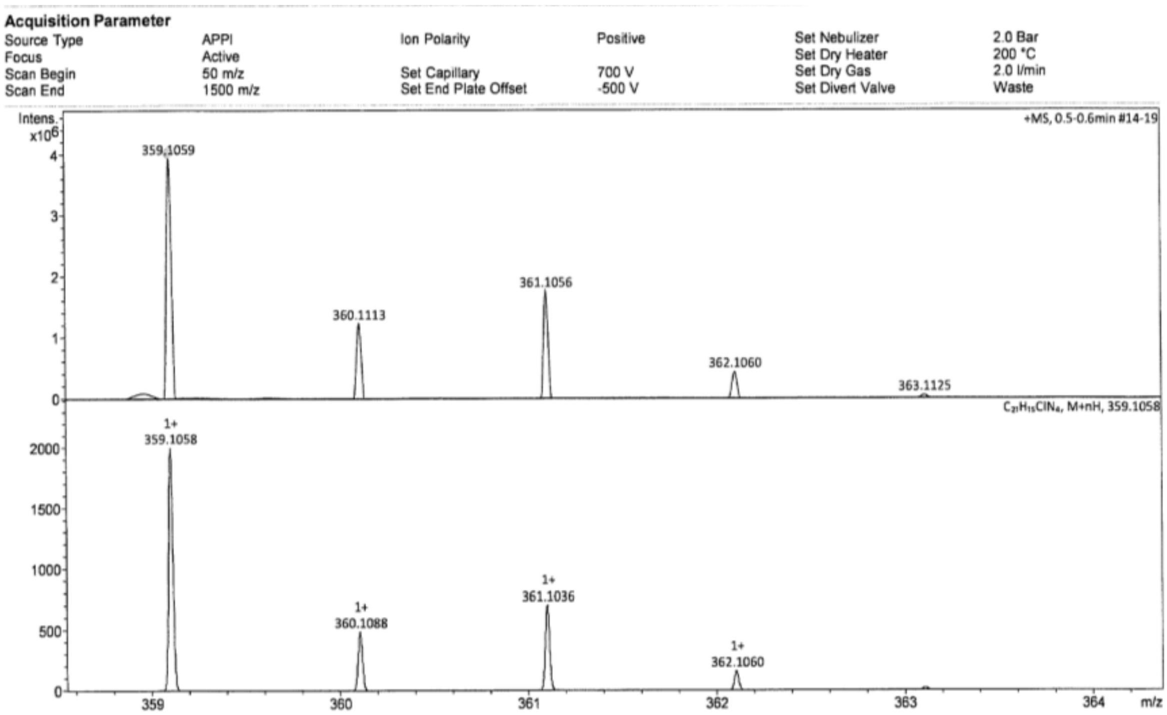

*Figure S 49: Zoom of HRMS (APPI) of **2b**.*

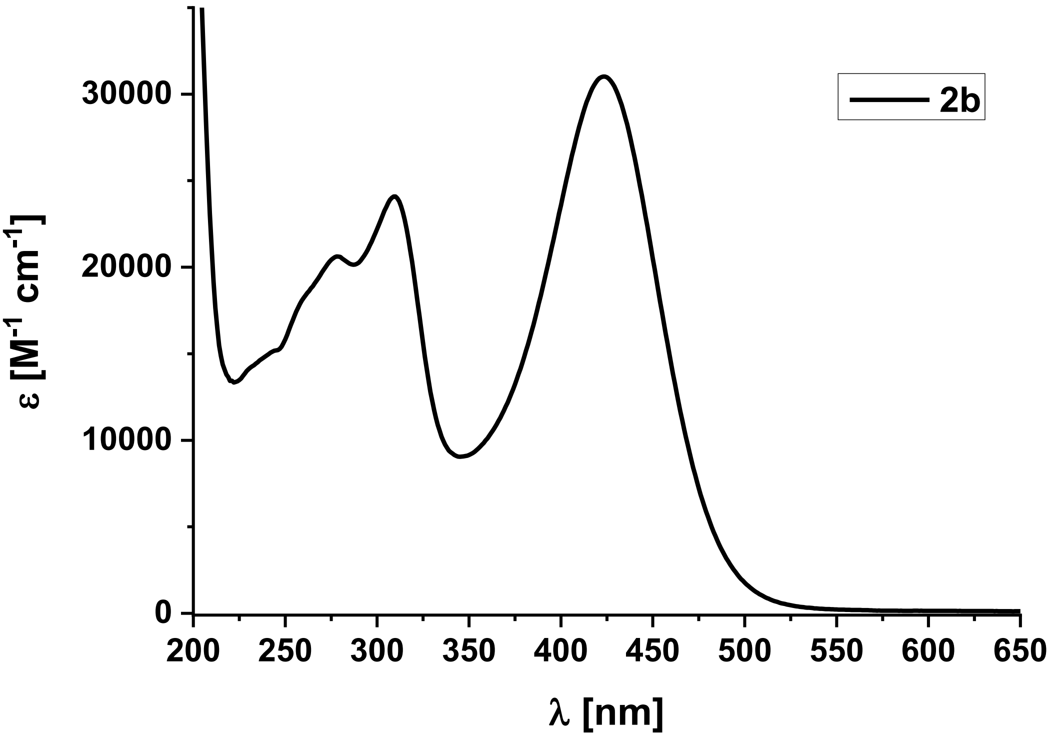

*Figure S 50: UV/Vis extinction spectrum of **2b** measured in MeCN.*

3.4.2 **7**

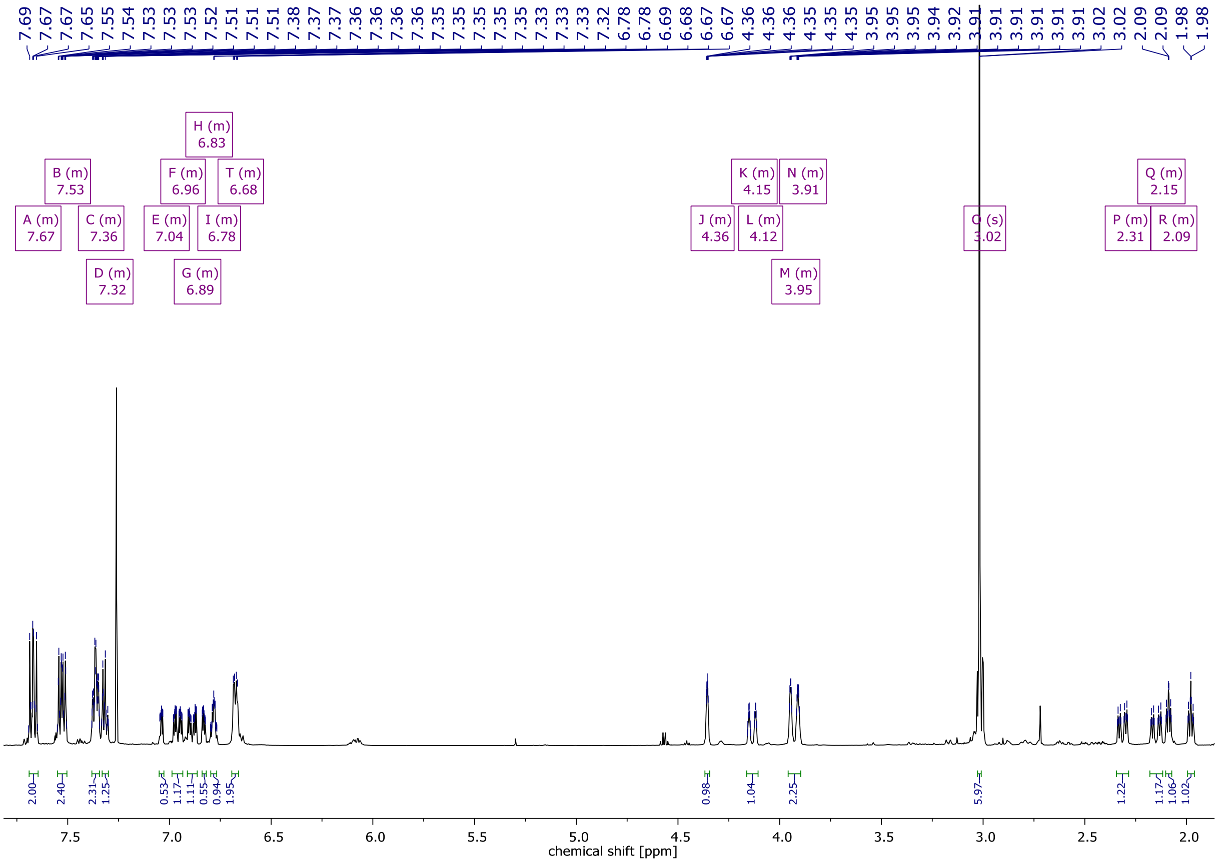

*Figure S 51: ¹H NMR Spectrum of **7** measured in $CDCl_3$ (600 MHz). The minor impurities at 6.06 ppm and in the olefinic region belong to minor bis Diels-Alder adducts.*

Due to the complexity of **7** including the presence of multiple diastereoisomers, integration of the signals is disturbed and therefore slightly unprecise.

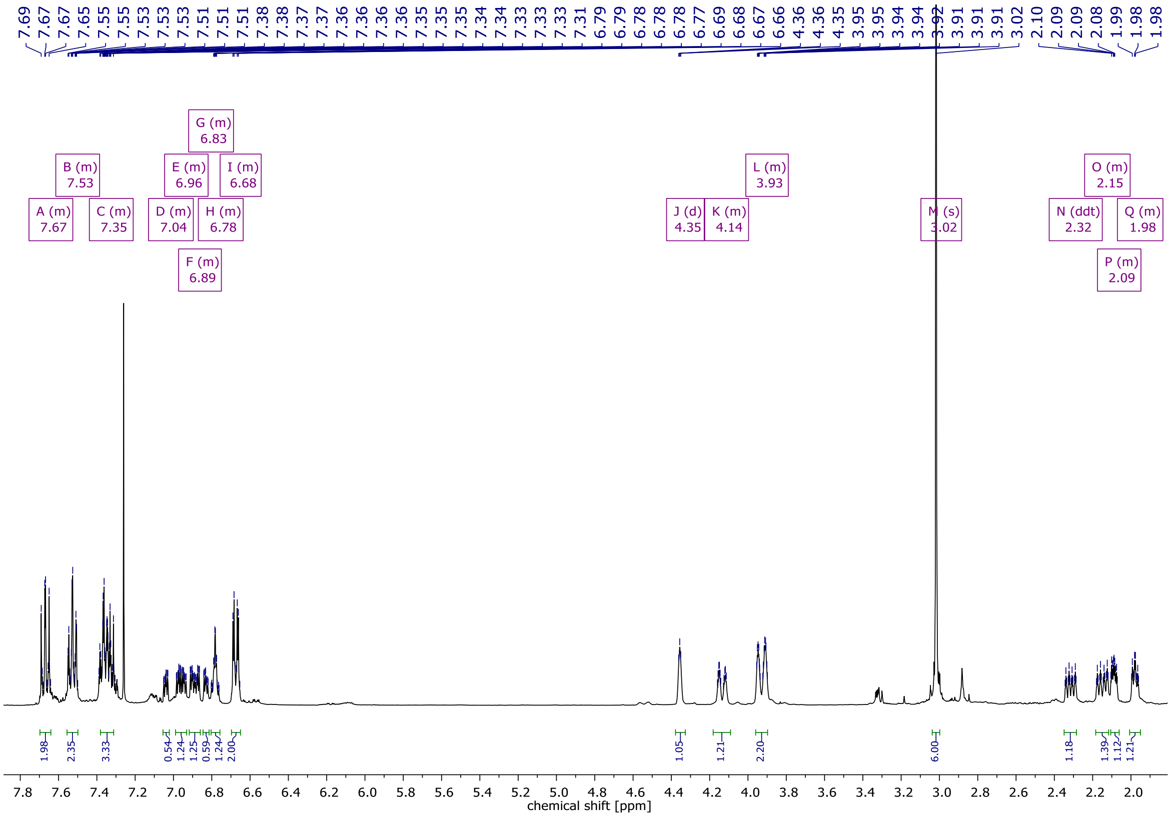

*Figure S 52: ^{1}H NMR Spectrum of **7** measured in $CDCl_3$ (400 MHz).*

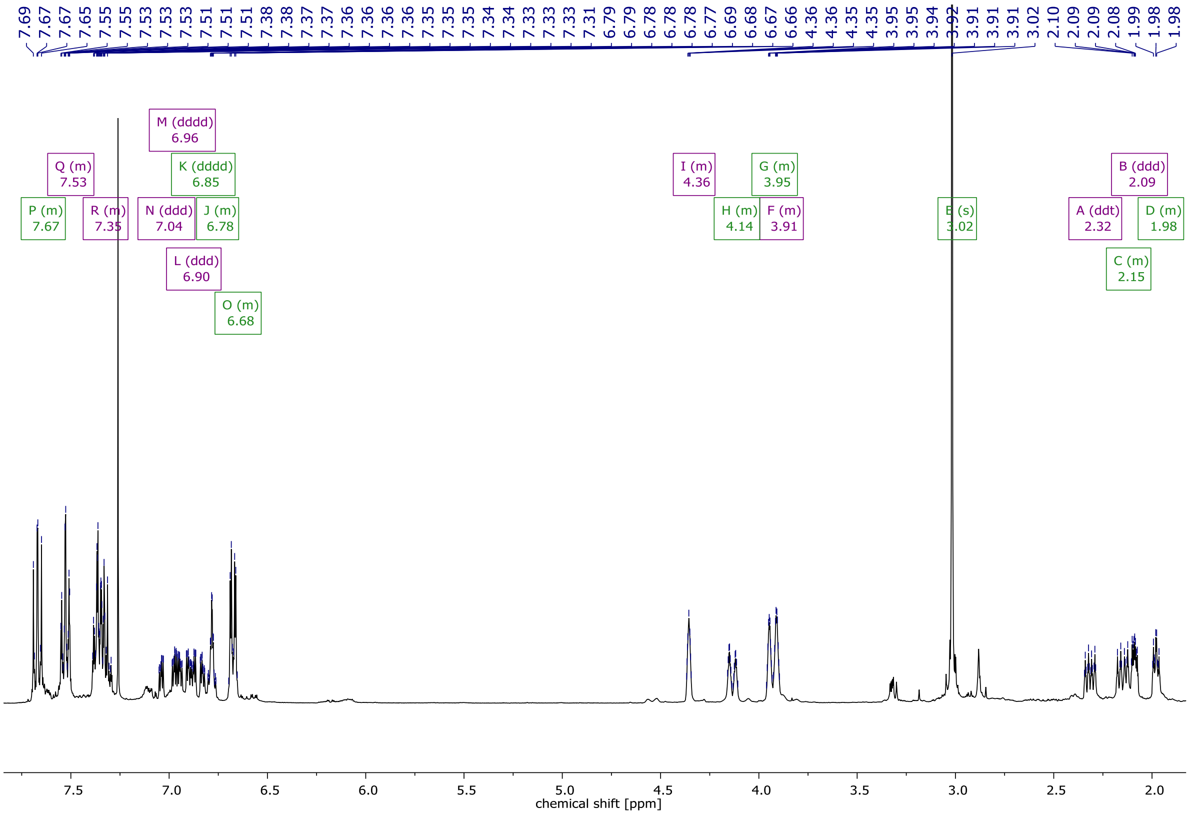

*Figure S 53: ^{1}H NMR spectrum of **7** measured in $CDCl_3$ (400 MHz). Singles labeled with green boxes belong to the dimethylaniline substituted NBD, while purple highlighted signals belong to the phenyl sided NBD. Assignment of the respective signals was done via 2D correlation NMR spectroscopy and comparison to the respective mono-NBD (**4** and **6**) and bis-NBD (**5**) spectra.*

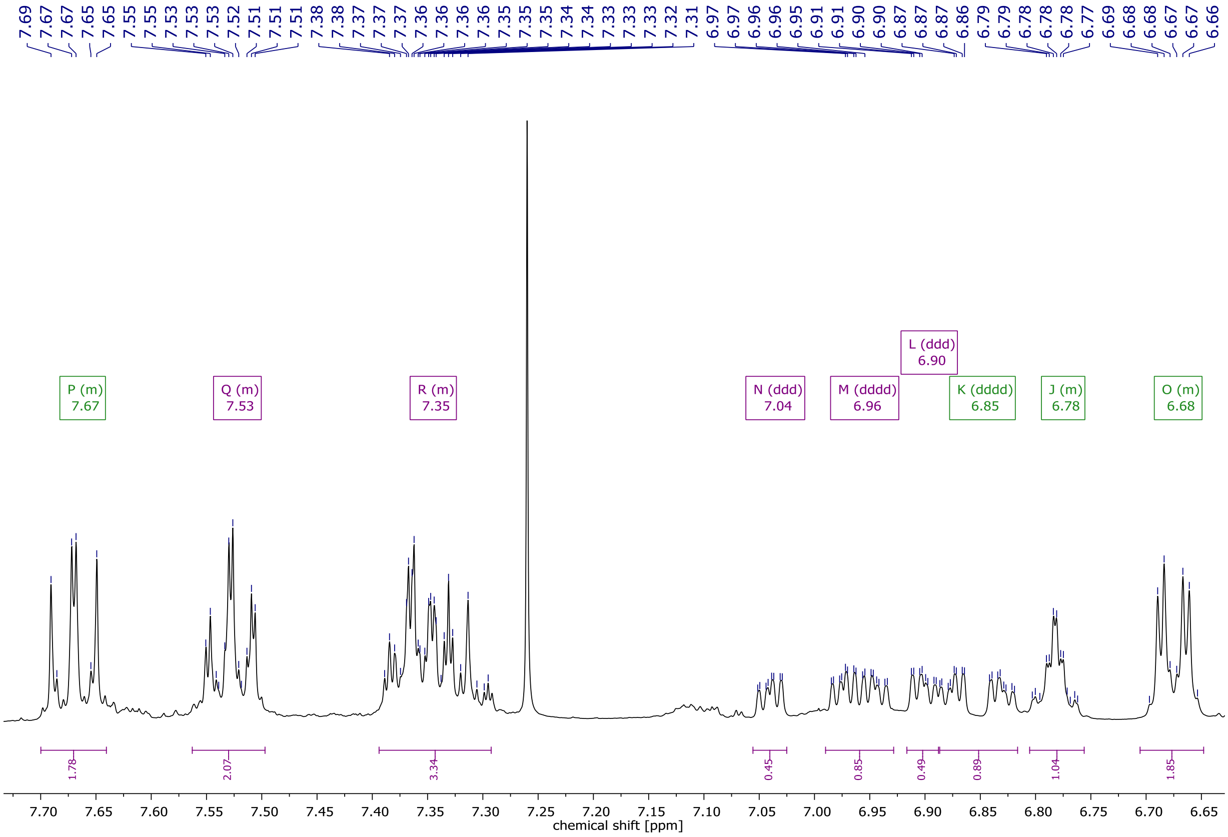

*Figure S 54: Zoom of the aromatic region of the ^{1}H NMR spectrum of **7** measured in $CDCl_3$ (400 MHz).*

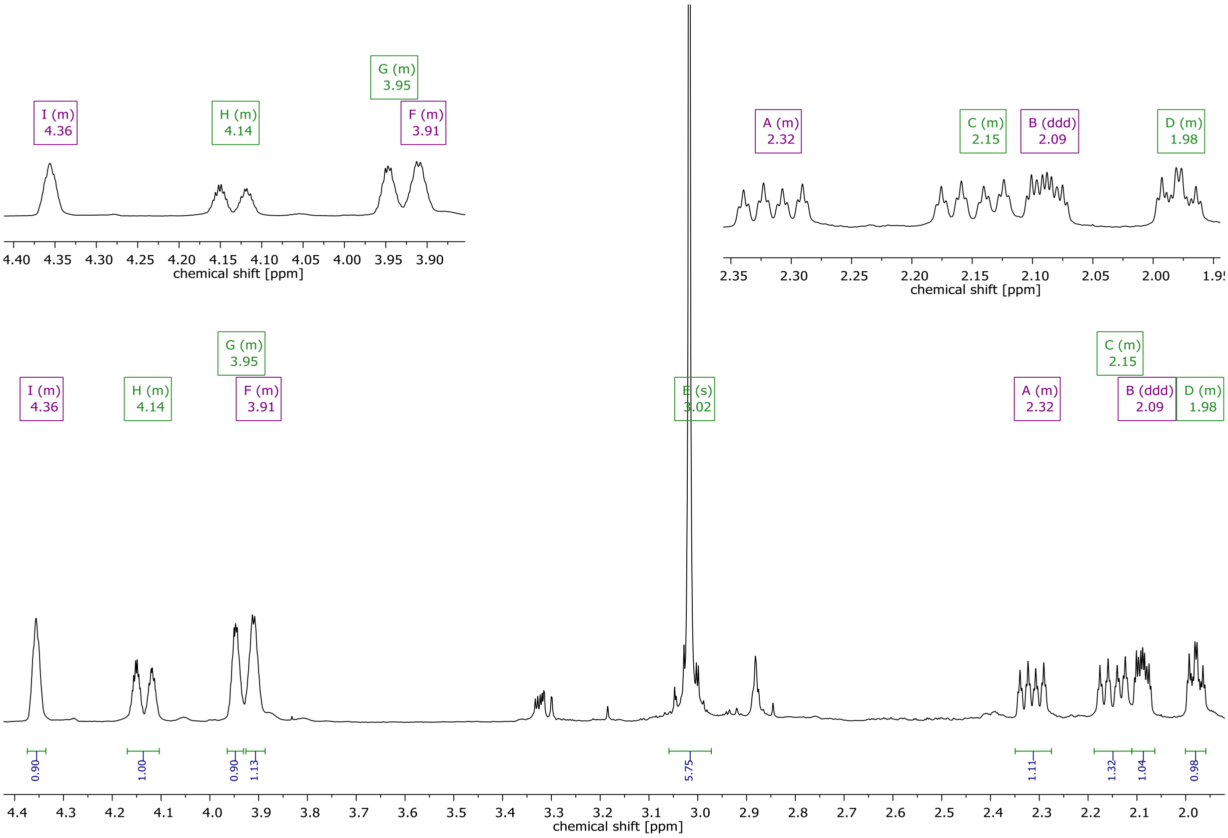

*Figure S 55: Zoom of the olefinic region of the ^{1}H NMR spectrum of **7** measured in $CDCl_3$ (400 MHz).*

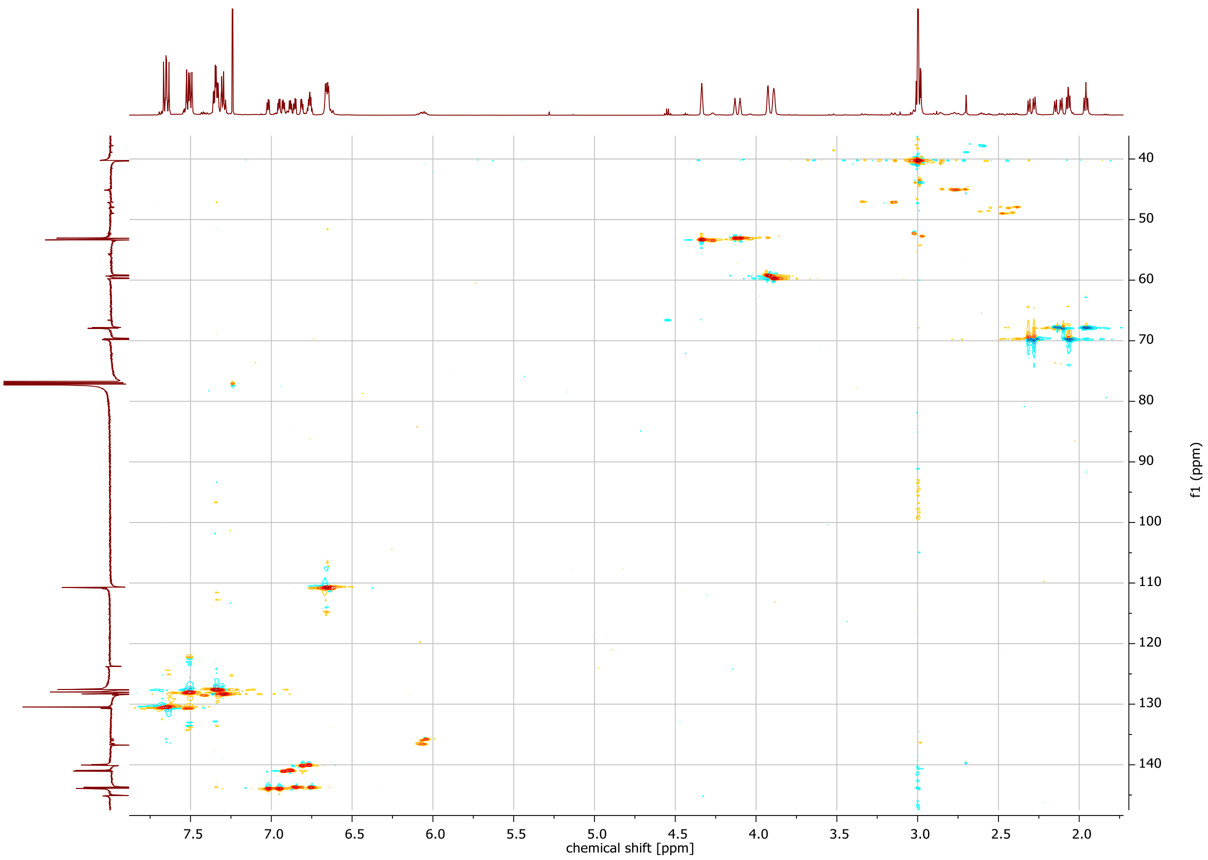

*Figure S 56: HSQC spectrum of **7** measured in $CDCl_3$ (600 MHz).*

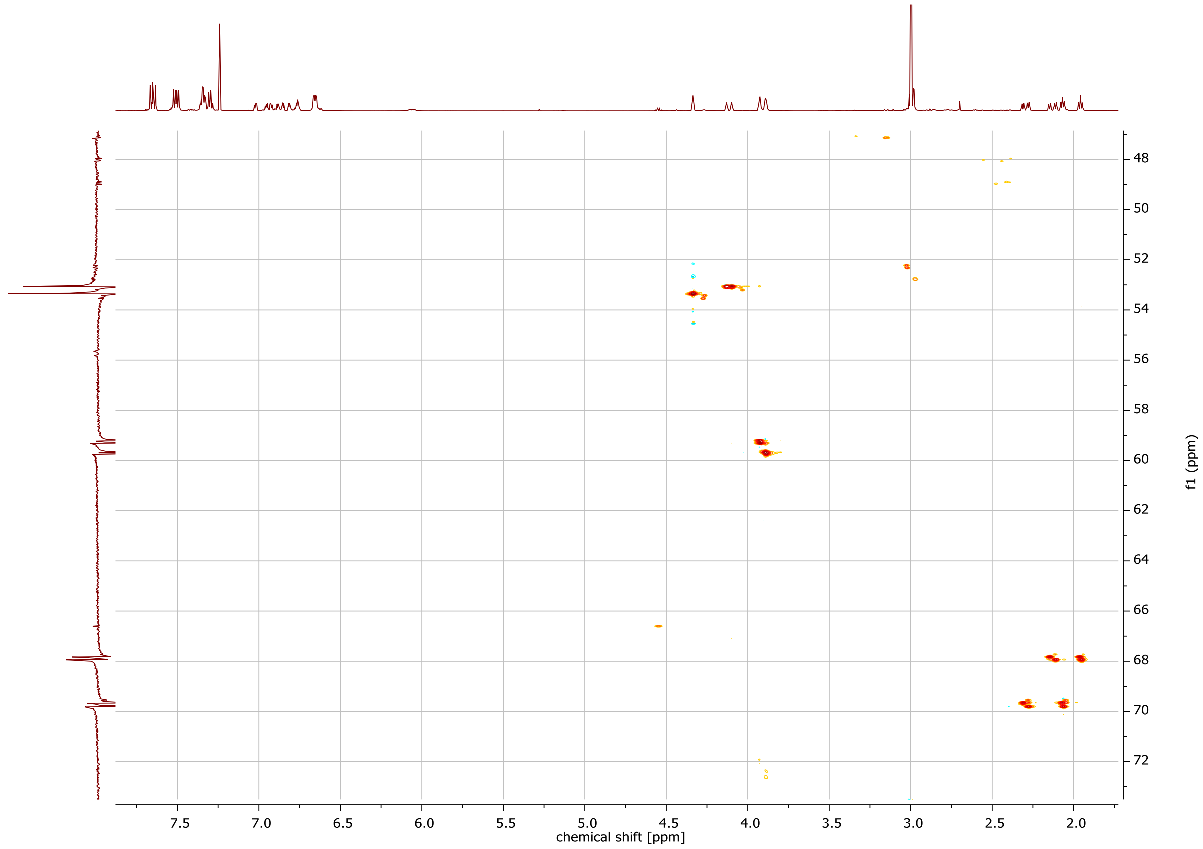

*Figure S 57: Significant section of the 2D selective HSQC spectrum of **7** measured in $CDCl_3$ (600 MHz).*

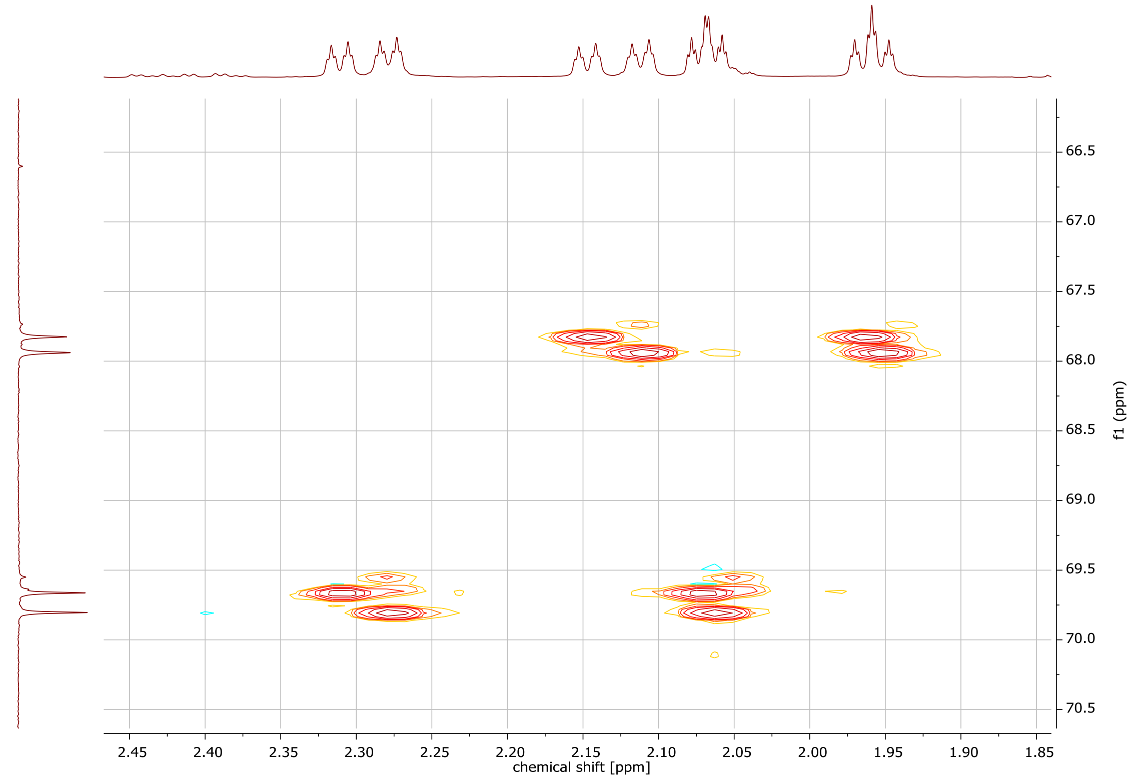

*Figure S 58: Significant section of the 2D selective HSQC spectrum of **7** measured in $CDCl_3$ (600 MHz). On the y-axis the respective deptq spectrum is depicted.*

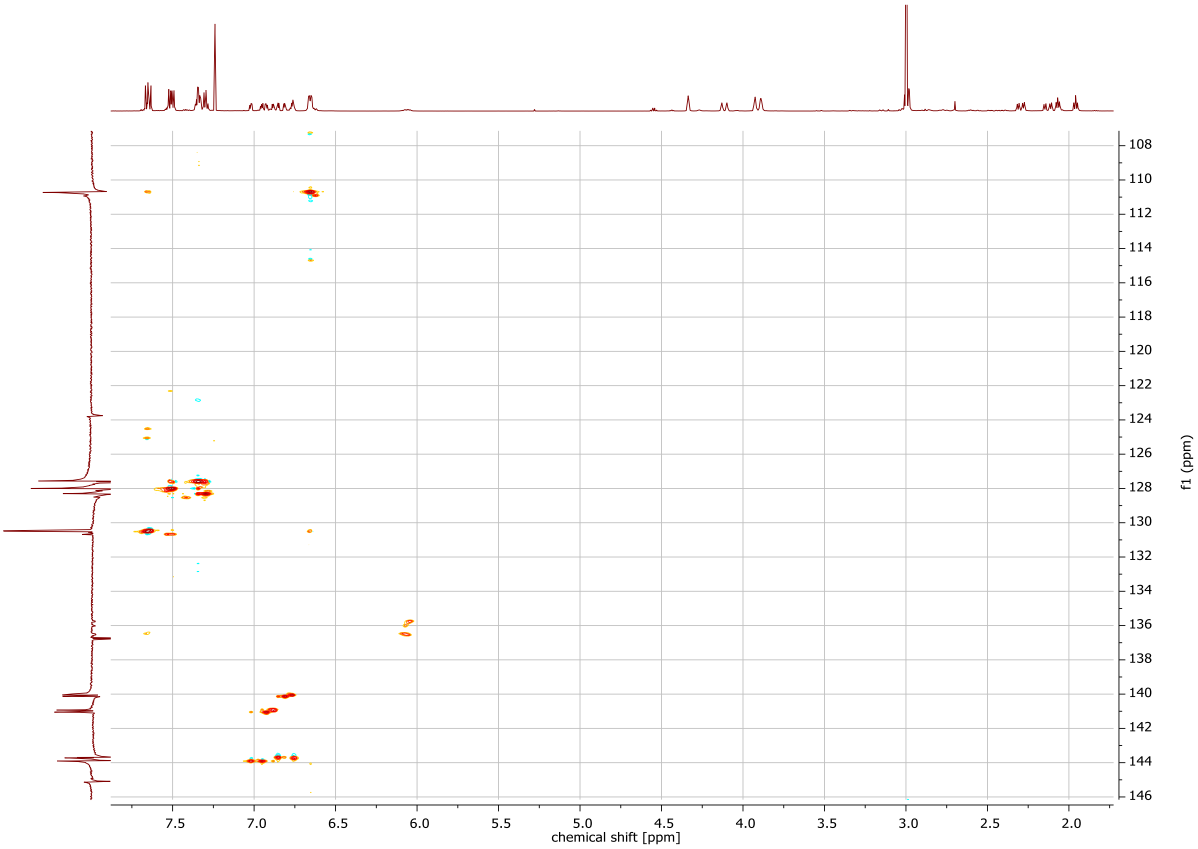

*Figure S 59: Significant section of the 2D selective HSQC spectrum of **7** measured in $CDCl_3$ (600 MHz).*

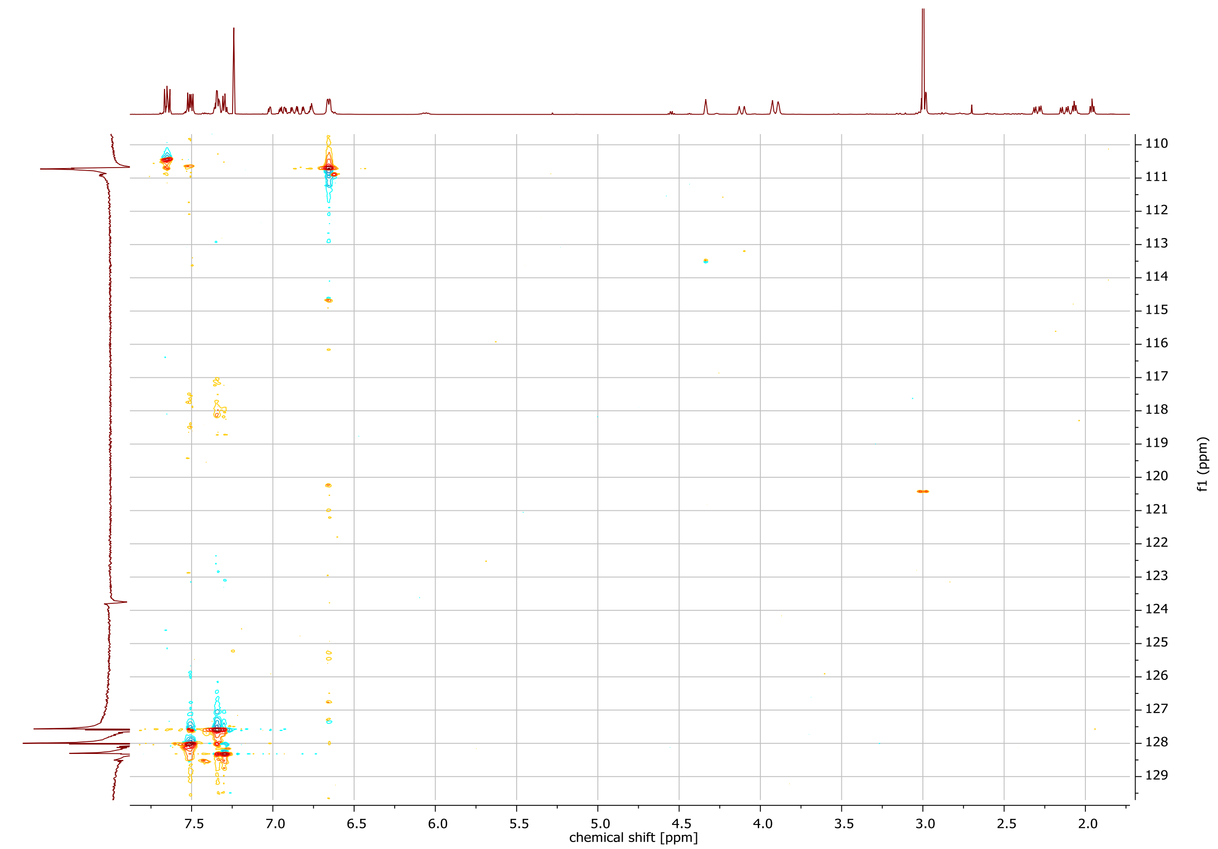

*Figure S 60: Significant section of the 2D selective HSQC spectrum of **7** measured in $CDCl_3$ (600 MHz).*

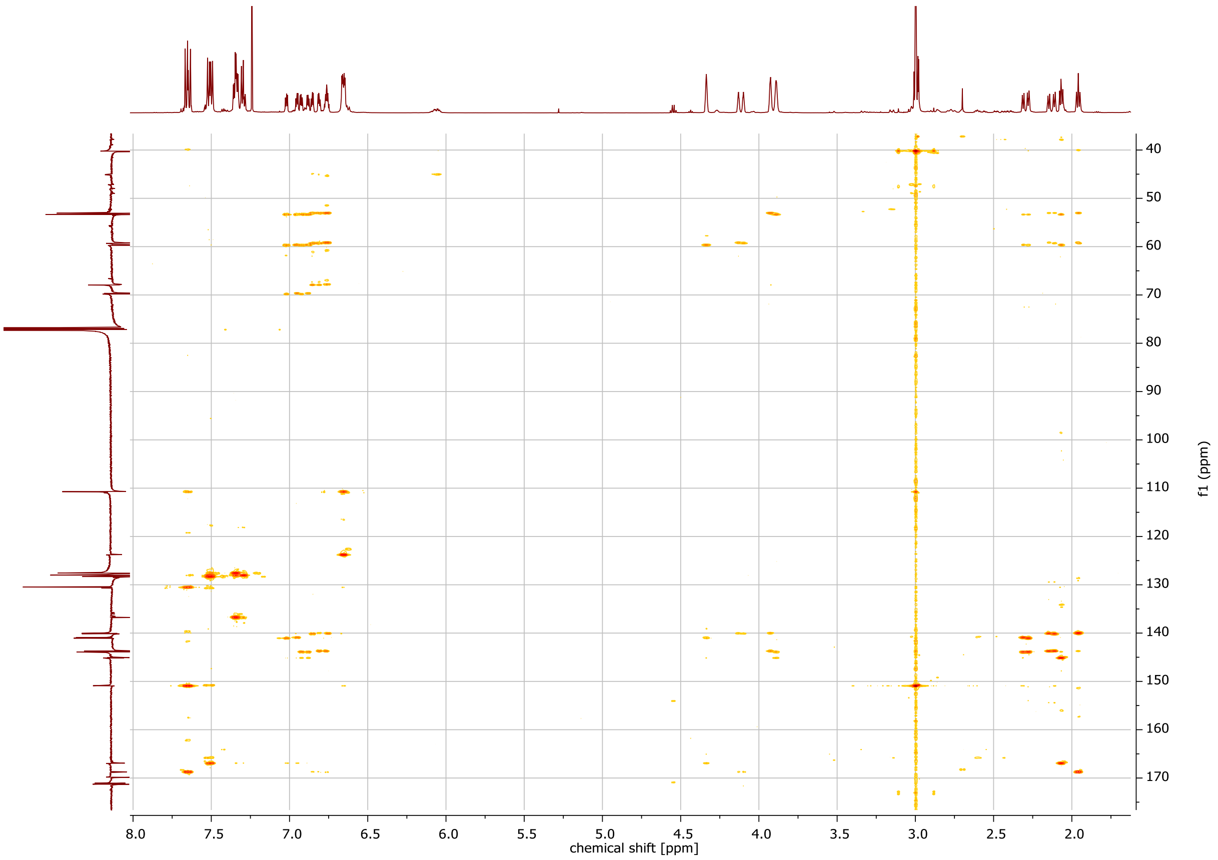

*Figure S 61: HMBC spectrum of **7** measured in $CDCl_3$ (600 MHz).*

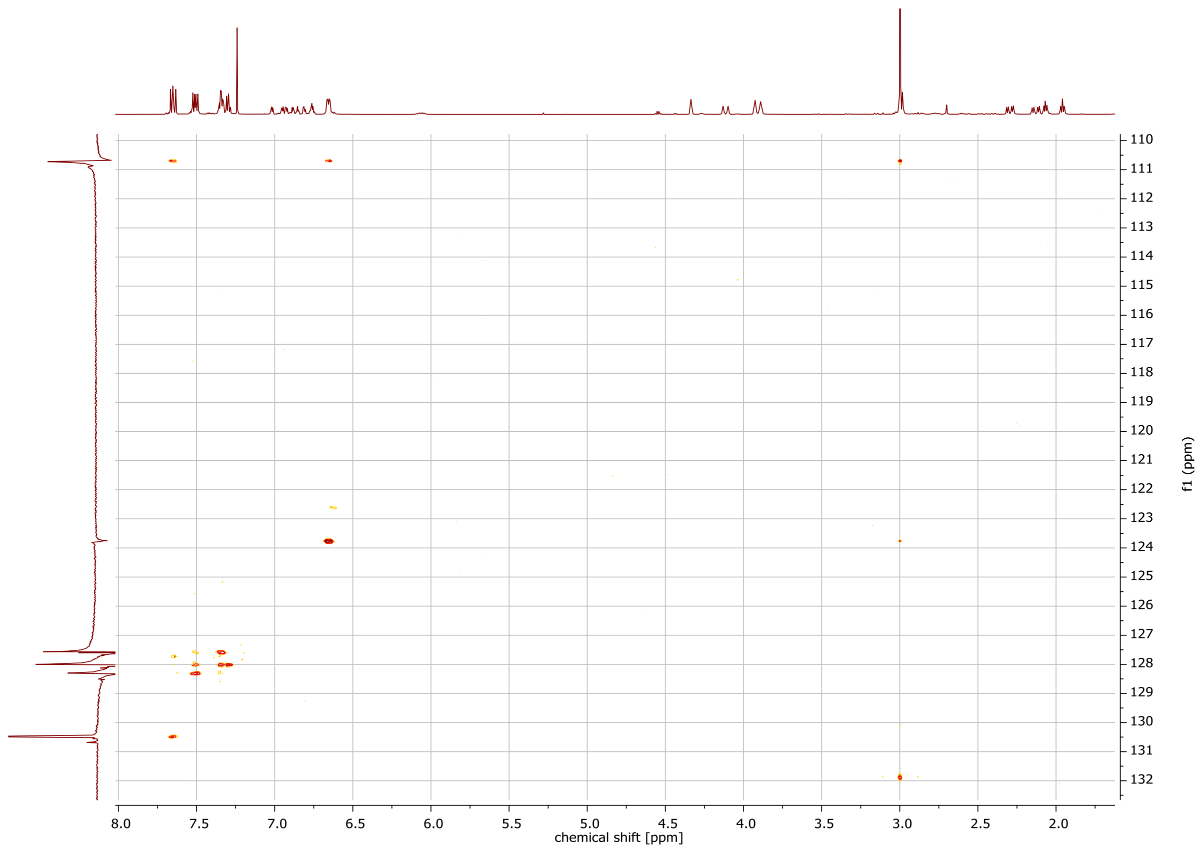

*Figure S 62: Significant section of the 2D selective HMBC spectrum of **7** measured in $CDCl_3$ (600 MHz).*

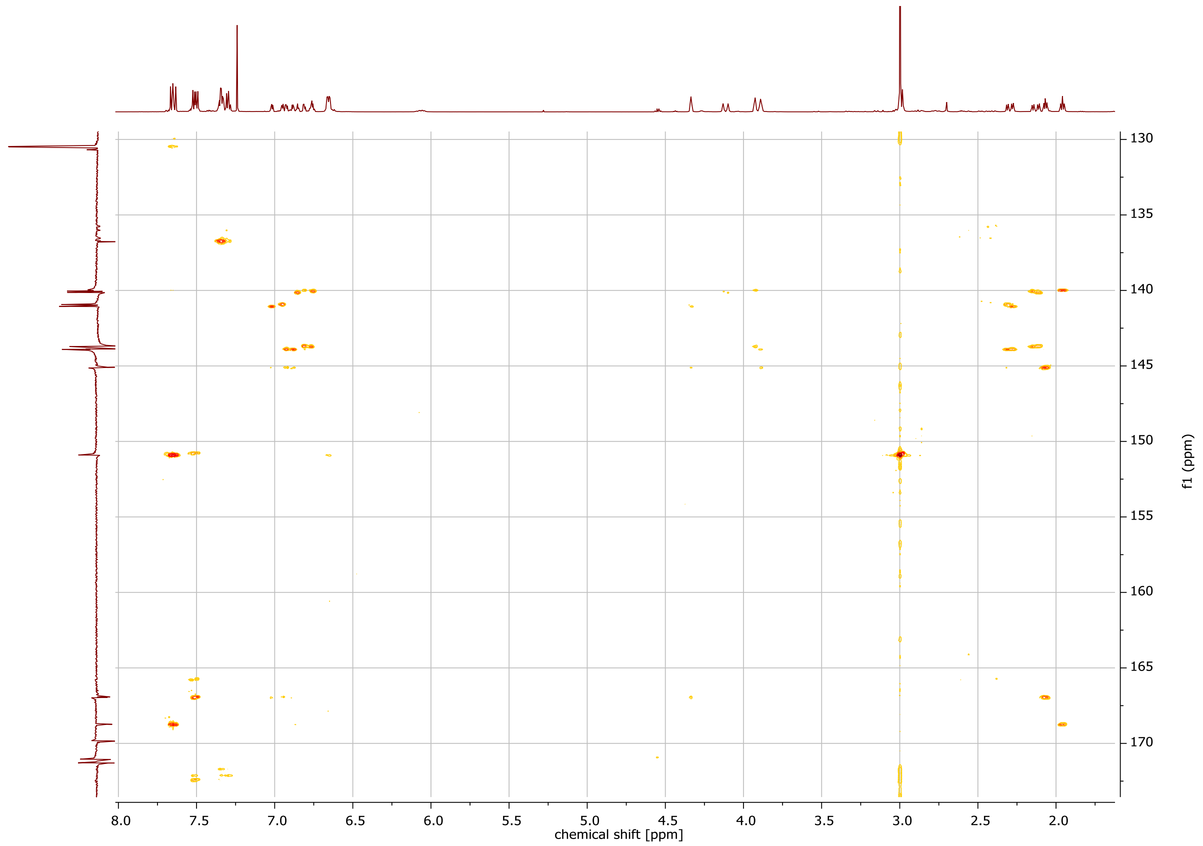

*Figure S 63: Significant section of the 2D selective HMBC spectrum of **7** measured in $CDCl_3$ (600 MHz).*

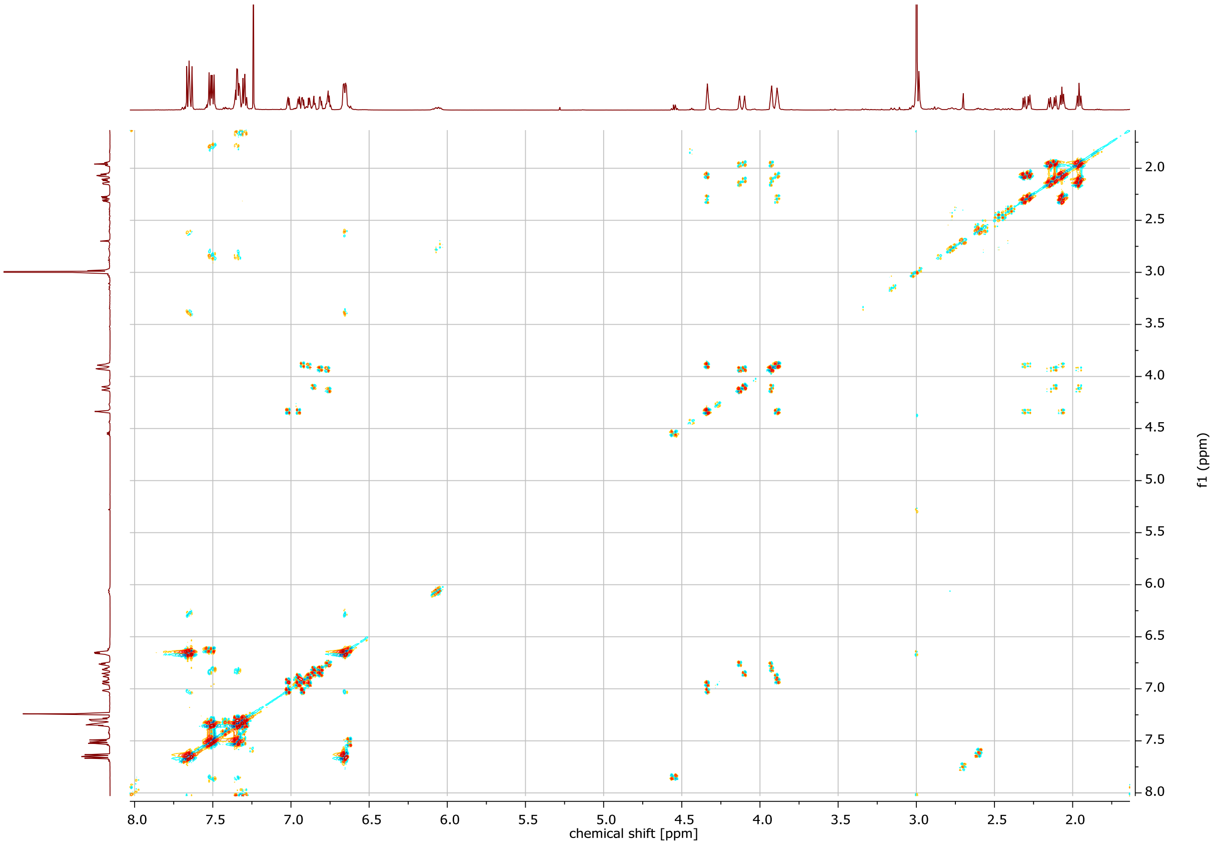

*Figure S 64: COSY spectrum of **7** measured in $CDCl_3$ (600 MHz).*

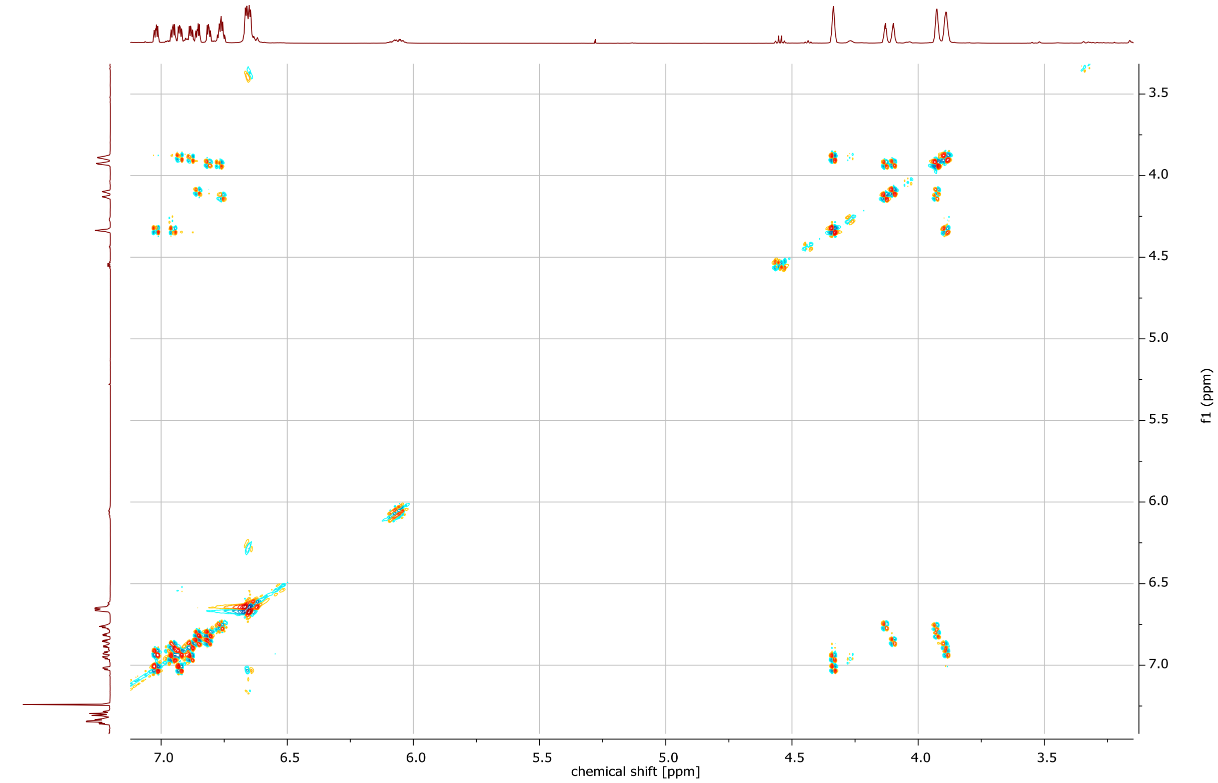

*Figure S 65: Significant section of the COSY spectrum of **7** (600 MHz).*

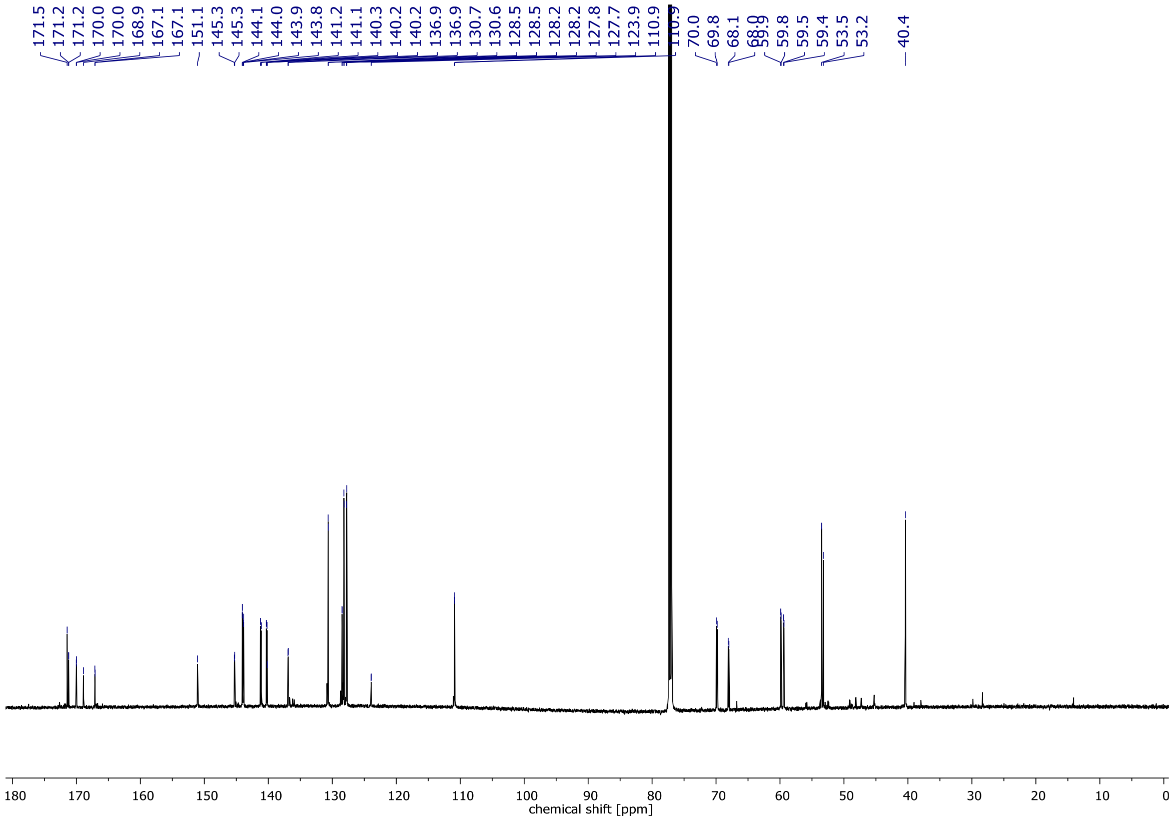

*Figure S 66: ^{13}C NMR Spectrum of **7** measured in $CDCl_3$ (151 MHz). Here, minor indications of aliphatic QC signals are already found.*

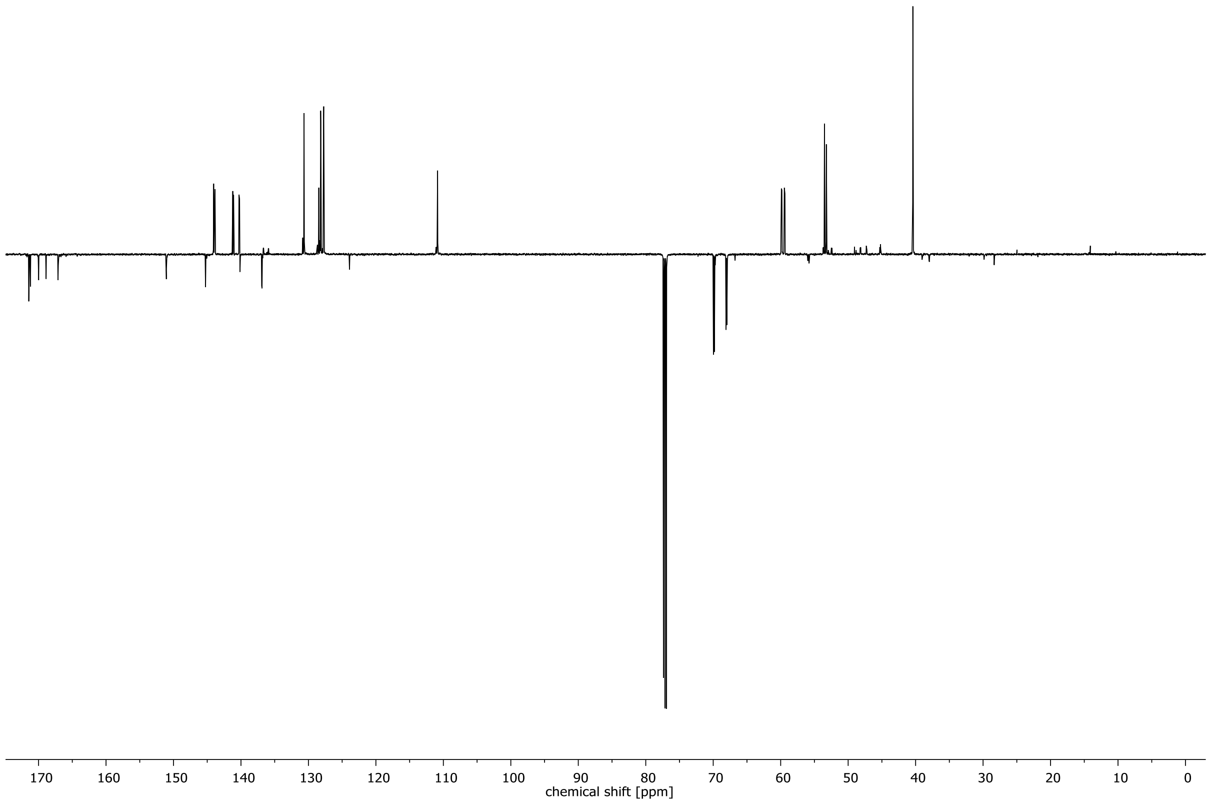

*Figure S 67: Deptq spectrum of **7** measured in $CDCl_3$ (151 MHz).*

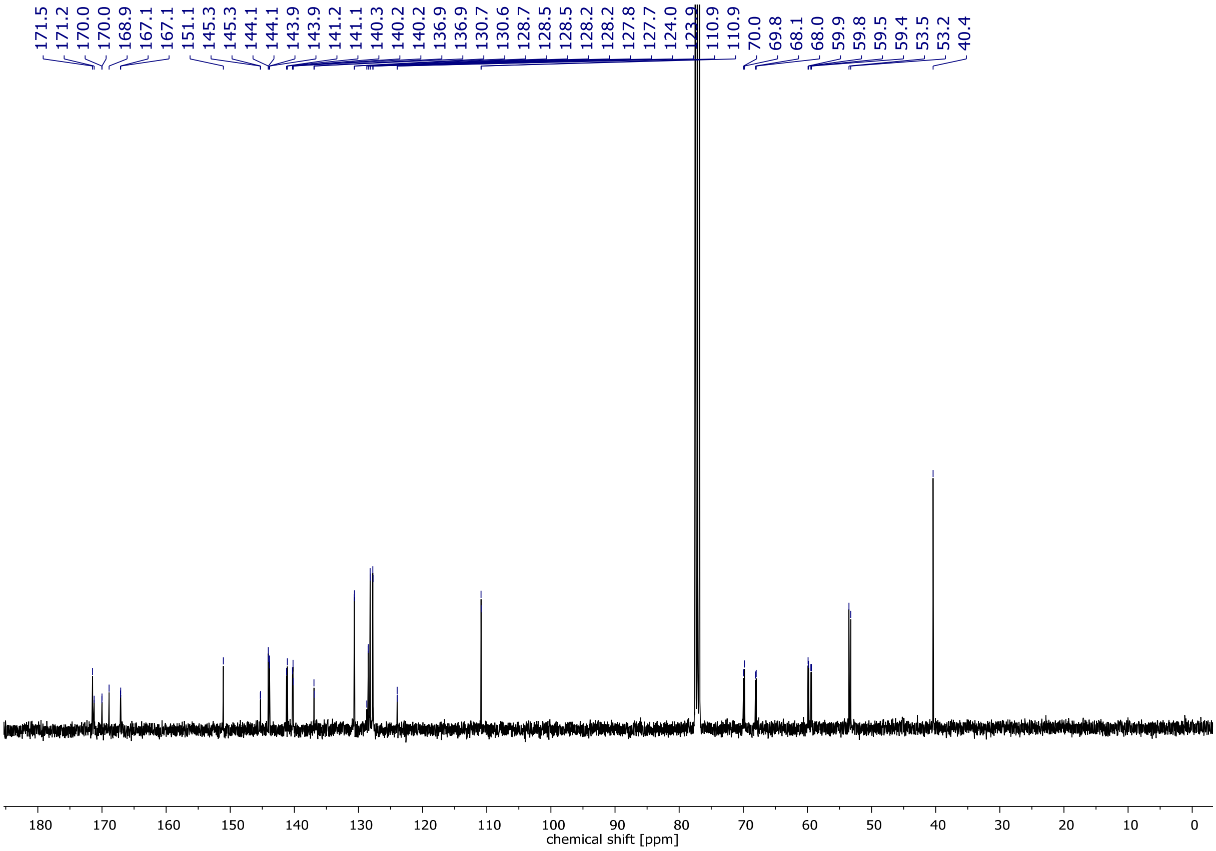

*Figure S 68: Additional ^{13}C NMR spectrum of **7** measured in $CDCl_3$ (101 MHz). This sample was measured with less resolution but without the presence of any QC species.*

Acquisition Parameter

Source Type	APPI	Ion Polarity	Positive	Set Nebulizer	2.0 Bar
Focus	Active			Set Dry Heater	200 °C
Scan Begin	50 m/z	Set Capillary	700 V	Set Dry Gas	2.0 l/min
Scan End	1500 m/z	Set End Plate Offset	-500 V	Set Divert Valve	Waste

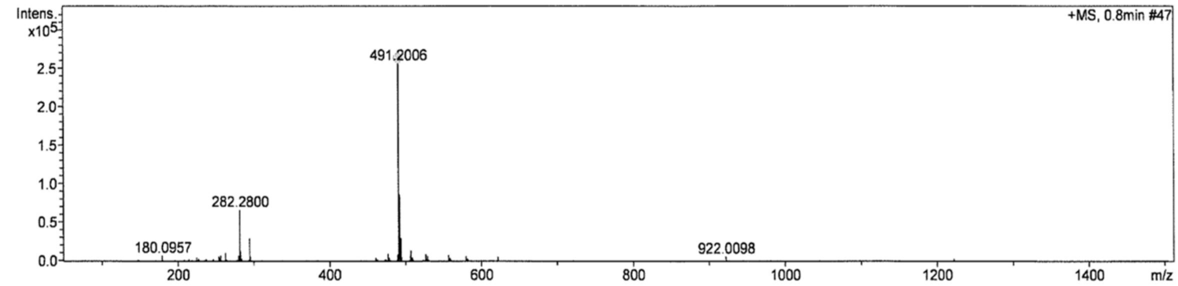

Meas. m/z	#	Ion Formula	m/z	err [ppm]	mSigma	# mSigma	Score	rdb	e⁻ Conf	N-Rule
491.2006	1	C31H28ClN4	491.1997	-1.9	25.9	1	100.00	19.5	even	ok

*Figure S 69: HRMS (APPI) of **7**. Impurity at 922 m/z similar as indicated before.*

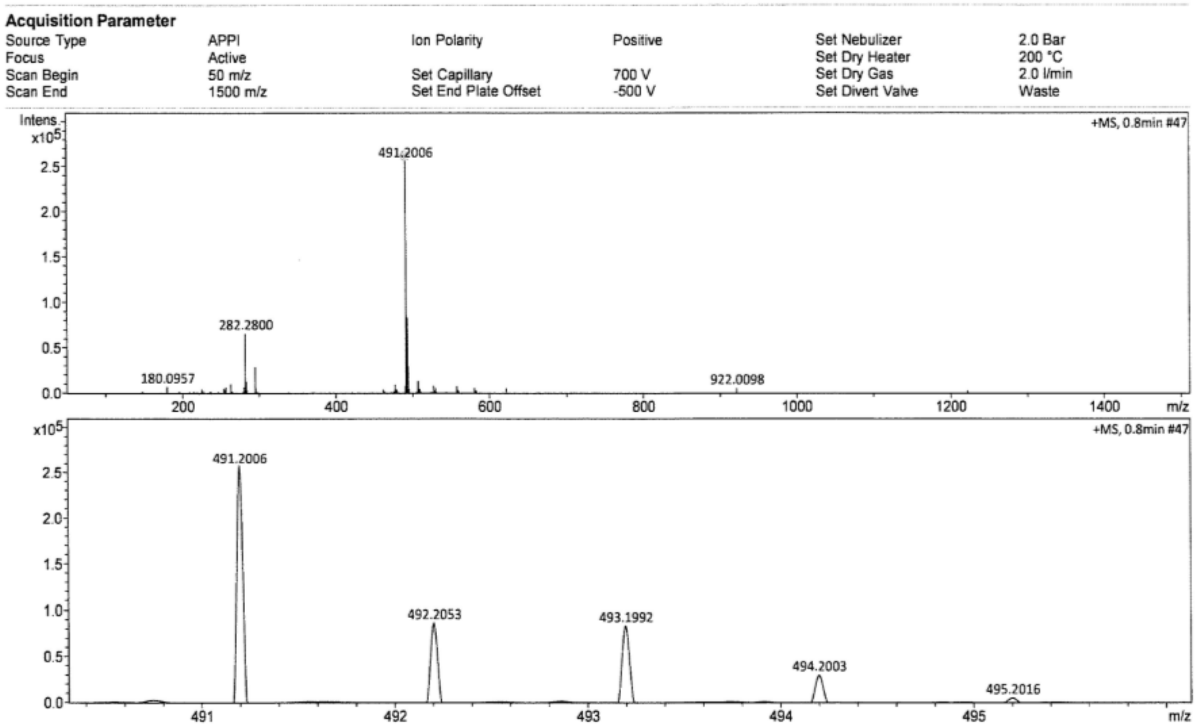

Figure S 70: Zoom of HRMS (APPI) of **7**.

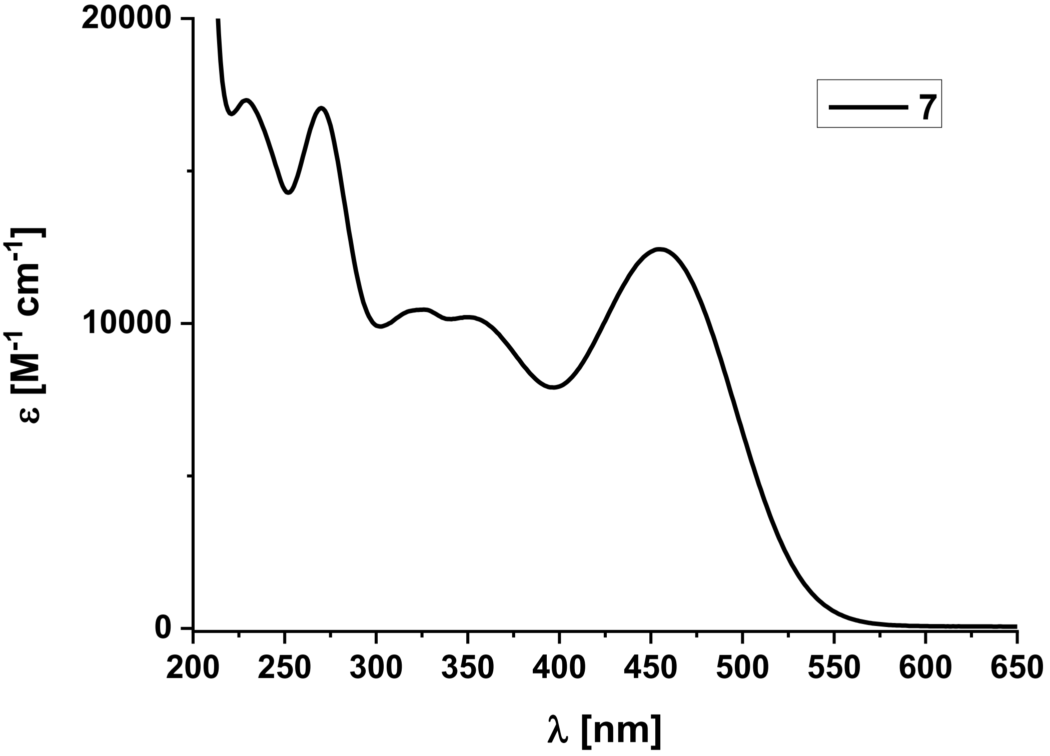

Figure S 71: UV/Vis extinction spectrum of **7** measured in MeCN.

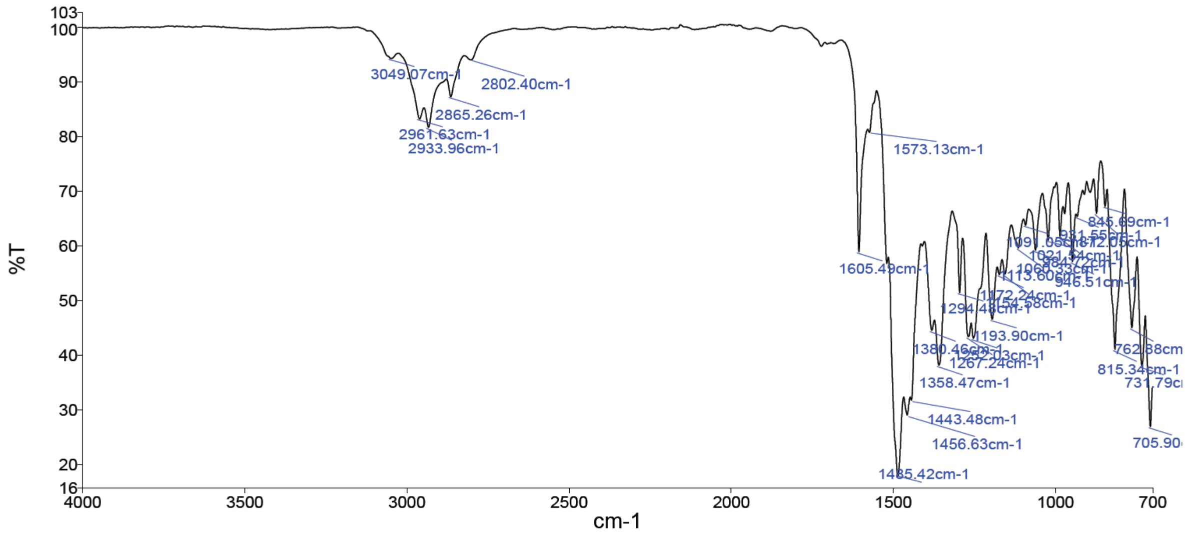

*Figure S 72: ATIR spectrum of **7**.*

3.4.3 **12b**

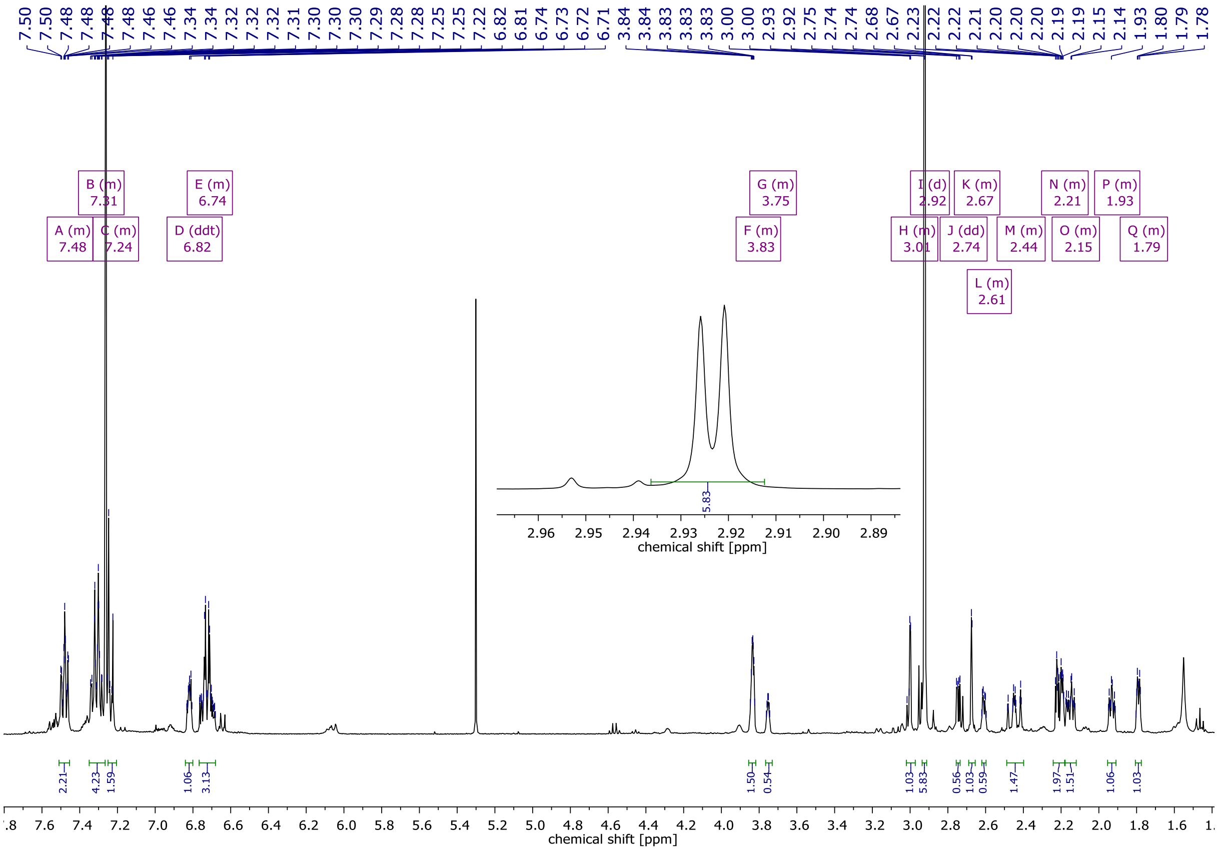

*Figure S 73: ^{1}H NMR spectrum of **12b** measured in $CDCl_3$ (400 MHz) including a zoom of the signal originated by the CH_3 moieties at the amine group of the dimethylaniline.*

Due to the complexity of the system, including the presence of multiple diastereoisomers and species, overlapping signals disturb the integration signals. Since these spectra were recorded after an irradiation experiment, baseline uncertainties resulted by minor photodecomposition processes

additionally influence the quality of integration. The shown spectra are obtained after 1 h of irradiation at 475 nm.

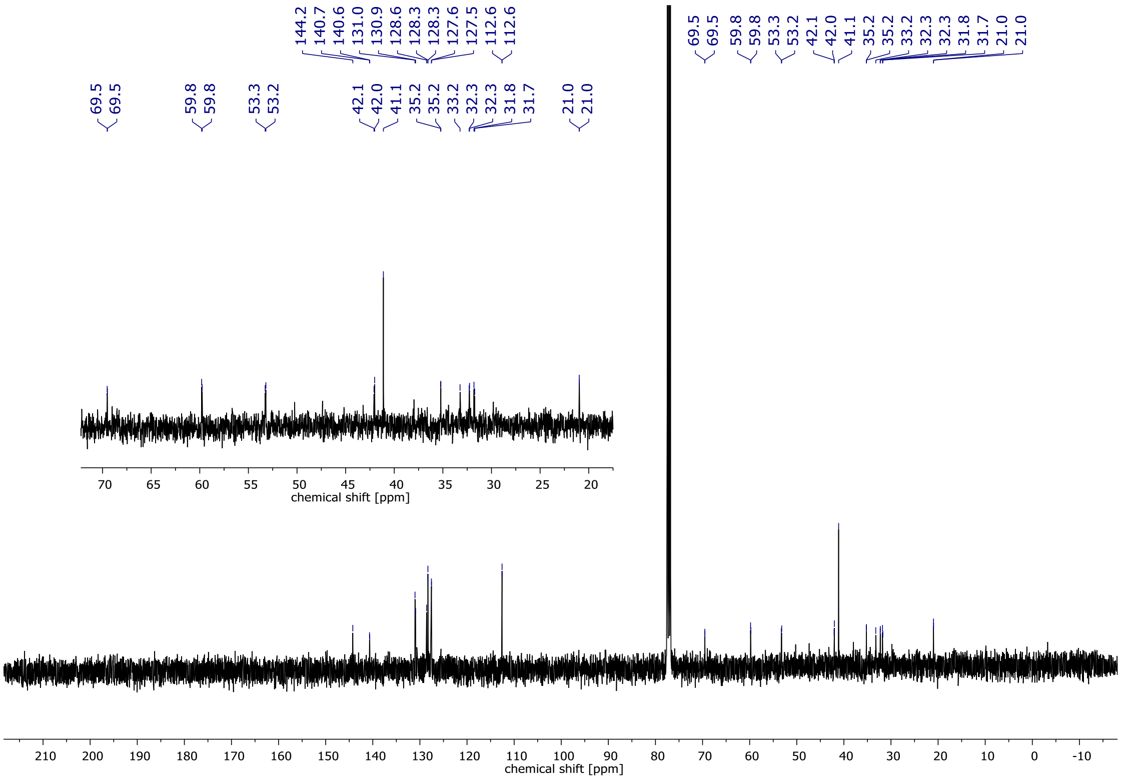

*Figure S 74: ^{13}C NMR Spectrum of **12b** measured in $CDCl_3$ (101 MHz). Although the intensity is relatively low, compared to the spectrum of **7**, the lack of one set of olefinic signals combined with the arise of a new set of aliphatic signals corresponding to the QC species can be found.*

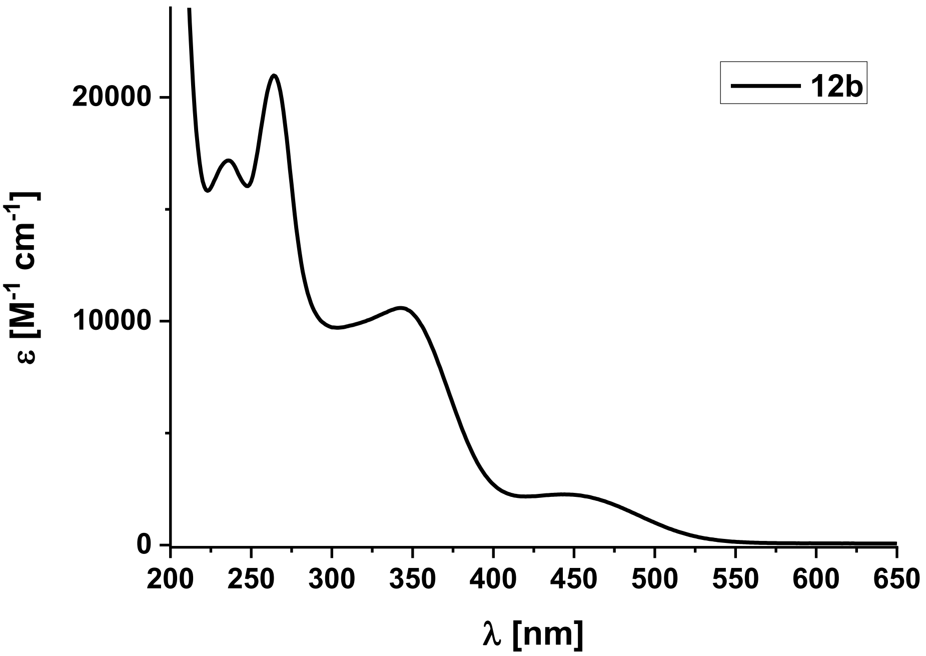

*Figure S 75: UV/Vis extinction spectrum of **12b** measured in MeCN.*

3.4.4 **12**

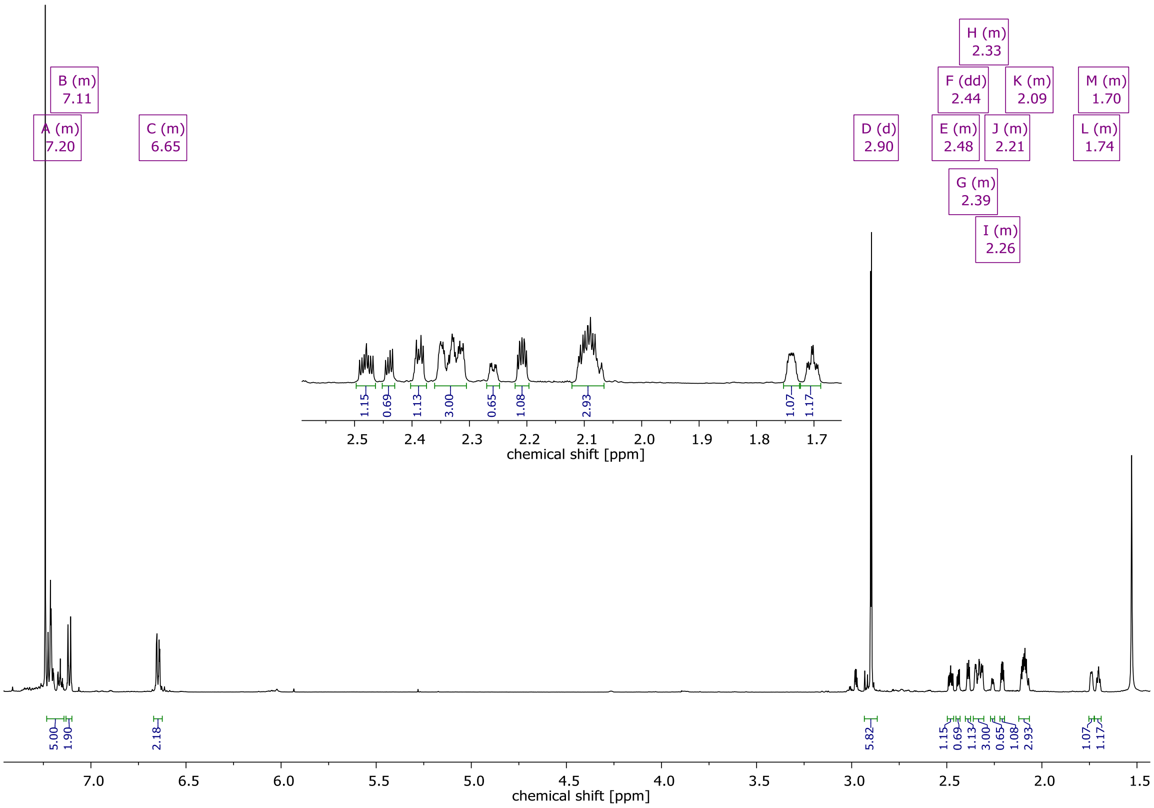

*Figure S 76: ¹H NMR spectrum of **12** measured in $CDCl_3$ (600 MHz). Obtained after direct irradiation of **7** at 340 nm.*

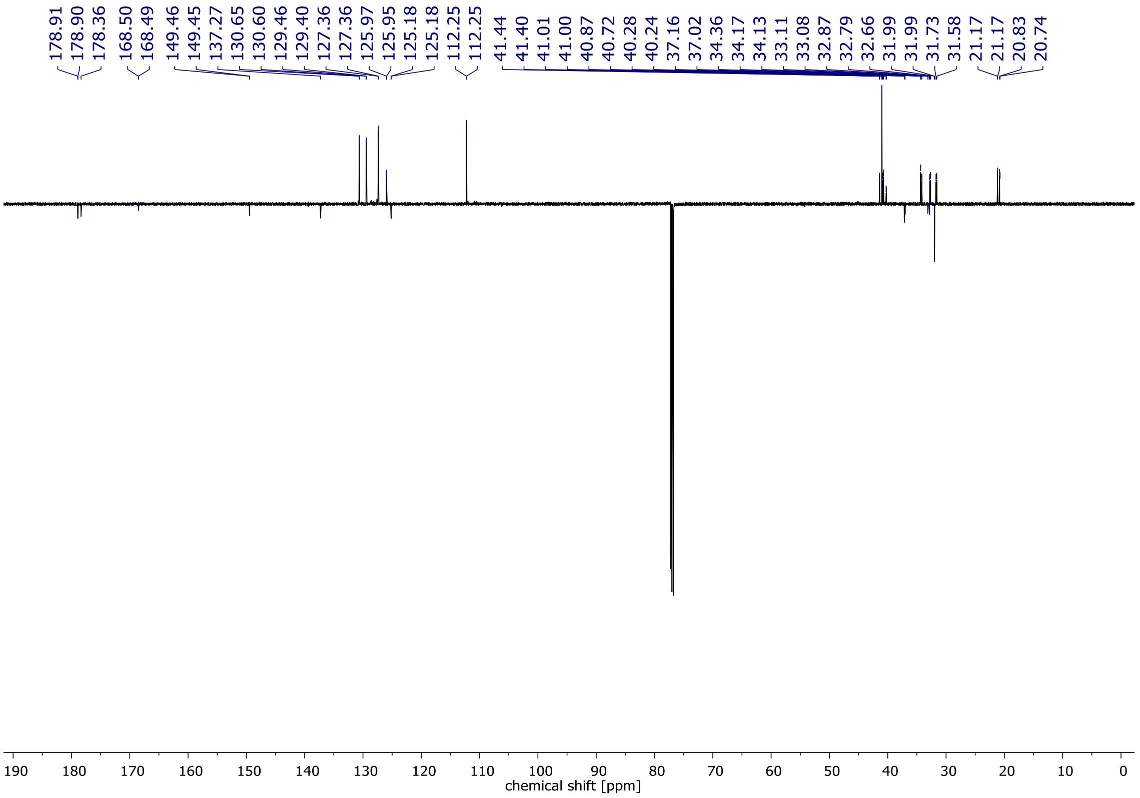

*Figure S 77: Deptq spectrum of **12** measured in $CDCl_3$ (151 MHz).*

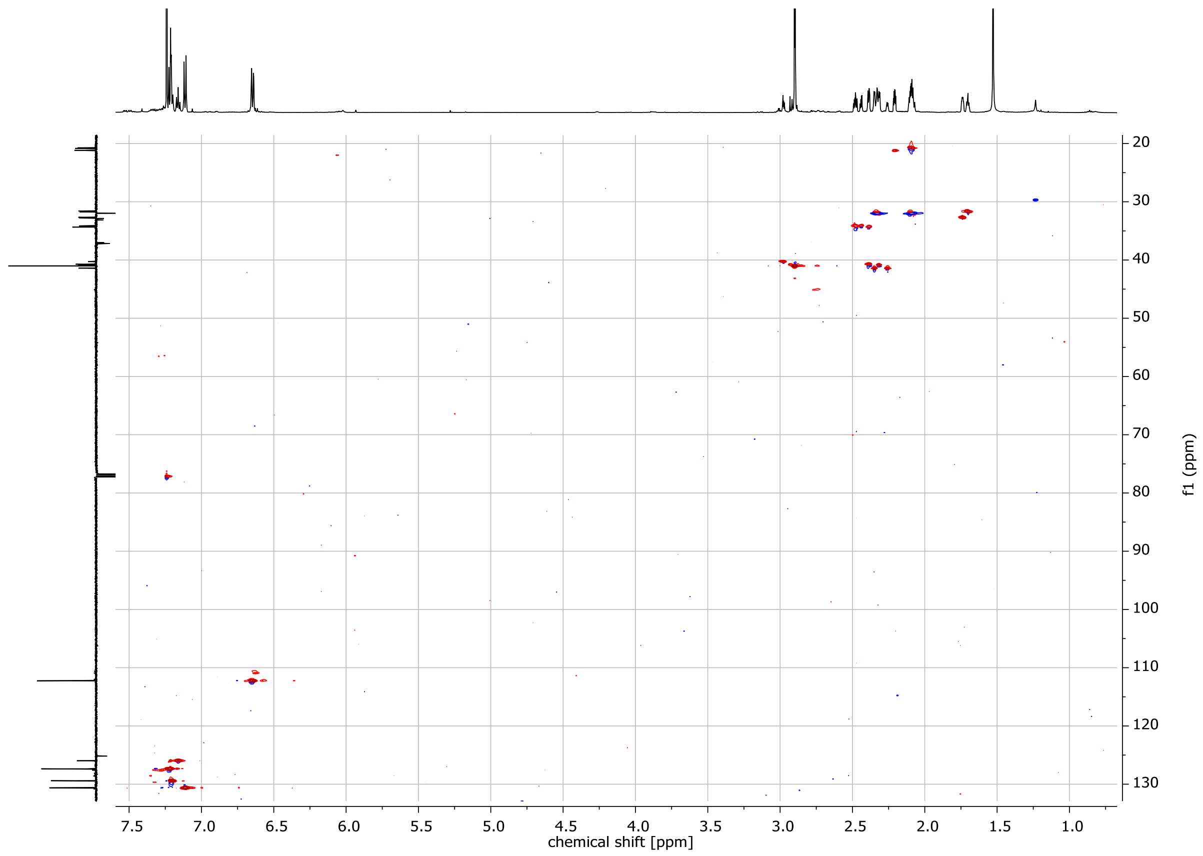

*Figure S 78: HSQC spectrum of **12** measured in $CDCl_3$ (600 MHz).*

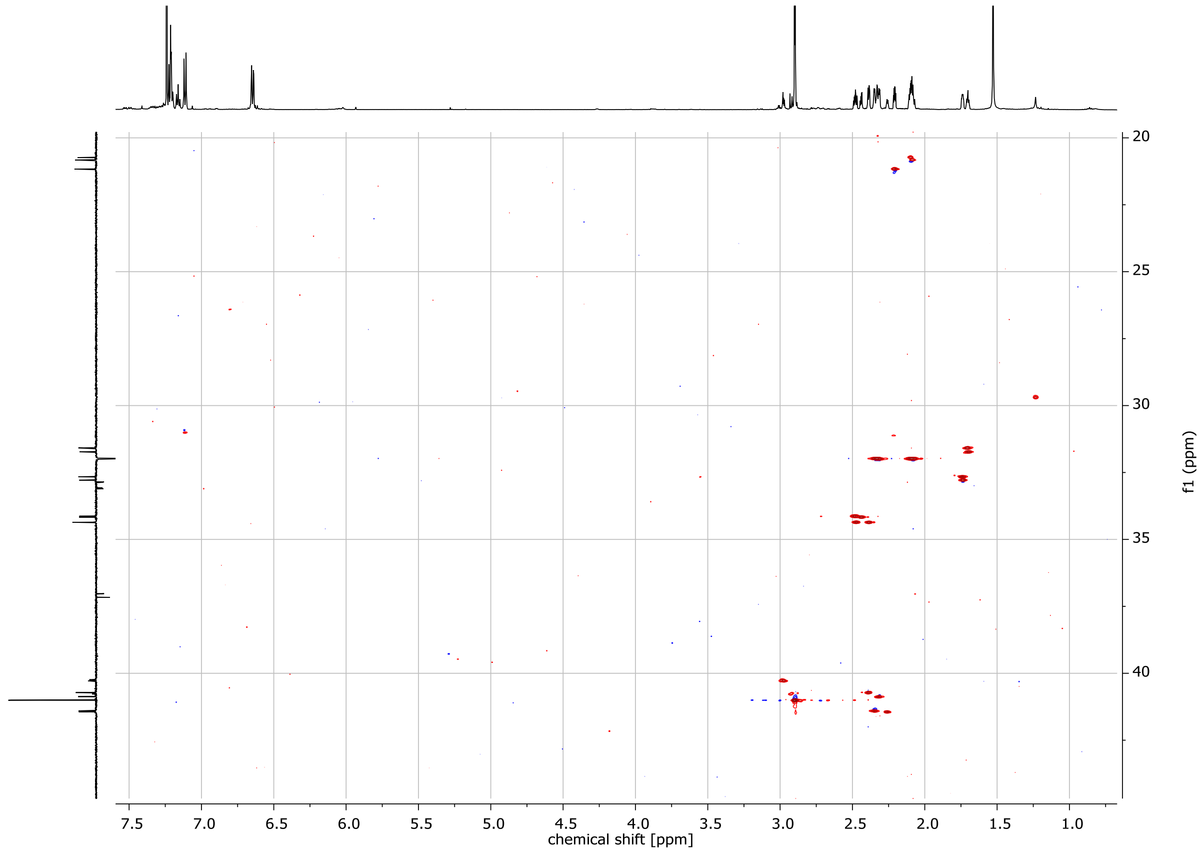

*Figure S 79: Significant section of the 2D selective HSQC spectrum of **12** measured in $CDCl_3$ (600 MHz).*

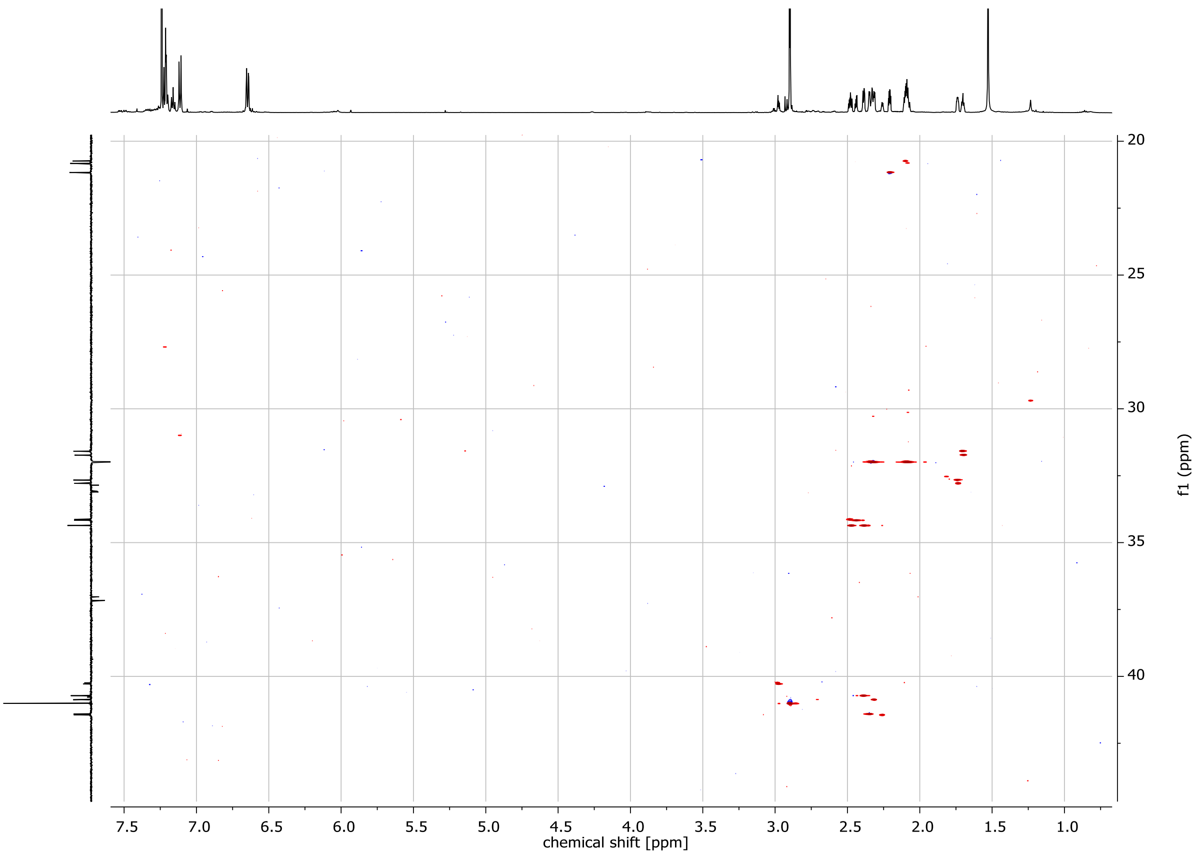

*Figure S 80: Significant section of the 2D selective HSQC spectrum of **12** measured in $CDCl_3$ (600 MHz).*

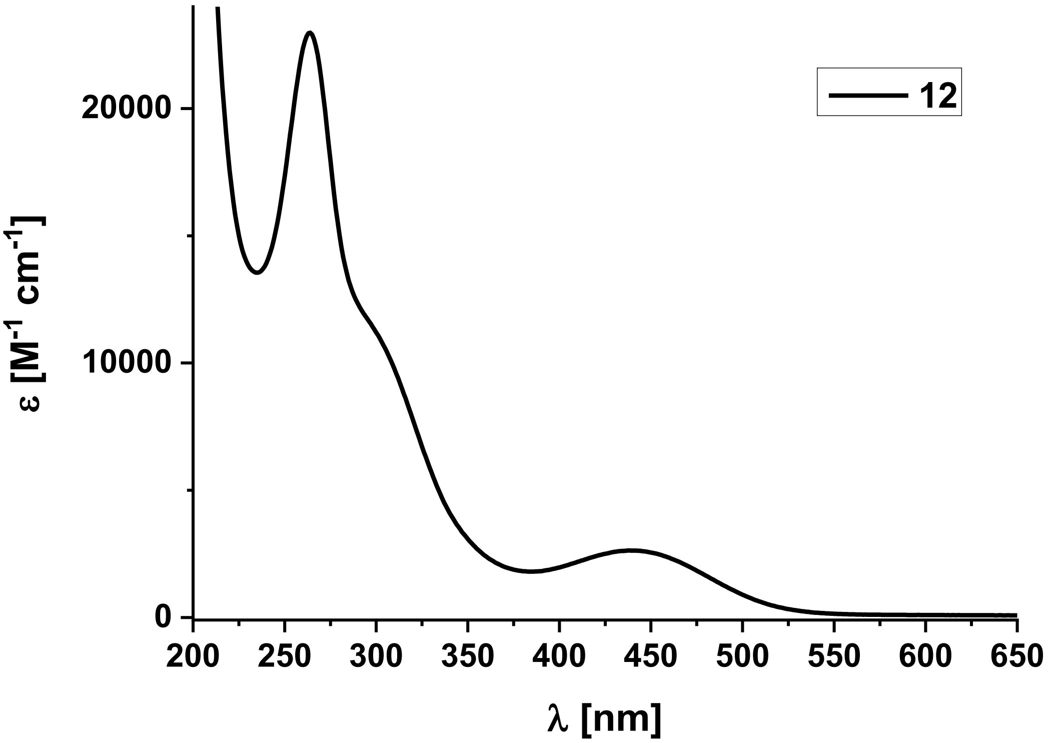

*Figure S 81: UV/Vis extinction spectrum of **12** measured in MeCN.*

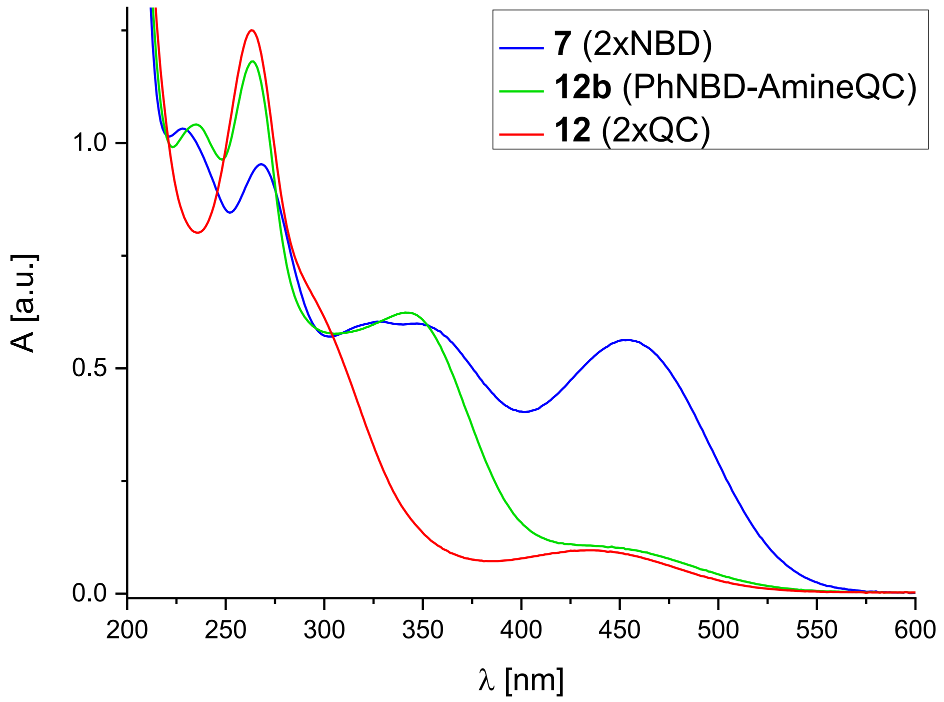

*Figure S 82: UV/Vis spectra of NBD **7**, single switched QC **12b** and bis-QC **12** measured in MeCN.*

3.5 Asymmetric dimethylaniline-anisyl-phenyl-tris-NBD hybrid

3.5.1 **3**

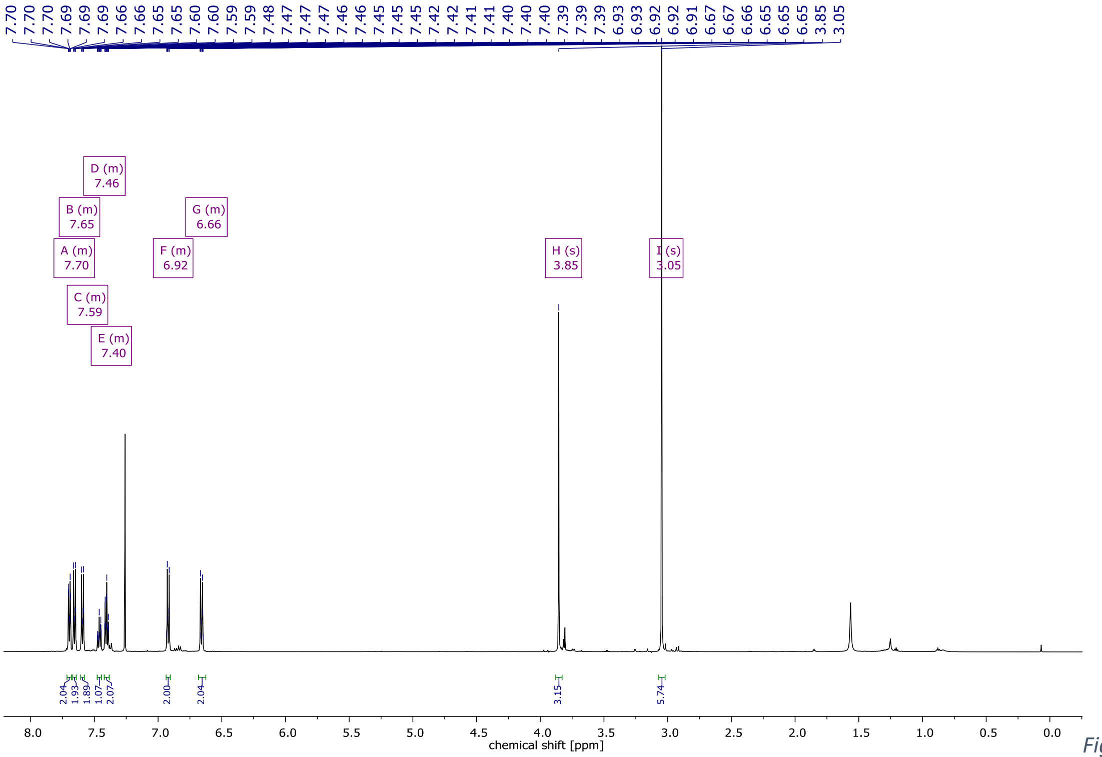

Figure S 83: ^{1}H NMR spectrum of **3** measured in $CDCl_3$ (600 MHz).

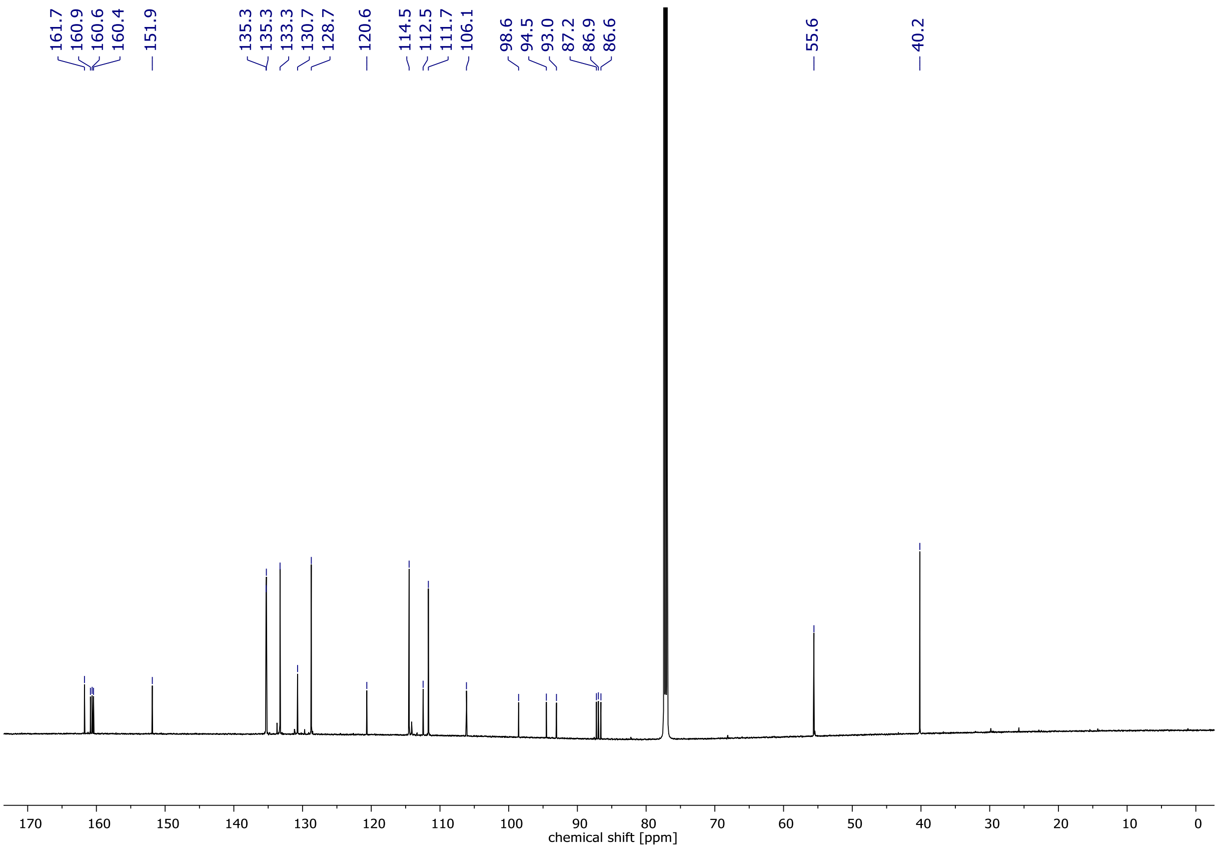

*Figure S 84: ^{13}C NMR Spectrum of **3** measured in $CDCl_3$ (151 MHz).*

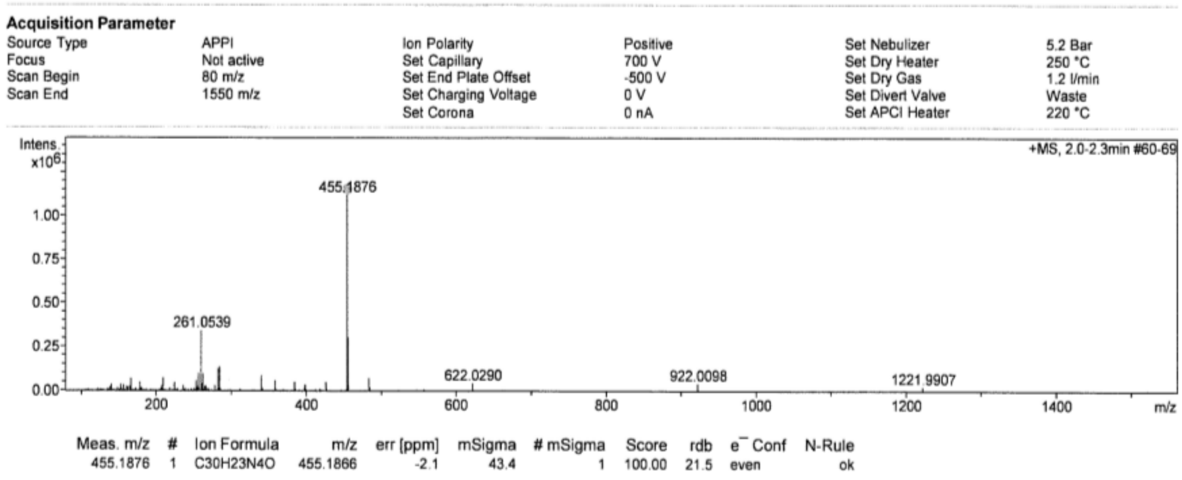

*Figure S 85: HRMS (APPI) of **3**.*

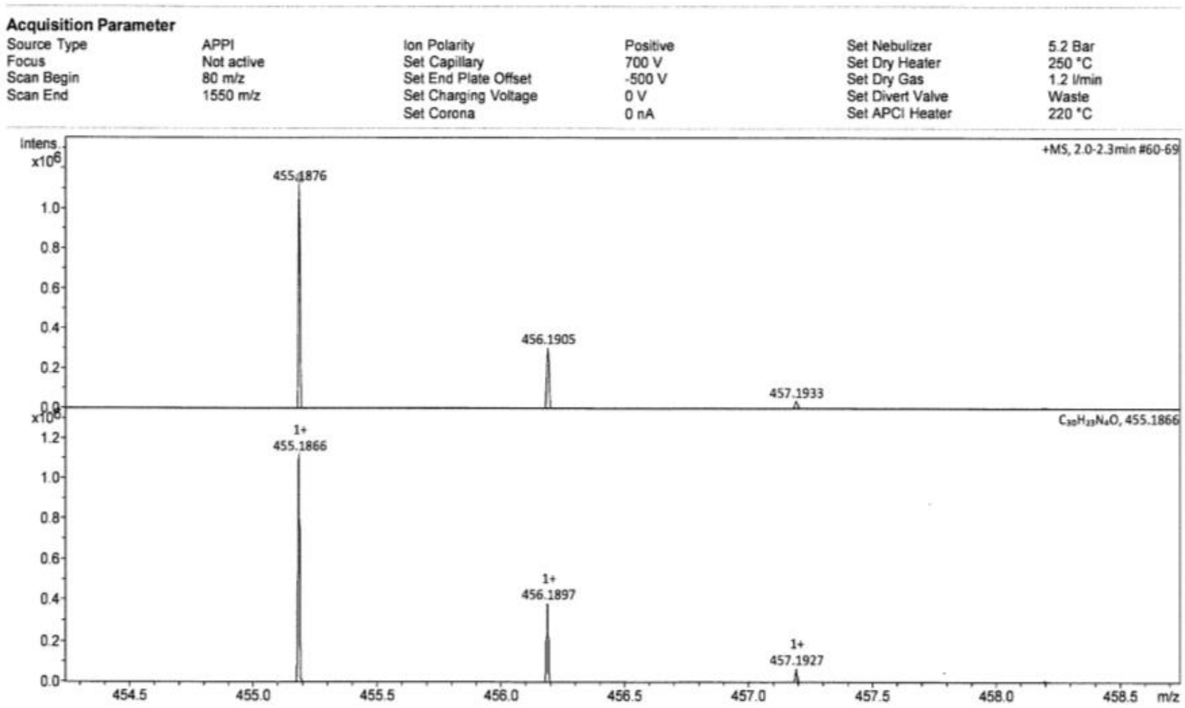

*Figure S 86: Zoom of HRMS (APPI) of **3**.*

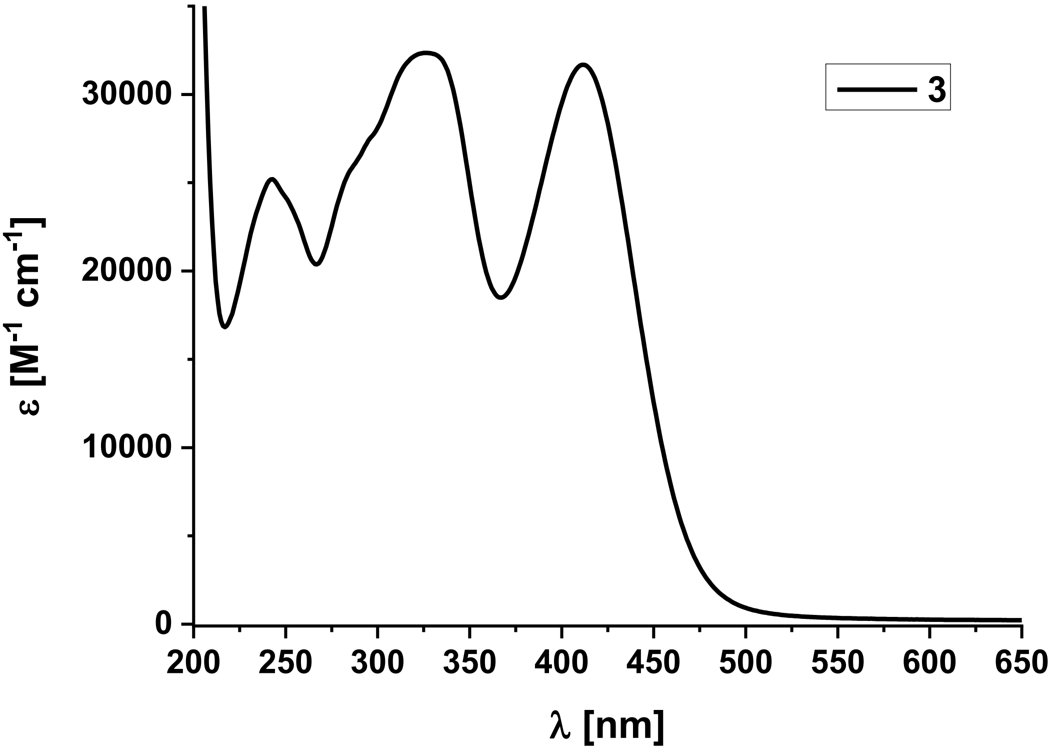

*Figure S 87: UV/Vis extinction spectrum of **3** measured in MeCN.*

3.5.2 **8**

Due to the complexity of **8**, including the presence of multiple diastereoisomers, integration of the signals is disturbed. 2D correlation spectra are recorded and shown below. Based on the limit of resolution and the multitude of overlapping signals, assignment of the single resonances is barely possible and therefore neglected.

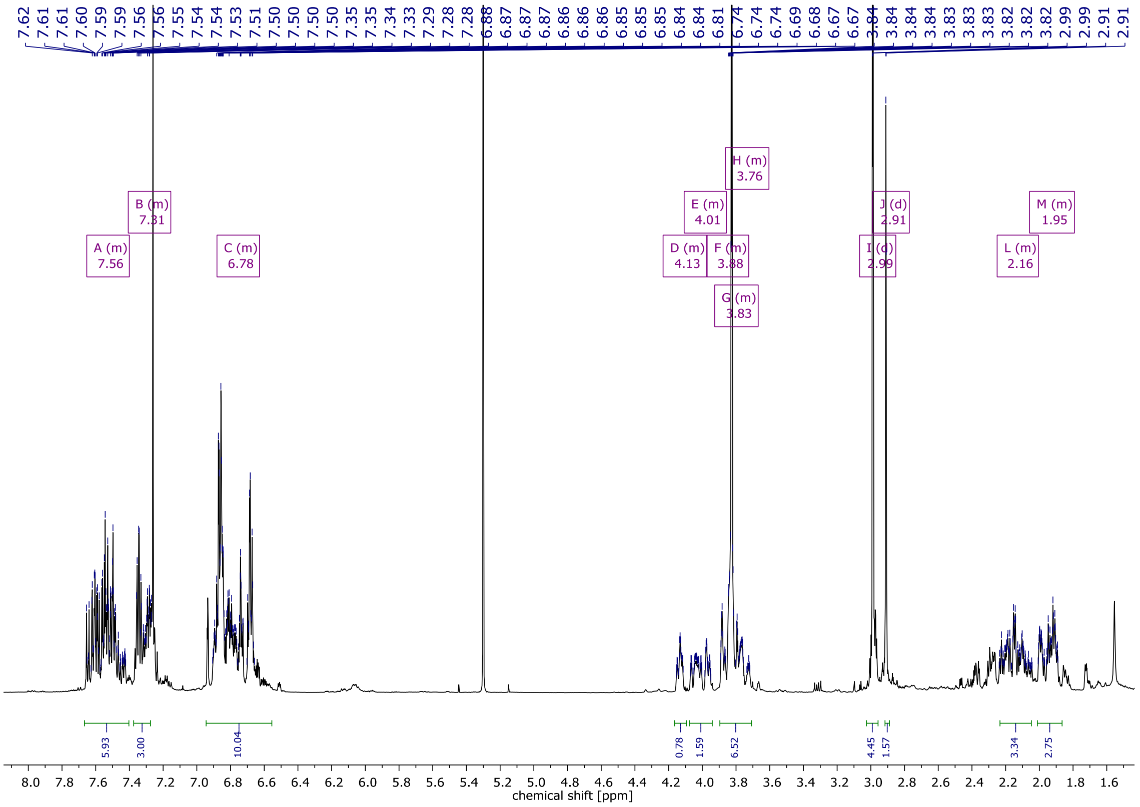

*Figure S 88: ^{1}H NMR spectrum of **8** measured in $CDCl_3$ (600MHz). The integration and peak picking in the aliphatic region is slightly disturbed by the presence of already switched **13** and not further identified small impurities.*

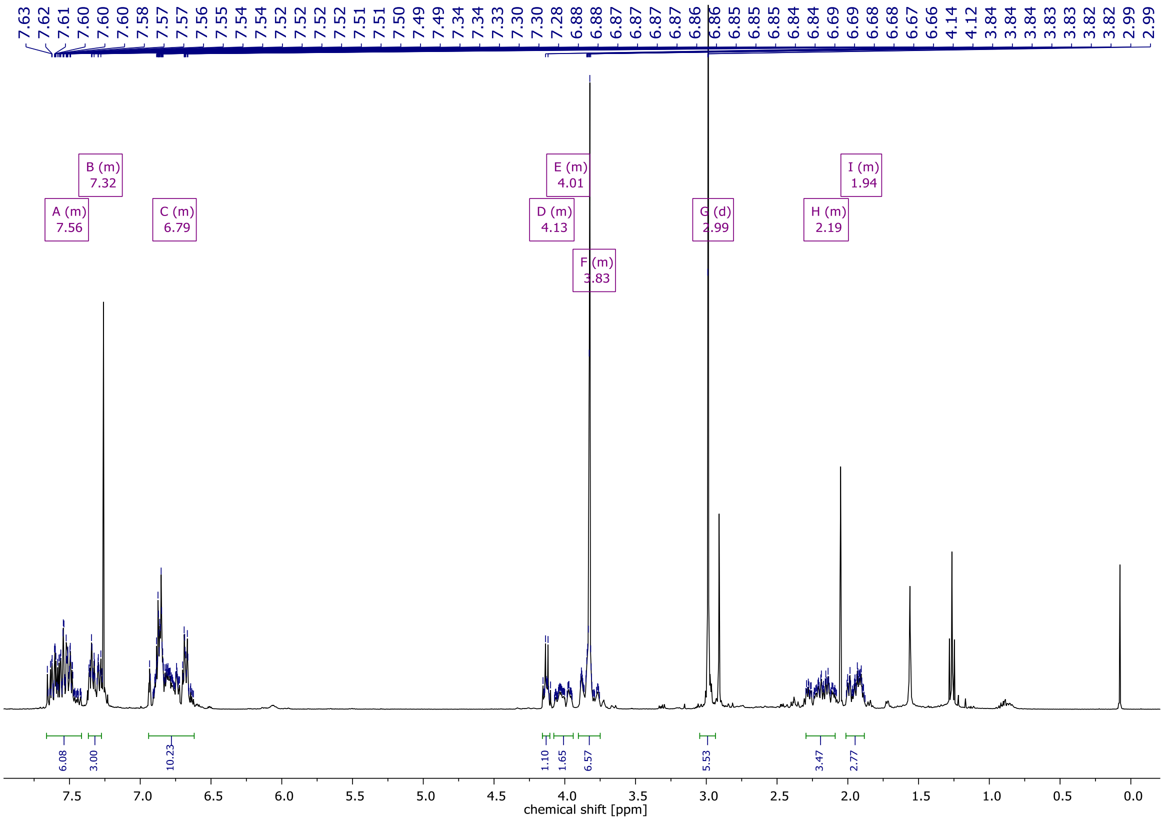

*Figure S 89: Additional ^{1}H NMR spectrum of **8** measured in $CDCl_3$ (400MHz) providing the best possible integration. The signals at 4.12, 2.05 and 1.25 belong to leftover EtOAc. The singlet signal at 2.91 ppm originated of an unknown impurity which disappears upon irradiation. The respective QC signal has a different shift and splitting (triplet at 2.90 ppm) compared to the impurity.*

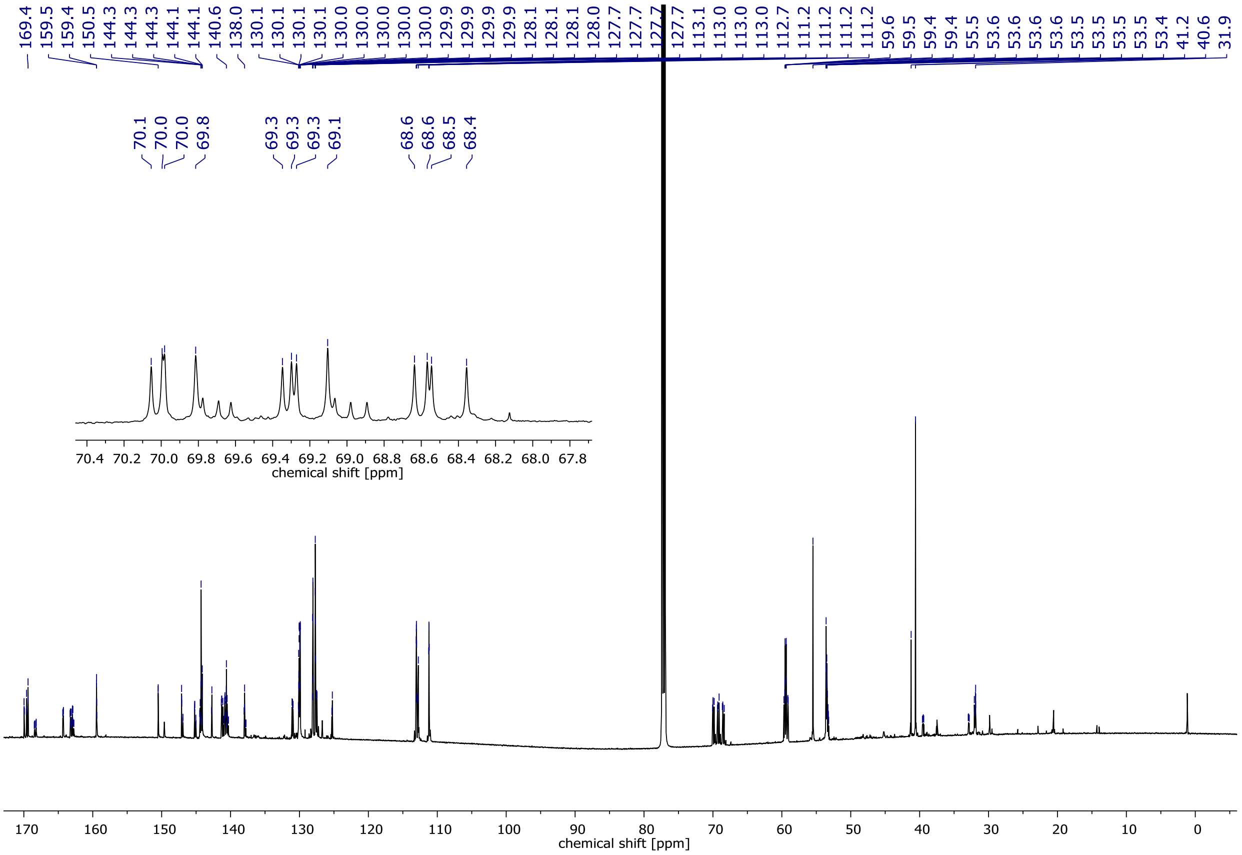

*Figure S 90: ^{13}C NMR Spectrum of **8** measured in $CDCl_3$ (151 MHz). Every carbon signal splits as 4-fold signal set corresponding to the number of available diastereoisomer pairs. The minor signals next to the picked peaks belong to already switched QC species (compare below).*

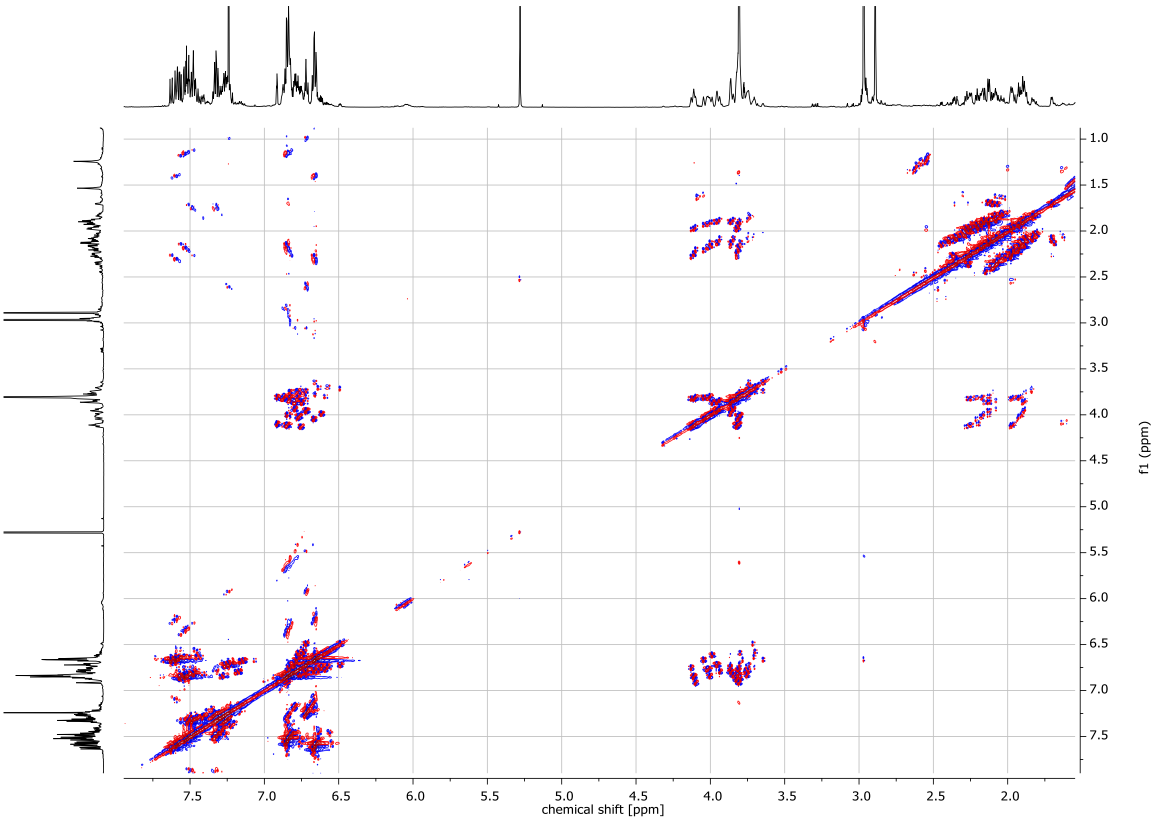

*Figure S 91: COSY spectrum of **8** measured in $CDCl_3$ (600 MHz).*

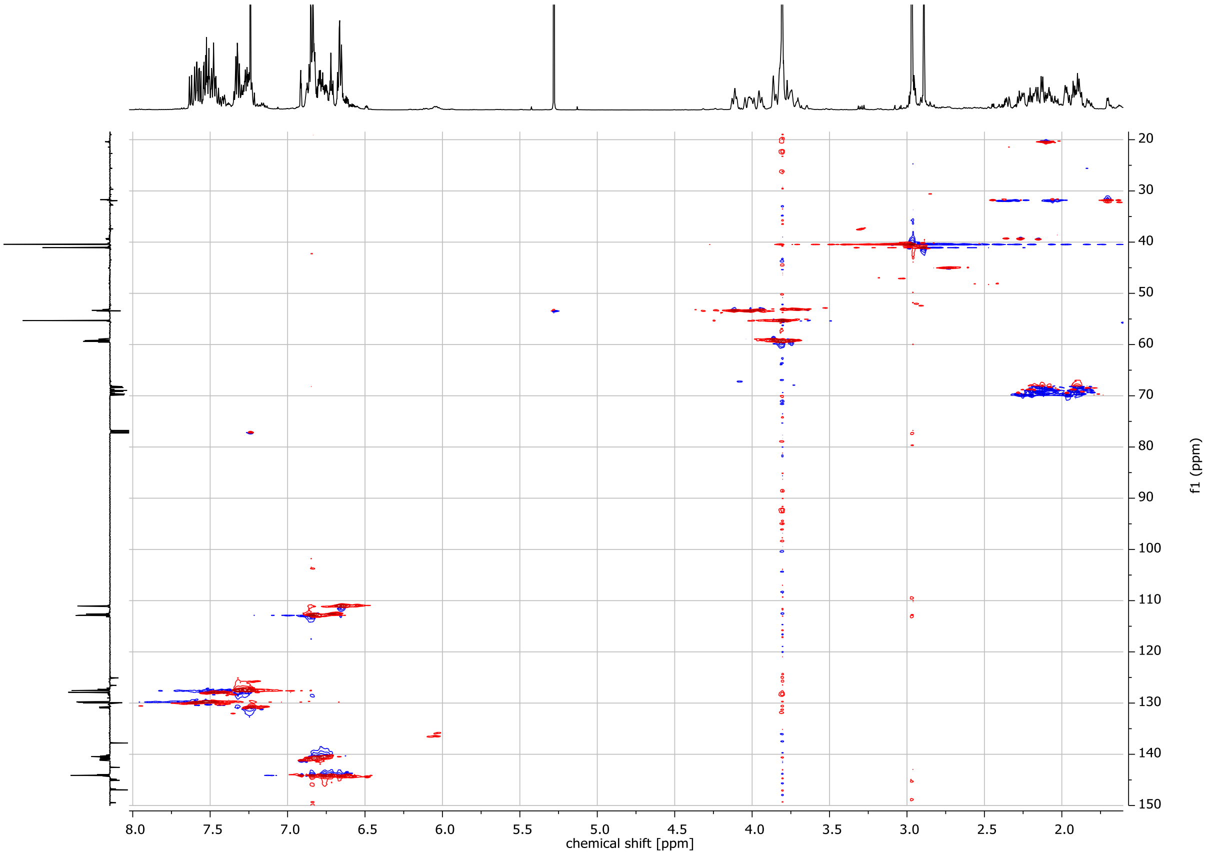

*Figure S 92: HSQC spectrum of **8** measured in $CDCl_3$ (600 MHz).*

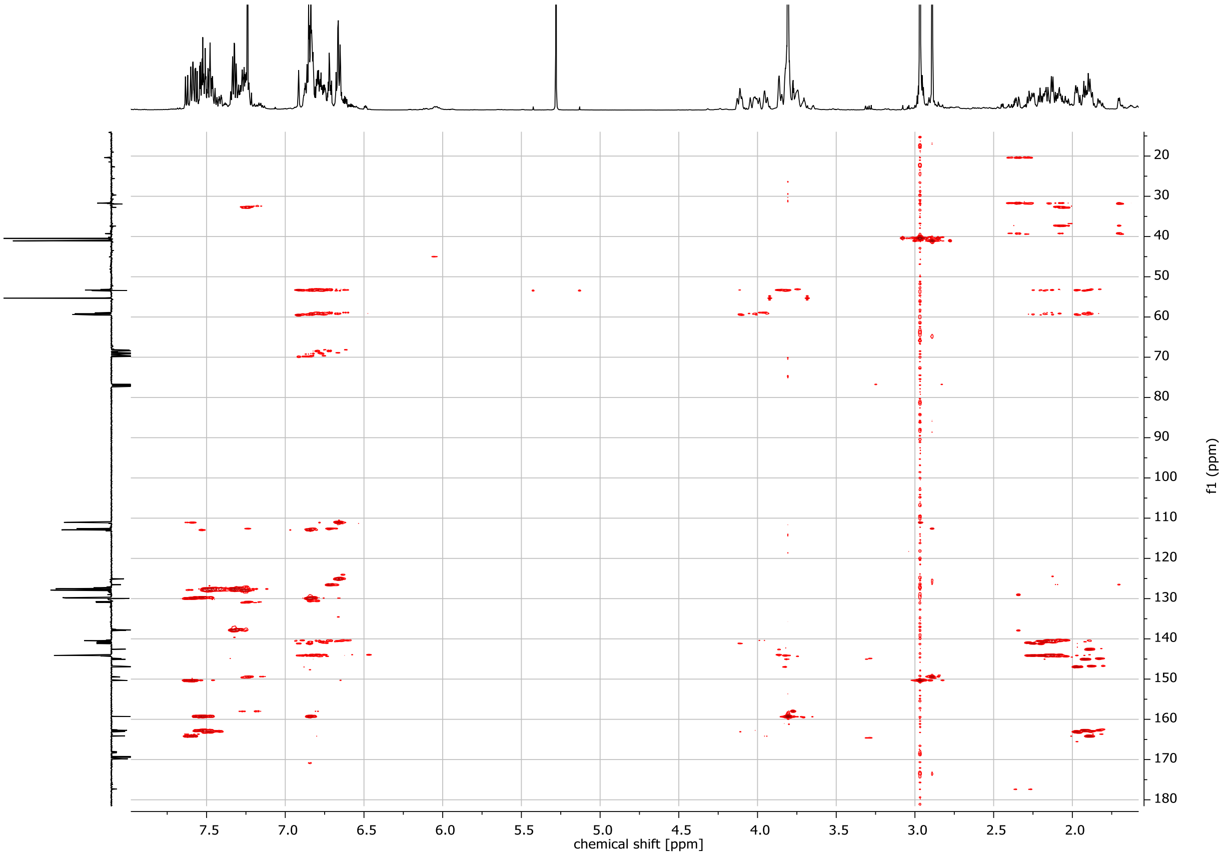

*Figure S 93: HMBC spectrum of **8** measured in $CDCl_3$ (600 MHz).*

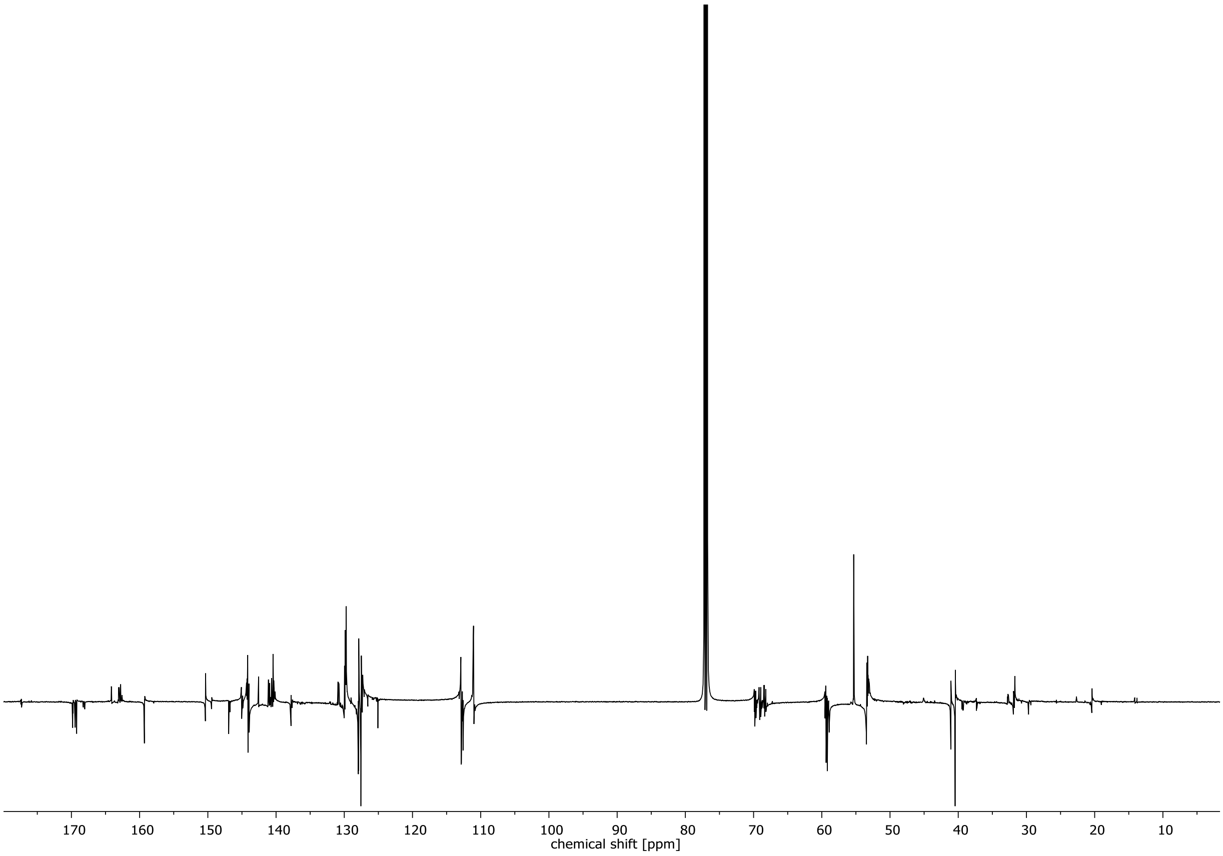

*Figure S 94: Deptq spectrum of **8** measured in $CDCl_3$ (600 MHz).*

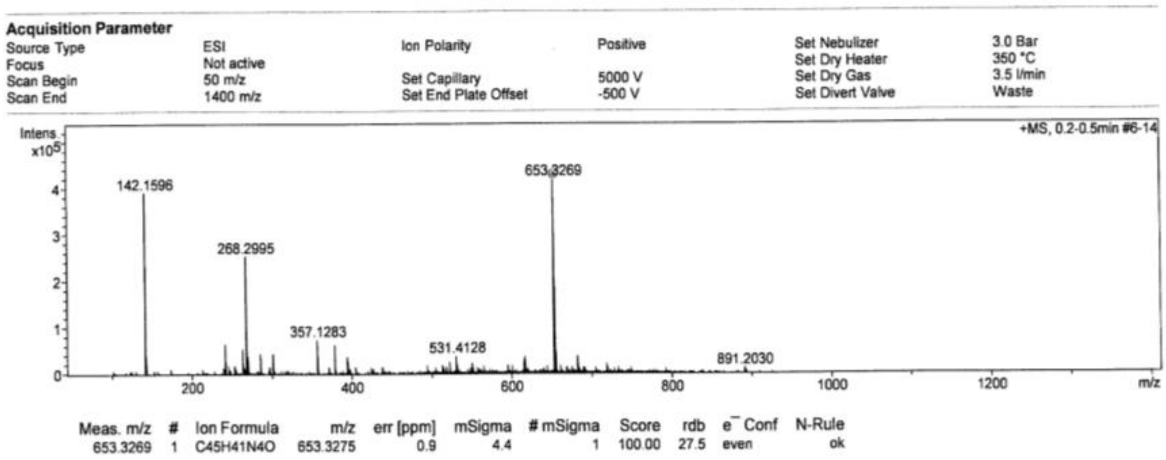

*Figure S 95: HRMS (APPI) of **8**.*

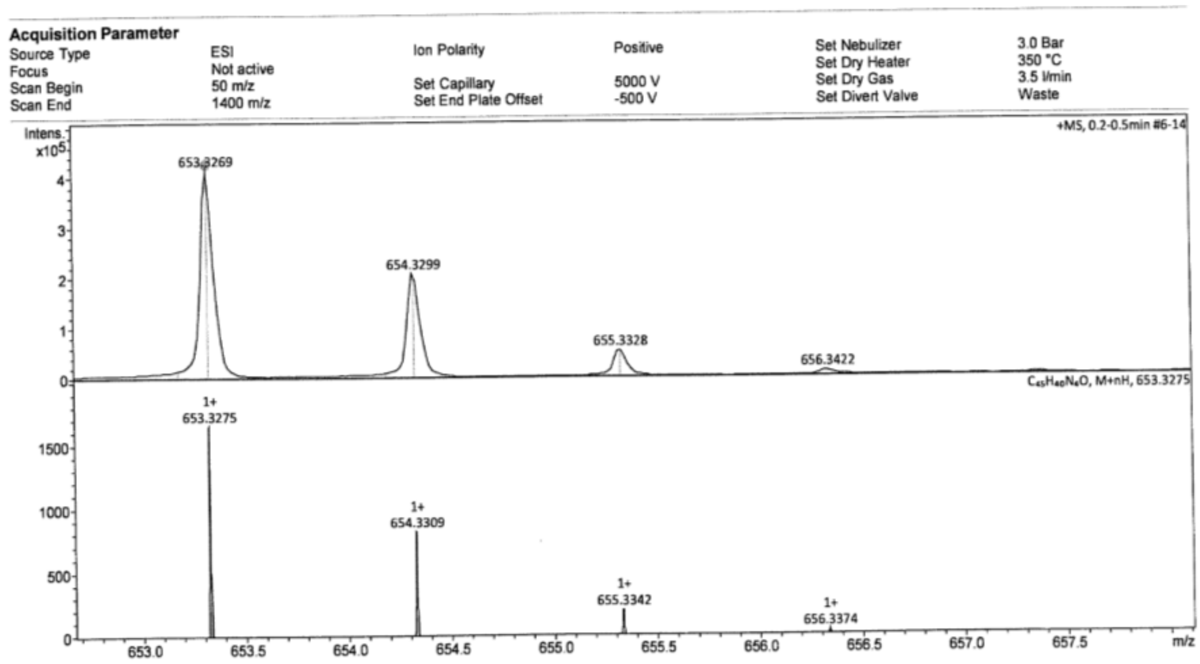

*Figure S 96: Zoom of HRMS (APPI) of **8**.*

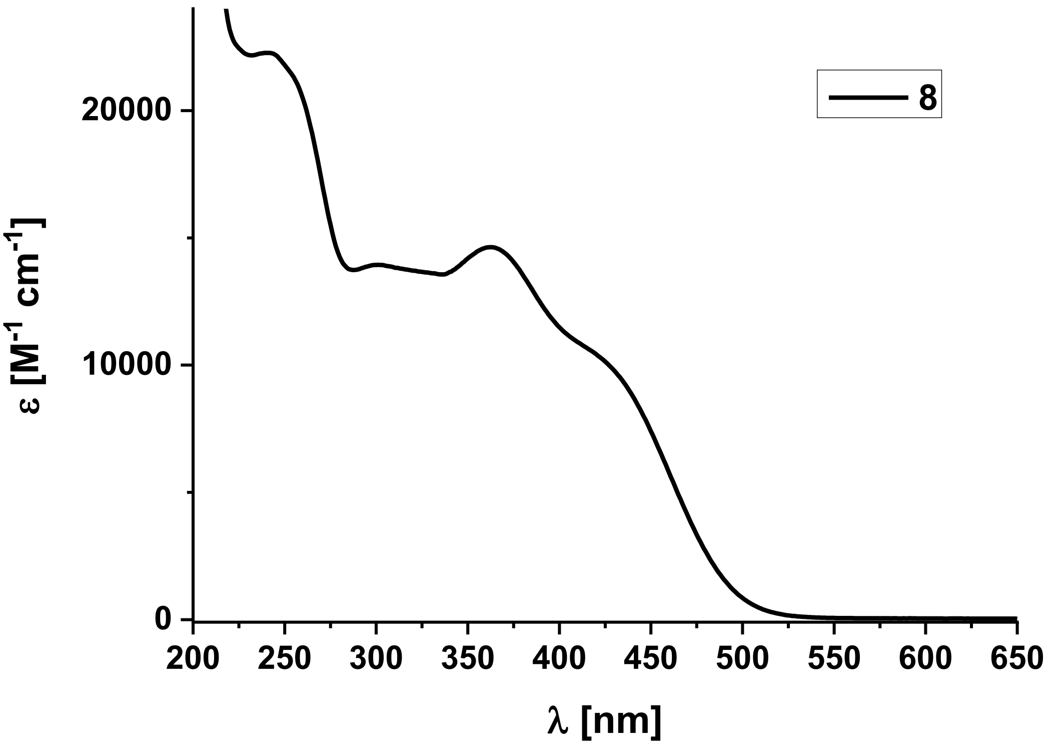

*Figure S 97: UV/Vis extinction spectrum of **8** measured in MeCN.*

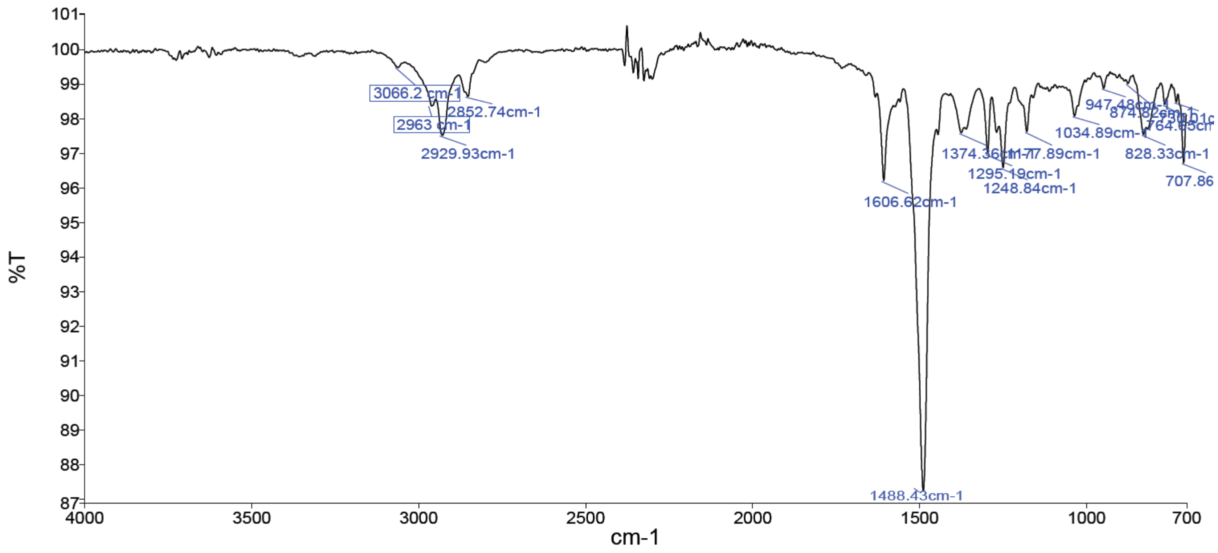

*Figure S 98: ATIR spectrum of **8**.*

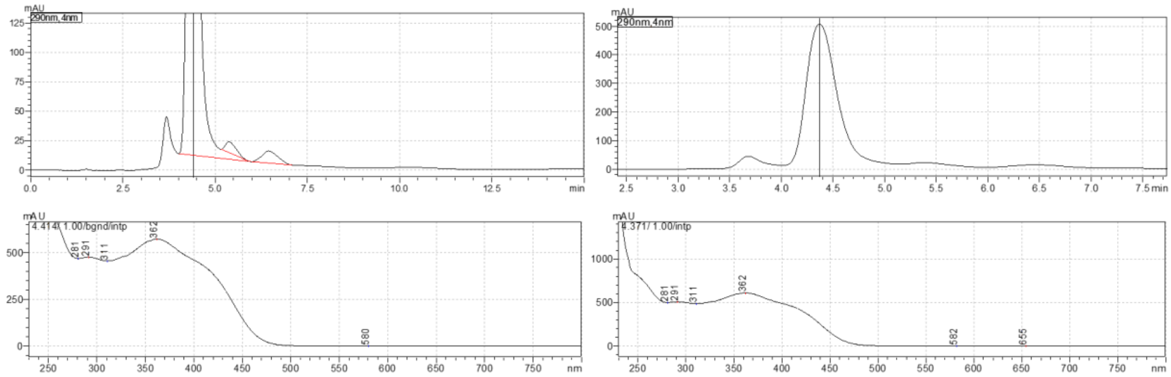

Peak number	1	2	3	4
Retention time [min]	3.681	4.371	5.376	6.452
Area [%]	4.513	91.176	1.623	2.689

*Figure S 99: HPLC elugram and corresponding UV/Vis spectrum of **8**. Analysis performed with a nucleosil 100-5 column using a mixture of 95%/5% of hexane/ethyl acetate with a flowrate of 2 ml/min. 30 µl of a saturated sample solution was injected. On top: Obtained elugram using the mentioned method with a zoom of the significant section on the right, middle: The absorption spectra of the main peak bottom: A table describing the integrated areas corresponding to the respective peaks. The combined purity of the isomers is > 95%, claiming **8** as sufficiently purified.*

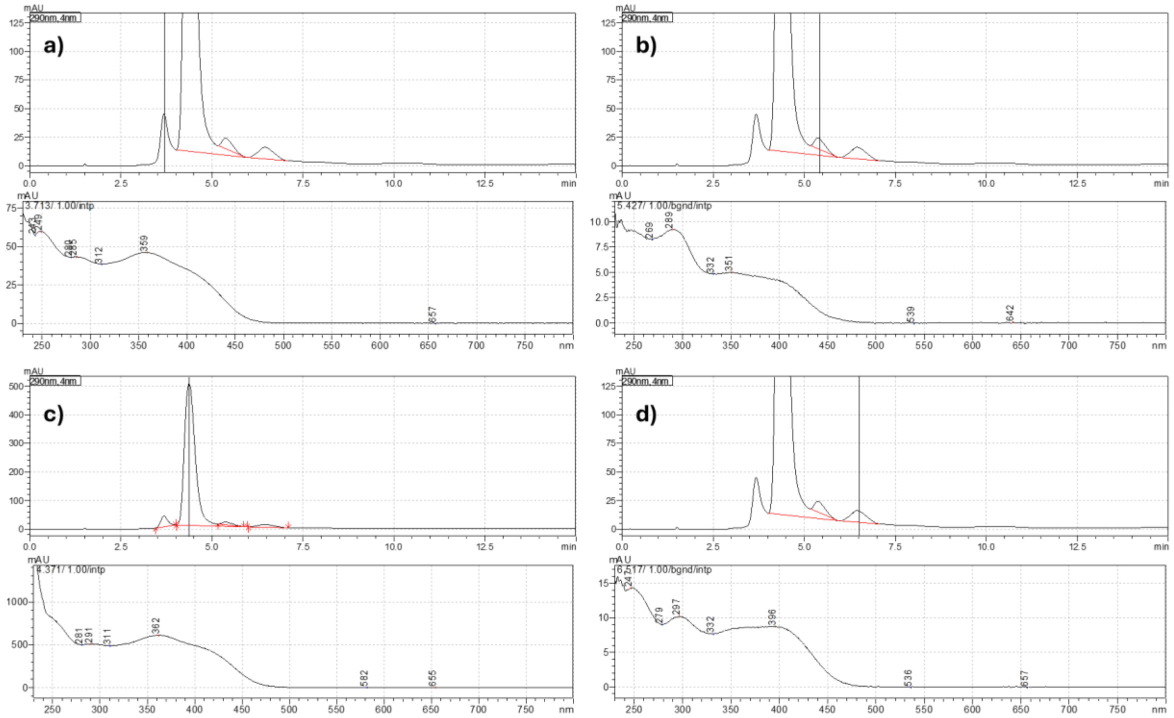

*Figure S 100: Corresponding UV/Vis absoprtion spectra of the individual picked peaks of the elugram of **8**.*

For the first (a) and second peak (c), the absorption spectra are completely similar and equivalent to the extinction spectrum of **8** measured in MeCN (see above). Furthermore, for both signals, the main peak found in HRMS measurements corresponds to compound **8**. Therefore, both signals probably correspond to different diastereoisomers. The absorption spectra for the peaks depicted in b) and d) slightly differ and might correspond to negligible impurities rather than diastereoisomers.

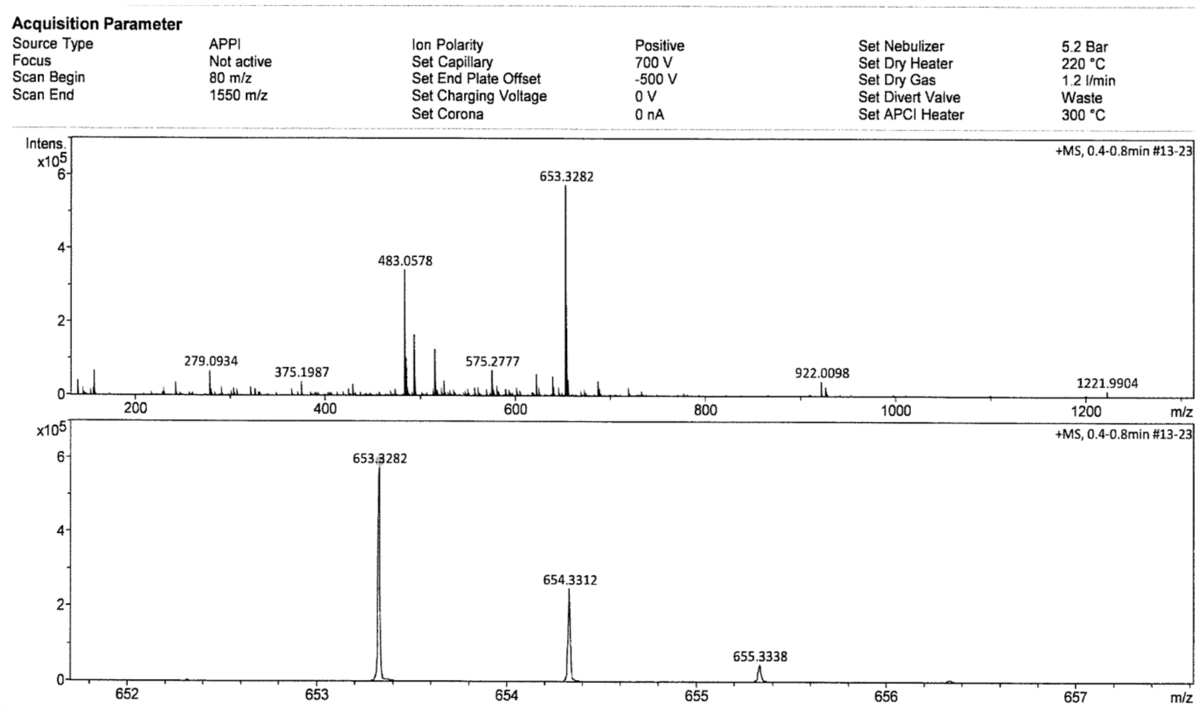

*Figure S 101: MS corresponding to HPLC spot a) of **8**.*

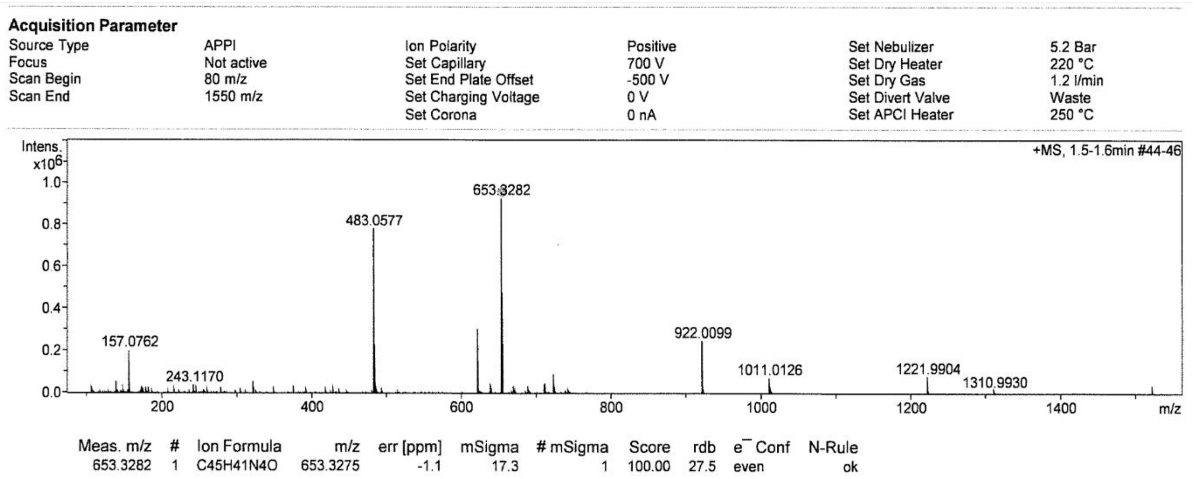

*Figure S 102: MS corresponding to HPLC spot c) of **8**.*

3.5.3 **13**

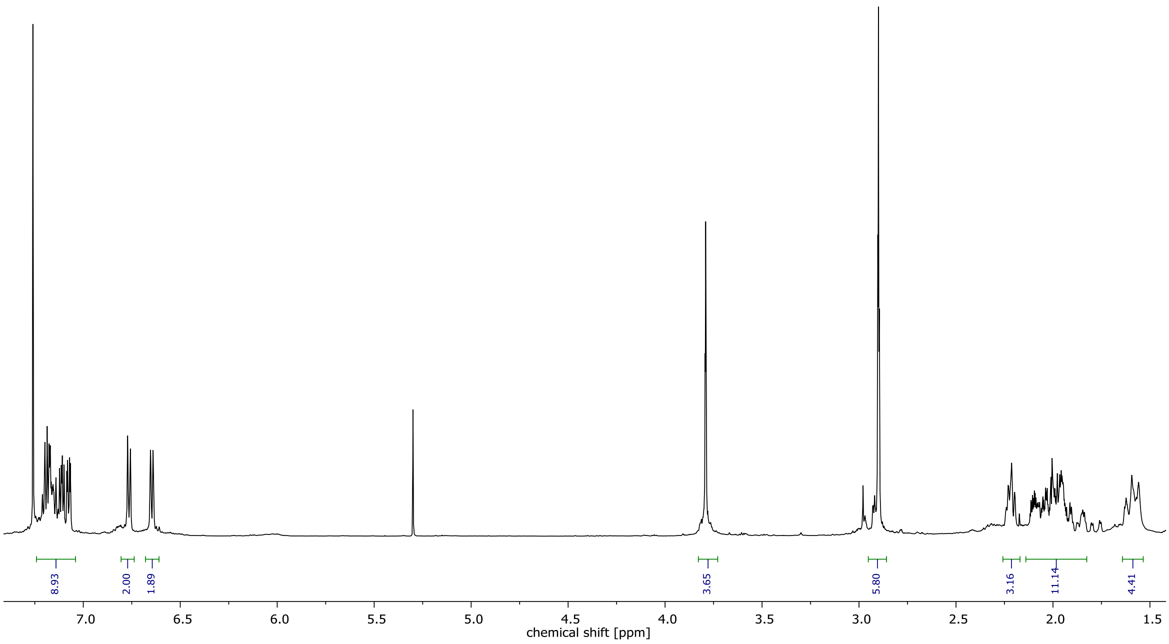

*Figure S 103: ^{1}H NMR spectrum of **13** measured in $CDCl_3$ (600 MHz).*

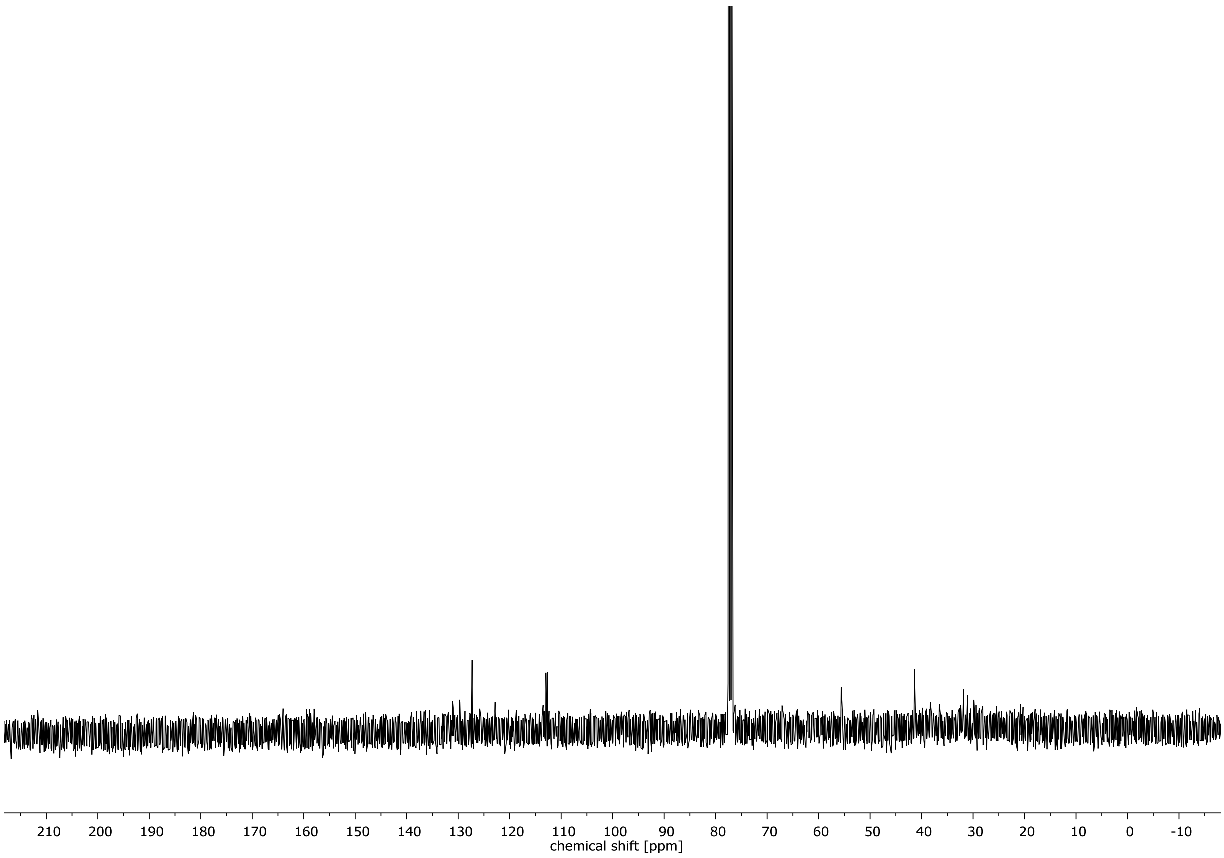

*Figure S 104: ^{13}C NMR spectrum of **13** measured in $CDCl_3$ (101 MHz). For better visibility and intensity of the signals, compare to the deptq spectrum of **13** below.*

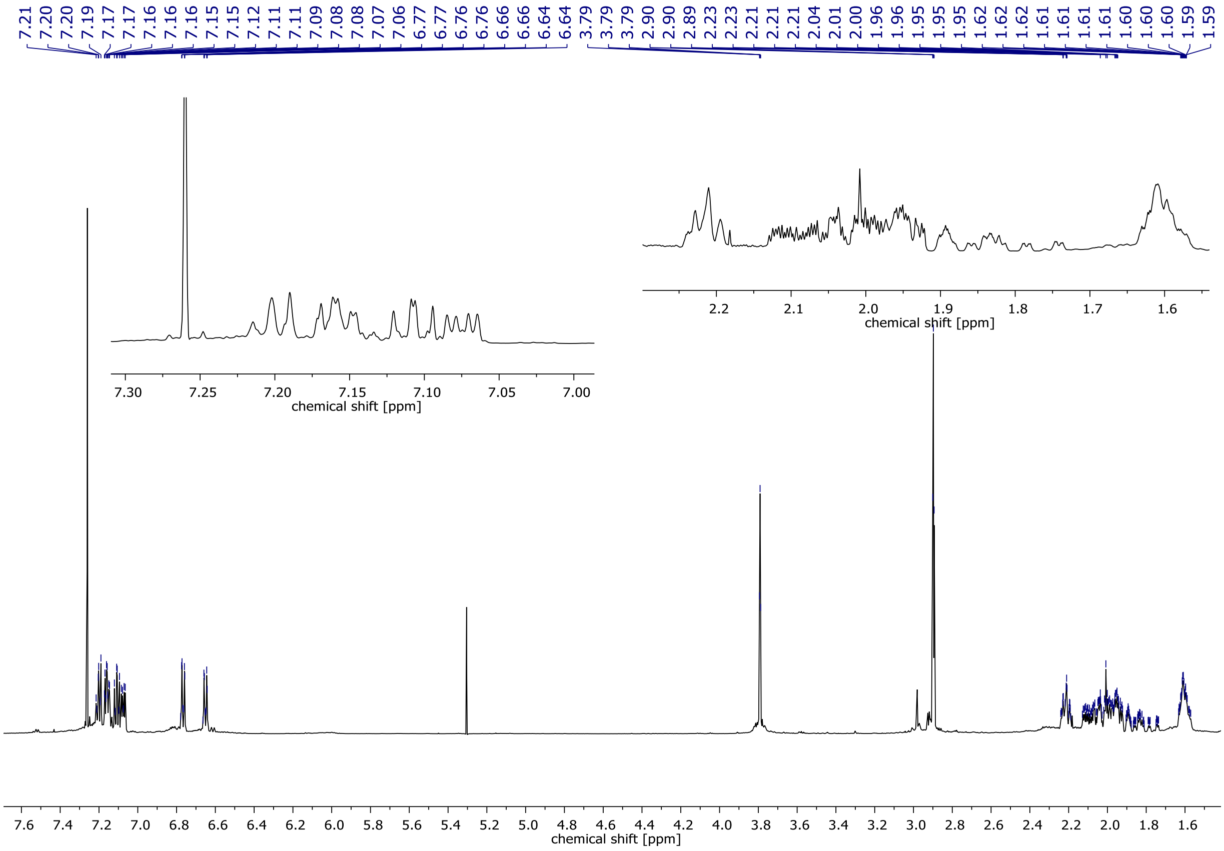

Figure S 105: ^{1}H NMR spectrum of **13** measured in $CDCl_3$ (600 MHz, 10 °C) including zoom of the significant sections.

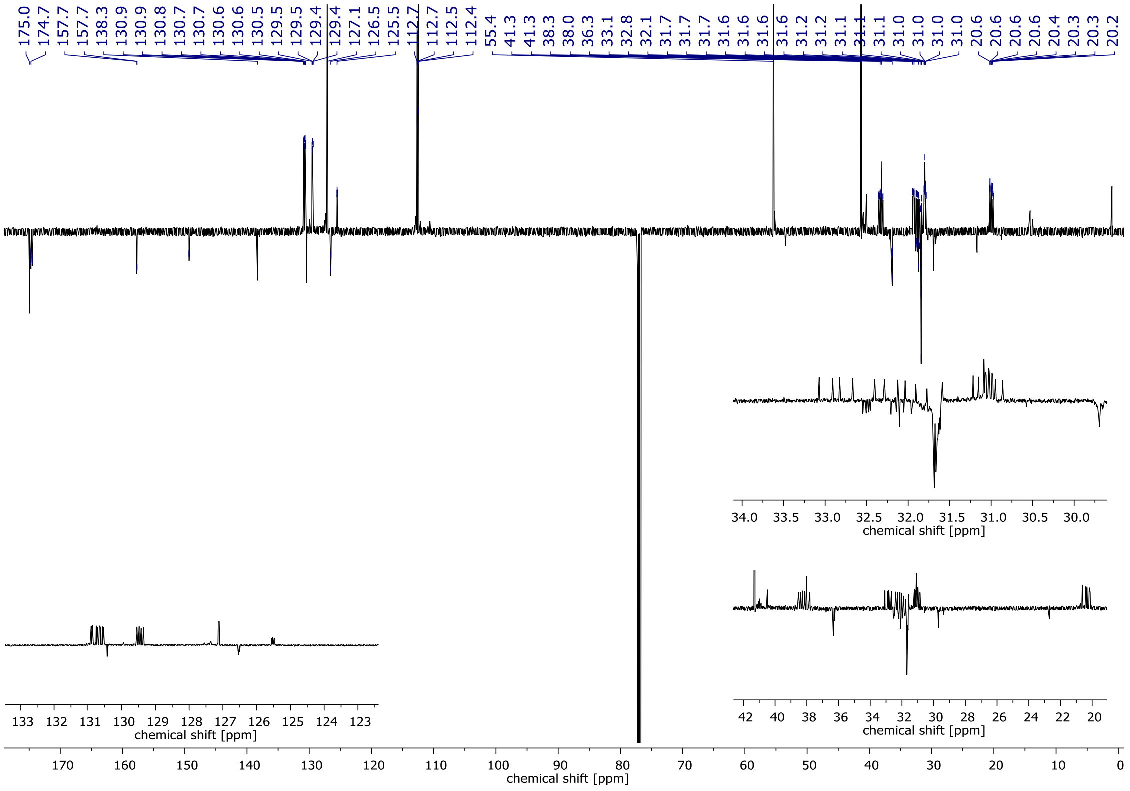

Figure S 106: Deptq spectrum of **13** measured in $CDCl_3$ (151 MHz, 10 °C) including zooms of the significant sections.

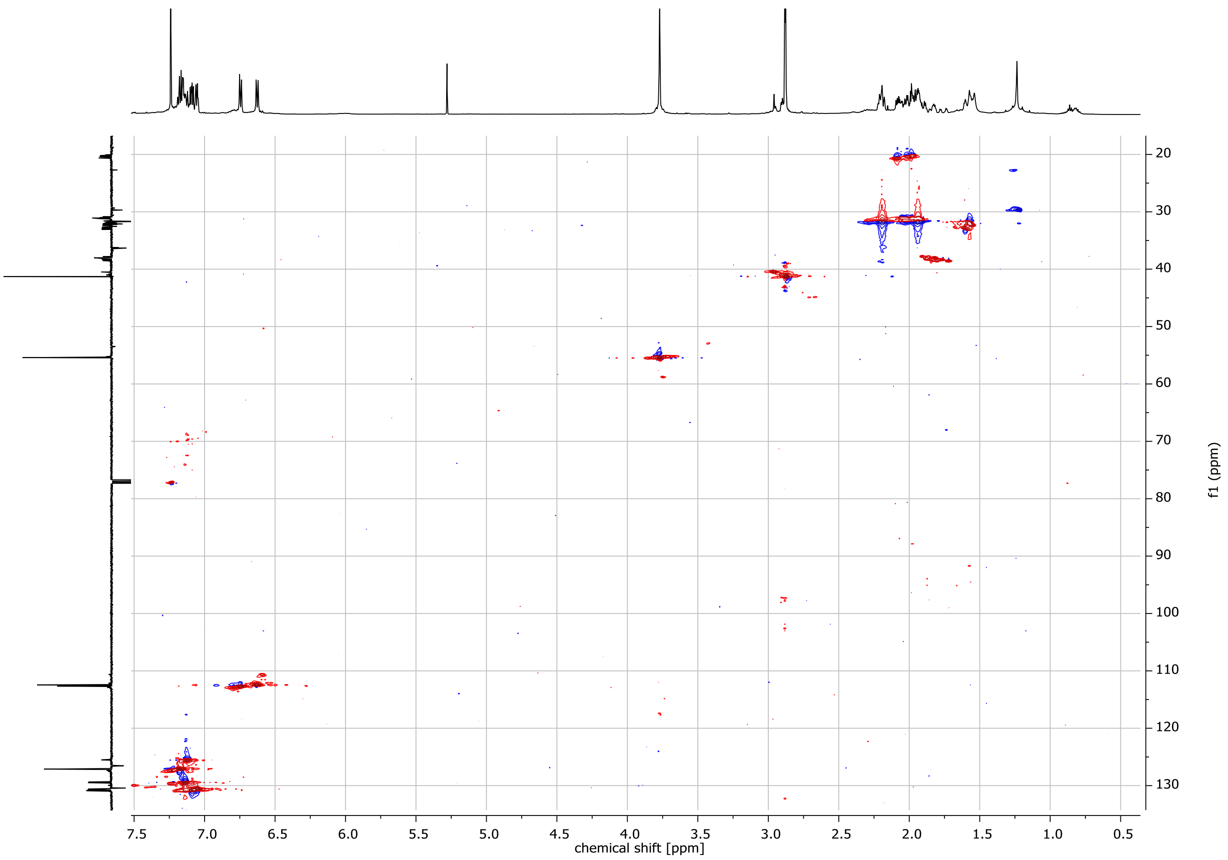

*Figure S 107: HSQC spectrum of **13** measured in $CDCl_3$ at 10 °C (600 MHz).*

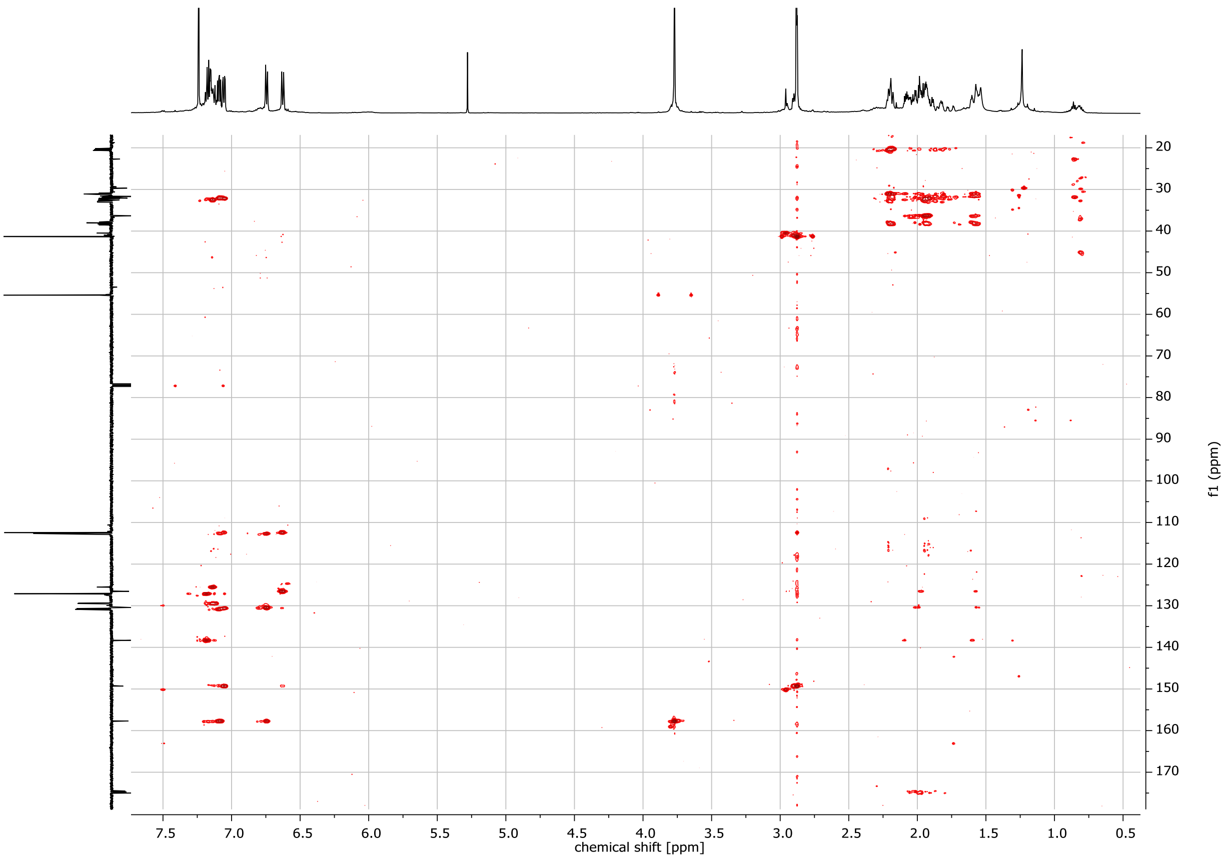

*Figure S 108: HMBC spectrum of **13** measured in $CDCl_3$ at 10 °C (600 MHz).*

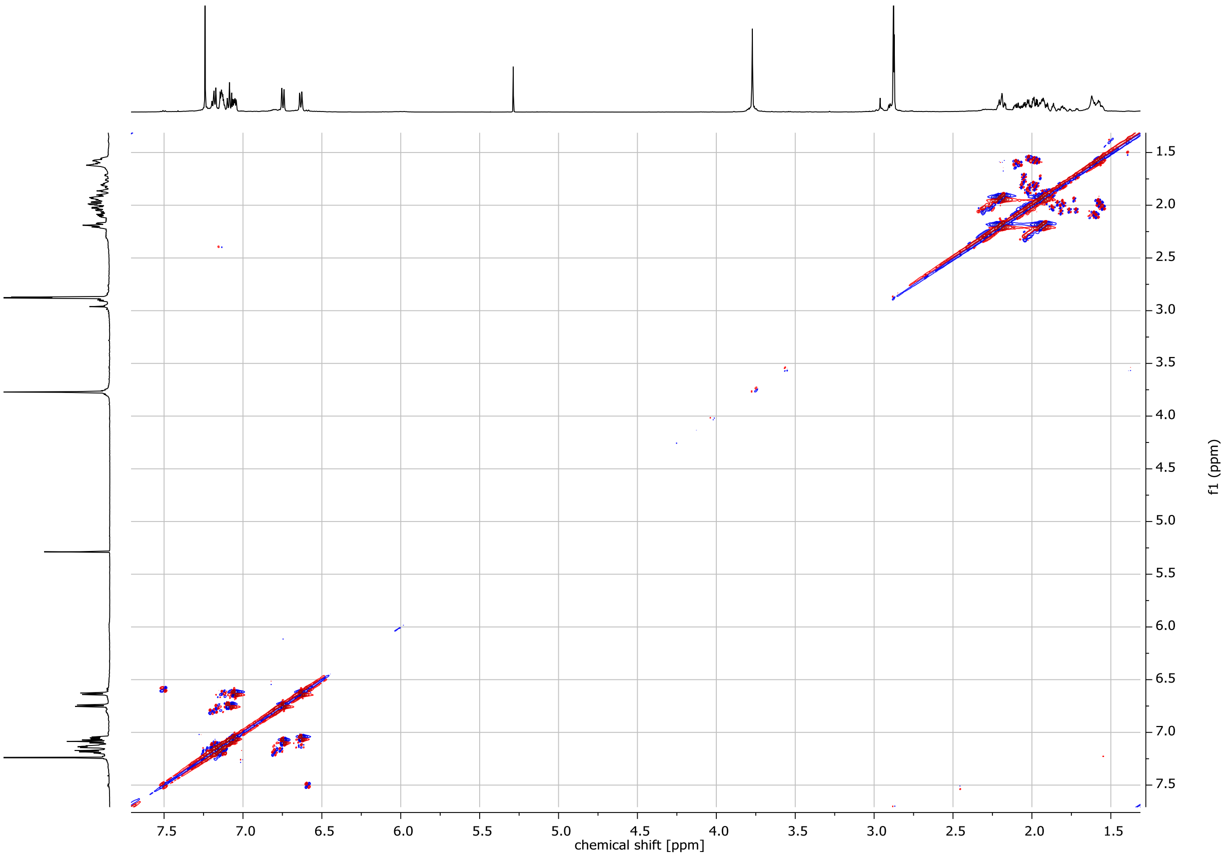

*Figure S 109: COSY spectrum of **13** measured in $CDCl_3$ at 10 °C (600 MHz).*

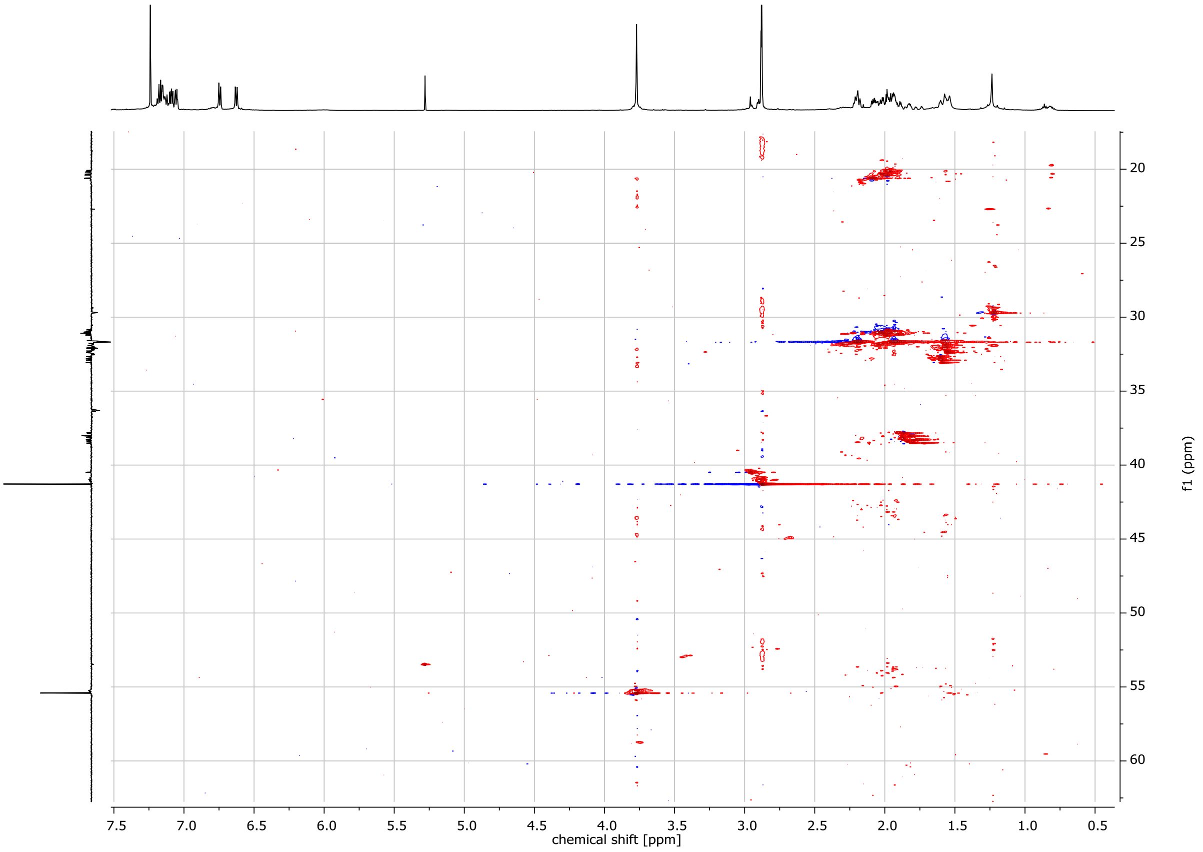

*Figure S 110: 2D selective HSQC spectrum of **13** measured in $CDCl_3$ at 10 °C (600 MHz).*

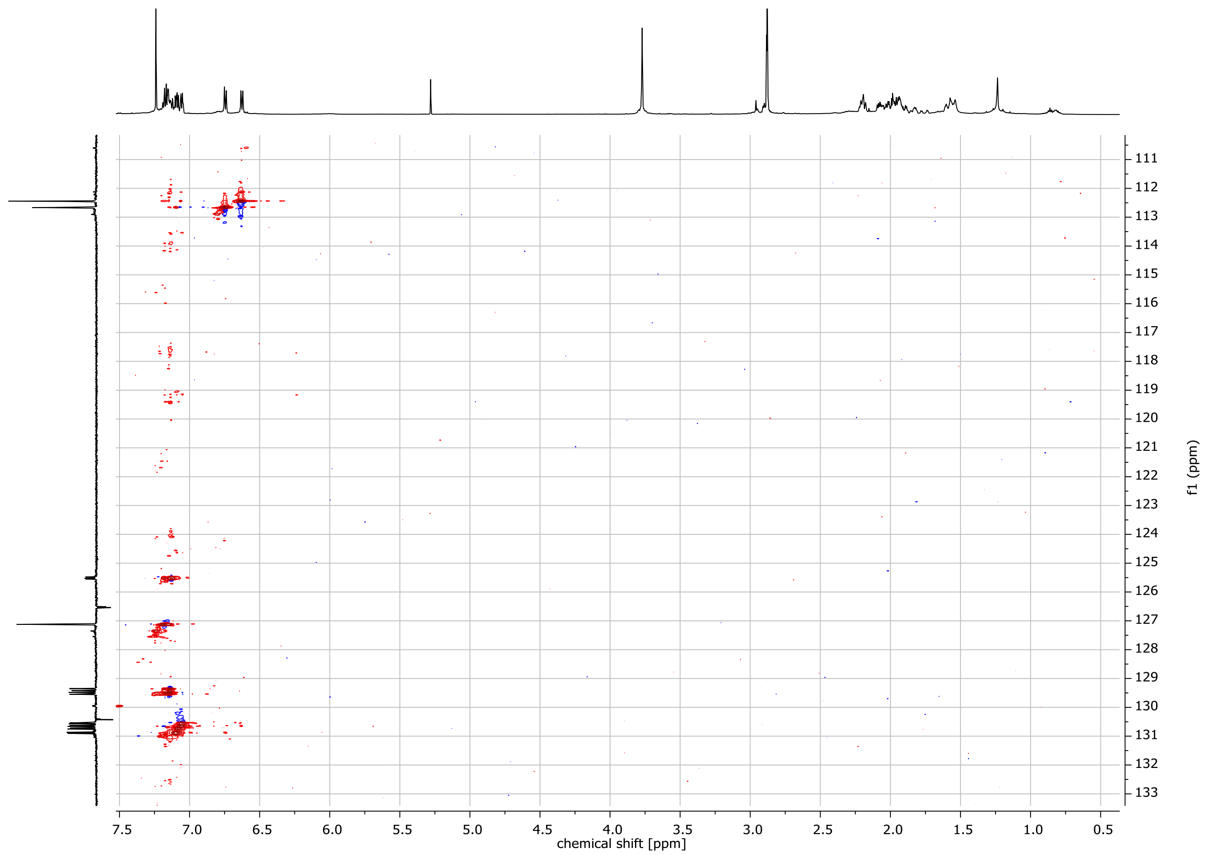

*Figure S 111: 2D selective HSQC spectrum of **13** measured in $CDCl_3$ at 10 °C (600 MHz).*

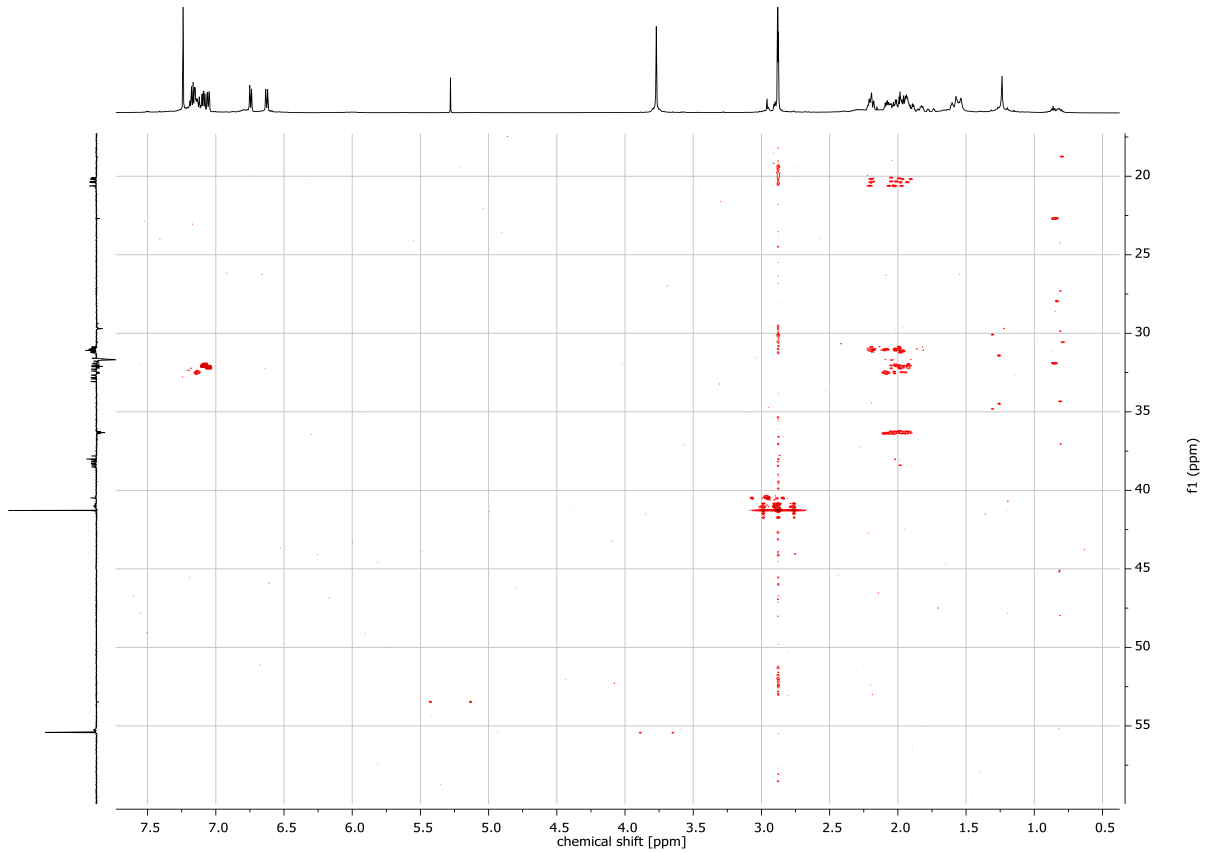

*Figure S 112: 2D selective HMBC spectrum of **13** measured in $CDCl_3$ at 10 °C (600 MHz).*

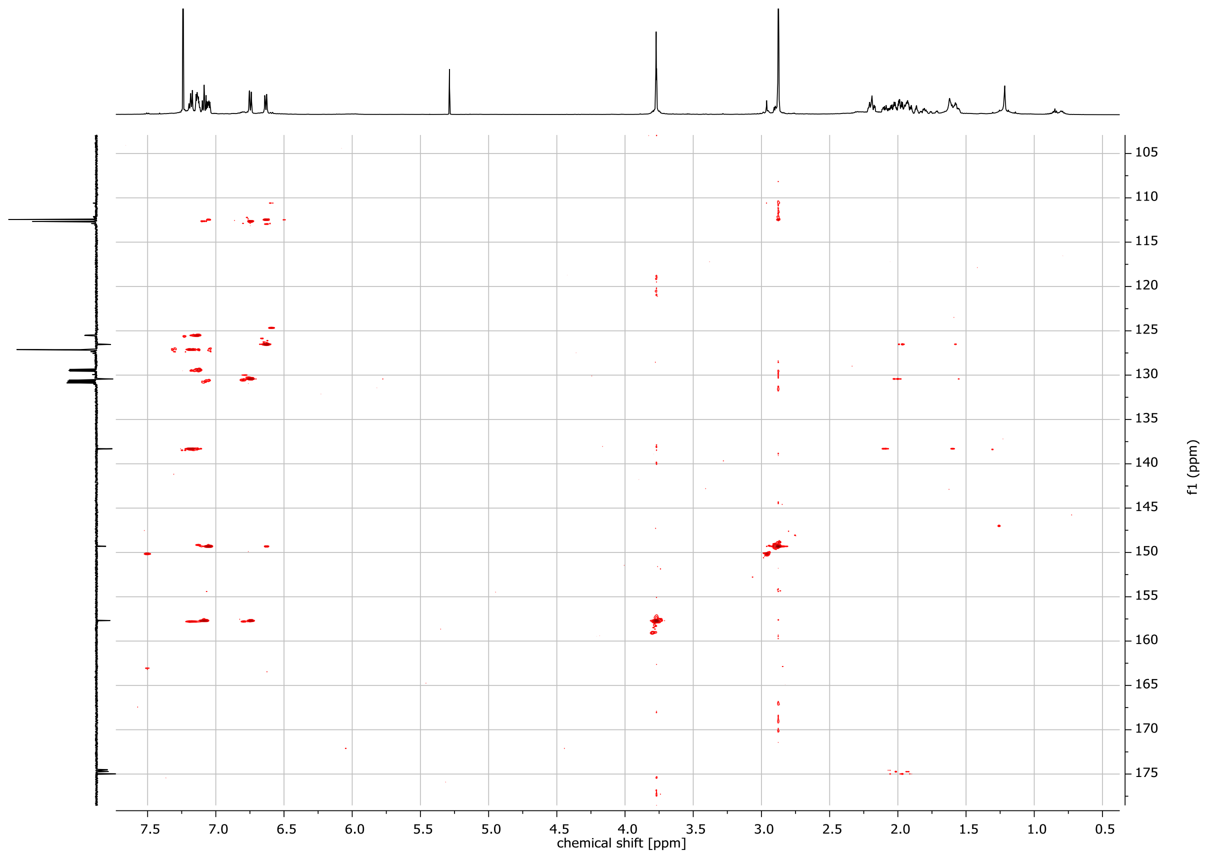

*Figure S 113: 2D selective HMBC spectrum of **13** measured in CDCl3 at 10 °C (600 MHz).*

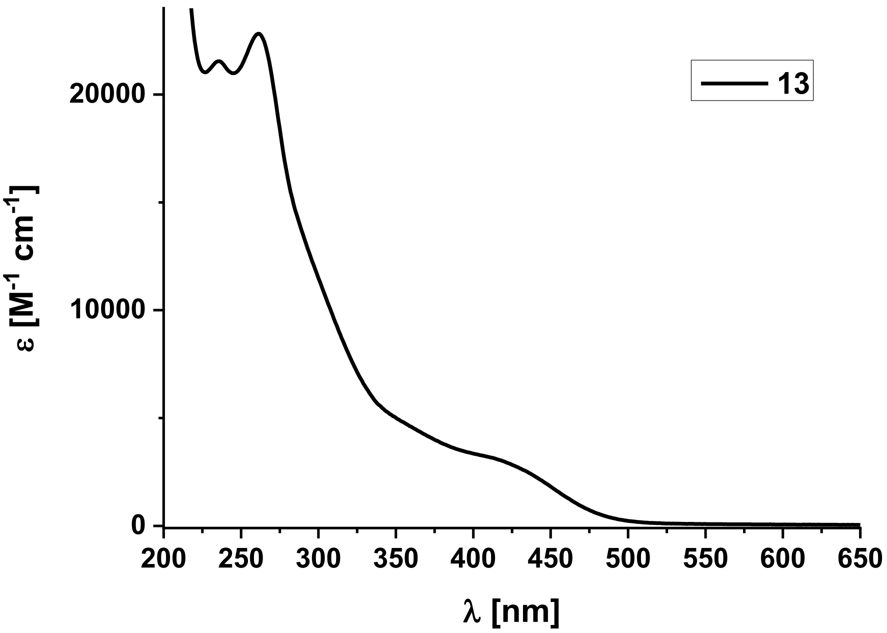

*Figure S 114: UV/Vis extinction spectrum of **13** measured in MeCN.*

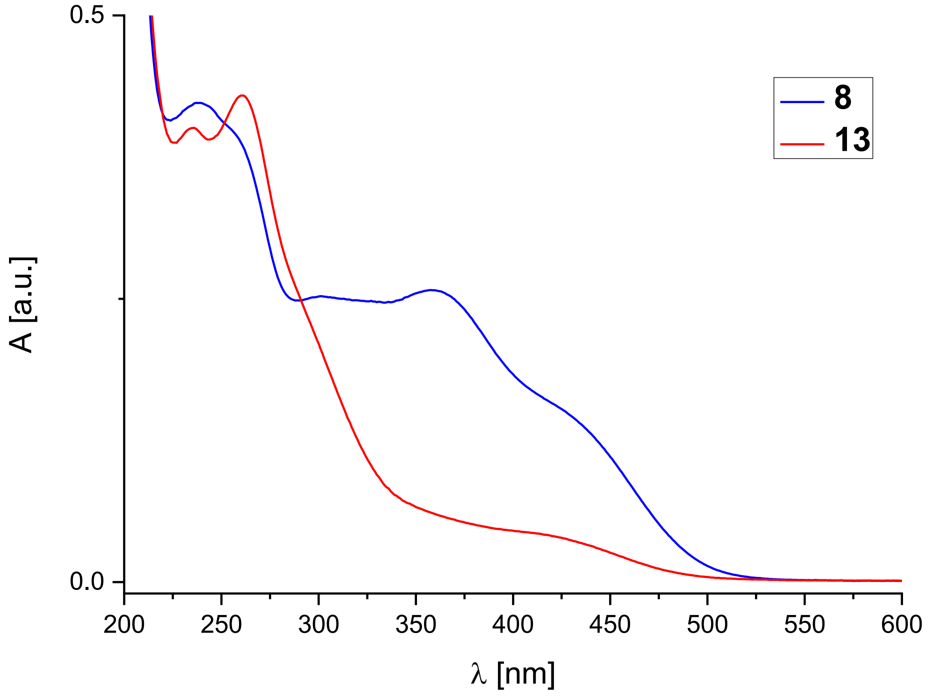

*Figure S 115: UV/Vis spectra of QC **13** and NBD **8** measured in MeCN.*

3.6 Additional acetylene derivatives

3.6.1 **1b**

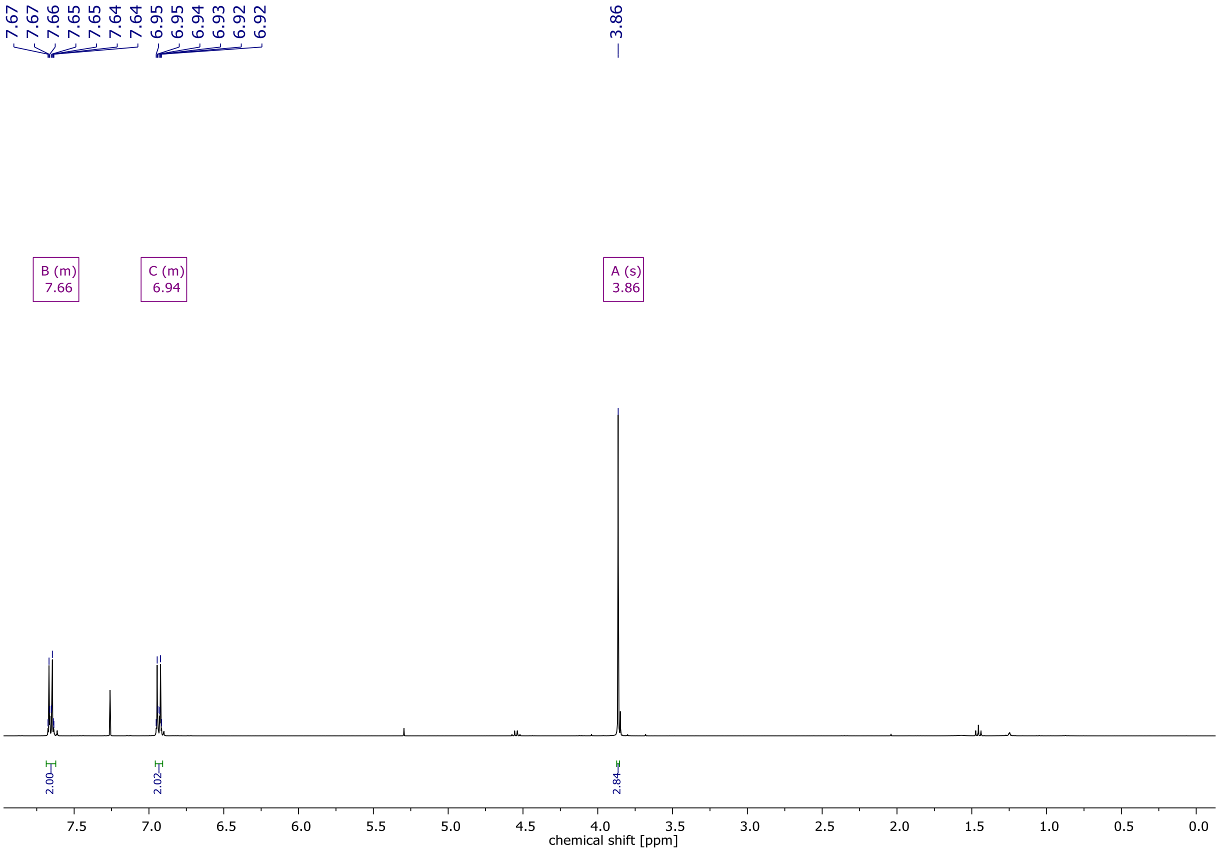

*Figure S 116: ^{1}H NMR spectrum of **1b** measured in $CDCl_3$ (400 MHz).*

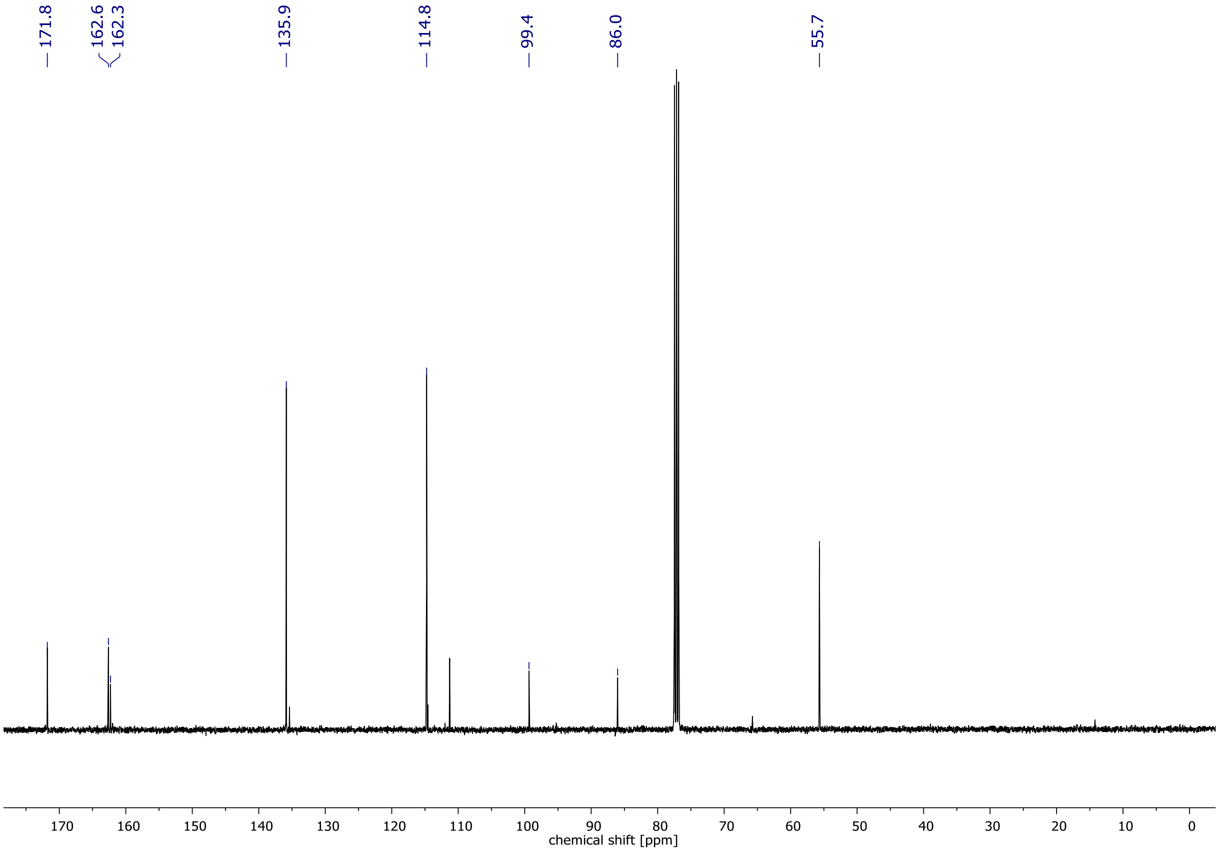

*Figure S 117: ^{13}C NMR Spectrum of **1b** measured in $CDCl_3$ (101 MHz).*

Acquisition Parameter

Source Type	APPI	Ion Polarity	Positive	Set Nebulizer	5.2 Bar
Focus	Not active	Set Capillary	700 V	Set Dry Heater	220 °C
Scan Begin	80 m/z	Set End Plate Offset	-500 V	Set Dry Gas	1.2 l/min
Scan End	1550 m/z	Set Charging Voltage	0 V	Set Divert Valve	Waste
		Set Corona	0 nA	Set APCI Heater	250 °C

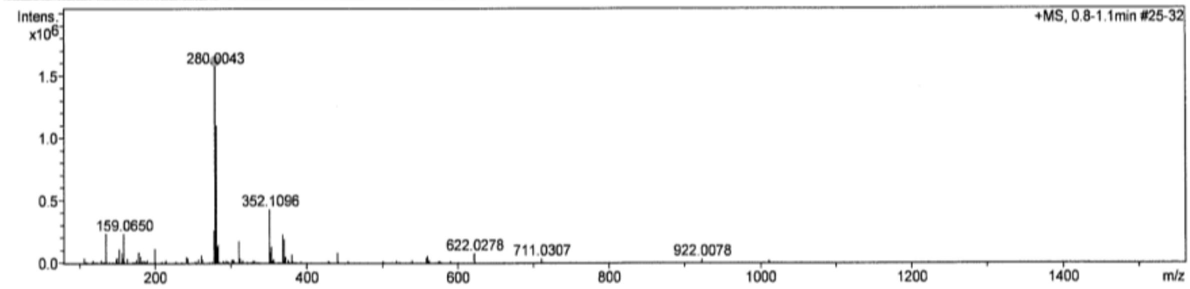

Meas. m/z	#	Ion Formula	m/z	err [ppm]	mSigma	# mSigma	Score	rdb	e⁻ Conf	N-Rule
280.0043	1	C12H8Cl2N3O	280.0039	-1.4	22.5	1	100.00	9.5	even	ok

*Figure S 118: HRMS (APPI) of **1b**.*

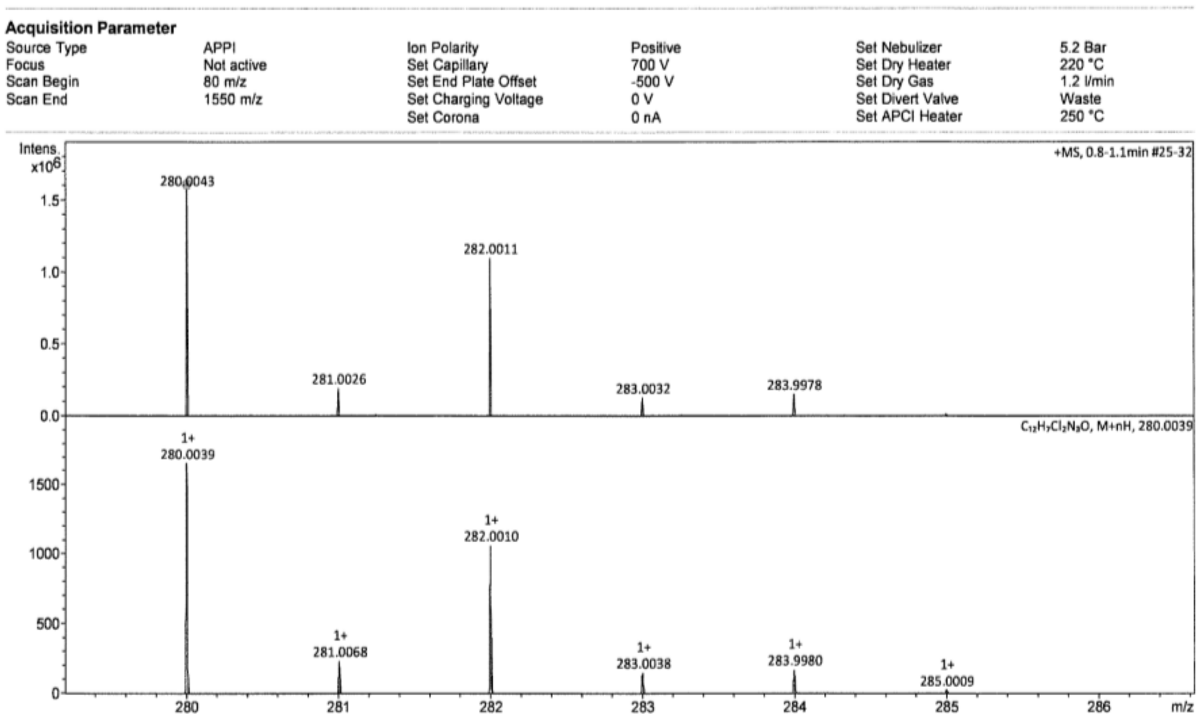

Figure S 119: Zoom of HRMS (APPI) of ***1b****.*

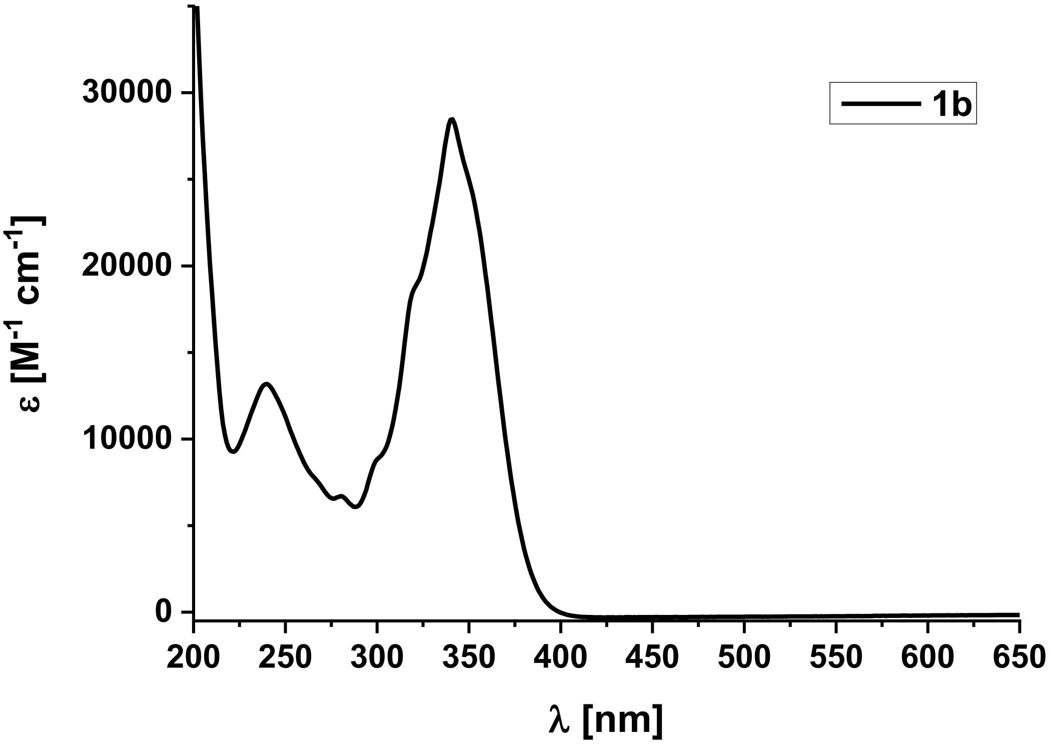

Figure S 120: UV/Vis extinction spectrum of ***1b*** *measured in MeCN.*

In addition, the characterization spectra for the 2-fold substituted analogue described in the synthesis section (*compare 2.6.1.*) are provided in the following:

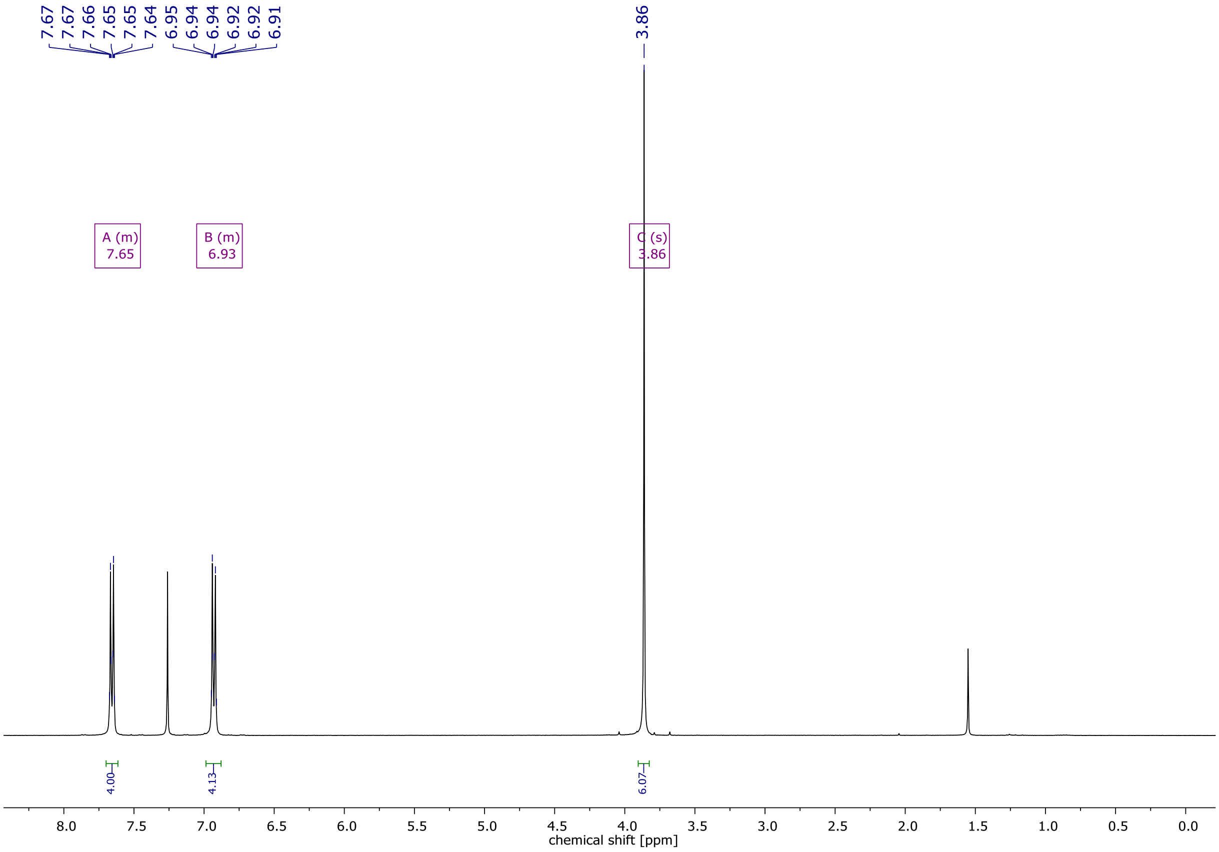

*Figure S 121: ^{1}H NMR spectrum of the 2-fold substituted analogue of **1b** measured in $CDCl_3$ (400 MHz).*

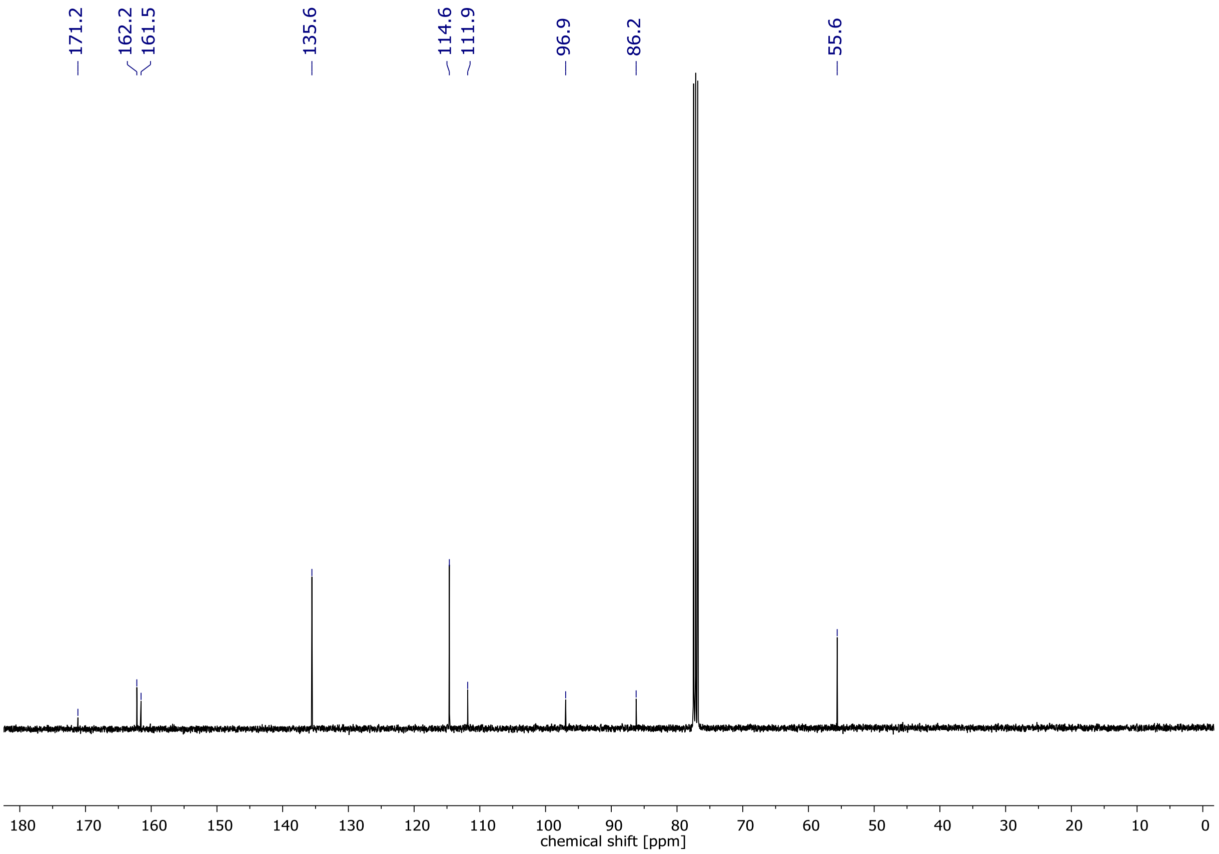

*Figure S 122: ^{13}C NMR spectrum of the 2-fold substituted analogue of **1b** measured in $CDCl_3$ (101 MHz)*

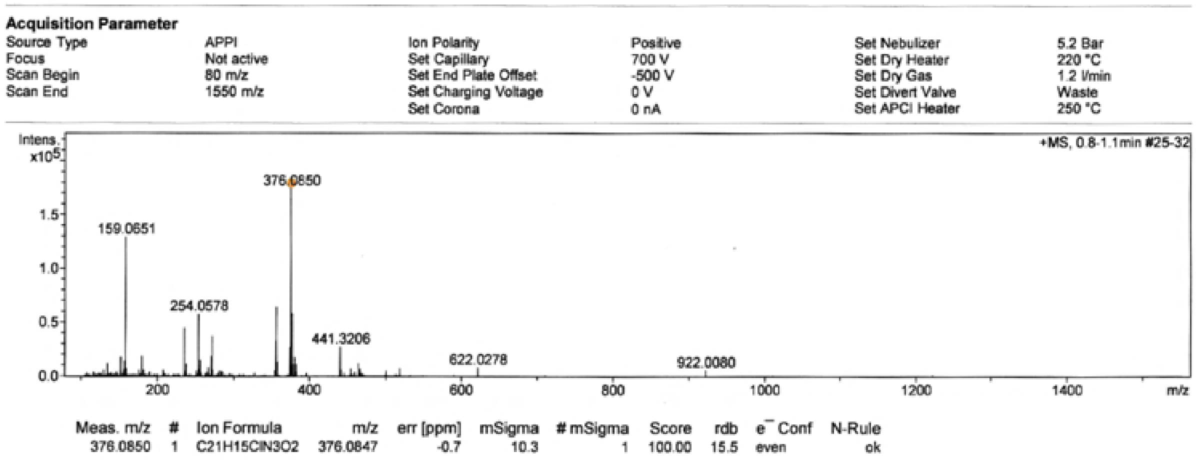

*Figure S 123: HRMS (APPI) of the 2-fold substituted analogue of **1b**.*

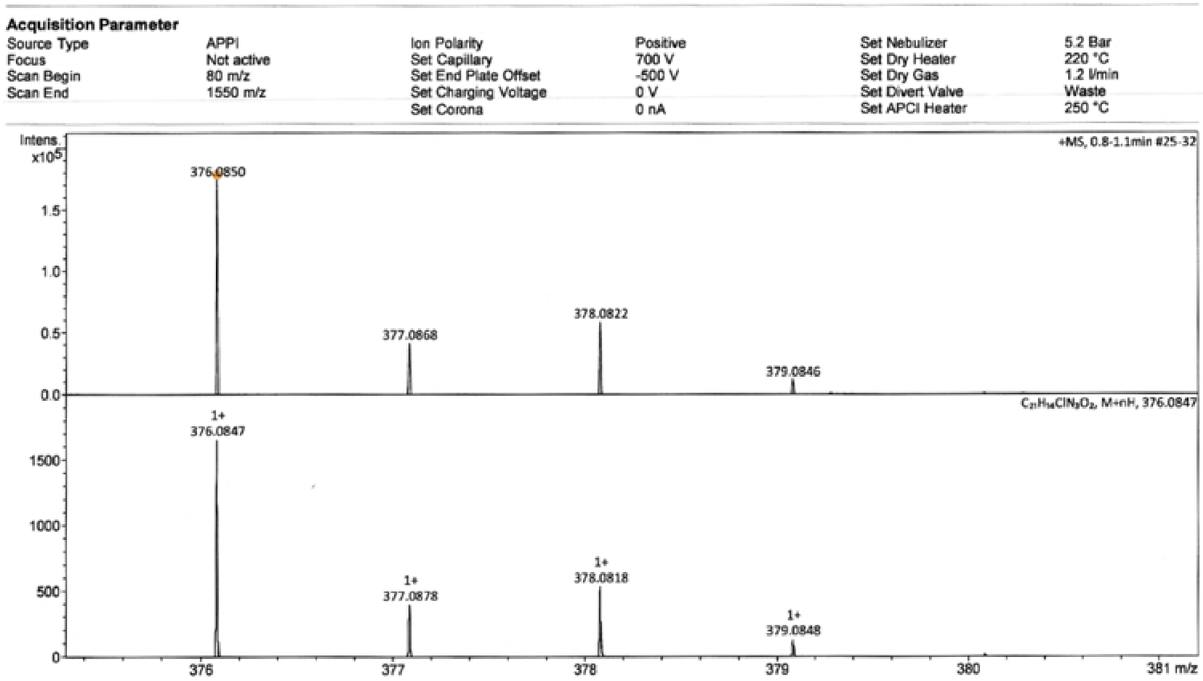

*Figure S 124: Zoom of HRMS (APPI) of the 2-fold substituted analogue of **1b**.*

3.6.2 **2c**

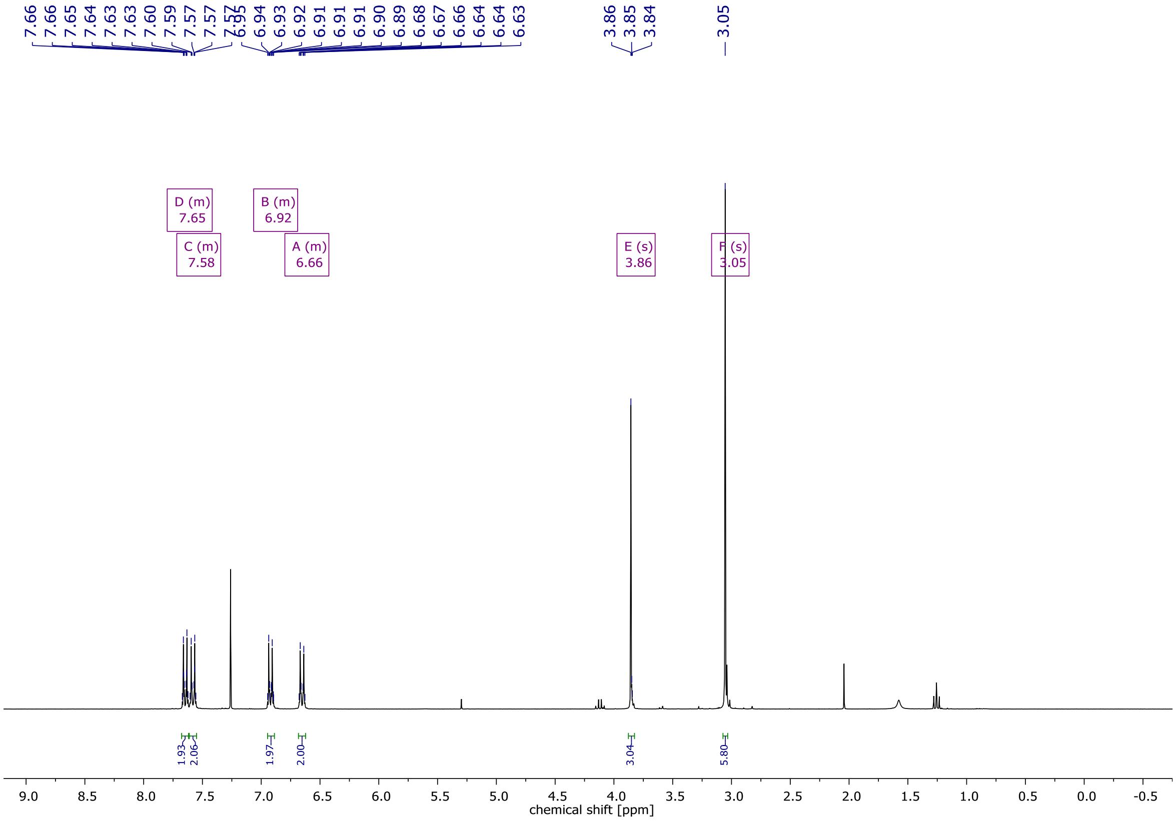

*Figure S 125: ^{1}H NMR spectrum of **2c** measured in $CDCl_3$ (400 MHz).*

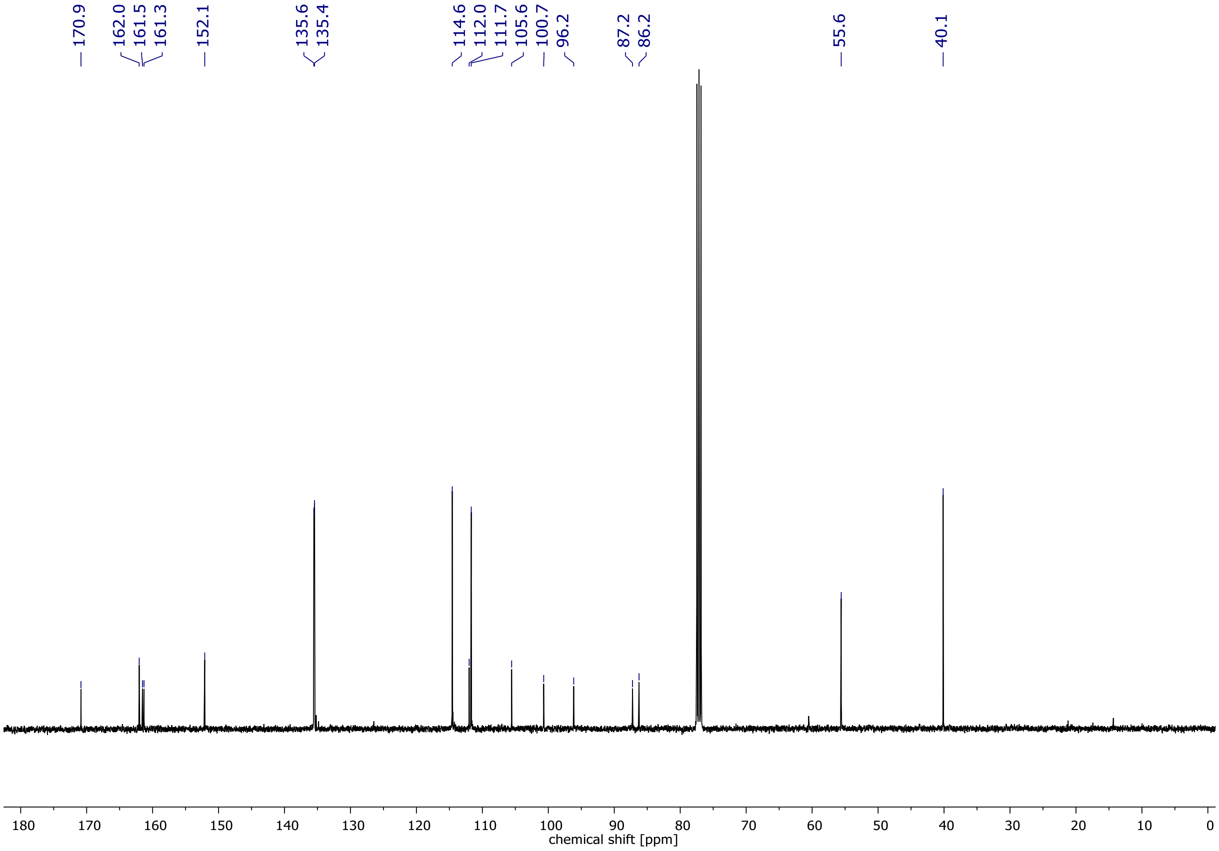

*Figure S 126: ^{13}C NMR Spectrum of **2c** measured in $CDCl_3$ (101 MHz).*

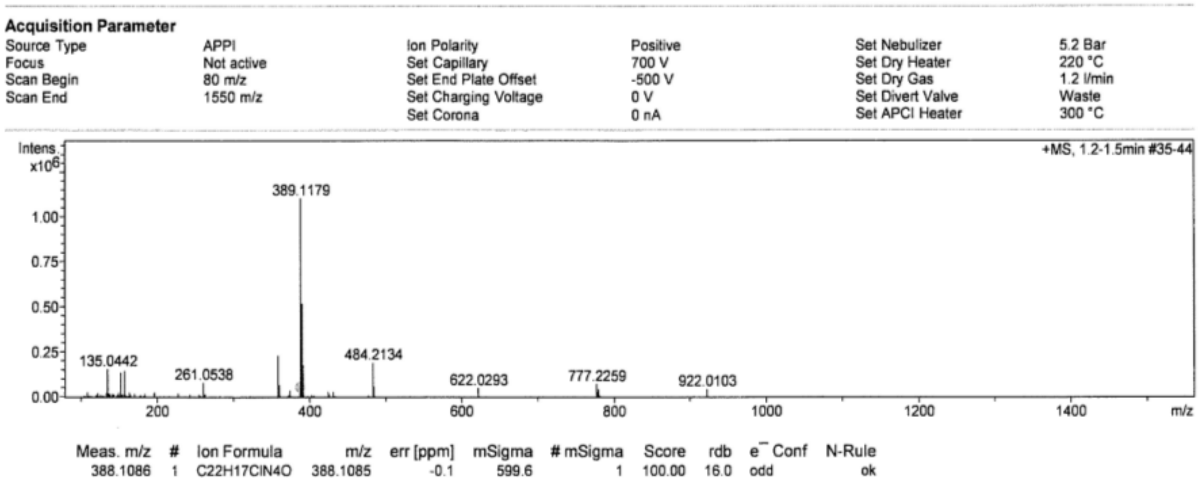

*Figure S 127: HRMS (APPI) of **2c***

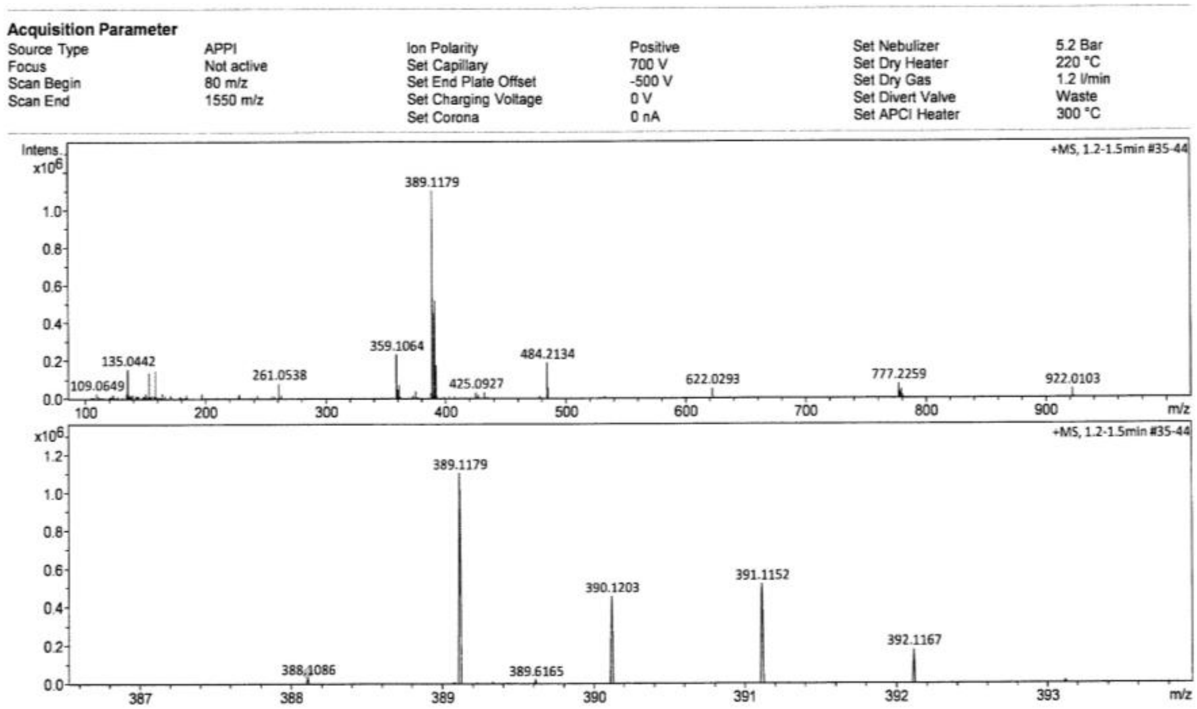

*Figure S 128: Zoom of HRMS (APPI) of **2c**.*

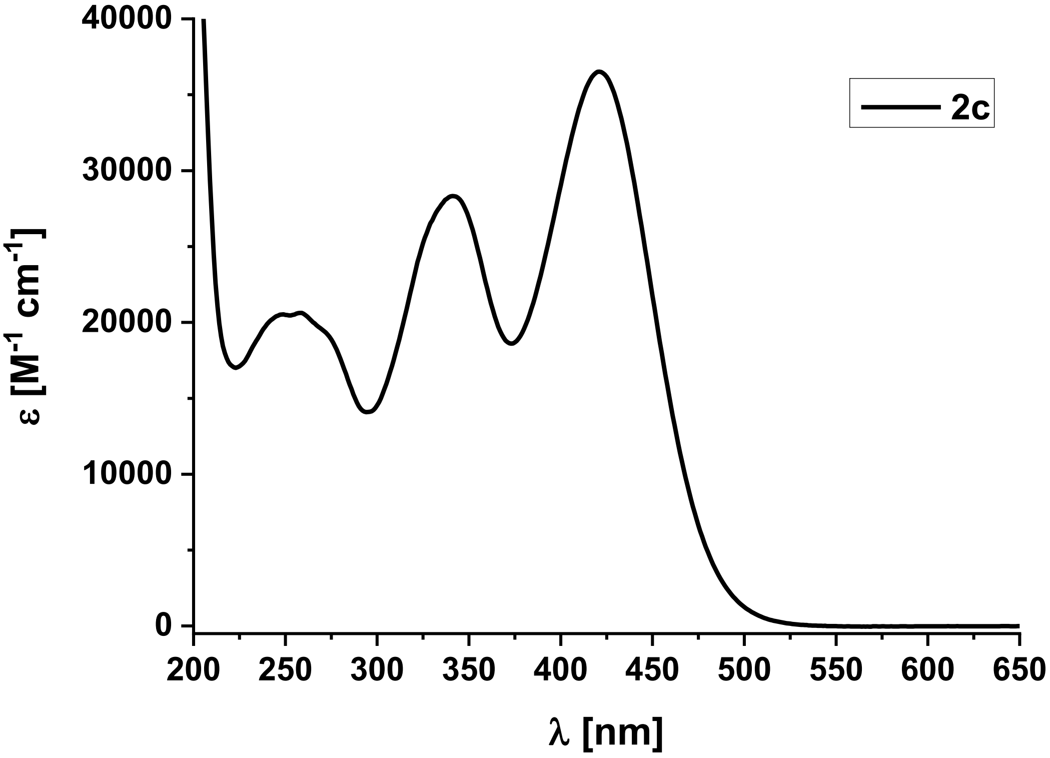

*Figure S 129: UV/Vis extinction spectrum of **2c** measured in MeCN.*

4 Switching studies monitored via NMR spectroscopy

All provided conversion ratios are given by correlating respective NBD and QC signals as described in the main manuscript. Thereby, significant non-overlapping signals were chosen. Due to baseline uncertainties potentially occurring during photo-irradiation experiments, some values must be seen as approximations. Therefore, although visually only signals corresponding to QC species and no leftover NBD signals can be found, quantitative conversion was not reported.

4.1 **Phenyl-mono NBD hybdrid:** NBD **4** to QC **9**

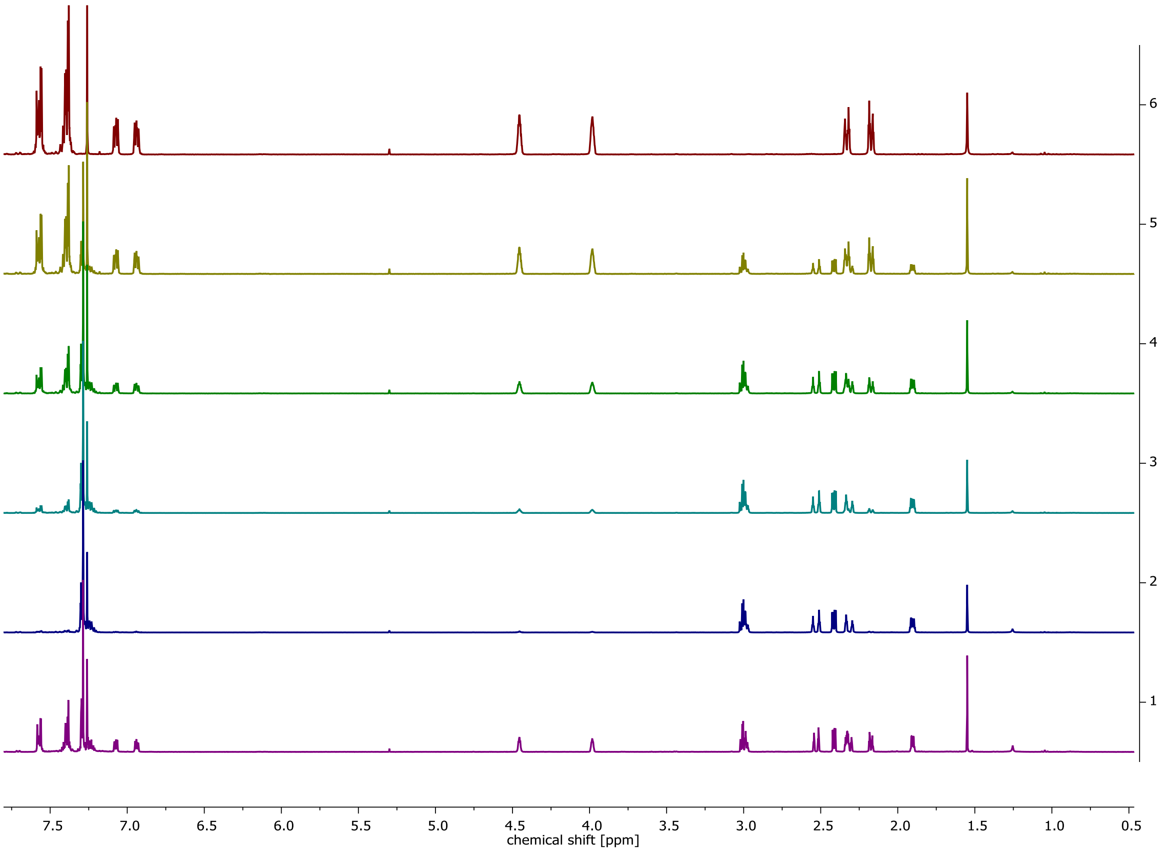

*Figure S 130: ^{1}H NMR switching study of 7.5 mg **4** to **9** measured in $CDCl_3$. For the conversion a 367 nm LED was used. The respective spectra can be assigned as follows: 6 = initial NBD spectrum, 5 = 2 minutes irradiation, 4 = after 5 minutes irradiation, 3 = after 10 minutes irradiation, 2 = after 20 minutes irradiation at, 1 = after 12 h at room temperature.*

*Table S 3: Corresponding results obtained for the ^{1}H NMR switching study of **4** to **9** measured in $CDCl_3$.*

**Irradiation time**	**QC [%]**
0	0
2 min	25
5 min	54
10 min	80
20 min	95
12h @ rt	50

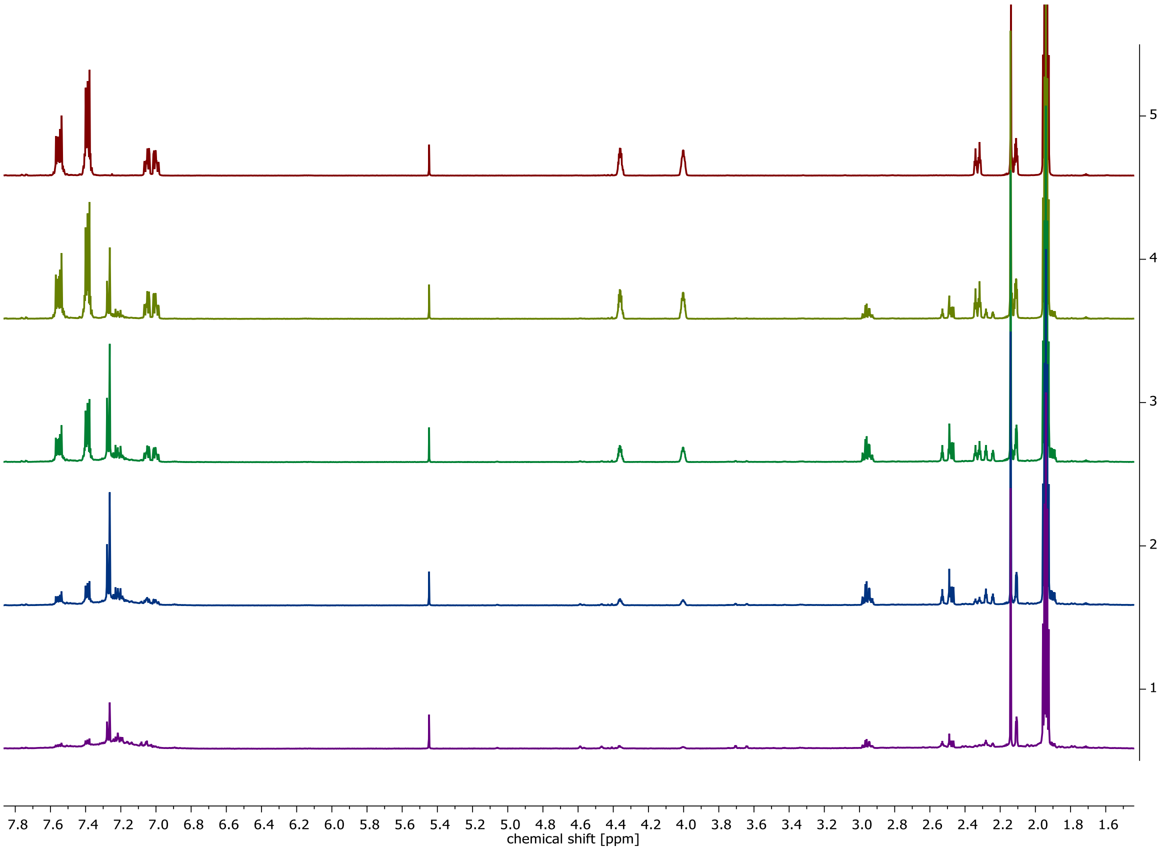

*Figure S 131: ^{1}H NMR switching study of 7.5 mg **4** to **9** measured in MeCN-d_3. For the conversion a 367 nm LED was used. The respective spectra can be assigned as follows: 5 = initial NBD spectrum, 4 = 2 minutes irradiation, 3 = after 5 minutes irradiation, 2 = after 10 minutes irradiation, 1 = after 20 minutes irradiation at 367 nm.*

*Table S 4: Corresponding results obtained for the ^{1}H NMR switching study of **4** to **9** measured inMeCN-d_3.*

Irradiation time	QC [%]
0	0
2 min	24
5 min	48
10 min	71 + Decomposition signals (approx. 5%)
20 min	Major decomposition observed

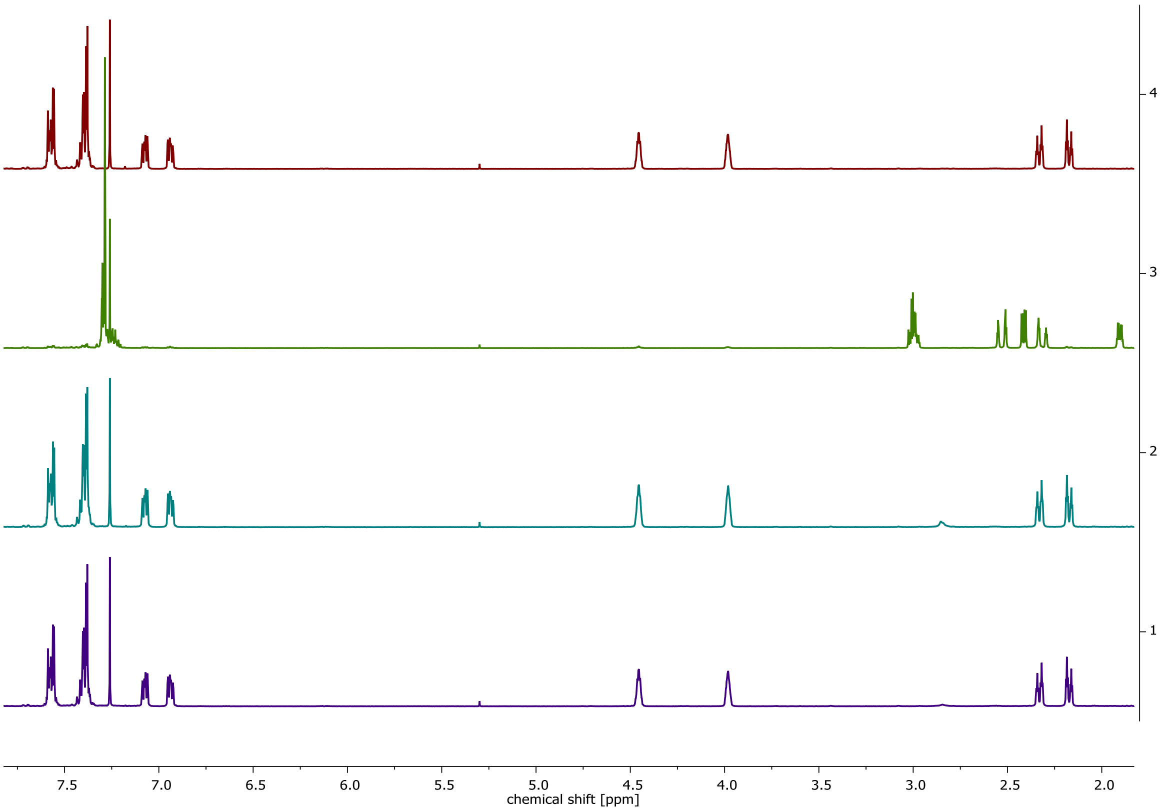

*Figure S 132: ^{1}H NMR switching study of 7.5 mg **4** to **9** measured in $CDCl_3$. For the conversion a 365 nm LED was used. The respective spectra can be assigned as follows: 4 = initial NBD spectrum, 3 = 5 minutes irradiation at 365 nm (50 % power, 15°C) ,2 = addition of a spatula tip **Por**, 1 = after 10 minutes after **Por** addition.*

*Table S 5: Corresponding results obtained for the ^{1}H NMR switching study of **4** to **9** measured in $CDCl_3$.*

Irradiation time	QC [%]
0	0
5 min	Quant.
+ **Por**	0
+Por and 10 min waiting	0

Additional waiting time and shaking of the sample after Por addition did not result in any further change of the recorded spectrum. No significant line broadening or other influences by the paramagnetic Co(II) porphyrin were observable.

4.2 Diphenyl-bis NBD hybrid: NBD **5** to QC **10**

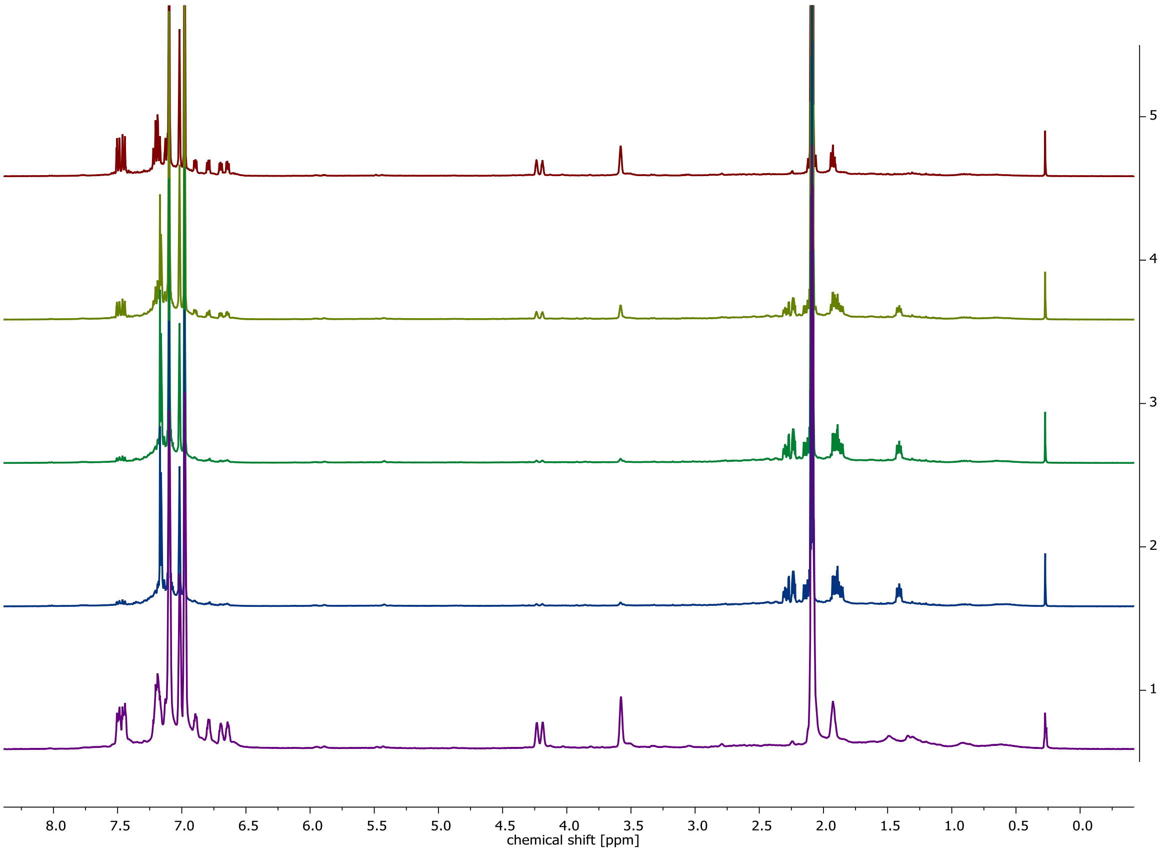

*Figure S 133: ^{1}H NMR switching study of 7.5 mg **5** to **10** measured in toluene-d_8. For the conversion a 365 (30%, 15°C) nm LED was used. The respective spectra can be assigned as follows: 5 = initial NBD spectrum, 4 = 2 minutes irradiation, 3 = after 5 minutes irradiation, 2 = after 5h at room temperature, 1 = addition of a spatula tip **Por**.*

*Table S 6: Corresponding results obtained for the ^{1}H NMR switching study of **5** to **10** measured in toluene-d_8.*

**Irradiation time**	**QC [%]**
0	0
2 min	42
5 min	85
5h @ rt	85
+ **Por**	0

After 5 minutes of irradiation, 85% conversion was determined according to integration, but visually no leftover NBD signals can be found (suggesting quantitative conversion). No spectral change proved thermal stability after 5h at rt. Upon **Por** addition, significant line broadening due to paramagnetic Co^{II} species was observed.

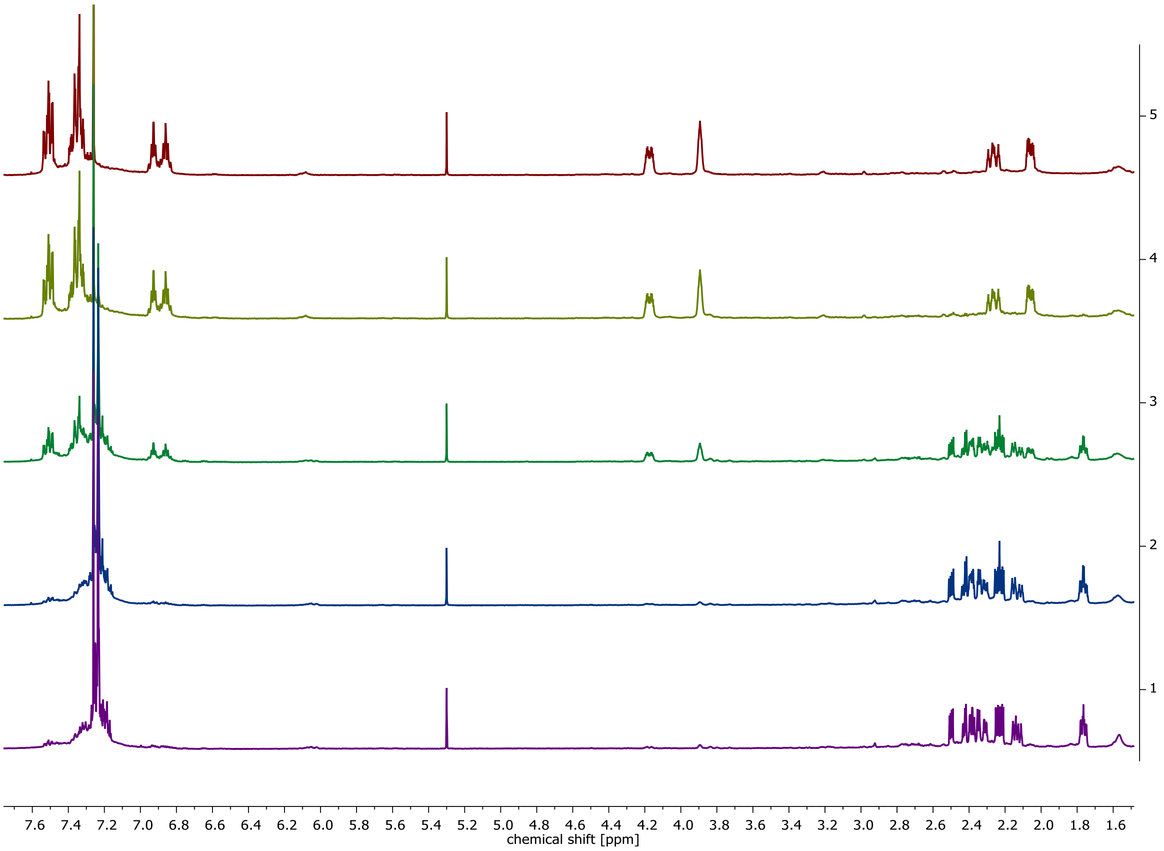

*Figure S 134: CDCl3 ^{1}H NMR switching study of 7.5 mg **5** to **10** measured in $CDCl_3$. For the conversion a 365 nm LED was used. The respective spectra can be assigned as follows: 5 = initial NBD spectrum, 4 = 1 minute irradiation (365 nm, 10%, 15°C), 3 = after 2 minutes irradiation (365 nm, 30%, 15°C), 2 = after 5 minutes irradiation (365nm, 30%, 15°C) at room temperature, 1 = after 8h thermal decay at room temperature.*

*Table S 7: Corresponding results obtained for the ^{1}H NMR switching study of **5** to **10** measured in $CDCl_3$.*

Irradiation time	QC [%]
0	0
1 min	11
2 min	48
5 min	85
8h @ rt	85

During the experiment, the LED power was stepwise increased, as indicated in the figure description above. After 5 minutes of irradiation, 85% conversion was determined according to integration, but visually, no leftover NBD signals can be found (suggesting quantitative conversion). Thermal stability was proven by no spectral change after 8h at rt.

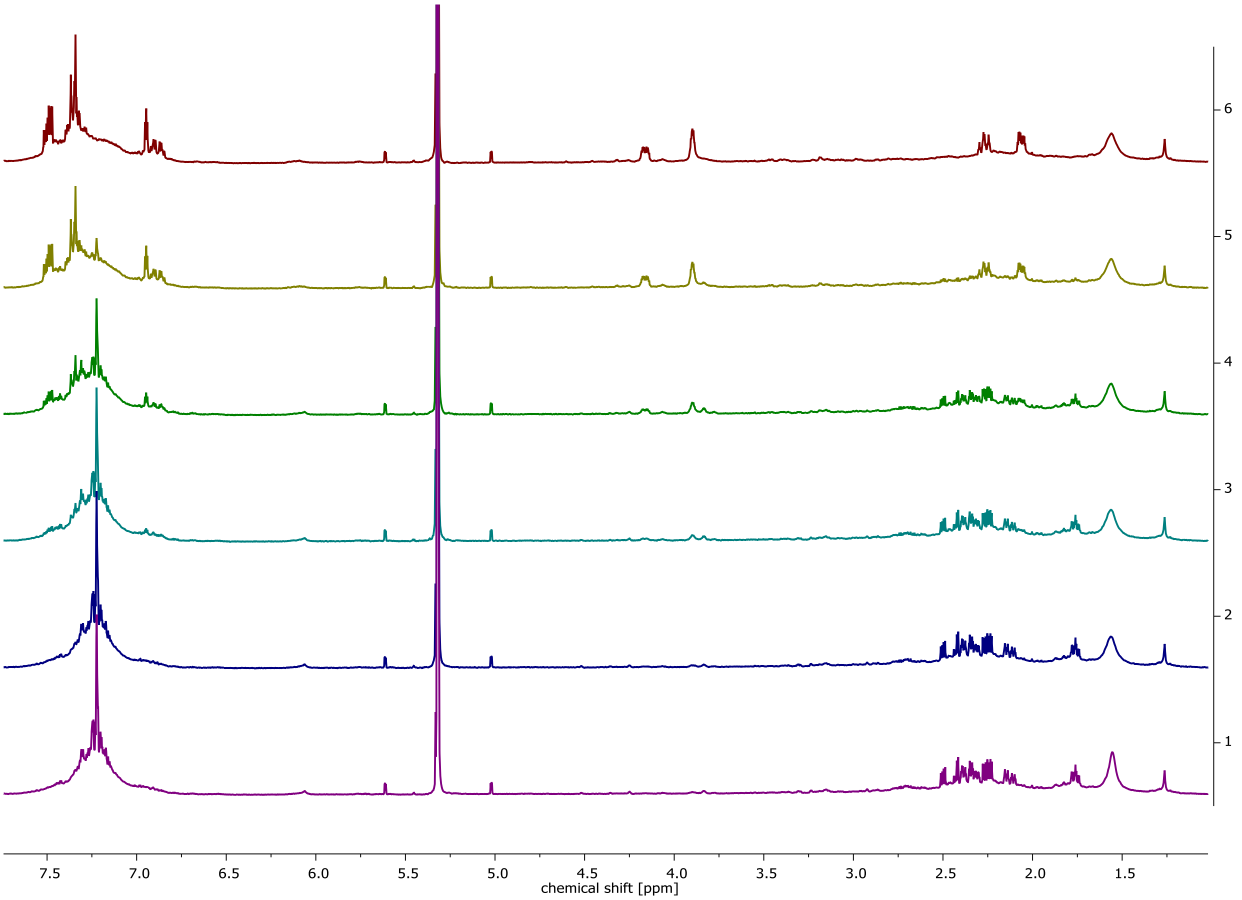

*Figure S 135: ^{1}H NMR switching study of 7.8 mg **5** to **10** measured in CD_2Cl_2. For the conversion a 367 nm LED was used. The respective spectra can be assigned as follows: 6 = initial NBD spectrum, 5 = 1 minute irradiation, 4 = after 3 minutes irradiation, 3 = after 5 minutes irradiation, 2 = after 7 minutes irradiation, 1 = after 7 h thermal decay at room temperature*

*Table S 8: Corresponding results obtained for the ^{1}H NMR switching study of **5** to **10** measured in CD_2Cl_2. Overall, integration is not ideal due to a rough baseline during the switch.*

Irradiation time	QC [%]
0	0
1 min	37
3 min	59
5 min	72
7 min	83
7h @ rt	83

After 7 minutes of irradiation, 83% conversion was determined according to integration, but visually, no leftover NBD signals can be found (suggesting quantitative conversion). Thermal stability was proven by no spectral change after 7h at rt.

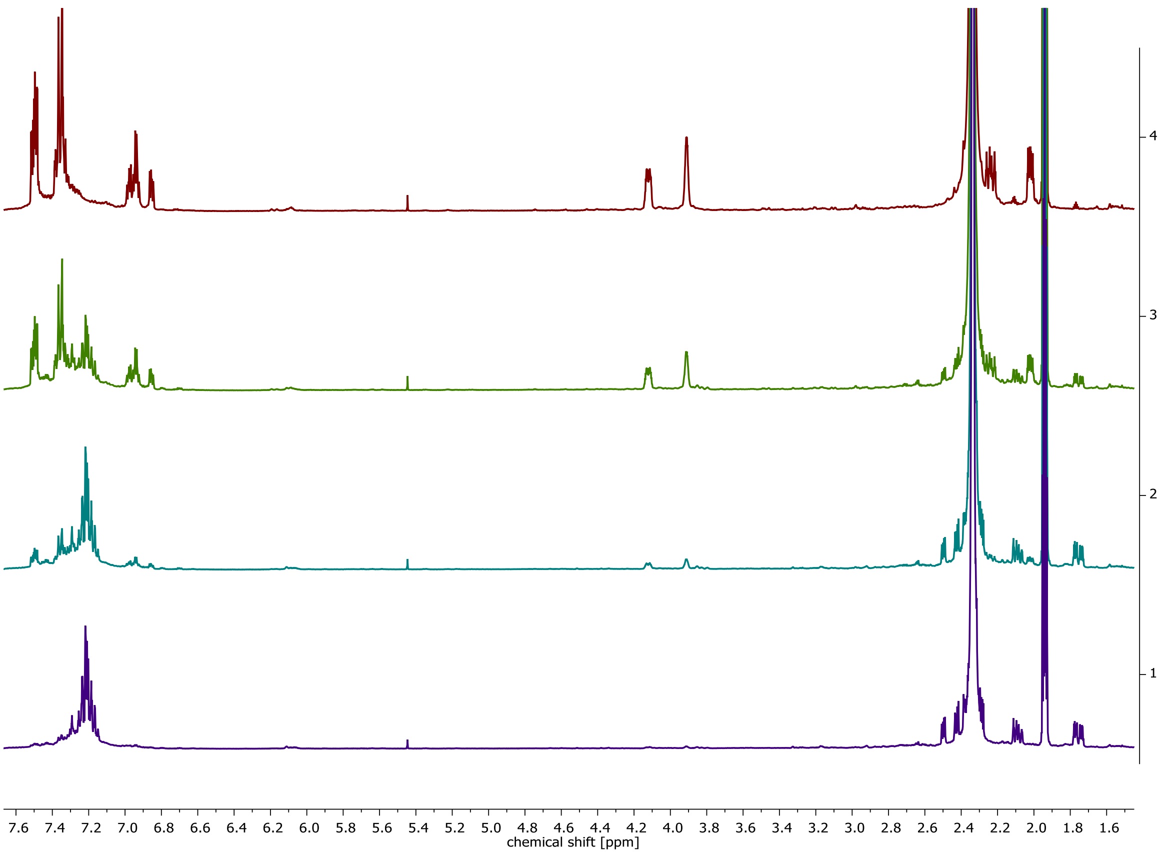

*Figure S 136: ^{1}H NMR switching study of 8.7 mg **5** to **10** measured in MeCN-d_3. Filtered NMR sample! For the conversion a 365 nm LED was used. The respective spectra can be assigned as follows: 4 = initial NBD spectrum, 3 = 1 minute irradiation at 365 nm (30% power, 15°C), 2 = 3 minutes irradiation at 365 nm (30% power, 15°C), 1 = 5 minutes irradiation at 365 nm (30% power, 15°C).*

*Table S 9: Corresponding results obtained for the ^{1}H NMR switching study of **5** to **10** measured in MeCN-d_3.*

Irradiation time	QC [%]
0	0
1 min	40
3 min	79
5 min	92

4.3 *N,N*-dimethylaniline-mono NBD hybrid: NBD **6** to QC **11**

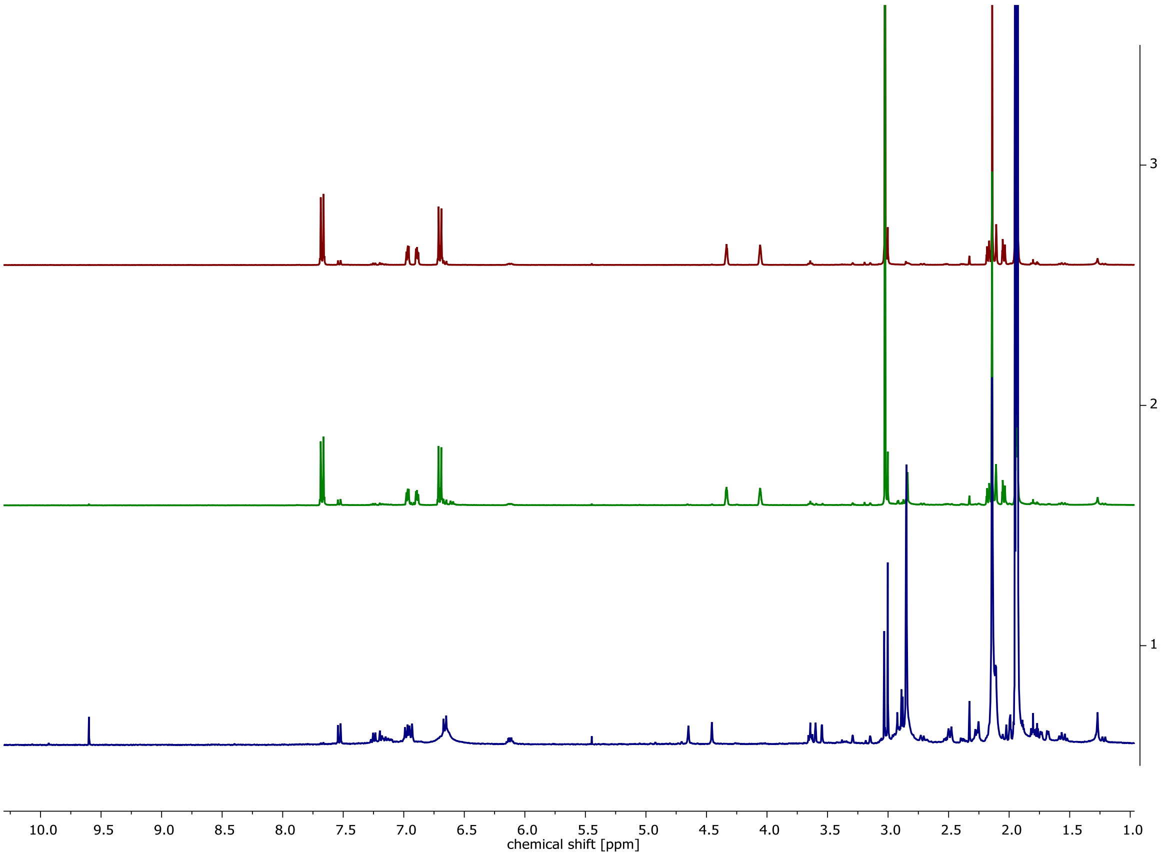

*Figure S 137: ^{1}H NMR switching study of 4.1 mg **6** to **11** measured in MeCN-d_3. For the conversion a 475 nm LED was used. The respective spectra can be assigned as follows: 3 = initial NBD spectrum, 2 = 30 minutes irradiation, 1 = after 4 h irradiation.*

*Table S 10: Corresponding results obtained for the ^{1}H NMR switching study of **6** to **11** measured in MeCN-d_3.*

Irradiation time	QC [%]
0	0
30 min	/
4 h	/

In this experiment, no clear QC formation can be observed while the NBD signals decrease over time. Photodecomposition is more likely to occur since the resulting new signals cannot be correlated to some sort of QC species. However, since the shape of the new signals is quite good, the appearance of another not further determined species can be assumed.

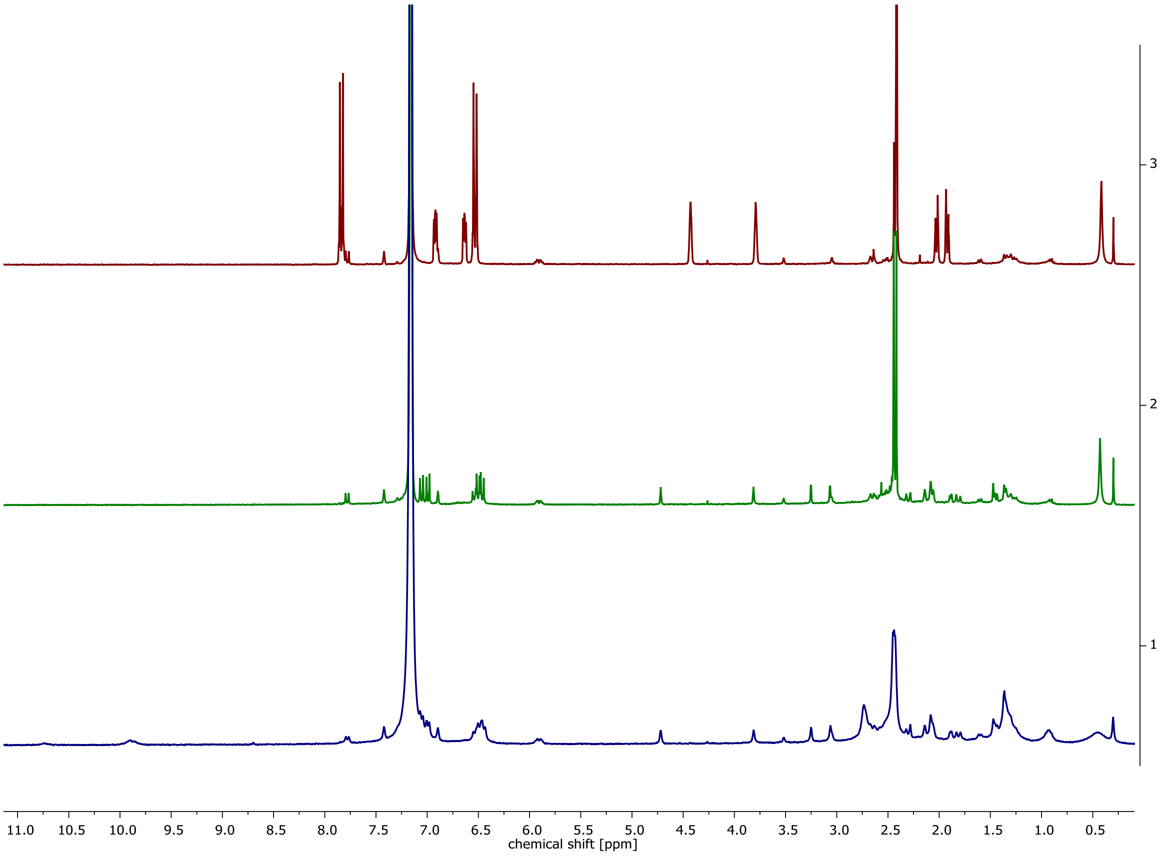

*Figure S 138: ^{1}H NMR switching study of 4.7 mg **6** to **11** measured in benzene-d$_6$. For the conversion a 475 nm LED was used. The respective spectra can be assigned as follows: 3 = initial NBD spectrum, 2 = 2 h irradiation, 1 = addition of 0.4 mg **Por**.*

*Table S 11: Corresponding results obtained for the ^{1}H NMR switching study of **6** to **11** measured in benzene-d$_6$.*

**Irradiation time**	**QC [%]**
0	0
2 h	/
+ **Por**	/

After 2 h of irradiation, the NBD signals have completely vanished, while no corresponding QC signals could be found. Further, upon the addition of small amounts of **Por**, recovery of NBD **6** was not possible. Thus, photodecomposition or generation of a species that is not QC must have occurred.

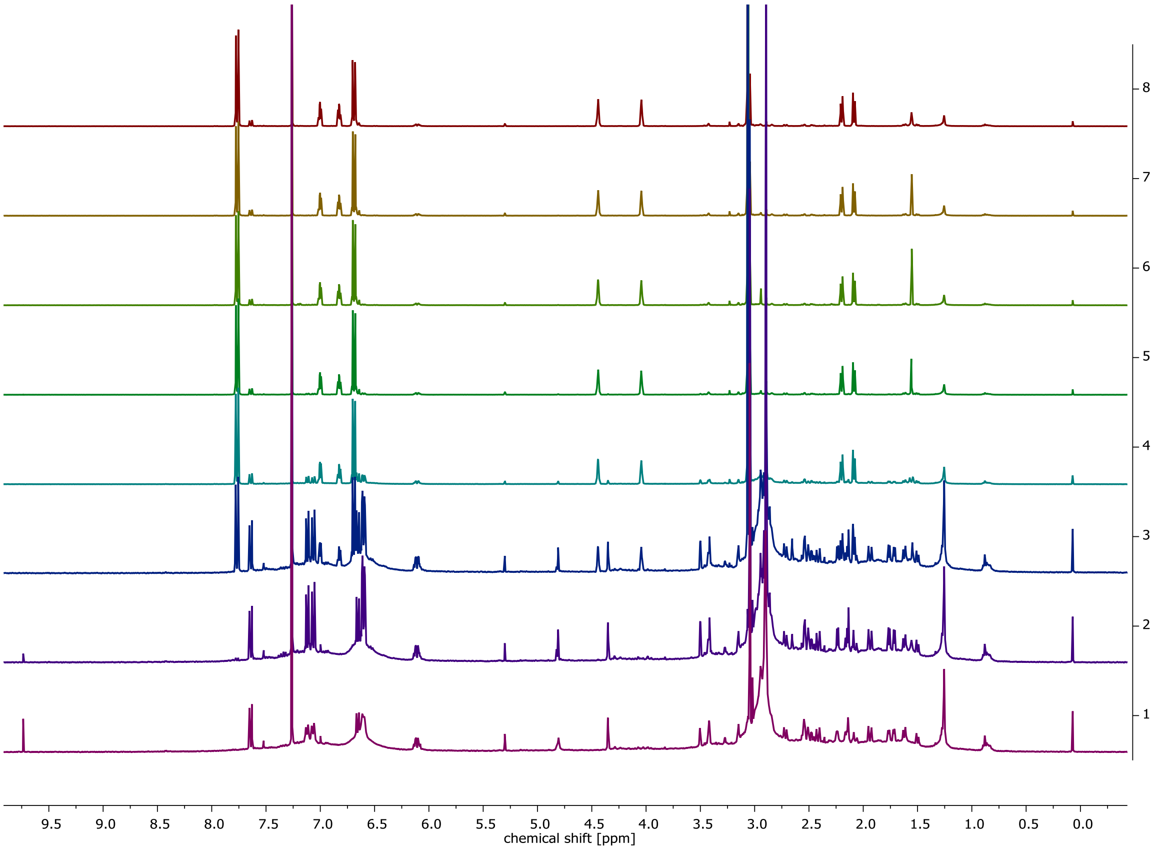

*Figure S 139: ^{1}H NMR switching study of 7.5 mg **6** to **11** measured in $CDCl_3$. For the conversion different LEDs were consecutively used. After each switch of the LED the very same sample as before was irradiated but the irradiation times stated at zero. The respective spectra can be assigned as follows: 8 = initial NBD spectrum, 7 = 30 s irradiation at 475 nm, 6: 2 min irradiation at 475 nm, 5 = 2 min irradiation at 425 nm (30%, 15 °C), 4 = 10 min irradiation at 425 nm (60%, 15 °C), 3 = 20 min irradiation at 425 nm (80%, 15 °C),2 = 40 min irradiation at 425 nm (80%, 15 °C), 1 = 80 min irradiation at 425 nm (80%, 15 °C).*

In this experiment, the complete vanishing of the NBD signals over time was found increasing the relative intensity of the leftover side species. The multiplet at 6.14-6.08 ppm can be assigned to the formed DIELS-ALDER adduct of norbornadiene with an additional cyclopentadiene formed during the reaction process, as described in the main manuscript.[9] Therefore, it has to be assumed that, combined with the precipitation, the signals left in the spectrum after 20 minutes at 80% irradiation power are just intensified signals of the already included adduct (Especially visible by comparison with the intensity of the signals corresponding to impurities, such as grease, at 0.88 ppm). Signals corresponding to a newly formed QC species could not be found. In combination with the observed precipitation occurring during the experiment, the dimerization or polymerization of **6** could be assumed, resulting in the insoluble precipitate. Thus, two molecules of **6** would be connected *via* a 2+2 cycloaddition of the unsubstituted double bonds. During the theoretical calculations process for all molecules, the formation of such species was indicated to be possible. Considering high-resolution mass spectroscopic analysis, signals approx. fitting for the mentioned species, combined with one Na+ atom, can be found (*section 3.3.3*). Prolonged irradiation led to further formation of undefined side products and precipitation. Further analysis of the formed precipitate was impossible due to solubility issues after the isolation process. Therefore, neither a minimal amount necessary for mass spectroscopic analysis could be solubilized, and thus no measurements could be conducted.

4.4 ***N,N*-dimethylaniline-phenyl-bis-NBD hybrid:** NBD **7** to QC **12** (and intermediate species)

4.4.1 NBD to QC interconversion experiments monitored via NMR

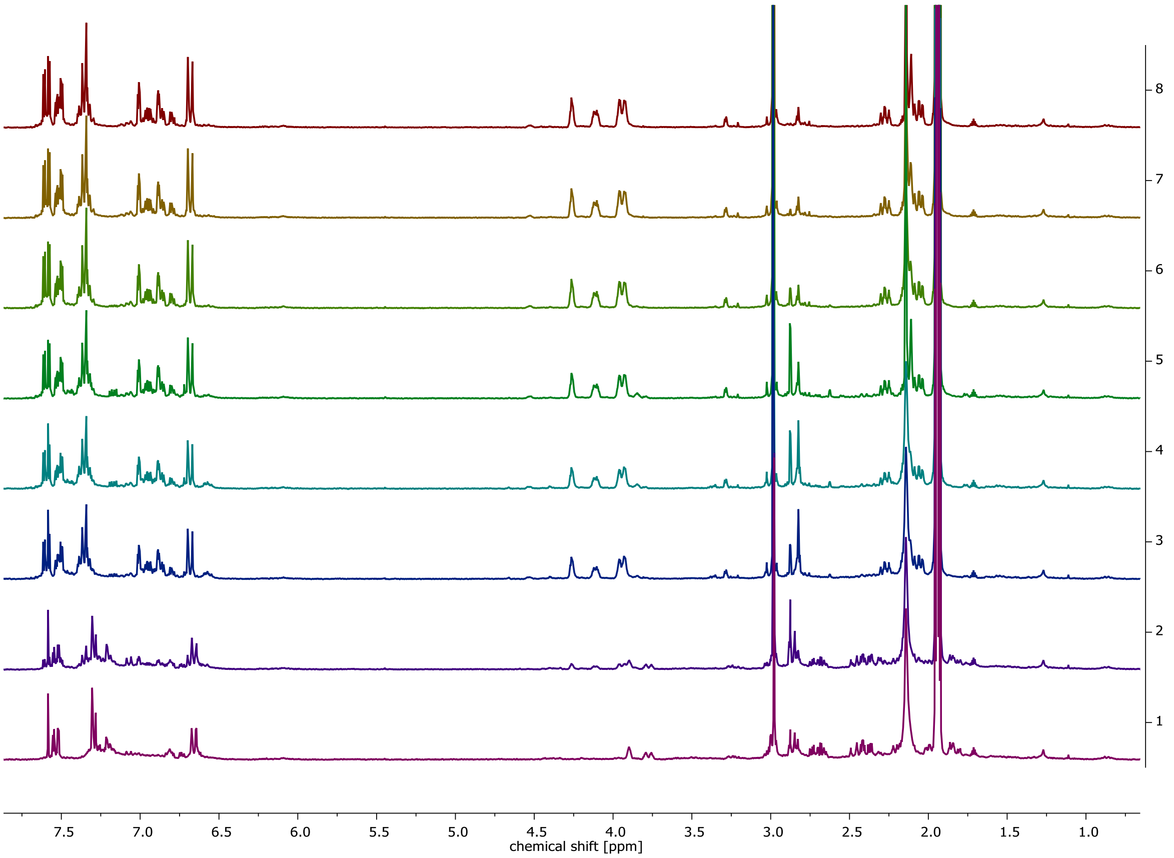

*Figure S 140: ^{1}H NMR switching study of 7.5 mg **7** to **12b** measured in MeCN-d_3. For the conversion different LEDs were consecutively used. After each switch of the LED the very same sample as before was irradiated but the irradiation times stated at zero. The respective spectra can be assigned as follows: 8 = initial NBD spectrum, 7 = 2min irradiation at 475 nm, 6: 10 min irradiation at 475 nm, 5 = 70 min irradiation at 475 nm, 4 = 4 h irradiation at 475 nm, 3 = 5 min irradiation at 310 nm (50%, 15 °C), 2 = 10 min irradiation at 425 nm (75%, 15 °C), 1 = 20 min irradiation at 425 nm (75%, 15 °C).*

*Table S 12: Corresponding results obtained for the ^{1}H NMR switching study of **7** to **12b** measured in MeCN-d_3.*

**Irradiation time**	**QC [%]** (relative to **7**)
0	0
2 min (475 nm)	0
10 min (475 nm)	9
70 min (475 nm)	24
4 h (475 nm)	26
5 min (310 nm)	21 (no additional signals corresponding to **12a** were found)
10 min (425 nm)	64

20 min (425 nm)	Quantitative (potentially leftover signals of **7** are overshadowed by the baseline)

During the experiment precipitation occurred especially after prolonged irradiation using the higher-power 310 nm and 425 nm LED blocks. Therefore, integration was disturbed by a worse signal to noise ratio and the relative ratio of **12b** decreased at some point. The subsequent switch back to 310 nm to convert **12b** into **12** led to photodecomposition.

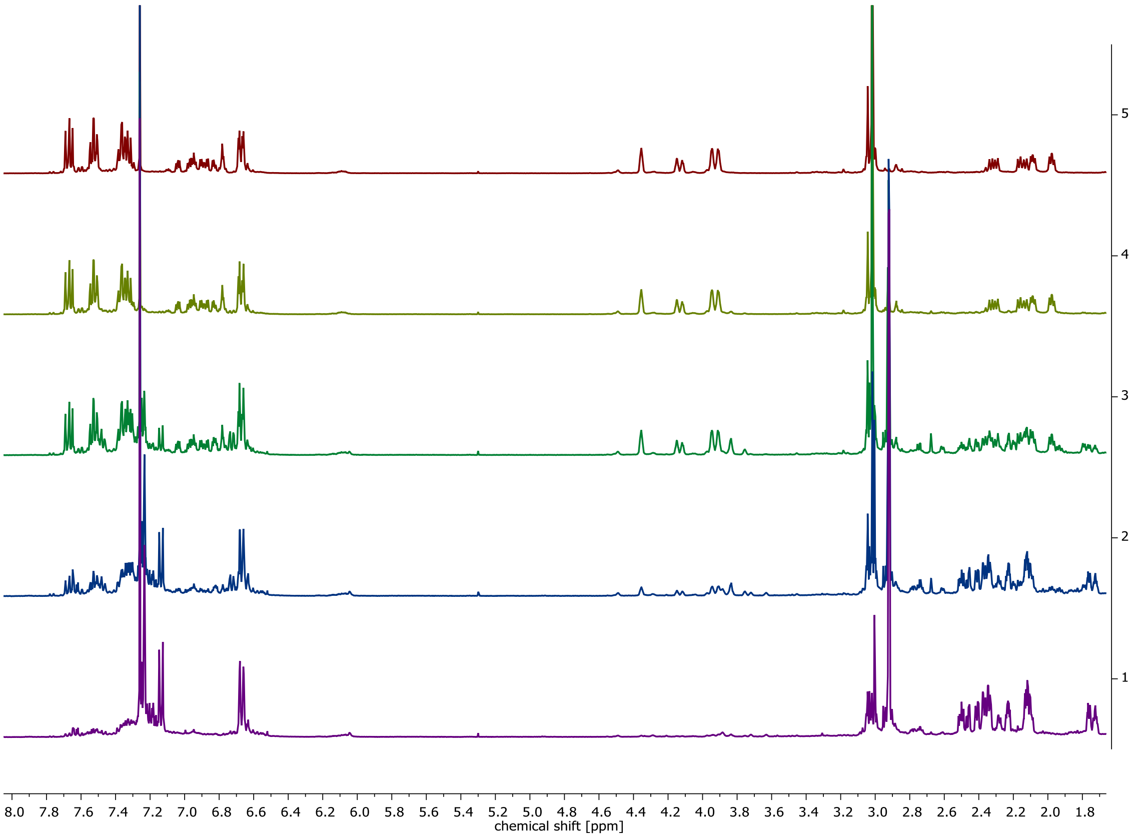

*Figure S 141: ^{1}H NMR switching study of **7** to **12** measured in $CDCl_3$. For the conversion a 340 nm LED was used. The respective spectra can be assigned as follows: 5 = initial NBD spectrum, 4 = 2 min irradiation, 3 = 10 min irradiation, 2 = 30 min irradiation, 1 = 70 min irradiation.*

*Table S 13: Corresponding results obtained for the ^{1}H NMR switching study of **7** to **12** measured in $CDCl_3$.*

Irradiation time	QC [%]
0	0
2 min	8
10 min	31
30 min	64
70 min	87

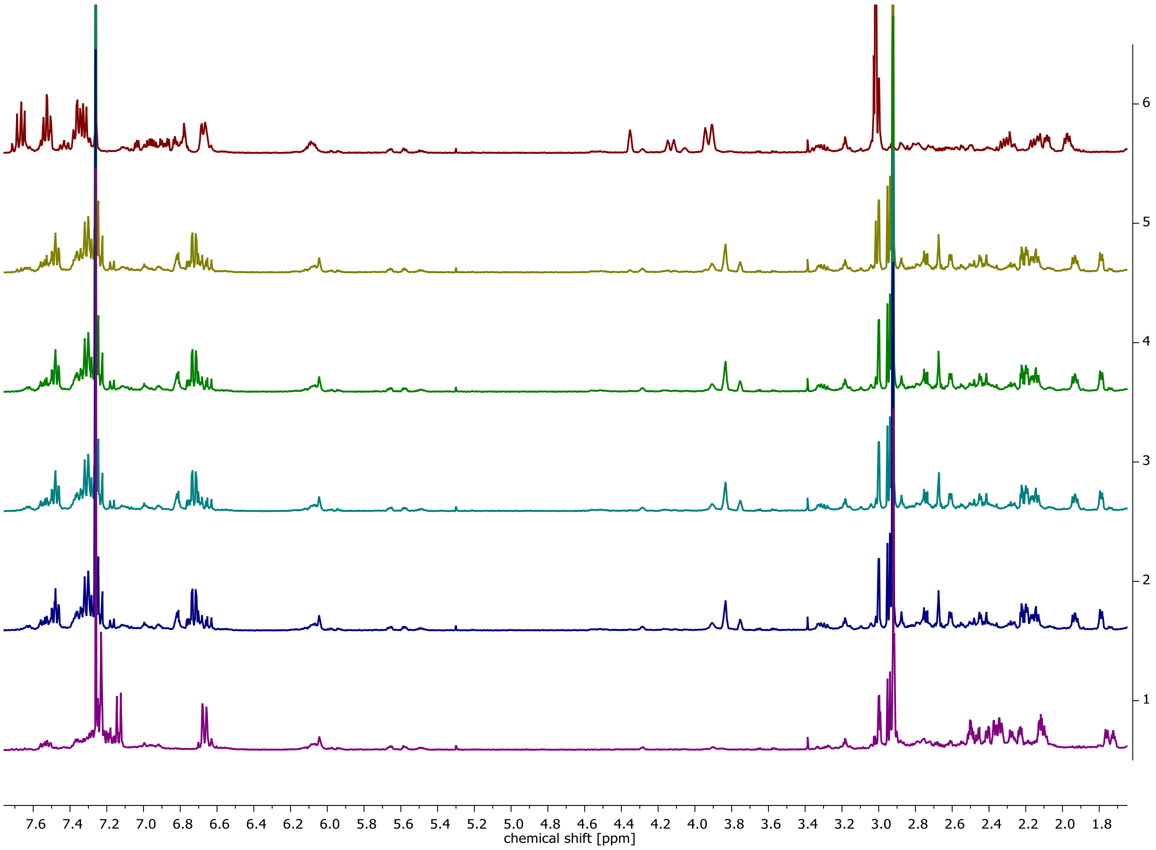

*Figure S 142: ^{1}H NMR switching study of **7** to **12b** and subsequently to **12** measured in $CDCl_3$. Signals in the range of 6.15-5.46 ppm correspond to impurities in this sample which could not be removed during purification. For the conversion different LEDs were consecutively used. After each switch of the LED the very same sample as before was irradiated but the irradiation times stated at zero. The respective spectra can be assigned as follows: 6 = initial NBD spectrum, 5 = 30 min irradiation at 475 nm, 4 = 60 min irradiation at 475 nm, 3 = 120 min irradiation at 475 nm, 2 = 165 min irradiation at 475 nm, 1 = 35 min irradiation at 340 nm.*

*Table S 14: Corresponding results obtained for the ^{1}H NMR switching study of **7** over **12b** to **12** measured in $CDCl_3$.*

**Irradiation time**	**QC [%]** (formed single side species **12b** relative to **7**)
0	0
30 min (475 nm)	81
60 min (475 nm)	91
120 min (475 nm)	Quant. to **12b**
165 min (475 nm)	Quant. to **12b**
35 min (340 nm)	Quant. conversion to QC **12**

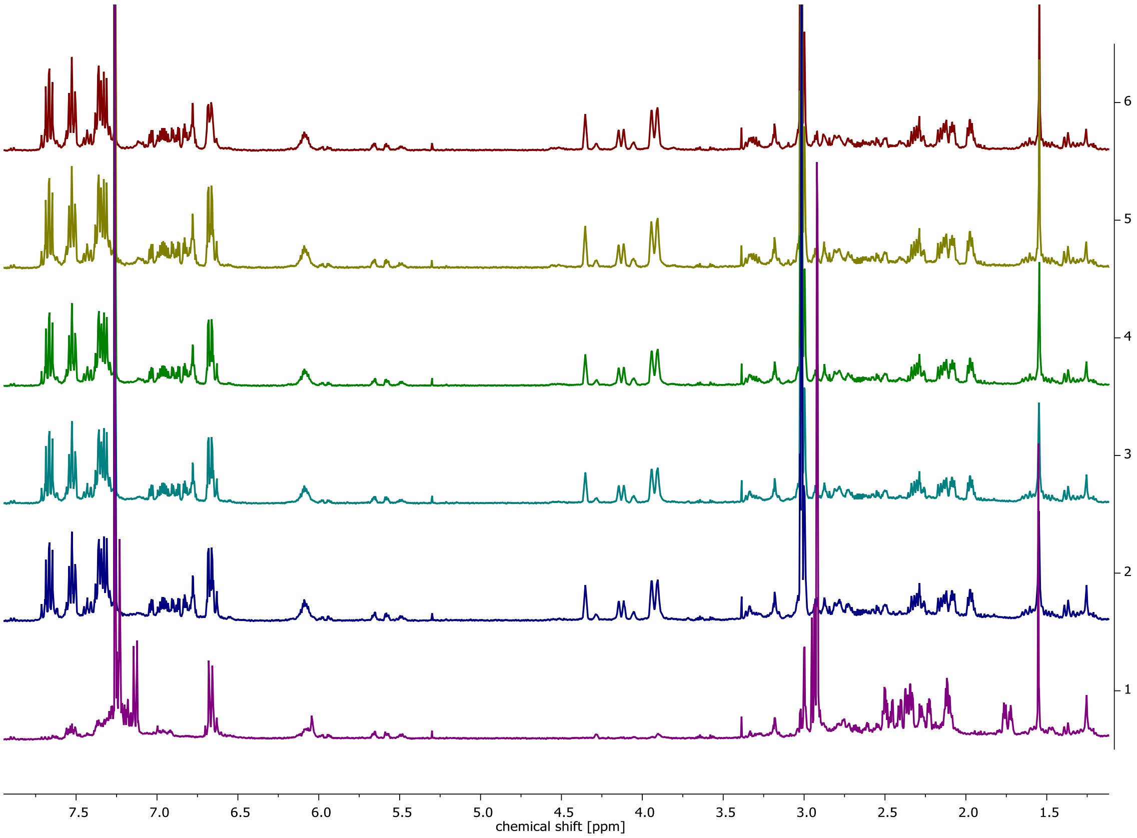

*Figure S 143: ^{1}H NMR switching study of **7** to **12a** and subsequently to **12** measured in $CDCl_3$. Signals in the range of 6.15-5.46 ppm correspond to impurities in this sample which could not be removed during purification. For the conversion different LEDs were consecutively used. After each switch of the LED the very same sample as before was irradiated but the irradiation times stated at zero. The respective spectra can be assigned as follows: 6 = initial NBD spectrum, 5 = 30 min irradiation at 310 nm, 4 = 60 min irradiation at 310 nm, 3 = 120 min irradiation at 310 nm, 2 = 165 min irradiation at 310 nm, 1 = 35 min irradiation at 340 nm.*

*Table S 15: Corresponding results obtained for the ^{1}H NMR switching study of **7** over **12a** to **12** measured in $CDCl_3$.*

Irradiation time	QC [%]
0	0
30 min (310 nm)	/
60 min (310 nm)	/
120 min (310 nm)	/
165 min (310 nm)	/
35 min (340 nm)	Quant. conversion to QC **12**

At this scale no conversion in the single phenyl side switched QC **12a** could be obtained. Subsequent irradiation at 340 nm however yielded complete conversion to **12**.

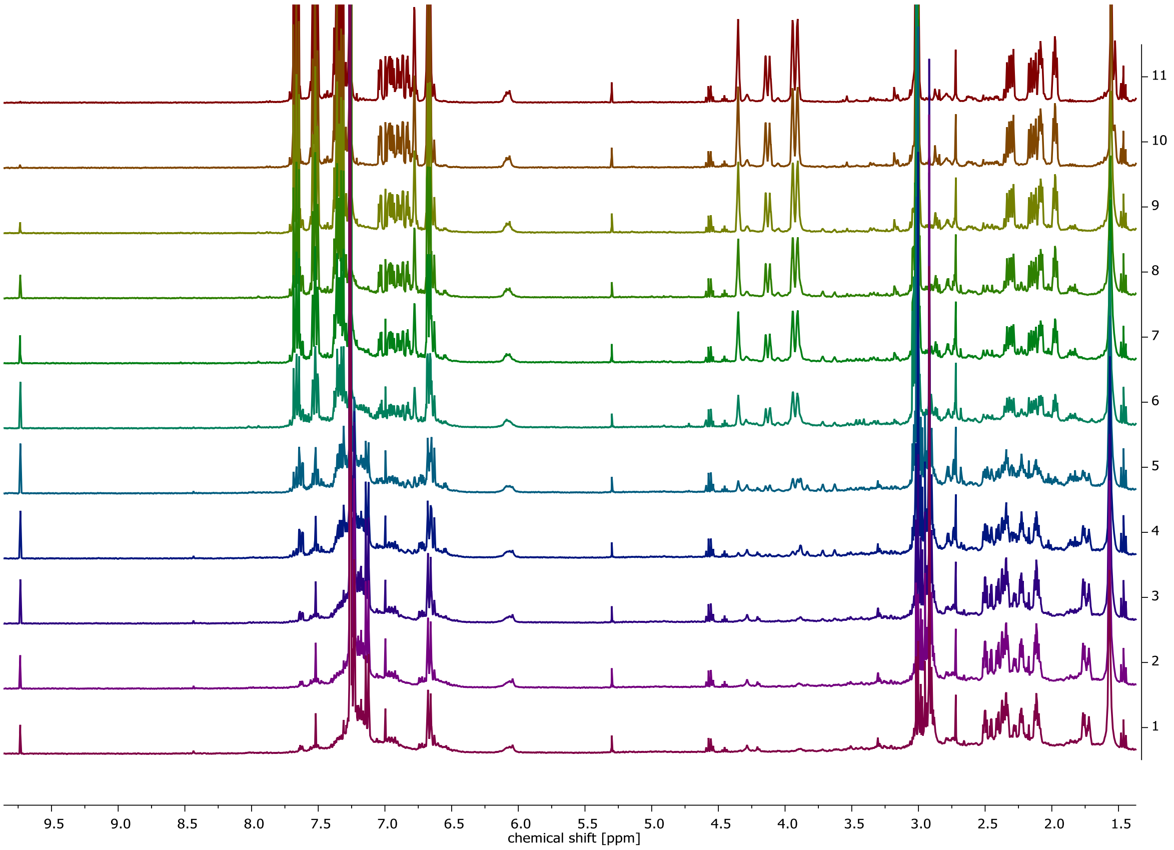

*Figure S 144: 310 ^{1}H NMR switching study of 7.5 mg **7** to **12** measured in MeCN-d_3. The multiplet, quartet and triplet signals at 6.15-6.05 ppm, 4.57 ppm and 1.46 ppm, respectively, correspond to impurities in this sample which could not be removed during purification. For the conversion different LEDs were consecutively used. After each switch of the LED or adjustment of the used power the very same sample as before was irradiated but the irradiation times stated at zero. The respective spectra can be assigned as follows: 11 = initial NBD spectrum, 10 = 5 min irradiation at 310 nm (30%, 15 °C), 9 = 15 min irradiation at 310 nm (50%, 15 °C), 8 = 30 min irradiation at 310 nm (80%, 15 °C), 7 = 60 min irradiation at 310 nm (80%, 15 °C), 6: 150 min irradiation at 310 nm (80%, 15 °C), 5 = 5 min irradiation at 425 nm (30%, 15 °C), 4 = 10 min irradiation at 425 nm (30%, 15 °C), 3 =20 min irradiation at 425 nm (30%, 15 °C), 2 = 10 min irradiation at 475 nm, 1 = 30 min irradiation at 340 nm.*

*Table S 16: Corresponding results obtained for the ^{1}H NMR switching study of **7** to **12** measured in $CDCl_3$.*

**Irradiation time**	**QC [%]** (of **12** relative to **7**, the intermediate side species was neglected for the calculation)
0	0
5 min (310 nm)	/
15 min (310 nm)	/
30 min (310 nm)	/
60 min (310 nm)	/
150 min (310 nm)	/
5 min (425 nm)	66

10 min (425 nm)	81
20 min (425 nm)	88 (visually quantitative)
10 min (475 nm)	No further change
30 min (340 nm)	No further change

Initially, using the 310 nm LED, no conversion to QC species besides some small photodecomposition was found, similar to what was described before. By changing the wavelength to 425 nm, conversion into QC species was initiated providing signals which would fit for an intermediate one-side switched NBD and QC bearing species. However, the shape of the formed signals does not look as previously shown for **12b**. Additionally, changing the LED to 475 nm and later 340 nm did not induce further isomerization. Thus, with 425 nm complete conversion into **12** must have occurred already.

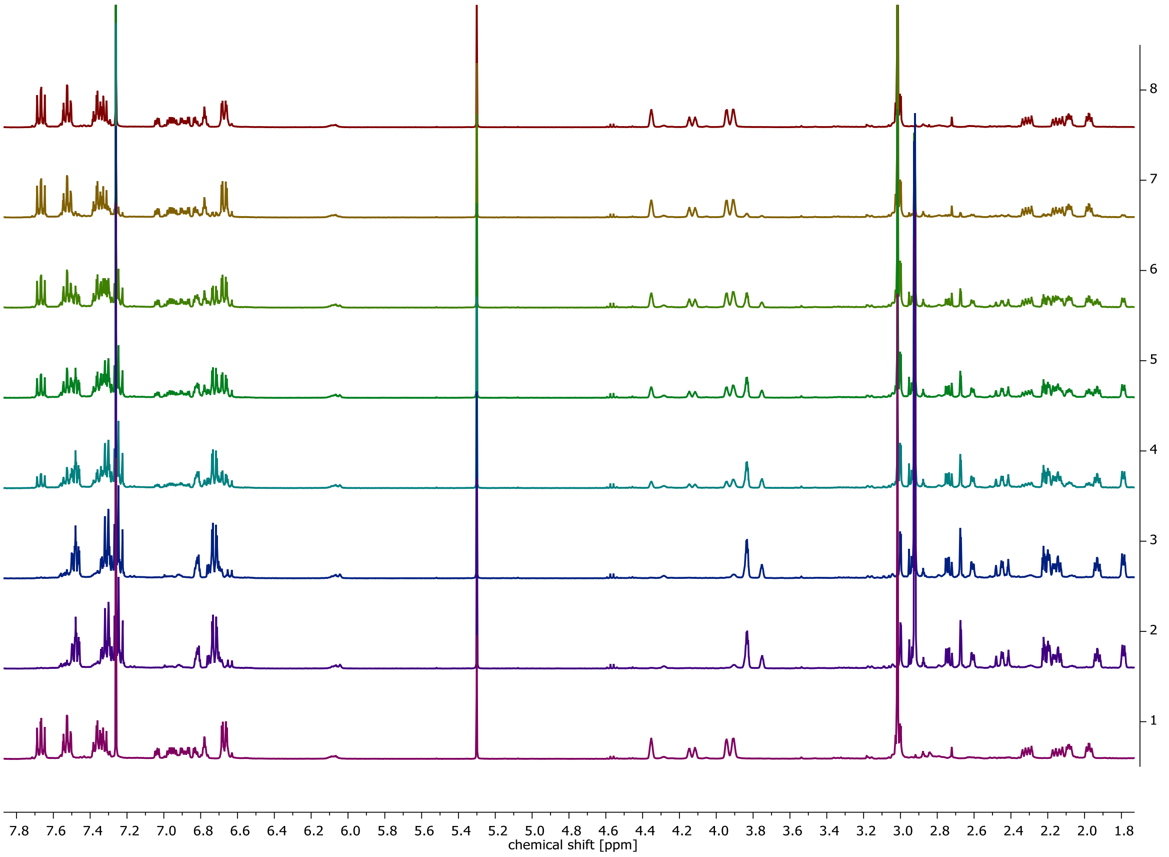

*Figure S 145: ^{1}H NMR switching study of **7** to **12b** and subsequent back-conversion to 7 measured in $CDCl_3$. For the conversion a 475 nm LED was used. The respective spectra can be assigned as follows: 8 = initial NBD spectrum, 7= 2 min irradiation, 6 = 5 min irradiation, 5 = 7.5 min, 4 = 10 min irradiation, 3 = 20 min irradiation, 2 = 50 min irradiation, 1 = addition of **Por**.*

*Table S 17: Corresponding results obtained for the ^{1}H NMR switching study of **7** to **12b** and back to **7** measured in $CDCl_3$.*

Irradiation time	QC [%]
0	0

2 min	24
5 min	52
7.5 min	65
10 min	78
20 min	95
50 min	95
+ **Por**	0

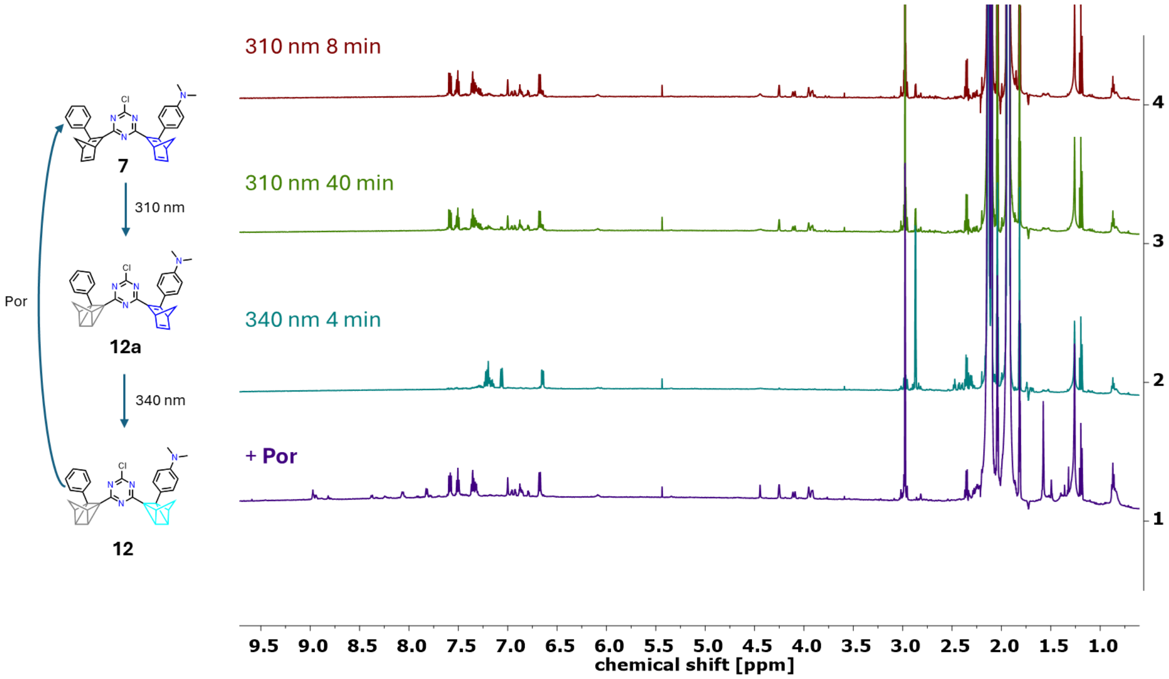

*Figure S 146: ^{1}H NMR switching study of **7** to **12a** and subsequent back-conversion to **7** measured in MeCN-d_3. The experiment was prepared and monitored via UV/Vis spectroscopy and the sample afterwards measured at 600 MHz in the NMR. For the conversion a 310 nm and 340 nm LED was used. The respective spectra can be assigned as follows: = 8 min irradiation at 310 nm, 3 = 40 min irradiation at 310 nm, 2 = 4 min irradiation at 340 nm, 1 = addition of traces **Por**.*

The experiment was prepared on a very small scale and monitored via UV/Vis spectroscopy and the sample afterwards measured at 600 MHz in the NMR. Therefore, only approximately 0.1 mg of **7** was dissolved in the measured NMR sample. Only a very minor indication for the formation of **12a** can be found. Upon irradiation at 340 nm, complete conversion to **12** was achieved, which was reversible after the addition of **Por**. In this scale, the signals of the porphyrin are clearly visible, slightly overshadowing the recovered NBD signals.

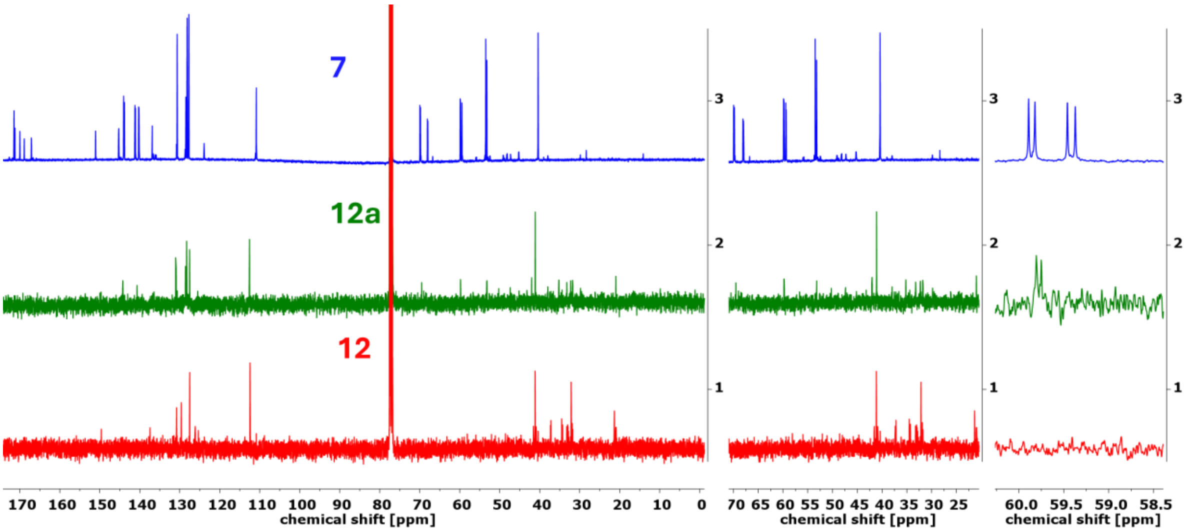

*Figure S 147: Comparison of the ^{13}C NMR spectra of **7** to **12b** and **12** measured in $CDCl_3$.*

4.4.2 Reversible protonation experiments monitored via NMR

Experiments conducted in MeCN-*d*$_3$:

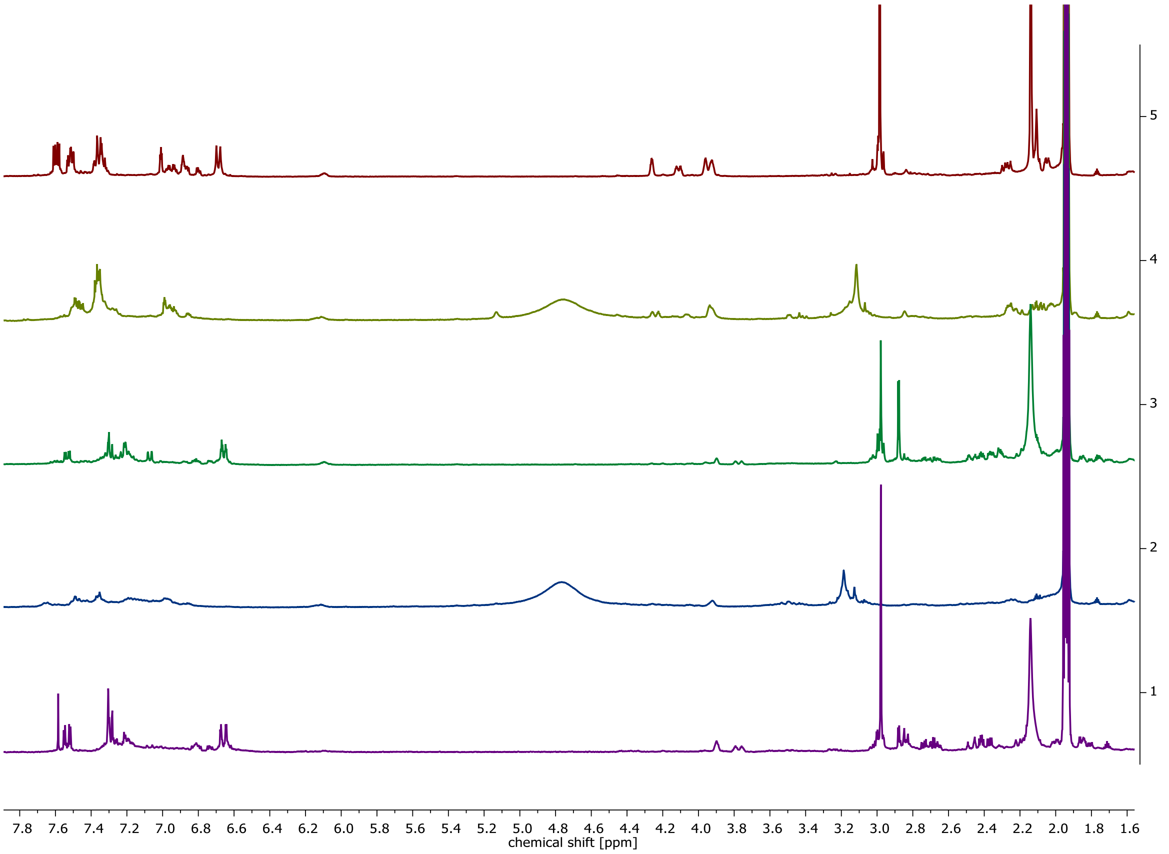

*Figure S 148: ^{1}H NMR reversible protonation study of **7** MeCN-d$_3$. The respective spectra can be assigned as follows: 5 = initial NBD spectrum, 4 = addition of 1 µL TFA-d resulting in **12c**, 3: 35 min irradiation at 340 nm to **12f** (with traces of an NBD species left), 2 = addition of another 1 µL TFA-d, 4 = 4 h irradiation at 475 nm, 1 = spectrum of **12** obtained after consecutive irradiation with 475 nm , 310 nm (75%) and 425 nm (75%).*

The difference in the spectrum 3 (green) and 1 (purple) is barely observable. Therefore, first protonation seems to introduce conversion to 12c and while subsequent irradiation at 340 nm did not result in the formation of **12f** but **12**.

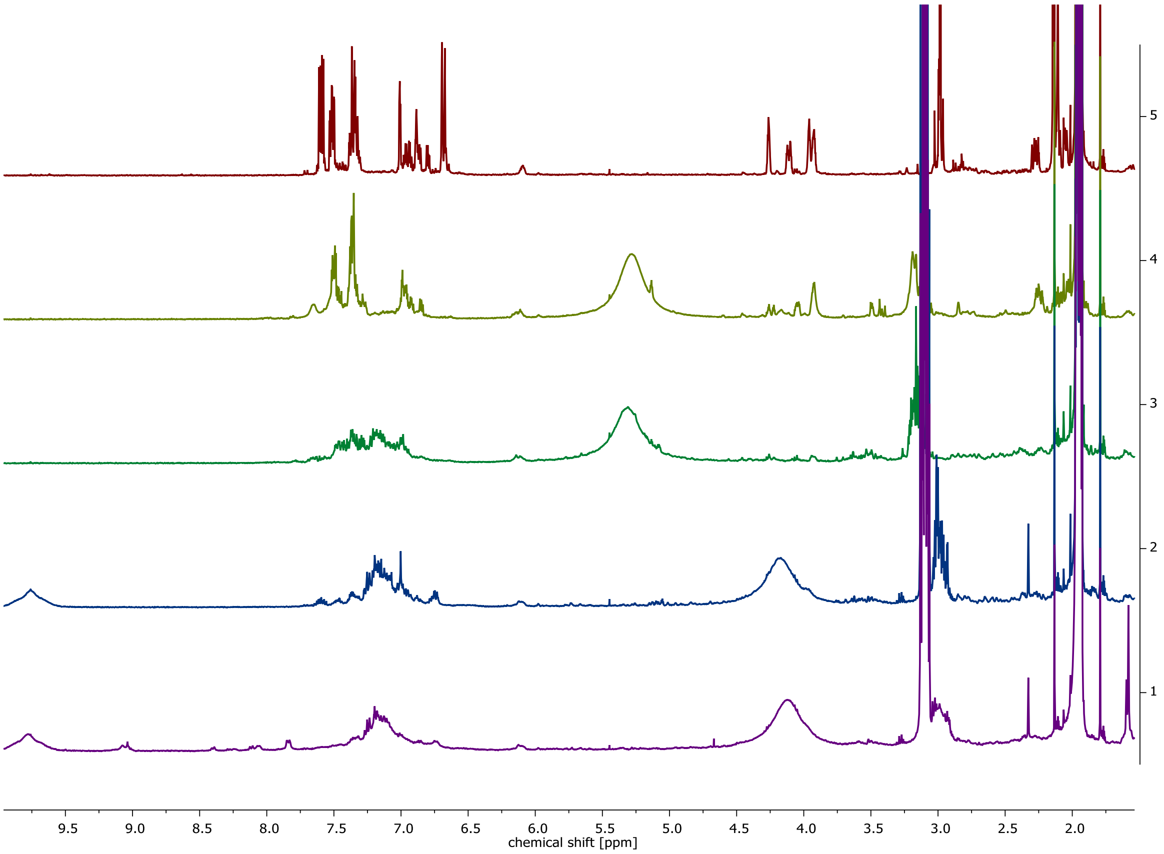

*Figure S 149: ^{1}H NMR reversible protonation study of **7** MeCN-d_3. The respective spectra can be assigned as follows: 5 = initial NBD spectrum, 4 = addition of 1 μL TFA-d resulting in **12c**, 3: 35 min irradiation at 340 nm, 2 = addition of 3 μL NEt_3, 1 = Addition of **Por**.*

During the irradiation with 340 nm of **12c**, mainly photodecomposition seems to occur. Thus, Subsequent addition of base or catalyst (**Por**) did not result in the regain of **12c** or **7**, respectively.

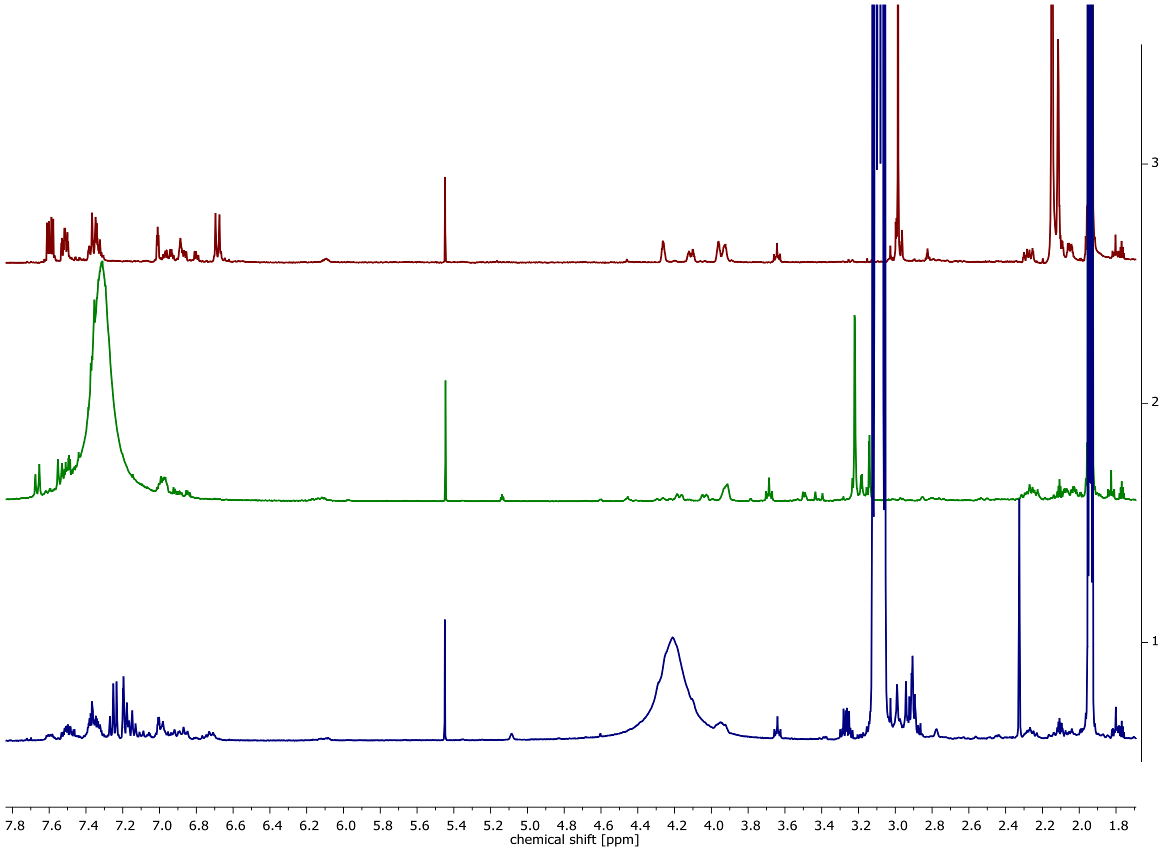

*Figure S 150: ^{1}H NMR reversible protonation study of **7** MeCN-d$_3$. The respective spectra can be assigned as follows: 3 = initial NBD spectrum, 2 = addition of 5 µL TFA (non-deuterated), 1 = addition of 10 µL NEt$_3$.*

First, addition of excessive 5 mL TFA (non-deuterated) induces the conversion to **12c** as found before. However, the aromatic signals are mainly overshadowed by the signal of the acidic proton provided by TFA. Subsequent addition of 10 mL NEt$_3$ did not result in the regeneration of the previously found NBD signals for **7**. Due to the massive excess of acid and base, the whole baseline is disturbed and the signals corresponding to any kind of sample are mainly overlapped.

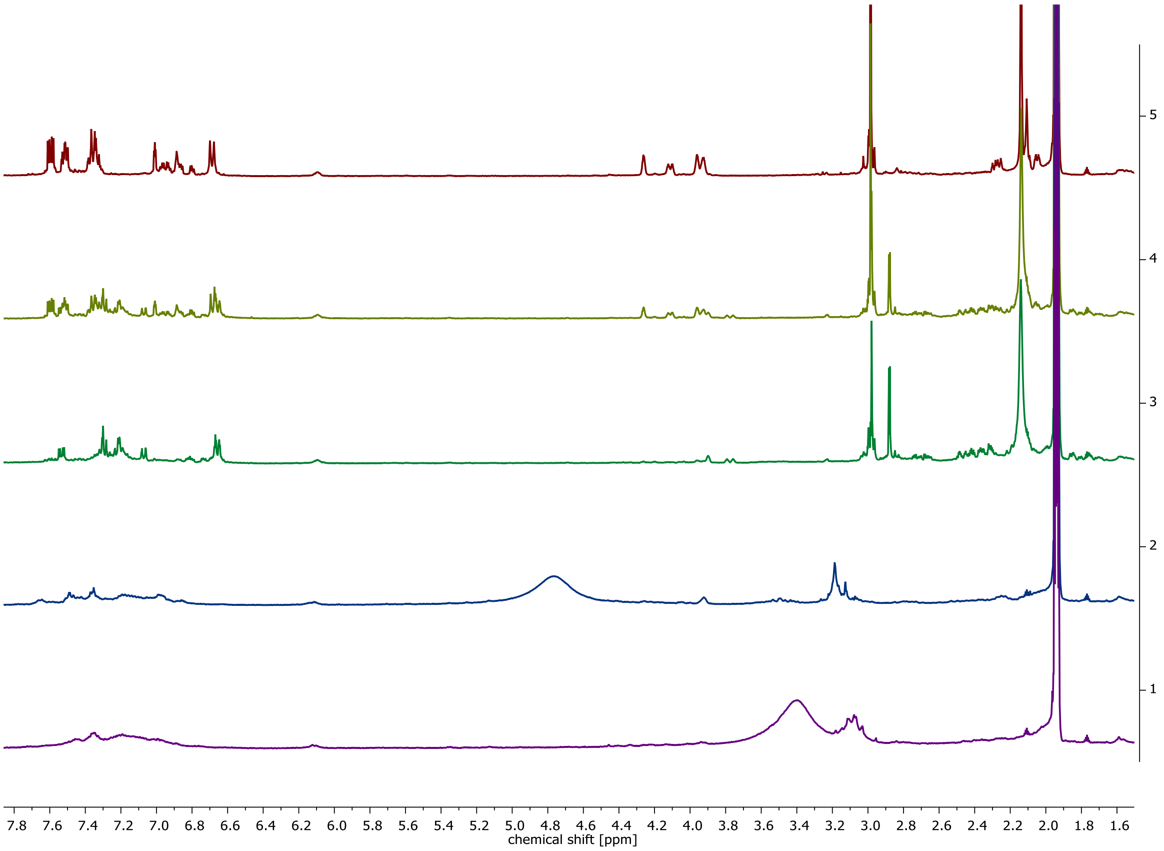

Figure S 151:[1]*H NMR reversible protonation study of* ***7*** *MeCN-d_3. The respective spectra can be assigned as follows: 5 = initial NBD spectrum, 4 = 10 min irradiation at 340 nm yielding partial conversion to* ***12****, 3: 35 min irradiation at 340 nm resulting in* ***12****, 2 = addition of 1 µL TFA (non-deuterated), 1 = isolation of the formed species with subsequent aqueous workup and redissolving in MeCN-d_3.*

Upon addition of TFA, the regain of the significant NBD bridgehead signal at 3.92 ppm of **12c** can be found. However, other significant signals corresponding to either **7** or **12f**, cannot be found. Isolation of the protonated species, including workup and subsequent redissolving in MeCN-*d_3*, did not enhance the quality of the spectrum.

Since the results obtained using MeCN-*d_3* were only partially satisfactory, additional experiments in $CDCl_3$ were performed. Although lacking the possibility for direct comparison to the UV/Vis experiments, additional conclusions considering these measurements were drawn. For these measurements, a fraction of **7** containing partial impurities containing was used. However, the significant signals discussed above are clearly observable in every spectrum.

Experiments conducted in $CDCl_3$:

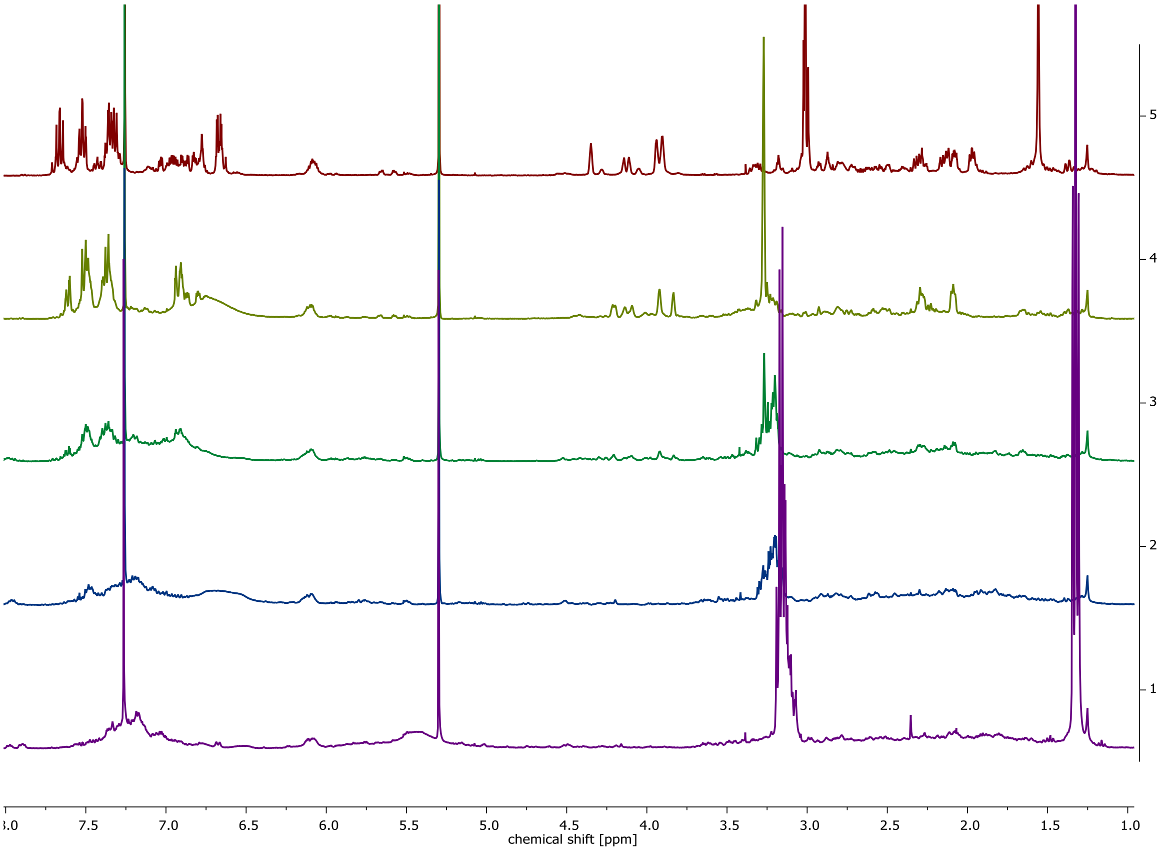

*Figure S 152: ^{1}H NMR reversible protonation study of **7** $CDCl_3$. The respective spectra can be assigned as follows: 5 = initial NBD spectrum, 4 = addition of 1 μL TFA yielding **12c**, 3: 30 min irradiation at 400 nm, 2 = subsequent irradiation at 340 nm for 10 min, 1 = addition of 1.5 μL NEt_3.*

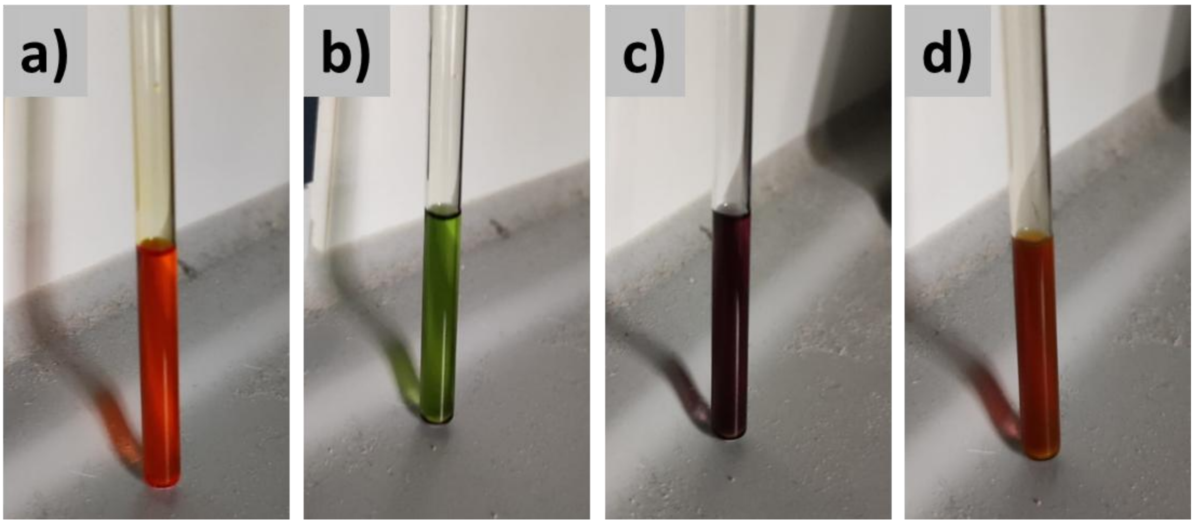

*Figure S 153: Depiction of the color change of the NMR tube during the above-mentioned experiment (Figure S141). a) initial sample of **7** dissolved in $CDCl_3$ (orange). b) Sample after addition of TFA (green). c) After irradiation at 340 nm (deep purple) (during the irradiation at 400 nm, no significant color change occurred). d) After addition of NEt_3 (brownish orange).s*

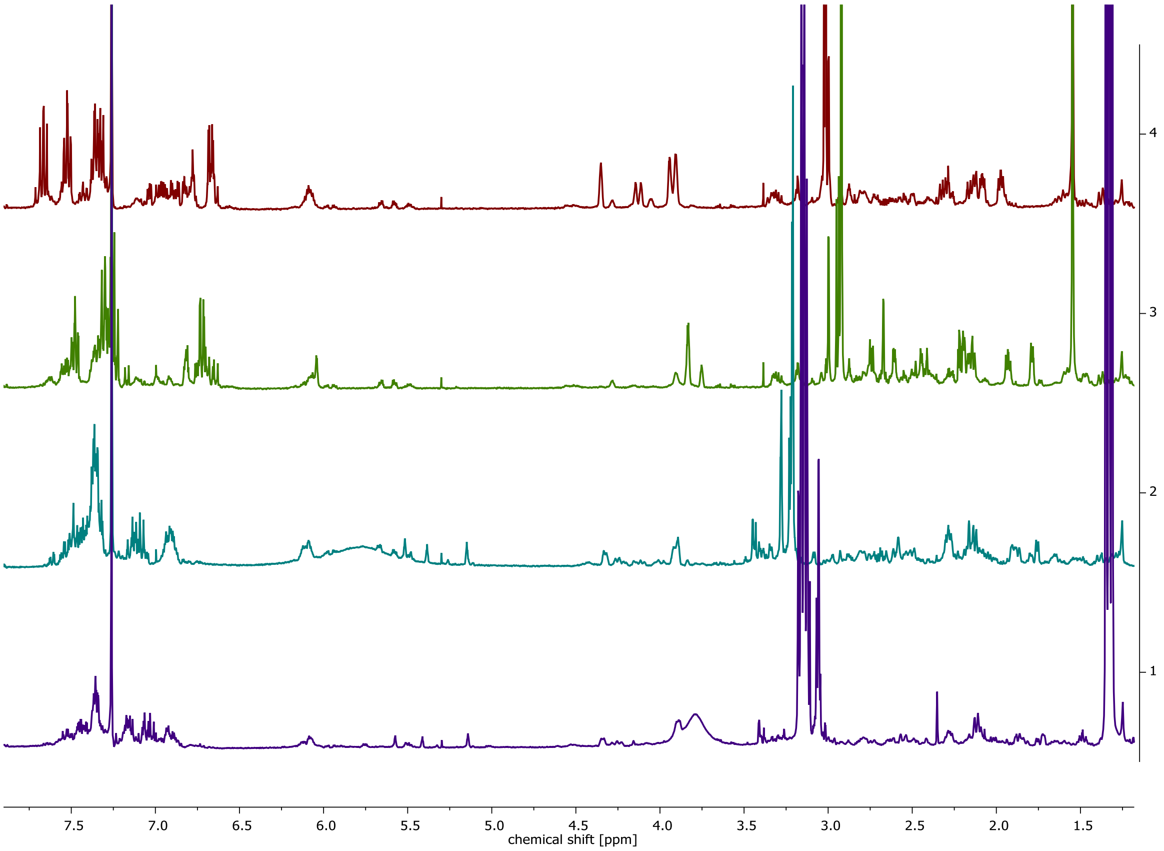

*Figure S 154: ^{1}H NMR reversible protonation study of **7** $CDCl_3$. The respective spectra can be assigned as follows: 4 = initial NBD spectrum, 3 = addition of 1 µL TFA yielding **12c**, 2: 30 min irradiation at 340 nm, 1 = addition of 1.5 µL NEt_3.*

After irradiation at 340 nm, a color change from greenish to colorless was observed. Upon addition of base, a bright yellow color accompanied by fluorescence was generated.

To examine whether back-conversion initiated by protic acids[10],[11] is possible for reversible protonation at the dimethylamine functionality. Therefore, a previously published compound, which was still available in our laboratory, was utilized as a model compound using procedures established for the reversible protonation in toluene.[2],[12] As depicted in *figure S155*, the *N,N*-dimethylaniline functionalized NBD derivative was dissolved in toluene-d_8 and converted to the respective QC derivative after irradiation at 365 nm. Subsequent addition of TFA-*d* did not lead to the back-conversion towards the parent NBD, but in the generation of the protonated QC analogue. Thus, the conclusion is derived that either acid-catalyzed back-isomerization is only possible for distinct derivatives (as indicated for **7**) or is strongly solvent dependent. However, a solvent dependency for this phenomenon was not discussed in literature, and the findings for MeCN and $CDCl_3$ were similar as described.

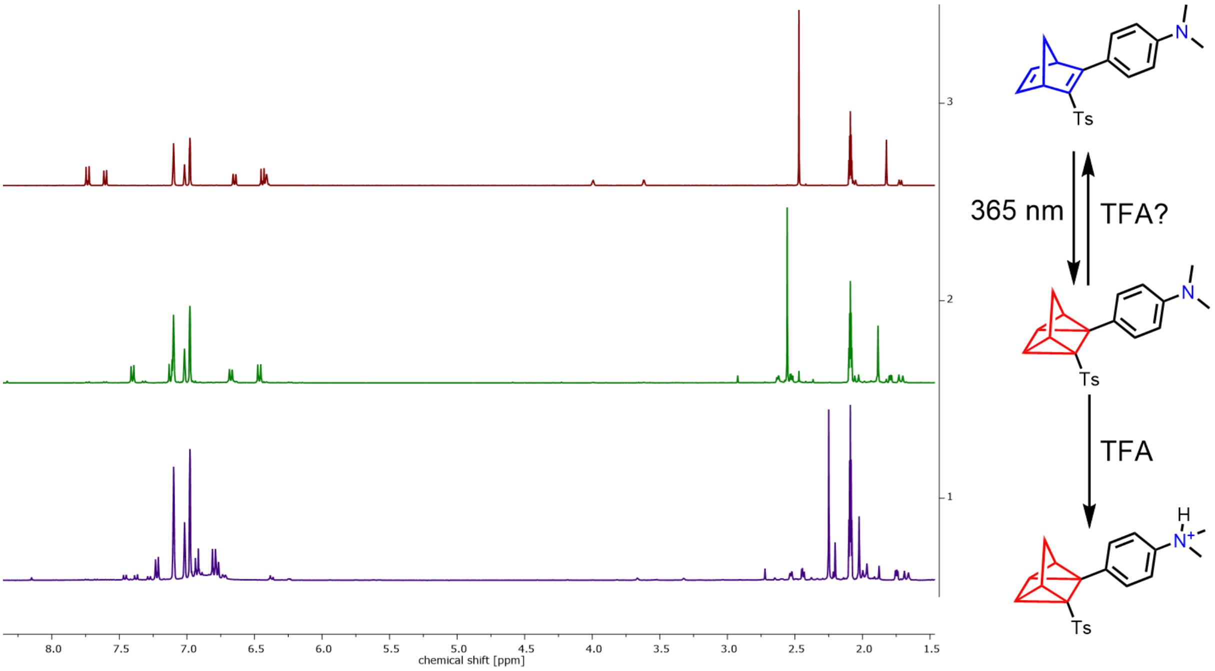

Figure S 155: ^{1}H NMR protonation study of the shown model compound[2] measured in toluene-d_8. The respective spectra can be assigned as follows: 3 = initial NBD spectrum, 2 = Spectrum of the corresponding QC after irradiation at 365 nm, 1 = addition of 1 µL TFA-d resulting in the formation of the protonated QC analogue.

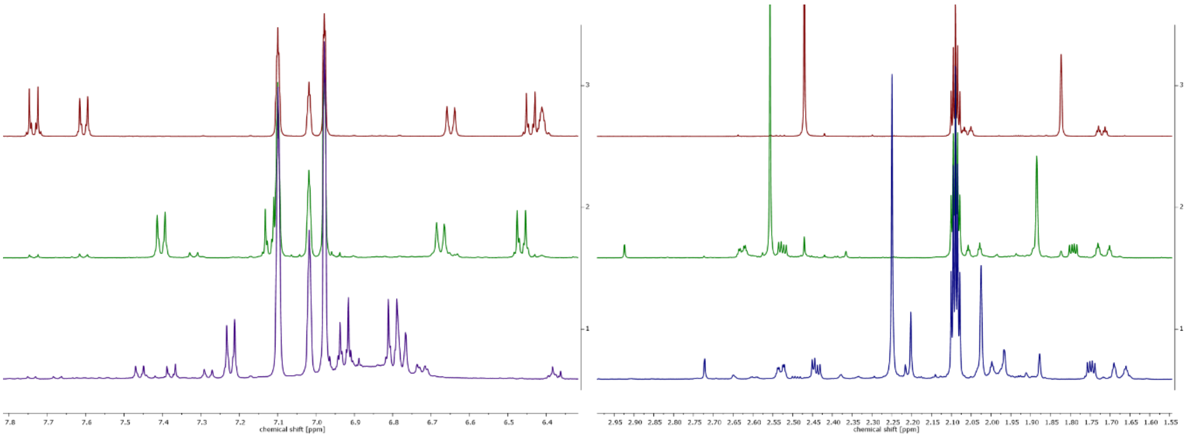

Figure S 156: Zoom of the aromatic and aliphatic region of the measurement above (Figure S144).

4.5 ***N,N*-dimethylaniline-anisyl-phenyl-tris-NBD hybrid:** NBD **8** to QC **13**

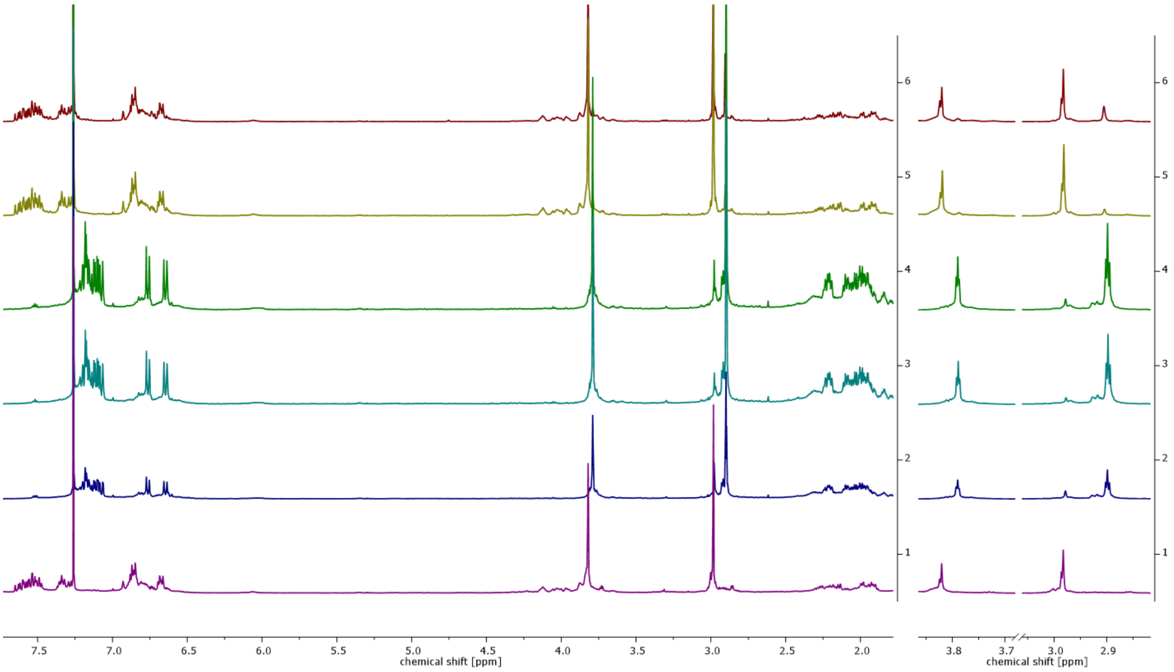

*Figure S 157: ^{1}H NMR switching study of **8** to **13** measured in $CDCl_3$. On the right side a zoom of the section of the OMe and NMe_2 signals is provided. For the conversion a 310 nm and a 365 nm consecutively used. The respective spectra can be assigned as follows: 6 = initial NBD spectrum, 5 = 10 min irradiation at 310 nm (80%, 15 °C), 4 = 5 min irradiation at 365 nm (80%, 15 °C), 3 = 10 min irradiation at 365 nm (80%, 15 °C), 2 = leaving the sample at rt for 23 h, 1 = addition of **Por**.*

*Table S 18: Corresponding results obtained for the ^{1}H NMR switching study of **8** to **13** measured in $CDCl_3$.*

Irradiation time	QC [%]
0	0
10 min (310 nm)	0
5 min (365 nm)	100
10 min (365 nm)	100
23 h at rt	100
+ **Por**	0

Due to the complexity of the spectrum (including signals for all diastereoisomers), proper integration is barely possible. Therefore, either complete conversion or back-isomerization can be claimed. Furthermore, thermal stability can be assumed as described in the main manuscript.

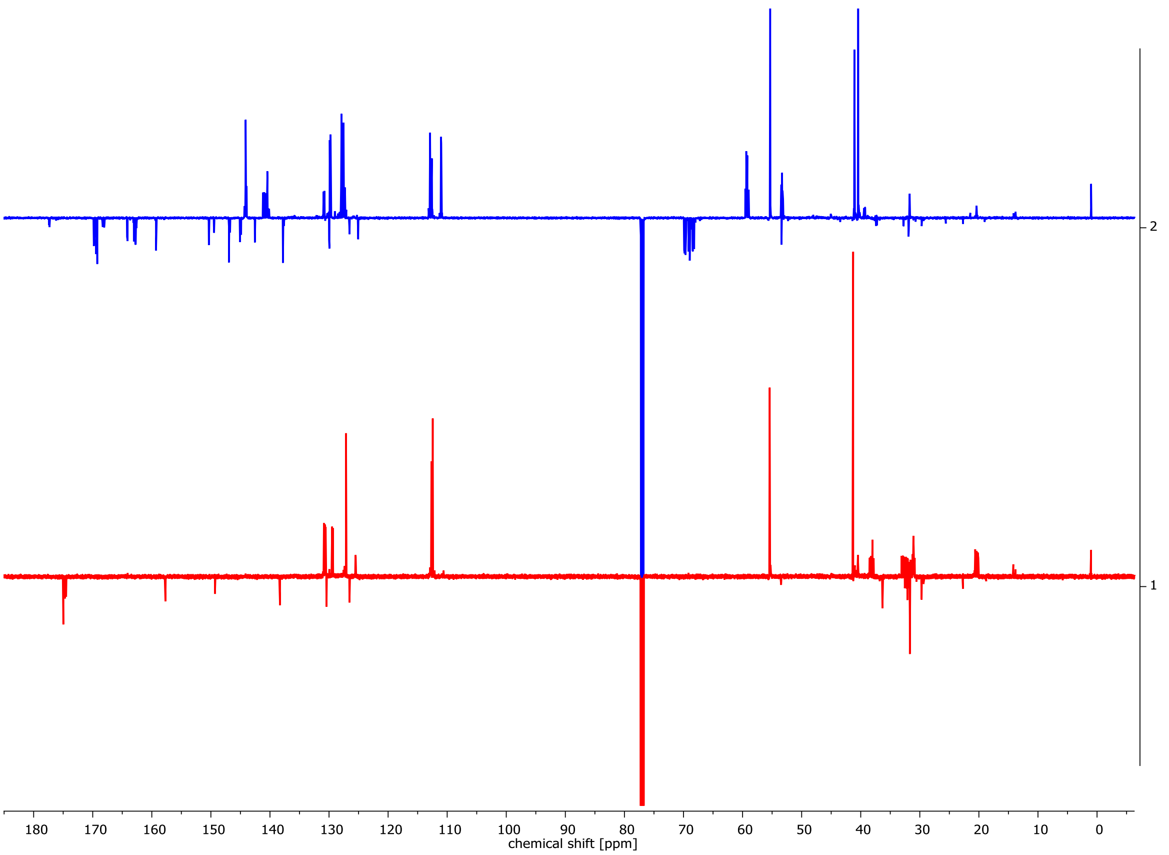

*Figure S 158: Comparison of the deptq spectra recorded of **8** (top, 25 °C) and **13** (bottom, 10 °C) recorded at 600 MHz in $CDCl_3$.*

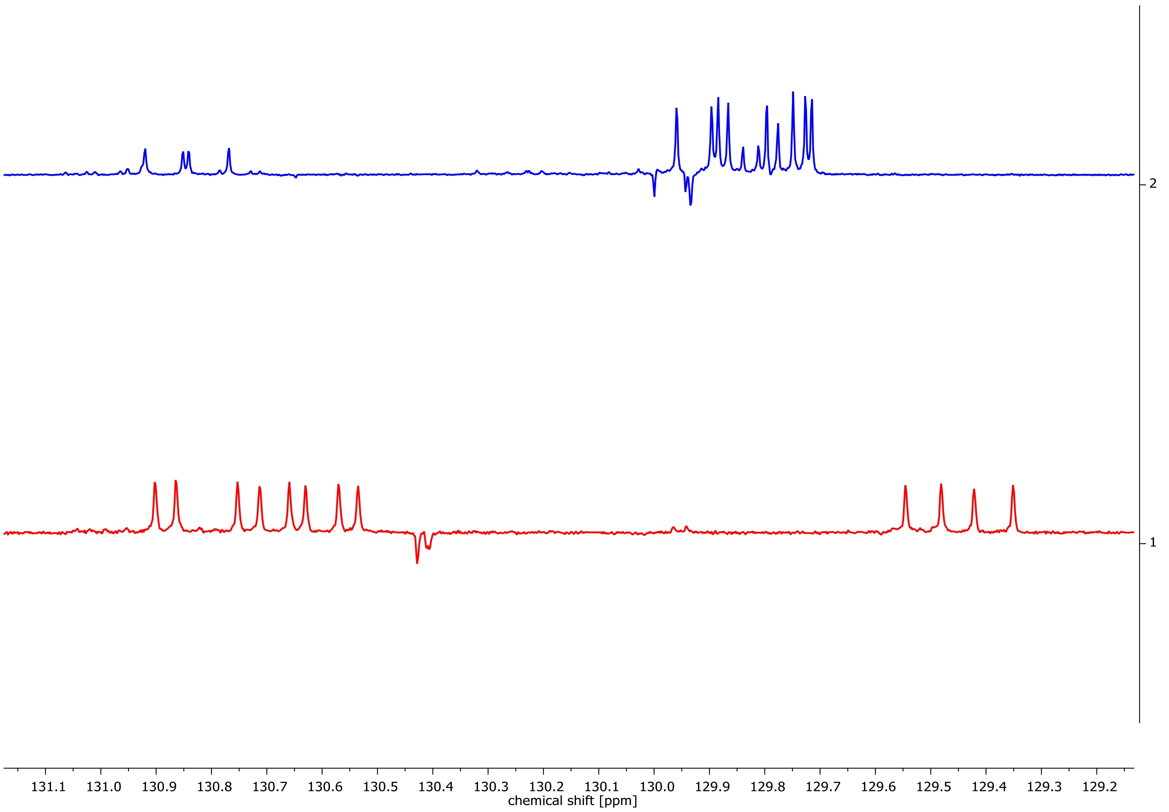

*Figure S 159: Significant section of the deptq shown above for **8** and **13** to elucidate to 4-fold signal splitting for each carbon signal resulted by the presence of 4 diastereoisomers.*

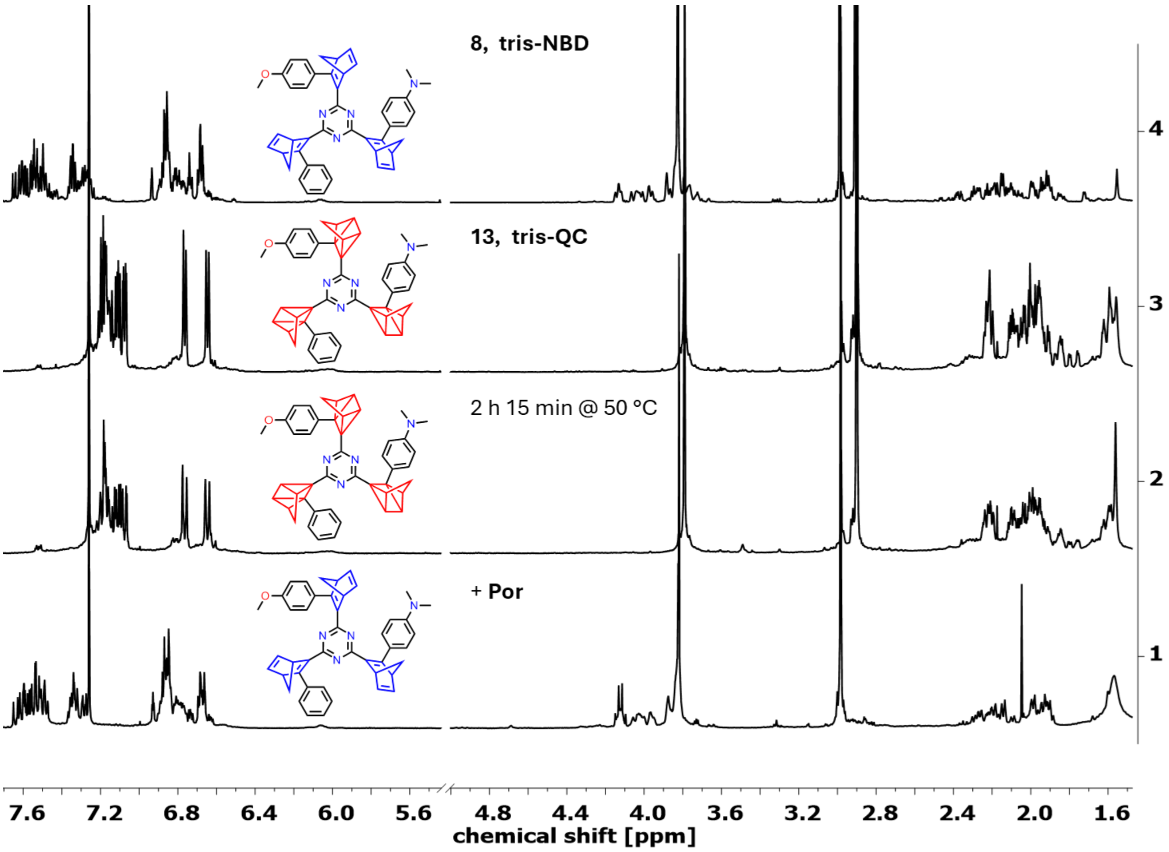

Figure S 160: ^{1}H NMR spectra of **8** and **13** measured in $CDCl_3$. 4: all NBD derivative **8**; 3: all QC derivative **13** after irradiation at 365 nm (80%, 5 min, 15 °C); 2: 50 °C for 2 h and 15 minutes → spectrum remains unchanged and still shows **13**; 1: back-conversion to **8** through addition of 0.1 mg **Por**.

5 Switching studies monitored via UV/Vis spectroscopy

5.1 NBD **4** to QC **9**

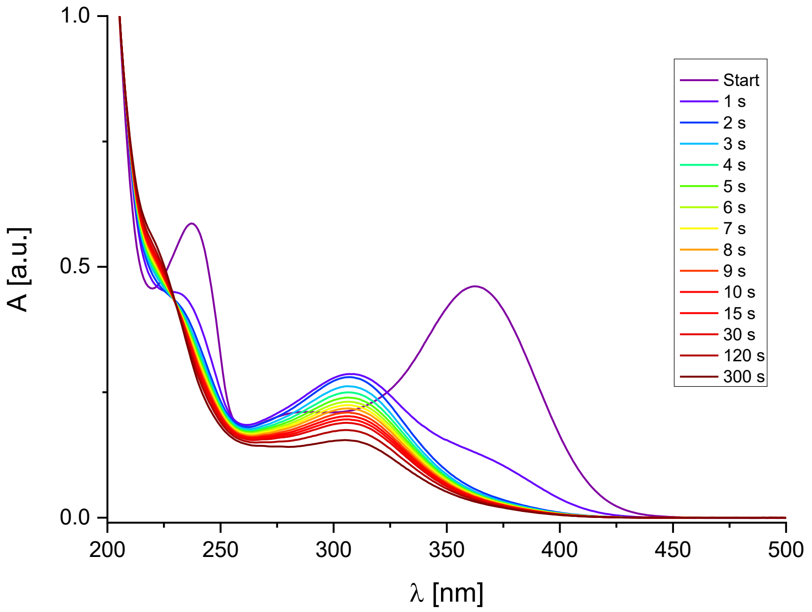

*Figure S 161: UV/Vis switching study of **4** measured in MeCN. For the irradiation a 340 nm LED was used. Prolonged irradiation led to photodecomposition.*

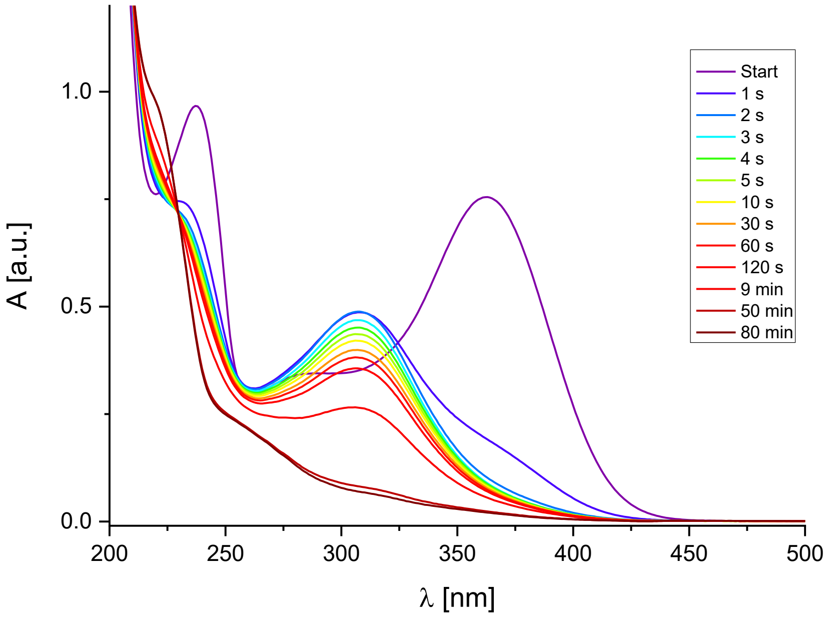

*Figure S 162: UV/Vis switching study of **4** measured in MeCN. For the irradiation a 367 nm LED was used. Prolonged irradiation led to photodecomposition.*

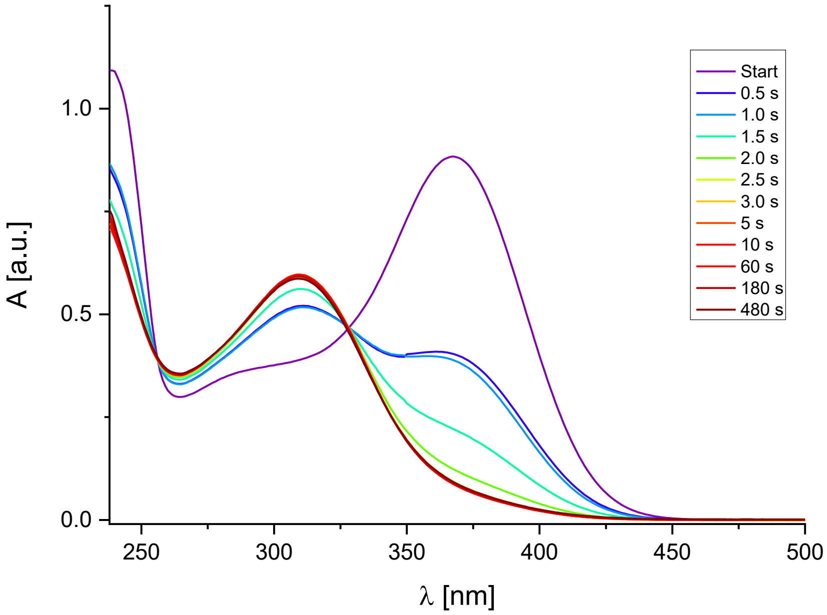

*Figure S 163: UV/Vis switching study of **4** measured in $CHCl_3$. For the irradiation a 367 nm LED was used. The absorption of the NBD species (λ_{max} = 367 nm) decreases with the simultaneous increase of the QC absorption at λ_{max} = 309 nm. Approximately 50% isomerization occurred already after just 0.5 seconds demonstrating, an impressively fast conversion. Even after prolonged irradiation up to 8 minutes, no significant photodecomposition could be observed. The presence of isosbestic points indicates clean conversion.*

5.2 NBD **5** to QC **10**

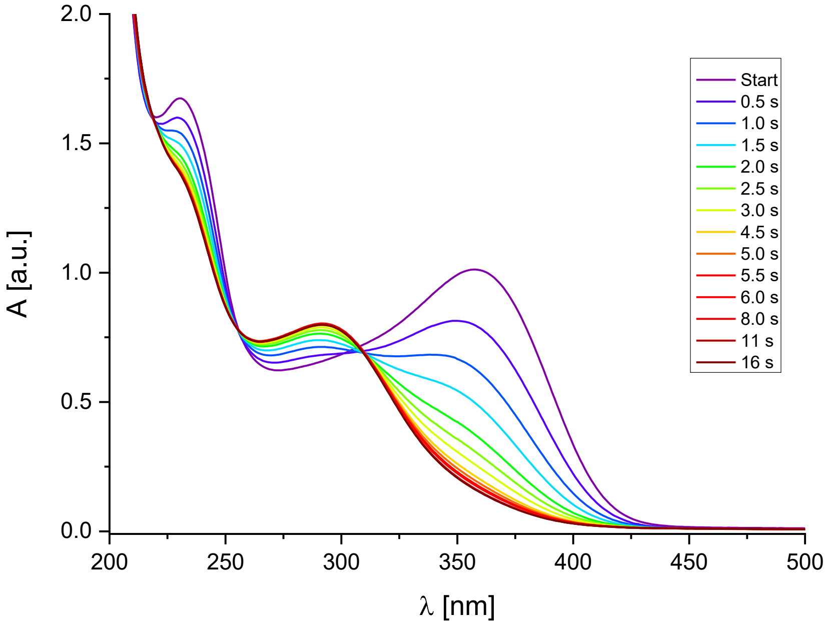

*Figure S 164: UV/Vis switching study of **5** measured in MeCN. For the irradiation a 367 nm LED was used.*

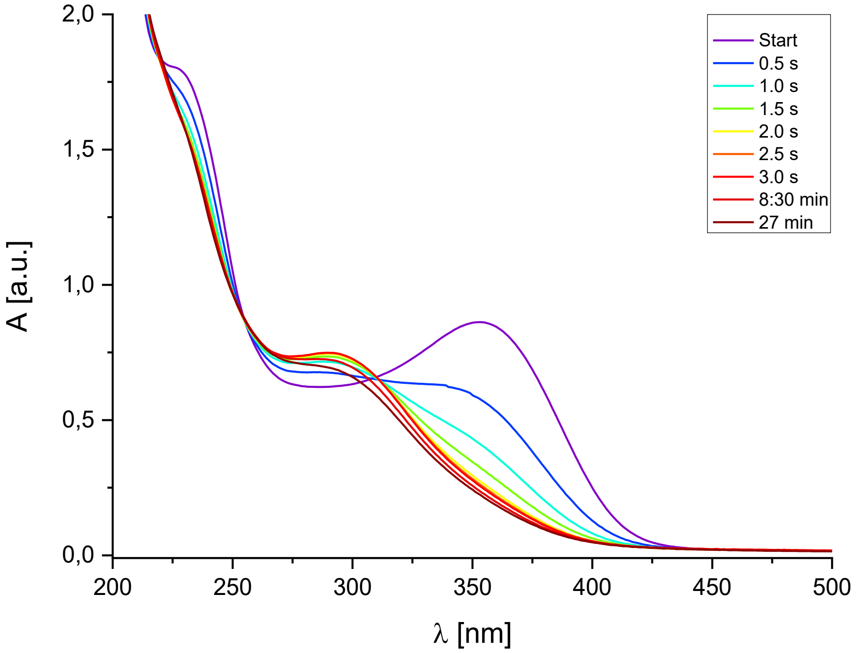

*Figure S 165: UV/Vis switching study of **5** measured in MeCN. For the irradiation a 367 nm LED was used. Prolonged irradiation led to slight photodecomposition*

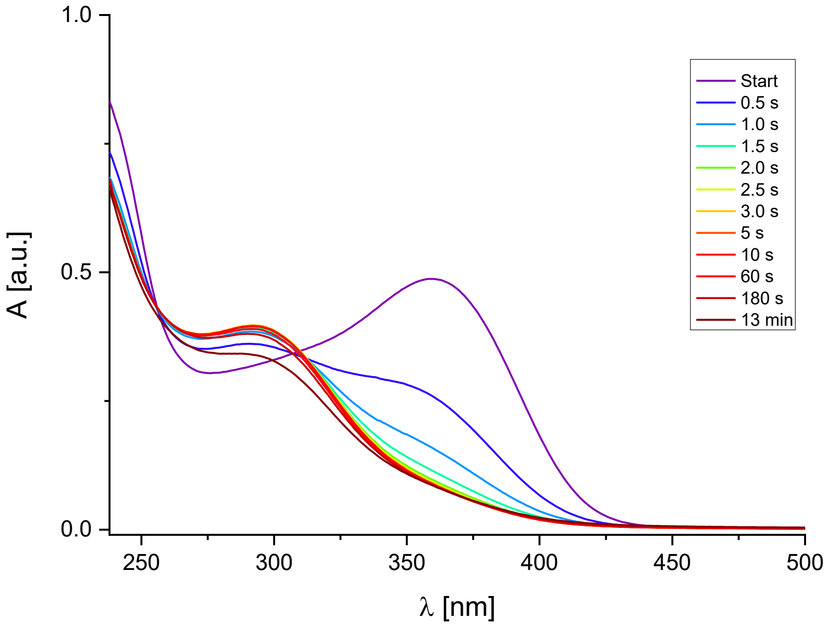

*Figure S 166: UV/Vis switching study of **5** measured in $CHCl_3$. For the irradiation a 367 nm LED was used. Prolonged irradiation led to slight decomposition.*

5.3 NBD **6** to QC **11**

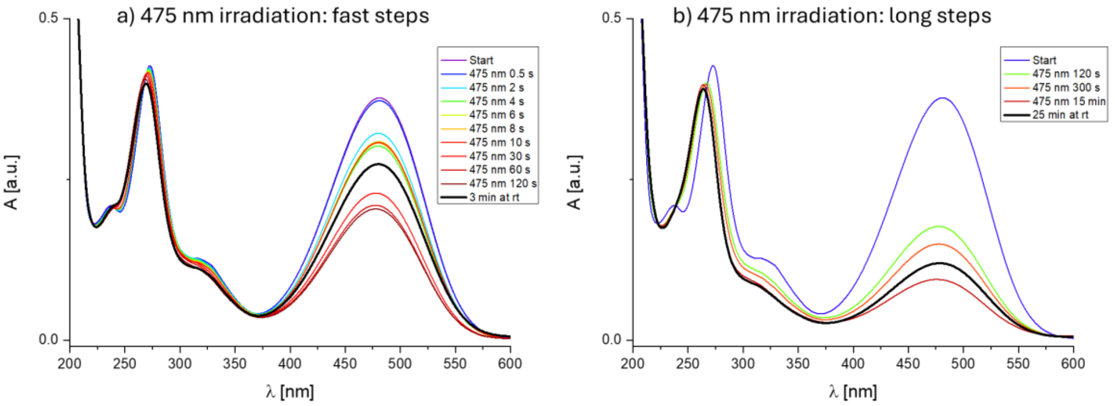

*Figure S 167: UV/Vis switching study of **6** measured in MeCN. For the irradiation a 475 nm LED was used. a) Irradiation at 475 nm with short time steps. After 120s, the sample was let at rt for 3 minutes without irradiation. b) Irradiation at 475 nm at longer, consecutive time steps. After 15 min, the sample was let at rt for 25 minutes without irradiation. In both cases, regeneration of the absorption band around 482 was observed upon standing at rt, while regain of the initial NBD absorption was never found.*

During the irradiation process, a 475 nm LED was first used to initiate a decrease of the absorption at 482 nm, indicating formation into QC **11**. After letting the sample rest at rt, partial back-conversion could be assumed. Repeated irradiation and reconversion led to the same results, considering the longer wavelength absorption. However, neither isosbestic points nor increase of a new formed QC absorption could be found. Only the previous absorption at 273 nm shifted stepwise to 264 nm while the absorption intensity decreased permanently. Therefore, combined with the results obtained via NMR spectroscopy, the formation of **11** cannot be claimed as successful.

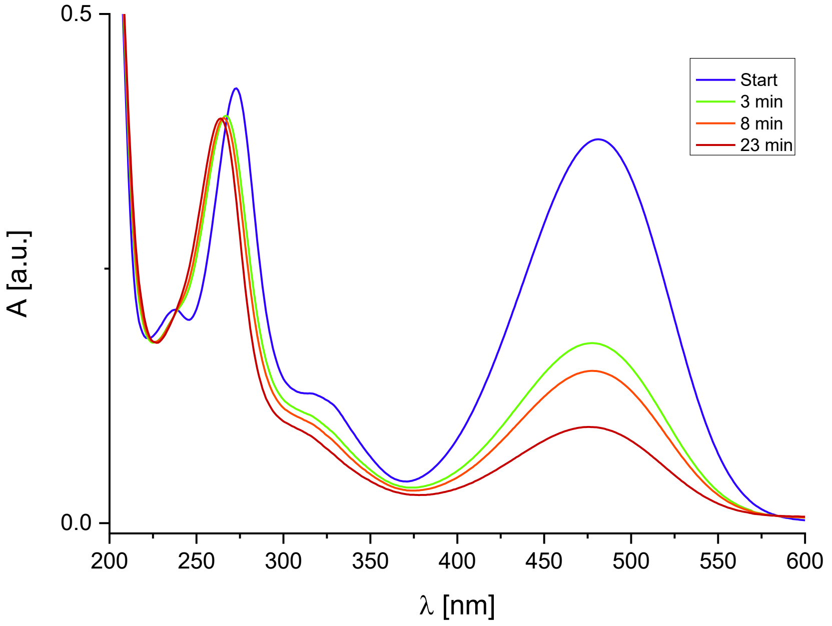

*Figure S 168: UV/Vis switching study of **6** measured in MeCN for extended times. For the irradiation a 475 nm LED was used.*

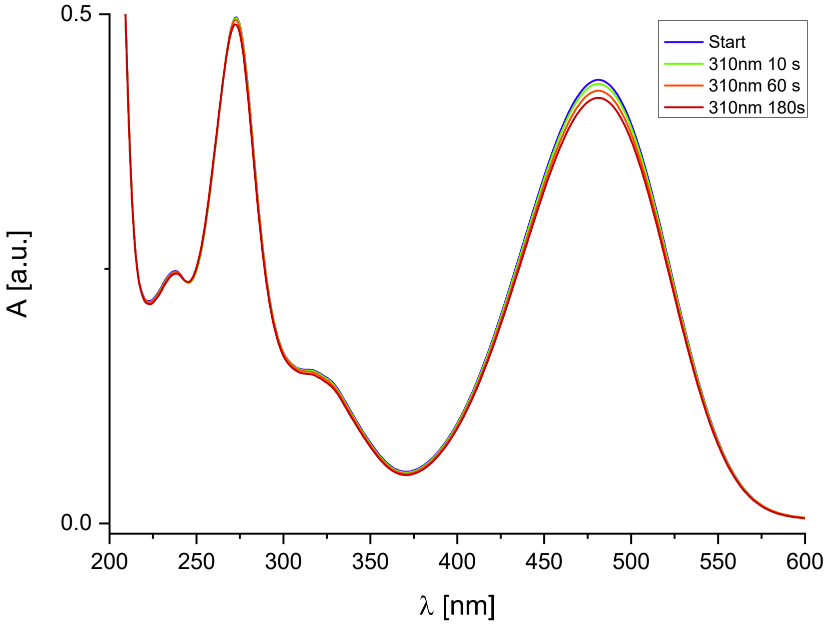

*Figure S 169: UV/Vis switching study of **6** measured in MeCN. For the irradiation a 310 nm LED was used. Barely any significant change could be observed. Therefore, neither switching nor photodecomposition is induced at this irradiation wavelength.*

5.4 NBD **7** to QC **12** (and single side switched intermediate species **12a** and **12b**)

5.4.1 **Switching experiments of the normal states**

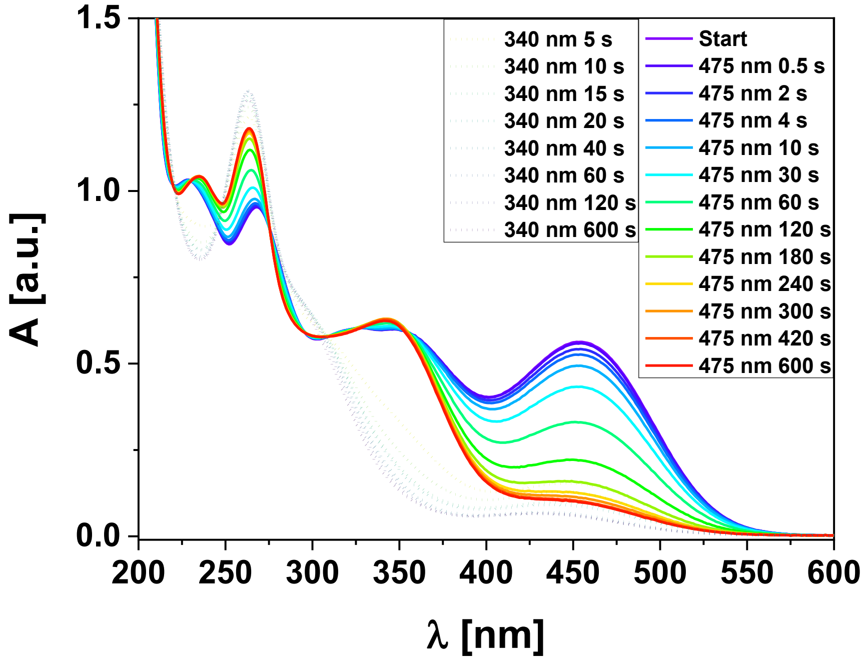

*Figure S 170: UV/Vis switching study of **7** measured in MeCN. Irradiation using 475 nm first to get **12b** and subsequently 340 nm to obtain **12**.*

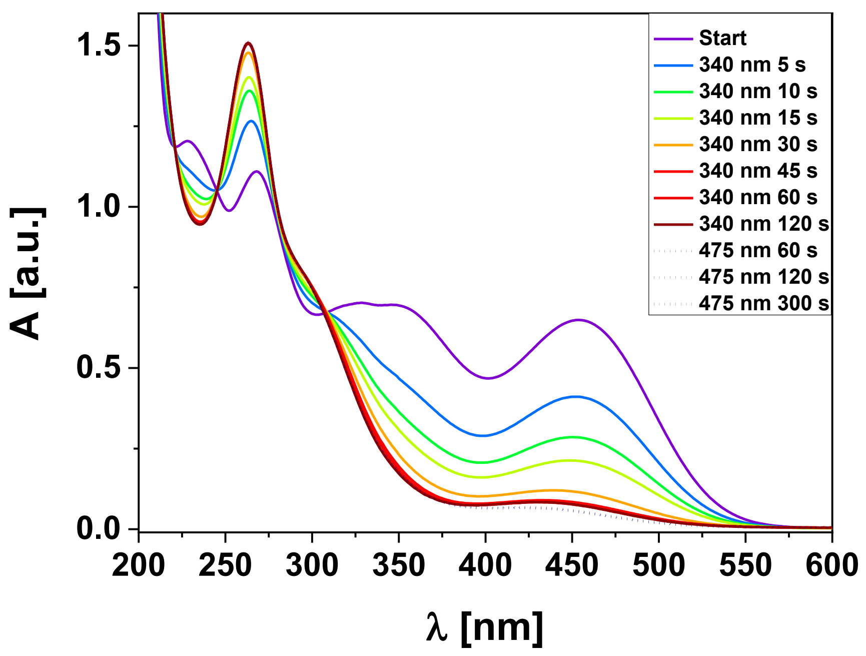

*Figure S 171: UV/Vis switching study of **7** measured in MeCN. Direct irradiation at 340 nm to get **12** and 475 nm afterwards to check for further changes.*

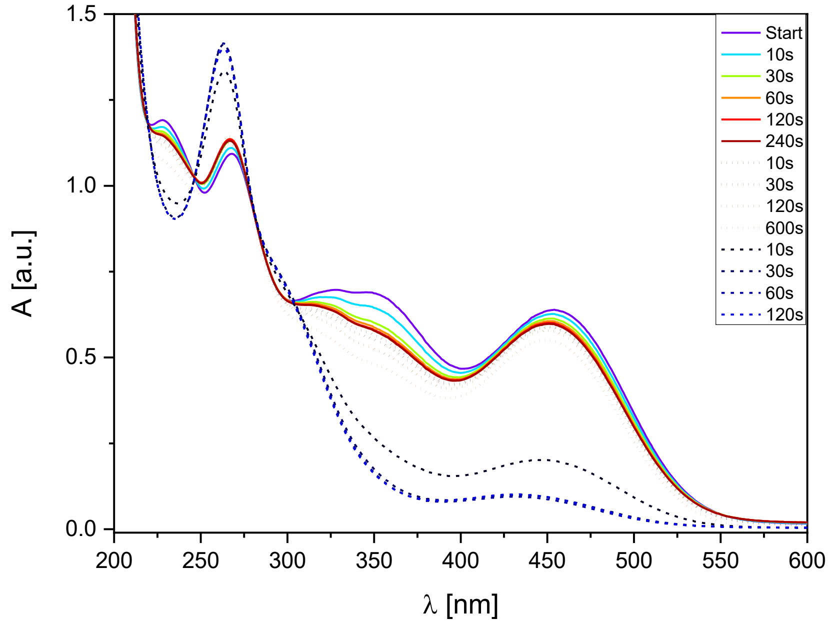

*Figure S 172: UV/Vis switching study of **7** measured in MeCN. To get **12a** using first 275 nm (solid lines), second 310 nm (dotted lines) and last 340 nm (dashed lines) to obtain **12**.*

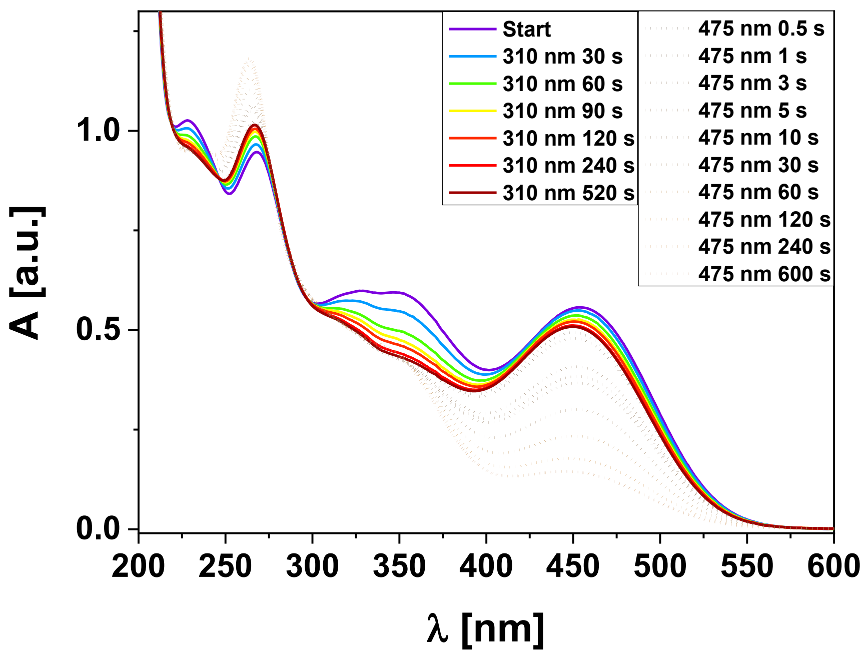

*Figure S 173: UV/Vis switching study of **7** measured in MeCN. First 310 nm to get **12a** and second 475 nm to obtain **12**. With this sequence a mixture of **12a**, **12b** and **12** can be expected in the sample.*

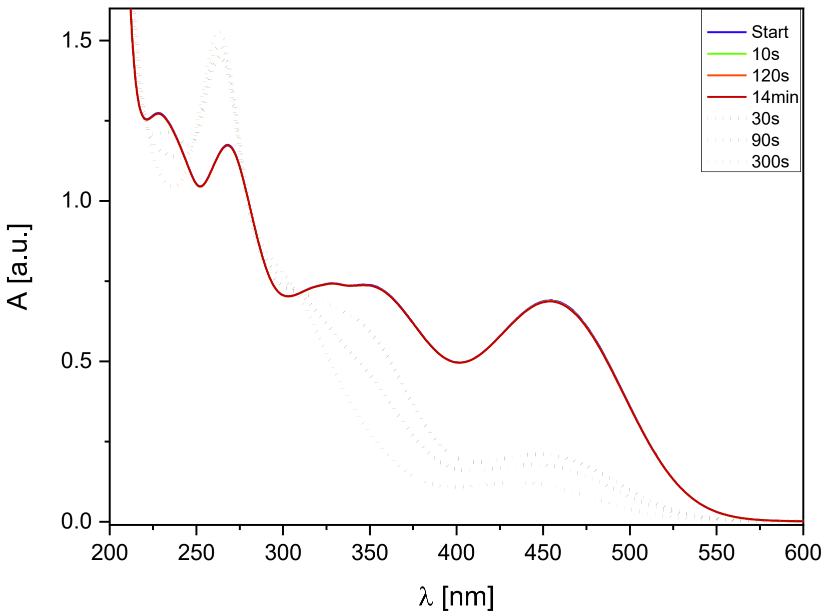

*Figure S 174: UV/Vis switching study of **7** measured in MeCN. First 525 nm which induced no chance and afterwards irradiation using a high-power flashlight converting the sample into all QC form **12**.*

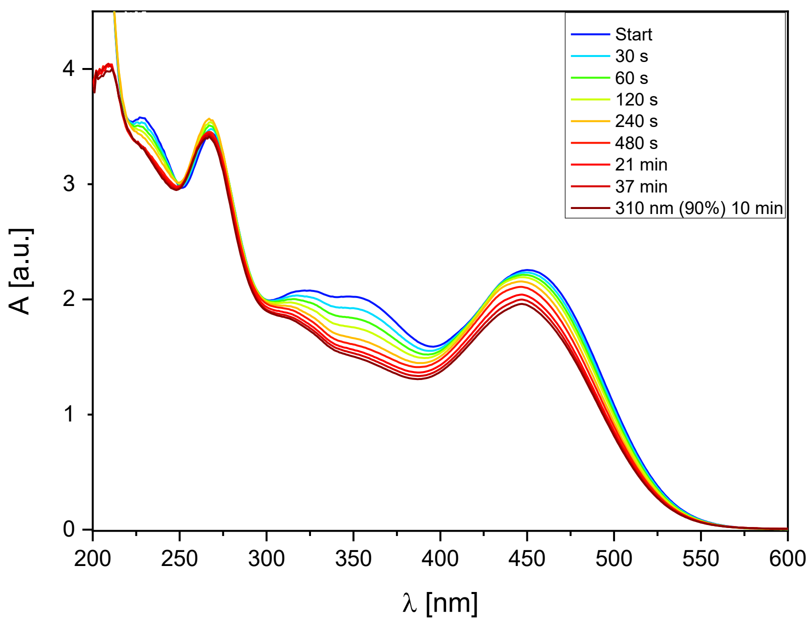

*Figure S 175: UV/Vis switching study of **7** measured in MeCN. Reproduction of the previous experiment using a 310 nm LED to get **12a** with prolonged times and chance to the high-power 310 nm LED block (90%, 15 °C) of the Lucent360 photoreactor.*

As found before, irradiation at 310 nm seems to induce single-sided conversion into **12a** since the absorption in this region decreases while the bathochromic absorption around 450 nm remains relatively stable. However, utilizing the higher-power LED block (310 nm, 90%, 15 °C) only led to decrease of the overall absorption giving indication or photodecomposition.

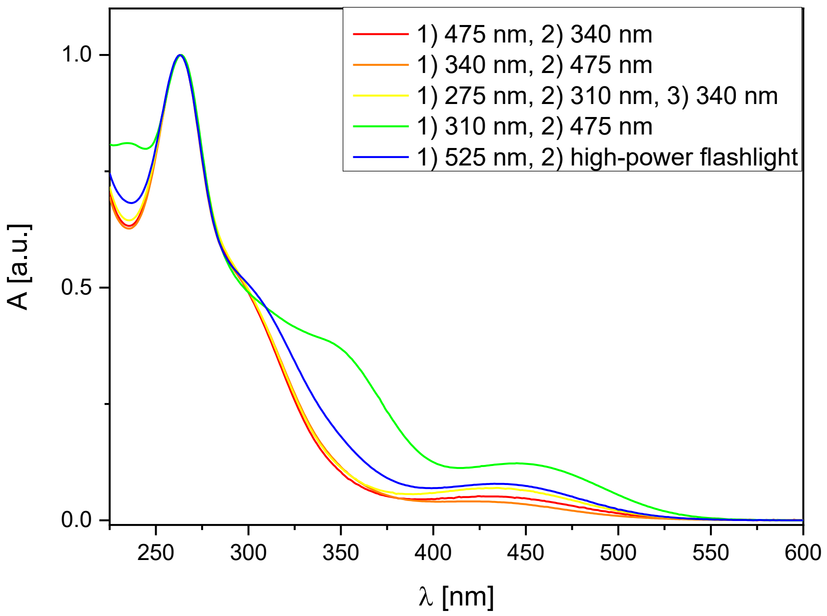

*Figure S 176: Normalized end spectra of all UV/Vis switching studies of **7** measured in MeCN.*

Only for the irradiation experiment in which no 340 nm LED was used (green line), a leftover absorption at 347 nm can be observed. Compared to the spectrum of **12b** and in accordance with calculation results, residues of **12b** are suggested. Therefore, irradiation at 340 nm seems to be essential to trigger complete conversion to **12**.

5.4.2 Reversible protonation experiments towards 12c-12f

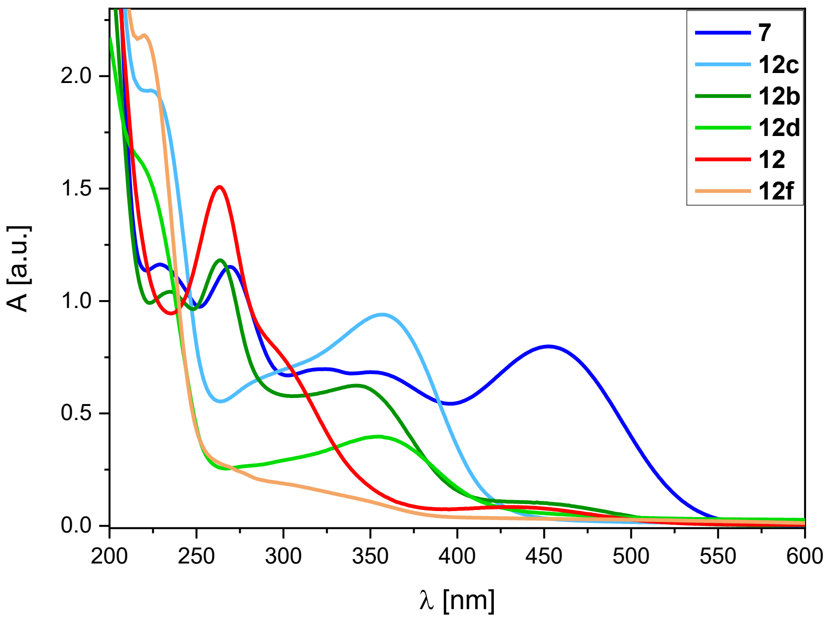

*Figure S 177: UV/Vis spectra of **7, 12b** and **12** and their respective protonated analogs **12c, 12d** and **12f** measured in MeCN.*

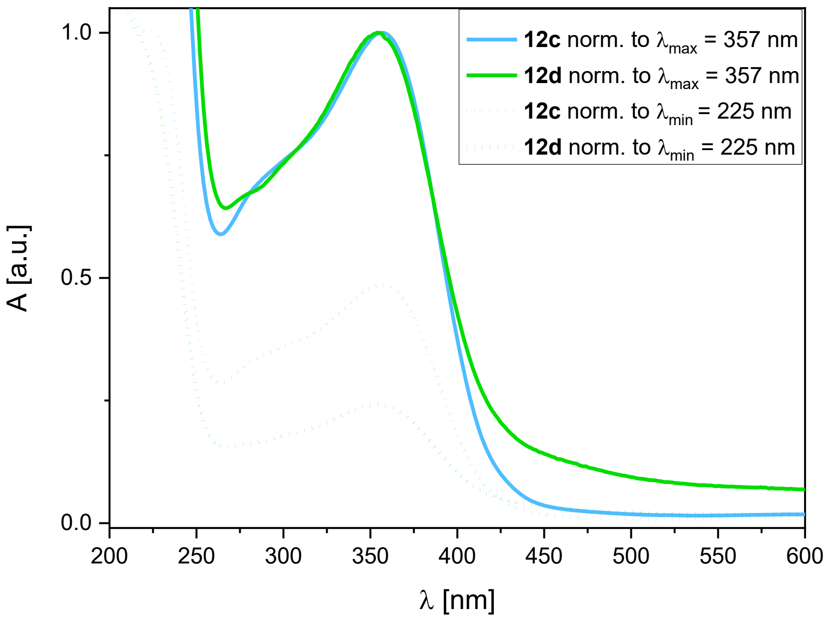

*Figure S 178: Comparison of the normalized UV/Vis spectra of **12c** and **12d** measured in MeCN. Solid lines are normalized to the λ_{max} = 357 nm absorption, while the dotted lines are normalized to the λ_{min} = 225 nm absorption.*

Combined with a different line shape, a shift of both the λ_{max} and λ_{min} values for both derivatives results in different shifts. Therefore, both spectra can be assigned to different species, as additionally supported by the results found by theoretical calculations.

Table S 19: This table is an extension of the table 2 of the manuscript and is just shown here again for easier comparison. Input possibilities starting from the basic form ***7*** *(0;0). All associated UV/Vis spectra were measured in MeCN at rt and provided in the SI. Text in red indicates if species were found not matching the expectations. [a] similar measurement to entry 5 but during addition of the specific inputs, the solution containing cuvette was cooled to 0 °C to exclude acid-based heat catalyzed back-conversion [c] the cuvette was placed in front of the window for 1 h on a slightly cloudy day (06.05.2024).*

Input Sequence	Input[a]	Resulting Species
**1**	TFA (10 μL)	**12c** (*0;0-H*$^+$)
	NEt(iPr)$_2$ (20 μL)	**7** (*0;0*)
	TFA (20 μL)	**12c** (*0;0-H*$^+$)
	NEt(iPr)$_2$ (20 μL)	**7** (*0;0*)
	TFA (50 μL)	**12c** (*0;0-H*$^+$)
	NEt(iPr)$_2$ (100 μL)	**7** (*0;0*)
	475 nm	**12b** (*0;1*)
	**Por**	/
**2**	340 nm	**12** (*1;1*)
	TFA (10 μL)	**12c** (*0;0-H*$^+$)
	NEt$_3$ (20 μL)	**7** (*0;0*)
**3**	340 nm	**12** (*1;1*)
	NEt$_3$ (10 μL)	**12** (*1;1*)
	TFA (20 μL)	**12c** (*0;0-H*$^+$)
	NEt$_3$ (10 μL)	**7** (*0;0*)
**4[b]**	340 nm	**12** (*1;1*)
	TFA (10 μL)	**12c** (*0;0-H*$^+$)
	NEt$_3$ (20 μL)	**7** (*0;0*)
**5**	475 nm	**12b** (*0;1*)
	TFA (10 μL)	**12d** (*0;1-H*$^+$)
	340 nm	**12f** (*1;1-H*$^+$)
	NEt$_3$ (20 μL)	/
**6**	TFA (10 μL)	**12c** (*0;0-H*$^+$)
	340 nm	**12f** (*1;1-H*$^+$)
	**Por**	/
	NEt$_3$ (50 μL)	/
**7**	475 nm	**12b** (*0;1*)
	TFA (10 μL)	**12d** (*0;1-H*$^+$)
	NEt$_3$ (20 μL)	**7** (*0;0*)
**8**	TFA (10 μL)	**12c** (*0;0-H*$^+$)
	475 nm	**12d** (*0;1-H*$^+$)
	NEt$_3$ (20 μL)	**7** (*0;0*)
**9**	Daylight[c]	**12** (*1;1*)
	TFA (10 μL)	**12c** (*0;0-H*$^+$)
	**Por**	**12c** (*0;0-H*$^+$)
	NEt(iPr)$_2$ (20 μL)	**7** (*0;0*)

We also investigated the accessibility of specific states *via* various combinations of alternative routes. An overview of the various inputs that we tested are listed in *table S19*. All measurements were con-

ducted in MeCN using **7** as initial form. The concentration of the resulting solution after the addition of acid or base was not adjusted leading to some dilution and thus a slight decrease of absorption. Sequences 1-5 are explained in the main manuscript and therefore neglected here.

The sequence 6 listed in *Table S19* consists of protonation and subsequent switching of **7** (*0;0*) to **12f** (*1;1-H⁺*). After the addition of 10 μL TFA, conversion into **12c** (*0;0-H⁺*) was achieved, which was further converted into **12f** (*1;1-H⁺*) by irradiation with 340 nm. Back-conversion to the NBD form by addition of **Por** was, however, not possible. Consequently, the subsequent deprotonation using NEt_3 to regain **7** (*0;0*) was impossible. Therefore, not only the original NBD species **7** (*0;0*) but also the protonated analog **12c** (*0;0-H⁺*) can completely convert into the respective QC derivatives (**12** and **12f**) by using just one distinct wavelength (340 nm).

Further, single-site switching was attempted with reversible protonation. Since single phenyl sided QC **12a** is not properly accessible, only the conversion of the amine side to **12b** was further investigated. Therefore, various input sequences were tested (*Table 2, sequences 5,7 and 8*). It was found that **12d** (*0;1-H⁺*) can be obtained via both directions. In sequence 7, first switching with 475 nm to **12b** (*1;1*) and subsequent protonation yielded **12d** (*0;1-H⁺*). The inverse procedure performed in sequence 8 also yielded **12d** (*0;1-H⁺*) with **12c** (*0;0-H⁺*) as intermediate after protonation. In both cases, **7** could be recovered after the addition of base, which indicates the possibility of a base-catalyzed back-conversion as well. For sequence 9, conversion into **12f** (*1;1-H⁺*) was accomplished by irradiation of **12d** (*0;1-H⁺*) with 340 nm. The subsequent addition of base, however, resulted in the formation of an unidentified species rather than recovery of either neutral species **7** or **12**. Last, in sequence 9, irradiation of **7** with daylight for 1 h resulted in the complete conversion to QC **12** (*1;1*). Addition of TFA yielded protonated reconverted NBD **12c** (*0;0-H⁺*) which was stable upon addition of **Por**. Through addition of base, **7** (*0;0*) was recovered.

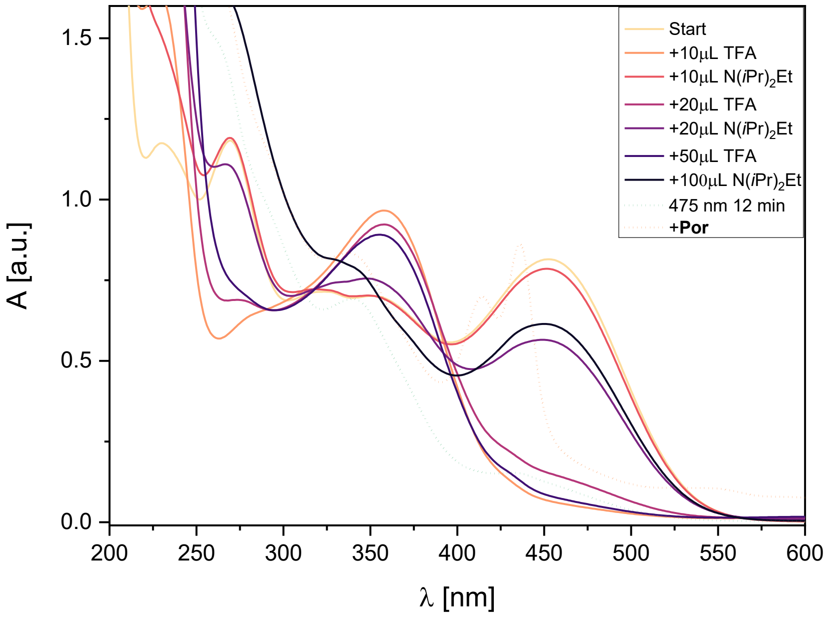

*Figure S 179: UV/Vis switching spectra of **7** measured during the reversible protonation experiments in MeCN. The figure corresponds to table 19, sequence 1. Through alternate addition of acid and base, conversion between **7** and **12c** was cycled. Subsequent irradiation at 475 nm yielded **12b**. Addition of **Por** did not recover **7**.*

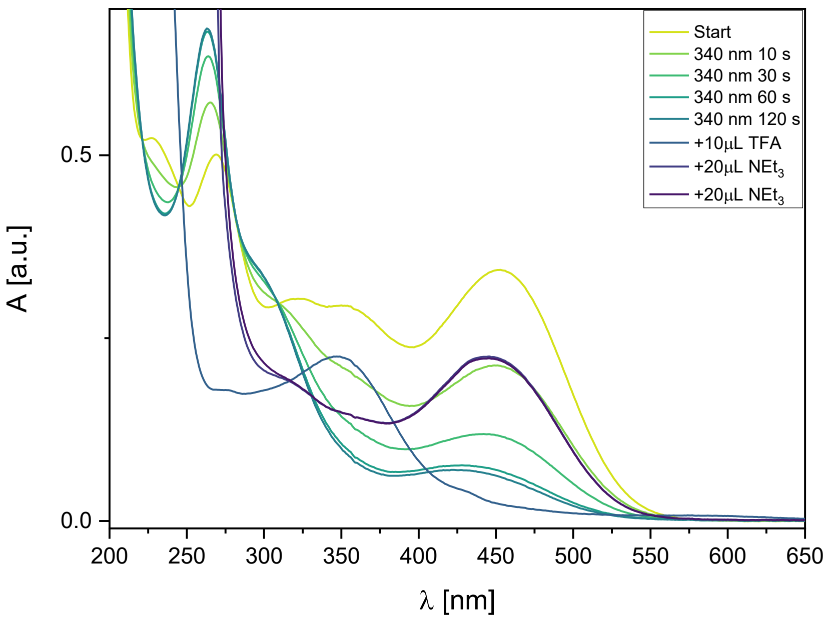

*Figure S 180: UV/Vis switching spectra of **7** measured during the reversible protonation experiments in MeCN. The figure corresponds to table S 19, sequence 2. First irradiation at 340 nm led to formation of **12**, subsequent protonation however yielded the protonated form of the allNBD derivative **12c.** Thus, acid induced back-conversion must be considered Addition of base regained **7**.*

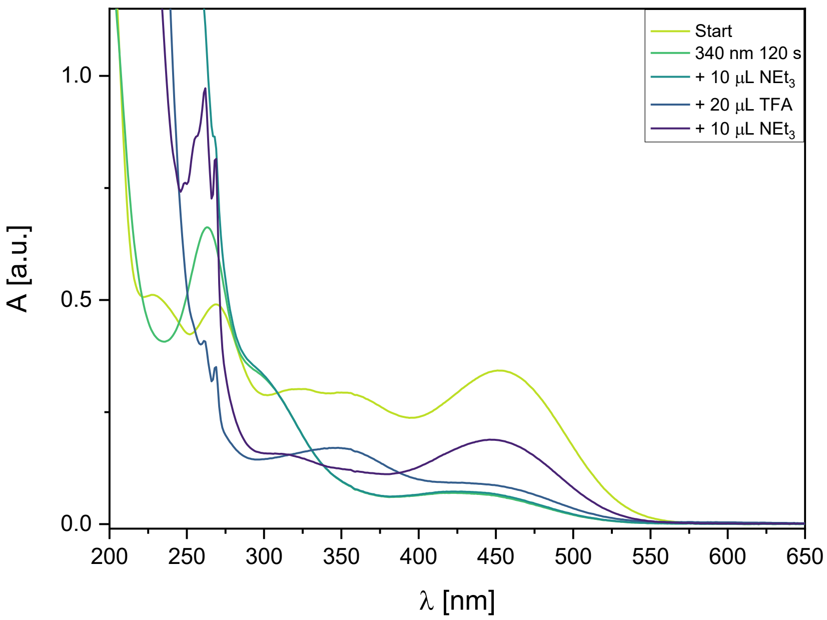

*Figure S 181: UV/Vis switching spectra of **7** measured during the reversible protonation experiments in MeCN. The figure corresponds to table S 19, sequence 3. Here the same observations as for entry 3 (figure S 169) were made.*

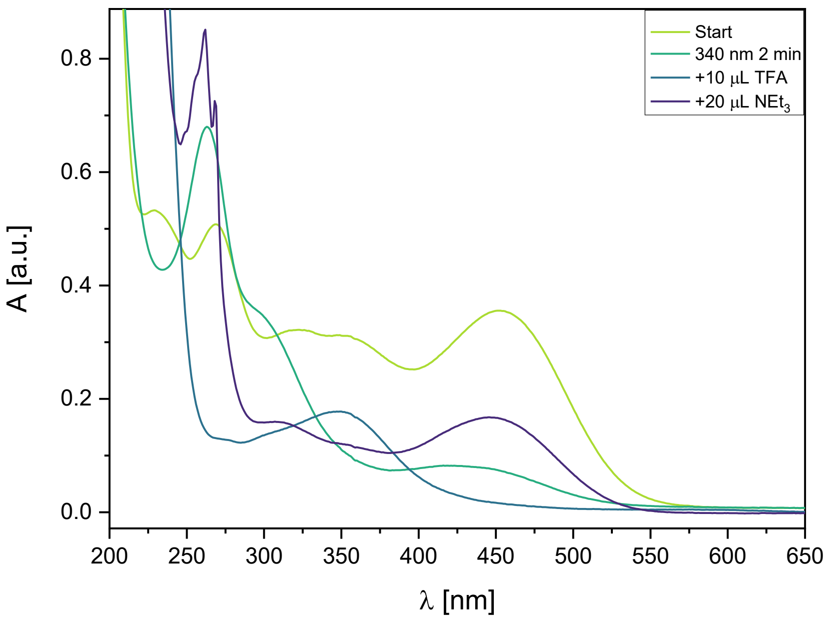

*Figure S 182: UV/Vis switching spectra of **7** measured during the reversible protonation experiments in MeCN. The figure corresponds to table S 19, sequence 4. To exclude potentially occurring back-conversion induced by the heat generated by the acid-base reaction, similar inputs as described in entry 3 were chosen while the sample was cooled to 0 °C. Nevertheless, the very same outcome as before was observed.*

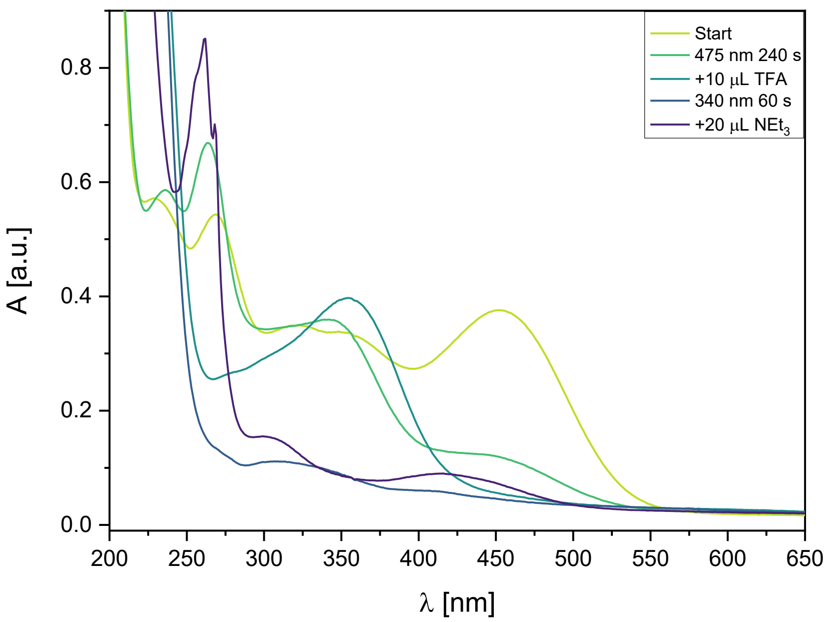

*Figure S 183: UV/Vis switching spectra of **7** measured during the reversible protonation experiments in MeCN. The figure corresponds to table S 19, sequence 5. Here, via different inputs the cycle from **7** over **12b** and **12d** to **12f** is shown. Addition of base yielded unidentified species.*

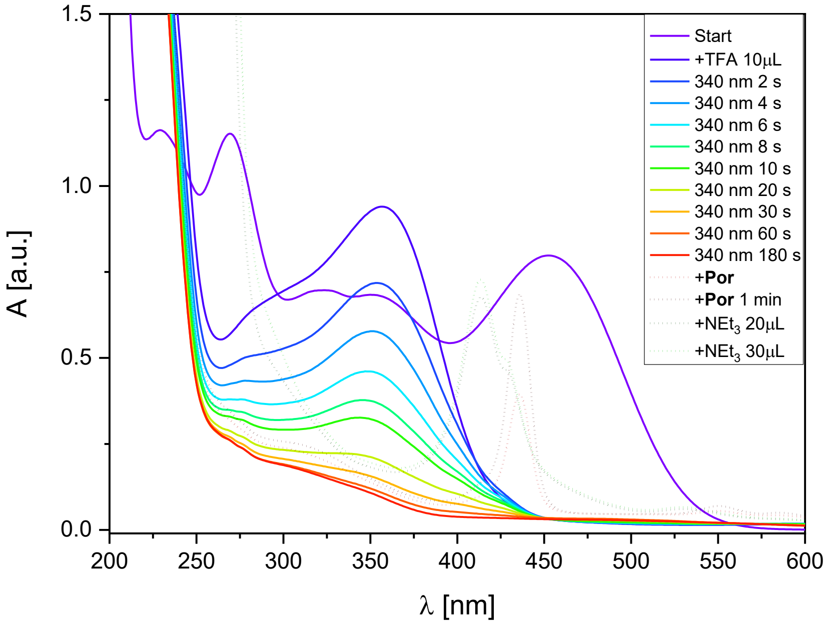

*Figure S 184: UV/Vis switching spectra of **7** measured during the reversible protonation experiments in MeCN. The figure corresponds to table S 19, sequence 6. Irradiation at 340 nm led to the conversion into the respective all-QC derivative. However, upon addition of **Por** and subsequent base (doddet lines), regain of the initial all NBD absorption was not possible.*

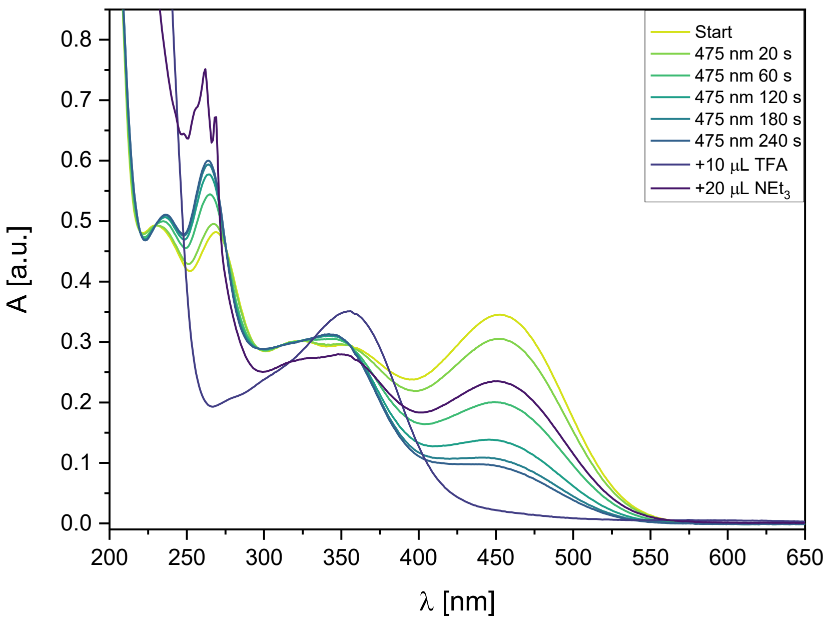

*Figure S 185: UV/Vis switching spectra of **7** measured during the reversible protonation experiments in MeCN. The figure corresponds to table S 19, sequence 7. First, irradiation at 475 nm yielded **12b**, subsequent protonation **12d** and last deprotonation by addition of base back-converted **7**.*

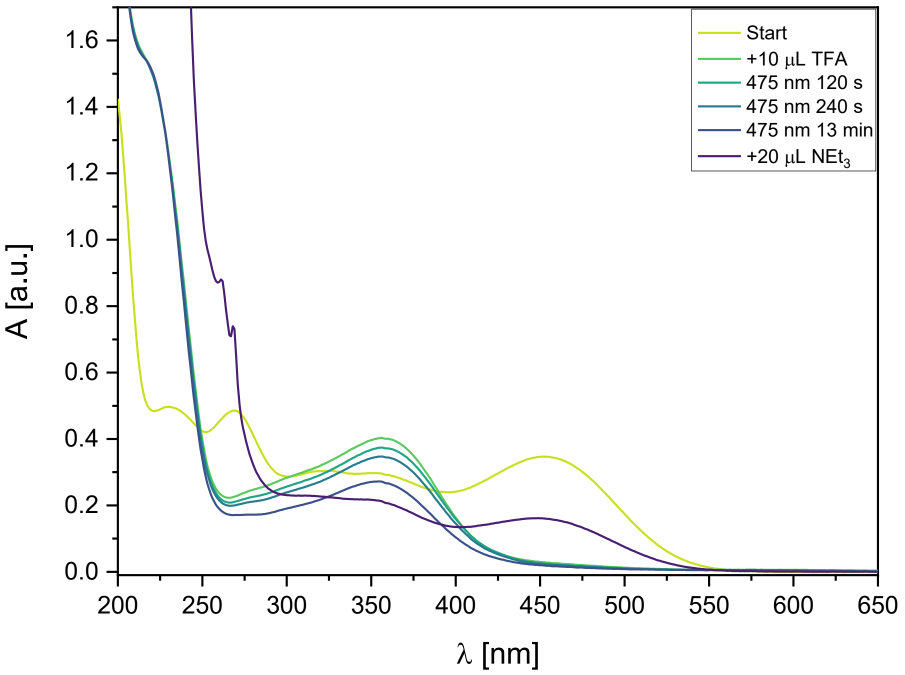

*Figure S 186: UV/Vis switching spectra of **7** measured during the reversible protonation experiments in MeCN. The figure corresponds to table S 19, sequence 8. Vice versa experiment to entry 6. First, protonation by acid yielded **12c** and afterwards irradiation at 475 nm led to **12d** (assigned due to the missing absorption shoulder at 225 nm which is significant for **12c**). Subsequent addition of base probably formed **7** while also the total absorption decreased.*

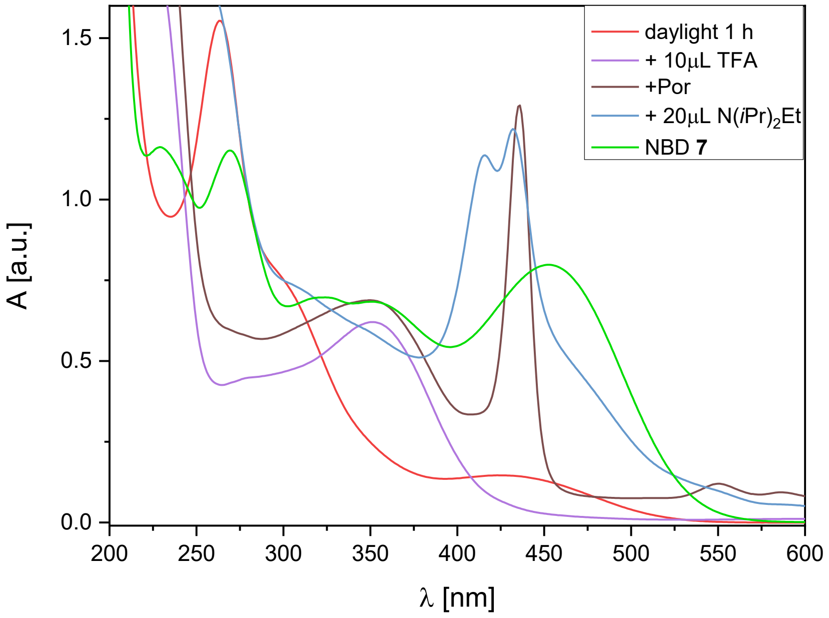

*Figure S 187: UV/Vis switching spectra of **7** measured during the reversible protonation experiments in MeCN. The figure corresponds to table S 19, sequence 9. Daylight irradiation resulted in complete conversion into **12**. Addition of acid then yielded the back-converted **12c**. Then, the addition of **Por** verified the previously induced back-isomerization to **12c** while just*

*shifting the baseline upwards. Finally, addition of base showed indication of the deprotonated species **7** again. The cuvette was placed in front of the window for 1 h on a slightly cloudy day (06.05.2024)*

In the following, an additional series of reversible protonation experiments is provided to investigate the single-side conversion to **12a** and **12e**. The protonation of **7** to **12c** results in a convolution of the absorption maxima, which is assumed to make independent switching barely possible. Therefore, irradiation of protonated **12c** is expected to generate the double-switched **12f** directly. Thus, to reach **12a**, the input order probably matters by first irradiation and subsequent protonation. However, as described in the main manuscript, TFA addition might induce the back-isomerization from QC to NBD, hindering the generation of **12e**. Since validation of these conversion species via NMR spectroscopy is not possible, estimations of the interconversion behavior are given based on the available UV/Vis data and compared to the previously described switching studies.

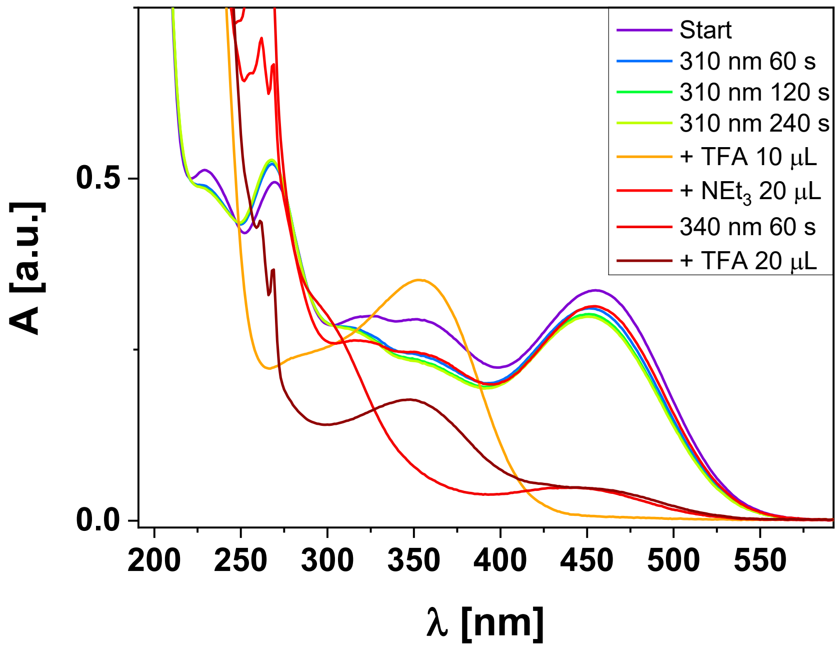

*Figure S 188: UV/Vis switching spectra of **7** measured during the reversible protonation experiments in MeCN. Irradiation at 310 nm yielded partial conversion to **12a** as discussed in the main manuscript. Subsequent addition of TFA resulted in conversion of either a potential mixture of 12c and 12e. Addition of base yielded the same spectrum as before corresponding to 12a, and subsequent irradiation at 340 nm resulted in **12**. Final addition of TFA seems to recover **12c**.*

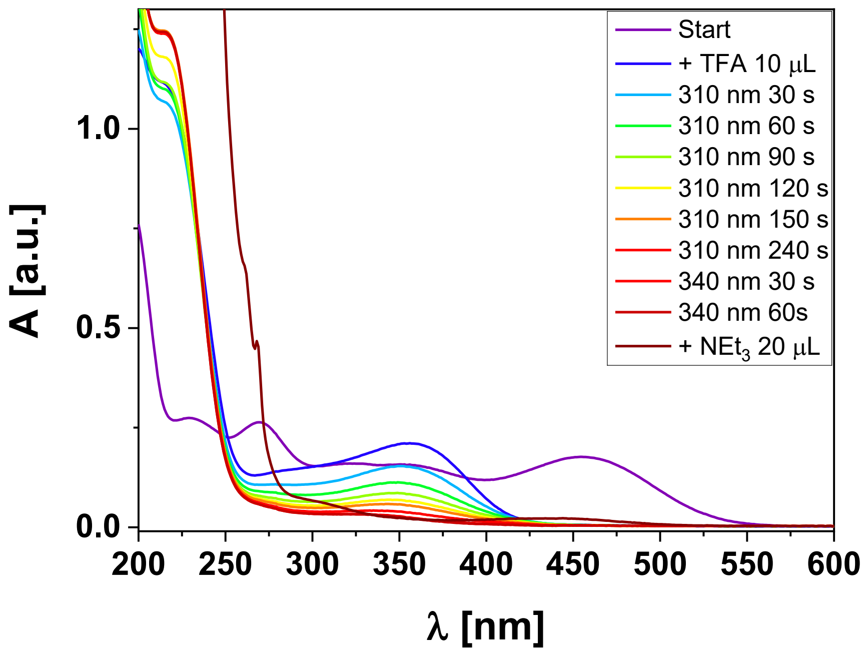

*Figure S 189: UV/Vis switching spectra of **7** measured during the reversible protonation experiments in MeCN. First protonation with TFA yielded **12c**. Irradiation at 310 nm seems to lead to first partial isomerization and subsequently complete conversion to **12f**. This conclusion is mainly derived from the absorption between 200 and 250 nm which first decreases (as given for e.g. **12d**) and subsequently increases to the spectrum of **12f**. The switch to 340 nm did not induce any further changes and the final addition of base seems to yield the spectrum of **12** which is mainly overshadowed by the NEt_3 absorption.*

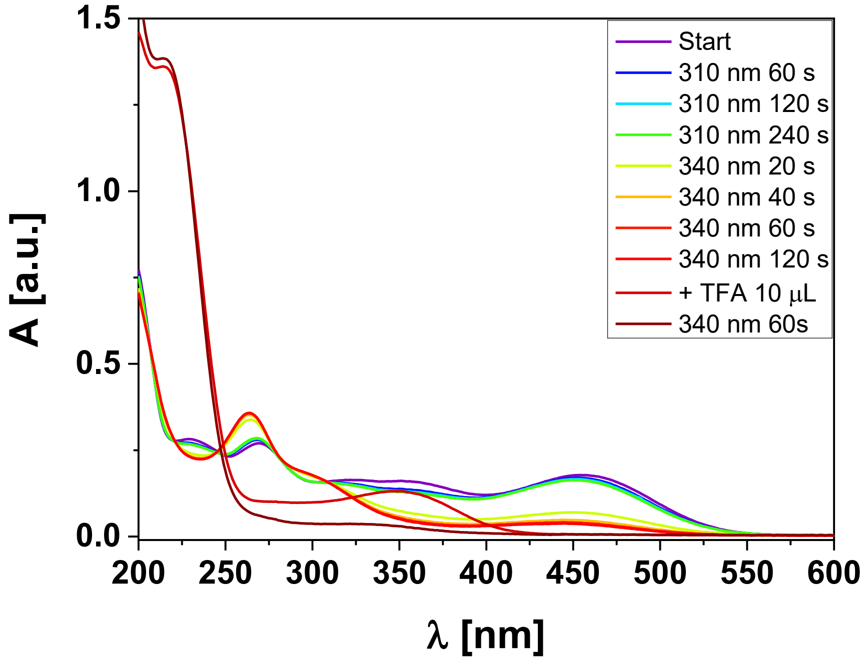

*Figure S 190: UV/Vis switching spectra of **7** measured during the reversible protonation experiments in MeCN. First irradiation at 310 nm yielded partial conversion to **12a**, which was transformed to **12** by application of the 340 nm LED. Subsequent addition of TFA seems to induce the back-conversion to **12c**. Once more usage of the 340 nm LED led to formation of **12f**.*

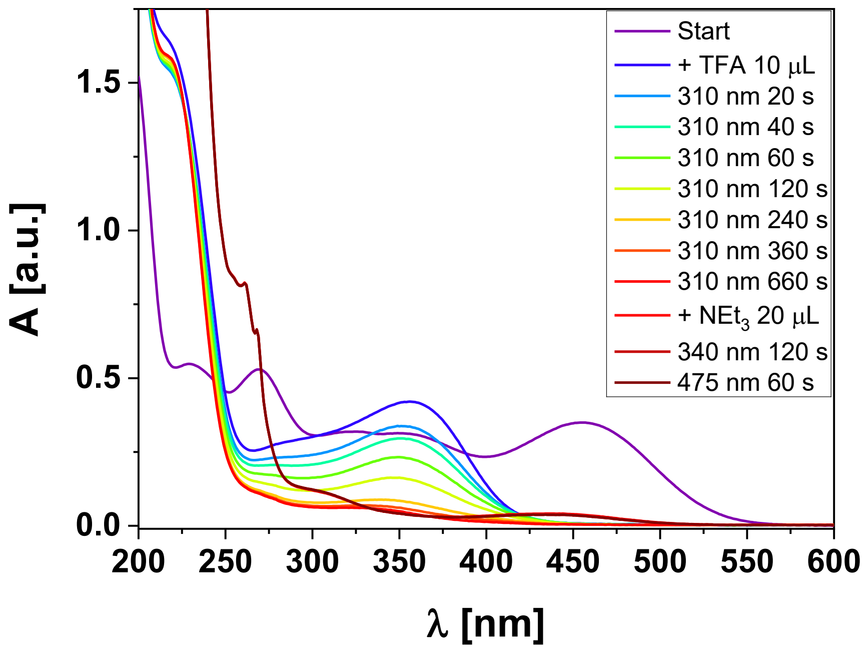

*Figure S 191: UV/Vis switching spectra of **7** measured during the reversible protonation experiments in MeCN. In principle the initial input sequence is similar as described in figure S 178. However complete conversion to **12f** was induced by only using 310 nm underlying the assumption of now convoluted absorption properties for **12c**. Subsequent addition of base resulted in the formation of **12**. Additional irradiation at 340 nm and 475 nm did not induce further changes proving the presence of **12**.*

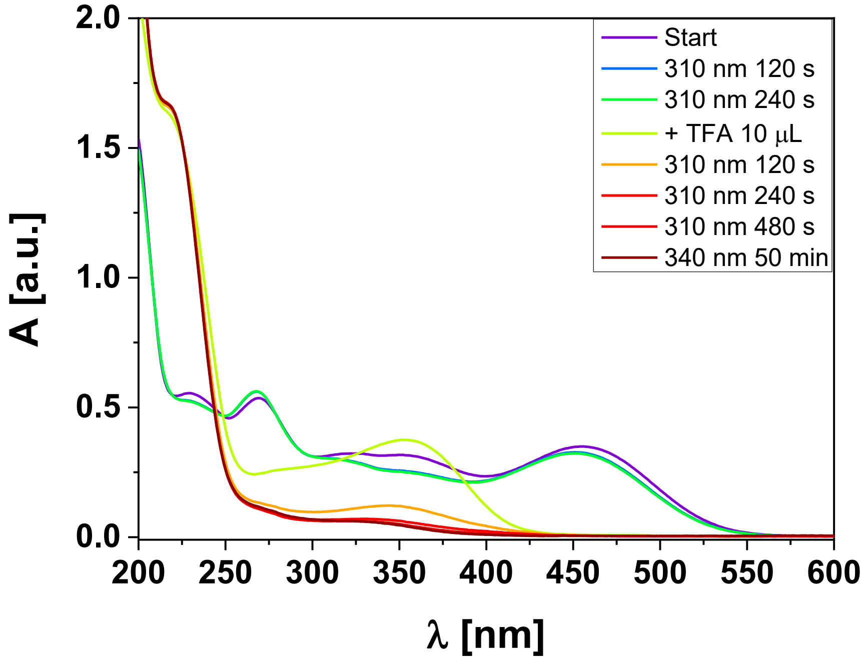

*Figure S 192: UV/Vis switching spectra of **7** measured during the reversible protonation experiments in MeCN. Irradiation at 310 nm induced partial conversion to **12a**, which was converted to either **12c** or **12e** upon addition of TFA. Subsequent irradiation at 310 nm resulted in **12f**. Also, after prolonged irradiation of 50 min, no photodecomposition could be observed.*

5.5 NBD **8** to QC **13**

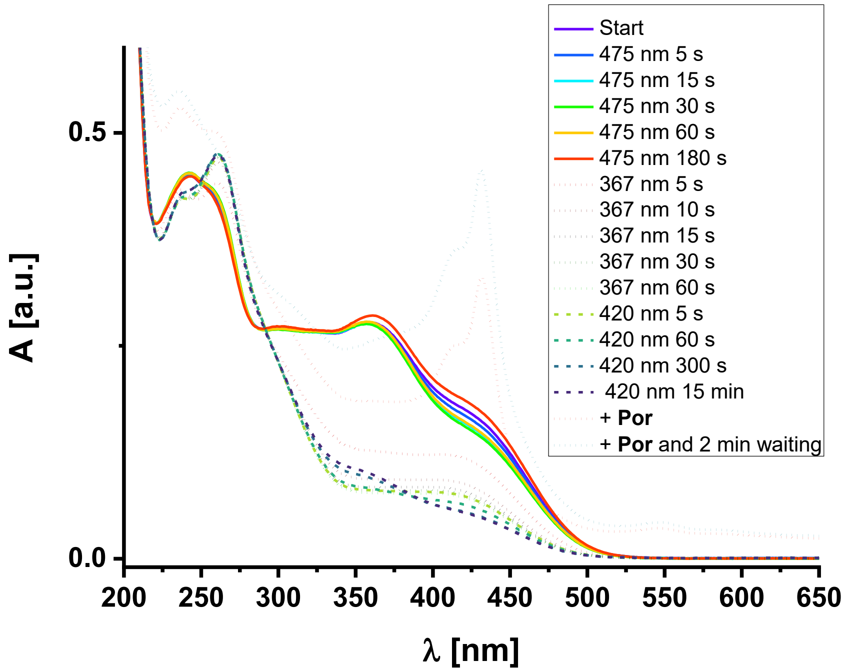

*Figure S 193: UV/Vis switching study of **8** measured in MeCN. First, a 475 nm LED was used to try to trigger the selective isomerization of the amine side. However, only slight changes in the overall absorption were found. Therefore, 367 nm were utilized to induce complete conversion to **13**. Since a leftover absorption plateau was present between 400 and 450 nm, a 420 nm LED was additionally used. A slight decrease in leftover absorption was still found indication some leftover species which were not complete switched at this point. By addition of **Por**, the initial all NBD absorption of **8** could be recovered especially visible at the curve shape in the lower wavelength region (compare solid lines and dotted blue and red lines).*

An interesting feature in *Figure S193* is that an absorption band at 360 nm emerges (again) during the last irradiation period (λ_{irrad} = 420 nm. Based on the quantum chemical calculations, this might be due to the QC-Ph NBD-Ph back switching matching the absorption band of **4**.

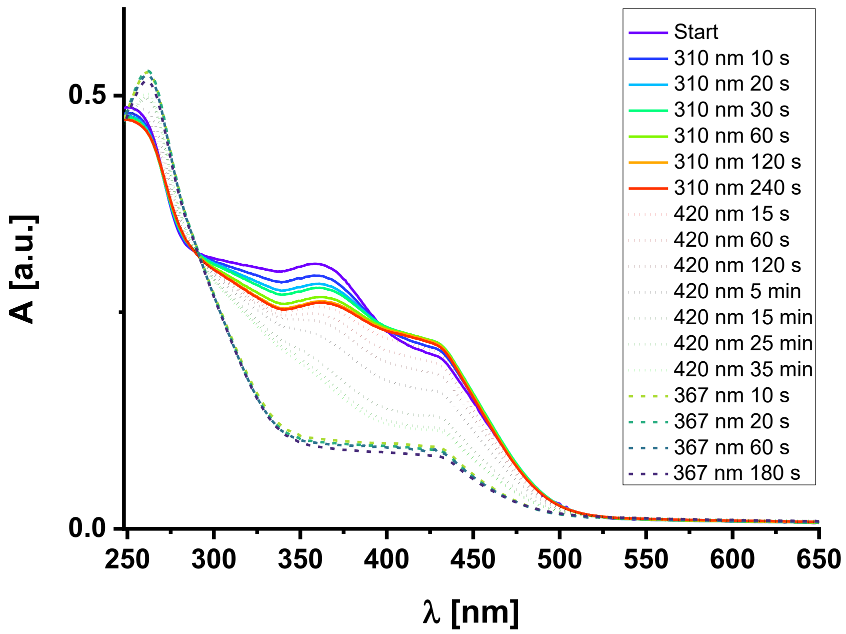

*Figure S 194: UV/Vis switching study of **8** measured in MeCN. First, a 310 nm LED was used to attempt the selective isomerization of either the phenyl or methoxyphenyl substituted side. An indication of a conversion similar as described for the two-fold substituted **7** to **12a** was found. Afterwards, a 420 nm LED was used. Finally, by changing to 367 nm, complete conversion to **13** was observed.*

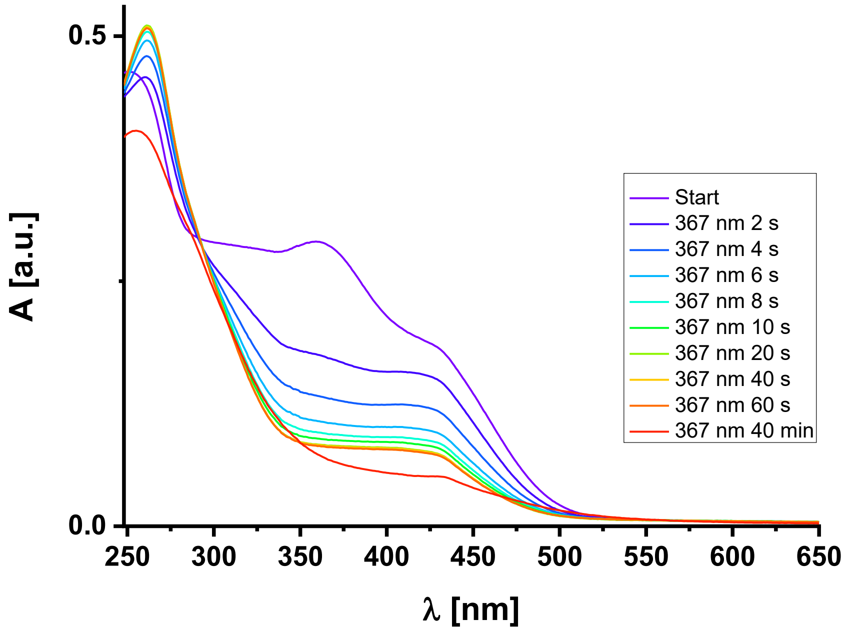

*Figure S 195: UV/Vis switching study of **8** measured in MeCN. Direct irradiation at 367 nm was investigated yielding complete conversion to **13**. Prolonged irradiation time seems to lead to photodecomposition.*

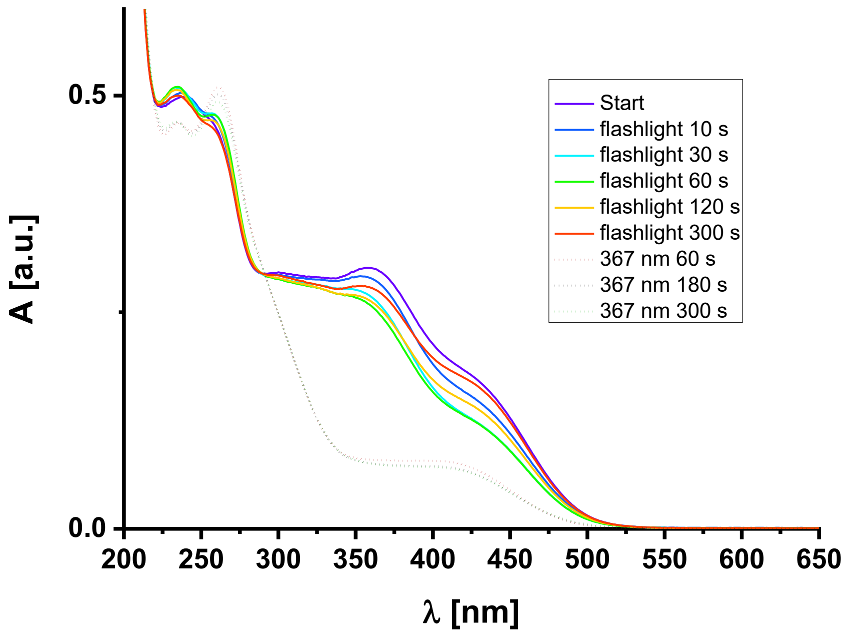

*Figure S 196: UV/Vis switching study of **8** measured in MeCN. As tested for **7**, irradiation using a high-power flashlight was investigated. However, only slight change in absorption was found without a clean conversion pattern. Therefore, the assumption can be made that "white" light triggers every available NBD moiety leading to a combination of different parallel switches. Afterwards with 367 nm complete conversion into **13** was achieved.*

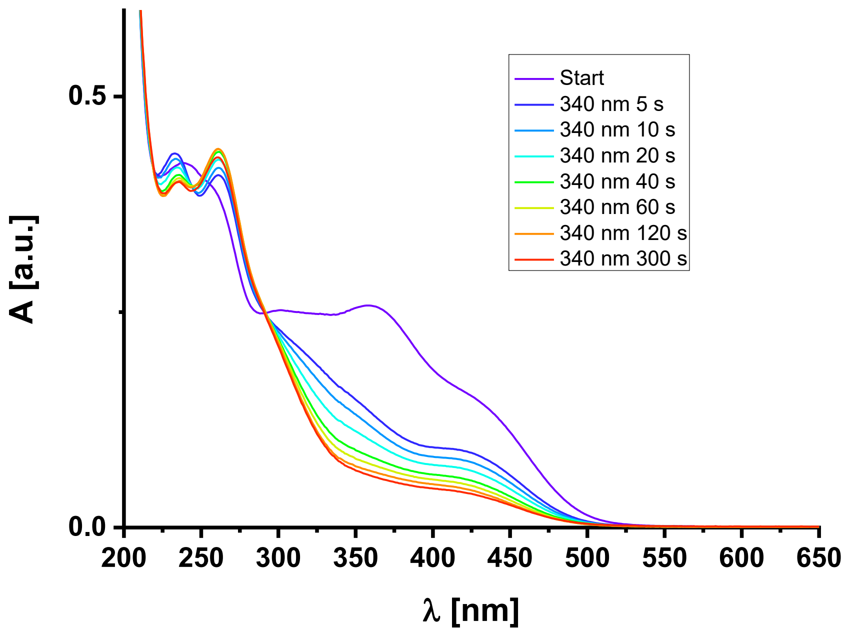

*Figure S 197: UV/Vis switching study of **8** measured in MeCN using a 340 nm LED.*

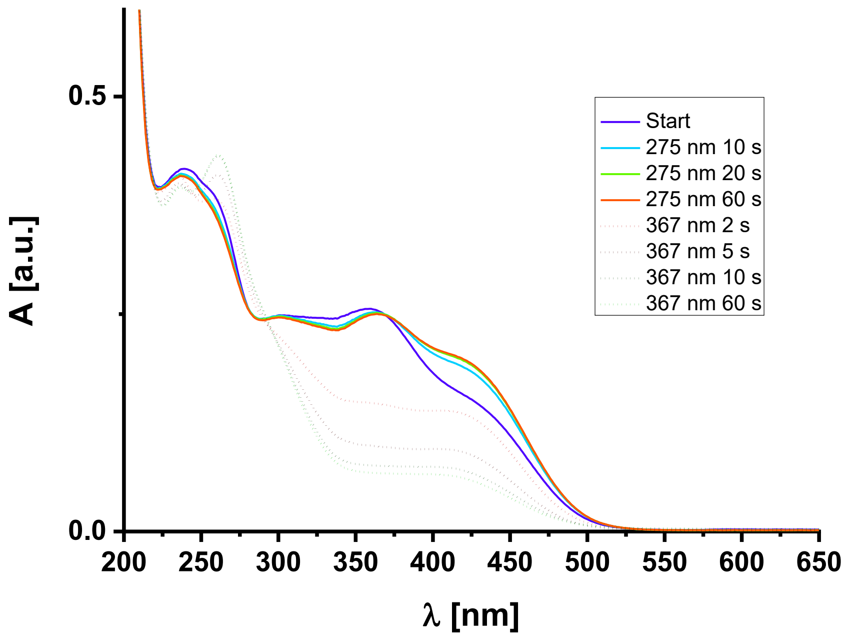

*Figure S 198: UV/Vis switching study of **8** measured in MeCN using a 275 nm LED first initiating partial conversion. Subsequent irradiation at 367 nm resulted in formation of **13**.*

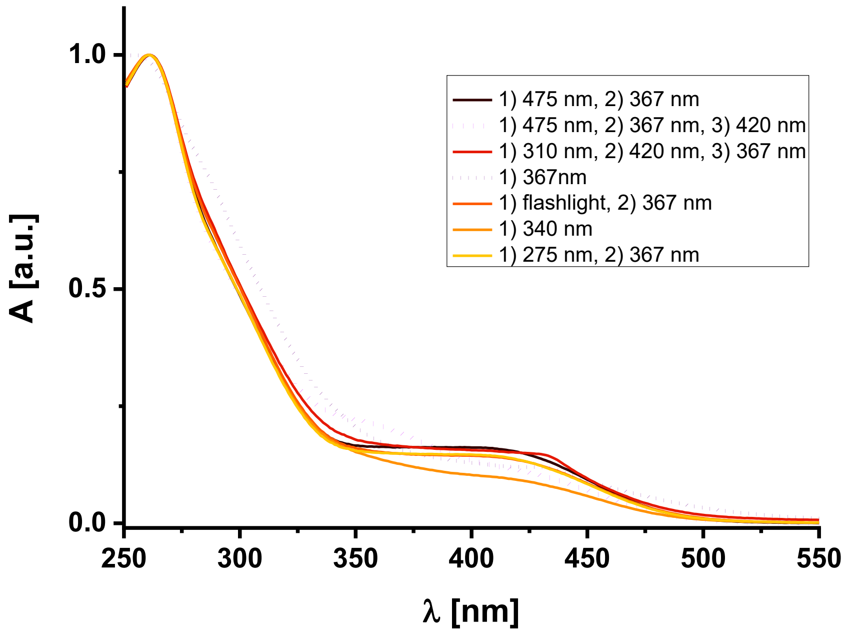

*Figure S 199: Comparison of the normalized UV/Vis end point spectra of **8** to **13** measured in MeCN. Irradiation of 340nm seems to be essential to initiate complete conversion to **13** since with 367, a leftover absorption plateau around 425nm is always observable.*

6 Photoisomerization Quantum Yields (ϕ_{iso})

The photoisomerization quantum yield describing the efficiency of a photoreaction is defined as:

$$\phi_{iso} = \frac{Isomerization\ events}{Absorbed\ photons}$$

Considering an ideal photoswitch, the quantum yield should be close to unity. In recent studies,[13] determination of the quantum yield by classical chemical actinometry in a high absorption regime[8],[14]–[17] has been unsuccessful due to limitations with our available setup (compare Figure S1).

Therefore, an automated reaction setup was used. The photoisomerization quantum yield for **4** to **9,** **5** to **10, 7** to **12**, and **12b**, and **8** to **13**, was determined in chloroform or MeCN solutions using the setup developed by *E. Riedle*.[18] An overview of the obtained results is provided in table S21. 2.0 mL of the respective solution was filled in a cuvette, and the illumination power of the LED P_{ill} was determined prior to the actual measurement. For **4** and **5**, a LED with an emission maximum at 365 nm was used while for **7** and **8** a LED emitting at 340 nm was utilized, in agreement with the best results obtained during the UV/Vis conversion experiments (compare SI *section 5*). For the single-sided conversion of **7** to **12b** a 470nm LED was used. Afterwards, the respective NBD compound was added to the cuvette, and the concentration adjusted to obtain absorption values of approximately 1. The solutions were irradiated at the respective wavelength in defined time intervals until complete conversion to the respective QC was obtained or no further changes in absorption were observed. After each irradiation cycle, one UV/Vis absorption spectrum was recorded, and the power readout at the solar cell detector was recorded. Previously measured extinction coefficients of the pure NBD and QC isomer (and single-sided NBD-QC **12b**) were used to determine the change in concentration and thus the number of isomerized molecules during the experiment. For the mathematical derivation and the formulas necessary for the evaluation of the raw data we would like to refer to the publication of E. *Riedle* and coworkers.[18] For **4**, **5** and **7** to **12b**, clean conversion was observed in the UV/Vis spectra. Nevertheless, proper fitting of the obtained data was not possible in every case, which is why only partially satisfactory results could be obtained using this approach.

Table S 20: Measured quantum yields for the NBD to QC isomerization in $CHCl_3$ or MeCN solutions.

Compound	Solvent	Wavelength	ϕ_{iso}
**4** to **9**	$CHCl_3$	365 nm	76.4%
**5**to **10**	MeCN	365 nm	/
**7 to 12b**	MeCN	470 nm	1.83%
**12b to 12**	MeCN	340 nm	/
**7 to 12**	MeCN	340 nm	/
**8 to 13**	MeCN	340 nm	/

6.1 4 → 9

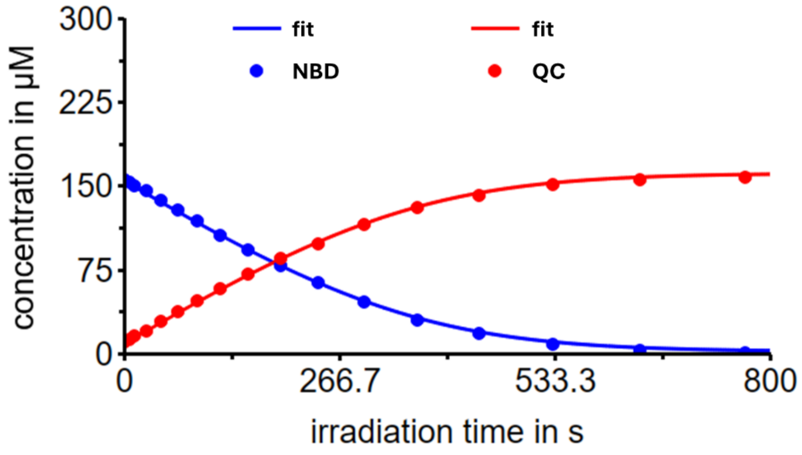

*Figure S 200: Quantum yield determination for the NBD to QC photoisomerization of **4** measured in $CHCl_3$ with 365 nm light source. The graph displays the change in concentration during the experiment and the respective fits.*

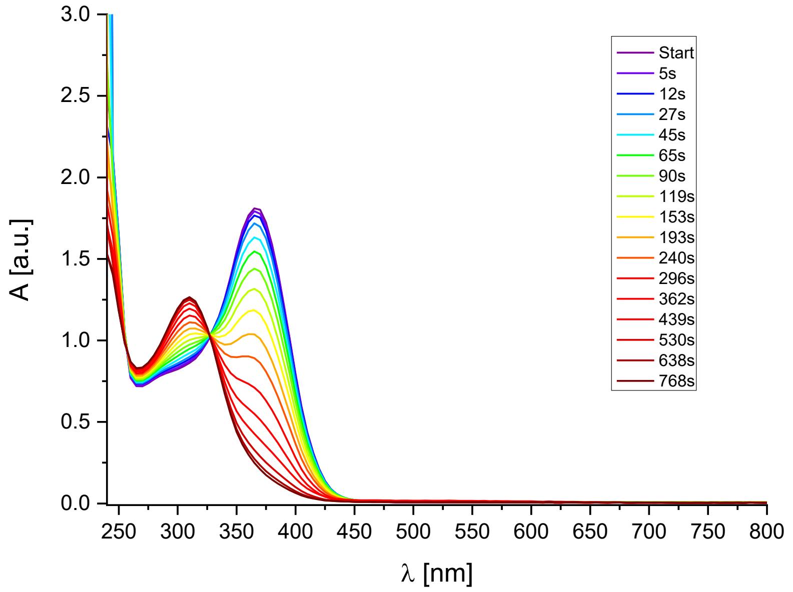

*Figure S 201: UV/Vis absorption spectra (**4** to **9**, measured in $CHCl_3$) during the irradiation of the quantum yield experiment.*

6.2 5 → 10

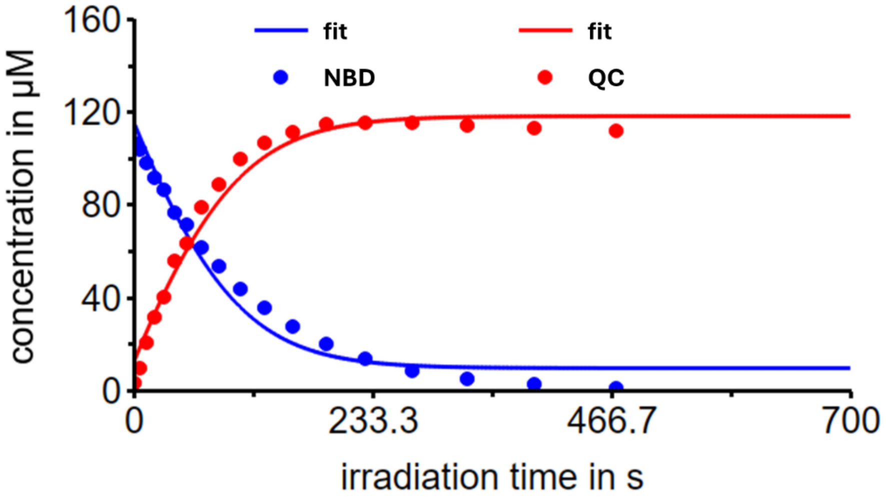

*Figure S 202: Quantum yield determination for the NBD to QC photoisomerization of **5** measured in MeCN with 365 nm light source. The graph displays the change in concentration during the experiment and the respective fits.*

The overall concentration decreases during the illumination process, indicating some degree of photodecomposition. Therefore, combined with the unsatisfactory fit, the calculated quantum yield for the **5** to **10** conversion is estimated to be 112%, which is not a realistic value. A possible explanation relies on the process which is executed by the program to calculate the values. The concentration determination is performed by fitting the respective measurement points based on the extinction spectra of the initial isomers A and B (NBD and QC, respectively). Every intermediate absorption spectrum is attempted to be deconvoluted into the partial spectra of isomer A and isomer B. Due to the lack of isosbestic points, reasoned by the presence of multi-conversion processes the

calculation and resulting fitting is disturbed resulting. This results in negative concentrations which hinders the proper evaluation.

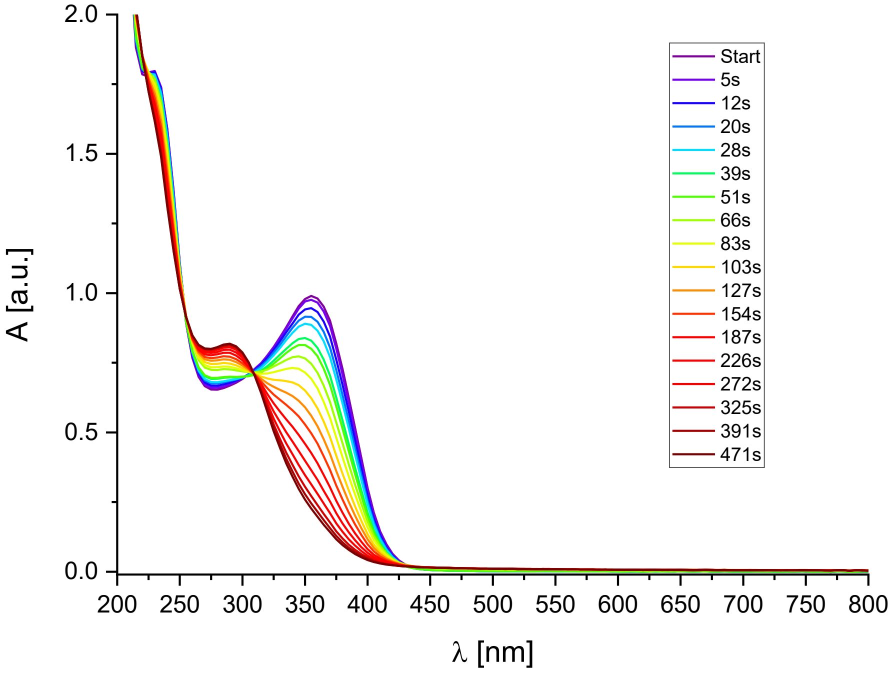

*Figure S 203: UV/Vis absorption spectra (**5** to **10**, measured in MeCN) during the irradiation of the quantum yield experiment.*

6.3 7 → 12b

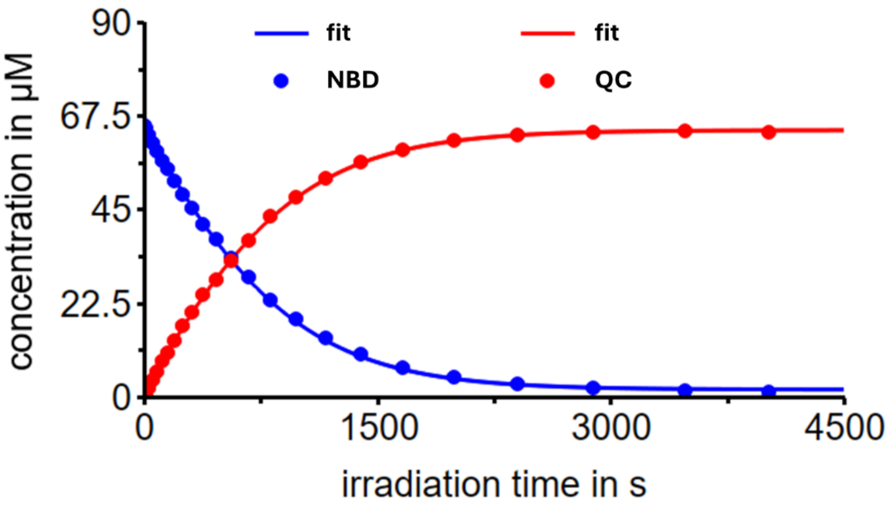

*Figure S 204: Quantum yield determination for the NBD to QC photoisomerization of **7** to **12b** measured in MeCN with 470 nm light source. The graph displays the change in concentration during the experiment and the respective fits.*

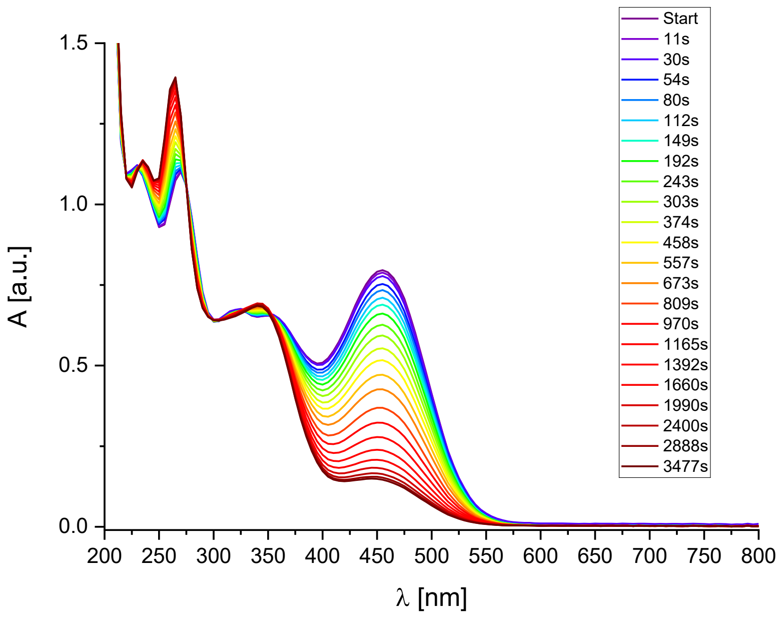

*Figure S 205: UV/Vis absorption spectra (**7** to **12b**, measured in MeCN) during the irradiation of the quantum yield experiment.*

6.4 12b → 12

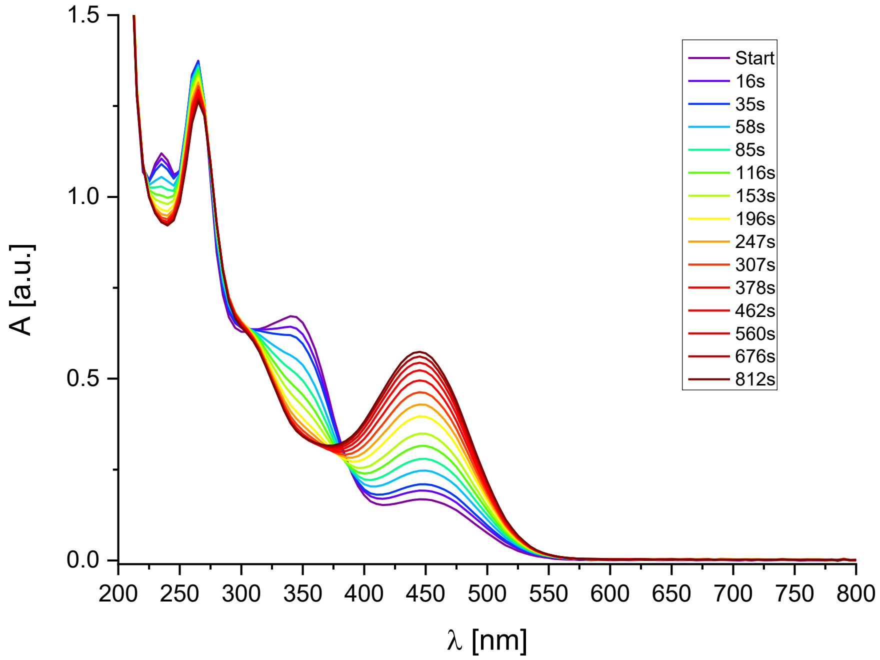

*Figure S 206: UV/Vis absorption spectra (**12b** to **12**, measured in MeCN) during the irradiation of the quantum yield experiment.*

The subsequent irradiation of the sample containing **12b** with light of 340nm, did not yield conversion to **12** as found before (compare main article). The shape of the newly formed absorption

curve, combined with the lack of isosbestic points suggest conversion to an unknown intermediate species which was not found before and not further investigated.

6.5 7 → 12

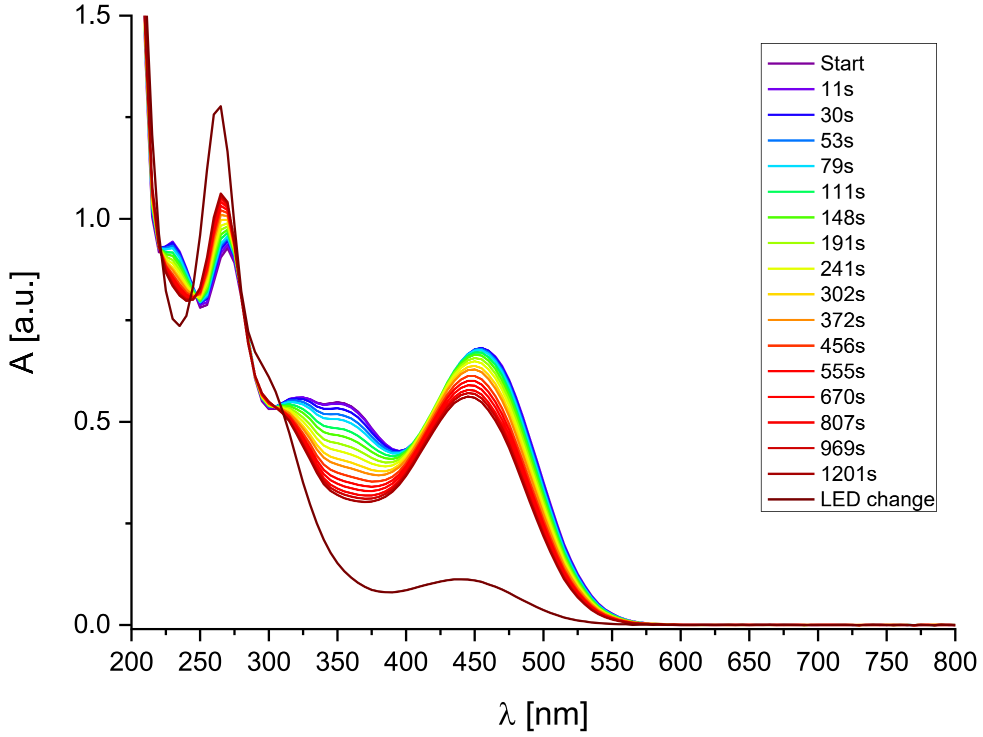

*Figure S 207: UV/Vis absorption spectra (**7** to **12**, measured in MeCN) during the irradiation of the quantum yield experiment.*

After 1201 seconds, no further changes were observed. To check whether complete conversion of this sample to 12 was possible and not somehow hindered, for the last curve, the irradiation was not performed in the quantum yield setup (LED power 0.674 mW) but in the stationary setup (max. LED power 55 mW, compare Figure S1a and Table S2). Here, illumination at 340nm for 2 minutes yielded nearly quantitative conversion to **12**. The utilized LEDs in the stationary setup provide more than 100 times the power of the LEDs investigated in the quantum yield setup. This suggests that for the complete conversion of the investigated species (**7** and **8**), higher power is required, potentially initiating other underlying conversion processes which were not determined.

Due to the incomplete isomerization, fitting the respective concentration and deconvolution of the individual spectra was impossible. This results in the calculation of negative concentrations which hinders the determination of ϕ_{iso} values (compare below).

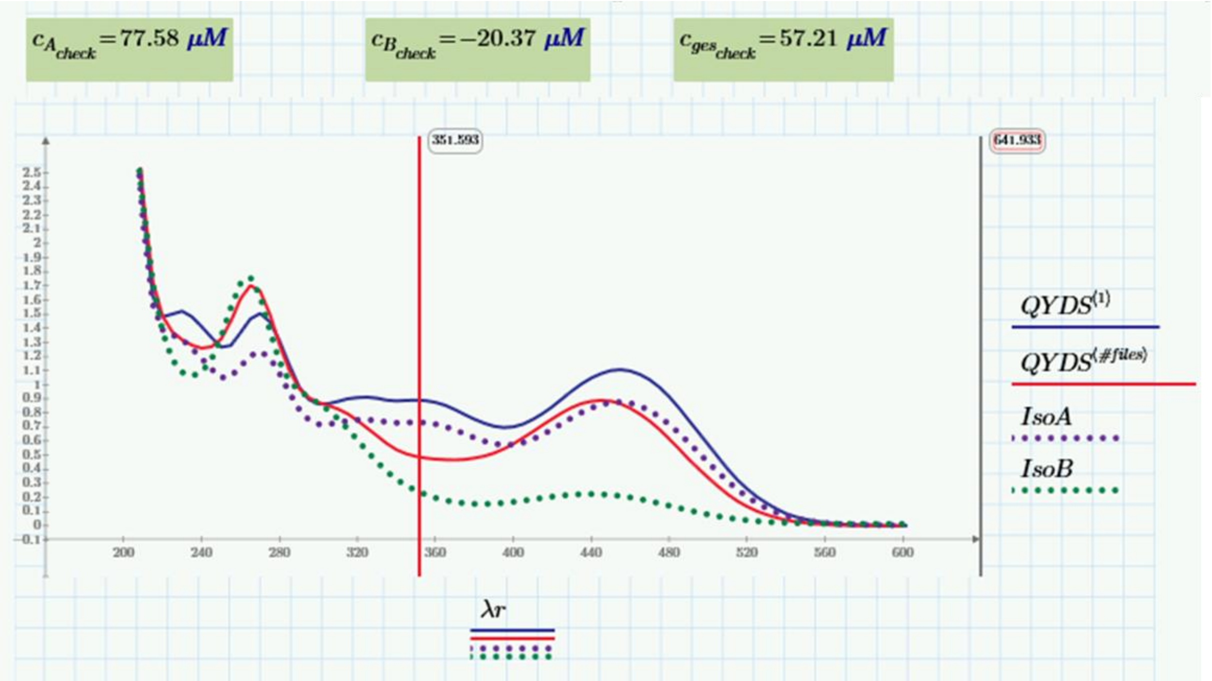

*Figure S 208: Depiction of the automized concentration determination. IsoA belongs to the clean isomer **7** while IsoB is the absorption of clean **12**. The blue and the red curve resemble the start and end spectra of the isomerization experiment, respectively. With these input data, the calculated concentration of QC has a negative value.*

6.6 8 → 13

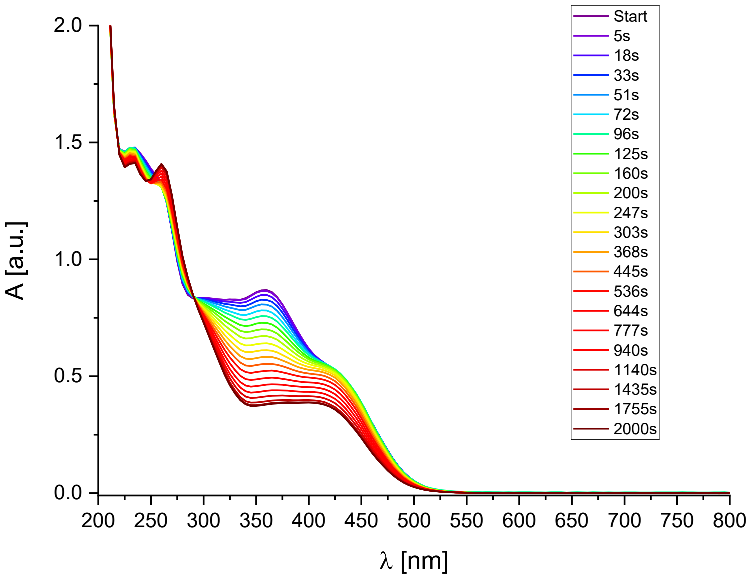

*Figure S 209: UV/Vis absorption spectra (**8** to **13**, measured in MeCN) during the irradiation of the quantum yield experiment. After 2000s the spectral change was neglectable without reaction complete conversion to **13**.*

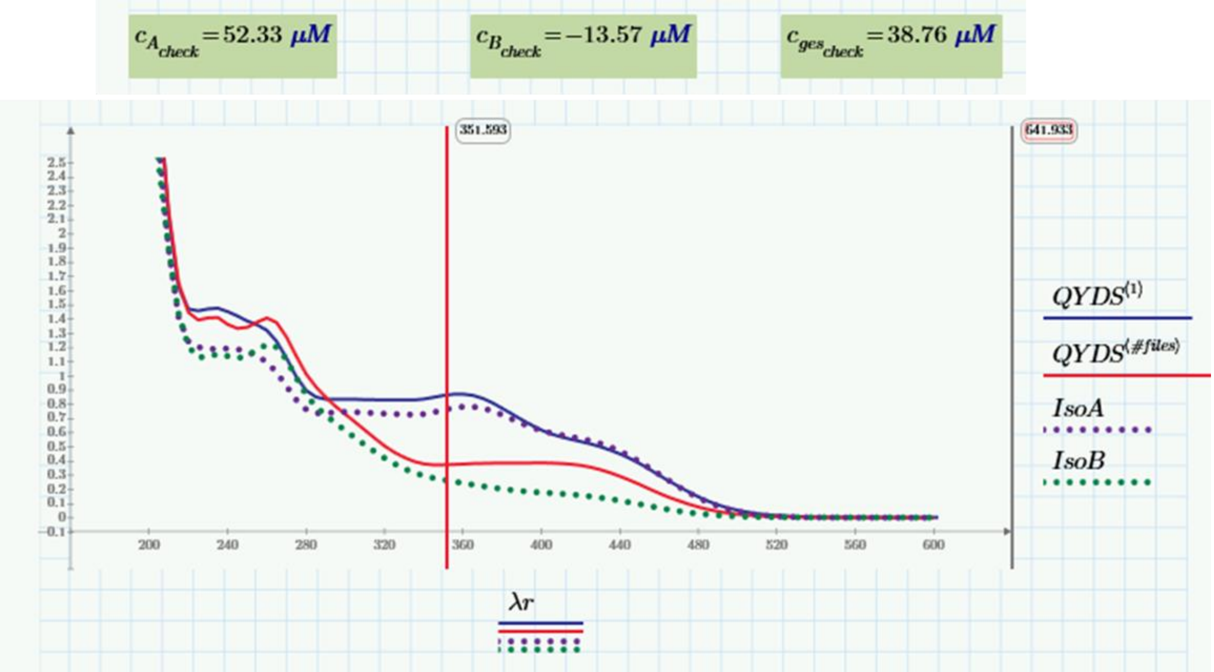

Figure S 210: Depiction of the automized concentration determination. IsoA belongs to the clean isomer ***8*** *while IsoB is the absorption of clean* ***13****. The blue and the red curve resemble the start and end spectra of the isomerization experiment, respectively. With these input data, the calculated concentration of QC has a negative value.*

7 Thermal half-lives ($t_{1/2}$) and cyclability of the QC derivatives

Determination of the Half-lives:

To determine the half-lives $t_{1/2}$ of the metastable QC isomers **9**, **10**, **12**, **12b**, and **13** thermal-induced back-conversion was monitored via ^{1}H NMR spectroscopy. Therefore, 2-4 mg of each NBD sample (**4**, **5**, **7** and **8**) was dissolved in 500 µL $C_2D_2Cl_4$, transferred into an NMR tube and irradiated in the tube for a certain period at the respective wavelength (given in the individual subsections). Each sample was measured at three different temperatures. $\Delta H^{\ddagger}_{thermal}$, $\Delta S^{\ddagger}_{thermal}$ and $t_{1/2}$ were determined using the EYRING plot. Extrapolation of the linear fit led to the evaluation of the values at 25 °C. For unimolecular processes, for evaluation of the thermodynamic properties as $t_{1/2}$ first order kinetics are commonly applied.[19] For multi-NBD derivatives, first-order exponential fits provide a relatively large error, making the linear fit of the EYRING plot imprecise. For these molecules, pseudo first-order or higher order kinetic behavior must be assumed. However, as for instance for the back-conversion of **10** to **5**, the overall process was considered for evaluation, neglecting the formation of occurring intermediates (for details see below).[20] Therefore, the single conversion processes were investigated (**7** to **12b**) which can be assumed to behave like first order kinetics. For first order kinetic fitting of the obtained data to determine the rate constant k, the following question was used:

$$f(x) = y0 + A0 * \exp(-k * x)$$

In an ideal scenario (following first-order kinetic behavior), the back-conversion should be complete and therefore y0 = 0. However, to correct integration errors reasoned by the signal-to-noise ratio in the NMR spectra, y0 was added to the first-order kinetic formula to obtain better fits. Since k mainly depends on the slope of the initial measurement points (left segment of the graph), the fitting was significantly enhanced by including y0 rather than neglecting the factor by setting y0 = 0.

Cyclization experiments:

For cyclization experiments, a fresh sample was prepared in $C_2D_2Cl_4$ and repeatedly irradiated at a specific wavelength and subsequently heated to 120-130°C for 10 minutes. At the beginning and after each step, ^{1}H NMR spectrum was recorded to analyze the conversion process. For the evaluation, in each spectrum, integration of a decomposition signal (main decomposition species) vs. NBD signal vs. QC signal was performed to calculate the overall composition ratio. During this process, disturbance of the overall baseline was found, resulting in a deterioration of the signal-to-noise ratio over the experiment. Therefore, this experiment was conducted to get an overall

indication of the degree of decomposition while precise quantification was not possible using the applied methodology.

Table S 21: Overview of the obtained kinetic data. The cyclability column is a vague attempt to describe the findings, since precise quantification is not possible.

**Entry**	$t_{1/2}$ **(25 °C) [min]**	**Cyclability** (increasing decomposition with increasing complexity)
**9 to 4**	158	Minimal decomposition
**10 to 5**	3912 days[a]	Minor decomposition
**12b to 7**	301	Significant decomposition
**12 to 12b**	/	/
**12 to 7**	/	Massive decomposition
**13 to 8**	/	Complete decomposition

7.1 9 → 4

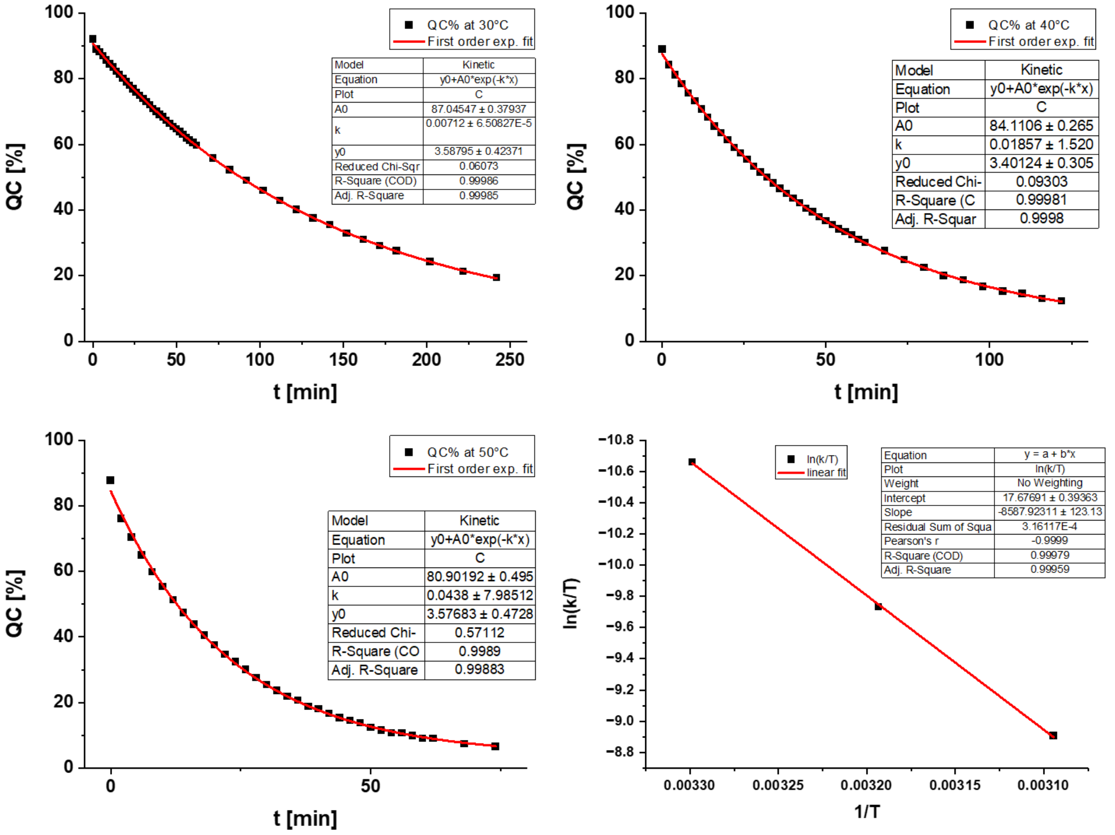

*Figure S 211: Kinetic study of the thermally induced back-conversion of **9** to **4** measured in $C_2D_2Cl_4$. Assuming first order kinetics, exponential fits were applied to determine the rate constants (k) at different temperatures (30, 40 and 50 °C). Bottom right: Linear fit of the Eyring plot of the obtained data of **9** to **4**.*

*Table S 22: Estimated k values for the **9** to **4** conversion obtained from the first order exponential fits.*

**T [K]**	303.15	313.15	323.15
**k [s^{-1}]**	0.00712	0.01857	0.0438

From the data obtained from the Eyring plot $\Delta H^{\ddagger}_{thermal}$ of **9** was estimated to be 17.07 kcal/mol. $\Delta S^{\ddagger}_{thermal}$ was calculated as -12.12 cal*mol^{-1}*K^{-1}. From the extrapolation of the data a thermal half-life at 25 °C of 158 minutes in tetrachlorethane-d_2 was determined for **9**.

$t_{1/2}$ (**9** to **4** at rt) = 158 minutes.

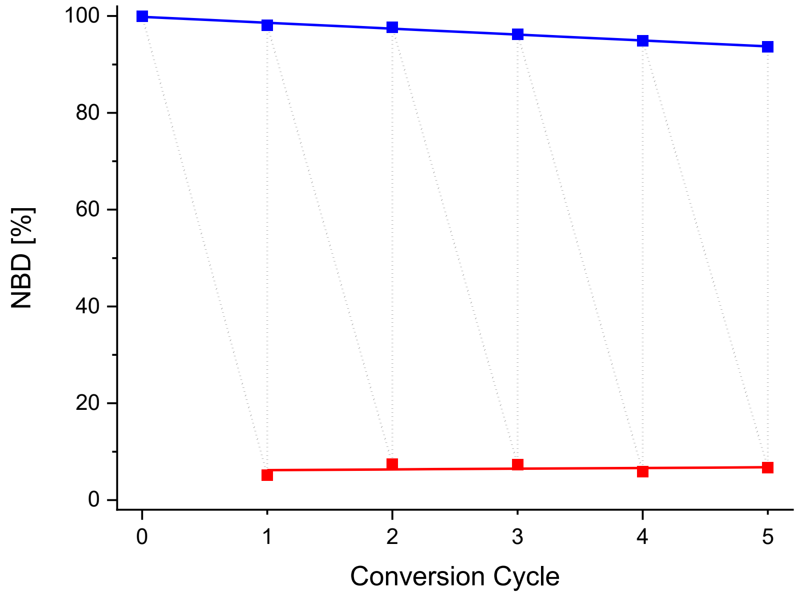

*Figure S 212: Repeated cyclication experiment for the conversion of **4** to **9** and vice versa. A sample was prepared in $C_2D_2Cl_4$ and repeatadly irradiated at 365 nm (50%, 15°C) for 10 minutes subsequently heated to 120°C for 10 minutes. After each step, ^{1}H NMR spectrum was recorded to analyze the conversion process. Minor decomposition during each cycle was observed.*

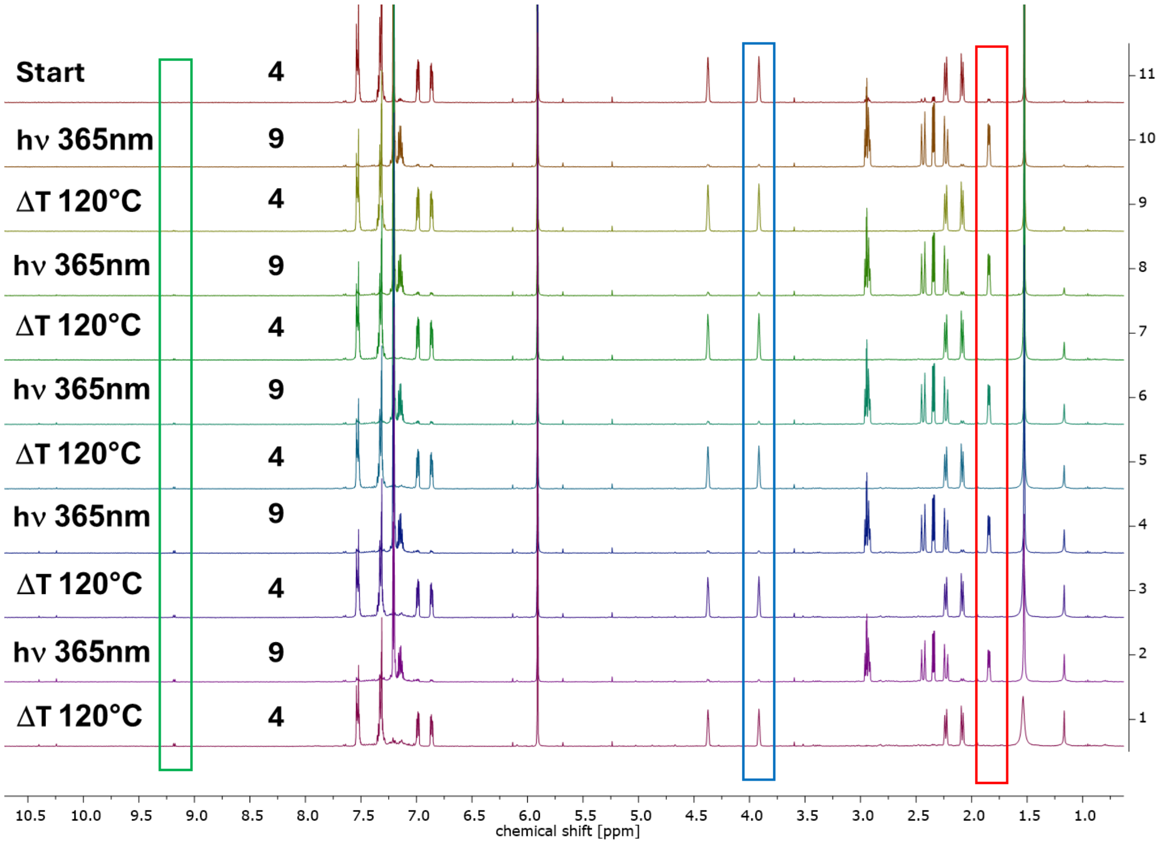

*Figure S 213: Corresponding ^{1}H NMR spectra of the cyclization experiment of **4** to **9** and vice versa. The respective signals which were used for integration are highlighted in green (decomposition species), blue (NBD, **4**) and red (QC, **9**).*

7.2 10 → 5

Since the back-conversion of **10** to **5** is no longer an unimolecular process, the determined data must be seen as an approximation rather than distinct values. As mentioned above, for the evaluation of the data, only the signals corresponding to the QC-QC species and the NBD-NBD species were considered. Intermediate occurring NBD-QC species initially formed upon the heating process and ultimately vanished again, were neglected (signal at 1.85-1.81 ppm). Therefore, to determine the QC ratio, the signal at 4.24-4.18 ppm (NBD-NBD, **5**) and the signal at 1.79-1.75 ppm (QC-QC, **10**) were integrated. However, after a certain period of time, complete reconversion of **10** to **5** was accomplished in every experiment.

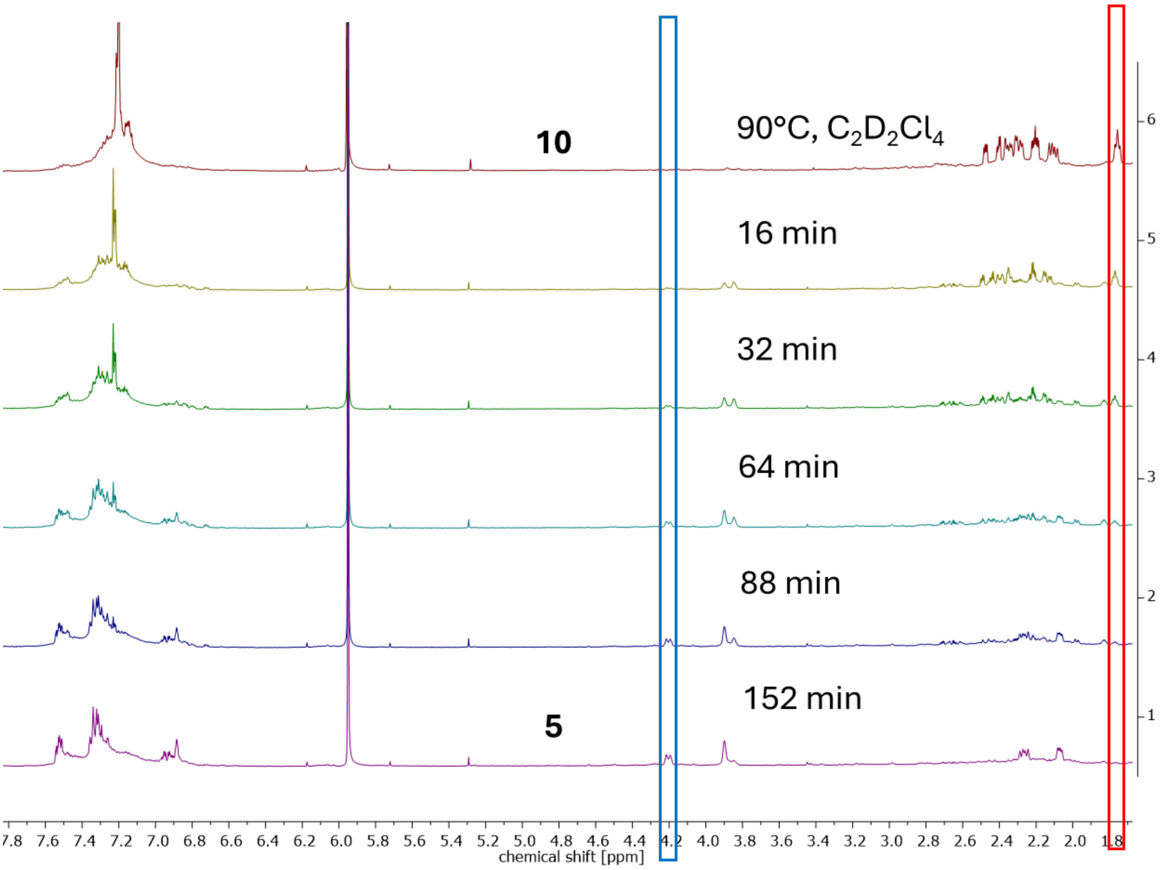

*Figure S 214: Exemplary ^{1}H NMR spectra for the conversion study at 90°C (400 MHz) showing the evolution of the NBD, QC and intermediate species signals. Every spectrum was recorded directly at the used temperature. The respective signals which were used for integration are highlighted in blue (NBD, **5**) and red (QC, **10**).*

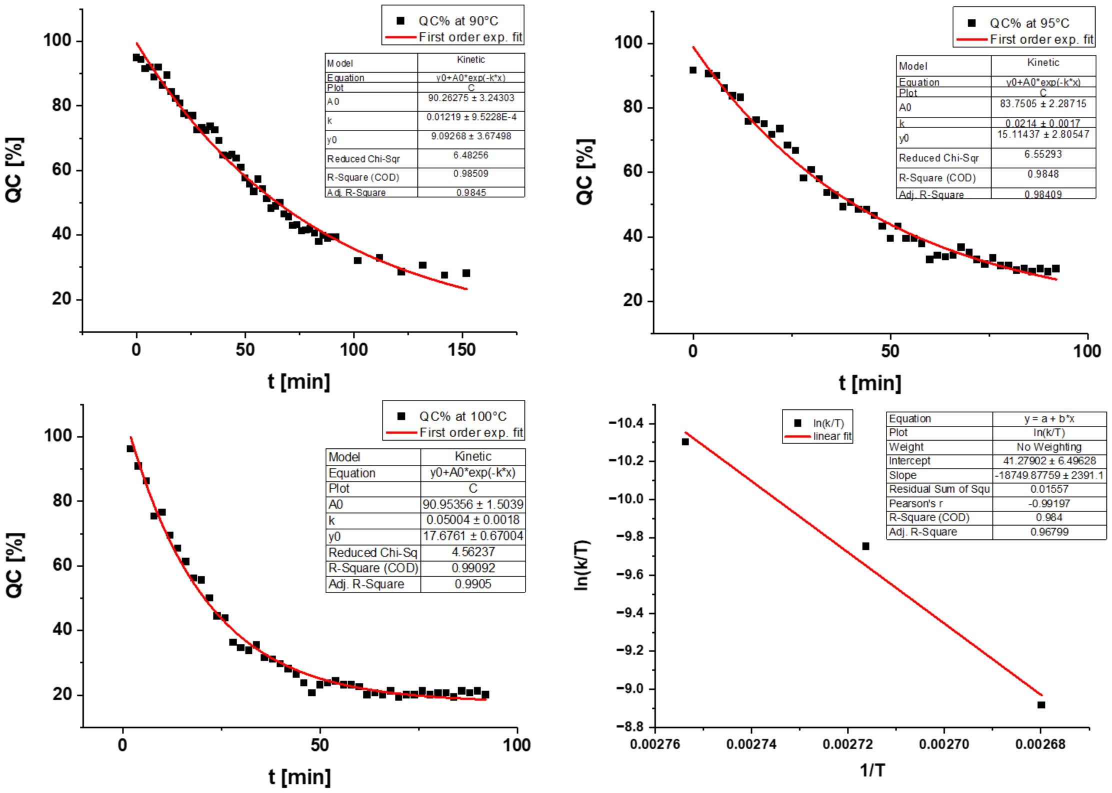

*Figure S 215: Kinetic study of the thermally induced back-conversion of **10** to **5** measured in $C_2D_2Cl_4$. Assuming first order kinetics, exponential fits were applied to obtain the rate constants (k) values at four different temperatures (90, 95, 100 °C). Bottom right: Linear fit of the Eyring plot of the obtained data of **10** to **5**.*

*Table S 23: Estimated k values for the **10** to **5** conversion obtained from the first order exponential fits.*

**T [K]**	363.15	383.15	373.15
**k [s^{-1}]**	0.01219	0.0214	0.05004

From the data obtained from the Eyring plot $\Delta H^{‡}_{thermal}$ of **10** was estimated to be 37.26 kcal/mol. $\Delta S^{‡}_{thermal}$ was calculated as 34.91 cal*mol^{-1}*K^{-1}. From the extrapolation of the data a thermal half-life at 25 °C of 3912 days (10.72 years) in tetrachlorethane-d_2 was determined for **10**.

$t_{1/2}$ (**10** to **5** at rt) = 3912 days.

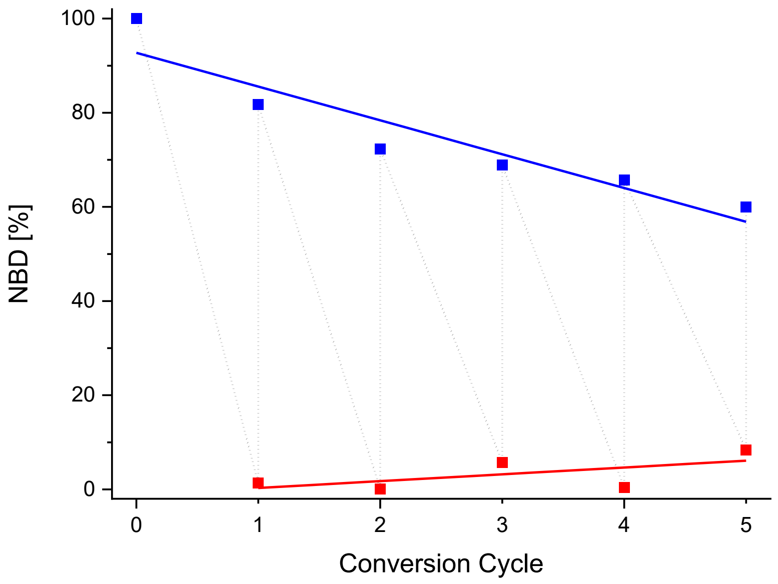

*Figure S 216: Repeated cyclization experiment for the conversion of **5** to **10** and vice versa. A sample was prepared in $C_2D_2Cl_4$ and repeatedly irradiated at 365 nm (50%, 15°C) for 10 minutes subsequently heated to 130°C for 10 minutes. After each step, ^{1}H NMR spectrum was recorded to analyze the conversion process. Therefore, thermal back-conversion of **10** is possible while occurring decomposition is not negligible anymore.*

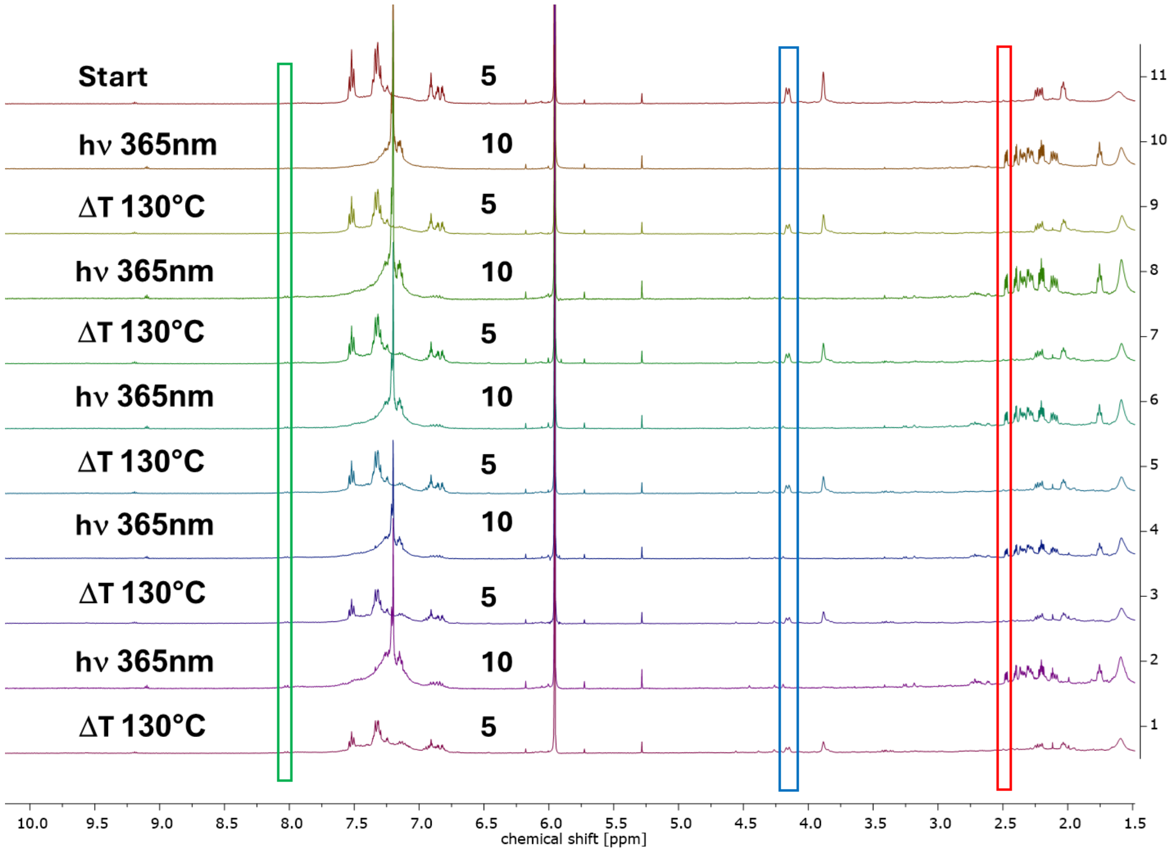

*Figure S 217: Corresponding ^{1}H NMR spectra of the cyclization experiment of **4** to **9** and vice versa. The respective signals which were used for integration are highlighted in green (decomposition species), blue (NBD, **4**) and red (QC, **9**). However, emergence of multiple different additional signals (especially between 4.6 and 2.5 ppm) can be observed disturbing proper integration.*

7.3 12 → 7

During the cyclization experiments, massive decomposition of approximately 30% after 5 cycles at 130°C was observed. Therefore, for the kinetic studies, significantly lower temperatures were chosen to prevent potential side reactions and thermally induced decomposition. However, at 60°C and 70°C, formation of an unidentified species was observed rather than the stepwise regeneration of **7**. When no further changes in the NMR were observed, the temperature was increased to 100°C to initiate further conversion which was found to be at least possible at 130°C (cyclization experiments). In both cases, partial regeneration of **7** was possible, while mainly another unidentified species was formed, accompanied by photodecomposition (see below). Therefore, the back-isomerization of **12** (QC-QC) to **7** (NBD-NBD) no $t_{1/2}$ value could be determined. Combining the findings for the conversion of **10** to **5**, with the complexity of the system **7**, considering multiple different rearrangement processes, further investigations were neglected. Furthermore, clean recovery of **7** from **12** was proven to be possible by addition of **Por** (compare main article).

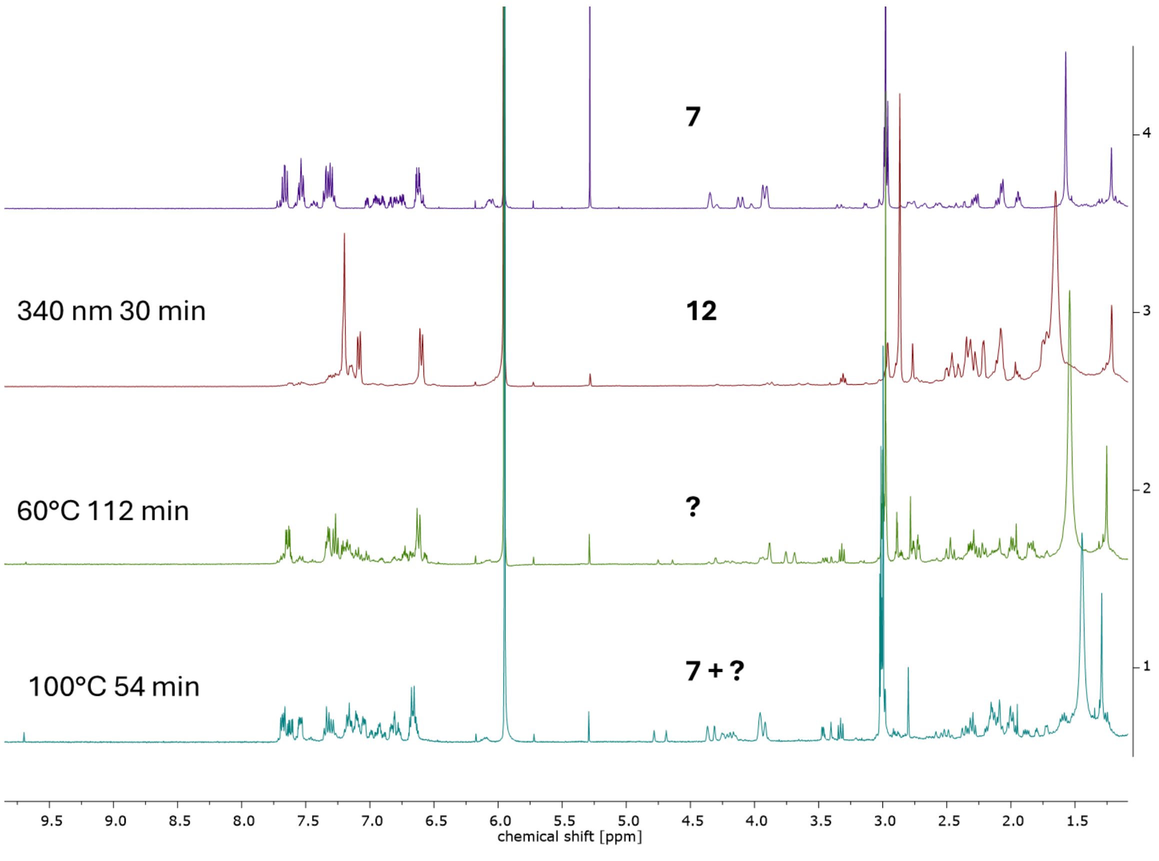

*Figure S 218: Exemplary ^{1}H NMR spectra for the conversion study of **12** to **7** at 60°C (400 MHz). Every spectrum was recorded directly at the used temperature. The same sample of **7** was first irradiated at 340nm to form **12**, which was subsequently heated to 60°C until no further changes in the spectra were observed. Afterwards, the temperature was increased to 100°C.*

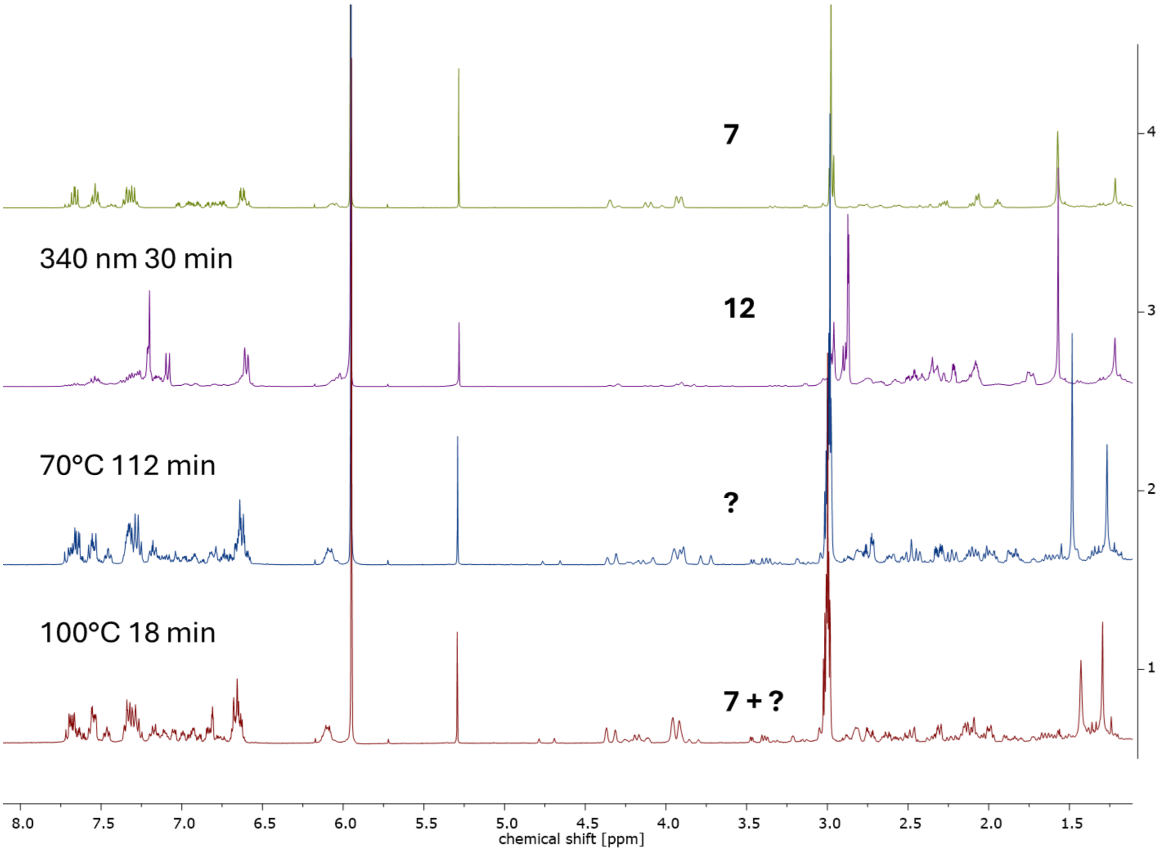

*Figure S 219: Exemplary ^{1}H NMR spectra for the conversion study of **12** to **7** at 60°C (400 MHz). Every spectrum was recorded directly at the used temperature. The same sample of **7** was first irradiated at 340nm to form **12**, which was subsequently heated to 70°C until no further changes in the spectra were observed. Afterwards, the temperature was increased to 100°C.*

$t_{1/2}$ (**12** to **7** at rt) = could not be determined.

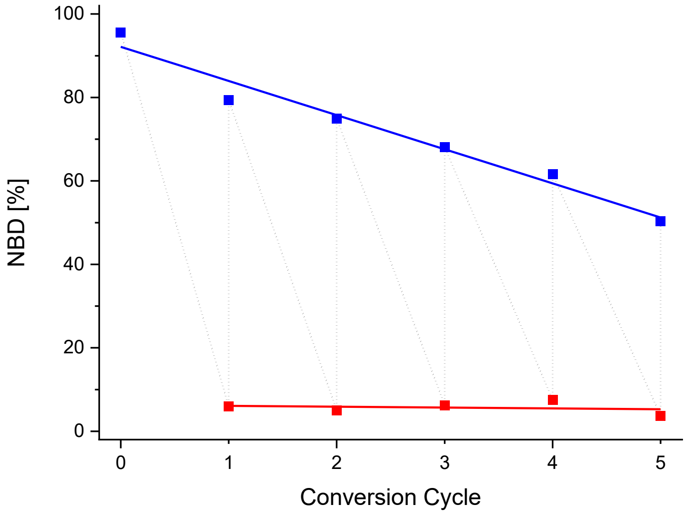

*Figure S 220: Repeated cyclization experiment for the conversion of **7** to **12** and vice versa. A sample was prepared in $C_2D_2Cl_4$ and repeatedly irradiated at 340 nm 30 minutes subsequently heated to 130°C for 10 minutes. After each step, ^{1}H NMR spectrum was recorded to analyze the conversion process. A certain degree of decomposition during each cycle was observed especially during the heating process. Therefore, thermal back-conversion of **12** is possible while occurring decomposition significant.*

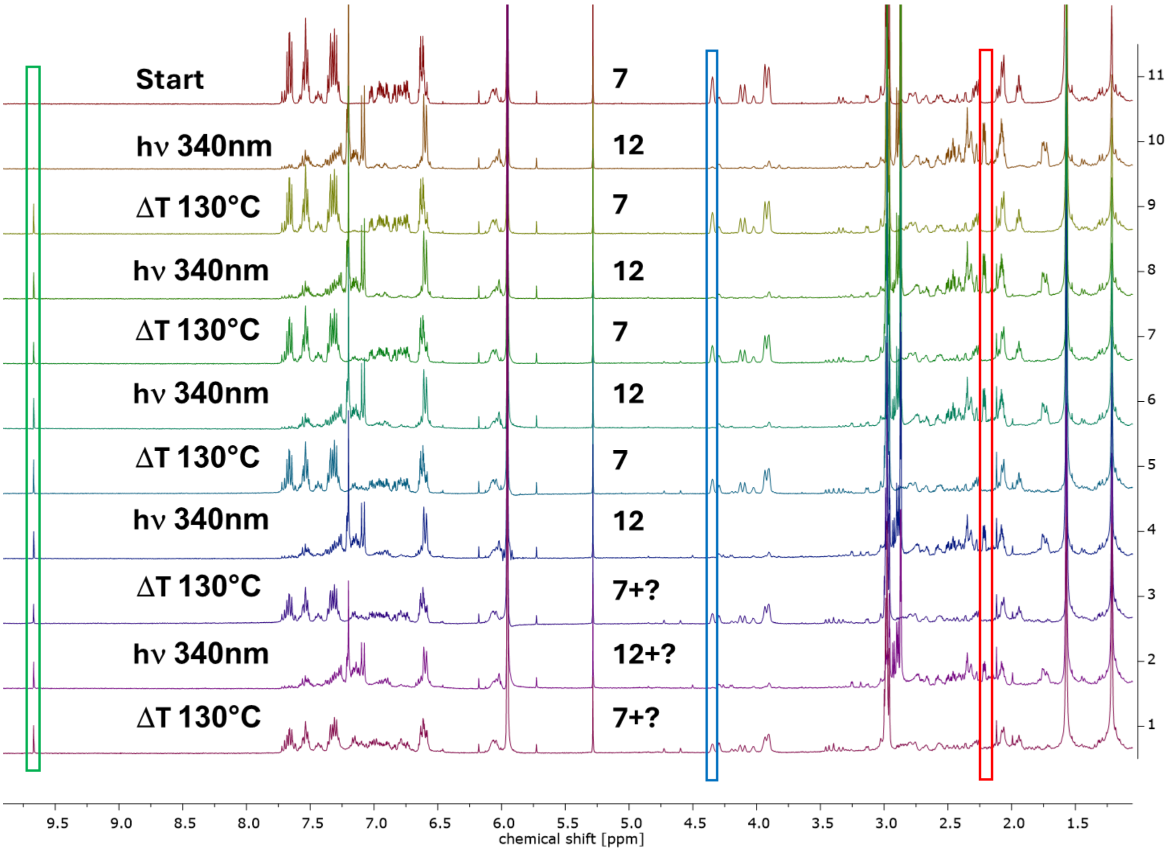

*Figure S 221: Corresponding ^{1}H NMR spectra of the cyclization experiment of **7** to **12** and vice versa. The respective signals which were used for integration are highlighted in green (decomposition species), blue (NBD, **7**) and red (QC, **12**). However, emergence of multiple different additional signals can be observed disturbing proper integration.*

7.4 12b → 7

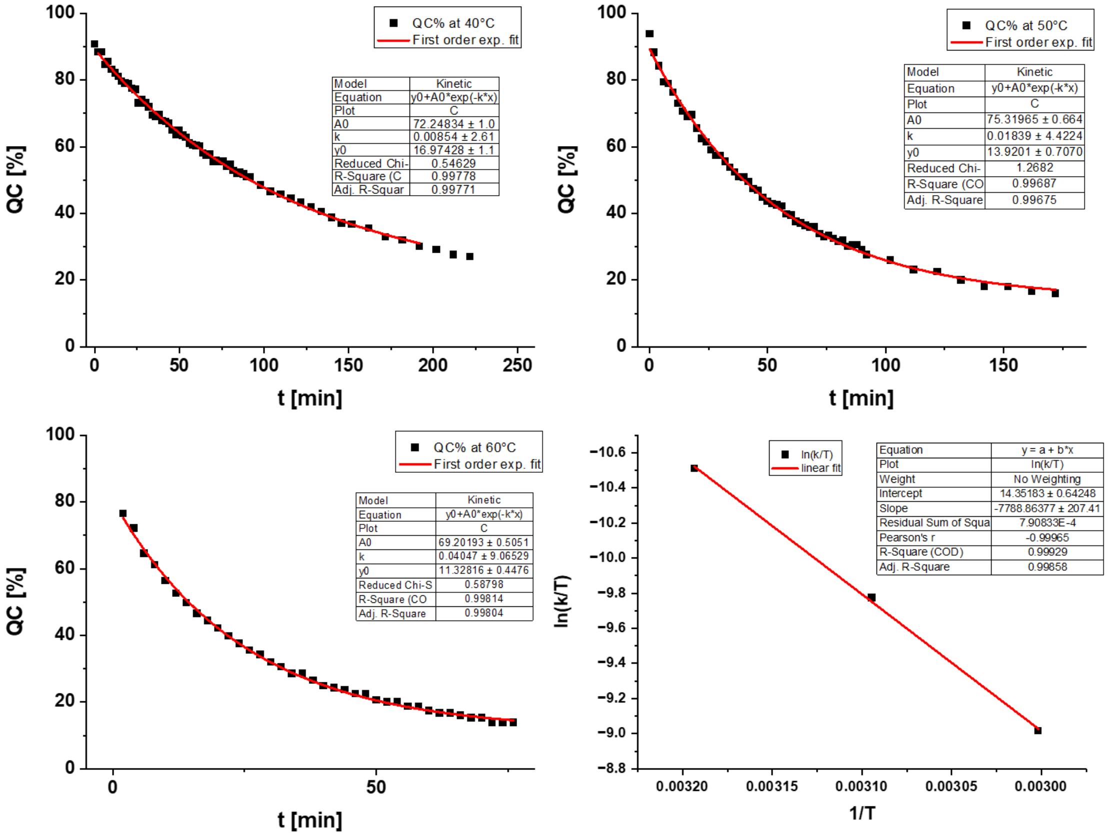

*Figure S 222: Kinetic study of the thermally induced back-conversion of **12b** to **7** measured in $C_2D_2Cl_4$. Assuming first order kinetics, exponential fits were applied to determine the rate constants (k) at different temperatures (40, 50 and 60 °C). Bottom right: Linear fit of the Eyring plot of the obtained data of **12b** to **7**.*

*Table S 24: Estimated k values for the **12b** to **7** conversion obtained from the first order exponential fits*

**T [K]**	313.15	323.15	333.15
**k [s^{-1}]**	0.00854	0.01839	0.04047

From the data obtained from the Eyring plot $\Delta H^{‡}_{thermal}$ of **12b** was estimated to be 15.48 kcal/mol. $\Delta S^{‡}_{thermal}$ was calculated as -18.75 cal*mol^{-1}*K^{-1}. From the extrapolation of the data a thermal half-life at 25 °C of 301 minutes in tetrachlorethane-d_2 was determined for **12b**.

$t_{1/2}$ (**12b** to **7** at rt) = 301 minutes.

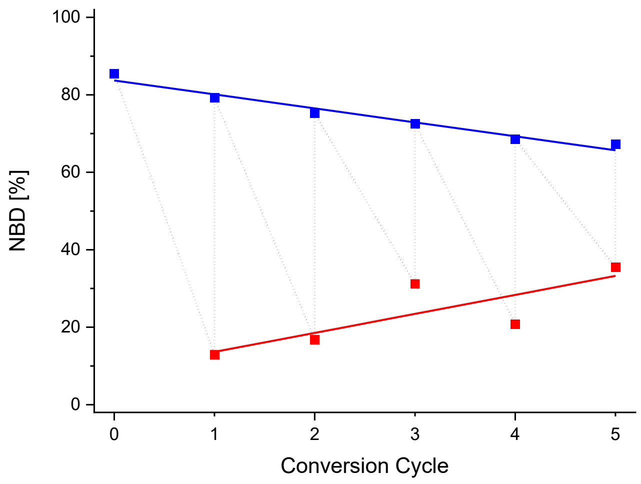

*Figure S 223: Repeated cyclization experiment for the conversion of **7** to **12b** and vice versa. A sample was prepared in $C_2D_2Cl_4$ and repeatedly irradiated at 340 nm 30 minutes subsequently heated to 130°C for 10 minutes. After each step, ^{1}H NMR spectrum was recorded to analyze the conversion process. A significant degree of decomposition during each cycle was observed during both the irradiation and the heating process. Therefore, thermal back-conversion of **12b** is possible but accompanied with massive decomposition. Recovery of **7** from **12b** with **Por** was proven to be possible.*

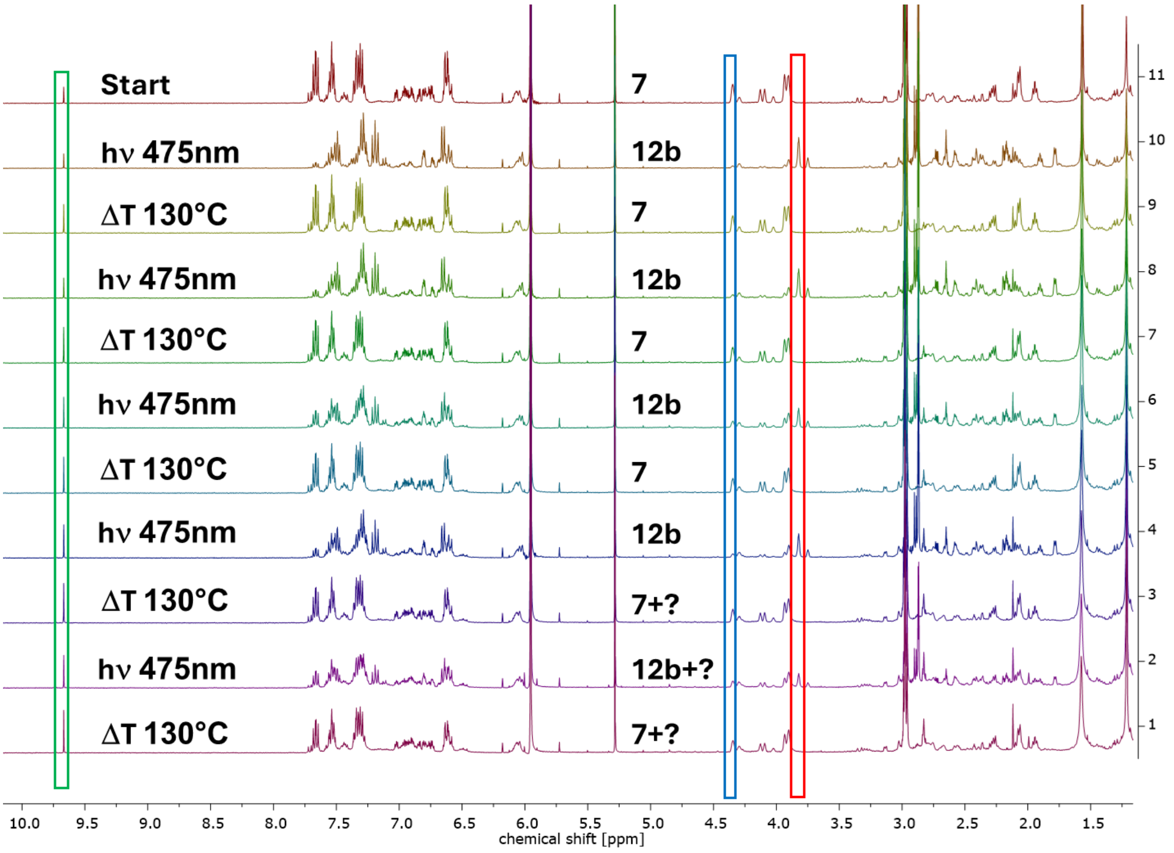

*Figure S 224: Corresponding ^{1}H NMR spectra of the cyclization experiment of **7** to **12b** and vice versa. The respective signals which were used for integration are highlighted in green (decomposition species), blue (NBD, **7**) and red (QC, **12b**). However, emergence of multiple different additional signals can be observed disturbing proper integration.*

7.5 13 → 8

During the cyclization experiments of **8** to **13** and vice versa, massive decomposition was observed. Therefore, only 2 cycles could be conducted before complete degradation of the sample was found. To exclude that degradation was induced by just too high temperatures, Kinetic studies at lower temperatures of 70°C and 90°C were conducted, both resulting in either the formation of unknown side species or decomposition. At 50°C, no changes at all could be observed (compare main article) while potential reversibility was proven by the recovery of **8** upon catalytic back-conversion with **Por**. Therefore, the back-isomerization of **13** (QC-QC-QC) to **8** (NBD-NBD-NBD) no $t_{1/2}$ value could be determined. Combined with the previous findings for less complex systems and the limited amount of available material, further investigations were neglected at this point.

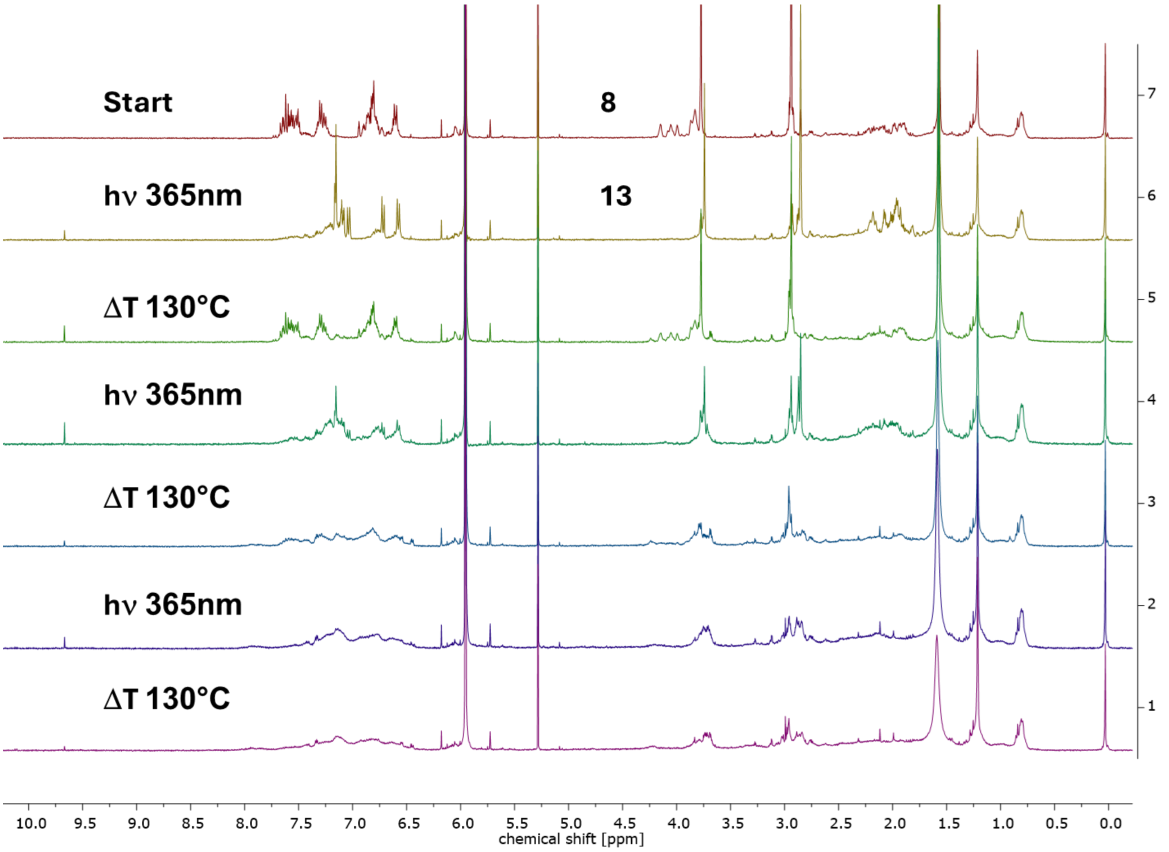

*Figure S 225: ^{1}H NMR spectra of the repeated cyclication experiment for the conversion of **8** to **13** and vice versa. A sample was prepared in $C_2D_2Cl_4$ and repeatadly irradiated at 365 nm (80%, 15°C) for 5 minutes subsequently heated to 130°C for 10 minutes. After each step, ^{1}H NMR spectrum was recorded to analyze the conversion process. After eacht heating step, the sample turned from yellow to green, which was reversible after illumination. However, massive decomposition led to abort of the experiment after 2 cycles.*

$t_{1/2}$ (**13** to **8** at rt) = could not be determined.

8 Computational Methods

All calculations were performed within the Q-Chem 6.0 software package.[21] The range-separated hybrid DFT functional CAM-B3LYP[22] DFT functional was used in combination with the 6-311G* basis set[23] and Grimme's D3(BJ) dispersion correction[24] for ground state geometries as well as for the lowest 20 singlet excited states using the TDDFT/TDA approximation. All ground state geometries were confirmed as local minima with a subsequent frequency calculation. This level of theory was chosen since it performed well on similar NBD/QC systems, also without any solvent effects.[25] As soon as multiple chromophores are introduced within a single molecule, multiple conformers have to be considered. On the one hand, rotamers with degrees of dispersion interactions depending on the interactions of the aromatic side chains. Since solvent interactions are neglected, relative energies of conformers with less dispersion interaction are overestimated. On the other hand, the bridges of the NBD/QC moieties give rise to diastereomers depending on the direction they are pointing to.For the symmetric derivative **5**, a π-stacked conformation where both bridgeheads point in the same direction. This conformer is for all molecules with two chromophores the lowest or at least a low-energy conformer compared to the other conformers. Hence this conformer was chosen to illustrate the simulated spectra to follow the same switching pathway for consistency. For the three-fold substituted compound, the lowest π-stacked conformer was chosen which is where the N,N-dimethylamine and methoxy moieties are in a π-stacked conformation. In general, all UV/Vis spectra were simulated employing a broadening with a FWHM of 0.2 eV and an isovalue of 0.002 was chosen for all detachment/attachment densities.

8.1 (TD-)DFT predicted spectra

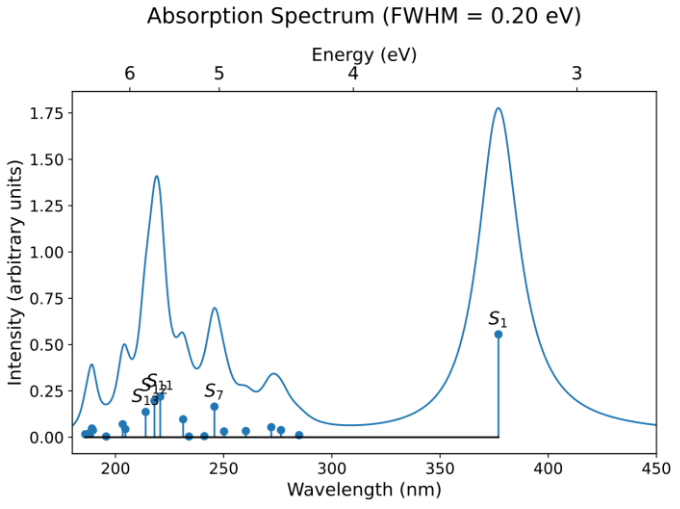

*Figure S 226: (TD-)DFT simulated spectrum of mono-NMe$_2$-NBD **6**.*

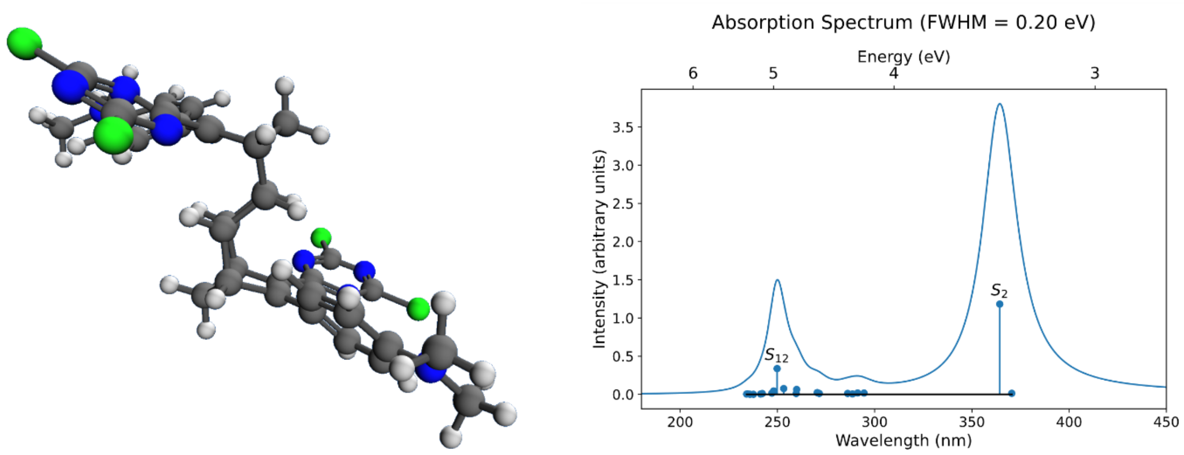

*Figure S 227: Left: Optimized geometry of the dimer formed of two molecules of **6** which is 42.2 kcal/mol more stable than the parent single NBD. Right: respective (TD-)DFT simulated spectrum of the dimer.*

8.2 Geometries

Compound **4**:

32

-1700.7530509084

Cl	5.3631502754	1.2966511409	0.0577733739
Cl	0.7053320929	3.5761022983	-0.3375905147
N	2.9756292565	2.3146231889	-0.1405240936
N	1.0210660091	0.9993995649	-0.2304940948
N	3.1318223559	-0.0358440851	-0.0400037358
C	3.6369014788	1.1748894141	-0.0536202325
C	1.6663021959	2.1372512735	-0.2265138012
C	1.7920454601	-0.0945641488	-0.1286381612
C	1.1917504801	-1.4076886340	-0.1334677038
C	-0.1050065225	-1.7846144264	-0.1471300084
C	-1.3641781231	-1.0391661007	-0.0656652534
C	-1.5658098411	-0.0324995716	0.8786064415
C	-2.7833136674	0.6204157193	0.9615775087
C	-3.8150102676	0.2899588923	0.0930490894
C	-3.6273727655	-0.7098191614	-0.8503427306
C	-2.4166176649	-1.3797544295	-0.9177335644
C	2.0389114421	-2.6927911206	-0.1278210989
C	1.7480929021	-3.4030257286	1.1993187267
C	0.4751913012	-3.7746333153	1.1724580472
C	-0.0955979437	-3.3261515483	-0.1815208432
C	1.1553384493	-3.5523385857	-1.0612496943
H	-0.7630820544	0.2321166040	1.5530633328
H	-2.9258151964	1.3969251907	1.7043180026
H	-4.4276121922	-0.9723526218	-1.5329604430

H	-2.2763273222	-2.1613061638	-1.6561341275
H	3.0830335891	-2.5501618589	-0.3852891079
H	2.4573868907	-3.5052882154	2.0091640182
H	-0.1060806231	-4.2454927043	1.9531552151
H	-1.0360214460	-3.7854982070	-0.4760019102
H	1.4617713910	-4.5977388487	-1.1061101631
H	1.0607253192	-3.1371943721	-2.0665814948
H	-4.7645352590	0.8098305612	0.1532890214

Compound **9**:

32

-1700.7186589399

Cl	0.3947687057	3.2279896484	-1.1030879594
Cl	5.2110238194	1.5067983511	-0.1647275399
N	3.1025300174	-0.0071709274	0.0111675699
N	0.9165641864	0.7718659169	-0.4341059612
N	2.7522819917	2.2420793195	-0.6035847011
C	1.1049087833	-3.8484435989	-0.5697453714
C	-0.1362584785	-3.4599414430	0.1984159222
C	0.2380131549	-2.8618729026	1.5365195028
C	1.6992481928	-2.4219299108	1.3527125240
C	2.0472101743	-2.7920708430	-0.0438403813
C	-2.0872313323	-1.0465196772	-0.9488574437
C	-3.1953069903	-0.2275588328	-1.0945815360
C	-3.6536763397	0.5197281060	-0.0179614635
C	-3.0003876391	0.4393188967	1.2031686327
C	-1.8910725713	-0.3808959872	1.3445072372
C	-1.4219867540	-1.1291392096	0.2705368118
C	-0.2087250968	-1.9663707577	0.4040799013
C	1.2577948705	-1.5070855503	0.2056218392
C	1.7749129520	-0.1919490917	-0.0879291075
C	1.4676422695	1.9407706837	-0.6666324891
C	3.5051475057	1.2091173174	-0.2610935746
H	-0.2637522484	-3.0725565176	2.4700345173
H	2.4164455701	-2.1640874163	2.1183348754
H	-4.5189717029	1.1632728663	-0.1311268754
H	0.9668682771	-3.7775919865	-1.6514404407
H	1.4499422587	-4.8560804094	-0.3239929448
H	-1.0647832281	-4.0095428898	0.1139348299
H	3.0680087092	-2.6813688221	-0.3834924694
H	-1.7231788427	-1.6247523775	-1.7915246711
H	-3.7021310123	-0.1689270797	-2.0514578251
H	-3.3542045046	1.0198715928	2.0479716718
H	-1.3746746974	-0.4381864678	2.2967369198

Compound **5**:
55
-1742.3275797350

Cl	0.3813855532	-3.5424454211	2.7662981406
C	-0.1061450513	-2.0589113128	1.9880993434
C	-1.6696285906	-0.8618113584	0.9363282045
N	-0.8456181181	0.1761279179	0.8399673176
C	0.3766173228	0.0175065360	1.3369343040
C	1.3266827752	1.1068840046	1.2209272775
C	1.3624266839	2.1208613550	0.3366246779
C	-3.0050382638	-0.7565653235	0.3774632464
C	-3.6930203934	0.3254601091	-0.0396044404
C	0.5516481678	2.4080781970	-0.8513948574
C	0.1678333318	1.3937679884	-1.7300368286
H	0.4534803247	0.3718053671	-1.5182528098
C	-0.5554958157	1.6832952875	-2.8729616626
H	-0.8438630563	0.8805039862	-3.5423593686
C	-0.9106941173	2.9945148789	-3.1607352827
H	-1.4818985280	3.2214230096	-4.0541891634
C	-0.5322493561	4.0117871781	-2.2987881444
H	-0.8116318868	5.0375505473	-2.5113085548
C	0.1985041421	3.7215357044	-1.1577799731
H	0.4773833217	4.5227637045	-0.4826802476
C	-3.4520118988	1.7656313239	0.0617997952
C	-2.8723067978	2.3439704973	1.1932191145
H	-2.5531894291	1.7114131811	2.0097500697
C	-2.7204134341	3.7151466797	1.2869292639
H	-2.2750932429	4.1449575136	2.1773748467
C	-3.1394111017	4.5396448224	0.2505084311
H	-3.0175968465	5.6146312730	0.3253566220
C	-3.7146627332	3.9799970722	-0.8791365232
H	-4.0392324322	4.6133588839	-1.6971194989
C	-3.8778362662	2.6068182679	-0.9674425768
H	-4.3270879341	2.1784364237	-1.8563817951
C	-4.9724566229	-0.2230463596	-0.7106064951
C	-3.8357021460	-1.9984910476	0.0108665458
H	-3.5535222218	-2.9040020198	0.5386449604
C	-3.8528064930	-2.0668576101	-1.5211643739
H	-3.3418676737	-2.8168145577	-2.1098496867
C	-4.5284865429	-1.0085860514	-1.9512087353
C	-5.2468266085	-1.4111350675	0.2416832881
H	-6.0511849734	-2.0597072213	-0.1078929496
H	-5.4220280386	-1.1070107127	1.2750513830

C	2.5782433288	1.2203449040	2.1038945845
H	2.5490239030	0.6327671643	3.0166454611
C	3.7897173275	1.0008266263	1.1873942832
H	4.4336826034	0.1330679985	1.2229938700
C	3.8209568549	2.0148192139	0.3333933770
H	4.4936942632	2.1735722415	-0.4981693837
C	2.6314353786	2.9290757228	0.6739952703
H	2.6699838649	3.9328330077	0.2568579648
C	2.6344737185	2.7629966187	2.2110383305
H	3.5487969389	3.1300122851	2.6783586502
H	1.7558017903	3.1933765534	2.6956449662
H	-4.6982542208	-0.6918669986	-2.9715291241
H	-5.7633990014	0.5095075770	-0.8534664009
N	0.7919364256	-1.0998068631	1.9605323635
N	-1.3240381839	-2.0362236986	1.4976329227

Compound **10**:
55
-1742.2576797959

Cl	0.5332870352	-3.8045511346	2.4385226106
C	0.0669318233	-2.2626840625	1.7683920057
C	-1.4890922342	-0.9633328359	0.8390082075
N	-0.6443523696	0.0619337430	0.7809834611
C	0.5816337563	-0.1647669491	1.2414427252
C	1.5366299086	0.9243769940	1.1631631407
C	1.5800337784	2.0767821946	0.1297757310
C	-2.8290620452	-0.7875684529	0.3051090994
C	-3.5678215274	0.4981813313	-0.1504130341
C	0.7199128502	2.2199181994	-1.0635757917
C	0.6000075210	1.1612424792	-1.9646931549
H	1.1373501231	0.2375831902	-1.7763016612
C	-0.1924442160	1.2779246664	-3.0927738735
H	-0.2749554617	0.4454116598	-3.7832769982
C	-0.8709273389	2.4640451364	-3.3473848632
H	-1.4855372673	2.5608775685	-4.2358577256
C	-0.7583074381	3.5202379304	-2.4586415218
H	-1.2949795529	4.4438659655	-2.6396007092
C	0.0235772522	3.3939238028	-1.3188559965
H	0.0791258775	4.2116304335	-0.6100521071
C	-3.2617780475	1.9207508272	0.0993390752
C	-2.6506180549	2.3401385963	1.2789786797
H	-2.3229032762	1.6062586249	2.0027671733
C	-2.4520410024	3.6879630508	1.5326281158
H	-1.9701442799	3.9932511204	2.4553271174

C	-2.8758348180	4.6451282959	0.6208448320
H	-2.7273504998	5.6997920002	0.8251818017
C	-3.4920993189	4.2383882836	-0.5528590069
H	-3.8243589249	4.9746541474	-1.2770498168
C	-3.6714144574	2.8890295280	-0.8142525455
H	-4.1296300491	2.5830081646	-1.7485076047
C	-5.0079207181	0.0212157479	-0.3091841295
C	-3.8866423663	-1.8742332770	0.3211775710
H	-3.6150810263	-2.8198966411	0.7682278800
C	-3.2836552635	-1.4795447393	-0.9880161864
C	-4.0169244107	-0.2025746924	-1.4159979868
C	-5.2498489603	-1.2468443706	0.4724571790
H	-6.0367583006	-1.8652890168	0.0326016627
H	-5.4996117320	-1.0480792608	1.5174119319
C	2.2825672095	1.4649850583	2.3827167346
H	2.2081621462	0.8978391999	3.3014563265
C	3.0495769290	0.8968575508	1.2346516147
C	3.0965016687	2.0363699283	0.2020523040
C	2.3304876618	3.1696216002	0.8453662980
H	2.3808009099	4.1533822282	0.3969808176
C	2.2697150387	2.9754004099	2.3417144963
H	3.1350664486	3.4036355307	2.8546262593
H	1.3576340157	3.3915620424	2.7771125973
H	-5.7644018076	0.7867858495	-0.4213526704
N	0.9899538550	-1.3275587408	1.7714224927
N	-1.1646342434	-2.1752143428	1.3202998235
H	3.7969410297	2.1499088524	-0.6126021974
H	3.6791445558	0.0210569516	1.2904584535
H	-4.0922401642	0.1913049995	-2.4195613725
H	-2.6599202211	-2.1121253670	-1.6023472650

Compound **6**:

40

-1834.6871690816

Cl	0.5312834426	-3.0641678900	3.6918497201
Cl	-2.3743945974	-1.3588624154	-0.2713486229
N	-0.8310370831	-2.1143090473	1.6877471344
N	-0.1165623741	-0.2670032447	0.4076495501
N	1.2012820825	-1.0355818972	2.2076154809
N	-0.8474511488	3.0998525724	-4.6845216087
C	0.2773357363	-1.9449894569	2.3880820916
C	-0.9487571721	-1.2255359738	0.7140318244
C	0.9801476895	-0.1863773602	1.1834465089
C	1.9545818212	0.8470232159	0.9681461791

C	2.0313664168	1.8288716954	0.0314697867
C	1.2712404952	2.1281457699	-1.1720709871
C	0.7802520879	1.1380294222	-2.0288171836
C	0.0972598891	1.4487540065	-3.1827772555
C	-0.1568528697	2.7866837707	-3.5411729167
C	0.3328346650	3.7866827392	-2.6809831805
C	1.0415817784	3.4564458493	-1.5453004591
C	3.1536469885	1.0303960522	1.9166755226
C	4.4155281483	0.7830444651	1.0824176626
C	4.4809251191	1.7520313994	0.1791196793
C	3.2696349772	2.6667070126	0.4138330288
C	3.1841424930	2.5751227778	1.9522170061
C	-1.4600420796	2.0478478460	-5.4652092981
C	-1.1838503590	4.4772852735	-4.9674104611
H	0.9492676158	0.0984893171	-1.7898336274
H	-0.2487550236	0.6389252278	-3.8093047278
H	0.1594409434	4.8312640896	-2.8982102131
H	1.3943116218	4.2607275963	-0.9096596289
H	3.0757518265	0.4904135027	2.8541421318
H	5.0683568773	-0.0721717358	1.1928915226
H	5.1899549668	1.8752615811	-0.6279477990
H	3.3266541877	3.6480304060	-0.0494595620
H	4.0639590747	2.9805674566	2.4530162477
H	2.2728152364	3.0129737443	2.3644876453
H	-2.1956719607	1.4759488527	-4.8876750138
H	-0.7135973078	1.3470147726	-5.8494800088
H	-1.9686971344	2.4850438895	-6.3212379849
H	-1.7002519516	4.5319857509	-5.9227300424
H	-0.2873909423	5.0986998684	-5.0428273583
H	-1.8365341768	4.9139190972	-4.2017507828

Compound **11**:
40
-1834.6502720160

Cl	-2.5438652378	-0.0896919697	-0.3558845072
Cl	-0.2782027548	-2.5963832239	3.5981958185
N	-0.9393064827	3.0369146134	-4.5824148733
N	0.9753503787	-0.8735227284	2.1035083142
N	-0.0620226880	0.2781420701	0.3201387726
N	-1.2907264358	-1.2808131793	1.5927727926
C	-1.8611430000	4.1263122393	-4.3484983415
C	-1.3273915236	2.0347498401	-5.5498749514
C	3.8797169224	2.3480530366	1.6158642387
C	3.8352401648	2.3309688883	0.1059532968

C	4.0044204258	0.9195838468	-0.4110326913
C	3.6358417886	0.0374085263	0.7914637638
C	3.2901242111	0.9831483658	1.8835703525
C	1.0478382590	3.1614929614	-1.4701783754
C	0.1565214928	3.5170035155	-2.4642437903
C	-0.0592019111	2.6819044502	-3.5730527676
C	0.6602331789	1.4769314986	-3.6188589405
C	1.5456252194	1.1391254264	-2.6118180674
C	1.7578358836	1.9677570024	-1.5178078090
C	2.6366509600	1.5648822477	-0.4007054123
C	2.2439225472	0.6816556458	0.8112505373
C	0.9988977298	0.0075851270	1.0874803092
C	-1.1462385732	-0.3951636566	0.6233864059
C	-0.1804975379	-1.4593091253	2.2907606524
H	4.6131556541	0.6391737876	-1.2585228694
H	3.8381557738	-1.0145199172	0.9308295122
H	-2.5255096186	3.9463352539	-3.4921829210
H	-1.3275689848	5.0625862986	-4.1725732947
H	-2.4770184938	4.2702800383	-5.2339613224
H	-2.0099840995	2.4791296989	-6.2717004623
H	-0.4615574574	1.6715438098	-6.1072118388
H	-1.8252058087	1.1685068833	-5.0923701661
H	3.2740183312	3.1507048350	2.0436453516
H	4.8984114898	2.4311948040	2.0036344819
H	4.2017826876	3.1540188627	-0.4932606568
H	3.0987395591	0.6008953764	2.8769266606
H	1.1822879132	3.8251888451	-0.6219774292
H	-0.3784414038	4.4514109136	-2.3685942070
H	0.5257889078	0.7892918105	-4.4417978216
H	2.0757748148	0.1939984422	-2.6718949733

Compound **8**:
90
-2032.3482830810

C	1.0061255141	0.5091657226	0.0273897157
N	2.3187950215	0.2764934488	-0.0364756416
C	2.6856342111	-1.0012480286	-0.1174681446
N	1.8339057321	-2.0259626130	-0.1096967621
C	0.5328642463	-1.7079666787	-0.0102236274
N	0.0809791523	-0.4522656519	0.0471368212
C	-0.5332963361	2.5726684952	-0.1224012380
C	0.6231268536	1.9172467108	0.1090280840
C	1.6403493019	2.9596492079	0.6215798750
C	1.1252526405	3.4284515773	1.9863147914

C	-0.0110518071	4.0809312330	1.7731032219
C	-0.2654519787	4.0486749339	0.2620315276
C	1.1939678981	4.1644264948	-0.2330220054
C	-1.8635424912	-4.6178657081	-1.5772690540
C	-0.5507489021	-4.5382264075	-1.7591261890
C	0.0742626797	-4.2389029483	-0.3917102835
C	-0.3993260479	-2.8258662378	0.0055321610
C	-1.7361685839	-2.9009093413	0.2083631941
C	-2.1233805467	-4.3709002496	-0.0880241245
C	-0.8755569817	-5.0682757329	0.4971823435
C	5.2856395233	-2.7805998418	1.4035545832
C	6.3413058857	-1.9774009117	1.3698178696
C	6.4175015307	-1.3934929199	-0.0480413077
C	5.1757865545	-0.4955170715	-0.2369093007
C	4.1099973027	-1.3147626092	-0.1847623921
C	4.6449847617	-2.7427246546	0.0088228232
C	5.9325988656	-2.6298929546	-0.8408983066
C	-1.8422055793	2.1601509180	-0.6215457751
C	5.3023815295	0.9575402073	-0.3865692492
C	6.1748811844	1.6617251748	0.4430477847
C	6.3164322174	3.0365711993	0.3226719439
C	5.6096364373	3.7249078463	-0.6518245276
C	4.7599867481	3.0289072221	-1.5031627541
C	4.6047088020	1.6605774967	-1.3693874915
C	-2.7556947039	-1.9458726118	0.6162087459
C	-2.9888848833	2.8729853813	-0.2457653605
C	-4.2435263822	2.5062113184	-0.6853161293
C	-4.3897306288	1.4108444873	-1.5306425626
C	-3.2633359808	0.7116780574	-1.9473380551
C	-2.0116390280	1.0871162836	-1.4929258988
C	-4.0775603658	-2.1069683107	0.1902042754
C	-5.0758931428	-1.2190545485	0.5395242964
C	-4.8003765705	-0.1196270738	1.3695804021
C	-3.4850223433	0.0080475953	1.8517549626
C	-2.4982874463	-0.8716075161	1.4728862220
O	-5.6630134009	1.0935915699	-1.8871325547
C	-5.8494692801	0.0255318066	-2.7912498278
N	-5.7677220260	0.7980651674	1.6938848146
C	-5.4808450400	1.8312633830	2.6606369180
C	-7.1156127496	0.6335714691	1.1978429740
H	2.6758089492	2.6440635070	0.5687849340
H	1.5920591412	3.1947720715	2.9339379085
H	-0.6898083918	4.5009220531	2.5038173780
H	-0.9864255326	4.7640006169	-0.1271751083
H	1.3025669133	3.9923816265	-1.3054825692

H	1.6695144400	5.1031529481	0.0555920871
H	-2.6355501754	-4.7547946911	-2.3230790491
H	-0.0024638697	-4.5949283791	-2.6902093331
H	1.1430335413	-4.3973498371	-0.3011569230
H	-3.0948542513	-4.6729319971	0.2957693632
H	-0.7368048866	-4.8835424352	1.5639282765
H	-0.8493222091	-6.1388505444	0.2866956505
H	4.8833512315	-3.3198708969	2.2504673248
H	7.0051232756	-1.7098424166	2.1811299096
H	7.3611756227	-0.9350551986	-0.3365476980
H	3.9336826258	-3.5294930854	-0.2201967868
H	6.5966637663	-3.4892646312	-0.7385378974
H	5.7368065876	-2.4196188570	-1.8939591705
H	6.7327782936	1.1293229652	1.2062111132
H	6.9853439102	3.5694284105	0.9894535111
H	5.7231925952	4.7985477188	-0.7526223386
H	4.2086846616	3.5585757105	-2.2722775968
H	3.9304516644	1.1245192752	-2.0238268817
H	-2.8978962028	3.7213894621	0.4226713047
H	-5.1316121453	3.0423778630	-0.3740435462
H	-3.3465492427	-0.1444299335	-2.6021931340
H	-1.1522622690	0.5157492955	-1.8087425543
H	-4.3331515472	-2.9380205697	-0.4581740336
H	-6.0763561143	-1.3820300609	0.1636607169
H	-3.2205169499	0.8241917570	2.5085551652
H	-1.4961783407	-0.7182473537	1.8430881064
H	-6.9228708725	-0.0511880110	-2.9500032905
H	-5.3575760181	0.2252222040	-3.7476494724
H	-5.4735502782	-0.9138779471	-2.3782000825
H	-5.1982805777	1.4191169504	3.6365243763
H	-4.6698218021	2.4833045563	2.3226041025
H	-6.3658061970	2.4481373270	2.8008541834
H	-7.5918903426	-0.2792390612	1.5769109717
H	-7.7193629364	1.4815757324	1.5139621151
H	-7.1264574338	0.6083500703	0.1059671803

Compound **13**:
90
-2032.2360515198

C	1.1572590475	0.3679917888	0.1605756866
N	2.4582015110	0.1144713743	-0.0009335739
C	2.7841197889	-1.1660134726	-0.1392189731
N	1.9147084292	-2.1730694551	-0.1122420526

C	0.6361108435	-1.8288052388	0.0764309635
N	0.2082306492	-0.5711017836	0.2066532517
C	-0.5274291852	2.5690217647	0.2169249486
C	0.8056970877	1.7776275291	0.3577070163
C	1.8785022195	2.8438147219	0.4638030139
C	1.0702843816	2.5118522165	1.6782476909
C	-0.2431998765	3.2879697881	1.5210940863
C	-0.0655506634	4.0215473262	0.2210674505
C	1.3912880529	4.1297465913	-0.1498692732
C	-1.8808509114	-4.1064332255	-0.8293251849
C	-0.3541589611	-4.0322232158	-0.9596477088
C	0.1747406870	-4.3601447700	0.3945424496
C	-0.3132422027	-2.9410665031	0.1133830348
C	-1.8633105527	-3.0274666665	0.2328815527
C	-2.0983456822	-4.4847394429	0.6111097063
C	-0.8584701002	-5.1059040168	1.1996939418
C	4.9764477658	-2.5632742335	0.4317778361
C	6.1946918848	-1.6262952717	0.4743838517
C	6.5006394041	-1.3603289704	-0.9813435327
C	5.4305663034	-0.5589227099	-0.2725071952
C	4.1927669050	-1.4945396744	-0.3224671922
C	4.6802651155	-2.7550948395	-1.0195772352
C	5.8941044242	-2.4297771416	-1.8575766477
C	-1.8183305196	2.1827051206	-0.3819487106
C	5.4555952327	0.9147596647	-0.1295862722
C	5.6848010285	1.5143008229	1.1031098594
C	5.7157921716	2.8976997434	1.2248754100
C	5.5144514319	3.6983876884	0.1109193559
C	5.2796514551	3.1083112924	-1.1253952687
C	5.2528935871	1.7287031966	-1.2424249172
C	-2.8719325625	-1.9917528862	0.5326817461
C	-2.9807545545	2.8860872478	-0.0490148764
C	-4.1958747606	2.5809697320	-0.6245247996
C	-4.2870108489	1.5439194524	-1.5482253770
C	-3.1452316947	0.8429102757	-1.8999567881
C	-1.9257579207	1.1671776742	-1.3186038491
C	-4.1380729981	-2.0475890897	-0.0401144637
C	-5.1346691273	-1.1471570695	0.2960575824
C	-4.8942862261	-0.1247350345	1.2249968058
C	-3.6210517088	-0.0807463610	1.8135602767
C	-2.6422353059	-0.9922659718	1.4725270608
O	-5.5355967742	1.2792300204	-2.0320320095
C	-5.6549866059	0.2873683030	-3.0255159154
N	-5.8571173509	0.8142780675	1.5329197376
C	-5.6151264300	1.7582302506	2.5944152030

C	-7.1733653488	0.7122089665	0.9486544221
H	6.9062858675	-1.5205155113	1.2812247059
H	-7.7824804873	1.5472869052	1.2894810352
H	-7.1205015774	0.7640379641	-0.1412951295
H	-0.7958982143	4.7635326890	-0.0754429114
H	1.5428837607	4.1520931020	-1.2320112096
H	1.8759128562	5.0050938356	0.2919063771
H	2.9047278929	2.5174624976	0.4014234758
H	1.4605845561	2.0638637322	2.5806319034
H	1.2383915341	-4.4894945227	0.5290652120
H	-3.0937453733	-4.7448219395	0.9471612276
H	-0.7678271635	-4.9121434431	2.2716822789
H	-0.8066155284	-6.1855280369	1.0313093563
H	-0.9363901510	3.5563925606	2.3064215995
H	4.5922918905	-3.1847609952	1.2274978876
H	7.4240997372	-0.8569226996	-1.2376006789
H	3.9282038572	-3.4731561393	-1.3158044950
H	6.5532401180	-3.2937461203	-1.9787523193
H	5.6234477507	-2.0531577452	-2.8475986736
H	5.8352017502	0.8877826330	1.9754272179
H	5.8953856990	3.3502868186	2.1939430339
H	5.5357900374	4.7784766237	0.2043037037
H	5.1159665407	3.7278929618	-2.0003699503
H	5.0651625955	1.2686301486	-2.2066280856
H	-2.9338173205	3.6860256938	0.6835784548
H	-5.0986399589	3.1154169065	-0.3550988843
H	-3.1877683160	0.0158986315	-2.5952244741
H	-1.0552605738	0.5848829172	-1.5832103568
H	-4.3606709850	-2.8160305596	-0.7745803975
H	-6.1005386454	-1.2329103594	-0.1824971887
H	-3.3771774579	0.6930939364	2.5277158489
H	-1.6645274736	-0.9029362474	1.9262433231
H	-6.7092594382	0.2497948551	-3.2920582219
H	-5.0681952874	0.5414091334	-3.9138414884
H	-5.3395136135	-0.6910130266	-2.6528975675
H	-5.4304367160	1.2674333866	3.5587909793
H	-4.7540454701	2.3972144353	2.3733274523
H	-6.4837573511	2.4038634529	2.7074058296
H	-7.6882743838	-0.2157905938	1.2311104192
H	0.2305670542	-4.0314792329	-1.8680536142
H	-2.5871225464	-4.2616422238	-1.6330077997

Compound **7**:

63

-1876.2642111740

Cl	5.4980913292	-0.7444863943	-0.8720944160
C	3.8219776810	-0.5240303925	-0.4317046516
C	1.8217076070	-1.4066697844	0.0307456361
N	1.3064074116	-0.1964544640	0.2342437219
C	2.1282070021	0.8437521143	0.0714990207
C	1.6677388174	2.2043890601	0.2574207441
C	0.4405681651	2.7737325621	0.1916581813
C	1.0067101487	-2.6054793171	0.1655812669
C	-0.2825781181	-2.8082910449	0.5180915114
C	-0.8788896598	2.2645375378	-0.1591276237
C	-1.0663570110	1.1811307238	-1.0228846277
H	-0.2070055358	0.6868923361	-1.4506389881
C	-2.3195927244	0.7215656991	-1.3543011759
H	-2.3971395623	-0.1258163226	-2.0204861971
C	-3.4767046273	1.3173672872	-0.8215017598
C	-3.2983065807	2.4327985873	0.0131191717
H	-4.1511954542	2.9474844940	0.4331822407
C	-2.0327784214	2.8862513924	0.3252494521
H	-1.9470443355	3.7362424153	0.9917449011
C	-1.3630644870	-1.9564872854	1.0189457626
C	-1.1424899224	-0.8695571170	1.8668831442
H	-0.1315137420	-0.5897184496	2.1201688500
C	-2.2055234269	-0.1412980848	2.3711077159
H	-2.0112157426	0.7038370774	3.0211352280
C	-3.5101702950	-0.4778691986	2.0407476081
H	-4.3385844648	0.0984096503	2.4374580641
C	-3.7474324474	-1.5510896352	1.1956066992
H	-4.7629353197	-1.8180768230	0.9249576780
C	-2.6847386227	-2.2854906701	0.6967581263
H	-2.8827295091	-3.1248102717	0.0400151749
C	-0.5461578594	-4.3166233845	0.2896318160
C	1.5761065976	-3.9717581497	-0.2695667500
H	2.6585978936	-4.0169537353	-0.3043009358
C	0.8333030371	-4.3641185313	-1.5516140463
H	1.2788665744	-4.3921897224	-2.5368557214
C	-0.4345134174	-4.5705506784	-1.2185159411
C	0.8243709784	-4.8702342040	0.7338559639
H	0.9371348389	-5.9365062620	0.5332154399
H	1.0647935195	-4.6490350130	1.7752810582
C	2.6867600916	3.3396888003	0.4722559427
H	3.6677893986	3.0134366963	0.8016872236
C	2.6477134462	4.2151402453	-0.7855315395
H	3.4452593793	4.2785225946	-1.5130809777
C	1.4460851806	4.7748183495	-0.8300231925

H	1.0225679975	5.4016509725	-1.6027939116
C	0.6776412913	4.2853432490	0.4074416907
H	-0.2015360181	4.8670597817	0.6701827348
C	1.8449243756	4.2266045136	1.4142642955
H	2.2928455668	5.2025144715	1.6047766577
H	1.5851456904	3.7332166690	2.3530334698
H	-1.2683897357	-4.8072083067	-1.8659931720
H	-1.4322597522	-4.7040355095	0.7862598502
N	3.4324715934	0.7125521614	-0.2459198125
N	3.1037793698	-1.6160968055	-0.3294763001
N	-4.7267056355	0.8184216150	-1.0975553093
C	-4.8792672537	-0.2382779296	-2.0711930349
H	-4.5237640070	0.0560264837	-3.0658791692
H	-5.9317289440	-0.5003652252	-2.1545066862
H	-4.3357838035	-1.1374353265	-1.7689510535
C	-5.8979983576	1.5025919211	-0.6035236740
H	-5.8765196146	1.5873928780	0.4866870703
H	-6.7866226463	0.9347019018	-0.8701521137
H	-6.0035379275	2.5130497970	-1.0180803310

Compound **12**:
63
-1876.1914200002

Cl	-5.3547896969	-0.8651440054	1.1353945346
C	-3.7324992798	-0.4994972710	0.6051870439
C	-1.6932965978	-1.2034429608	0.0396621756
N	-1.3142960690	0.0366945791	-0.2511332907
C	-2.2139643118	0.9915432337	-0.0364240334
C	-1.7787347652	2.3630102051	-0.2315156576
C	-0.3501428202	2.9161213284	0.0153650748
C	-0.7103697265	-2.2711419045	-0.0643552212
C	0.6910300441	-2.2990497740	-0.7325684547
C	0.7778304723	2.1224388206	0.5398795103
C	0.6582297874	1.4530149953	1.7543416398
H	-0.2383491036	1.5896971040	2.3514405964
C	1.6394701314	0.5960389190	2.2140920313
H	1.4830605345	0.0960155094	3.1600387095
C	2.8080540679	0.3766896496	1.4691367825
C	2.9594039137	1.1102581763	0.2869345002
H	3.8480559057	1.0066481005	-0.3168016652
C	1.9524339072	1.9398878120	-0.1745062223
H	2.0813729354	2.4344378149	-1.1308623008
C	1.3229037569	-1.3944164366	-1.7119133266
C	0.5828480699	-0.5955126925	-2.5787446403

H	-0.4954002791	-0.5855264093	-2.5113129557
C	1.2158596181	0.2072313632	-3.5163540827
H	0.6189852506	0.8285956438	-4.1756838639
C	2.5990214073	0.2160135820	-3.6193303531
H	3.0905482339	0.8411427461	-4.3564707519
C	3.3459946121	-0.5899499324	-2.7712931832
H	4.4289462896	-0.5966622501	-2.8401244468
C	2.7133022792	-1.3776428918	-1.8246217407
H	3.3098824112	-1.9801849236	-1.1476995789
C	1.0591929431	-3.7784897373	-0.6873263144
C	-0.9915636246	-3.7054010965	0.3312713638
H	-1.9905087913	-3.9381128368	0.6716672608
C	-0.0886185395	-2.8877620756	1.1992327306
C	1.2849329237	-2.9143682721	0.5188504317
C	-0.1718337872	-4.6362957698	-0.5273098783
H	0.0397997303	-5.5816052199	-0.0202190498
H	-0.6547929738	-4.8492891577	-1.4839837433
C	-2.4200525852	3.3506027118	-1.2009101056
H	-3.3572023024	3.0665566759	-1.6619990650
C	-2.3883568745	3.6574845735	0.2621305438
C	-0.9729232477	4.2053755286	0.5147211794
C	-0.2973296553	4.1543830610	-0.8393170485
H	0.6506722717	4.6607268951	-0.9686701517
C	-1.3321903673	4.0944824664	-1.9394498350
H	-1.6627963872	5.0868374986	-2.2583667583
H	-0.9762880298	3.5422503982	-2.8129659674
H	1.9032507774	-4.0888306140	-1.2890716582
N	-3.4710821331	0.7676623241	0.3743326055
N	-2.9166079490	-1.5230556797	0.4946649737
N	3.7631113009	-0.5594327815	1.8719005339
C	3.7195400784	-1.0279032846	3.2406266623
H	3.8407213913	-0.2206444265	3.9767961245
H	4.5158734277	-1.7539556272	3.3947800855
H	2.7769750878	-1.5366747075	3.4484676517
C	5.0800425588	-0.4816864099	1.2752125979
H	5.0273766738	-0.6278062347	0.1960101423
H	5.7022827158	-1.2787194701	1.6786458577
H	5.5806605890	0.4785374273	1.4640460473
H	2.2374250808	-2.6834473073	0.9756062354
H	-0.3111013053	-2.5753229737	2.2087146793
H	-3.2656784931	3.7623524691	0.8833763269
H	-0.6513814823	4.8271535224	1.3378287123

Compound **12b**:

63
-1876.2272759349

Cl	-4.4052784591	-3.4166219197	0.4568923950
C	-3.1384944885	-2.2447106240	0.1943253842
C	-0.9993375090	-1.8059771187	-0.2814783414
N	-1.2475028210	-0.4969206588	-0.2930444296
C	-2.4847450083	-0.1277289334	0.0141656051
C	-2.7502603779	1.2942424280	0.1389585927
C	-1.7482106377	2.3984426439	0.5692058652
C	0.3498673216	-2.2880733062	-0.5241601028
C	1.4318782255	-1.6960880733	-1.0724303836
C	-0.3416606098	2.1597172538	0.9445701065
C	-0.0285375818	1.2498934070	1.9498399005
H	-0.8303246670	0.7832652427	2.5135667223
C	1.2761347028	0.8999566258	2.2355487136
H	1.4560599640	0.1730062041	3.0146806561
C	2.3433798033	1.4561376181	1.5142598915
C	2.0306505022	2.4252945695	0.5533566513
H	2.8112786442	2.9075926337	-0.0154228470
C	0.7158001390	2.7463516533	0.2649977125
H	0.5121751335	3.4579807340	-0.5275910468
C	1.6550739027	-0.4229774596	-1.7586467958
C	0.6642217171	0.2074378895	-2.5151819812
H	-0.3231072525	-0.2269224300	-2.5673710217
C	0.9353008748	1.3735102089	-3.2078477145
H	0.1518580149	1.8428744873	-3.7925855342
C	2.2053437282	1.9346090282	-3.1693703919
H	2.4178973030	2.8441426821	-3.7205155543
C	3.2002337003	1.3191226815	-2.4260674650
H	4.1964183828	1.7472158090	-2.3905505203
C	2.9278461280	0.1533699279	-1.7288921020
H	3.7081425136	-0.3031157636	-1.1321205427
C	2.5947160548	-2.7002109237	-0.8896660044
C	0.7975600131	-3.6688675527	-0.0060542590
H	-0.0144213340	-4.3606363825	0.1925247976
C	1.7758756101	-3.3996438240	1.1444500403
H	1.5675567252	-3.5952129648	2.1878968177
C	2.8469432392	-2.8194031760	0.6171958724
C	1.8147943937	-4.0157257082	-1.1137348600
H	2.4097312082	-4.9044586569	-0.8980772670
H	1.3627789569	-4.0879851690	-2.1044550234
C	-3.8571554749	2.0348301555	-0.6056387395
H	-4.5735299064	1.4410049006	-1.1583916160
C	-3.8691846784	1.9921978082	0.8872974416
C	-2.8748506949	3.0837178832	1.3185983232

C	-2.3575797697	3.6607134946	0.0187025479
H	-1.7801560384	4.5759770484	0.0476199529
C	-3.3164583689	3.3536776851	-1.1083407255
H	-4.1040575310	4.1061705186	-1.2037659140
H	-2.8077553291	3.2517623178	-2.0706080003
H	3.7159876491	-2.4242098344	1.1253346024
H	3.4637822577	-2.5159614744	-1.5160644455
N	-3.4964433391	-0.9828798267	0.2413481419
N	-1.9360641033	-2.7326459031	-0.0132874754
N	3.6570996992	1.0365887146	1.7218507004
C	3.9404323089	0.2467436163	2.9003911938
H	3.7273035549	0.7783911994	3.8390017887
H	4.9928615345	-0.0330278146	2.8974111900
H	3.3598174386	-0.6761504351	2.8933739902
C	4.7259281850	1.8970427222	1.2618086925
H	4.6881949041	2.0218105941	0.1792170896
H	5.6831794430	1.4358793711	1.4994867148
H	4.6995175449	2.8957484995	1.7202340417
H	-4.6383169832	1.5186127496	1.4794201854
H	-2.8320200709	3.5857818054	2.2744702436

Compound **12a**:

63

-1876.2283414184

Cl	-5.6162905229	-0.4205181635	1.0736814321
C	-3.9490486742	-0.3185163991	0.5598259849
C	-2.0280901964	-1.3261563121	0.0431685853
N	-1.4391879716	-0.1642055663	-0.1999545782
C	-2.1968684483	0.9318664385	-0.0429591135
C	-1.6683435497	2.2538015158	-0.2997500379
C	-0.4137675367	2.7647262953	-0.2652815973
C	-1.3004907681	-2.5804003445	-0.1036317894
C	0.1796156076	-2.9312479767	-0.4266074449
C	0.8765202981	2.2101430929	0.1174635077
C	1.0049265252	1.1555198822	1.0269070458
H	0.1189561494	0.7199556649	1.4642956407
C	2.2319003728	0.6567821457	1.3961092048
H	2.2643499090	-0.1618212501	2.1008016960
C	3.4214236705	1.1794900465	0.8572598476
C	3.3029560339	2.2629769362	-0.0293024085
H	4.1832735891	2.7172405222	-0.4621316335
C	2.0635126246	2.7586312876	-0.3769409133
H	2.0236805909	3.5822937495	-1.0798684239
C	1.3136551494	-2.1085690289	-0.8955266103

C	1.1382000683	-1.0435488484	-1.7751917342
H	0.1386582504	-0.7281621938	-2.0384989409
C	2.2327092975	-0.3619466422	-2.2828805915
H	2.0739077978	0.4770830750	-2.9506727894
C	3.5215266320	-0.7387113212	-1.9365698457
H	4.3747170438	-0.2050541223	-2.3405346783
C	3.7083284690	-1.7928595588	-1.0553897354
H	4.7106871433	-2.0892018552	-0.7650848147
C	2.6134545849	-2.4637577805	-0.5333150080
H	2.7723573914	-3.2825077580	0.1612409282
C	0.1087251164	-4.4098047771	-0.7973937258
C	-2.0595371535	-3.8955909970	-0.2810677833
H	-3.1380719572	-3.8394622114	-0.2668033586
C	-1.3029431929	-3.6655536160	0.9794078760
C	0.1563187873	-4.0008249918	0.6472733992
C	-1.2878200051	-4.8034727462	-1.2039690151
H	-1.4993948464	-5.8584419563	-1.0085566112
H	-1.4885300051	-4.5928425135	-2.2571468279
C	-2.6306932929	3.4264394601	-0.5712351424
H	-3.6283525909	3.1325438731	-0.8810233585
C	-2.5427182926	4.3632396362	0.6390879676
H	-3.3329806837	4.5038624944	1.3636431111
C	-1.3146498323	4.8637163859	0.6518820457
H	-0.8566415413	5.5079320046	1.3898906737
C	-0.5770433557	4.2727156849	-0.5600638334
H	0.3286068072	4.7957599631	-0.8540289735
C	-1.7500361286	4.2186773562	-1.5601438319
H	-2.1496542374	5.2043138835	-1.8012639050
H	-1.5189771169	3.6637538393	-2.4716417521
H	0.9887347015	-4.8373512891	-1.2596678651
N	-3.4913941483	0.8879379119	0.3228532569
N	-3.3071284163	-1.4533756653	0.4543572753
N	4.6441993341	0.6478582507	1.1812183738
C	4.7343616000	-0.4118787400	2.1587182920
H	4.3974926166	-0.0926298686	3.1523135081
H	5.7694931360	-0.7354504368	2.2430339415
H	4.1388003725	-1.2777508069	1.8584042756
C	5.8504483618	1.2475858130	0.6629884977
H	5.8617058685	1.2426883127	-0.4313783364
H	6.7116652859	0.6774522036	1.0037412972
H	5.9787013710	2.2842675549	0.9968814126
H	0.9291492171	-4.2717138782	1.3528369860
H	-1.7492553101	-3.5626356647	1.9575209460

Compound **12c**:

64

-1876.6402513143

Cl	-5.4590841767	-0.7403127257	0.0697381968
C	-3.7661526694	-0.4071331710	-0.0367827814
C	-1.6527087887	-1.1489225291	-0.1139491611
N	-1.1882686264	0.0868279386	-0.2772405029
C	-2.1025155294	1.0640823946	-0.2589028326
C	-1.6750806651	2.4542525850	-0.3363787041
C	-0.4626379790	2.9827538979	-0.1123070313
C	-0.7498949640	-2.3002603678	-0.0262473133
C	0.4953707644	-2.5628741022	-0.4768508993
C	0.7674688505	2.2951928076	0.2880889406
C	0.7999991832	1.4927758435	1.4255033206
H	-0.0692128714	1.4460383018	2.0681775704
C	1.9014206648	0.7084945002	1.7139090595
H	1.8713675271	0.0562473292	2.5751974956
C	2.9825740303	0.7475877364	0.8532423512
C	3.0117445410	1.5995969450	-0.2359827747
H	3.8574125283	1.6137322859	-0.9127179766
C	1.9068374405	2.3837087798	-0.5061519720
H	1.9043413976	3.0177520056	-1.3844814965
C	1.4721799990	-1.8168565559	-1.2778723694
C	1.1158009381	-0.7999534419	-2.1688297160
H	0.0826977640	-0.4967389541	-2.2440267188
C	2.0661234627	-0.1835597017	-2.9657300380
H	1.7555946566	0.5851187530	-3.6640598784
C	3.4024512367	-0.5526726489	-2.8937875592
H	4.1362310821	-0.0855795847	-3.5405933961
C	3.7812978697	-1.5596438223	-2.0135943888
H	4.8091975449	-1.9101070720	-1.9962446154
C	2.8241393016	-2.1870522835	-1.2245253232
H	3.1295411311	-3.0092531027	-0.5888220313
C	0.8269084778	-3.9943639045	0.0196308951
C	-1.2190412711	-3.5449907545	0.7553937819
H	-2.2878665873	-3.5863639900	0.9299184523
C	-0.3136472760	-3.6559295993	1.9873489495
H	-0.6433431892	-3.5118586959	3.0073422067
C	0.9091505585	-3.9325453722	1.5495727335
C	-0.5706827033	-4.6336590841	-0.1258237913
H	-0.6289156491	-5.6334608284	0.3041529194
H	-0.9435079379	-4.6360137938	-1.1508667725
C	-2.6326033745	3.6236681443	-0.6078274822
H	-3.5779446071	3.3466708153	-1.0627691781
C	-2.7090289464	4.4392143859	0.6906374322

H	-3.5892165322	4.5112673559	1.3138437073
C	-1.5088160038	4.9639879875	0.8980996912
H	-1.1718895528	5.5657843810	1.7305311383
C	-0.6089155719	4.5056132847	-0.2636449456
H	0.3210398311	5.0554733554	-0.3969761325
C	-1.6606155746	4.5310448113	-1.4016085517
H	-2.0590956253	5.5272865214	-1.5889171412
H	-1.3086949021	4.0796574854	-2.3314344266
H	1.8089382714	-4.0865312232	2.1318250062
H	1.6469589138	-4.4891844979	-0.4938623743
N	-3.4197235513	0.8580701222	-0.1617391908
N	-2.9556438588	-1.4395958056	0.0248398130
N	4.1348846276	-0.1763347512	1.0073257153
C	3.9018188707	-1.3109121343	1.9454932344
H	3.8616138554	-0.9327773555	2.9633588777
H	4.7331437734	-2.0053426217	1.8496798368
H	2.9696515096	-1.8042652462	1.6871156995
C	5.4012838023	0.5408920239	1.3509642761
H	5.5868087051	1.3159197819	0.6140846305
H	6.2226600935	-0.1727373595	1.3582425159
H	5.2825335828	0.9924699927	2.3327486057
H	4.2397021976	-0.6000654713	0.0757124141

Compound **12f**:

64

-1876.5742428070

Cl	-4.8533620498	-2.8562049407	1.0109747687
C	-3.4609456974	-1.9322952158	0.5550308620
C	-1.3072048915	-1.8612009896	-0.0568675060
N	-1.3399130540	-0.5315340587	-0.1634484392
C	-2.5119884792	0.0401000077	0.1381560602
C	-2.6449835977	1.4884537798	0.1039066408
C	-1.5936472792	2.6063653317	0.3117918877
C	-0.0935346240	-2.6280301094	-0.3243656942
C	1.3847763278	-2.2833043136	-0.6740255515
C	-0.1813674082	2.4048622089	0.6641290896
C	0.1687368362	1.4506045404	1.6185859306
H	-0.6064176400	0.9057079437	2.1407740943
C	1.4905536887	1.1570815436	1.8923801214
H	1.7141774517	0.3814364666	2.6107990742
C	2.4809687555	1.8459525283	1.2141857615
C	2.1645992074	2.8458415627	0.3128569461
H	2.9344499226	3.3712994162	-0.2377806077
C	0.8362976532	3.1209344322	0.0442176206

H	0.5914671644	3.8693779403	-0.6990193829
C	2.0669351910	-1.0515961621	-1.1115708400
C	1.4025519771	-0.0267304001	-1.7854611384
H	0.3248998151	-0.0597117049	-1.8642078036
C	2.1096487854	1.0268830186	-2.3442925727
H	1.5718714868	1.7988962861	-2.8824524258
C	3.4925773250	1.0945489167	-2.2388220919
H	4.0420529067	1.8988617517	-2.7148291813
C	4.1672626994	0.0917354282	-1.5490674005
H	5.2530460588	0.0916671451	-1.5089396275
C	3.4576157040	-0.9619858212	-0.9833474756
H	4.0033801957	-1.7587588785	-0.4882604892
C	1.9418373064	-3.6222852404	-1.1700166387
C	-0.2149903003	-4.1285966265	-0.6198965037
H	-1.2115372953	-4.5427203900	-0.6030682464
C	0.4112452674	-3.6986275775	0.6561196140
C	1.8613114955	-3.3492496897	0.2998932888
C	0.8343951022	-4.5372265282	-1.6172043700
H	1.1022133754	-5.5917813930	-1.5178498491
H	0.5279561416	-4.3465012556	-2.6476123873
C	-3.7866920817	2.1759706311	-0.6512777474
H	-4.5519503764	1.5379928743	-1.0716489409
C	-3.6941020617	2.3213294422	0.8263793451
C	-2.6478809494	3.4249602101	1.0415275607
C	-2.2111249970	3.8179596250	-0.3469112360
H	-1.6218189460	4.7177733204	-0.4704391206
C	-3.2534434286	3.3988144922	-1.3571648028
H	-4.0270140089	4.1584009150	-1.4893223300
H	-2.8224425749	3.1609734137	-2.3321926975
H	2.9006881482	-3.5954366464	-1.6701350626
N	-3.6163358084	-0.6281134250	0.4858250399
N	-2.3580159092	-2.6040110479	0.3260351298
N	3.9135625654	1.4662388222	1.3326700502
C	4.1749586104	0.2635308215	2.1701865469
H	3.9170558017	0.4799412967	3.2036396120
H	5.2334102008	0.0258885314	2.0985078620
H	3.5870939818	-0.5663676260	1.7917727535
C	4.7776122061	2.6028698749	1.7757197384
H	4.6513620106	3.4421438954	1.1004001327
H	5.8161345987	2.2789935137	1.7723197456
H	4.4738602314	2.8929802534	2.7785374383
H	2.7030080534	-3.3260345638	0.9790759204
H	0.0012496767	-3.8809328956	1.6383854131
H	-4.4263575497	1.9520802374	1.5283000304
H	-2.5133613494	4.0318708380	1.9250206084

H	4.1829184319	1.1992442429	0.3733234733

Compound **12d**:

64

-1876.6090790034

Cl	4.5619206374	-3.1390895821	0.2859423980
C	3.2251043730	-2.0445265928	0.1927723738
C	1.0115448142	-1.7038767893	0.2270324481
N	1.1733803838	-0.3813531464	0.1300997809
C	2.4385664720	0.0362182187	0.0224888125
C	2.7236476694	1.4450896015	-0.1793341126
C	1.7878177233	2.5914503643	-0.6353523643
C	-0.3194575343	-2.3200673031	0.2921213261
C	-1.5097672355	-1.9453334761	0.8024014760
C	0.3480893930	2.4183121333	-0.8910223610
C	-0.0829364465	1.6286033196	-1.9529706027
H	0.6437238630	1.2407699348	-2.6564283725
C	-1.4134372787	1.2774941890	-2.0892276761
H	-1.7092798703	0.6206359552	-2.8958421848
C	-2.3139540635	1.7434282489	-1.1489607232
C	-1.9288908831	2.5932150062	-0.1305279748
H	-2.6439723257	2.9382431055	0.6069503635
C	-0.5935258820	2.9277042023	-0.0061240756
H	-0.2702989161	3.5473045907	0.8211480051
C	-1.9801210523	-0.7526159071	1.5140691700
C	-1.1283184973	0.0814664568	2.2424740491
H	-0.0669989548	-0.1171909681	2.2487440618
C	-1.6264575229	1.1488644958	2.9715654395
H	-0.9442191497	1.7638911259	3.5471665424
C	-2.9870397379	1.4240760240	2.9888101181
H	-3.3730898443	2.2451297669	3.5816945499
C	-3.8534795943	0.6098889249	2.2695962271
H	-4.9250027904	0.7759892269	2.3208700657
C	-3.3539645563	-0.4684541311	1.5484606192
H	-4.0500918862	-1.1309964714	1.0464766013
C	-2.4659008364	-3.1453361443	0.5683417693
C	-0.4989560045	-3.7465322211	-0.2704633741
H	0.4306929276	-4.2739517750	-0.4505974395
C	-1.4758748500	-3.6443166617	-1.4456263960
H	-1.2134320128	-3.8193399602	-2.4802831840
C	-2.6557013769	-3.2965841985	-0.9444105495
C	-1.4642547754	-4.2981298477	0.8031603231
H	-1.8754426529	-5.2771087956	0.5570572166
H	-1.0336719270	-4.3037501867	1.8051498268

C	3.9281854860	2.1553884634	0.4407942881
H	4.6315267069	1.5429915912	0.9878971856
C	3.8499879179	2.0228514113	-1.0375851907
C	2.9034827889	3.1472345378	-1.4899576925
C	2.5101111763	3.8429337744	-0.2048499638
H	1.9970060028	4.7945383935	-0.2683206564
C	3.5132053515	3.5387499738	0.8828186492
H	4.3548587595	4.2346398147	0.8757020027
H	3.0668436013	3.5380254096	1.8799147755
H	-3.5880465450	-3.1460461366	-1.4734718345
H	-3.3660523560	-3.1461586756	1.1772493913
N	3.5089805859	-0.7704717197	0.0665829888
N	2.0223340035	-2.5796928843	0.2456485442
N	-3.7328767409	1.3098077316	-1.1487838298
C	-3.9803002331	0.0191793802	-1.8517854150
H	-3.9033909394	0.1719053855	-2.9246318954
H	-4.9864659730	-0.3137835655	-1.6069605060
H	-3.2495331398	-0.7133193843	-1.5203027388
C	-4.6539072848	2.3805444301	-1.6386281835
H	-4.5049966776	3.2757843522	-1.0420494261
H	-5.6819372205	2.0345271139	-1.5507644964
H	-4.4122454754	2.5905521251	-2.6775513849
H	4.5389776969	1.4509427982	-1.6410355928
H	2.8452105534	3.5947700910	-2.4717215156
H	-3.9394978437	1.1324748552	-0.1582096764

Compound **12e**:
64
-1876.6066937064

Cl	-5.3922313196	-0.5866231981	1.2660799608
C	-3.7743384892	-0.3871710073	0.6853663310
C	-1.7765001591	-1.2658425819	0.1787712131
N	-1.3079736847	-0.0820708886	-0.2054742189
C	-2.1652424511	0.9429246104	-0.0986446540
C	-1.7399085078	2.2962067358	-0.4385744312
C	-0.5278557997	2.8653413462	-0.3609669013
C	-0.9497811230	-2.4673031178	0.1403081045
C	0.5400980789	-2.7672696004	-0.2000716036
C	0.7384922493	2.2886237889	0.0884112019
C	0.8097564776	1.5083879880	1.2400050533
H	-0.0718167085	1.3811536834	1.8532389542
C	1.9816267192	0.8695902939	1.6004176493
H	1.9849364873	0.2461790719	2.4829548610
C	3.1009250167	1.0278357013	0.8036491993

C	3.0790524493	1.8579474335	-0.3042496969
H	3.9471755301	1.9718996496	-0.9410599333
C	1.9029840349	2.4956751665	-0.6475259985
H	1.8767935074	3.1166145515	-1.5347102040
C	1.6101396220	-1.9910267593	-0.8533693233
C	1.3445339082	-0.9807202611	-1.7784412255
H	0.3256311925	-0.6498340685	-1.9218208832
C	2.3721008347	-0.4014506878	-2.5066880805
H	2.1372117775	0.3641880591	-3.2372014310
C	3.6899236467	-0.8021291537	-2.3266547324
H	4.4816888683	-0.3756555503	-2.9323165858
C	3.9730707079	-1.7936140628	-1.3921704081
H	4.9870317191	-2.1685521503	-1.2828319252
C	2.9421565969	-2.3717191921	-0.6584886024
H	3.1730766877	-3.1760842175	0.0326194617
C	0.5531950838	-4.2904961315	-0.3688865266
C	-1.6215728428	-3.8453146324	0.1694192946
H	-2.6996302140	-3.8575696018	0.2254920193
C	-0.8384888061	-3.4053230078	1.3524337443
C	0.6266509959	-3.6933502438	1.0019433802
C	-0.8265867106	-4.8138196521	-0.6635820513
H	-0.9587425158	-5.8443964607	-0.3260330731
H	-1.0764576459	-4.7552588881	-1.7247576953
C	-2.7345053923	3.4036206341	-0.8223519023
H	-3.6977028955	3.0522193737	-1.1784594839
C	-2.7586251916	4.3944880977	0.3493625967
H	-3.6044868371	4.5379146917	1.0066587339
C	-1.5602084922	4.9585413021	0.4119221459
H	-1.1902165542	5.6751820483	1.1318534027
C	-0.7163425414	4.3499058302	-0.7214455335
H	0.1911927852	4.8903973192	-0.9840028762
C	-1.8249128075	4.1976784633	-1.7907974680
H	-2.2544202692	5.1506817604	-2.0969153093
H	-1.5131255064	3.6202927743	-2.6632842036
H	1.4385687455	-4.7232952272	-0.8152481002
N	-3.4277450742	0.8324071950	0.3172410818
N	-3.0193346638	-1.4562087371	0.6587583222
N	4.3377823286	0.2373582252	1.0358055589
C	4.2418319953	-0.7689793654	2.1292614942
H	4.1312094231	-0.2572114517	3.0816054881
H	5.1587308992	-1.3531003651	2.1308923912
H	3.3935026554	-1.4179497611	1.9387027175
C	5.5457581151	1.0972310332	1.2288185954
H	5.6547306922	1.7684469076	0.3840427699
H	6.4235862916	0.4596628332	1.3088932790

H	5.4127090209	1.6759075295	2.1396772478
H	1.4395103661	-3.8320640187	1.7020810421
H	-1.2524482101	-3.2057238179	2.3296925260
H	4.4534159030	-0.3077862389	0.1672752406

8.3 Excited states

Compound **4**:

State	Energy [eV]	Osc. Strength [a.u.]
1	3.8094	0.3136
2	4.4796	0.0121
3	4.7687	0.0526
4	4.8289	0.0335
5	4.8718	0.0577
6	5.0147	0.0973
7	5.1478	0.0040
8	5.2825	0.0033
9	5.3714	0.1038
10	5.7824	0.0408
11	5.8899	0.0363
12	6.0992	0.1962
13	6.1487	0.0773
14	6.4208	0.0308
15	6.4417	0.0263
16	6.5316	0.0331
17	6.6279	0.0541
18	6.6963	0.0275
19	6.7227	0.0594
20	6.8105	0.0110

9

State	Energy [eV]	Osc. Strength [a.u.]
1	4.8604	0.0444
2	5.0242	0.2341
3	5.1234	0.0143
4	5.2490	0.0661
5	5.2870	0.0080
6	5.3409	0.0218
7	5.5072	0.0000
8	5.6361	0.0022
9	5.9077	0.0033
10	5.9541	0.0059

11	6.1684	0.0049
12	6.2391	0.0127
13	6.3320	0.0689
14	6.3982	0.0329
15	6.4663	0.0917
16	6.7622	0.0320
17	6.7926	0.0786
18	6.8372	0.0094
19	6.8981	0.1810
20	6.9575	0.0198

5

State	Energy [eV]	Osc. Strength [a.u.]
1	3.6995	0.1347
2	4.1065	0.2945
3	4.2361	0.0060
4	4.2958	0.0405
5	4.4634	0.0629
6	4.5191	0.0169
7	4.6801	0.0525
8	4.7980	0.0051
9	4.9088	0.0053
10	4.9460	0.0185
11	4.9850	0.0085
12	5.0734	0.1048
13	5.4067	0.0236
14	5.4221	0.0792
15	5.6317	0.0107
16	5.7098	0.0312
17	5.7450	0.0361
18	5.7704	0.0181
19	5.8609	0.0910
20	5.8929	0.0157

10

State	Energy [eV]	Osc. Strength [a.u.]
1	4.6750	0.0263
2	4.7885	0.1884
3	4.9859	0.0146
4	5.0477	0.0899
5	5.2100	0.0112
6	5.2673	0.0147
7	5.3045	0.0024

8	5.3587	0.0162
9	5.4182	0.0013
10	5.4835	0.0007
11	5.7281	0.0576
12	5.8657	0.0257
13	5.9645	0.0719
14	5.9741	0.2387
15	6.0700	0.1046
16	6.1252	0.0059
17	6.2126	0.0503
18	6.3410	0.0229
19	6.4296	0.0571
20	6.4573	0.0057

6

State	Energy [eV]	Osc. Strength [a.u.]
1	3.2888	0.5554
2	4.3520	0.0115
3	4.4841	0.0382
4	4.5583	0.0544
5	4.7637	0.0336
6	4.9557	0.0313
7	5.0455	0.1653
8	5.1425	0.0058
9	5.2994	0.0040
10	5.3607	0.0968
11	5.6181	0.2187
12	5.6865	0.1966
13	5.7948	0.1366
14	6.0605	0.0437
15	6.0972	0.0702
16	6.3353	0.0043
17	6.5397	0.0376
18	6.5556	0.0471
19	6.6013	0.0204
20	6.6646	0.0162

11

State	Energy [eV]	Osc. Strength [a.u.]
1	4.1904	0.0046
2	4.4836	0.0036
3	4.9269	0.0631

4	4.9386	0.2301
5	5.0803	0.0036
6	5.1810	0.0701
7	5.2858	0.0055
8	5.3108	0.0007
9	5.4118	0.0112
10	5.5718	0.5159
11	5.8179	0.0081
12	6.0389	0.0323
13	6.0976	0.0438
14	6.2370	0.0337
15	6.4308	0.1044
16	6.4546	0.0165
17	6.6977	0.0020
18	6.7598	0.0011
19	6.7769	0.0554
20	6.8056	0.0225

8

State	Energy [eV]	Osc. Strength [a.u.]
1	3.3080	0.2139
2	3.6640	0.0013
3	3.8159	0.7841
4	3.9680	0.0663
5	4.0639	0.0811
6	4.2276	0.1146
7	4.3279	0.0629
8	4.3501	0.0472
9	4.5125	0.0132
10	4.6418	0.0225
11	4.6528	0.0497
12	4.7308	0.0556
13	4.8019	0.0474
14	4.8243	0.0654
15	4.8325	0.1228
16	4.9285	0.0095
17	5.0958	0.0074
18	5.1358	0.0242
19	5.1687	0.0550
20	5.2746	0.0080

13

State	Energy [eV]	Osc. Strength [a.u.]

1	4.4457	0.0564
2	4.6066	0.0731
3	4.7297	0.0383
4	4.7660	0.0762
5	4.7859	0.0078
6	4.9711	0.0196
7	5.0486	0.0232
8	5.0879	0.0287
9	5.1290	0.0007
10	5.1973	0.0024
11	5.2928	0.0083
12	5.3162	0.3970
13	5.4089	0.0994
14	5.5052	0.0035
15	5.5442	0.2646
16	5.5618	0.0019
17	5.5797	0.0539
18	5.6206	0.1472
19	5.8005	0.2477
20	5.8293	0.1815

7

State	Energy [eV]	Osc. Strength [a.u.]
1	3.2215	0.1712
2	3.6174	0.0231
3	4.0166	0.5414
4	4.3217	0.0561
5	4.3976	0.0089
6	4.5564	0.0127
7	4.6282	0.0724
8	4.6705	0.0269
9	4.7139	0.0173
10	4.7696	0.0140
11	4.9006	0.0055
12	4.9427	0.0218
13	4.9988	0.1402
14	5.2138	0.1183
15	5.3659	0.0554
16	5.4323	0.0362
17	5.4847	0.0382
18	5.5992	0.0919
19	5.6629	0.0415
20	5.7138	0.0743

12

State	Energy [eV]	Osc. Strength [a.u.]
1	4.3426	0.0185
2	4.6444	0.0542
3	4.7361	0.0871
4	4.9252	0.0408
5	5.0056	0.0616
6	5.1262	0.0251
7	5.1726	0.0189
8	5.2087	0.0668
9	5.2713	0.0059
10	5.2993	0.0016
11	5.3352	0.0032
12	5.4891	0.3427
13	5.5986	0.0156
14	5.6603	0.0309
15	5.8305	0.0726
16	5.8476	0.0064
17	5.9105	0.0844
18	5.9554	0.0479
19	5.9636	0.1826
20	6.1607	0.0088

12b

State	Energy [eV]	Osc. Strength [a.u.]
1	3.5234	0.0005
2	3.8921	0.3001
3	4.3736	0.0148
4	4.5416	0.0748
5	4.6680	0.0089
6	4.6999	0.0619
7	4.8508	0.0020
8	4.9038	0.0122
9	4.9275	0.0266
10	5.0251	0.0699
11	5.0846	0.0029
12	5.1451	0.0034
13	5.3093	0.0017
14	5.3728	0.0542
15	5.4608	0.1062
16	5.4990	0.1518
17	5.5233	0.1592
18	5.7303	0.0608

19	5.8419	0.0212
20	5.8940	0.1057

12a

State	Energy [eV]	Osc. Strength [a.u.]
1	3.3432	0.4245
2	4.4168	0.0056
3	4.4843	0.0145
4	4.5285	0.0302
5	4.6574	0.0802
6	4.7397	0.0209
7	4.9244	0.0152
8	5.0771	0.0220
9	5.1230	0.1457
10	5.2345	0.0401
11	5.2909	0.1805
12	5.3437	0.0093
13	5.3568	0.0147
14	5.4236	0.0118
15	5.4963	0.1991
16	5.5386	0.0816
17	5.5575	0.0181
18	5.6559	0.0550
19	5.7774	0.0909
20	5.8067	0.0363

12c

State	Energy [eV]	Osc. Strength [a.u.]
1	3.8293	0.1108
2	4.1524	0.2664
3	4.3393	0.0017
4	4.4222	0.0008
5	4.5399	0.0086
6	4.6080	0.0021
7	4.6500	0.0045
8	4.6769	0.0724
9	4.7907	0.0159
10	4.9137	0.2248
11	4.9715	0.0104
12	5.1143	0.0050
13	5.3435	0.1596
14	5.4691	0.0099
15	5.5719	0.0187

16	5.6154	0.0242
17	5.6972	0.0186
18	5.7711	0.0533
19	5.7861	0.0011
20	5.8585	0.0465

12f

State	Energy [eV]	Osc. Strength [a.u.]
1	4.7881	0.0657
2	4.9670	0.0505
3	5.0375	0.1463
4	5.0862	0.0393
5	5.1297	0.0173
6	5.1399	0.0776
7	5.2267	0.0250
8	5.2752	0.0232
9	5.3169	0.1072
10	5.3488	0.1206
11	5.4447	0.0436
12	5.5767	0.1373
13	5.7153	0.0379
14	5.8697	0.0175
15	5.9279	0.0433
16	6.1300	0.0103
17	6.1634	0.0195
18	6.2260	0.0688
19	6.2714	0.0014
20	6.3409	0.0435

12d

State	Energy [eV]	Osc. Strength [a.u.]
1	4.0542	0.3073
2	4.4090	0.0058
3	4.6338	0.0304
4	4.6908	0.0149
5	4.7531	0.0598
6	5.0004	0.0031
7	5.0179	0.0706
8	5.1626	0.0203
9	5.2046	0.0139
10	5.2598	0.0127
11	5.3072	0.0480
12	5.3813	0.0825

13	5.5064	0.1300
14	5.6707	0.0051
15	5.7027	0.0085
16	5.7675	0.0363
17	5.8475	0.1035
18	5.9128	0.0391
19	5.9683	0.0659
20	6.0298	0.0573

12e

State	Energy [eV]	Osc. Strength [a.u.]
1	3.9388	0.1302
2	4.4927	0.0070
3	4.5424	0.0240
4	4.6857	0.0323
5	4.8162	0.1061
6	4.9557	0.0173
7	5.0692	0.0640
8	5.1531	0.0184
9	5.2148	0.1567
10	5.2833	0.0073
11	5.3024	0.1251
12	5.3833	0.0781
13	5.4897	0.0328
14	5.5337	0.1529
15	5.7360	0.0292
16	5.8481	0.0749
17	5.8928	0.0179
18	5.9605	0.0455
19	6.0543	0.0231
20	6.0770	0.0701

8.4 Attachment and detachment densities

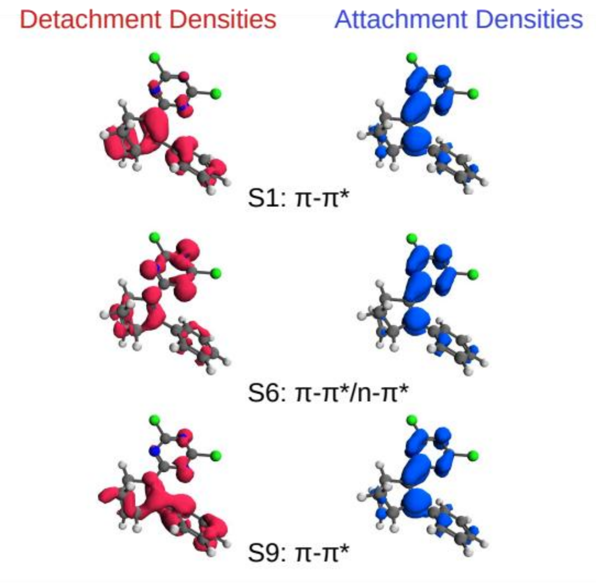

Figure S 228: Detachment/attachment (red/blue) densities of the important transitions of ***4****.*

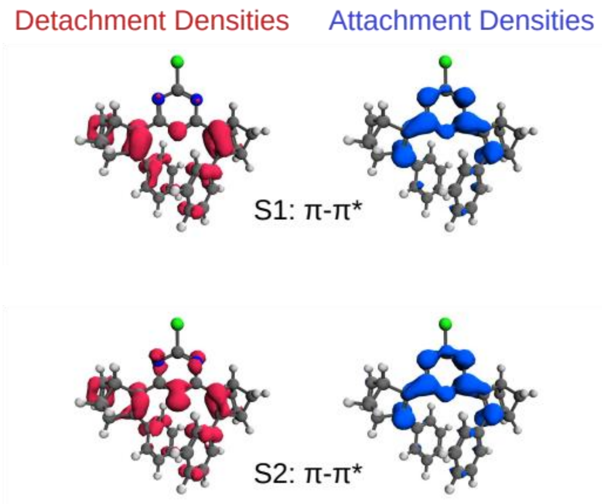

Figure S 229: Detachment/attachment (red/blue) densities of the important transitions of ***5****.*

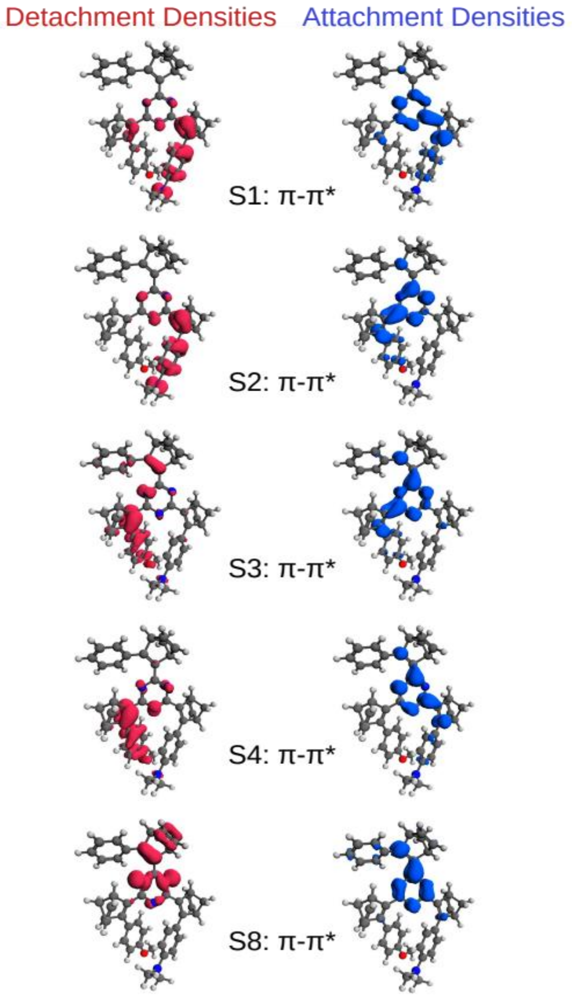

*Figure S 230: Detachment/attachment (red/blue) densities of the important transitions of **8**.*

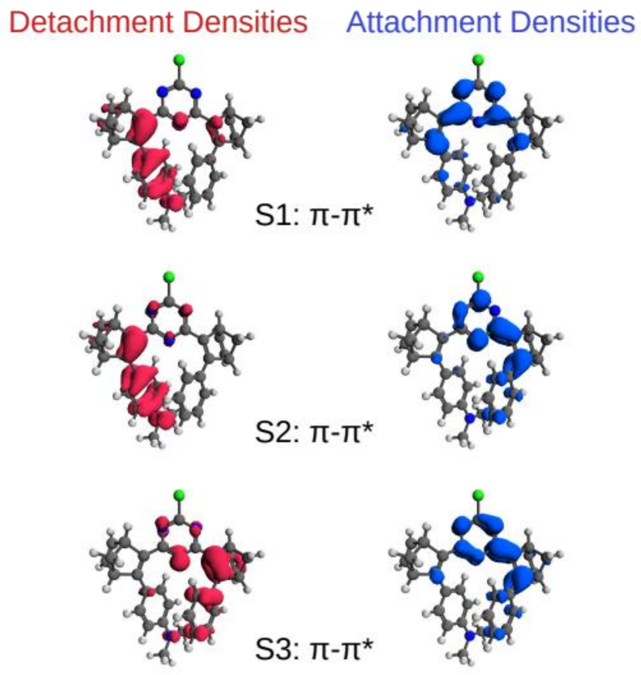

*Figure S 231: Detachment/attachment (red/blue) densities of the important transitions of **7**.*

9 References

[1] G. R. Fulmer, A. J. M. Miller, N. H. Sherden, H. E. Gottlieb, A. Nudelman, B. M. Stoltz, J. E. Bercaw, K. I. Goldberg, "NMR chemical shifts of trace impurities: Common laboratory solvents, organics, and gases in deuterated solvents relevant to the organometallic chemist" *Organometallics* **2010**, *29*, 2176–2179.

[2] D. Krappmann, A. Hirsch, "Synthesis, Characterization and Interconversion of p–Tolylsulfone-Functionalized Norbornadiene/Quadricyclane Couples" *Chem. – A Eur. J.* **2024**, *30*, e202401391.

[3] B. Gabriele, R. Mancuso, G. Salerno, M. Costa, "Cascade Reactions: Sequential Homobimetallic Catalysis Leading to Benzofurans and β,γ-Unsaturated Esters" *Adv. Synth. Catal.* **2006**, *348*, 1101–1109.

[4] R. Menicagli, S. Samaritani, V. Zucchelli, "2-Alkyl-4,6-dialkylamino-1,3,5-triazines via Grignard Alkylation of Cyanuric Chloride: An Aged Reaction Revisited" *Tetrahedron* **2000**, *56*, 9705–9711.

[5] J. E. Milne, S. L. Buchwald, "An extremely active catalyst for the Negishi cross-coupling reaction" *J. Am. Chem. Soc.* **2004**, *126*, 13028–13032.

[6] L. Zhang, L. Zou, J. Xiao, P. Zhou, C. Zhong, X. Chen, J. Qin, I. F. A. Mariz, E. MaçÔas, "Symmetrical and unsymmetrical multibranched D–π–A molecules based on 1,3,5-triazine unit: synthesis and photophysical properties" *J. Mater. Chem.* **2012**, *22*, 16781–16790.

[7] T. Luchs, P. Lorenz, A. Hirsch, "Efficient Cyclization of the Norbornadiene-Quadricyclane Interconversion Mediated by a Magnetic [Fe3O4–CoSalphen] Nanoparticle Catalyst" *ChemPhotoChem* **2020**, *4*, 52–58.

[8] P. Lorenz, T. Luchs, A. Hirsch, "Molecular Solar Thermal Batteries through Combination of Magnetic Nanoparticle Catalysts and Tailored Norbornadiene Photoswitches" *Chem. – A Eur. J.* **2021**, *27*, 4993–5002.

[9] C. M. Marshall, J. Molineux, K.-S. Kang, V. Kumirov, K.-J. Kim, R. A. Norwood, J. T. Njardarson, J. Pyun, "Synthesis of Polycyclic Olefinic Monomers from Norbornadiene for Inverse Vulcanization: Structural and Mechanistic Consequences" *J. Am. Chem. Soc.* **2024**, *146*, 24061–24074.

[10] V. A. Bren', A. D. Dubonosov, V. I. Minkin, V. A. Chernoivanov, "Norbornadiene–quadricyclane — an effective molecular system for the storage of solar energy" *Russ. Chem. Rev.* **1991**, *60*, 451–469.

[11] V. A. Bren, V. I. Minkin, A. D. Dubonosov, V. A. Chernoivanov, V. P. Rybalkin, G. S. Borodkin, "Biphotochromic Norbornadiene Systems" *Mol. Cryst. Liq. Cryst. Sci. Technol. Sect. A. Mol. Cryst. Liq. Cryst.* **1997**, *297*, 247–253.

[12] A. Dreos, Z. Wang, B. E. Tebikachew, K. Moth-Poulsen, J. Andréasson, "Three-Input Molecular Keypad Lock Based on a Norbornadiene-Quadricyclane Photoswitch" *J. Phys. Chem. Lett.* **2018**, *9*, 6174–6178.

[13] D. Krappmann, A. Hirsch, "Synthesis, Characterization and Interconversion of p-Tolylsulfone-Functionalized Norbornadiene/Quadricyclane Couples" *Chem. – A Eur. J.* **2024**, DOI 10.1002/chem.202401391.

[14] K. Stranius, K. Börjesson, "Determining the Photoisomerization Quantum Yield of Photoswitchable Molecules in Solution and in the Solid State" *Sci. Reports 2017 71* **2017**, *7*, 1–9.

[15] Z. Wang, A. Roffey, R. Losantos, A. Lennartson, M. Jevric, A. U. Petersen, M. Quant, A. Dreos, X. Wen, D. Sampedro, K. Börjesson, K. Moth-Poulsen, "Macroscopic heat release in a molecular solar thermal energy storage system" *Energy Environ. Sci.* **2019**, *12*, 187–193.

[16] C. A. Parker, "A new sensitive chemical actinometer. I. Some trials with potassium ferrioxalate" *Proc. R. Soc. London. Ser. A. Math. Phys. Sci.* **1953**, *220*, 104–116.

[17] C. G. Hatchard, C. A. Parker, "A new sensitive chemical actinometer - II. Potassium ferrioxalate as a standard chemical actinometer" *Proc. R. Soc. London* **1956**, *235*, 518–536.

[18] H. Volfova, Q. Hu, E. Riedle, "Determination of Reaction Quantum Yields: LED based setup with better 5 % Precision Henrieta" *EPA Newsl.* **2019**, 51.

[19] K. Börjesson, A. Lennartson, K. Moth-Poulsen, "Efficiency limit of molecular solar thermal energy collecting devices" *ACS Sustain. Chem. Eng.* **2013**, *1*, 585–590.

[20] M. Mansø, A. U. Petersen, Z. Wang, P. Erhart, M. B. Nielsen, K. Moth-Poulsen, "Molecular solar thermal energy storage in photoswitch oligomers increases energy densities and storage times" *Nat. Commun. 2018 91* **2018**, *9*, 1–7.

[21] E. Epifanovsky, A. T. B. Gilbert, X. Feng, J. Lee, Y. Mao, N. Mardirossian, P. Pokhilko, A. F. White, M. P. Coons, A. L. Dempwolff, Z. Gan, D. Hait, P. R. Horn, L. D. Jacobson, I. Kaliman, J. Kussmann, A. W. Lange, K. U. Lao, D. S. Levine, J. Liu, S. C. McKenzie, A. F. Morrison, K. D. Nanda, F. Plasser, D. R. Rehn, M. L. Vidal, Z.-Q. You, Y. Zhu, B. Alam, B. J. Albrecht, A. Aldossary, E. Alguire, J. H. Andersen, V. Athavale, D. Barton, K. Begam, A. Behn, N. Bellonzi, Y. A. Bernard, E. J. Berquist, H. G. A. Burton, A. Carreras, K. Carter-Fenk, R. Chakraborty, A. D. Chien, K. D. Closser, V. Cofer-Shabica, S. Dasgupta, M. de Wergifosse, J. Deng, M. Diedenhofen, H. Do, S. Ehlert, P.-T. Fang, S. Fatehi, Q. Feng, T. Friedhoff, J. Gayvert, Q. Ge, G. Gidofalvi, M. Goldey, J. Gomes, C. E. González-Espinoza, S. Gulania, A. O. Gunina, M. W. D. Hanson-Heine, P. H. P. Harbach, A. Hauser, M. F. Herbst, M. Hernández Vera, M. Hodecker, Z. C. Holden, S. Houck, X. Huang, K. Hui, B. C. Huynh, M. Ivanov, Á. Jász, H. Ji, H. Jiang, B. Kaduk, S. Kähler, K. Khistyaev, J. Kim, G. Kis, P. Klunzinger, Z. Koczor-Benda, J. H. Koh, D. Kosenkov, L. Koulias, T. Kowalczyk, C. M. Krauter, K. Kue, A. Kunitsa, T. Kus, I. Ladjánszki, A. Landau, K. V. Lawler, D. Lefrancois, S. Lehtola, R. R. Li, Y.-P. Li, J. Liang, M. Liebenthal, H.-H. Lin, Y.-S. Lin, F. Liu, K.-Y. Liu, M. Loipersberger, A. Luenser, A. Manjanath, P. Manohar, E. Mansoor, S. F. Manzer, S.-P. Mao, A. V. Marenich, T. Markovich, S. Mason, S. A. Maurer, P. F. McLaughlin, M. F. S. J. Menger, J.-M. Mewes, S. A. Mewes, P. Morgante, J. W. Mullinax, K. J. Oosterbaan, G. Paran, A. C. Paul, S. K. Paul, F. Pavošević, Z. Pei, S. Prager, E. I. Proynov, Á. Rák, E. Ramos-Cordoba, B. Rana, A. E. Rask, A. Rettig, R. M. Richard, F. Rob, E. Rossomme, T. Scheele, M. Scheurer, M. Schneider, N. Sergueev, S. M. Sharada, W. Skomorowski, D. W. Small, C. J. Stein, Y.-C. Su, E. J. Sundstrom, Z. Tao, J. Thirman, G. J. Tornai, T. Tsuchimochi, N. M. Tubman, S. P. Veccham, O. Vydrov, J. Wenzel, J. Witte, A. Yamada, K. Yao, S. Yeganeh, S. R. Yost, A. Zech, I. Y. Zhang, X. Zhang, Y. Zhang, D. Zuev, A. Aspuru-Guzik, A. T. Bell, N. A. Besley, K. B. Bravaya, B. R. Brooks, D. Casanova, J.-D. Chai, S. Coriani, C. J. Cramer, G. Cserey, A. E. DePrince, R. A. DiStasio, A. Dreuw, B. D. Dunietz, T. R. Furlani, W. A. Goddard, S. Hammes-Schiffer, T. Head-Gordon, W. J. Hehre, C.-P. Hsu, T.-C. Jagau, Y. Jung, A. Klamt, J. Kong, D. S. Lambrecht, W. Liang, N. J. Mayhall, C. W. McCurdy, J. B. Neaton, C. Ochsenfeld, J. A. Parkhill, R. Peverati, V. A. Rassolov, Y. Shao, L. V. Slipchenko, T. Stauch, R. P. Steele, J. E. Subotnik, A. J. W. Thom, A. Tkatchenko, D. G. Truhlar, T. Van Voorhis, T. A. Wesolowski, K. B. Whaley, H. L. Woodcock, P.

M. Zimmerman, S. Faraji, P. M. W. Gill, M. Head-Gordon, J. M. Herbert, A. I. Krylov, "Software for the frontiers of quantum chemistry: An overview of developments in the Q-Chem 5 package" *J. Chem. Phys.* **2021**, *155*, DOI 10.1063/5.0055522.

[22] T. Yanai, D. P. Tew, N. C. Handy, "A new hybrid exchange–correlation functional using the Coulomb-attenuating method (CAM-B3LYP)" *Chem. Phys. Lett.* **2004**, *393*, 51–57.

[23] R. Krishnan, J. S. Binkley, R. Seeger, J. A. Pople, "Self-consistent molecular orbital methods. XX. A basis set for correlated wave functions" *J. Chem. Phys.* **1980**, *72*, 650–654.

[24] S. Grimme, S. Ehrlich, L. Goerigk, "Effect of the damping function in dispersion corrected density functional theory" *J. Comput. Chem.* **2011**, *32*, 1456–1465.

[25] M. J. Kuisma, A. M. Lundin, K. Moth-Poulsen, P. Hyldgaard, P. Erhart, "Comparative Ab-Initio Study of Substituted Norbornadiene-Quadricyclane Compounds for Solar Thermal Storage" *J. Phys. Chem. C* **2016**, *120*, 3635–3645.
